# A foundation monograph of *Convolvulus* L. (Convolvulaceae)

**DOI:** 10.3897/phytokeys.51.7104

**Published:** 2015-06-18

**Authors:** John R.I. Wood, Bethany R.M. Williams, Thomas C. Mitchell, Mark A. Carine, David J. Harris, Robert W. Scotland

**Affiliations:** 1 Department of Plant Sciences, South Parks Road, University of Oxford, OX1 3RB, UK; 2 Department of Life Sciences, Natural History Museum, Cromwell Road, London SW7 5BD, UK; 3 Plant Biodiversity Research, Technische Universität München, Maximus-von-Imhof Forum 2, 85354 Freising, Germany; 4 Royal Botanic Garden Edinburgh, 20A Inverleith Row, Edinburgh EH3 5LR, UK; 5 Honorary Research Associate, Royal Botanic Gardens, Kew

**Keywords:** Convolvulaceae, global revision, lectotypification, monograph, morning glories, new species, new taxa

## Abstract

A global revision of *Convolvulus* L. is presented, *Calystegia* R.Br. being excluded on pragmatic grounds. One hundred and ninety species are recognised with the greatest diversity in the Irano-Turanian region. All recognised species are described and the majority are illustrated. Distribution details, keys to species identification and taxonomic notes are provided. Four new species, *Convolvulus austroafricanus* J.R.I.Wood & R.W.Scotland, **sp. nov**., *Convolvulus iranicus* J.R.I.Wood & R.W.Scotland, **sp. nov**., *Convolvulus peninsularis* J.R.I.Wood & R.W.Scotland, **sp. nov**. and *Convolvulus xanthopotamicus* J.R.I.Wood & R.W.Scotland, **sp. nov**., one new subspecies Convolvulus chinensis subsp. triangularis J.R.I.Wood & R.W.Scotland, **subsp. nov**., and two new varieties Convolvulus equitans var. lindheimeri J.R.I.Wood & R.W.Scotland, **var. nov**., Convolvulus glomeratus var. sachalitarum J.R.I.Wood & R.W.Scotland, **var. nov**. are described. *Convolvulus incisodentatus* J.R.I.Wood & R.W.Scotland, **nom. nov.**, is provided as a replacement name for the illegitimate *Convolvulus incisus* Choisy. Several species treated as synonyms of other species in recent publications are reinstated including *Convolvulus chinensis* Ker-Gawl., *Convolvulus spinifer* M.Popov., *Convolvulus randii* Rendle and *Convolvulus aschersonii* Engl. Ten taxa are given new status and recognised at new ranks: *Convolvulus namaquensis* (Schltr. ex. A.Meeuse) J.R.I.Wood & R.W.Scotland, **stat. nov**., Convolvulus hermanniae subsp. erosus (Desr.) J.R.I.Wood & R.W.Scotland, **stat. nov**., Convolvulus crenatifolius subsp. montevidensis (Spreng.) J.R.I.Wood & R.W.Scotland, **stat. nov.**, Convolvulus fruticulosus subsp. glandulosus (Webb) J.R.I.Wood & R.W.Scotland, **stat. nov.**, Convolvulus capituliferus subsp. foliaceus (Verdc.) J.R.I.Wood & R.W.Scotland, **stat. nov.**, Convolvulus hystrix subsp. ruspolii (Dammer ex Hallier f.) J.R.I.Wood & R.W.Scotland, **stat. nov**., Convolvulus hystrix subsp. inermis (Chiov.) J.R.I.Wood & R.W.Scotland, **stat. nov.**, Convolvulus rottlerianus subsp. stocksii (Boiss.) J.R.I.Wood & R.W.Scotland, **comb. et stat. nov**., Convolvulus calvertii subsp. ruprechtii (Boiss.) J.R.I.Wood & R.W.Scotland, **stat. nov.**, Convolvulus cephalopodus subsp. bushiricus (Bornm.) J.R.I.Wood & R.W.Scotland, **stat. nov**. The status of various infraspecific taxa is clarified and numerous taxa are lectotypified. This account represents a new initiative in terms of taxonomic monography, being an attempt to bring together the global approach of the traditional monograph with the more pragmatic and identification-focussed approach of most current floras while at the same time being informed by insights from molecular systematics.

## Introduction

The approach adopted in this study arises from a consideration of what taxonomists should be focussed on in the 21st century. Our knowledge of flowering plant diversity comes primarily from taxonomy that has accumulated piecemeal through studies that are geographically restricted. A limitation of this approach to the taxonomy of any widespread or sizeable group is that species usually comprise a combination of restricted endemic species, just over half of all species of flowering plant are single country endemics (WCSP 2014) and widespread species, and accurately delimiting and distinguishing species in these two categories demands a global perspective (Thomas et al. 2012). Extensive sampling across the entire range of a taxon’s geographical distribution is also important if substantial and existing levels of synonymy are to be accurately detected (Govaerts 2003; Scotland and Wortley 2003; Wortley and Scotland 2004).

In contrast to the geographical focus of floristic projects, monographic studies at a global level have been undertaken by individual botanists or teams of botanists (Thomas 1999; Thomas et al. 2012) and these studies are viewed as the ‘gold standard’ for achieving accurate species delimitation, effective identification keys and the optimal treatment of variation. Such monographs are usually associated with intensive studies of many aspects of plant systematics including phylogeny, conservation and anatomy, and in consequence tend to be few in number and mostly deal with relatively small plant groups. A potential way to make more rapid taxonomic progress with species-rich groups of plants is to combine the accuracy that comes from a global monographic treatment of plant variation with the more focused and practical aims of regional floristic projects.

To speed up the taxonomic process while retaining the extensive specimen sampling of a global monographic treatment we believe it is necessary to focus on species delimitation at the expense of other aspects of the traditional monograph. This can be achieved as long as the pragmatic and heuristic nature of taxonomy is appreciated. In other words, for many groups we can rapidly assess specimen level variation, write keys and delimit species without devoting large amounts of time to infra-specific levels of variation and hybridisation and similar issues. Our approach, however, is quite different from many traditional flora projects as we have made full use of electronic tools and technologies, DNA sequencing and basic phylogeny estimates for the group. We use the term “Foundation Monograph” for our approach.

This monograph of *Convolvulus* represents what can be achieved in a 12 month period with an experienced botanist working 75 per cent of the time on the taxonomy, a 3 month DNA barcoding project plus additional student input, and a team of four people meeting to discuss relevant issues and progress along the way.

### Our methodology and its implications

We faced two severe constraints in preparing the *Convolvulus* monograph: time and money. This meant that there was no opportunity for field work during the research for the monograph, no possibility of obtaining extensive loan material from a large number of herbaria and limited funds to visit other institutions. There was no time to carry out intensive studies into infraspecific taxa or study variation in populations of individual species over and above that evident from the herbarium samples available. However, we did have some advantages. The first was the proximity of the two major collections in London at Kew (K) and the Natural History Museum (BM), and the second rapid communication through e-mail and the sending of digital images from all over the world. Once the project was underway it became possible to make decisions which allowed us to maximise the benefits of the few visits and loans we required to see type material and a sufficient range of specimens of individual species to make taxonomic decisions. Thus visits were made to Edinburgh (E) and Vienna (W) because of their rich holdings in Middle Eastern material, to St Petersburg (LE) for its holdings from former Soviet Central Asia and to Paris for its collections from North Africa. All four institutions were additionally rich in type material. It would have been desirable to visit other herbaria, particularly Montpellier (MPU) and Geneva (G), but we had to work within budget and time constraints and nearly all the type material in these herbaria was duplicated elsewhere or could be viewed online. Small loans of up to six specimens in each case totalling about twenty five specimens were received from European herbaria (B, E, GOET, M, P, URT, W). Digital images from many herbaria (B, C, E, FI, FT, G, GOET, LISE, KW, M, MA, MPU, PRE, UPS) enabled us to see almost all type material. Australia and North America presented particular difficulties in species delimitation but we were able to receive more substantial loans of sufficient material from AD, BRI and TEX to facilitate more informed taxonomic decisions.

For species delimitation and the preparation of descriptions we examined specimens and made use of the available literature. Dissection of flowers and fruiting capsules was carried out where possible, but care was taken not to damage types or unusual material and capsules are rare or unknown in many species. In species delimitation we gave additional weight to authors who had extensive experience in the genus and knowledge of the species in the field as we were acutely aware that our field knowledge was limited to certain geographical areas. Thus, we have followed the treatment of (Johnson 2001) for *Convolvulus* in Australia in most particulars despite having some doubts about the characterisation of several species. However, in general we have made our own decisions based on the evidence of the material we have seen and believe that our broader approach from looking at the genus worldwide will provide useful insights. We would nevertheless emphasise that we do not believe in reinventing the wheel and we have made use of any insight available to us whether in the literature, on herbarium sheets, by personal observation or any other source, and, with permission, we have also made use of the illustrations that accompanied (Sa’ad 1967), which we believe will add to the utility of this work.

Our desire to produce a concise account of the genus in a limited time has of necessity resulted in the exclusion of certain elements common to many monographs. We have accepted subspecies and the more significant varieties but have accounted for all the recognised infraspecific taxa scattered through the literature down to the level of variety. Forms and subvarieties are not accounted for and are only cited when also treated as the basionym of a taxon of higher rank. For reasons of budget and time we have only seen and cited representative specimens of each species. To cite and map all known collections of each species would be an impossible task within our constraints. A glance at the *Flora of Iran* (Nowroozi 2002) or the *Flora of Southern Africa* (Meeuse and Welman 2000) will give an idea of how many records there are of each species in these two countries and how daunting a task comprehensive global distribution details would be. All that we would claim is the level of information we provide is at least comparable to that of *Flora Europaea* and similar to regional Floras and not much less than in most national floras.

### Generic delimitation

The use of the name *Convolvulus* predates Linnaeus by well over a century but the first formal description of the genus was in *Species Plantarum* (Linnaeus 1753) where 31 species were accepted. However, Linnaeus’ concept of the genus was very broad and contained elements which were early removed to other genera, namely *Argyreia* Lour., *Ipomoea* L. and *Evolvulus* L. The modern definition of the genus was essentially established by the end of the 18th century based on the filiform stigmas, axillary flowers subtended by small bracteoles and the bilocular ovary. Webb and Berthelot (1844) separated off some species from the Canary Islands as *Rhodorhiza* Webb on the basis of the short style, the abortion of one locule and the irregular dehiscence of the capsule. However, all these characters are found in other species of the genus, the short style correlating with a conical ovary. Consequently both Choisy (1845) and Peter (1891) included *Rhodorhiza* within *Convolvulus* and this decision is confirmed by modern molecular studies. Another group of species was separated off by Robert Brown (Brown 1810) as *Calystegia* and his decision was followed by most subsequent authors (Choisy 1845; O’Donell 1959; Sa’ad 1967) on the grounds of its large bracteoles, which are adjacent to the calyx, and different pollen grains (Hallier 1893). However, many North American authors never accepted this division and molecular studies do not support it.

Prior to beginning this monograph of *Convolvulus* we were aware that *Calystegia* was nested within *Convolvulus* and, therefore, *Convolvulus* as treated in this monograph is paraphyletic (Carine et al. 2004; Stefanovic et al. 2002). Molecular work completed as part of the monograph encompassed broader sampling of both genera and the data support the hypothesis that *Calystegia* is a monophyletic group nested within *Convolvulus* (Williams et al. 2014). *Calystegia* may be distinguished from *Convolvulus* by its pollen (polypantoaperturate versus equatorially triaperturate), stigmas (globose versus linear/clavate) and bracteoles that are large and inflated and enclose the calyx in *Calystegia* whereas in *Convolvulus* they are typically small and often remote, large bracteoles only occurring in *Convolvulus scammonia* and *Convolvulus pseudoscammonia*. Taxonomically, the European species of *Calystegia* have been treated by (Brummitt 1972) with molecular data supporting taxon circumscriptions proposed therein (Brown et al. 2009). Most of the diversity in *Calystegia* is distributed in North America and the taxa in this region have been treated by (Brummitt submitted) for the *Flora of North America*. Thus, *Calystegia* is a clearly defined subgroup within *Convolvulus* that has been treated at a more or less global-level by a single author. For these reasons we decided to pursue a pragmatic approach excluding *Calystegia* from this monograph of *Convolvulus*. For those who would consider all taxonomy should be based on monophyletic taxa, this issue is readily resolved by re-naming all species of *Calystegia* as *Convolvulus*. The necessary combinations are already available for most of the taxa concerned.

### Geographical distribution

Species of *Convolvulus* are distributed on all the main land masses of the world but the genus is most diverse in areas with a Mediterranean climate and in semi-desert regions of around the same latitude. It is principally a genus of dry, stony and sandy habitats from sea level to around 3000 m. In the tropics it is almost entirely absent within 15° of the equator except on mountains with one major exception–the Horn of Africa where there is a local centre of diversity. It is largely absent from smaller islands with two notable exceptions on either side of the African continent, Socotra to the east and the Canary Islands to the west, both of which are local centres of diversity in the genus. With the exception of the cosmopolitan weed, *Convolvulus arvensis*, the genus is absent at latitudes higher than 45°N. There are centres of diversity with similar number of species on all three major southern hemisphere land masses, Australia, southern Africa and South America. In the northern hemisphere, the genus is poorly represented in North America, where it is largely represented by *Calystegia* and is virtually absent from East Asia. Conversely it is most diverse in the Irano-Turanian regions of Central Asia with the greatest number of species being found in Iran.

### Discovery

The European species of *Convolvulus* were largely known by the end of the 18th century and only a few, very localised species, not all generally accepted, have been described subsequently, the most recent being *Convolvulus mairei* (Halacsy 1907), *Convolvulus suendermanii* (Bornmüller 1938), *Convolvulus sericocephalus* (Juzepczuk 1950), *Convolvulus argyrothamnos* (Greuter 1967) and *Convolvulus fernandesii* (Silva and Teles 1980). Most American and South African species were known by the middle of the 19th century and only a trickle of new species has been found subsequently in these areas, the most recent being *Convolvulus ensifolius* (Ferreira et al. 2013) in South America and *Convolvulus carrii* (Turner 2009) in North America. The travels and collections of Boissier, Kotschy and Aucher-Eloy showed that the Middle East was especially rich in *Convolvulus* and Boissier’s monumental *Flora Orientalis* (Boissier 1875) summarises their achievement by listing 66 species. Further exploration has confirmed that this region is the main centre of diversity in *Convolvulus* and the travels and publications of Bornműller, Davis and Rechinger amongst others have resulted in a steady increment to the number of species known from this region. The 50 years before the First World War revealed the presence of considerable *Convolvulus* diversity in North Africa and Central Asia. Outstanding among a number of important French collectors was Rene Maire, who during the course of a long career found new species of *Convolvulus* across North Africa from Libya to Morocco. At the same time the species of Central Asia were being discovered, with M.Popov completing the work begun by Schrenk, Regel and Schmalhausen. South Africa was known as a centre of diversity in *Convolvulus* in the early 19th century but only since the end of the 19th century has botanical exploration shown the Horn of Africa and the island of Socotra to be the only truly tropical centre of diversity in the genus. Recent publications (Sebsebe 1993; 1999; Thulin 2005) have added considerably to species numbers from this region. Very recently (Johnson 2001) has made considerable progress in unravelling the complex group of species found in Australia. It now seems unlikely that any new centres of diversity in the genus will be discovered.

We have treated 190 species of *Convolvulus* worldwide and do not believe that the final number of species is likely to much exceed 200 even after more intensive study. During the course of preparing this monograph only four new species have been described and only two of these were, in fact, first found in the last thirty years.

### Economic importance

*Convolvulus* is of relatively little economic importance. Several species are reported to be of importance for grazing in desert conditions including *Convolvulus oxyphyllus* and *Convolvulus pilosellifolius* in Arabia (Dickson 1955) and *Convolvulus hamadae*, *Convolvulus eremophilus*, *Convolvulus divaricatus* and Convolvulus dorycnium subsp. subhirsutus in Central Asia (Grigoriev 1953). Several species produce attractive flowers and are cultivated in suitable climates, most commonly *Convolvulus tricolor* and *Convolvulus valentinus* and, to a lesser extent, *Convolvulus althaeoides* and *Convolvulus cneorum*. The Canary Island species *Convolvulus floridus* and *Convolvulus scoparius* are sometimes planted, especially the former. Both species are the source of a fragrant oil obtained from the distillation of their roots and stem, known as Rhodium (oil) or rosewood (bois de rose). *Convolvulus scammonia* has roots that produce a pale brown exude used as a laxative, known as scammony. On the negative side, *Convolvulus arvensis* is a widespread and persistent weed, which is difficult to eradicate once established and has a considerable negative economic impact. Other species are occasionally reported as weeds, such as *Convolvulus pilosellifolius*, which can occur amongst cotton (Grigoriev 1953).

### Molecular systematics

Molecular data formed an integral component of our approach. Silica gel dried (10%) and herbarium (90%) material were used to sample 130 species of *Convolvulus* and 18 species of *Calystegia* for the plant barcoding regions *mat*K and *rbc*L (CBOL Plant Working Group 2009), together with the nuclear ribosomal Internal Transcribed Spacer region (ITS) that has been proposed as a third barcoding marker (China Plant BOL Group 2011). For fifty-one taxa, data was obtained from one or more of the markers for two or more accessions. Whilst a high level of sampling was achieved overall, sampling of taxa in central Asia was limited, reflecting the lack of suitable material for molecular work for many of the species endemic to this region.

Molecular data were used in conjunction with morphology to resolve species delimitation in a number of complexes and to inform decisions regarding infrageneric classification and the systematic order used in the taxonomic treatment (Williams et al. 2014).

At the species level, molecular data supports the distinction of a number of geographically separate but morphologically similar species pairs such as *Convolvulus althaeoides* (Mediterranean) and *Convolvulus capensis* (South African) and *Convolvulus demissus* (South American) and *Convolvulus sagittatus* (African). Molecular data also support the separation of *Convolvulus chinensis* from *Convolvulus arvensis* and of *Convolvulus aschersonii* and *Convolvulus namaquensis* from *Convolvulus sagittatus* (Williams et al. 2014). Distinct clades were resolved within *Convolvulus lineatus* and *Convolvulus oxysepalus* by DNA sequence data. However, we were unable to correlate these infraspecific groupings with morphology or geography. Further work may be necessary to accurately delimit these widespread and morphologically variable taxa. We have discussed this in the text under each individual species.

**Figure 1.**
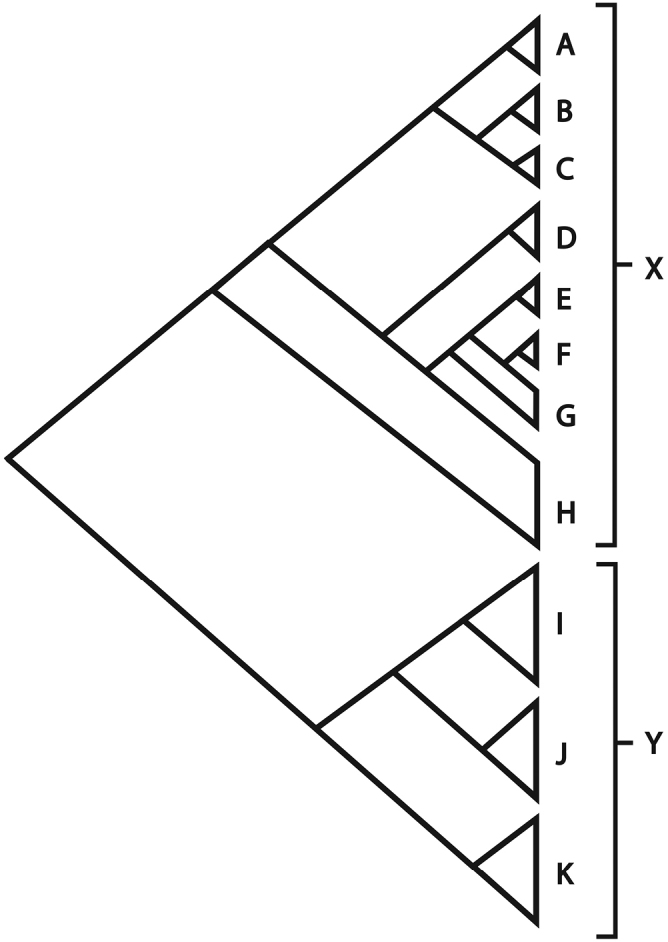
Summary cladogram showing major groupings resolved in *Convolvulus* based on a phylogenetic analysis of 140 species of *Convolvulus* and 19 species of *Calystegia* analysed for matK, rbcL and ITS. For details of clades, see text.

The phylogenetic and biogeographic implications of the results are discussed elsewhere (Williams et al. 2014). However, a summary cladogram is provided in Figure 1. The results concur with those of (Carine et al. 2004) in highlighting the non-monophyly of infrageneric groups proposed originally by (Boissier 1875) and elaborated by Peter (1891), Petrov (1935) and Sa’ad (1967) and in recognising two major clades within the genus (Figure 1, Clades X and Y). Clade X comprises species that are mostly annual or perennial herbs, sometimes woody at the base and typically with a trailing or climbing habit and leaves that are distinctly petiolate. *Calystegia* (Clade D) is resolved in this clade. Clade Y mostly comprises erect shrubs, with indistinct petioles.

Within Clade X, there is a strong geographical signal with Australasian (Clade E) and South American (Clade F) clades of *Convolvulus* resolved within a paraphyletic tropical African grade G. Other groups resolved within Clade X include subclade C, comprising blue-flowered annuals that are largely Mediterranean in distribution but include also *Convolvulus simulans* from North America. Taxa in this clade do not exhibit the distinctly petiolate leaves typical of Clade X more generally. Subclade B, comprises western Mediterranean and Macaronesian taxa (the latter exhibiting a woody habit), many of which have blue flowers, and clade A, centred on the Red Sea, is a morphologically diverse assemblage of species including taxa with dense capitulate inflorescences, blue flowers and/or clavate stigmas. Three species of *Convolvulus* (*Convolvulus kossmatii*, *Convolvulus semhaensis* and *Convolvulus socotranus*) previously treated in *Seddera* (Sebsebe and Mill 2009) were recently transferred to *Convolvulus* on the basis of molecular sequence data (Luna et al. 2014). They are resolved in this clade and exhibit the clavate stigmas found in a number of members of the group. The remaining taxa in Clade X constitute a grade of Eurasian taxa (H) that includes *Convolvulus arvensis*.

Clade Y is restricted to the Eurosiberian region and three major subgroups are resolved within it. The first (Clade I) comprises species that are often fastigiate in habit and have flowers that are not aggregated into heads. The second (Clade J) comprises species that are typically subshrubs with sericeous leaves, pubescent seeds and often a spiny habit. The third clade (Clade K) comprises species with leaves that are typically tomentose and flowers that are borne in heads.

Whilst some attempt has been made above to correlate morphological traits with the clades resolved in the molecular analysis, it should be noted that the fit is imperfect. We have been unable to identify unique, un-reversed morphological synapomorphies for any of the groups highlighted in Figure 1 with the notable exception of *Calystegia*, a situation that reflects a more widespread problem in Convolvulaceae wherein high levels of homoplasy have rendered super-specific classification problematic (Austin 1998; Manos et al. 2001). Given the nature of the morphological variation, we are unable to propose an adequate infrageneric classification (Carine and Scotland 2002).

As noted above, there were pragmatic grounds for excluding *Calystegia* from this treatment. Nevertheless, the molecular data do support the inclusion of *Calystegia* within *Convolvulus*, in agreement with Carine et al. (2004) and Stefanovic et al. (2002).

The molecular results have been used to establish a framework for the linear sequence of species adopted in the taxonomic account. Efforts have been made to establish an order that maximises morphological similarity between adjacent species within the constraints of relationships inferred using molecular data. Species that are not sampled for molecular data were placed on the basis of their morphology. Within major groups, the molecular sequence has occasionally been abandoned so as to place species which are known to hybridise next to each other or to place species with close morphological similarity together. The sequence used is as follows:

Old world species with petiolate leaves and mostly twining/trailing stems (Grade H)

Southern African species (Grade G)

New world species (Clade F)

Australasian species (Clade E)

North west African (mostly) and Canary Island species (Clade B)

Annual mostly blue-flowered species (Clade C)

Red Sea group, often with bluish flowers and/or clavate stigmas but very diverse in habit (Clade A)

Old world species with mostly separate flowers, often fastigiate in habit, not spiny (Clade I)

Old world species with sericeous leaves, often undershrubs, sometimes spiny (Clade J)

Old world species with flowers in heads, leaves commonly tomentose, rarely spiny (Clade K)

### Species concept

The species concept is problematic in *Convolvulus*. While there are a small number of species that are outstandingly distinct (*Convolvulus persicus* from the Caspian and Black Seas, *Convolvulus floridus* from the Canary Islands, *Convolvulus assyricus* from Turkey and *Convolvulus kilimandschari* from East Africa are good examples) and many that are perfectly adequately delimited although having close relatives, there are a large number of species clusters where the boundaries of individual species are far from satisfactory. This will be apparent in any region where *Convolvulus* is reasonably diverse, whether South America, Australia, Central Asia, the Canary Islands, Southern Africa, the Horn of Africa, Arabia, Turkey or elsewhere. It is apparent in the long synonomies of several species such as *Convolvulus althaeoides*, *Convolvulus sagittatus* and *Convolvulus prostratus*. It is also apparent in the many species that historically have been subsumed in other species such as *Convolvulus libanoticus*, *Convolvulus mazicum* and *Convolvulus subsericeus* in *Convolvulus cantabrica* or the inclusion of all Australian species in *Convolvulus erubescens*. It can also be seen in the placement of a number of infraspecific taxa which have been moved by different authors from one species to another, such as var. melliflorus which has moved between *Convolvulus valentinus* and *Convolvulus supinus*.

Notes are provided to indicate where plants intermediate between two species are known to occur. The status of these intermediates is rarely known with certainty. Hybrids are only well documented in a few cases (Carine et al. 2007, Aykurt and Sümbül 2011b, for example) and may be common, but this cannot be confirmed at the present time. Clearly detailed population studies and experimental work may clarify uncertainties over the years, but quite obviously evolution of species is taking place both in the island context of the Canaries and Socotra and across continents. We have tried to provide solutions to these problems on a consistent and pragmatic basis and the notes indicate where we have had difficulties. We have accepted as a ‘good’ species any that is distinct over most of its range and have indicated when intermediates are known. A very strict species concept could result in a series of species collapsing into each other ending in a counter-intuitive and uninformative taxonomy. In any case it is not clear in most cases whether these intermediates are hybrids or not. We have tried to avoid excessive resort to subspecies and varieties, but these ranks are necessary in quite a few cases.

An important and perhaps surprising problem in species delimitation is that a considerable number of species are only known from a handful of collections and in several cases from the type only. Moreover, many specimens are inadequate. Only in a few cases do we know what the rootstock is like and in many species the capsule and seeds are unknown. This makes any taxonomy based on capsule and seed characters difficult to formulate and, where it has been attempted, difficult to use or evaluate, as in the case of the Australian species. Good field observations are also lacking in many cases. We do not know the flower colour or the potential height of many species as these details are often not recorded.

**Figure 2.**
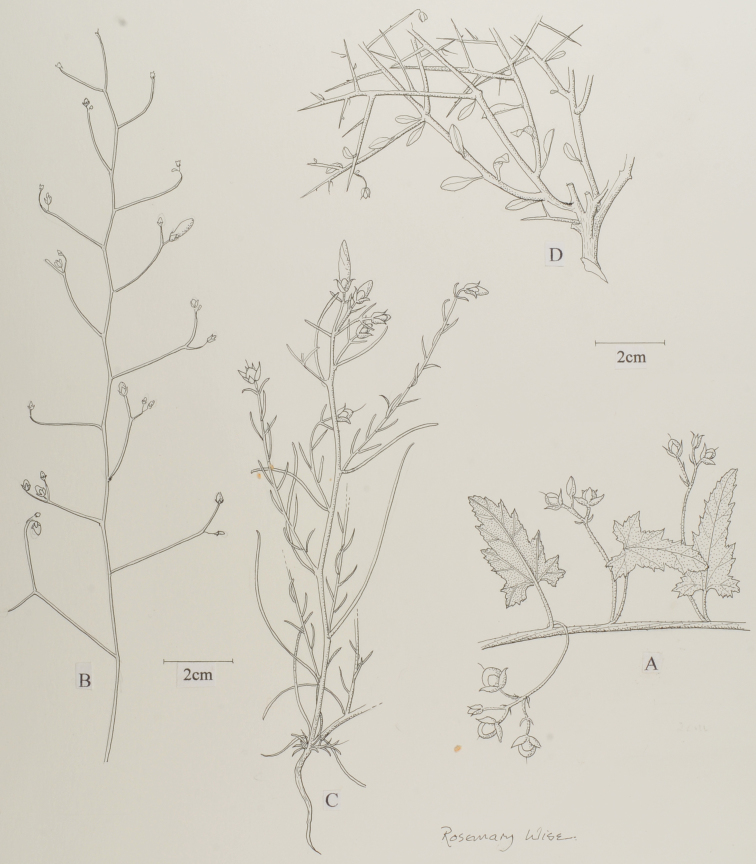
Habits of the four new species described in this treatment. **A** *Convolvulus austroafricanus* **B** *Convolvulus peninsularis* **C** *Convolvulus xanthopotamicus* **D** *Convolvulus iranicus* **A** from Fanshawe 6566 (K) **B** from *Whitcombe* 807 (E) **C** from *Purdom* s.n. (K) **D** from *Alava & Bokhari* 10629 (W).

Very few new species are described in this monograph. This is mainly a reflection of the fact that *Convolvulus* is an essentially temperate genus which has been well-studied over the years. We have avoided describing new species based on single collections, however odd, which belong to a species complex. *Santos* 544 from Angola is unmatched by any other collection but is clearly part of a complex group of species centred on *Convolvulus sagittatus*, so has been noted but not described. The same is true of *Bramwell & Humphries* 3448 from the Canary Islands. Conversely three species, *Convolvulus iranicus*, *Convolvulus xanthopotamicus* and *Convolvulus peninsularis* have been described because their relationships are clear and they separate from related species by one or more ‘strong, qualitative’ characters. We have, however, described one new species from a species complex, *Convolvulus austroafricanus*, but in this case there are a good number of specimens and it has a distinct geographical range (Figure 2).

For the reasons expressed in the previous paragraphs it should be emphasised that the status of a number of species remains uncertain until further collections and more detailed study in the field is possible. We are not completely convinced that *Convolvulus lindbergii* is distinct from *Convolvulus eremophilus* or *Convolvulus rectangularis* from *Convolvulus lanjouwii* or *Convolvulus semhaensis* from *Convolvulus kossmatii* or that *Convolvulus jordanensis*, *Convolvulus spicatus*, *Convolvulus schimperi* and *Convolvulus cephalopodus* merit recognition as separate species. Other examples could be found from Australia, America and elsewhere. In contrast, *Convolvulus sagittatus* is so variable that it is quite possible that some populations merit recognition as separate species but intensive genetic studies are clearly needed within this complex to explain the variation and introgression between species and forms.

At the infraspecific level, we have utilised the ranks of subspecies and variety:

The rank of **subspecies** is used to separate two closely related and usually intergrading taxa which are geographically separated but may have an overlapping range. Usually they are separated by one or two characters. Characteristically they behave as distinct species through much of their range.

The rank of **variety** is used for a distinct infraspecific taxon which is either known from a single location or group of close-by locations within the broader range of the species or occurs sporadically over the whole or, at least, most of the range of the species but with no obvious geographical patterning.

### Morphological characters and their use in species delimitation

During our studies we have noted a range of characters of use in species delimitation. Their value is discussed below and we have provided lists of distinctive features, which may be of use in identification. It should be noted that the lists are not always exhaustive as information is not available for all species and not all useful characters are clearly defined.

Habit. Species of *Convolvulus* may be herbs or undershrubs. Herbs may be perennial or annual, entirely herbaceous or somewhat woody below, twining, trailing or more or less erect. Undershrubs may be cushion-forming, fastigiate, liana-like, erect and unarmed; or low compact and spiny; or low, compact and unarmed. Habit is therefore of great use in species delimitation and some of these distinctive habits are geographically restricted.

All southern hemisphere species are perennial trailing or twining herbs with cordate, hastate or sagittate, petiolate leaves with the partial exceptions of *Convolvulus hasslerianus* and *Convolvulus randii* which are often (but apparently not always) erect herbs. In the northern hemisphere there is much greater diversity of life form and habit although trailing perennial herbs similar in habit to southern hemisphere species are well-represented. Amongst the distinct forms are:

- Annual herbs: *Convolvulus fatmensis*, *Convolvulus coelesyriacus*, *Convolvulus siculus*, *Convolvulus pentapetaloides*, *Convolvulus simulans*, *Convolvulus gharbensis*, *Convolvulus humilis*, *Convolvulus tricolor*, *Convolvulus meonanthus*, *Convolvulus rhyniospermus*, *Convolvulus rottlerianus*. *Convolvulus rhyniospermus* may occasionally perenniate. Several Australian species regarded as perennials may be (at least sometimes) annual. These include *Convolvulus crispifolius*, *Convolvulus eyreanus* and *Convolvulus recurvatus*. *Convolvulus capituliferus* and *Convolvulus grantii* have been interpreted as annuals but appear always to be perennial. None of the annuals are obviously twiners. They are characteristically slender, entirely herbaceous and with a poorly developed rootstock. Many have recurved fruiting peduncles.
- Erect (or at least ascending) herbs: *Convolvulus pseudoscammonia*, *Convolvulus hasslerianus* (apparently sometimes twining), *Convolvulus randii* (sometimes twining).
- Trailing or twining herbaceous perennials, which may be woody below, with hastate, sagittate or truncate leaves: *Convolvulus scammonia*, *Convolvulus durandoi*, *Convolvulus arvensis*, *Convolvulus mairei*, *Convolvulus chinensis*, *Convolvulus fatmensis*, *Convolvulus steppicola*, *Convolvulus sinuatodentatus*, *Convolvulus rufescens*, Convolvulus galaticus, *Convolvulus germaniciae*, *Convolvulus cassius*, *Convolvulus betonicifolius*, *Convolvulus longipedicellatus*, *Convolvulus stachydifolius*, *Convolvulus althaeoides*, *Convolvulus palaestinus*, *Convolvulus maireanus*, *Convolvulus pitardii*, *Convolvulus glaouorum*, *Convolvulus vidalii*, *Convolvulus lanjouwii*, *Convolvulus rectangularis*, *Convolvulus dryadum*, *Convolvulus supinus*, *Convolvulus sabatius*, *Convolvulus valentinus*, *Convolvulus vollesenii*, *Convolvulus bidrensis*, *Convolvulus jefferyi*, *Convolvulus capituliferus*, *Convolvulus stenocladus*, *Convolvulus subspathulatus*, *Convolvulus grantii*, *Convolvulus sarmentosus* (perhaps), all American and southern hemisphere species except *Convolvulus simulans* (annual), *Convolvulus kilimandschari* (liana), *Convolvulus hasslerianus* and *Convolvulus randii* (both usually erect herbs). Most of these species seem to be normally trailing herbs but a few show strong evidence of usually being twiners although this might be an artifice of their preferred habitat. Common twining species include *Convolvulus farinosus* from Africa and *Convolvulus remotus* from Australia.
- Lianas: *Convolvulus canariensis*, *Convolvulus massonii*, *Convolvulus volubilis*, *Convolvulus lopezsocasii*, *Convolvulus fernandesii*, *Convolvulus kilimandschari*.
- Unarmed non-twining, non-fastigiate shrubs: *Convolvulus persicus*, *Convolvulus floridus*, *Convolvulus scindicus*, *Convolvulus fruticulosus*. The first three are commonly erect whereas the last is more or less prostrate in form.
- Erect unarmed undershrubs with stiff, thin, woody, subfastigiate branches and (usually) few, relatively small leaves: *Convolvulus scoparius*, *Convolvulus scopulatus*, *Convolvulus socotranus*, *Convolvulus sericophyllus*, *Convolvulus hildebrandtii*, *Convolvulus peninsularis*, *Convolvulus leptocladus*, *Convolvulus eremophilus*, *Convolvulus lindbergii*, *Convolvulus hamadae*, *Convolvulus erinaceus*, *Convolvulus divaricatus*, *Convolvulus tujuntauensis*, *Convolvulus subsericeus*, *Convolvulus chondrilloides*, *Convolvulus gracillimus*, *Convolvulus kurdistanicus*, *Convolvulus koieanus*, *Convolvulus sarothrocladus*, *Convolvulus pseudocantabrica*, *Convolvulus dorycnium*.
- Low cushion plants with branched woody rootstock: *Convolvulus assyricus*, *Convolvulus mazicum*, *Convolvulus boissieri*, *Convolvulus suendermannii*, *Convolvulus libanoticus*, *Convolvulus carduchorum*, *Convolvulus cataonicus*, *Convolvulus phrygius*, *Convolvulus aitchisonii*, *Convolvulus asyrensis*, *Convolvulus ammannii*, *Convolvulus tragacanthoides*. These intergrade with species like *Convolvulus lineatus*, *Convolvulus calvertii* and *Convolvulus holosericeus*, which may be cushion-like in form but with erect stems.
- Spiny undershrubs can be divided into three subgroups:
  - Only the old lower branchlets spinescent: *Convolvulus lanatus*, *Convolvulus tragacanthoides*, *Convolvulus grigorjevii*.
  - All (or most) branches spinescent: *Convolvulus kossmatii*, *Convolvulus semhaensis*, *Convolvulus caput-medusae*, *Convolvulus oxyphyllus*, *Convolvulus ulicinus*, *Convolvulus spinifer*, *Convolvulus virgatus*. In all of these except *Convolvulus virgatus* the flowers are sessile. A few other species have sometimes been interpreted as having spinescent branches including *Convolvulus turrillianus*, *Convolvulus oxysepalus*, *Convolvulus erinaceus*, *Convolvulus hamadae* and *Convolvulus sericophyllus* and a few others are rarely subspinescent, such as some forms of *Convolvulus prostratus*.
  - Branches and peduncles (where present) spinescent, sterile lateral spines (formed from abnormal sterile peduncles?) also present: *Convolvulus fruticosus*, *Convolvulus gortschakovii*, *Convolvulus spinosus*, *Convolvulus argyracanthus*, *Convolvulus acanthocladus*, *Convolvulus iranicus*, *Convolvulus leiocalycinus*, *Convolvulus verdcourtianus*, *Convolvulus trabutianus*.

Underground parts. These are poorly known. *Convolvulus arvensis* is well-known for producing extensive underground rhizomes, which explain its persistence as a weed of cultivation. Many herbaceous species put down a shallow tap root, from which thin adventitious side roots develop. The perennial species of desert and semi desert regions commonly have a thickened woody rootstock which allows survival in the long periods between rain and from which annual, herbaceous or near herbaceous stems arise. *Convolvulus hasslerianus* and perhaps *Convolvulus randii* have a xylopodium which allows them to survive savannah fires.

Leaf and stem indumentum. There is much variation in indumentum, with the majority of species hairy in some form. Sa’ad (1967: 25 ff.) drew attention to different hair structures in *Convolvulus* but she did not make use of this in species delimitation and we have made no use of it either. Instead, we have indicated below some indumentum features which we have found of taxonomic importance. In the majority of species the indumentum of the leaves, stem, bracts, bracteoles and sepals is similar although varying somewhat in density. However, in a few species the indumentum of the sepals (and occasionally also that of the bracteoles) is strikingly different to that of the leaves. Reference in this part is consequently to the indumentum of the leaves and stem unless otherwise indicated. It should also be noted that some species are glabrescent, the older parts glabrous while the younger parts are hirsute to some degree.

- Leaves velvety-tomentose: *Convolvulus galaticus*, *Convolvulus germaniciae*, Convolvulus althaeoides subsp. tenuissimus, *Convolvulus eyreanus*, *Convolvulus crispifolius*, *Convolvulus thomsonii*. The distinction between this and the next category is not very clear.
- Leaves densely sericeous/canescent: *Convolvulus lanuginosus*, *Convolvulus cneorum*, *Convolvulus krauseanus*, *Convolvulus lineatus*, *Convolvulus oleifolius*, *Convolvulus argyrothamnos*, *Convolvulus holosericeus*, *Convolvulus calvertii*, *Convolvulus ammannii*, *Convolvulus xanthopotamicus*, *Convolvulus tragacanthoides*, *Convolvulus spinifer*, *Convolvulus grigorjevii*, *Convolvulus fruticosus*, *Convolvulus hermanniae*, *Convolvulus carrii*, *Convolvulus randii*, *Convolvulus lindbergii*, *Convolvulus boissieri*, *Convolvulus suendermannii*, *Convolvulus caput-medusae*, *Convolvulus mazicum*.
- Very finely sericeous and often somewhat glabrescent: *Convolvulus sericophyllus*, *Convolvulus kossmatii*, *Convolvulus semhaensis*, *Convolvulus gracillimus*, *Convolvulus vollesenii*, *Convolvulus subspathulatus*, *Convolvulus jefferyi*.
- Villous-tomentose perennials (dense longish hairs), characteristically woody at the base but with more or less herbaceous branches (always associated with flowers in heads): *Convolvulus aitchisonii*, *Convolvulus asyrensis*, *Convolvulus lanatus*, *Convolvulus secundus*, *Convolvulus spicatus*, *Convolvulus schimperi*, *Convolvulus jordanensis*, *Convolvulus cephalopodus*, *Convolvulus euphraticus*, *Convolvulus reticulatus*, *Convolvulus cephalophorus*, *Convolvulus stapfii*, *Convolvulus kotschyanus*, *Convolvulus pyrrotrichus*, *Convolvulus prostratus*, *Convolvulus pilosellifolius*, *Convolvulus calvertii*, *Convolvulus elymaiticus*, *Convolvulus commutatus*, *Convolvulus schirazianus*.
- Plants glabrous or nearly so (several of these are sometimes partially puberulent): *Convolvulus scammonia*, *Convolvulus pseudoscammonia*, *Convolvulus durandoi*, *Convolvulus arvensis*, *Convolvulus chinensis*, *Convolvulus lopezsocasii*, *Convolvulus demissus*, *Convolvulus laciniatus*, *Convolvulus montanus*, *Convolvulus ensifolius*, *Convolvulus microsepalus*, *Convolvulus remotus*, Convolvulus angustissimus, *Convolvulus waitaha*, *Convolvulus dregeanus*, *Convolvulus bidentatus*, *Convolvulus namaquensis*.
- Sepal indumentum strikingly different from that of stem and leaves: *Convolvulus acanthocladus*, *Convolvulus hamrinensis*, *Convolvulus oxyphyllus*, *Convolvulus ulicinus*, *Convolvulus urosepalus*, *Convolvulus iranicus*, *Convolvulus oxysepalus*, *Convolvulus turrillianus*, *Convolvulus virgatus*, *Convolvulus glomeratus*, *Convolvulus scopulatus*, *Convolvulus sericocephalus*, Convolvulus boissieri subsp. compactus, *Convolvulus cephalopodus* (not as strongly as in other species).

Leaves and bracts. In this treatment, no great distinction is generally made between bracts and leaves. In the great majority of cases the leaves which subtend flowering peduncles (i.e. bracts) are almost identical to the lower stem leaves and differ only in their progressively smaller size towards the apex of the stem. Only in a few cases, principally those species where the inflorescence appears terminal, have we made a clear distinction between leaves and bracts.

In terms of leaf shape and molecular phylogeny the genus can be divided into two main natural groups, which are mostly easily separated apart from a few Somali or Socotran species. The first group (Species 1–106) has leaves with cordate, truncate, sagittate or hastate leaf bases and clearly demarcated petioles; basal leaf auricles are frequent. Leaves may be entire, undulate, crenate, dentate or sinuate-lobed. Included are a small number of petiolate species with rounded to broadly cuneate leaf bases, which include *Convolvulus persicus* and a few species from East Africa including *Convolvulus oppositifolius*, *Convolvulus rhyniospermus*, *Convolvulus capituliferus*, *Convolvulus stenocladus*, *Convolvulus jefferyi*, *Convolvulus bidrensis*. All trailing and twining species belong to this first group. The second group (Species 107–190) have leaves that are gradually narrowed at base and lack a distinct petiole. This group is most diverse in Central Asia and does not occur in the southern hemisphere or the Americas. It includes all cushion plants, most spiny species, most species with a sericeous or canescent indumentum and all species of a fastigiate habit. Leaves are usually linear, oblanceolate, oblong or elliptic and are usually entire, although undulate leaves occur in some fastigiate species and especially in the herbaceous *Convolvulus grantii*.

Amongst unusual leaf features are

- Leaves abruptly contracted to a subsessile base: *Convolvulus hasslerianus*, *Convolvulus ensifolius*, *Convolvulus hystrix*, *Convolvulus ocellatus*.
- Twining/trailing plants with strongly dimorphic leaves include most Australasian species and the following but the list is not exhaustive: *Convolvulus althaeoides*, *Convolvulus palaestinus*, *Convolvulus glaouorum*, *Convolvulus vidalii*, *Convolvulus dregeanus*, *Convolvulus sagittatus* (sometimes), *Convolvulus capensis* (?), *Convolvulus chilensis*, *Convolvulus equitans*, *Convolvulus maireanus*, *Convolvulus assyricus*, *Convolvulus grantii*.
- Leaf auricles bifurcate or otherwise divided: *Convolvulus chilensis*, *Convolvulus bonariensis*, *Convolvulus equitans*, *Convolvulus aschersonii*, *Convolvulus steppicola*, *Convolvulus chinensis*.
- Leaves equilaterally triangular in form: *Convolvulus scammonia*, Convolvulus chinensis subsp. triangularis, *Convolvulus dryadum*, *Convolvulus farinosus* (commonly).

Inflorescence. Inflorescence structure is quite diverse in the genus and is of taxonomic importance. Flowers are arranged in axillary cymes but this structure is not always obvious. Most commonly, cymes are clearly pedunculate with paired bracteoles at the branching point(s). In this account the peduncle is measured from the base where it arises from the main stem to this branching point. The term pedicel is used above this point. Pedicels are often very variable in length on a single inflorescence especially when the cyme has a clearly monochasial structure. Bracteoles are mostly small, sometimes caducous and relatively unimportant in distinguishing species.

- Inflorescence terminal: *Convolvulus turrillianus*, *Convolvulus oxysepalus*, *Convolvulus scindicus*, *Convolvulus maireanus*, *Convolvulus calvertii*, *Convolvulus commutatus*, *Convolvulus schirazianus*, *Convolvulus elymaiticus*, *Convolvulus lineatus*, *Convolvulus xanthopotamicus*, *Convolvulus spinifer*, *Convolvulus grigorjevii*, *Convolvulus krauseanus*, *Convolvulus oleifolius*, *Convolvulus cneorum*, *Convolvulus lanuginosus*, *Convolvulus gharbensis*, *Convolvulus humilis*.
- Axillary pedicellate flowers with peduncle absent or very short: *Convolvulus vidalii*, *Convolvulus pitardii*, *Convolvulus glaouorum* (somewhat so), *Convolvulus fruticulosus*, *Convolvulus boedeckerianus*, *Convolvulus ocellatus*, *Convolvulus randii*.
- Flowers sessile, solitary or paired: *Convolvulus hamrinensis*, *Convolvulus oxyphyllus*, *Convolvulus socotranus*, *Convolvulus hystrix* (subsp. ruspolii only), *Convolvulus kossmatii*, *Convolvulus semhaensis*, Convolvulus caput-medusae, *Convolvulus sericophyllus* (near sessile), *Convolvulus argillicola*.
- Inflorescence paniculate in form: *Convolvulus floridus*. Several other species could sometimes be interpreted as having a paniculate inflorescence including *Convolvulus aucheri*, *Convolvulus cantabrica*, *Convolvulus pilosellifolius* and *Convolvulus prostratus*.
- Inflorescence racemose in form: *Convolvulus sericophyllus*, *Convolvulus hildebrandtii*, *Convolvulus peninsularis*, *Convolvulus leptocladus*.
- Peduncles paired (at least sometimes): *Convolvulus rufescens*, *Convolvulus thomsonii*.

Sepals. The calyx consists of five separate overlapping sepals, which are commonly somewhat similar in size and shape but often slightly unequal. The two outer sepals are nearly identical; the middle is commonly asymmetric with two halves unequal and the inner pair similar to each other. All sepals may be scarious marginally, but the inner sepals often have wider scarious margins and are less hirsute than the outer pair. The sepal margins are entire or sometimes slightly undulate. In several species, the lower part of the sepal is more or less colourless and contrasts with the distinct green apical portion. In general the size and relative size of the inner and outer sepals, sepal shape, texture and indumentum are all of taxonomic importance. Unusual sepal structures include:

- Outer sepals noticeably shorter than inner sepals: *Convolvulus scammonia*, *Convolvulus pseudoscammonia*, *Convolvulus spinosus*.
- Outer sepals conspicuously larger than inner sepals: *Convolvulus gortschakovii*.
- Sepals conspicuously accrescent: *Convolvulus argillicola*.
- Sepals very lax (not appressed to base of corolla): *Convolvulus leiocalycinus*.
- Sepals spathulate (and reflexed): *Convolvulus durandoi*.
- Sepals rectangular: *Convolvulus rectangularis*, *Convolvulus lanjouwii*.
- Sepals with a conspicuously different coloured apex: *Convolvulus prostratus*, *Convolvulus pilosellifolius*, *Convolvulus cantabrica*, *Convolvulus aucheri*, Convolvulus betonicifolius, *Convolvulus tricolor*, *Convolvulus cataonicus*, *Convolvulus carduchorum*, *Convolvulus germaniciae*, *Convolvulus volubilis*, *Convolvulus massonii*, *Convolvulus oppositifolius*, *Convolvulus lineatus*.
- Sepals mostly > 10 mm long: *Convolvulus lilloi*, *Convolvulus hasslerianus* (South America), *Convolvulus carrii* (North America), *Convolvulus bidrensis*, *Convolvulus thomsonii*, *Convolvulus kilimandschari*, *Convolvulus thunbergii*, *Convolvulus capensis*, *Convolvulus natalensis*, *Convolvulus argillicola*, *Convolvulus bullerianus* (Africa), *Convolvulus massonii*, *Convolvulus lopezsocasii* (Atlantic Islands), *Convolvulus scammonia* (inner sepals), Convolvulus glomeratus var. sachalitarum, *Convolvulus holosericeus*, *Convolvulus sericocephalus*, *Convolvulus fruticosus*, *Convolvulus gortschakovii*, *Convolvulus betonicifolius*, *Convolvulus supinus*, *Convolvulus persicus*, *Convolvulus phrygius*, *Convolvulus kotschyanus*, *Convolvulus commutatus*, *Convolvulus lanatus*, *Convolvulus secundus*, *Convolvulus schimperi*, *Convolvulus pyrrotrichus*, *Convolvulus aitchisonii*, *Convolvulus oxysepalus*, *Convolvulus cephalopodus*, *Convolvulus reticulatus*, *Convolvulus stapfii*, *Convolvulus cephalophorus*, *Convolvulus urosepalus*.
- Sepals all very short, < 5 mm long: *Convolvulus arvensis*, *Convolvulus mairei*, *Convolvulus durandoi*, *Convolvulus fatmensis*, *Convolvulus vidalii*, *Convolvulus coelesyriacus*, *Convolvulus humilis*, *Convolvulus floridus*, *Convolvulus microsepalus*, *Convolvulus crispifolius*, *Convolvulus recurvatus*, *Convolvulus clementii*, *Convolvulus verecundus*, *Convolvulus waitaha*, *Convolvulus assyricus*, *Convolvulus dorycnium*, *Convolvulus sericophyllus*, *Convolvulus sarmentosus*, *Convolvulus grantii*, *Convolvulus hildebrandtii*, *Convolvulus peninsularis*, *Convolvulus leptocladus*, *Convolvulus eremophilus*, *Convolvulus erinaceus*, *Convolvulus chondrilloides*, *Convolvulus gracillimus*, *Convolvulus iranicus*, *Convolvulus verdcourtianus*.

Corolla. The corolla of all *Convolvulus* species is funnel-shaped. The short basal tube is usually more or less included in the calyx while the expanded part is strongly exserted and usually conspicuous. It is undulate to 5–lobed, although it is not completely certain how constant this distinction is. On the exterior, there are 5 darker coloured and usually hirsute bands which terminate at the apex of each corolla lobe. These are referred to as midpetaline bands. They do not extend to the basal, cylindrical portion of the corolla. Corolla colour is sometimes of taxonomic importance but it is difficult to assess from dried specimens and so has been used with caution. Some species apparently always have a white corolla such as *Convolvulus persicus* and *Convolvulus erinaceus* and its allies whereas others such as *Convolvulus chinensis* and *Convolvulus dorycnium* seem always to be pink-flowered but there is uncertainty about how constant this character is in many species.

- Corolla often or always deeply lobed: *Convolvulus rufescens*, *Convolvulus crenatifolius*, *Convolvulus bonariensis*, *Convolvulus montanus*, *Convolvulus bullerianus*, *Convolvulus argillicola*, *Convolvulus multifidus*, *Convolvulus farinosus*, *Convolvulus aschersonii*, *Convolvulus rhyniospermus*, *Convolvulus erinaceus*, *Convolvulus fatmensis*, *Convolvulus siculus*, *Convolvulus simulans*, *Convolvulus volubilis*, *Convolvulus scoparius*.
- Corolla yellow (or cream): *Convolvulus scammonia*, *Convolvulus pseudoscammonia*, *Convolvulus supinus*, *Convolvulus palaestinus*, *Convolvulus natalensis*, *Convolvulus bullerianus*.
- Corolla blue or bluish: *Convolvulus sabatius*, *Convolvulus valentinus* (?), *Convolvulus siculus*, *Convolvulus pentapetaloides*, *Convolvulus humilis*, *Convolvulus gharbensis*, *Convolvulus simulans*, *Convolvulus tricolor*, *Convolvulus meonanthus*, *Convolvulus subspathulatus*, *Convolvulus jefferyi*, *Convolvulus capituliferus*, *Convolvulus canariensis*, *Convolvulus fruticulosus*.
- Midpetaline bands glabrous: *Convolvulus scammonia*, *Convolvulus pseudoscammonia*, *Convolvulus durandoi*, *Convolvulus siculus*, *Convolvulus simulans*, *Convolvulus dregeanus*, *Convolvulus montanus*, *Convolvulus laciniatus* (sometimes), *Convolvulus waitaha*, Convolvulus rhyniospermus, *Convolvulus capituliferus* (almost).

Stamens. Stamens are unequal in length. The main taxonomic character of interest is in the filaments. Sessile or very shortly stipitate glands are present on the lower expanded part of the filaments in all annual species, and trailing and twining species from the Mediterranean and Central Asian regions. They are absent from species with cuneate or attenuate leaf bases and apparently from the twining and trailing species from the southern hemisphere.

Style and stigma. The length of the undivided portion of the style is provided in the descriptions but this may well be more variable than the dimensions given as we have not generally examined the style of many examples of each species. More significant is the relative length of the undivided portion of the style to that of the stigmas. In a number of species, the stigmas are almost as long as the undivided style although they are usually much shorter. The stigmas are weakly exserted from the corolla in a number of species (Convolvulus crenatifolius subsp. montevidensis, *Convolvulus equitans*), possibly because the corolla is somewhat wider than in most other species. These species are also unusual for the persistence of the style on the ripening capsule.

In most species the stigmas are linear-filiform and co-extensive with the style arm. In a small group of Socotran species previously placed in *Seddera* (*Convolvulus kossmatii*, *Convolvulus socotranus*, *Convolvulus semhaensis*) the stigmas are clavate and shorter than the style arm (Luna et al. 2014). Ellipsoid stigmas are found in a number of species in the same clade (*Convolvulus glomeratus*, *Convolvulus hystrix* etc.) and upwardly thickened stigmas occur in various other species, notably *Convolvulus leiocalycinus* and several South African species. Several South American species also have short, rather thick stigmas (*Convolvulus demissus*, *Convolvulus chilensis*). This last species is unusual in exhibiting rather different stigmas on different plants, linear stigmas 3.5 mm long occurring on some plants while oblong stigmas c. 1.5 mm long occur on others. The frequency or significance of this variation is unknown. In most plants both stigmas in each pair are equal but occasional specimens have been observed where they are asymmetric. This does not seem to be species specific.

- Stigmas 3: *Convolvulus maireanus*.
- Stigmas not coequal with style arm: *Convolvulus kossmatii*, *Convolvulus socotranus*, *Convolvulus semhaensis*.
- Stigmas thickened, oblong to ellipsoid in form, much shorter than style: *Convolvulus leiocalycinus*, *Convolvulus persicus*, *Convolvulus hystrix*, *Convolvulus glomeratus*, *Convolvulus virgatus*, *Convolvulus oppositifolius*, *Convolvulus subspathulatus*, *Convolvulus scopulatus*, *Convolvulus capensis*, *Convolvulus namaquensis*, *Convolvulus bidentatus*, *Convolvulus chilensis* (sometimes).
- Stigmas equalling or longer than style: *Convolvulus ulicinus*, *Convolvulus aschersonii*, *Convolvulus chondrilloides*, *Convolvulus oxysepalus*, *Convolvulus koieanus*, *Convolvulus leptocladus*, *Convolvulus peninsularis*, *Convolvulus hildebrandtii*, *Convolvulus trabutianus*, *Convolvulus sarmentosus*, *Convolvulus grantii*, *Convolvulus prostratus*, *Convolvulus pilosellifolius*, *Convolvulus aucheri*, *Convolvulus cantabrica*, *Convolvulus gracillimus*, *Convolvulus scoparius*, *Convolvulus floridus*, *Convolvulus lanuginosus*, *Convolvulus cneorum*. *Convolvulus calvertii*, *Convolvulus ammannii*, *Convolvulus oleifolius*, *Convolvulus holosericeus*, *Convolvulus assyricus*, *Convolvulus libanoticus*, *Convolvulus phrygius*, *Convolvulus cephalopodus*, *Convolvulus jordanensis*, *Convolvulus spicatus*, *Convolvulus secundus*, *Convolvulus lanatus*, *Convolvulus schimperi*, *Convolvulus kotschyanus*, *Convolvulus reticulatus*, *Convolvulus pyrrotrichus*, *Convolvulus fruticulosus*, *Convolvulus sabatius*, *Convolvulus valentinus*, *Convolvulus durandoi*, *Convolvulus pentapetaloides*, *Convolvulus humilis*, *Convolvulus massonii*, *Convolvulus volubilis*, *Convolvulus lopezsocasii* (?). *Convolvulus canariensis*.

Style and ovary indumentum. The indumentum of the ovary, ripening capsule and style is of considerable taxonomic importance. Style indumentum correlates closely with that of the ovary but there are cases where the ovary is hirsute and the style glabrous (Convolvulus hermanniae subsp. erosus). However, we know of no cases where the lower part of the style is hirsute but the ovary is glabrous. Two North American species (*Convolvulus equitans*, *Convolvulus carrii*) are unique in that the upper part of the style, immediately below its division into two arms, is pilose but only in some of the specimens we have seen. In both these cases the ovary is glabrous and the character, although interesting, appears not to be of taxonomic significance.

Ovary indumentum has been used extensively in species delimitation by Sa’ad, Rechinger and others. Indeed (Rechinger 1963) uses the presence or absence of ovary hairs as one of the first dichotomies in the key to species in Flora Iranica. It has been noted as the principal but not the only character to distinguish several pairs of species including Convolvulus lanuginosus/Convolvulus calvertii and Convolvulus oxysepalus/Convolvulus turrillianus. However, others have discounted its importance (O’Donell 1957: 169) or ignored it altogether (Meeuse 1958). Our own studies suggest that it is often species specific but there are many cases where the presence or absence of ovary hairs does not correlate either with other morphological differences or with geographical distribution. This is certainly the case with *Convolvulus ocellatus* from Southern Africa, *Convolvulus hildebrandtii* from Somalia and Socotra and *Convolvulus leiocalycinus* from the Iranian region as well as with a number of species from Madeira and the Canary Islands. More controversially, we have adduced that indumentum differences are not significant in separating several hitherto recognised species, which we have included within *Convolvulus eremophilus*. Where some geographical patterning is obvious, we have accepted existing taxonomic decisions (as in the separation of *Convolvulus spicatus* from *Convolvulus cephalopodus*) or recognised subspecies (as in the South American Convolvulus hermanniae subsp. hermanniae and subsp. erosus), although no additional characters seem to separate these taxa

- Style sometimes hirsute immediately below stigmas: *Convolvulus equitans*, *Convolvulus carrii*.
- Ovary (and usually the capsule) hirsute at least at apex: Convolvulus hermanniae subsp. erosus, *Convolvulus ocellatus* (sometimes), *Convolvulus semhaensis* (sometimes), *Convolvulus hildebrandtii* (sometimes), *Convolvulus mairei*, *Convolvulus leiocalycinus* (sometimes), *Convolvulus galaticus*, *Convolvulus germaniciae*, *Convolvulus cassius*, *Convolvulus betonicifolius*, *Convolvulus palaestinus*, *Convolvulus maireanus*, *Convolvulus massonii* (sometimes), *Convolvulus canariensis*, *Convolvulus lopezsocasii*, *Convolvulus fruticulosus* (sometimes), *Convolvulus* sp. A, *Convolvulus supinus* (occasionally), *Convolvulus erinaceus*, *Convolvulus eremophilius* (usually), *Convolvulus divaricatus*, *Convolvulus tujuntauensis*, *Convolvulus subsericeus*, *Convolvulus hamadae*, *Convolvulus chondrilloides*, *Convolvulus lindbergii* (sometimes), *Convolvulus sarothrocladus*, *Convolvulus koieanus*, *Convolvulus gracillimus*, *Convolvulus ammannii*, *Convolvulus xanthopotamicus*, *Convolvulus grigorjevii*, *Convolvulus krauseanus*, *Convolvulus tragacanthoides*, *Convolvulus spinifer*, *Convolvulus fruticosus*, *Convolvulus gortschakovii*, *Convolvulus spinosus*, *Convolvulus argyracanthus*, *Convolvulus acanthocladus*, *Convolvulus iranicus*, *Convolvulus urosepalus*, *Convolvulus turrillianus*, *Convolvulus cantabrica*, *Convolvulus aucheri*, *Convolvulus schirazianus*, *Convolvulus commutatus*, *Convolvulus elymaiticus*, *Convolvulus calvertii*, *Convolvulus sericocephalus*, *Convolvulus holosericeus*, *Convolvulus boissieri*, *Convolvulus suendermannii*, *Convolvulus lineatus*, *Convolvulus oleifolius*, *Convolvulus argyrothamnos*, *Convolvulus mazicum*, *Convolvulus phrygius*, *Convolvulus libanoticus*, *Convolvulus assyricus*, *Convolvulus cataonicus*, *Convolvulus cneorum*, *Convolvulus caput-medusae*, *Convolvulus scoparius*, *Convolvulus floridus*, *Convolvulus oxyphyllus*, *Convolvulus hamrinensis*, *Convolvulus cephalopodus*, *Convolvulus asyrensis*, *Convolvulus stapfii*, *Convolvulus cephalophorus*, *Convolvulus ulicinus*.

Capsule and seeds. The fruit is a capsule and is ovoid, subglobose or somewhat ellipsoid and acuminate in shape. It has the same indumentum as the ovary. The base of the style is persistent in some species. The dehiscence is loculicidal. The capsule is basically bilocular and 4-seeded with trigonous seeds. However, unilocular capsules and single seeds occur quite frequently by abortion and may sometimes be species specific. The shape of the seed is more or less ellipsoidal if only one seed is present. Seeds may be glabrous or variously hairy. The surface may be smooth, reticulate or tuberculate.

Despite the great variety of fruit characters there are severe practical limitations in their use for taxonomic purposes, particularly in the herbarium. Most specimens are collected in flower and capsules and ripe seeds are often missing. Still more serious is the fact that for a large number of species the capsule and seeds are unknown. Given the small number of fruiting specimens available it is often impossible to be certain whether single seeded or 2–4-seeded capsules are species specific or the result of chance abortion. Our observations of seed ornamentation do not always agree with those of other authors and it is not always easy to be sure whether this is the result of natural variation, wrong identification or observation using different strengths of magnification. Consequently, caution should be exercised in relying on distinctions based solely on seed characters. Some generalisations include:

- Many fastigiate species are 1-seeded (*Convolvulus scopulatus*, *Convolvulus erinaceus*, *Convolvulus dorycnium*, *Convolvulus eremophilus*, *Convolvulus divaricatus*, *Convolvulus pseudocantabrica*) but 1-seeded capsules are also known in *Convolvulus floridus*, *Convolvulus hystrix* and *Convolvulus commutatus*.
- All herbaceous petiolate species have glabrous seeds, the seeds commonly being tuberculate or rugose, sometimes more or less reticulate with raised wavy lines as in several African species *Convolvulus kilimandschari*, *Convolvulus farinosus* and *Convolvulus sagittatus*.
- Capsules and seeds are usually found in abundance on annual species.
- Capsules and seeds are usually present on herbaceous petiolate species.

The following character lists may prove useful although they are not necessarily exhaustive:

- Peduncles reflexed in fruit (most common in annual and Australian species): *Convolvulus mairei*, *Convolvulus fatmensis*, *Convolvulus palaestinus*, *Convolvulus coelesyriacus*, *Convolvulus germaniciae* (?), Convolvulus pitardii, *Convolvulus vidalii*, *Convolvulus glaouorum*, *Convolvulus siculus*, *Convolvulus pentapetaloides*, *Convolvulus tricolor*, *Convolvulus simulans*, *Convolvulus microsepalus*, *Convolvulus recurvatus*, *Convolvulus graminetinus*, *Convolvulus crispifolius*, *Convolvulus eyreanus*, *Convolvulus angustissimus*, *Convolvulus waitaha*, *Convolvulus stenocladus*.
- Seeds hirsute: *Convolvulus acanthocladus*, *Convolvulus fruticosus*, *Convolvulus dorycnium*, *Convolvulus chondrilloides*, *Convolvulus eremophilus*, *Convolvulus erinaceus*, *Convolvulus divaricatus*, *Convolvulus tujuntauensis*, *Convolvulus pseudocantabrica*, *Convolvulus floridus*, *Convolvulus calvertii*, *Convolvulus commutatus*, *Convolvulus cantabrica*, *Convolvulus cneorum*, *Convolvulus lanuginosus*, *Convolvulus lineatus*, *Convolvulus oleifolius*, *Convolvulus holosericeus*, *Convolvulus assyricus*, *Convolvulus libanoticus*, *Convolvulus asyrensis*, *Convolvulus cephalopodus*, *Convolvulus reticulatus*, *Convolvulus prostratus*, *Convolvulus pilosellifolius*, *Convolvulus rottlerianus*, *Convolvulus lanjouwii*, *Convolvulus sericophyllus*, *Convolvulus sarmentosus*, *Convolvulus hildebrandtii* (sometimes), *Convolvulus verdcourtianus*, *Convolvulus xanthopotamicus*, *Convolvulus ammannii*.
- Seeds smooth, glabrous: *Convolvulus leiocalycinus*, *Convolvulus hystrix*, *Convolvulus lanatus*, *Convolvulus chondrilloides*.

## Dichotomous keys

Keys for the identification of *Convolvulus* species are provided on a regional basis. This ensures that keys are relatively short and the user has only a small number of species to consider if facing difficulties in deciding to which species a particular specimen belongs. Subspecies are only keyed out where more than one occurs in a particular region. Efforts have been made to ensure that similar or confusable species are contrasted in couplets, rather than being placed far apart by the use of an arbitrary character. The following 14 regional keys are provided:

1. South America
2. North America
3. Australia
4. New Zealand
5. Southern Africa (Botswana, Lesotho, Namibia, South Africa, Swaziland)
6. Tropical Africa (Sahel south to Angola, Mozambique and Zimbabwe, including Socotra and Madagascar)
7. North Africa (Morocco, Algeria, Tunisia, Libya, Egypt, Mauritania and Niger)
8. Atlantic Islands (Azores, Canaries, Cape Verde, Madeira)
9. Europe (Flora Europaea area)
10. Levant (Turkey, Cyprus, Syria, Lebanon, Palestine/Israel, Jordan)
11. Arabian Peninsular (including Socotra and Kuwait)
12. Indo-Iranian Region (Afghanistan, Bhutan, India, Iran, Iraq, Nepal, Pakistan)
13. Former Soviet Union
14. East Asia (Burma/Myanmar, China, Japan, Korea, Mongolia, Eastern Siberia).

### Regional keys to *Convolvulus* species

**1. Key to species in South America**

**Table:** 

1	Erect cerrado perennial with woody xylopodium; leaves sessile or subsessile	**55. *Convolvulus hasslerianus***
–	Twining, trailing or prostrate herbs of varied habitats; leaves distinctly petiolate	**2**
2	Outer sepals 3–6 mm long	**3**
–	Outer sepals 6–14 mm long	**4**
3	corolla <1 cm long; outer sepals 5–6 mm long	**47. *Convolvulus schulzei***
–	corolla 2–3 cm long; outer sepals 3–4.5 mm long	**4. *Convolvulus arvensis***
4	Leaves deeply palmatisect or pinnatisect, the segments usually very fine	**48. *Convolvulus laciniatus***
–	Leaves entire, weakly lobed, dentate or crenate, the basal auricles entire or bifid, never palmatisect or pinnatisect	**5**
5	Outer sepals 10–14 mm long; corolla 2.5–4 cm long	**53. *Convolvulus lilloi***
–	Outer sepals 7–10 mm long; corolla 1–3 cm long	**6**
6	Prostrate plants; leaves < 3 cm long, usually much less; flowers solitary (rarely paired)	**7**
–	Prostrate or twining plants; leaves mostly >3 cm long; flowers in 1–many-flowered cymes	**9**
7	Leaves suborbicular, glabrous or nearly so; corolla with glabrous midpetaline bands	**50. *Convolvulus montanus***
–	Leaves ovate-deltoid, pubescent; corolla with hirsute midpetaline bands	**8**
8	Leaves entire or very shallowly lobed (Chile)	**46. *Convolvulus demissus***
–	Leaves incised-dentate (Peru)	**51. *Convolvulus incisodentatus***
9	Corolla (1.5-)2–3 cm long, pink; leaf auricles usually bifid	**44. *Convolvulus chilensis***
–	Corolla 1–2.5 cm long but, if more than 1.5 cm corolla cream; leaf auricles entire, rarely bifid and, if so, corolla < 1.5 cm long	**10**
10	Leaves linear oblong, glabrous, petiole < 6 mm long, flowers always solitary	**54. *Convolvulus ensifolius***
–	Leaves ovate, deltoid or strap-shaped, pubescent or hirsute, petiole > 5 mm long, flowers 1–many	**11**
11	Ovary and capsule apically pilose; plant commonly white-pilose	**49. Convolvulus hermanniae subsp. erosus**
–	Ovary and capsule glabrous; plant variously hairy to subglabrous	**12**
12	Corolla 1.6–2.5 cm long, cream; flowering stems slender, c. 1–1.5 mm thick	**52. Convolvulus crenatifolius subsp. montevidensis**
–	Corolla 1–1.8 cm long, white or pink; flowering stems relatively stout, 2–3 mm thick	**13**
13	Leaves usually 4–5 times as long as broad, puberulent, the auricles sometimes bifid	**45. *Convolvulus bonariensis***
–	Leaves ovate-deltoid, 2–3 times as long as broad, usually hirsute, the hairs more or less spreading, the auricles never bifid	**14**
14	Inflorescence of (1-)3–7-flowered cymes; peduncles 1.5–12 cm long; corolla pinkish	**52. Convolvulus crenatifolius subsp. crenatifolius**
–	Flowers solitary or paired; peduncles 1–3(-6) cm long; corolla white	**49. *Convolvulus hermanniae***

**2. Key to species in North America**

**Table:** 

1	Annual. Leaves narrowly oblong-oblanceolate with a long petiole-like base; corolla 5–6 mm long	**92. *Convolvulus simulans***
–	Perennial. Leaves various but never narrowly oblong-oblanceolate with a long petiole-like base; corolla more than 10 mm long	**2**
2	Corolla pink, 1.8–4.5 cm long, leaves strongly dimorphic, the upper leaves deeply incised (naturalised in California)	**22. *Convolvulus althaeoides***
–	Corolla < 2.5 cm long but, if longer, white; leaves not dimorphic; upper stem leaves not deeply incised	**3**
3	Outer sepals 3–4.5 cm long; corolla 3–4 times longer than calyx, usually pink	**4. *Convolvulus arvensis***
–	Outer sepals 6–11 mm long; corolla mostly about twice as long as calyx but, if much more, white	**4**
4	Sepals mostly 9–11 mm long; corolla > 2 cm long	**5**
–	Sepals < 8 mm long; corolla < 1.8 cm long	**6**
5	Leaves and stem white-tomentellous; leaves abaxially with prominent raised veins	**43. *Convolvulus carrii***
–	Leaves and stem not white-tomentellous; leaves lacking prominent raised veins	**42. Convolvulus equitans var. lindheimeri**
6	Leaves ovate-deltoid, neither lobed nor deeply incised, auricles simple; outer sepals narrowed to base; peduncles with 1–5 flowers	**7**
–	Leaves deeply lobed or incised and/or auricles deeply bifid; outer sepals often truncate to auriculate at base; peduncles with 1–2 flowers, rarely more	**42. *Convolvulus equitans***
7	Corolla 1.3–1.8 cm long; leaf margin incised-dentate; sepals reddish-brown	**52. *Convolvulus crenatifolius***
–	Corolla 1–1.5 cm long; leaf margin usually entire or undulate; sepals not reddish-brown	**36. *Convolvulus farinosus***

**3. Key to species in Australia**

**Table:** 

1	Corolla more than 1.5 cm long	**2**
–	Corolla less than 1.5 cm long	**3**
2	Leaves usually strongly dimorphic and/or with narrowly linear segments; sepals > 4 mm long	**66. *Convolvulus angustissimus***
–	Leaves not or only weakly dimorphic, the segments never linear; sepals < 4.5 cm long	**4. *Convolvulus arvensis***
3	Sepals less than 3 mm long	**56. *Convolvulus microsepalus***
–	Sepals more than 4 mm long, often much more	**4**
4	Fruiting peduncles straight or sinuate, never recurved	**5**
–	Fruiting pedicels recurved	**8**
5	Leaves with central lobe entire or undulate; basal auricles distinct, not intergrading with sinuate-margined central lobe; corolla 1–1.5 cm long	**58. *Convolvulus remotus***
–	Leaves with central lobe dentate, sinuate or dissected, the basal auricles variously lobed or dentate	**6**
6	Flowers usually solitary, peduncles solitary; seeds winged; corolla 7–9 mm long	**61. *Convolvulus clementii***
–	Flowers usually in small axillary cymes, peduncles sometimes paired in leaf axils; seeds unwinged; corolla 9–15 mm long	**7**
7	Corolla < 10 mm long; stems stout, coarsely hairy	**62. *Convolvulus tedmoorei***
–	Corolla > 12 mm long; stems relatively slender, softly pubescent or glabrous; corolla 1.2–1.5 mm	**65. *Convolvulus erubescens***
8	Leaves sericeous-tomentose with appressed hairs, basal lobes usually not prominent	**9**
–	Leaves glabrous to roughly pubescent with spreading hairs; basal lobes usually prominent	**10**
9	Peduncles very short, < 12 mm long	**59. *Convolvulus crispifolius***
–	Peduncles > 12 mm long	**60. *Convolvulus eyreanus***
10	Sepals glabrous to sparsely hairy	**57. *Convolvulus graminetinus***
–	Sepals pubescent, often densely so	**11**
11	Seeds winged; corolla 7–9 mm long	**63. *Convolvulus recurvatus***
–	Seeds unwinged; corolla 9–12 mm long	**64. *Convolvulus wimmerensis***

**4. Key to species in New Zealand**

**Table:** 

1	Leaves dimorphic on the same plant, some being ovate-deltoid in form, others being deeply laciniate with filiform lobes	**2**
–	Leaves more or less uniform in shape, generally ovate to suborbicular in form	**3**
2	Sepals 6–8 mm long; peduncles not reflexed in fruit	**67. *Convolvulus fractosaxosus***
–	Sepals 4–6 mm long; peduncles reflexed in fruit	**57. *Convolvulus graminetinus***
3	Corolla > 2 cm long	**4. *Convolvulus arvensis***
–	Corolla < 2 cm long	**4**
4	Corolla relatively small, < 14 mm long; plant subglabrous with only a few hairs, these especially on petioles	**69. *Convolvulus waitaha***
–	Corolla 15–19 mm long; plant pubescent	**68. *Convolvulus verecundus***

**5. Key to species in Southern Africa**

**Table:** 

1	Flowers sessile; corolla scarcely exceeding calyx; calyx strongly accrescent in fruit	**31. *Convolvulus argillicola***
–	Flowers pedicellate and/or pedunculate; corolla much exceeding calyx; calyx not markedly accrescent in fruit	**2**
2	Calyx < 6 mm long	**3**
–	Calyx 6–15 mm long	**6**
3	Corolla 2–4 times longer than the calyx; leaves usually < 2 cm long	**4**
–	Corolla only slightly exceeding calyx; leaves usually > 2 cm long	**35. *Convolvulus aschersonii***
4	Leaves ovate-deltoid, sagittate, never lobed	**4. *Convolvulus arvensis***
–	Leaves usually lobed or segmented but, if entire, linear-oblong	**5**
5	Plant completely glabrous, even on the exterior of the corolla; flowers pedunculate	**28. *Convolvulus dregeanus***
–	Plant pubescent; flowers pedicellate but peduncles absent or very short	**29. *Convolvulus boedeckerianus***
6	Peduncles short or absent, flowers solitary	**7**
–	Peduncles always present, short or long, 1–5-flowered	**9**
7	Leaves with 5–9 linear or filiform lobes; corolla lobes obtuse or rounded	**30. *Convolvulus multifidus***
–	Leaves entire or 3–5-fid, lobes not markedly narrow; corolla lobes acute	**8**
8	Leaves always entire, silvery-sericeous, margins not inrolled; sepals acute	**33. *Convolvulus randii***
–	Leaves usually palmately-lobed, rarely entire, brownish-villous, margins inrolled; sepals obtuse	**32. *Convolvulus ocellatus***
9	Corolla < 15 mm long	**10**
–	Corolla > 15 mm long	**13**
10	Leaves broadly to narrowly triangular to ovate, the base truncate, sagittate or hastate but auricles not lobed	**11**
–	Leaves palmately-lobed with the central lobe much longer than the auricles, which are usually bilobed	**12**
11	Flowers solitary; petioles short; plant decumbent to erect, rarely twining; corolla indistinctly lobed	**37. *Convolvulus sagittatus***
–	Flowers in cymes of 1–6; petioles to 6 cm, plant usually twining; corolla lobed	**36. *Convolvulus farinosus***
12	Central lobe of leaf broad, coarsely serrate to pinnatisect; plant roughly hairy	**34. *Convolvulus austroafricanus***
–	Central lobe of leaf linear-oblong (rarely broad), entire; plant glabrous to finely pubescent	**35. *Convolvulus aschersonii***
13	Leaves linear with hastate base	**14**
–	Leaves various, usually pinnately to palmately lobed or triangular-ovate	**15**
14	Sepals obtuse; corolla shallowly lobed	**25. *Convolvulus bidentatus***
–	Sepals acute; corolla deeply lobed	**41. *Convolvulus bullerianus***
15	Leaves unlobed, entire or crenate	**16**
–	Leaves palmately or pinnately lobed	**19**
16	Corolla < 2 (-2.5)cm long; calyx < 10 mm long	**17**
–	Corolla 2–3. 5 cm long; calyx > 10 mm long	**40. *Convolvulus natalensis***
17	Sepals abruptly narrowed into an apiculate point	**39. *Convolvulus galpinii***
–	Sepals rounded to acute but not abruptly narrowed	**18**
18	Central lobe of leaf sinuate; flowers usually 2 or more	**26. *Convolvulus namaquensis***
–	Leaf entire; flowers usually solitary	**37. *Convolvulus sagittatus***
19	Leaves pinnately nerved with sinuous-margined central lobe	**27. *Convolvulus thunbergii***
–	Leaves palmately lobed or palmatifid	**24. *Convolvulus capensis***

**6. Key to species in Tropical Africa**

**Table:** 

1	Undershrub with woody spinescent branches	**2**
–	Plant unarmed although stems sometimes stiff and woody	**6**
2	Leaves abruptly narrowed at base; flowers in subsessile clusters	**3**
–	Leaves cuneate at base, flowers solitary or paired, not pilose	**4**
3	Stems and leaves hirsute; flower clusters usually 2–6-flowered; corolla > 12 mm long; bracteoles 3–5 mm wide	**104. Convolvulus hystrix subsp. hystrix**
–	Stems and leaves glabrous to thinly pubescent; flower clusters 1(-2)-flowered; corolla 8–10 mm long; bracteoles 1–2 mm wide	**104. Convolvulus hystrix subsp. ruspolii**
4	Flowers shortly pedicellate, solitary or paired; stigmas linear (Somalia)	**108. *Convolvulus verdcourtianus***
–	Flowers sessile, solitary; stigmas clavate (Socotra)	**5**
5	Ovary glabrous; sepals broadly obovate-elliptic	**106. *Convolvulus kossmatii***
–	Ovary hirsute; sepals oblong-lanceolate	**107. *Convolvulus semhaensis***
6	Annual herb; plant entirely herbaceous	**7**
–	Perennial herb or undershrub	**10**
7	Lamina ovate, abruptly narrowed into a distinct petiole	**8**
–	Lamina oblong, lanceolate or oblong (rarely ovate), sessile or narrowed at base with petiole not clearly demarcated	**9**
8	Leaves crenate; sepals obtuse; corolla pink	**7. *Convolvulus fatmensis***
–	Leaves entire; sepals acute; corolla blue	**87. *Convolvulus siculus***
9	Flowers 3–6 in sessile axillary clusters	**93. *Convolvulus rhyniospermus***
–	Flowers up to 3 in pedunculate cymes	**110. *Convolvulus rottlerianus***
10	Corolla large, 2.5–4 cm in length; mountain liana	**23. *Convolvulus kilimandschari***
–	Corolla < 2 cm in length; low herb if occurring on mountains	**11**
11	Flowers arranged in few- to many-flowered heads, these pedunculate or sessile, the flower bases often concealed by bracts	**12**
–	Flowers solitary or in lax cymes with pedicels clearly developed, flower bases usually easily visible	**21**
12	Leaves linear, < 5 mm long. Undershrub	**103. *Convolvulus scopulatus***
–	Leaves oblong or lanceolate, > 10 cm long. Herbs or undershrubs	**13**
13	Bracts and calyx glabrous	**97. *Convolvulus bidrensis***
–	Bracts and calyx variously hirsute	**14**
14	Flower heads subsessile; peduncles < 5 mm long	**15**
–	Flower heads distinctly pedunculate with peduncles > 10 mm long	**18**
15	Corolla pale pink or white; woody below	**16**
–	Corolla blue; herbaceous	**17**
16	Leaves abruptly narrowed into a short petiole up to 6 mm long; sepals uniform in colour	**104. Convolvulus hystrix subsp. inermis**
–	Leaves gradually narrowed to base; sepals bicoloured, pale basally, green at the apex	**111. *Convolvulus prostratus***
17	Leaves obovate, pubescent, up to 10 mm wide; sepals 5–7 mm long	**94. *Convolvulus capituliferus***
–	Leaves oblong-oblanceolate, sericeous, < 6mm wide; sepals 8–9 mm long	**98. *Convolvulus vollesenii***
18	Leaves linear, cuneate at base; bracts linear, appressed to flower head	**96. *Convolvulus stenocladus***
–	Leaves suborbicular, ovate, lanceolate or oblong; bracts neither linear nor appressed to flower head	**19**
19	Leaves suborbicular; plant densely covered in brownish sericeous hairs	**99. *Convolvulus subspathulatus***
–	Leaves lanceolate, ovate or oblong; plant not sericeous	**20**
20	Leaves usually basally cordate; bracts and sepals villous with brownish hairs; flowers usually whitish	**101. *Convolvulus glomeratus***
–	Leaves basally truncate to subhastate; bracts and sepals pubescent; corolla blue	**95. *Convolvulus jefferyi***
21	Leaves sessile, the base of the lamina attenuate at the base	**22**
–	Leaves petiolate, lamina hastate or sagittate, well demarcated from the petiole	**26**
22	Flowers solitary, sessile; stigmas clavate, shorter than the style arm	**105. *Convolvulus socotranus***
–	Flowers grouped into cymes or, if solitary, pedunculate; stigmas linear	**23**
23	Inflorescence racemose in form with very shortly pedunculate cymes	**114. *Convolvulus sericophyllus***
–	Inflorescence paniculate or cymose; flowers with long peduncles	**24**
24	Herbaceous plant; leaves with sinuate or undulate margins; basal rosette present	**115. *Convolvulus grantii***
–	Herbaceous plant becoming woody with age; leaves entire; basal rosette absent	**25**
25	Basal leaves villous, ephemeral; bracts linear	**117. *Convolvulus hildebrandtii***
–	Basal leaves sericeous, persistent; bracts oblong to oblanceolate	**116. *Convolvulus sarmentosus***
26	Corolla 2–5 times as long as calyx; sepals <4.5 mm long	**4. *Convolvulus arvensis***
–	Corolla up to twice as long as calyx; sepals usually > 5 mm long	**27**
27	Leaves crenate; fruiting peduncle reflexed	**7. *Convolvulus fatmensis***
–	Leaves not crenate; fruiting pedicels not reflexed	**28**
28	Erect or twining plant; leaves sericeous with prominent veining on abaxial surface (Zimbabwe)	**33. *Convolvulus randii***
–	Trailing or twining herb, veining on abaxial surface of leaf not prominent	**29**
29	Flowers in 1–7-flowered cymes; peduncles 1.5–12 cm long; corolla < 1.2 cm long	**30**
–	Flowers usually solitary; peduncles 1–3(-6) cm long; corolla > 1.2 cm long	**32**
30	Central lobe of leaves coarsely dentate; stems and leaves roughly hirsute	**34. *Convolvulus austroafricanus***
–	Central lobe of leaf entire to undulate; stems and leaves farinose to softly pubescent	**31**
31	Leaves ovate to triangular; auricles not bifurcate	**36. *Convolvulus farinosus***
–	Leaves oblong or strap-shaped; auricles commonly bifurcate	**35. *Convolvulus aschersonii***
32	Plant densely pubescent to subtomentose; leaf margins shallowly lobed; outer sepals 9–11 mm long	**38. *Convolvulus thomsonii***
–	Plant thinly to densely pubescent: leaf margin usually entire; sepals 6–8 mm long	**37. *Convolvulus sagittatus***

**7. Key to species in North Africa**

**Table:** 

1	Annual herbs; plants slender, herbaceous, never rhizomatous or woody at the base	**2**
–	Perennial herbs or undershrubs, usually robust, the base woody or, if herbaceous, rootstock rhizomatous	**11**
2	Flowers densely clustered, peduncles and pedicels, absent or, if present, very short	**3**
–	Flowers solitary or in lax cymes	**5**
3	Flower 3–6 in axillary clusters; corolla pale pink (Sahara)	**93. *Convolvulus rhyniospermus***
–	Flower clusters terminal (formed from the uppermost leaf axils); corolla blue	**4**
4	Corolla c. 1 cm long; ovary and capsule hirsute; some flowers usually present in uppermost leaf axils (Mediterranean)	**91. *Convolvulus humilis***
–	Corolla 1.5–2.5 cm long; ovary and capsule glabrous; flowers all terminal (Morocco)	**86. *Convolvulus gharbensis***
5	Leaves petiolate; leaf blade abruptly narrowed to a truncate or cordate base	**6**
–	Leaves clearly sessile or leaf blade gradually narrowed to base	**8**
6	Flowers blue; leaves entire, fruiting peduncle not strongly recurved	**7**
–	Flowers pinkish, leaves crenate; peduncle strongly recurved in fruit	**7. *Convolvulus fatmensis***
7	Pedicel absent, bracteole adjacent to calyx	**87. Convolvulus siculus subsp. siculus**
–	Pedicel present, bracteole distant from calyx	**87. Convolvulus siculus subsp. elongatus**
8	Corolla 7–10 mm long, entirely blue	**88. *Convolvulus pentapetaloides***
–	Corolla 14–40 mm long, usually blue, white and yellow banded	**9**
9	Capsule pubescent; sepals with distinct, different coloured lower and upper portions, pubescent (*Convolvulus tricolor*)	**10**
–	Capsule glabrous; sepals without distinct upper and lower areas, glabrous to pubescent	**89. *Convolvulus meonanthus***
10	Upper portion of sepals acute to acuminate, longer than basal portion	**90. Convolvulus tricolor subsp. cupanianus**
–	Upper portion of sepals obtuse to acute, shorter than or equalling the basal portion	**90. Convolvulus tricolor subsp. tricolor**
11	Plant with spinescent branches, at least below	**12**
–	Plant unarmed	**14**
12	Plant subglabrous to densely sericeous (Morocco and Algeria); flowers solitary or in ebracteate clusters	**109. *Convolvulus trabutianus***
–	Plant densely pilose to tomentose (Egypt); flowers in bracteate heads	**13**
13	Leaves < 15 mm long, abruptly narrowed at base; all branches spinescent	**104. *Convolvulus hystrix***
–	Leaves 1–3 cm long, tapered at base; only the old basal, often leafless branches spinescent	**176. *Convolvulus lanatus***
14	Leaves attenuate at base and lacking a distinct petiole; plants never twining	**15**
–	Leaves hastate, sagittate or (less commonly oblong), abruptly narrowed into a distinct petiole; plants twining or not	**24**
15	Mature stems woody and divaricately branched	**167. *Convolvulus dorycnium***
–	Mature stems not woody except below, not divaricately branched	**16**
16	Stems and leaves with spreading hairs	**17**
–	Stems and leaves appressed hairy and more or less sericeous with silvery hairs	**21**
17	Sepals with a pale lower portion and green apex; flowers separate or in few-flowered clusters; leaves mostly pilose or pubescent, sometimes subtomentose	**18**
–	Sepals uniform in colour; flowers in dense heads; leaves densely tomentose (Sinai)	**20**
18	Ovary and capsule hirsute; corolla c. 2 cm long	**146. *Convolvulus cantabrica***
–	Ovary and capsule glabrous; corolla <1.5 cm long	**19**
19	Sepals oblong–oblanceolate, acute; inflorescence lax with some flowers separate	**112. *Convolvulus pilosellifolius***
–	Sepals lanceolate or ovate, acuminate; flowers clustered into heads	**111. *Convolvulus prostratus***
20	Leaf margin undulate; plants apparently prostrate	**180. *Convolvulus schimperi***
–	Leaf margin entire; plants ascending to erect	**178. *Convolvulus spicatus***
21	Cushion plant; flowers solitary or paired, very shortly peduncled	**22**
–	Plants not cushion–forming; flowering stems mostly > 5 cm long, flowers in lax terminal groups	**23**
22	Upper surface of leaves glabrous (Morocco)	**159. *Convolvulus mazicum***
–	Upper surface of leaves at least thinly pubescent (widespread)	**156. *Convolvulus lineatus***
23	Plant usually < 25 cm high; extreme base of leaf widened and scarious; sepals sericeous and spreading pilose	**156. *Convolvulus lineatus***
–	Plant usually 20–50 cm high; extreme base of leaf not widened and scarious; sepals with spreading hairs only	**157. *Convolvulus oleifolius***
24	Leaves almost completely entire, occasionally lobed at base	**25**
–	Leaves undulate, dentate, sinuate–lobed or incised	**31**
25	Inner sepals longer and more prominent than outer sepals; plant completely glabrous	**26**
–	Inner sepals equalling or shorter than the outer sepals; plant hirsute or glabrous	**27**
26	Corolla yellow, 3–4 cm long; outer sepals oblong–obovate without recurved apex (East Mediterranean)	**1. *Convolvulus scammonia***
–	Corolla pink < 2.3 cm long, outer sepals spathulate with reflexed apex (Algeria)	**3. *Convolvulus durandoi***
27	Stem base herbaceous; plant usually glabrous to adpressed pubescent	**28**
–	Stem base woody, plant pubescent, often densely so	**29**
28	Sepals < 5 mm long	**4. *Convolvulus arvensis***
–	Sepals > 6 mm long	**37. *Convolvulus sagittatus***
29	Corolla yellow or yellowish; stigma much shorter than style; petioles all very short, more or less 1 mm	**85. *Convolvulus supinus***
–	Corolla blue or white; stigma and style more or less equal or stigma only slightly shorter; petioles > 2 mm	**30**
30	Leaves more than twice as long as broad, usually acute; bracteoles 0.5 mm wide	**83. *Convolvulus valentinus***
–	Leaves less than twice as long as broad, rounded; bracteoles 1–3.5 mm wide	**84. Convolvulus sabatius subsp. mauritanicus**
31	Flowers in compact pilose axillary heads; stigma clavate	**101. *Convolvulus glomeratus***
–	Flowers solitary or in lax cymes or, if clustered, at apex of stem; stigma linear	**32**
32	Sepals < 5 mm long; peduncle deflexed in fruit; slender trailing herb	**7. *Convolvulus fatmensis***
–	Sepals > 5 mm long; peduncles not deflexed in fruit; plant moderately robust	**33**
33	Flowers clustered at the apex of a peduncle–like stem; stigmas commonly three	**73. *Convolvulus maireanus***
–	Flowers not clustered, solitary to several; stigmas 2	**34**
34	Perennial herbs with herbaceous base; flowers 1–several; peduncle not suppressed	**35**
–	Woody based plants from Morroco; flowers solitary, peduncle commonly short	**39**
35	Leaves undulate to sinuate, not dimorphic	**36**
–	Leaves (or some of them) deeply incised, commonly dimorphic with basal leaves differing markedly from upper stem leaves	**37**
36	Flowers solitary; leaves truncate (NW Africa)	**71. *Convolvulus dryadum***
–	Flowers up to 5; leaves cordate (East Mediterranean)	**14. *Convolvulus stachydifolius***
37	Corolla yellow	**15. *Convolvulus palaestinus***
–	Corolla pink or white	**38**
38	Leaves sericeous, the segments of the upper leaves linear	**22. Convolvulus althaeoides subsp. tenuissimus**
–	Leaves pubescent to pilose, the segments of the upper lanceolate to oblong–elliptic	**22. Convolvulus althaeoides subsp. althaeoides**
39	Corolla purple, the centre white usually with 5 purple spots; peduncles absent; sepals < 5 mm long	**21. *Convolvulus vidalii***
–	Corolla pink or white, lacking a purple-spotted centre; peduncles almost always present; sepals > 4.5 mm long	**40**
40	Calyx lanceolate in outline; sepals lanceolate to ovate	**19. *Convolvulus pitardii***
–	Calyx oblong in outline; sepals obovate, obtuse to truncate	**20. *Convolvulus glaouorum***

**8. Key to species in the Atlantic Islands**

**Table:** 

1	Leaves sessile; shrubs or woody-based herbs	**2**
–	Leaves abruptly narrowed into a distinct petiole; herbs or shrubs	**5**
2	Ovary glabrous; leaves not sericeous; flowers of sessile or pedunculate clasters; plant herbaceous with a woody base (Cape Verde)	**111. *Convolvulus prostratus***
–	Ovary usually hirsute; leaves sericeous; flowers varied but not in sessile or pedunculate clusters; undershrubs or shrubs (Canaries)	**3**
3	Prostrate hummock-forming undershrub with spinescent branches	**169. *Convolvulus caput-medusae***
–	Erect shrubs or herbs, the branches not spinescent	**4**
4	Leaves oblong, > 0.5 cm wide; inflorescence terminal, paniculate, many-flowered	**171. *Convolvulus floridus***
–	Leaves filiform to linear < 0.5 cm wide; inflorescence unbranched or sparingly branched, axillary and terminal, few-flowered (< 10)	**170. *Convolvulus scoparius***
5	Annual herbs, neither twining nor trailing; corolla blue	**6**
–	Perennial herbs, lianas or undershrubs, twining or trailing the basal parts often woody	**8**
6	Leaves sessile, tapered at base; corolla 3-coloured	**90. *Convolvulus tricolor***
–	Leaves abruptly narrowed into a distinct petiole; corolla entirely blue (*Convolvulus siculus*)	**7**
7	Pedicels absent, bracteole appressed to base of calyx, filiform to more or less lanceolate	**87. Convolvulus siculus subsp. siculus**
–	Pedicels borne at least 5 mm below calyx, always filiform	**87. Convolvulus siculus subsp. elongatus**
8	Calyx < 5 mm long, plant entirely herbaceous	**4. *Convolvulus arvensis***
–	Calyx > 5 mm long, base of plant woody, rarely entirely herbaceous	**9**
9	Corolla 10–15 mm long; plant entirely herbaceous (Azores)	**36. *Convolvulus farinosus***
–	Corolla > 15 mm long; plant woody at base (Madeira, Canaries)	**10**
10	Leaves dimorphic, upper leaves deeply segmented	**22. *Convolvulus althaeoides***
–	All leaves similar, upper leaves not deeply segmented	**11**
11	Leaves strongly hirsute	**12**
–	Leaves glabrous or nearly so	**14**
12	Leaves oblong-ovate, densely villous beneath	**77. *Convolvulus canariensis***
–	Leaves elliptic, lanceolate, ovate or oblong, finely pubescent to tomentellous beneath	**13**
13	Leaves ovate, outer sepals 10–13 mm long	**80. *Convolvulus* sp. A**
–	Leaves linear-lanceolate to oblong, sepals 6–9 mm long	**81. Convolvulus fruticulosus subsp. fruticulosus**
14	Cymes long-pedunculate; leaves large 4–11 cm long (Madeira)	**75. *Convolvulus massonii***
–	Cymes borne on peduncles < 2 cm long; leaves < 6 cm long	**15**
15	Leaves < 4 × 1.5 cm wide; cymes 1–2-flowered (Gran Canaria)	**81. Convolvulus fruticulosus subsp. glandulosus**
–	Leaves mostly > 4 × 1.5 cm; cymes 1–6-flowered	**16**
16	Sepals 9–10 mm long; corolla white with pink midpetaline bands (Lanzarote)	**79. *Convolvulus lopezsocasii***
–	Sepals 5 mm long; corolla bluish (Tenerife, La Gomera)	**78. *Convolvulus volubilis***

**9. Key to species in Europe**

**Table:** 

1	Leaves abruptly narrowed at the base into a distinct petiole	**2**
–	Leaves gradually narrowed at the base, lacking a distinct petiole	**15**
2	Leaves cuneate or truncate at the base	**3**
–	Leaves sagittate or hastate at the base	**8**
3	Robust plant, densely lanate; sepals obtuse	**72. *Convolvulus persicus***
–	Slender plant, pubescent; sepals acute to acuminate	**4**
4	Annual herb, corolla 7–12 mm long (*Convolvulus siculus*)	**5**
–	Perennial herb, corolla 1.5–2 cm long	**6**
5	Pedicels absent, bracteole appressed to base of calyx, filiform to more or less lanceolate	**87. Convolvulus siculus subsp. siculus**
–	Pedicels borne at least 5 mm below calyx, always filiform	**87. Convolvulus siculus subsp. elongatus**
6	Pedicels 0–3 mm long; leaves ovate to suborbicular	**83. *Convolvulus valentinus***
–	Pedicels 3–12 mm; leaves lanceolate to oblong, often falcate	**7**
7	Calyx with mostly short appressed hairs	**84. Convolvulus sabatius subsp. sabatius**
–	Calyx with spreading hairs only	**84. Convolvulus sabatius subsp. mauritanicus**
8	Leaves strongly dimorphic, the upper leaves deeply divided (*Convolvulus althaeoides*)	**9**
–	Leaves not strongly dimorphic; the upper leaves not deeply divided	**10**
9	Leaves sericeous beneath; leaf segments narrow, linear to oblong	**22. Convolvulus althaeoides subsp. tenuissimus**
–	Leaves with spreading, often slightly asperous hairs; leaf segments mostly broad	**22. Convolvulus althaeoides subsp. althaeoides**
10	Sepals < 5 mm long	**11**
–	Sepals > 5 mm long	**12**
11	Flowers very small; capsule pubescent, borne on recurved peduncles	**6. *Convolvulus mairei***
–	Flowers usually 15–25 mm long; capsule glabrous, not recurved	**4. *Convolvulus arvensis***
12	Corolla < 17 mm long. Portugal	**13**
–	Corolla 25–45 mm long. Eastern Europe	**14**
13	Liana; leaves oblong and elliptic, retuse, glabrescent; corolla 15–17 mm long	**82. *Convolvulus fernandesii***
–	Twining or decumbent perennial herb with herbaceous stems; leaves usually triangular, acute, farinose or pubescent; corolla 10–15 mm long	**36. *Convolvulus farinosus***
14	Plant glabrous; flowers yellow; sepals emarginate and apiculate	**1. *Convolvulus scammonia***
–	Plant pubescent; flowers usually pink; sepals acute or acuminate	**11. *Convolvulus betonicifolius***
15	Annual herbs; plants entirely herbaceous	**16**
–	Perennial plants, at least the basal portions and rootstock woody	**20**
16	Flowers sessile or nearly so, peduncle and pedicel shorter than calyx	**91. *Convolvulus humilis***
–	Flowers distinctly pedunculate, the peduncle and pedicel several times longer than the calyx	**17**
17	Corolla 7–10 mm long, entirely blue	**88. *Convolvulus pentapetaloides***
–	Corolla 14–40 mm long, usually blue, white and yellow banded	**18**
18	Capsule pubescent; sepals with distinct, different coloured lower and upper portions, pubescent (*Convolvulus tricolor*)	**19**
–	Capsule glabrous; sepals without distinct upper and lower areas, glabrous to pubescent	**89. *Convolvulus meonanthus***
19	Upper portion of sepals acute to acuminate, longer than basal portion	**90. Convolvulus tricolor subsp. cupanianus**
–	Upper prtion of sepals obtuse to acute, shorter than or equalling the basal portion	**90. Convolvulus tricolor subsp. tricolor**
20	Cushion plants with prostrate stems with or without short flowering stems	**21**
–	Plants not cushion forming; flowering stems at least 5 cm high	**25**
21	Flowering stems absent or extremely short	**22**
–	Short but distinct flowering stems present	**24**
22	Leaves glabrous above, midrib only distinct	**161. *Convolvulus libanoticus***
–	Leaves sericeous above, lateral veins distinct (*Convolvulus boissieri*)	**23**
23	Indumentum of sepals more or less spreading and distinct from that of the leaves (Spain)	**154. Convolvulus boissieri subsp. boissieri**
–	Indumentum of leaves and sepals similar (Balkans)	**154. Convolvulus boissieri subsp. compactus**
24	Lateral veins distinct (Bulgaria)	**155. *Convolvulus suendermannii***
–	Lateral veins not distinct (widespread).	**156. *Convolvulus lineatus***
25	Mature stems woody and divaricately branched	**167. *Convolvulus dorycnium***
–	Mature stems not woody, or only so below, not divaricately branched	**26**
26	Plants silvery-sericeous, the hairs appressed	**27**
–	Plants densely pubescent to pilose, some hairs conspicuously spreading	**33**
27	Outer sepals more or less cordate at the base, conspicuously gibbous	**153. *Convolvulus holosericeus***
–	Outer sepals neither cordate basally nor gibbous	**28**
28	Flowers in dense heads overtopped by bracts, which form a kind of involucre	**29**
–	Flowers in lax terminal groups, the pedicels usually obvious	**31**
29	Ovary and capsule glabrous (Spain and France)	**165. *Convolvulus lanuginosus***
–	Ovary and capsule hirsute	**30**
30	Stem leaves distant, few; sepals all acuminate (Caucasus)	**151. Convolvulus calvertii subsp. ruprechtii**
–	Stem leaves imbricate, numerous, some sepals obtuse (Adriatic region)	**166. *Convolvulus cneorum***
31	Cliff plant with long pendent sems	**158. *Convolvulus argyrothamnos***
–	Plant of open slopes, decumbent to erect	**32**
32	Plant usually < 25 cm high; extreme base of leaf widened and scarious	**156. *Convolvulus lineatus***
–	Plant usually 20–50 cm high; extreme base of leaf not widened and scarious	**157. *Convolvulus oleifolius***
33	Inflorescence with flowers clustered at apex of stem	**34**
–	Inflorescence lax	**146. *Convolvulus cantabrica***
34	Sepals densely pilose	**151. Convolvulus calvertii subsp. calvertii**
–	Sepals with scattered spreading hairs	**152. *Convolvulus sericocephalus***

**10. Key to species in the Levant**

**Table:** 

1	Annual herbs; plants slender, herbaceous, never rhizomatous or woody at the base	**2**
–	Perennial herbs or undershrubs, usually robust, the base woody or, if herbaceous, rootstock rhizomatous	**7**
2	Flowers densely clustered at the apex, peduncles and pedicels, absent or, if present, very short	**91. *Convolvulus humilis***
–	Flowers solitary or in lax cymes	**3**
3	Sepals terminating in a prominent mucro 1.5–3 mm long	**18. *Convolvulus coelesyriacus***
–	Sepals acute to obtuse, lacking a distinct terminal mucro	**4**
4	Leaves petiolate; leaf blade abruptly narrowed to a truncate or cordate base	**5**
–	Leaves clearly sessile or leaf blade gradually narrowed to base	**8**
5	Flowers blue; leaves entire, fruiting peduncle not strongly recurved	**87. *Convolvulus siculus***
–	Flowers pinkish, leaves crenate; peduncle strongly recurved in fruit	**7. *Convolvulus fatmensis***
6	Corolla 7–10 mm long, entirely blue	**88. *Convolvulus pentapetaloides***
–	Corolla 14–40 mm long, usually blue, white and yellow banded	**90. *Convolvulus tricolor***
7	Leaves distinctly petiolate, base of lamina hastate, sagittate, cordate, rounded or very broadly cuneate	**8**
–	Leaves sessile or base of lamina tapering at base	**22**
8	Inner sepals conspicuously longer than outer sepals; plant glabrous (including midpetaline bands); corolla yellow	**9**
–	Inner sepals equalling or shorter than outer sepals; plant hirsute at least on the midpetaline bands; corolla pink or white (yellowish only in *Convolvulus palaestinus*)	**10**
9	Rigidly erect, divaricately branched plant	**2. *Convolvulus pseudoscammonia***
–	Trailing plant, stems not divaricately branched	**1. *Convolvulus scammonia***
10	Flowers in axillary, pedunculate, pilose heads	**101. *Convolvulus glomeratus***
–	Flowers solitary or in lax cymes, never arranged in dense pilose heads	**11**
11	Leaf base broadly cuneate to rounded; undershrub with tomentose leaves and solitary white flowers	**72. *Convolvulus persicus***
–	Leaf base hastate, sagittate or truncate; trailing or twining herbs, only slightly woody at base; flowers or not, commonly pinkish	**12**
12	Sepals < 6 mm long; leaves never deeply incised or lobed	**13**
–	Sepals > 6 mm long; upper leaves often incised or dentate	**15**
13	Leaf margin entire; fruiting peduncles not recurved	**14**
–	Leaf margin strongly crenate; fruiting pedicels recurved	**7. *Convolvulus fatmensis***
14	Sepals < 4.5 mm long, scarious-margined	**4. *Convolvulus arvensis***
–	Sepals 5.5–6 mm long, margins not scarious	**12. *Convolvulus longipedicellatus***
15	Sepals 10–15 mm long, leaf margin entire to obscurely undulate	**11. *Convolvulus betonicifolius***
–	Sepals 6–10 mm long, leaf margin crenate dentate to incised	**16**
16	Leaves dimorphic, at least the upper ones incised; ovary glabrous; corolla pink or white	**17**
–	Leaves not dimorphic nor upper leaves incised except sometimes in *Convolvulus palaestinus*, which has a yellow corolla; ovary hirsute or glabrous	**18**
17	Leaves sericeous, the segments of the upper leaves linear	**22. Convolvulus althaeoides subsp. tenuissimus**
–	Leaves pubescent to pilose, the segments of the upper lanceolate to oblong-ellipti	**22. Convolvulus althaeoides subsp. althaeoides**
18	Leaves glabrous with a ciliate margin	**13. *Convolvulus cassius***
–	Leaves densely pubescent to tomentose	**19**
19	Corolla yellow; leaves commonly dimorphic	**15. *Convolvulus palaestinus***
–	Corolla pink or white, leaves never dimorphic	**20**
20	Ovary glabrous; sepals obovate to broadly elliptic, leaves sinuate, coarsely pubescent	**14. *Convolvulus stachydifolius***
–	Ovary hirsute (? rarely glabrous); sepals ovate to elliptic; leaves undulate to crenate, softly tomentose	**21**
21	Leaves with spreading hairs; sepals acute; corolla white to pale pink	**17. *Convolvulus germaniciae***
–	Leaves uniformly short-tomentose; sepals apiculate; corolla pink	**16. *Convolvulus galaticus***
22	Flowers several in axillary heads; leaves, stem and sepals densely villous	**23**
–	Flowers in terminal heads (sometimes a few axillary also) or not in head-like structures; stem and leaves glabrous, pubescent, pilose or sericeous	**27**
23	Bracts ovate, cordate, up to 3 cm wide; leaves reticulate below	**185. *Convolvulus reticulatus***
–	Bracts oblong-elliptic or lanceolate, up to 1.5 cm wide; leaves not reticulate below	**24**
24	Flower heads sessile or subsessile	**25**
–	Flower heads pedunculate	**26**
25	Lower, old stems spinescent, stems ascending, < 40 cm long	**176. *Convolvulus lanatus***
–	Plant unarmed, stems procumbent, > 40 cm long	**177. *Convolvulus secundus***
26	Bracts linear to lanceolate, < 0.5 cm wide	**179. *Convolvulus jordanensis***
–	Bracts oblong-elliptic or lanceolate 0.5–1.5 cm wide	**178. *Convolvulus spicatus***
27	Plants with branched stems forming a lax open inflorescence	**28**
–	Plants with compact terminal inflorescences or cushion plants, never forming a much branched open inflorescence	**33**
28	Corolla white, c. 1 cm long; stem glabrous	**113. *Convolvulus chondrilloides***
–	Corolla pink or with pink midpetaline bands, > 1 cm long, stem appressed hairy to pilose	**29**
29	Ovary glabrous	**30**
–	Ovary hirsute	**32**
30	Corolla 1–1.5 cm long; stems flexible, herbaceous, pilose	**112. *Convolvulus pilosellifolius***
–	Corolla 2–2.5 cm long; stems stiff and woody, appressed pubescent	**31**
31	Sepals at apex abruptly narrowed and mucronate	**167. Convolvulus dorycnium subsp. dorycnium**
–	Sepals gradually narrowed to an acute or acuminate apex	**167. Convolvulus dorycnium subsp. oxysepalus**
32	Stems and leaves densely spreading pilose; leaves all oblong (Gaziantep region)	**147. *Convolvulus aucheri***
–	Stems and leaves pubescent or thinly pilose; leaves variously shaped, often oblanceolate-spathulate near the base of the stem, rarely oblong	**146. *Convolvulus cantabrica***
33	Leaves and stem adpressed-sericeous	**34**
–	Leaves and stem with spreading hairs (often also sericeous) or more or less glabrous	**39**
34	Sepals with a conspicuous pouch near base	**35**
–	Sepals lacking a conspicuous pouch near base	**36**
35	Sepals < 10 × 8 mm	**153. Convolvulus holosericeus subsp. holosericeus**
–	Sepals > 11 × 11 mm	**153. Convolvulus holosericeus subsp. macrocalycinus**
36	Dwarf cushion-forming shrublet	**37**
–	Low perennial with woody base and distinct ascending or erect stems	**38**
37	Sepals with conspicuous spreading hairs; leaves with forked central vein	**154. Convolvulus boissieri subsp. compactus**
–	Sepals with appressed hairs; leaves with one simple central vein	**160. *Convolvulus phrygius***
38	Plant usually < 25 cm high; extreme base of leaf widened and scarious; sepals sericeous and spreading pilose	**156. *Convolvulus lineatus***
–	Plant usually 20–50 cm high; extreme base of leaf not widened and scarious; sepals with spreading hairs only	**39**
39	Flowers clustered, usually at apex of peduncle-like stem; plants cushion-forming or not	**40**
–	Flowers solitary or clearly separate in a lax terminal cyme; plants strictly cushion forming	**44**
40	Outer sepals bicoloured with a pale base and green apex	**41**
–	Outer sepals uniformly coloured	**151. *Convolvulus calvertii***
41	Corolla < 1.5 cm long	**112. *Convolvulus pilosellifolius***
–	Corolla > 1.5 cm long	**42**
42	Stems, leaves, sepals and ovary glabrous or nearly so	**164. *Convolvulus carduchorum***
–	Stems, leaves, sepals and ovary conspicuously pilose	**43**
43	Plant < 15 cm high; sepals with long caudate apex	**163. *Convolvulus cataonicus***
–	Plant usually > 25 cm high; sepals acute to acuminate	**146. *Convolvulus cantabrica***
44	Plant with conspicuous spreading hairs; corolla pink	**162. *Convolvulus assyricus***
–	Plant subglabrous or thinly appressed pubescent; corolla white or pale pink	**161. *Convolvulus libanoticus***

**11. Key to species in the Arabian Peninsula (including Socotra)**

**Table:** 

1	Plants annual; all parts of the plant herbaceous	**2**
–	Plants perennial; plants usually woody below, but, if entirely herbaceous, with perennial rhizomatous roots	**4**
2	Leaves crenate; sepals obtuse; corolla pink	**7. *Convolvulus fatmensis***
–	Leaves entire; sepals acute; corolla blue	**3**
3	Pedicels absent, bracteole appressed to base of calyx, filiform to more or less lanceolate	**87. Convolvulus siculus subsp. siculus**
–	Pedicels borne at least 5 mm below calyx, always filiform	**87. Convolvulus siculus subsp. elongatus**
4	Trailing or twining herbs with leaves abruptly narrowed at the base into a distinct petiole; plants not with woody stems nor flowers arranged in head-like clusters	**5**
–	Herbs or shrubs, never twining or trailing, leaves gradually narrowed at the base, lacking a distinct petiole but if petiolate, stems woody or flowers in head-like clusters	**9**
5	Corolla > 15 mm long; flowers usually solitary	**6**
–	Corolla < 12 mm long; flowers 1–5 in axillary cymes	**7**
6	Sepals < 4.5 mm long; plant usually glabrescent	**4. *Convolvulus arvensis***
–	Sepals > 6 mm long; plant pubescent	**37. *Convolvulus sagittatus***
7	Leaves crenate; fruiting peduncles deflexed	**7. *Convolvulus fatmensis***
–	Leaves not crenate, entire apart from (sometimes) forked auricles or weakly sinuate margins; fruiting peduncles not deflexed	**8**
8	Central leaf lobe ovate to triangular in outline; basal auricles not forked; plant often twining	**36. *Convolvulus farinosus***
–	Central leaf lobe linear-oblong in outline; basal auricles sometimes forked; plant usually trailing	**35. *Convolvulus aschersonii***
9	Leaves with lamina abruptly narrowed at base and clearly separate from the (sometimes very short) petiole; stigmas clavate or at least thickened upwards	**10**
–	Leaves sessile or with lamina attenuate at base with no distinct petiole; stigmas various	**14**
10	Flowers in hirsute heads, the hairs spreading and somewhat concealing the calyx	**11**
–	Flowers solitary; sepals glabrous or sericeous, easily visible (Oman)	**70. *Convolvulus leiocalycinus***
11	Flower heads pedunculate	**12**
–	Flower heads sessile or nearly so	**13**
12	Leaves glabrous; stems woody, sometimes spinescent	**100. *Convolvulus virgatus***
–	Leaves pubescent; stems herbaceous (except below), never spinescent	**101. *Convolvulus glomeratus***
13	Spiny undershrub; leaves all alternate; flower clusters of up to 6 flowers	**104. *Convolvulus hystrix***
–	Unarmed undershrub; leaves often opposite towards branch tips; flowers usually 1–2 together	**102. *Convolvulus oppositifolius***
14	Branches spinescent	**15**
–	Branches not spiny although sometimes woody and rigid	**21**
15	Flowers solitary or clustered, sessile; sterile spinescent peduncles absent	**16**
–	Flowers solitary or clustered borne on spinescent peduncles; sterile peduncles often present as spines (Oman)	**142. *Convolvulus acanthocladus***
16	Sepals addressed pubescent; stigma clavate, shorter than style arm (Socotra)	**17**
–	Sepals with spreading hairs; stigmas linear, co-extensive with style arm	**18**
17	Ovary glabrous; sepals broadly obovate-elliptic	**106. *Convolvulus kossmatii***
–	Ovary hirsute; sepals oblong-lanceolate	**107. *Convolvulus semhaensis***
18	Sepals long-pilose with woolly hairs (Oman)	**189. *Convolvulus ulicinus***
–	Sepals densely pubescent to tomentose but lacking long woolly hairs	**19**
19	Plant with long, slender, spine-tipped branches, short spinescent side shoots absent or very few	**172. Convolvulus oxyphyllus subsp. oxyphyllus**
–	Plant with stout spinescent primary branches and numerous short (< 4 cm long), usually stout lateral spine-like shoots	**20**
20	Leaves with rigid, acute apex, basal leaves not undulate; flowers usually in clusters of > 1, clusters elongating at maturity	**172. Convolvulus oxyphyllus subsp. oxycladus**
–	Leaves with soft obtuse to subacute apex, the basal leaves often undulate; flowers mostly solitary	**173. *Convolvulus hamrinensis***
21	Flowers arranged in sessile or pedunculate, pilose clusters	**22**
–	Flowers arranged in a lax open inflorescence or sessile or shortly pedunculate along an elongate axis, not in pilose cluster	**28**
22	Nearly leafless subshrub with glabrous to adpressed pubescent stem and leaves; leaves minute, linear, < 5 mm long (Hadramaut)	**103. *Convolvulus scopulatus***
–	Leafy plants at least basally; leaves and stem pubescent, pilose or villous; leaves > 2 cm long	**23**
23	Sepals bicoloured, base colourless, apex greenish; ovary glabrous	**24**
–	Sepals uniformly coloured green; ovary hirsute	**25**
24	Flowers usually more or less solitary, sometimes clustered; sepals oblong with an acute apex	**112. *Convolvulus pilosellifolius***
–	Flowers in heads, very rarely solitary; sepals gradually narrowed to an acute to long acuminate apex	**111. *Convolvulus prostratus***
25	Heads subsessile; dwarf mountain plant with stems < 10 cm high (Asir)	**183. *Convolvulus asyrensis***
–	Heads pedunculate; desert plant with stems usually >15 cm	**26**
26	Stems and leaves with long villous hairs; style pilose	**181. Convolvulus cephalopodus subsp. bushiricus**
–	Stems and leaves shortly hairy; style glabrous or nearly so	**181. Convolvulus cephalopodus subsp. cephalopodus**
27	Sepals bicoloured; base colourless, apex greenish	**112. *Convolvulus pilosellifolius***
–	Sepals uniformly green	**28**
28	Leaves sinuate margined; basal rosette persistent; plant entirely herbaceous (Abd ul Kuri Island)	**115. *Convolvulus grantii***
–	Leaf margins entire; basal rosette absent or ephemeral; plant usually woody at least below	**29**
29	Ovary hirsute; corolla deeply lobed; undershrub to 3 m with very rigid branches and peduncles arising at 90° to each other (Saudi Arabia)	**120. *Convolvulus erinaceus***
–	Ovary glabrous; corolla shallowly lobed; herbs or undershrubs to 50 cm, branching not as above	**30**
30	Flowers sessile or nearly so, forming a long narrow inflorescence	**31**
–	Flowers borne on conspicuous, often rigid peduncles; inflorescence open	**32**
31	Flowers solitary (Socotra)	**105. *Convolvulus socotranus***
–	Flowers in very shortly pedunculate cymes (Yemen)	**114. *Convolvulus sericophyllus***
32	Peduncles bearing monochasial cymes, inflorescence pubescent (Oman)	**118. *Convolvulus peninsularis***
–	Peduncles mostly bearing single flowers; inflorescence almost glabrous except sepals	**33**
33	Basal leaves villous, ephemeral; bracts linear	**117. *Convolvulus hildebrandtii***
–	Basal leaves sericeous, persistent; bracts oblong or oblanceolate	**116. *Convolvulus sarmentosus***

**12. Key to species in the Indo-Iranian region**

**Table:** 

1	Plants annual; all parts of the plant herbaceous	**2**
–	Plants perennial; plants usually woody below, but, if entirely herbaceous, with perennial rhizomatous roots	**7**
2	Lamina ovate, abruptly narrowed into a distinct petiole	**3**
–	Leaves oblong, lanceolate or oblong (rarely ovate), sessile or narrowed at base with petiole not clearly demarcated	**4**
3	Leaves crenate; sepals obtuse; corolla pink	**7. *Convolvulus fatmensis***
–	Leaves entire; sepals acute; corolla blue	**87. *Convolvulus siculus***
4	Flowers solitary, corolla blue	**88. *Convolvulus pentapetaloides***
–	Flowers clustered or grouped, very rarely solitary; corolla pinkish	**5**
5	Flowers 3–6 in sessile axillary clusters	**93. *Convolvulus rhyniospermus***
–	Flowers up to 3 in pedunculate cymes	
6	Sepals glabrous	**110. Convolvulus rottlerianus subsp. stocksii**
–	Sepals adpressed-pilose	**110a. Convolvulus rottlerianus subsp. rottlerianus**
7	Trailing or twining herbs with leaves abruptly narrowed at the base into a distinct petiole; plants not with woody stems nor flowers arranged in head-like clusters	**8**
–	Herbs or shrubs, never twining or trailing, leaves gradually narrowed at the base, lacking a distinct petiole but if petiolate, stems woody or flowers in head-like clusters	**15**
8	Sepals < 5 mm long	**9**
–	Sepals > 6 mm long	**10**
9	Leaves crenate; fruiting peduncles deflexed	**7. *Convolvulus fatmensis***
–	Leaves entire; fruiting peduncles not deflexed	**4. *Convolvulus arvensis***
10	Plant completely glabrous; corolla yellow	**1. *Convolvulus scammonia***
–	Plant pubescent at least on the midpetaline bands and usually elsewhere; corolla variously coloured	**11**
11	Sepals rectangular in form (Afghanistan)	**12**
–	Sepals variously shaped, never rectangular in form (India, Iran, Iraq)	**13**
12	Leaves entire, densely hirsute	**74. *Convolvulus lanjouwii***
–	Leaves sinuate-margined, sparsely pubescent	**75. *Convolvulus rectangularis***
13	Ovary and capsule hirsute; sepals bicoloured with distinct apical portion; leaves entire to undulate	**11. *Convolvulus betonicifolius***
–	Ovary and capsule glabrous; sepals lacking a distinctly coloured apical portion; leaves undulate to sinuate or dentate	**14**
14	Corolla < 1.2 cm long, white or cream (India)	**10. *Convolvulus rufescens***
–	Corolla 2.5–3.5 cm long, pink or purplish	**14. *Convolvulus stachydifolius***
15	Leaves distinctly petiolate, the lamina clearly separate from the petiole	**16**
–	Leaves sessile or with lamina attenuate or cuneate at base with no distinct petiole	**19**
16	Flowers in pedunculate hirsute heads	**17**
–	Flowers solitary	**18**
17	Leaves glabrous; stems woody, sometimes spinescent	**100. *Convolvulus virgatus***
–	Leaves pubescent; stems herbaceous (except below), never spinescent	**101. *Convolvulus glomeratus***
18	Spiny undershrub; leaves glabrous to finely sericeous, < 1 cm wide	**70. *Convolvulus leiocalycinus***
–	Unarmed undershrub; leaves tomentose, 1–3.5 cm wide	**72. *Convolvulus persicus***
19	Plant spiny or with spinescent branches	**20**
–	Plant unarmed, although branches sometimes rigid and hard	**30**
20	Flowers in a terminal cluster towards the apex of the stem	**21**
–	Flowers mostly or entirely axillary	**22**
21	Ovary and style glabrous; primary branches only spinescent	**188. *Convolvulus oxysepalus***
–	Ovary and style hirsute; lateral shoots spinescent as well as primary branches	**145. *Convolvulus turrillianus***
22	Flowers borne on spinescent peduncles; sterile spines often also present	**23**
–	Flowers sessile or nearly so; sterile spines usually absent	**27**
23	Outer sepals much shorter than the inner sepals	**140. *Convolvulus spinosus***
–	Outer and inner sepals similar in length	**24**
24	Sepals 3–10 mm long	**25**
–	Sepals 10–11 mm long	**138. *Convolvulus fruticosus***
25	Sepals long-pilose	**26**
–	Sepals adpressed pubescent	**141. *Convolvulus argyracanthus***
26	Sepals 4–5 mm long, thinly pilose; corolla 1.5–1.7 cm long	**143. *Convolvulus iranicus***
–	Sepals 7–10 mm long, densely pilose; corolla 1.6–2.5 cm long	**142. *Convolvulus acanthocladus***
27	Sepals 12–15 mm long	**144. *Convolvulus urosepalus***
–	Sepals < 8 mm long	**28**
28	Plant with long, slender, spine-tipped branches, short spinescent side shoots absent or very few	**172. Convolvulus oxyphyllus subsp. oxyphyllus**
–	Plant with stout spinescent primary branches and numerous short (< 4 cm long), usually stout lateral spine-like shoots	**29**
29	Leaves with rigid, acute apex, basal leaves not undulate; flowers usually in clusters of > 1, clusters elongating at maturity	**172. Convolvulus oxyphyllus subsp. oxycladus**
–	Leaves with soft obtuse to subacute apex, the basal leaves often undulate; flowers mostly solitary	**173. *Convolvulus hamrinensis***
30	Flowers arranged in dense terminal or axillary heads or clusters	**31**
–	Flowers variously arranged in a lax, branched inflorescence	**54**
31	Heads terminal (occasionally with a few flowers below the terminal head)	**32**
–	Heads axillary, sometimes terminal as well	**40**
32	Plant with woody stems	**33**
–	Plant with herbaceous stems, woody only at the base	**35**
33	Leaves with impressed veins, hairs dense but short; sepals < 10 mm long; Corolla < 1.2 cm long	**190. *Convolvulus scindicus***
–	Leaves without impressed veins; sepals > 10 mm long; corolla > 1.5 cm long	**34**
34	Ovary and style glabrous; stigmas c. 6 mm long	**188. *Convolvulus oxysepalus***
–	Ovary and style hairy; stigmas c. 3 mm long	**145. *Convolvulus turrillianus***
35	Plant silvery-sericeous; inflorescence very lax with individual peduncles and pedicels clearly visible	**36**
–	Plant not sericeous or, if somewhat so, inflorescence of dense heads with individual peduncles and pedicels not easily visible	**37**
36	Outer sepals with a conspicuous pouch; plant to 30 cm	**153. *Convolvulus holosericeus***
–	Outer sepals lacking a conspicuous pouch; plant usually < 10cm	**156. *Convolvulus lineatus***
37	Leaves linear, < 0.2 cm wide	**148. *Convolvulus schirazianus***
–	Leaves oblong-elliptic or oblanceolate, > 5 cm wide	**38**
38	Stem with spreading hairs	**39**
–	Stem with appressed hairs	**149. *Convolvulus commutatus***
39	Heads solitary, strictly terminal	**151. Convolvulus calvertii subsp. calvertii**
–	Heads with 1–2 flower groups below terminal heads	**150. *Convolvulus elymaiticus***
40	Lower peduncles absent or < 0.5 cm long; heads sessile or nearly so	**41**
–	Lower peduncles well-developed, > 1 cm long; heads mostly distinctly pedunculate	**44**
41	Cushion herbs from which arise erect flowering stems, ovary comose	**184. *Convolvulus aitchisonii***
–	Plants not cushion forming, ovary glabrous or hirsute	**42**
42	Sepals bicoloured, the base pale, apex green; ovary glabrous	**111. *Convolvulus prostratus***
–	Sepals of uniform colour; ovary hirsute at apex	**43**
43	Branches rigid and woody; leaves apiculate; corolla < 1.5 cm long; corolla < 1.5 cm long; flowers usually 1–3	**172. *Convolvulus oxyphyllus***
–	Branches not noticeably rigid; leaves acute but not apiculate; corolla > 1.5 cm long; heads many-flowered	**186. *Convolvulus stapfii***
44	Ovary hirsute, at least at apex	**45**
–	Ovary glabrous	**47**
45	Sepals 14–16 mm, ovate with a long aristate point, almost half its length	**187. *Convolvulus cephalophorus***
–	Sepals 10–12 mm, lanceolate to ovate, acuminate but not long-aristate	**46**
46	Stems and leaves with long villous hairs; style pilose	**181. Convolvulus cephalopodus subsp. bushiricus**
–	Stems and leaves shortly hairy; style glabrous or nearly so	**181. Convolvulus cephalopodus subsp. cephalopodus**
47	Plant densely brown-velvety-tomentose; leaves reticulate	**48**
–	Leaf indumentum not as above; leaves not reticulate	**49**
48	Stem stout, 4–5 mm wide; bracteoles elliptic, 4–5 mm wide, sepals obovate	**185. Convolvulus reticulatus subsp. waltherioides**
–	Stem relatively slender, < 3 mm wide; bracteoles lanceolate, 2–3 mm wide; sepals lanceolate	**185. Convolvulus reticulatus subsp. reticulatus**
49	Sepals bicoloured; base colourless, apex greenish	**50**
–	Sepals uniformly coloured green	**52**
50	Corolla 1.7–2.5 cm long, ovary and capsule hirsute	**146. *Convolvulus cantabrica***
–	Corolla 1–1.5 cm long; ovary and capsule glabrous	**51**
51	Flowers usually more or less solitary, sometimes laxly clustered, sepals oblong with an acute apex	**112. *Convolvulus pilosellifolius***
–	Flowers always in dense heads, sepals tapered to an acute to long acuminate apex	**111. *Convolvulus prostratus***
52	Only lower heads pedunculate; heads on upper part of stem sessile	**53**
–	All heads distinctly pedunculate except perhaps the uppermost	**182. *Convolvulus euphraticus***
53	Bracts < 3 × 1 cm, lanceolate (Iraq–Iran)	**174. *Convolvulus kotschyanus***
–	Bracts mostly 3–4 × 1.2–2 cm, ovate (Afghanistan)	**175. *Convolvulus pyrrotrichus***
54	Sepals bicoloured, pale below with a green apex; plants with herbaceous stems, flowers somewhat clustered	**55**
–	Sepals of one colour; plants commonly with woody rigid stems, few leaves and flowers well separated	**56**
55	Corolla < 1.5 cm long; ovary and capsule glabrous; sepals oblong-obovate	**112. *Convolvulus pilosellifolius***
–	Corolla 1.7–2.5 cm long; ovary and capsule hirsute; sepals ovate to lanceolate, acuminate	**146. *Convolvulus cantabrica***
56	Sepals glabrous or nearly so	**57**
–	Sepals pubescent, canescent or otherwise hirsute	**63**
57	Stems completely glabrous	**113. *Convolvulus chondrilloides***
–	Stems adpressed pubescent	**58**
58	Corolla 1.7–2 cm long, pink	**59**
–	Corolla < 1. 5 cm long, white or very pale pink	**60**
59	Sepals obovate, mucronate, c. 5 mm long	**131. Convolvulus pseudocantabrica subsp. pseudocantabrica**
–	Sepals oblong, acuminate, c. 7 mm long	**131. Convolvulus peudocantabrica subsp. askabadensis**
60	Leaves filiform	**61**
–	Leaves linear, oblong or oblanceolate	**62**
61	Sepals ovate	**128. *Convolvulus kurdistanicus***
–	Sepals obovate-elliptic	**129. *Convolvulus koieanus***
62	Sepals 6–7 × 3–4 mm; inflorescence narrow, few-flowered	**127. *Convolvulus sarothrocladus***
–	Sepals 4–5 × 2 mm; inflorescence commonly much branched and many flowered	**125. *Convolvulus eremophilus***
63	Stems appressed hairy, finely sericeous to strigose	**64**
–	Stems with spreading hairs at least below	**69**
64	Sepals tiny, suborbicular, c. 2 mm; plant divaricately branched	**130. *Convolvulus gracillimus***
–	Sepals > 3 mm long, longer than broad; plant not divaricately branched	**65**
65	Sepals obtuse to rounded; corolla deeply lobed; inflorescence much branched forming an intricate mass	**120. *Convolvulus erinaceus***
–	Sepals acute, acuminate or obtuse and mucronate, always terminating in a point. Corolla at most shallowly lobed; branching not so extensive as to form an intricate mass	**66**
66	Corolla 0.8–1 cm long; ovary pubescent	**121. *Convolvulus hamadae***
–	Corolla > 1.2 cm long; ovary glabrous	**67**
67	Corolla pink	**68**
–	Corolla white	**119. *Convolvulus leptocladus***
68	Sepals lanceolate, elliptic or oblong, acuminate; stems not leafy, very rigid	**167. Convolvulus dorycnium subsp. oxysepalus**
–	Sepals obovate, abruptly narrowed to a muconate apex; stems leafy and somewhat herbaceous	**168. Convolvulus dorycnium subsp. subhirsutus**
69	Stem and leaves white-sericeous	**126. *Convolvulus lindbergii***
–	Stem and leaves not white-sericeous	**70**
70	Branches slender, not very rigid; leaves lanceolate to ovate	**123. *Convolvulus divaricatus***
–	Branches short, stiff, relatively stout; leaves linear-oblong	**125. *Convolvulus eremophilus***

**13. Key to species in the Former Soviet Union**

**Table:** 

1	Plant an annual herb; flowers solitary, blue	**88. *Convolvulus pentapetaloides***
–	Plant perennial, herbaceous or woody; flowers pink, white or yellow	**2**
2	Trailing or twining herbs with leaves abruptly narrowed at the base into a distinct petiole; plants not with woody stems nor flowers arranged in head-like clusters	**3**
–	Herbs or shrubs, never twining or trailing, leaves gradually narrowed at the base, lacking a distinct petiole but if petiolate, stems woody	**7**
3	Sepals < 5 mm long	**4. *Convolvulus arvensis***
–	Sepals > 5 mm long	**4**
4	Corolla < 2.8 cm long, pink, flowers usually solitary	**5**
–	Corolla >2.8 cm long, white, yellowish or pink, flowers usually more than one	**6**
5	Leaves with an elongated strap-shaped central lobe	**5. Convolvulus chinensis subsp. chinensis**
–	Leaves triangular in form	**5. Convolvulus chinensis subsp. triangularis**
6	Plant completely glabrous; inner sepals longer than outer sepals	**1. *Convolvulus scammonia***
–	Plant hirsute; inner sepals equalling or shorter than outer sepals	**11. *Convolvulus betonicifolius***
7	Undershrubs with petiolate leaves, the lamina abruptly narrowed at base	**8**
–	Herbs or undershrubs with sessile leaves or leaves gradually narrowed into an indistinct petiole	**9**
8	Spiny undershrub; leaves glabrous to finely sericeous, < 1 cm wide	**70. *Convolvulus leiocalycinus***
–	Unarmed undershrub; leaves tomentose, 1–3.5 cm wide	**72. *Convolvulus persicus***
9	Plant spiny or with spinescent branches	**10**
–	Plant unarmed, although branches sometimes rigid and hard	**14**
10	Flowers in a terminal inflorescence	**11**
–	Flowers axillary	**12**
11	Branches all spinescent; lower leaves oblanceolate-obovate; flowers 1–several in a terminal cluster	**137. *Convolvulus spinifer***
–	Only the old lower branches spinescent; lower leaves linear to narrowly oblanceolate; flowers in a terminal cyme	**134. *Convolvulus grigorjevii***
12	Flowers borne on spinescent peduncles; sterile spines often also present	**13**
–	Flowers sessile or nearly so; sterile spines absent	**136. *Convolvulus tragacanthoides***
13	Outer sepals glabrous, much larger than the inner sepals	**139. *Convolvulus gortschakovii***
–	Outer sepals pubescent, equalling or smaller than the inner sepals	**138. *Convolvulus fruticosus***
14	Flowers arranged in terminal heads or clusters, occasionally with a few flowers on the stem below the main cluster; stems herbaceous (if woody, see 134. *Convolvulus krauseanus*)	**15**
–	Flowers variously arranged in lax, branched inflorescences, stems often woody	**20**
15	Leaves, stem and sepals all silvery-sericeous	**16**
–	Leaves, stem or sepals with conspicuous spreading hairs, sometimes sericeous as well	**18**
16	Inflorescence a compact head, pedicels and peduncles not clearly visible	**151. Convolvulus calvertii subsp. ruprechtii**
–	Inflorescence lax, peduncles and pedicels easily visible	**17**
17	Outer sepals conspicuously pouched (Crimea); plant to 30 cm	**153. *Convolvulus holosericeus***
–	Outer sepals lacking a conspicuous pouch; plant rarely exceeding 15 cm	**156. *Convolvulus lineatus***
18	Stem with spreading hairs	**151. Convolvulus calvertii subsp. calvertii**
–	Stem with appressed hairs	**19**
19	Sepals with spreading hairs	**152. *Convolvulus sericocephalus***
–	Sepals with appressed hairs	**149. *Convolvulus commutatus***
20	Low perennial with linear, sericeous leaves; flowers solitary	**132. *Convolvulus ammannii***
–	Erect or ascending plants with stems usually > 10 cm tall; flowers mostly clustered	**21**
21	Sepals bicoloured, with pale base and green apex; plants with herbaceous stems and flowers somewhat clustered	**22**
–	Sepals of one colour; plants commonly with woody rigid stems, few leaves and flowers well separated	**23**
22	Corolla < 1.5 cm long; ovary and capsule glabrous; sepals oblong-obovate	**112. *Convolvulus pilosellifolius***
–	Corolla 1.7–2.5 cm long; ovary and capsule hirsute; sepals ovate to lanceolate, acuminate	**146. *Convolvulus cantabrica***
23	Sepals glabrous or nearly so	**24**
–	Sepals pubescent, canescent or otherwise hirsute	**26**
24	Corolla pink; sepals 5–7 mm long; plant divaricately branched	**25**
–	Corolla white; sepals 4–5 mm; plant not divaricately branched.	**125. *Convolvulus eremophilus***
25	Sepals obovate, mucronate, c. 5 mm long	**131. Convolvulus pseudocantabrica subsp. pseudocantabrica**
–	Sepals oblong, acuminate, c. 7 mm long	**131. Convolvulus peudocantabrica subsp. askabadensis**
26	Stems appressed pubescent, finely sericeous to strigose	**27**
–	Stems densely sericeous, pubescent or pilose, some hairs spreading at least below	**29**
27	Sepals obtuse to rounded; corolla deeply lobed; inflorescence much branched forming an intricate mass	**120. *Convolvulus erinaceus***
–	Sepals acute, acuminate or obtuse and mucronate, always terminating in a point; corolla at most shallowly lobed; branching not so extensive as to form an intricate mass	**28**
28	Corolla white, 0.8–1 cm long; ovary pubescent	**121. *Convolvulus hamadae***
–	Corolla pink, > 1. 2 cm long; ovary glabrous	**167. Convolvulus dorycnium subsp. subhirsutus**
29	Stem and leaves white-sericeous	**30**
–	Stem and leaves not white-sericeous	**31**
30	Inflorescence of very dense, axillary clusters; lower leaves clearly oblanceolate; lower branches often somewhat spinescent	**134. *Convolvulus grigorjevii***
–	Inflorescence scape-like, flowers1-several at apex of stem; leaves strictly linear, branches never spinescent	**135. *Convolvulus krauseanus***
31	Branches slender, not very rigid	**32**
–	Branches short, stiff, relatively stout	**34**
32	Leaves linear-lanceolate, up to 3 mm wide; sepals lanceolate, acuminate; stems subsericeous	**122. *Convolvulus subsericeus***
–	Leaves lanceolate to ovate, 3–15 mm wide; sepals often abruptly narrowed at apex; stems pubescent	**33**
33	Stems sparingly branched; corolla > 1.5 cm long, pink; ovary glabrous	**168. *Convolvulus tschimganicus***
–	Stems much branched; corolla < 1.5 cm long, white or pinkish; ovary usually hirsute	**123. *Convolvulus divaricatus***
34	Plant densely pubescent; leaves linear-oblanceolate	**124. *Convolvulus tujuntauensis***
–	Plant thinly pubescent; leaves linear-oblong	**125. *Convolvulus eremophilus***

**14. Key to species in East Asia**

**Table:** 

1	Leaves distinctly petiolate, the blade abruptly narrowed onto to the petiole; trailing or twining herbs	**2**
–	Leaves lacking a distinct petiole, the blade narrowed at base; undershrubs or perennial herbs with a woody rootstock, neither twining nor trailing	**5**
2	Sepals < 4.5 mm long	**4. *Convolvulus arvensis***
–	Sepals 5–7 mm long	**3**
3	Leaves glabrous or nearly so, margin entire	**5. *Convolvulus chinensis***
–	Leaves densely pubescent to tomentose, margin undulate, crenate or dentate	**4**
4	Leaf base hastate, often with bifid auricles; sepals 7–10 mm long	**8. *Convolvulus steppicola***
–	Leaf base truncate to subcordate with simple, poorly developed auricles; sepals 6–7 mm long	**9. *Convolvulus sinuatodentatus***
5	Unarmed perennials woody at base only	**6**
–	Undershrubs with spinescent branchlets	**9**
6	Stems erect, branched; leaves glabrous or adpressed hairy beneath; ovary and capsule glabrous	**131. *Convolvulus pseudocantabrica***
–	Stems prostrate to ascending but always low; leaves sericeous; ovary and capsule pubescent	**7**
7	Leaves oblong-oblanceolate, 5–25 mm wide; flowers 1–5 in compact cymes, 1.8–2.5 cm long	**156. *Convolvulus lineatus***
–	Leaves linear to linear oblanceolate, < 5 mm wide; flowers usually solitary, 1–1.6 cm long	**8**
8	Flowers mostly axillary; outer sepals 4.5–6 mm long; stems herbaceous	**132. *Convolvulus ammannii***
–	Flowers all terminal on the branches; outer sepals 6–7 mm long; stems somewhat woody and rigid	**133. *Convolvulus xanthopotamicus***
9	Sepals glabrous to thinly pubescent. Outer pair suborbicular, much wider than inner sepals	**139. *Convolvulus gortschakovii***
–	Sepals hirsute, all similar in shape and size	**10**
10	Flowers clustered at apex of peduncle-like stem	**137. *Convolvulus spinifer***
–	Flowers axillary	**11**
11	Prostrate cushion plant, the flowering branches without spines	**136. *Convolvulus tragacanthoides***
–	Erect undershrub, flowers borne on spinescent peduncles, usually (always?) with sterile stem spines towards the apex of the flowering shoots	**138. *Convolvulus fruticosus***

## Taxonomic treatment of *Convolvulus*

### Names

Accepted names are in bold italics. All names of specific, subspecific and varietal rank in the genus *Convolvulus* are accounted for in the synonomies that are provided for each species. Species now considered to belong to other genera but originally described in *Convolvulus* are not accounted for.

### Specimen citations

Type specimens and their location are cited for all recognised taxa whether species, subspecies or varieties. We have lectotypfied species where we have seen appropriate material for lectotypification but have not lectotypified where there is doubt about the selection of a lectotype. A particular problem relates to the plants described from North Africa by Maire. The types of these species were supposed to have been deposited at the Université d’Alger (AL) and are cited for AL by Sa’ad (1967). However, it seems that portions of the holotypes were removed from Algeria in 1962 and deposited in Montpelier (MPU) and perhaps Paris (P). We are not certain whether material remained in AL or if all or only some was removed.

Wherever possible, at least one specimen is cited for every country where a species is known to occur. Occasionally, a literature record is cited and in a few cases of common species, no specimen is cited as the species is assumed to be present because of its wider distribution. Records requiring confirmation are indicated with a question mark (?). Although cited specimens are limited to those seen by the authors and are representative of the species, some effort has been made to select material that is either widely distributed or likely to be available in the country where the plant occurs. Unfortunately this has not always been possible. The herbaria where these specimens are found are not cited as we are not generally aware of where they are distributed. We have seen all collections in BM, E, K, LE, OXF, P and W and sporadic examples from other herbaria if material has been loaned or images were available online.

Where a species is known from a few countries the country order in which specimens are cited is arbitrary but in cases where a species is known from many different countries the preferred order is as follows: European and Mediterranean countries are arranged from West to East beginning with the Atlantic Islands but southern African countries are arranged from South Africa northwards.

### Literature citations

We have cited references to where all type specimens were published. We have not cited references to pages in standard floras unless they add to the information in the present monograph by providing additional descriptive material, illustrations or maps. However we have cited recent works where illustrations, paintings, drawings or photographs are provided as these are often a very useful aid to identification, capturing the appearance of a particular species in a way that words do not. We have cited references to relevant literature in the discussion of infraspecific variation and taxonomic problems.

#### Convolvulus

Taxon classificationPlantaeSolanalesConvolvulaceae

L., Sp. Pl. 1: 153. 1753. (Linnaeus 1753: 153).

##### Type.

*Convolvulus arvensis* L.

##### Description.

Spiny or unarmed shrubs or subshrubs or prostrate or erect herbs, stems often twining or trailing. Leaves alternate (rarely subopposite), simple, sessile or petiolate. Flowers variously arranged, solitary or in various kinds of inflorescence, usually cymose in structure although reduced to heads, flower pairs or other arrangements; each flower subtended by a pair of small bracteoles; calyx of 5 free sepals, these usually entire, slightly to very unequal, usually of two similar outer sepals, two similar inner sepals and an asymmetric middle sepal whose two halves are dissimilar; corolla funnel-shaped with a spreading limb and a short glabrous basal tube, the limb with five hirsute external midpetaline bands which terminate in a tooth or lobe; stamens 5, included, inserted at the top of the basal tube, filaments unequal, the basal part slightly dilated, glabrous or minutely glandular, the glands sessile or shortly stipitate, anthers equal, oblong to oblong-sagittate, pollen tricolpate, more or less spherical, colpi long and broad, exine thick; ovary usually ovoid, less commonly globose or conical, hirsute or glabrous, the base with a distinct disc, bilocular, each locule with 2 ovules; styles glabrous or hirsute, filiform divided upwards into 2 (rarely 3) arms, stigmas coextensive with style arms (very rarely slightly shorter), linear or, rarely, thickened upwards and ellipsoid or clavate. Capsule bilocular or by abortion unilocular, the dehiscence loculicidal or from the base, 4-seeded or less by abortion; seeds hirsute or glabrous, smooth tuberculate or obscurely ridged, one side convex and the other flat (see Figure 15: 34, for an example) unless capsule is 1-seeded when shape is ellipsoidal.

### Species 1–22. Eurasian and North African species with leaves abruptly narrowed into a distinct petiole.

Nearly all species are trailing or twining perennial herbs with flowers in pedunculate cymes (sometimes reduced to single flowers) arising from the axils of leaf-like bracts. *Convolvulus coelesyriacus* is an annual herb and *Convolvulus fatmensis* may sometimes be so. *Convolvulus pseudoscammonia* is an erect herb. In a few species leaves are distinctly dimorphic with lower leaves very different in form from those on the upper part of the stem. *Convolvulus althaeoides*, *Convolvulus pitardii* and its allies, *Convolvulus palaestinus* and to some extent *Convolvulus galaticus* show this characteristic. Flower colour is quite variable but white, pinkish or pink are the norm. *Convolvulus scammonia*, *Convolvulus pseudoscammonia*, *Convolvulus cassius* and *Convolvulus palaestinus* are yellow or yellowish. As far as is known all these species have sessile glands on the dilated part of the filaments towards the base. These are not always easy to see except with a good microscope. Seeds are always glabrous, usually somewhat tuberculate but occasionally smooth.

#### 1. Convolvulus scammonia

Taxon classificationPlantaeSolanalesConvolvulaceae

L., Sp. Pl. 1: 153. 1753. (Linnaeus 1753: 153).

Figure 3, t. 1–9.

Convolvulus elongatus Salisb., Prodr. Stirp. Chap. Allerton 123. 1796; illegitimate superfluous name for *Convolvulus scammonia* L. (Salisbury 1896: 123). Type. SYRIA, Aleppo [Haleb], *P. Russell* (whereabouts unknown).

##### Type.

Plate “*Convolvulus syriacus* s. Scammoniaca syriaca” in Morison (1680: 2, sect. 1 plate 3, f. 5), lectotype (designated by Staples and Jarvis 2006: 1021).

##### Description.

Glabrous perennial herb with trailing or twining stems up to 2 m long. Leaves petiolate, 2.5–7 × 1.5–5 cm, deltoid, acute to acuminate, margin entire, base cordate but not cuneate onto the petiole, auriculate with auricles weakly 2 (– 3)-lobed, with one lobe larger than the other; petioles 0.5–4.5 cm long. Flowers 1–5 in pedunculate axillary cymes; peduncles 3.5–16 cm long; bracteoles 3–5 × 0.5–1 mm, linear to linear-lanceolate, acute; pedicels 8–11 mm, so inflorescence rather dense; outer sepals 6–7 × 5 mm, broadly oblong-obovate to rectangular, truncate and minutely mucronate, glabrous, scarious; inner sepals 7–11 × 4.5–6 mm; corolla 3–4 cm, pale yellow, undulate, midpetaline bands glabrous except for a few hairs near the apex; filaments with sessile glands below; ovary glabrous; style glabrous, divided 11–15 mm above the base, stigmas 3 mm. Capsule glabrous; seeds smooth (especially in Iraq) to tuberculate. [Sa’ad 1967: 241; Feinbrun-Dothan 1978: plate 69; Tohmé and Tohmé 2007: 216 (photo); Strid and Strid 2009: 386–387 (plate)]

**Figure 3.**
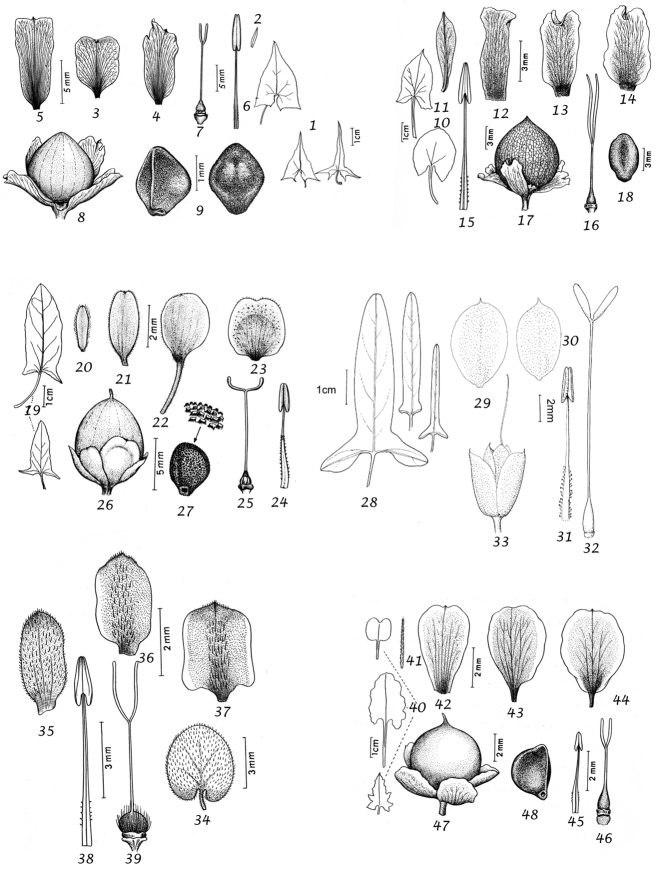
**1–9** *Convolvulus scammonia* **1** leaves **2** bracteole **3** outer sepal **4** middle sepal **5** inner sepal **6** stamen **7** ovary and style **8** capsule **9** seed. **1** from *Bourgeau* 114 (W) **2–7** from *Gathorne-Hardy* 547 (E) **8–9** from *Rechinger* 10708 (W) **10–18** *Convolvulus durandoi* **10** leaves **11** bracteole **12** outer sepal **13** middle sepal **14** inner sepal **15** stamen **16** ovary and style **17** capsule **18** seed **10 & 17–18** from *Battandier & Trabut* 9 (GOET) **11–16** from *Gay* 2792 (W) **19–27** *Convolvulus arvensis* **19** leaves **20** bracteole **21** outer sepal **22** middle sepal attached to pedicel **23** inner sepal **24** stamen **25** ovary and style **26** capsule, 27 seed **19–25** from *Abdallah et al.* 1725 (CAIM) **26–27** from *Abdallah et al.* 1671 (CAIM) **28–33** *Convolvulus chinensis* **28** leaves **29** outer sepal **30** inner sepal **31** stamen **32** ovary and style **33** sepals enclosing capsule **28** (centre/right) from *sin coll.* 12/6/1886 (OXF) **28** (left)–**33** from *Wang* 3259 (K) **34–39** *Convolvulus mairei* **34** leaf **35** outer sepal **36** middle sepal **37** inner sepal **38** stamen **39** ovary and style. From *Maire & Petitmengin* 628 (K) **40–48** *Convolvulus fatmensis* **40** leaves **41** bracteole **42** outer sepal **43** middle sepal **44** inner sepal **45** stamen **46** ovary and style **47** capsule **48** seed **40–46** from *Schimper* 839 (L) **47–48** from *Shalaby & Sharobiem* 1637 (CAIM).

##### Distribution.

East Mediterranean region to Crimea and Iraq: Greece (Aegean Islands only): Rhodes (*Rechinger* 7441), Chios (*Platt* 238); Turkey (*Balansa* 697, *Davis & Coode* 36447, *Sintenis* 1274); Crimea (*Rehmann* 606, *Callier* 323); Iraq (*Rawi* 23102, *Gillett* 8236, *Wheeler-Haines* s.n. [4/6/1960]); Syria (*Haradjian* 1508, 2711); Lebanon (*Breidy et al*. LEB-555); Palestine/Israel (*Davis* 4630); Jordan (*Täckholm et al.* 8934); Egypt: Sinai (fide Boulos 2000: 251).

##### Notes.

A very distinct, nearly completely glabrous species with a yellow corolla, acutely-angled deltoid leaves and the outer sepals much smaller than the inner sepals. Molecular studies (Williams et al. 2014) show this species and *Convolvulus pseudoscammonia* to be the most closely related species to *Calystegia* spp.

#### 2. Convolvulus pseudoscammonia

Taxon classificationPlantaeSolanalesConvolvulaceae

K.Koch, Linnaea 22: 746. 1849. (Koch 1849: 746).

Convolvulus scammonia var. pseudoscammonia (K.Koch) Sa’ad, Meded. Bot. Mus. Herb. Rijks Univ. Utrecht 281: 242. 1967. (Sa’ad 1967: 242). Type. Based on *Convolvulus pseudoscammonia* K.Koch
Convolvulus cappadocicus Hausskn. & Sint. ex Woronow, Vĕstn. Tiflissk. Bot. Sada (Monit. Jard. Bot. Tiflis) 10: 31. 1908. (Woronow 1908: 31). Type. TURKEY, Egin, *Sintenis* 2864 (lectotype TGM, designated by Sa’ad 1967: 242); isolectotypes B, K!, STU, W!).

##### Type.

TURKEY, Gaue Sber, *Koch* s.n. (holotype B†).

##### Description.

Perennial herb with tap root and stems somewhat woody below, similar in all details to *Convolvulus scammonia* but stems erect to 60 cm, leaves 1.5–5 × 0.3–0.8 cm, sagittate, the central lobe narrowly oblong-lanceolate, basal auricles small, simple. The peduncles appear always to bear only 1–2 flowers and the bracteoles are filiform, not more than 0.5 mm wide. [Sa’ad 1967: 242; Parris 1978: 218]

##### Distribution.

Northeast Turkey (*Sintenis* 1335, *Stainton & Henderson* 5763, *Davis & Hedge* 30166, *Woronov* 271, *Turkevicz* 603, *Herrero* 1361); Armenia (?).

##### Notes.

Molecular studies (Williams et al. 2014) confirm that this is a distinct species related to but distinct from *Convolvulus scamonia*.

#### 3. Convolvulus durandoi

Taxon classificationPlantaeSolanalesConvolvulaceae

Pomel, Nouv. Mat. Fl. Atl. 85. 1874. (Pomel 1874: 85).

Figure 3, t. 10–18

##### Type.

ALGERIA, *Durando* s.n. (holotype MPU004911!, possibly divided with AL; isotype MPU004912, P00417697!).

##### Description.

Glabrous, trailing perennial herb, the stems angular, reaching at least 75 cm. Leaves petiolate, 1.2–3 × 1–2.2 cm. ovate, apex acute to more or less rounded and mucronate, margin entire, base truncate (below) or cordate (above), the auricles small, triangular-acute, venation reticulate; petioles 1–2.5 cm. Flowers axillary, pedunculate, solitary; peduncles 3–9 cm, commonly flexuose in bud; bracteoles 3–9 × 0.25–0.5 mm, linear-oblanceolate; pedicels 0.5–3.5 cm; outer sepals 4–5 × 2.5–3 mm, spathulate, the apex abruptly widened above the oblong base, rounded, sparsely ciliate, commonly reflexed; inner sepals 5–6 × 2.5–3 mm, similar in shape but apex emarginate and not reflexed; corolla 1.7–2.3 cm long, pink, weakly lobed, midpetaline bands glabrous; filaments glandular below; ovary conical, glabrous; style glabrous, divided 5–6 mm above the base, stigmas 5–6 mm. Capsule glabrous, style persistent; seeds slightly rugose. [Sa’ad 1967: 224]

##### Distribution.

Restricted to the Magreb of northwestern Africa: Algeria (*Maire* 5944, *Gay* 2792, *Battandier* 3823); Tunisia (*Simpson* 38395); Morocco (?).

##### Notes.

A very distinctive species because of its reflexed spathulate sepals and unusual ovate, truncate, reticulate-veined leaves.

#### 4. Convolvulus arvensis

Taxon classificationPlantaeSolanalesConvolvulaceae

L., Sp. Pl. 1: 153. 1753. (Linnaeus 1753: 153).

Figure 3, t. 19–27

Convolvulus hastatus Forssk., Fl. Aegypt.-Arab. 203. 1775. (Forsskål 1775: 203). Type. EGYPT, Cairo, *Forssk* å*l* s.n. (syntype C10002044).
Convolvulus minor Gilib. Fl. Lit. Inch. 1: 43. 1782. (Gilibert 1782: 43). Type. Not specified.
Convolvulus auriculatus Desr., Encycl. [Lamarck et al.] 3: 540. 1792. (Desrousseaux 1792: 540). Type. MAURITIUS (“Isle de France”), *Commerson* s.n (holotype P00608776).
Convolvulus prostratus F.W.Schmidt, Fl. Boëm. Cent. 2: 93. 1793 [pub. 1794], nom illeg., non *Convolvulus prostratus* Forssk. (1775). (Schmidt 1793: 93). Type. Icon 237 linked to Schmidt (1793) in Prague University Library.
Convolvulus sagittifolius Salisb., Prodr. Stirp. Chap. Allerton 123. 1796, superfluous name for *Convolvulus arvensis* L. (Salisbury 1796: 123).
Convolvulus hastifolius Poir., Encycl. [Lamarck et al.] Suppl. 3: 467 (1814), lapsus [spelling mistake] for *Convolvulus hastatus* Forssk. (Poiret 1814: 467).
Convolvulus corsicus Roem. & Schult., Syst. Veg, ed. 15 bis [Roemer & Schultes] 4: 256. 1819. (Roemer and Schultes 1819: 256). Type: CORSICA, no type cited.
Convolvulus cherleri C.Agardh ex Roem. & Schult., Syst. Veg, ed. 15 bis [Roemer & Schultes] 4: 261. 1819. (Roemer and Schultes 1819: 261). Type. SPAIN, Malaga, *C. Agardh* (whereabouts unknown).
Convolvulus malcolmii Roxb., Fl. Ind. (Roxburgh) 2: 55.1824. (Roxburgh 1824: 55). Type. a plant cultivated in Kolkata from seed brought from Iran by Malcolm (lectotype, Icon. 1532 (K) accompanying Flora Indica, designated here).
Convolvulus arvensis var. pumilus Choisy, Prodr. [A.P. de Candolle] 9: 407. 1845. (Choisy 1845: 407). Type. GERMANY, Thuringia, *Wallroth* s.n. (P, not seen).
Convolvulus arvensis var. obtusifolius Choisy, Prodr. [A.P. de Candolle] 9: 406. 1845. (Choisy 1845: 406). Type. Based on *Convolvulus corsicus* Roem. & Schult.
Convolvulus arvensis var. biflorus Choisy, Prodr. [A.P. de Candolle] 9: 406. 1845. (Choisy 1845: 406). Type. EGYPT, Cairo, *Forsskål* s.n. (C10002044, lectotype designated here).
Convolvulus arvensis var. multiflorus Choisy, Prodr. [A.P. de Candolle] 9: 407. 1845. (Choisy 1845: 407). Type. LEBANON, Mount Lebanon, *Mergon* s.n. (lectotype G-DC, designated by Sa’ad 1967: 218).
Convolvulus arvensis var. linearifolius Choisy, Prodr. [A.P. de Candolle] 9: 407. 1845. (Choisy 1845: 407). Type. GERMANY, Thuringia, *Wallroth* s.n. (location unknown).
Convolvulus arvensis var. auriculatus (Desr.) Choisy, Prodr. [A.P. de Candolle] 9: 407. 1847. (Choisy 1847: 407). Type. Based on *Convolvulus auriculatus* Desr.
Convolvulus arvensis var. villosus Choisy. Prodr. [A.P. de Candolle] 9: 407. 1845. (Choisy 1845: 407). Type. CHILE, *J. Style* s.n. (holotype G-DC, not seen).
Convolvulus arvensis var. minor Lindem, Bull. Soc. Imp. Naturalistes Moscou 23: 508. 1850. (Lindemann 1850: 508). Type. BELARUS, Hainowka, *Lindem* s.n. with annotation *Convolvulus quinquelobus* (whereabouts uncertain, ?LE).
Convolvulus arvensis var. hastulatus Meisn., Fl. Bras. (Martius). 7: 313. 1869. (Meisner 1869: 313). Type: Southern Brazil, *Sello*; Uruguay and Argentina, *Tweedie*: Chile, *Maximowicz* (all syntypes, whereabouts unknown ?B†).
Convolvulus arvensis var. aphacoefolius Pomel, Nouv. Mat. Fl. Atl. 1: 85. 1874. (Pomel 1874: 85). Type. ALGERIA, Garrouban, *Pomel* s.n. (isotype MPU005194!).
Convolvulus arvensis var. filicaulis Pomel, Nouv. Mat. Fl. Atl. 1: 85. (Pomel 1874: 85). Type. ALGERIA, Sidi-Bouzid, Djebel-amour, *Pomel* s.n. (isotype MPU005193!).
Convolvulus segobricensis Pau, Not. Bot. Fl. Espan, 1: 7. 1887. (Pau 1887: 7). Type. SPAIN, Valencia, *Pau* s.n. (holotype MA?, not seen).
Convolvulus arvensis var. cherleri (C.Agardh ex Roem. & Schult.) Halácsy, Consp. Fl. Graec. 2: 307. 1902. (Halácsy 1902: 307). Type. Based on *Convolvulus cherleri* C.Agardh ex Roem. & Schult.
Convolvulus ambigens House, Bull. Torrey Bot. Club 32: 139. 1905. (House 1905: 139). Type. UNITED STATES OF AMERICA, Colorado, *C.S.Crandall* 4218 (holotype NY!; isotype US).
Convolvulus europaeus Barb.-Gamp., Bull. Soc. Bot. Genève, ser. 2, 12: 236. 1921 [“1920”]. (Barbey-Gampert 1921: 236). Type. SPAIN, Picos de Europa, *Barbey-Gampert* s.n. (holotype G, not seen).
Convolvulus incanus var. glabratus Farw., Pap. Michigan Acad. Sci. 2: 36. 1923. (Farwell 1923: 36).
Convolvulus arvensis Type. UNITED STATES OF AMERICA, Michigan, Detroit, *Farwell* 5950 (isotypes GH00112744, BLH0000114).
Convolvulus arvensis var. paui Maire, Bull. Soc. Hist. Nat. Afrique N. 28: 370. 1937. (Maire 1937: 370). Type. MOROCCO, *P. Font Quer* (holotype MPU006711!).
Convolvulus arvensis var. trigonophyllus Maire, Bull. Soc. Hist. Nat. Afrique N. 28: 370. 1937. (Maire 1937: 370). Type. MOROCCO, Mont Amezdour, *E.K. Balls* 2740 (holotype MPU003790!)
Convolvulus arvensis subsp. crispatus Franco, Nova Fl. Portugal 2: 565. 1984. (Franco 1984: 98). Type. PORTUGAL, Serpa, Herdade da Loja, *F.Goinhas Palma* (holotype LISI, not seen).

##### Type.

“Europe” (lectotype LINN 218.1!. designated by Meeuse 1958: 695).

##### Description.

Perennial herb from an extensive creeping underground rootstock, branched at base with trailing or twining quadrangular stems to about 75 cm long, plant glabrous to sparsely hairy. Leaves petiolate, 1–7 × 0.5–4 cm, broadly to narrowly ovate-deltoid, obtuse or acute, mucronulate, margin entire or undulate, base hastate to sagittate with simple auricles; petioles 1–2.5 cm. Flowers 1–3 in axillary pedunculate cymes; peduncles 1–5 cm; bracteoles 2.5–3 mm, filiform; pedicels 0.6–20 mm; sepals 3.5–4.5 × 2.5–3.5 mm, obovate to oblong, obtuse to mucronulate, scarious-margined; corolla 1.5–2.5 cm long, white or pink, undulate but not lobed, midpetaline bands often dark pink, pubescent; filaments glandular below; ovary glabrous, style glabrous, divided 7–8 mm above base, stigmas 2.5 mm. Capsule glabrous; seeds tuberculate. [Sa’ad 1967: 214; Feinbrun-Dothan 1978: plate 65; Collenette 1999: 226 (photo); Tohmé and Tohmé 2007: 213 (photo); Silvestre 2012: 153; Sell and Murrell 2009: 343–344; Austin and Ghazanfar 1979: 28; Siddiqui 1977: 7 (Figure 2); Breckle and Rafiqpoor 2010: 41 (photo); Pignatti 1982: 389]

##### Distribution.

A very common cosmopolitan weed of all temperate regions which also grows in upland regions throughout the tropics.

##### Notes.

A very variable species especially in indumentum, leaf shape and flower colour, of which many forms and varieties have been described (Choisy 1845: 406–407, Sa’ad 1967: 215–219; Franco 1984: 98, Sell and Murrell 2009: 343–344, for example). *Convolvulus arvensis* is usually easily recognised by the short sepals, which rarely exceed 4.5 mm, combined with a corolla about five times longer than the calyx. The leaves are usually, but not always, glabrous or nearly so and the auricles are unlobed.

#### 5. Convolvulus chinensis

Taxon classificationPlantaeSolanalesConvolvulaceae

Ker-Gawl., Bot. Reg. 4: t. 322. 1818. (Ker-Gawler 1818: t 322).

Figure 3, t. 28–33

##### Type.

CHINA, cultivated plant grown from seed collected by Staunton at “Pechelee” (holotype BM001053866!).

##### Description.

Perennial herb with long decumbent stems from a central rootstock to at least 50 cm, glabrous or, on older parts, minutely scabridulous. Leaves petiolate, 3–5 cm long, formed of an oblong, acute, entire central lobe 2–4 mm wide, a broadly cuneate base and horizontally to weakly reflexed auricles, these mostly bifid with acute segments; petioles 4–7 mm. Flowers axillary, pedunculate, solitary; peduncles 3.2–4.5 cm, slighty flexuous; bracteoles 3 mm, linear-filiform; pedicels 4–8 mm; sepals 6–7 × 3.5–4 mm, obovate, obtuse and sometimes mucronate, glabrous, margins scarious, inner sepals slightly larger; corolla 2–2.8 cm long, pink, very shallowly lobed, the midpetaline bands extended as short teeth, nearly glabrous but with a few hairs near apex; filaments glandular below; ovary glabrous; style glabrous, divided 12–14 mm above base, stigmas 2.5–3.5 mm. Capsule glabrous, seeds glabrous, minutely tuberculate.

##### Notes.

We recognise two subspecies:

#### 5a. Convolvulus chinensis subsp. chinensis

Taxon classificationPlantaeSolanalesConvolvulaceae

Convolvulus bicuspidatus Fisch. ex Link, Enum. Hort. Berol. 1: 201. 1821. (Link 1821: 201). Type. RUSSIA, Siberia, “Dahurica”, *Fischer* s.n. (B†).
Convolvulus arvensis var. sagittatus Ledeb., Fl. Altaic. [Ledebour] 1: 225. 1829. (Ledebour 1829: 225). Type. RUSSIA, based partially on Fischer specimen (?LE).cited in Cat. Hort. Gorenk. 28. (Fischer 1808).
Convolvulus arvensis var. crassifolius Choisy, Prodr. [A.P. de Candolle] 9: 406. 1845. (Choisy 1845: 406). Type. MONGOLIA, *Meyer & Turczaninov* (lectotype G-DC, designated by Sa’ad 1967: 218).
Convolvulus sagittifolius Fisch. ex T.Liou & Y.Ling, Fl. Ill. Nord Chine 1: 17. 1931, nom. illeg., non *Convolvulus sagittifolius* Michx. (1803). (Liou and Ling 1931: 17). Type. Based on Fischer specimen (?LE); cited in Cat. Hort. Gorenk. 28. (Fischer 1808).
Convolvolus fischerianus Petrov, Byull. Moskovsk. Obshch. Isp. Prir., Otd. Biol., n.s., 44: 147. 1935. (Petrov 1935: 147). Type. not specified, possibly *Fischer* s.n. (LE, not seen).

##### Distinguishing features.

Distinguished by the decumbent habit and distinctive strap-shaped leaves, the central lobe elongated.

##### Distribution.

Very common in northern China, Mongolia and Siberia becoming rare in Kazakhstan, where it is largely replaced by *Convolvulus arvensis*. Russia: Siberia (*Elias, Shetler & Murray* 7606, *Turkewitsch* 1043, *Kuznezow* 2709, *Timokhina & Danilyuk* 1249, *Shiskin* 332, *Castroviejo* 14317); Mongolia (*Pobedimova* 1323, *Campbell* 1901); Northern China (*David* 1851, *Chaffanjon* 1662, *Rock* 14359, *Ho et al*. 120, *R. C. Ching* 171, *Williams* 10515, *Licent* 120, *Petrov* s.n. [25/6/1957]); Kazakhstan (*Tsvelev et al.* 777).

#### 5b. Convolvulus chinensis subsp. triangularis

Taxon classificationPlantaeSolanalesConvolvulaceae

J.R.I.Wood & R.W.Scotland subsp. nov.

urn:lsid:ipni.org:names:77147664-1

##### Diagnosis.

A subsp. typo habitu suberecta et foliis triangularibus.

Convolvulus arvensis var. erectus Ledeb., Fl. Altaic. [Ledebour] 1: 224. 1829. (Ledebour 1829: 225).

**Type.** RUSSIA, Altai, Tiuguriuk stream by Katunja River (LE, not seen).

##### Type.

KAZAKHSTAN, “in rupestribus montium Tarbagatai ad torrentium Dschanybek”, *Karelin & Kiriloff* 328 (holotype LE ex Herb Ledebour!; isotypes BM001035796!, LE ex herb. Fischer!, LE ex herb. Schrenk!, P!).

##### Distinguishing features.

Distinguished by its suberect habit and triangular leaves, c. 3–5 × 1.5–4 cm.

##### Distribution.

Russia: Siberia (*Salefov* 794, 806 (sin data), *Mardovkin* s.n. (“Siberia altaica”), Kazakhstan (*Karelin & Kiriloff* 597 (“Tarbagatai ad torrentium Dschanybek”), *Roldugin* s.n. [12/8/1960] “Dzungarsky Alatai”), *Schrenk* s.n. (“Songaria”). Apparently rare.

##### Notes.

*Convolvulus chinensis* is most reliably distinguished from *Convolvulus arvensis* by the longer sepals. Additionally the auricles are often bifid, the central lobe oblong and the corolla usually deep pink and slightly larger than in *Convolvulus arvensis*. It is often considered to be a form of *Convolvulus arvensis* but intermediates are uncommon, mainly being found in the Tibet region, and could be of hybrid origin. Molecular studies (Williams et al. 2014) strongly support the recognition of *Convolvulus chinensis* as a distinct species.

#### 6. Convolvulus mairei

Taxon classificationPlantaeSolanalesConvolvulaceae

Halácsy, Bull. Soc. Sci., Nancy, sér. 3, 8: 176. 1908. (Maire and Petitmengin 1908: 176).

Figure 3, t. 34–39.

##### Type.

GREECE, Parnassus, Lake Zouvala, *R.Maire* 113 (holotype ?AL, not seen.).

##### Description.

Trailing perennial herb with very slender stems 10–30 cm long, vegetative parts densely pubescent. Leaves petiolate, 0.5–1.3 × 0.3–1 cm, suborbicular to ovate with obtuse apex to deltoid with acute apex, margin undulate, base cordate to hastate; petioles 2–9 mm. Flowers solitary, pedunculate, axillary; peduncles 2–11 mm, strongly recurved in fruit; bracteoles 1–1.5 mm, linear; pedicels 2.5–6 mm; outer sepals 2–3 × 1.5–2 mm, oblong-elliptic, somewhat truncate at both ends, pubescent, margins scarious. Corolla 0.8–1 cm long, pink, unlobed, midpetaline bands pubescent; filaments glandular; ovary pilose; style glabrous, persistent, divided 3–3.5 mm above base, stigmas c. 1.5 mm. Capsule borne on a recurved peduncle, pilose; seeds glabrous, obscurely rugose. [Sa’ad 1967: 235]

##### Distribution.

Greece (*Maire & Petitmengin* 668, *Atchley* 2314, *Guiol* 2411). Apparently rare and very localised to the area around Mount Parnassus near Delphi in central Greece.

##### Notes.

A distinctive species, superficially resembling a diminutive *Convolvulus arvensis*, with leaves and flower parts all very small. The plant is pubescent in its vegetative parts with a hirsute recurved capsule and a proportionally very small corolla.

#### 7. Convolvulus fatmensis

Taxon classificationPlantaeSolanalesConvolvulaceae

Kunze, Flora 23(1): 172. 1840. (Kunze 1840: 172).

Figure 3, t. 40–48.

Convolvulus amblycalyx Steud., Nomencl. Bot., ed.2 1: 407. 1840, illegitimate superfluous name for *Convolvulus fatmensis* Kunze (Steudel 1840: 407).

##### Type.

SAUDI ARABIA, Wadi Fatma, *G. W.Schimper* 839 (lectotype LZ, designated by Sa’ad 1967: 226); isolectotypes GOET, HBG, JE, L, LE!, OXF!, P!, W!).

##### Description.

Perennial (possibly sometimes annual) herb with trailing stems to at least 50 cm from a slender central tap root; stems glabrescent to pubescent. Leaves petiolate, 1.2–4.5 × 0.6–4 cm, ovate-deltoid, apex obtuse, margin sinuate, base auriculate and cordate; petioles 0.5–3.5 cm. Flowers 1(-3) borne on axillary peduncles; peduncles 7–30 mm, commonly recurved in fruit; bracteoles 2 mm, filiform; pedicels 3–5 mm; outer sepals 3–5 × 3–4 mm, obovate, rounded, glabrous, slightly concave; inner sepals slightly narrower, 2.5–3 mm wide; corolla 0.9–1.3 cm long, pink, distinctly lobed, midpetaline bands brownish, thinly pubescent; filaments glandular below; ovary glabrous; style glabrous, divided c. 2 mm above base, stigmas 1 mm. Capsule glabrous, strongly exserted from the sepals, recurved in fruit; seeds glabrous, smooth (not rugulose as stated by Sa’ad,1967: 226). [Sa’ad 1967: 226; Feinbrun-Dothan 1978 (plate 67); Collenette 1999: 229 (photo)]

##### Distribution.

A widespread Sahara-Sindian species, generally uncommon and very scattered in occurrence but most frequent in Egypt; usually a weed of sandy fields. “Mauretania” (*Chudeau* s.n. [10/2/1911]); Morocco (*Maire* 781); Algeria: Ahaggar (*Maire* 857); Tunisia (*Cosson* s.n. [22/5/1858]); Libya (*Guichard* KG/LIB/121); Egypt (*Abd El Ghani* 5994, *Kralik* 168); Sudan (*El Din* 1, *Colston* 257); Saudi Arabia (*Collenette* 1753, 7903; *Fischer* 20, *Mandaville* 2884); Yemen (*Wood* 2059); Oman (*Radcliffe-Smith* 4133); Palestine/Israel (fide Feinbrun-Dothan 1978: 42); Lebanon (?); Iran (*Popov* 51/11).

##### Notes.

Very distinct species with sinuate leaves and pink, lobed corolla borne on a recurved peduncle. The leaves are sometimes exceptionally small.

#### 8. Convolvulus steppicola

Taxon classificationPlantaeSolanalesConvolvulaceae

Hand.-Mazz., Symb. Sin. 7: 810. 1936. (Handel-Mazzetti 1936: 810).

##### Type.

CHINA, Yunnan, Dali, *Handel-Mazzetti* 6351 (holotype W!; isotype E00067083!).

##### Description.

Pubescent perennial herb with (probably) decumbent stems from a thickened rootstock, young growth brownish-tomentose; stems to 60 cm, probably reaching 1 m, Leaves shortly petiolate, 1.1–3.5 cm long, the central lobe 0.2–1 cm wide, linear or oblong, acute, margin entire, undulate, sinuate or more or less dentate, base hastate, the auricles simple or bifid, sometimes intergrading with sinuate leaf margin. Flowers 1–2, axillary, pedunculate; peduncles 1.5–4 cm; bracteoles 3–4 × 0.5 mm, linear or filiform; pedicels 6–15 mm long, straight to slightly bent; outer sepals 7–10 × 4–5 mm, ovate, acuminate, villous with ciliate margins; inner sepals similar but much less hairy; corolla 1.2–1.4 cm long, pink or white, unlobed, midpetaline bands pilose, extended as short teeth; ovary and style glabrous. Capsule glabrous; seeds nearly smooth, glabrous.

##### Distribution.

Endemic to SW China: Yunnan (*Ducloux* 6660, *E. Maire* 511, 581, *Delavay* s.n. [8/4/1884]), 1600–2450 m.

##### Notes.

Apparently rare and localised and no recent collections seen.

#### 9. Convolvulus sinuatodentatus

Taxon classificationPlantaeSolanalesConvolvulaceae

Collett & Hemsl., J. Linn. Soc. Bot. 28: 98. 1890. (Collett and Hemsley 1890: 98).

##### Type.

MYANMAR/BURMA, Shan plateau, *Collett* 464 (holotype K!; isotype CAL?).

##### Description.

Coarsely pilose perennial herb with decumbent stems from a thickened taproot; stems to 20 cm but probably much more. Leaves petiolate, 1–1.5 × 0.2–0.5 cm, ovate-deltoid, acute, margin sinuate-dentate, base truncate to subcordate, coarsely pilose; petioles 4–6 mm. Flowers axillary, solitary, pedunculate; peduncles 1–1.5 cm; bracteoles 1–2 mm, filiform; pedicels 4–7 mm; outer sepals 6–7 × 2–3 mm, oblong-oblanceolate, acute, pilose on dorsal surface; inner sepals similar but 5 × 3 mm, obovate, scarious-margined; corolla c. 1.3 cm long, colour unknown, apparently weakly lobed, midpetaline bands pilose; ovary glabrous; style glabrous, divided c. 2.5 mm above base; stigmas 2 mm. Capsule not known.

##### Distribution.

Myanmar (Burma). Only known from the type collection found at c. 1700 m.

##### Notes.

This poorly known species might prove to be a variant of *Convolvulus steppicola* but further collections are needed before its status can be asssessed.

#### 10. Convolvulus rufescens

Taxon classificationPlantaeSolanalesConvolvulaceae

Choisy, Mém. Soc. Phys. Genève 6: 477. 1834. (Choisy 1834: 479).

Convolvulus flavus sensu C.B.Clarke (1883: 219) et auct. mult.

##### Type.

INDIA, Tamil Nadu/Kerala, Nilgiri Hills, *J.P. Leschenault* s.n. (lectotype P03548937!, designated here).

##### Description.

Perennial scrambling and climbing herb to at least 50 cm, stems pubescent, the hairs reddish on young parts. Leaves petiolate, 2–8 × 2–6 cm, lanceolate to broadly ovate-deltoid, acute and mucronulate, margin variable, undulate to deeply dentate, base broadly cordate in outline but cuneate onto the petiole, auricles entire to deeply dentate, pubescent on both surfaces, especially on the veins beneath; petioles 1.5–3 cm. Flowers 1–2 (-3) in pedunculate, axillary cymes; peduncles often paired, 6–8 mm; bracteoles c. 1.25 mm, caducous, ovate, acuminate; pedicels 8–10 mm, more densely pubescent than peduncles; sepals 6–7 × 3–4 mm, outer sepals obovate-elliptic, abruptly narrowed at apex, apiculate, pubescent, inner sepals similar, obovate, mucronate, scarius-margined, subglabrous; corolla 10–12 mm, whire or cream, deeply lobed, mid-petaline bands terminating in a tuft of hairs; filaments glabrous; ovary glabrous, style glabrous, divided c. 5 mm above base, stigmas 1.5–2 mm, linear. Capsule glabrous, seeds glabrous.

##### Distribution.

Endemic to South India: Nilgiri and Palni (Pulney) Hills (*Wight* 1992, *Perottet* 892, *Clarke* 10758).

##### Notes.

Like the two preceeding species, this is a geographically isolated species. Although quite variable, the leaves are often strongly dentate and the auricles lobed. The corolla is similar to that of the South American species *Convolvulus crenatifolius* and *Convolvulus hermanniae* as well as to that of *Convolvulus sinuatodentatus* from Myanmar. The peduncles are unusual as they are commonly paired. We have not seen recent collections.

#### 11. Convolvulus betonicifolius

Taxon classificationPlantaeSolanalesConvolvulaceae

Mill., Gard. Dict. ed. 8: no. 20. 1768. (Miller 1768: 20).

Figure 4, t. 1–9.

Convolvulus pubescens Sol., in Russell, Aleppo, ed. 2, 2: 246 1794, illegitimate superfluous name for *Convolvulus betonicifolius* Mill. (Russell 1794: 246). Type. SYRIA, Aleppo, *Russell s. n.* (holotype BM001014565!).
Convolvulus lanuginosus Vahl, Symb. Bot. 3: 23. 1794., nom. illeg., non *Convolvulus lanuginosus* Desr. (1792). (Vahl 1794: 23). Type. sin data (holotype C10009605!).
Convolvulus tomentosus Choisy, Prodr. [A.P. de Candolle] 9: 413. 1845., nom. illeg., non *Convolvulus tomentosus* Vell. (1829). (Choisy 1845: 413). Type. Based on *Convolvulus lanuginosus* Vahl
Convolvulus sagittifolius Sm. Fl. Graec. Prod. 1: 133. 1806, nom. illeg., non *Convolvulus sagittifolius* Michx. (1803). (Sibthorp and Smith 1806: 133). Type. Icon., Fl. Graec. 2: 77, t. 193 (1816).
Convolvulus hirsutus M.Bieb., Fl. Taur.-Caucas 1: 422. 1808. (Marschall von Bieberstein 1808: 422). Type. CRIMEA, *Steven* s.n. (holotype LE!).
Convolvulus atriplicifolius Poir., Encycl. (Lamarck), Suppl. 3 (2): 467.1814. (Poiret 1814: 467). Type. SYRIA, *de Labillardière s.n.* (holotype FI).
Convolvulus sibthorpii Roem. & Schult., Syst. Veg, ed. 15 bis [Roemer & Schultes] 4: 285. 1819. (Roemer and Schultes 1819: 285). Type. GREECE, Samos, *sin col*. (whereabouts unknown).
Convolvulus amoenus K. Koch. Linnaea 19: 19. 1847, nom. illeg., non *Convolvulus amoenus*Dietrich (1816). (Koch 1847: 19). Type. TURKEY, Pontus Euxinus, *Thirke s. n.* (holotype B†, possible isotype MO).
Convolvulus peduncularis Boiss., Diagn. Pl. Orient. 11: 84. 1849. (Boissier 1849: 84). Type. TURKEY, between Orfa and Sierek, *Kotschy* 58 (holotype G; isotype K000852030).
Convolvulus pedunculatus Walp., Ann. Bot. Syst. 3(1): 112. 1852, lapsus [spelling mistake] for *Convolvulus peduncularis* Boiss. (Walpers 1852: 112).
Convolvulus hirsutus var. tomentosus Boiss., Fl. Orient. [Boissier] 4: 105. 1875. (Boissier 1875b: 105). Type. Based on *Convolvulus peduncularis* Boiss.
Convolvulus betonicifolius subsp. peduncularis (Boiss.) Parris, Fl. Turkey & E. Aegean Is (P.H.Davis). 6: 217. 1978. (Parris 1978: 217). Type. Based on *Convolvulus peduncularis* Boiss.
Convolvulus hirsutus var. virescens Boiss., Fl. Orient. [Boissier] 4: 105. 1875. (Boissier 1875b: 105). Type. TURKEY, Egirdir, *Heldreich s. n.* (G, E00285435, WAG0003915, K!).
Convolvulus armenus Boiss. & Kotschy ex Boiss., Fl. Orient. [Boissier] 4: 105. 1875. (Boissier 1875b: 105). Type. TURKEY, *Kotschy* 373 (lectotype G, designated by Sa’ad 1967: 221); isolectotypes K000852028, P00608770!, W!).
Convolvulus betonicifolius var. armenus (Boiss. & Kotschy ex Boiss) Sa’ad, Meded. Bot. Mus.Herb. Rijks Univ. Utrecht 281: 221. 1967. (Sa’ad 1967: 221). Type. Based on *Convolvulus armenus* Boiss.
Convolvulus aleppensis Sa’ad, Meded. Bot. Mus.Herb. Rijks Univ. Utrecht 281: 209. 1967. (Sa’ad 1967: 209). Type. SYRIA, Aleppo, *Kotschy* 232 (holotype P!).

##### Type.

Cultivated plant grown in Chelsea Physic Garden from seed received from Paris (holotype BM001035798!).

##### Description.

Very variable trailing or twining perennial herb up to 1 m high, stems angled, vegetative parts always hirsute, thinly to densely pubescent, pilose or tomentose. Leaves petiolate, 2.5–8 × 2–6 cm, ovate, apex obtuse or acute, often mucronate, margin entire to undulate, base cordate and cuneate onto the petiole, usually auriculate, auricles rounded to acute, entire or dentate; petioles 0.5–1.5 (-6) cm. Flowers 1–3 (-8) in pedunculate, axillary cymes (often clearly dichasial); peduncles 2–14 cm, very variable from specimen to specimen; bracteoles filiform to linear or linear-oblanceolate, acute, 6–14 × 0.5–1.5 mm, pedicels 5 –15 mm; outer sepals 7–15 × 3–5 mm, oblong-elliptic, acute or acuminate, bicoloured, sometimes slightly constricted below triangular, slightly deflexed dark green apical portion, inner sepals scarious-margined 8–10 × 5–6 mm, shorter but broader; corolla 2.8–3.6 cm, white, cream, or pink, unlobed, midpetaline bands pilose, sometimes darker coloured; filaments glandular below; ovary pilose, style pilose, divided c. 9 mm above base, stigmas 3 mm. Capsule pilose; seeds papillose. [Sa’ad 1967: 219; Feinbrun-Dothan 1978: plate 66; Tohmé and Tohmé 2007: 214 (photo); Silvestre 2012: 258; Strid and Strid 2009: 388–389 (plate); Pignatti 1982: 389; Grigoriev 1953: 12 (plate)]

**Figure 4.**
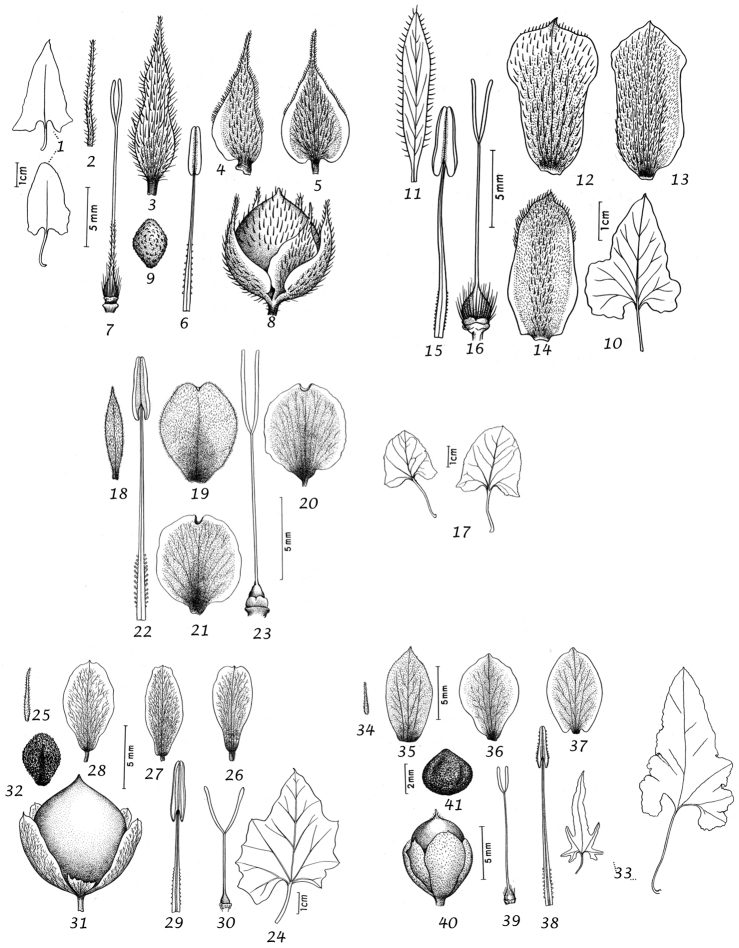
**1–9** *Convolvulus betonicifolius* **1** leaves **2** bracteole **3** outer sepal **4** middle sepal **5** inner sepal **6** stamen **7** ovary and style **8** capsule **9** seed. From *Stribrny* s.n. (G) **10–16** *Convolvulus cassius* **10** leaf **11** bracteole **12** outer sepal **13** middle sepal **14** inner sepal **15** stamen **16** ovary and style. From *Dinsmore* 10127 (K) **17–23** *Convolvulus longipedicellatus* **17** leaves **18** bracteole **19** outer sepal **20** middle sepal **21** inner sepal **22** stamen **23** ovary and style. From *Manisadjan* s.n. (W) **24–32** *Convolvulus stachydifolius* **24** leaf **25** bracteole **26** outer sepal **27** middle sepal **28** inner sepal **29** stamen **30** ovary and style **31** capsule **32** seed **24–30** from *Bornműller* 1528b (B) **31–32** from *sin coll.* (JE) **33–41** *Convolvulus palaestinus* **33** leaves **34** bracteole **35** outer sepal **36** middle sepal **37** inner sepal **38** stamen **39** ovary and style **40** capsule **41** seed **33–39** from *Dinsmore* 1409 (E) **40–41** from *sin coll*. (JE).

##### Distribution.

Widely distributed from the eastern Mediterranean region east to the Caucasus and Iran: Greece (*Rechinger* 8992): Albania (*Alston & Sandwith* 1730): Bulgaria (*Wiesniewski* 1161); Turkey (*Davis & Polunin* 4220); Cyprus (*Meikle* 2626); Crimea; Russia: North Caucasus (*Sokolova* 1149, *Kozo-Poljansky & Preobrashensky* s.n. [5/1915]); Iraq (*Al Kaisi et al*. 51085); Syria (*Kotschy* 232); Lebanon (*Gombault* 4491); Palestine/Israel (*Post* 460, *Heller & Shamash* 13434); Iran (*Jacobs* 6837). Naturalised in Spain, France (*Gay* s.n.) and Italy (*Fiori & Beguinot* 2509).

##### Notes.

A very variable species in indumentum, leaf shape, peduncle length, number and colour of flowers and size and shape of sepals Attempts have been made by Sa’ad (1967) and Parris (1978) to provide an infraspecific classification but the characters do not correlate well with each other and it seems best to treat this as a single widespread variable species.

#### 12. Convolvulus longipedicellatus

Taxon classificationPlantaeSolanalesConvolvulaceae

Sa’ad, Meded. Bot. Mus.Herb. Rijks Univ. Utrecht 281: 233. 1967. (Sa’ad 1967: 233).

Figure 4, t. 17–23.

##### Type.

TURKEY, Merzivan, *Manisadjan* s.n. (holotype W!).

##### Description.

Presumably trailing herb of unknown length; stems and leaves pubescent. Leaves similar to those of *Convolvulus betonicifolius*, petiolate, c. 2.5 × 2 cm, ovate, obtuse and mucronate, entire, shallowly sagittate with short auricles. Flowers 1–2, pedunculate, axillary; peduncles c. 2.5 cm; bracteoles c. 5 × 0.5 mm, linear, attenuate; pedicels equalling bracts; sepals 6 × 3 mm, oblong-oblanceolate, obtuse and retuse, mucronulate, pubescent, inner sepals glabrous, membranous; corolla 2.5 cm long, colour unknown, midpetaline bands pubescent, unlobed; filaments glandular below; ovary glabrous, style 8 mm long, glabrous, stigmas 5 mm. Capsule and seeds unknown.

##### Distribution.

Turkey. Only known from the type collection.

##### Notes.

This species is not conspecific with *Convolvulus arvensis* as stated in the *Flora of Turkey* (Parris 1978) but differs in the pubescent leaves and the pubescent, 6 mm long, herbaceous sepals which lack a membranous border. Instead it is clearly related to the very variable *Convolvulus betonicifolius*, as stated by Sa’ad, but appears to be distinct as we cannot match it with any specimens of *Convolvulus betonicifolius*. It differs in the shorter, obtuse and minutely retuse sepals 5–6 mm long, which lack a distinctive apical portion. The ovary is also glabrous.

#### 13. Convolvulus cassius

Taxon classificationPlantaeSolanalesConvolvulaceae

Sam. ex Rech.f., Ark. Bot., a.s., 1: 314. 1950. (Rechinger 1950: 314).

Figure 4, t. 10–16.

##### Type.

SYRIA, *Dinsmore* 10127 (holotype S; isotype K!).

##### Description.

Twining perennial herb, stems angled, glabrous. Leaves petiolate, 3–4 × 2–2.5 cm, ovate-deltoid, obtuse, margin undulate to crenate or weakly lobed, ciliate, base cordate and attenuate onto the petiole, beneath thinly pubescent. Flowers 1–3 in pedunculate axillary cymes; peduncles 4–14 cm, glabrous; bracteoles linear, acute, 6–8 × 1 mm, ciliate; pedicels 0.8–1 cm, thinly pilose with stiff spreading hairs; outer sepals 9–10 × 5–6 mm, oblong-obovate, slightly pandurate, abruptly constricted at apex into a mucro, the apical portion dark-coloured, pilose with stiff brown hairs; inner sepals glabrous, membranous; corolla 3.2 cm, yellow, unlobed, midpetaline bands thinly pilose towards the apex; filaments glandular below; ovary pilose; style thinly pilose, divided 5 mm above the base; stigmas 2 mm. Capsule and seeds not seen. [Sa’ad 1967: 222]

##### Distribution.

A rare and very local species of the Syrian border with Turkey, known from a handful of collections: Turkey (?); Syria (“Latakia” fide Parris 1978: 216; *Samuelson* 5265).

##### Notes.

Resembling *Convolvulus betonicifolius* and similar species but leaves glabrous except for the ciliate margins, which are crenate up to the apex.

#### 14. Convolvulus stachydifolius

Taxon classificationPlantaeSolanalesConvolvulaceae

Choisy, Prodr. [A.P. de Candolle] 9: 408. 1845. (Choisy 1845: 408).

##### Type.

SYRIA/IRAQ, Aleppo to Mosul, *Olivier* s.n. (lectotype G-DC, designated by Sa’ad 1967: 243); isolectotypes P04209089!, P04209090!).

##### Description.

Perennial herb with decumbent stems up to 1 m long from a central rootstock, vegetative parts pubescent with crisped, somewhat retrorse hairs, occasionally villous to subtomentose. Leaves petiolate, 1.5–6 × 1.5–5.5 cm, ovate-reniform, apex obtuse, margin undulate, crenate-dentate to sinuate-dentate, base cordate and cuneate onto the petiole; petioles 1–4.5 cm. Flowers 1–5 in pedunculate axillary cymes; peduncles 3–9 cm; bracteoles 3–8 mm, filiform; pedicels mostly 1–1.5 cm but sometimes longer resulting in a very lax inflorescence; outer sepals 6–8 × 4–5 mm, obovate or broadly oblong, obtuse, retuse or truncate and mucronate, scarious, pubescent, inner sepals membranous with a truncate base, glabrous or nearly so; corolla (1.5-)2.5–3.5 cm long, pink to purplish, unlobed, midpetaline bands thinly pilose; filaments glandular below; ovary glabrous or with a few apical hairs, style glabrous or sparsely pilose, divided 5 mm above base, stigmas 4 mm. Capsule glabrous; seeds glabrous, strongly tuberculate. [Sa’ad 1967: 243; Feinbrun-Dothan 1978 (plate 70); Tohmé and Tohmé 2007: 216 (photo); Nowroozi 2002: 84 (plate), 105 (map)]

##### Notes.

We recognise two varieties which can distinguished by indumentum and floral characters:

#### 14a. Convolvulus stachydifolius var. stachydifolius

Taxon classificationPlantaeSolanalesConvolvulaceae

Figure 4, t. 24–32.

Convolvulus quadriflorus Hochst., in J.A.Lorent, Wanderungen 335.1845. (Lorent 1845: 335). Type. Bir, *Lorent* s.n. (?B†).

##### Distinguishing features.

Indumentum of leaves and stem puberulent to pubescent; corolla 2.5–3.5 cm long.

##### Distribution.

Eastern Mediterranean region east to Iran, growing as a weed, often in fallow fields: Turkey (*Davis* 42295, *Davis & Hedge* 28188); Iran (*Wright & Bent* 519-103, *Koelz* 14798, *Bélanger* 431); Iraq (*Guest* 1376, 1467, *Rawi et al.* 28127, *Bornmüller* 1529); Syria (*Dinsmore* 3651, *Gaillardot* 2059, *Barkoudah* 1262); Lebanon (*Breidy et al.* LEB-409); Palestine/Israel (*Dinsmore* 7651); Jordan (*Dinsmore* 10620); Egypt.

#### 14b. Convolvulus stachydifolius var. villosus

Taxon classificationPlantaeSolanalesConvolvulaceae

Hallier f., Bot. Jahrb. Syst. 18: 107. 1894 [pub.1893]. (Hallier 1894: 107).

Convolvulus damascenus Boissier & Gaillardot, Diag. Pl. Orient. ser. 2, 6: 122. 1859. (Boissier 1859: 122). Type. SYRIA, Damascus, *Gaillardot* 2058 (holotype G, not seen).

##### Type.

EGYPT, *Aucher-Eloy* 193 (lectotype W!, designated by Sa’ad 1967: 246).

##### Distinguishing features.

Distinguished by its denser villous to tomentose indumentum combined with a smaller corolla about 1.5 cm long.

##### Distribution.

Scattered over the range of the species. Examples seen include *Maitland* 477 (Lebanon), *Gaillardot* 2058 (Syria), *Meyers & Dinsmore* 81776 (Palestine/Israel) and *Simpson* 4714 (Egypt).

##### Notes.

*Convolvulus stachydifolius* is usually easily distinguished from similar species by the sinuate-dentate leaves.

#### 15. Convolvulus palaestinus

Taxon classificationPlantaeSolanalesConvolvulaceae

Boiss., Diagn. Pl. Orient. ser. 1, 11: 84. 1849. (Boissier 1849: 84).

Figure 4, t. 33–41

Convolvulus palaestinus var. diversifolius Boiss., Diagn. Pl. Orient. ser. 1, 11: 85. 1849. (Boissier 1849: 85). Type. TURKEY (Bithynia) (Boissier 1875b: 107), *Pestalozza* s.n. (holotype G).
Convolvulus palaestinus var. stenophyllus Boiss., Diagn. Pl. Orient. ser. 2, 3: 124. 1856. (Boissier 1856: 124). Type. LEBANON, *Blanche* s.n. (holotype G; isotypes P00836226!, P00836227!, P00836228!).
Convolvulus stenophyllus (Boiss.) Boiss., Fl. Orient. [Boissier] 4: 106. 1875. (Boissier 1875b: 106). Type. Based on Convolvulus palaestinus var. stenophyllus Boiss.

##### Type.

PALESTINE/ISRAEL, *Boissier* s.n. (holotype G; isotype P!).

##### Description.

Perennial herb with trailing or twining stems from a woody base 0.4–1 m long; stem and vegetative parts adpressed tomentellous. Leaves petiolate, somewhat dimorphic; lower leaves 3–3.5 × 2–3 cm, broadly to narrowly ovate, acute, margin crenate, base broadly cordate and cuneate onto the petiole; middle and upper leaves with an acute triangular central lobe 3–5 × 0.4–0.6 cm, the margin entire to sinuate, basal auricles deeply lobed with many acute lobes; petioles 0.3–2.3 cm, diminishing in length upwards. Flowers 1–3 in pedunculate axillary cymes; peduncles 1.5–5.5 cm; bracteoles 2–4 mm long, filiform; pedicels 2–8 mm, frequently recurved; outer sepals 8–10 × 4–5 mm, obovate, obtuse, densely pubescent; inner sepals c. 1 mm shorter, obovate-elliptic, rounded and crenate at apex, scarious; corolla 2.2–3 cm long, yellow, unlobed, midpetaline bands shortly pubescent near apex; filaments glandular below; ovary pubescent; style glabrous, divided 8 mm above base, stigmas 2–3 mm. Capsule apically pubescent; seeds verruculose. [Sa’ad 1967: 238; Feinbrun-Dothan 1978: plate 68; Tohmé and Tohmé 2007: 217 (photo as *Convolvulus stenophyllus*)]

##### Distribution.

Lebanon south to Sinai: Palestine/Israel (*Meyers & Dinsmore* 1776, 3776, *Meyers* 3409, *Dinsmore* 10829, *Balls* 1539); Lebanon (*Bornmüller* 12139); Syria (*Gaillardot* 2054); Egypt: Sinai (fide Boulos 2000: 251).

##### Notes.

Resembles *Convolvulus scammonia* in its yellow flowers but inner sepals slightly shorter than outer sepals and plant tomentellous. The leaves are usually dimorphic; the type shows the ovate lower (or first) leaves while that of *Convolvulus stenophyllus* at P has both leaf forms.

#### 16. Convolvulus galaticus

Taxon classificationPlantaeSolanalesConvolvulaceae

Rostan ex Choisy, Prodr. [A.P. de Candolle] 9: 408. 1845. (Choisy 1845: 408).

Figure 5, t. 1–7.

Convolvulus agrophilos C.Koch, Linnaea 22: 745. 1849. (Koch 1849: 745). Type. TURKEY, Tschorukthale, *C.Koch* s.n. (?B†).

##### Type.

TURKEY, Ankara, *Rostan* s.n. (lectotype G-DC, designated by Sa’ad 1967: 227).

##### Description.

Perennial herb with decumbent or prostrate stems spreading from a central tap root and reaching 50 cm, vegetative parts softly tomentose. Leaves petiolate, 1.5–4 × 1–3 cm, ovate to ovate-triangular, apex acute to mucronate, margin undulate, sinuate or, above, weakly 5-lobed, base cordate and shortly attenuate onto the petiole, veins prominent below; petioles 0.5–1.4 cm. Flowers 1–2 in pedunculate, axillary cymes; peduncles 0.8–2.5 cm; bracteoles 2 –4 mm, linear to filiform; pedicels 0.5–1.4 cm; outer sepals 7–10 × 5–8 mm, broadly ovate, rounded and mucronate to acute, somewhat convex, tomentose, greyish, inner sepals c. 7 × 5 mm. glabrous, membranous; corolla 2.6–3 cm long, deep pink, unlobed, midpetaline bands adpressed pilose, terminating in a tooth; filaments glandular below; ovary pilose (or fide Sa’ad (1967: 230) glabrous), style glabrous or thinly pilose, divided 7 mm above base, stigmas 3–3.5 mm long. Capsule and seeds not seen. [Sa’ad 1967: 227; Tohmé and Tohmé 2007: 215 (photo)]

**Figure 5.**
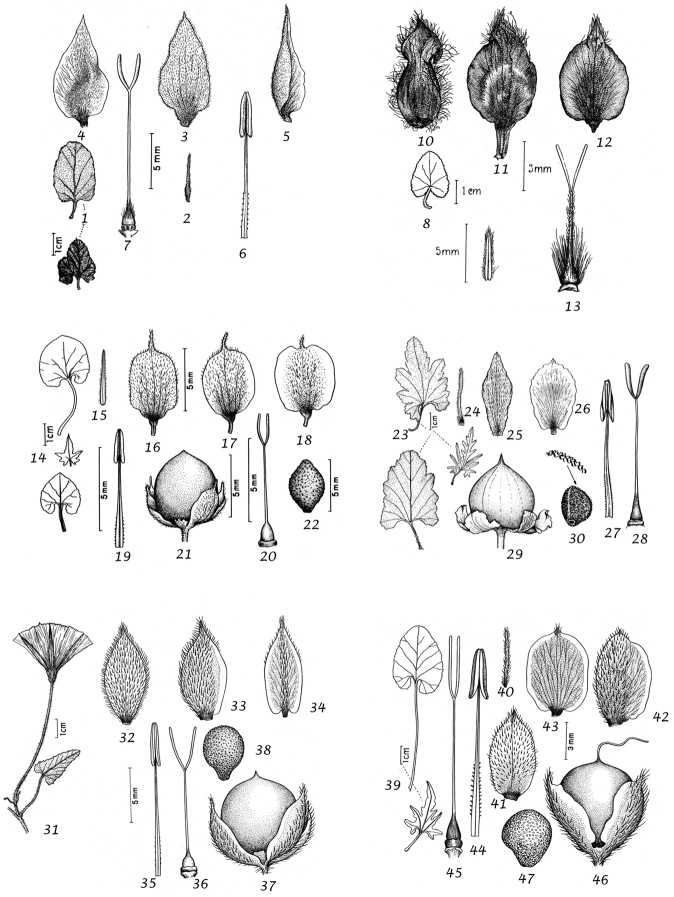
**1–7** *Convolvulus galaticus* **1** leaves **2** bracteole **3** outer epal **4** middle sepal **5** inner sepal **6** stamen **7** ovary and style **1** from *Siehe* 182 (W) **2–7** from *Bourgeau* 171 (W) **8–13** *Convolvulus germaniciae* **8** leaf **9** bracteole **10** outer sepal **11** middle sepal **12** inner sepal **13** ovary and style. From *Haussknecht* s.n. (W) **14–22** *Convolvulus coelesyriacus* **14** leaves **15** bracteole **16** outer sepal **17** middle sepal **18** inner sepal **19** stamen **20** ovary and style **21** capsule **22** seed **14** from *Davis* 2979 (E) **15–20** from *Davis* 3033 (E) **21–22** from *Meyer & Dinsmore* 3619 (L) **23–30** Convolvulus althaeoides subsp. althaeoides **23** leaves **24** bracteole **25** outer sepal **26** inner sepal **27** stamen **28** ovary and style **29** capsule **30** seed **23–28** from *van Soest* 131 (L) **29–30** from *Boulos* s.n. (CAIM) **31–38** *Convolvulus pitardii* 31leaf and flower showing short peduncle and bracteoles **32** outer sepal **33** middle sepal **34** inner sepal **35** stamen **36** ovary and style **37** capsule **38** seed **31** from *Souvage* 2412 (RAB) **32–36** from *Souvage* 2413 (RAB) **37–38** from *Souvage* 14933 (RAB) **39–47** *Convolvulus glaouorum* **39** leaves **40** bracteole **41** outer sepal **42** middle sepal **43** inner sepal **44** stamen **45** ovary and style **46** capsule **47** seed. From *Sauvage & Vindt* 2412 (RAB).

##### Distribution.

Almost restricted to Turkey: Turkey (*Rix* 322, *Bornmüller* 3176, *Siehe* 182, *Sintenis* 6078, *Balls* 516); Iraq? (*Kotschy* 73), Lebanon (fide Mouterde 1978: 37).

##### Notes.

The small ovate-triangular leaves and the softly tomentose indumentum help to identify this species. It might be confused with some forms of *Convolvulus stachydifolius* but the sepals are ovate and rather larger.

#### 17. Convolvulus germaniciae

Taxon classificationPlantaeSolanalesConvolvulaceae

Boiss. & Hausskn., Fl. Orient. [Boissier] 4: 104. 1875. (Boissier 1875b: 104).

Figure 5, t. 8–13

##### Type.

TURKEY, Marash, *Haussknecht* s.n. (holotype G; isotypes JE, W!).

##### Description.

Similar in overall morphology to *Convolvulus galaticus* but differing as follows: plant pilose with spreading hairs, leaves obscurely sinuate-margined but not crenate-lobed as commonly in *Convolvulus galaticus*, flowers mostly paired, the inflorescence commonly reflexed, sepals 7–9 × 3.5–5 mm, broadly elliptic, bicoloured, the apical part terminating in a distinct broad-based mucro; corolla white to pale pink, the ovary always hirsute, style pubescent below, divided 7–7.5 mm above base, stigmas 2–2.5 mm. Capsule pilose; seeds hirsute. [Sa’ad 1967: 230; Aykurt and Sümbül 2011c (photo, plate and full description)]

##### Distribution.

Endemic to Turkey. Previously known only from the type collection but rediscovered in 2008 (Aykurt and Sümbül 2011c). It is clearly very rare.

#### 18. Convolvulus coelesyriacus

Taxon classificationPlantaeSolanalesConvolvulaceae

Boiss., Diagn. Pl. Orient. ser. 1, 11: 85. 1849. (Boissier 1849: 85).

Figure 5, t. 14–22.

Convolvulus sintenisii Boiss., Fl. Orient., Suppl. 349. 1888. (Boissier 1888: 349). Type. CYPRUS, *Sintenis & Rigo* 55 (holotype G; isotype W!).

##### Type.

LEBANON, between Hasbey and Rasheiya, *Boissier* s.n. (holotype G, n.v).

##### Description.

Annual herb, mostly branched at base, with decumbent or ascending stems to c. 30 cm, vegetative parts thinly pubescent. Leaves petiolate, 2–4(-5) × 1.5–3 cm, ovate or reniform, apex rounded, margin entire or undulate, base weakly auriculate, cordate and cuneate onto the petiole; petioles up to 10 cm on basal leaves but mostly 2–3 cm on cauline leaves. Flowers solitary, axillary, pedunculate, becoming congested upwards; bracts resembling small leaves, but sometimes deeply palmately lobed with acute lobes; peduncles 0.5–5 cm, elongating and reflexing in fruit; bracteoles 3–4 mm, filiform to linear-lanceolate; pedicels 0.3–1.5 cm; sepals 3–6 × 3–4 mm, broadly oblong-obovate, prominently mucronate, stiffly hirsute with spreading hairs; corolla 1.5–2(-2.8) cm, pink or pinkish purple, unlobed, midpetaline bands pilose; filaments glandular below; ovary glabrous; style glabrous, divided c. 4 mm above base, stigmas c. 1.5 mm. [Sa’ad 1967: 174; Feinbrun-Dothan 1978: plate 57; Tohmé and Tohmé 2007: 214 (photo); Meikle 1985: 1173]

##### Distribution.

Eastern Mediterranean, apparently especially common in Cyprus: Cyprus (*Davis* 2979, 3033); Turkey; Syria (*Hasbani* 464, *Barbey* 612); Lebanon (*Polunin* 5208, *Gombault* 4497, 4499); Palestine/Israel (*Davis* 4214, 4500, *Eig et al.* 276).

##### Notes.

The retuse, strongly apiculate sepals, reflexed fruiting peduncles and annual habit are distinctive.

*Species 19–21*.

*Convolvulus pitardii*, *Convolvulus glaouorum* and *Convolvulus vidalii* form a complex of species. *Convolvulus vidalii* is the most restricted in distribution and the best defined. *Convolvulus pitardii* and *Convolvulus glaouorum* are more widely distributed, their geographical patterning only partially defined with the former mostly in the Eastern Rif and Middle Atlas while the latter is mostly in the High Atlas. Most specimens are easily assigned to one or other species but further study is needed.

#### 19. Convolvulus pitardii

Taxon classificationPlantaeSolanalesConvolvulaceae

Batt., in Pitard, Explor. Sci. Maroc, Bot. 74. 1913. (Pitard 1913: 74).

Figure 5, t. 31–38.

##### Type.

MOROCCO, Oued Cherrat, *C.-J. Pitard* 2977 (holotype P00332177!).

##### Description.

Perennial herb with stout somewhat woody rootstock from which arise various short decumbent, subglabrous, pubescent to pilose stems to 50 cm. Leaves petiolate, 0. 8–3.2 × 7.5–3.5 cm, ovate-deltoid or reniform, rounded to obtuse, margin undulate to coarsely serrate, base cordate, usually shortly and softly tomentose-sericeous but sometimes with longer hairs, occasionally subglabrous; petioles 0.5–0 7 cm, often flexuose. Flowers solitary, borne on axillary peduncles; peduncles 0–1 cm; bracteoles 2.5–7 mm, filiform; pedicels 1–8.5 cm, commonly flexuose and somewhat deflexed in fruit; calyx in flower clearly longer than broad, sepals 4.5–9 × 3–6 cm, lanceolate to linear-oblong, acute to apiculate forming a narrow calyx, the inner sepals broader; corolla 2.2–3(-4) cm long, pink with a darker centre, midpetaline bands sericeous near apex; filaments glandular below; ovary glabrous; style glabrous, divided c. 7 mm above base; stigmas 3–4 mm. Capsule glabrous; seeds finely tuberculate. [Sa’ad 1967: 239]

##### Notes.

Distinguished from *Convolvulus vidalii* and *Convolvulus glaouorum* by the narrow calyx (lanceolate in outline) and the narrow sepals which are much longer than broad. This species is divided into two varieties:

#### 19a. Convolvulus pitardii var. pitardii

Taxon classificationPlantaeSolanalesConvolvulaceae

##### Distinguishing features.

Leaves glabrous above

##### Distribution.

Morocco (Only known from the type).

#### 19b. Convolvulus pitardii var. leucochnous

Taxon classificationPlantaeSolanalesConvolvulaceae

(Benoist) Maire, Bull. Soc. Hist. Nat. Afrique N. 22: 57. 1931. (Maire 1931a: 57).

Convolvulus leucochnous Benoist, Bull. Mus. Hist. Nat. (Paris) 27: 112. 1921. (Benoist 1921: 112). Type. MOROCCO, Ain Leuh, *Benoist* 384 (holotype P00332176!).

##### Type.

Based on *Convolvulus leucochnous* Benoist

##### Distinguishing features.

Leaves sericeous. The long flexuose pedicels are also very distinct. Much more common than the type variety.

##### Distribution.

Endemic to Morocco where it usually grows on schists: Central Rif (*Carine et al.* 322; *Jury & Shakwa* 20997, *Font Quer* 358, *Bowring* 10) and Zaïan, east of Middle Atlas (*Lynes* 153, *Jahandiez* 80b, *Davis* 557, *Gattefossé* s.n. [3/4/1936], *Maire* s.n. [18/4/1926], *Sauvage* 1359, 8097, 8410).

#### 20. Convolvulus glaouorum

Taxon classificationPlantaeSolanalesConvolvulaceae

Braun-Blanquet & Maire, Bull. Soc. Hist. Nat. Afrique N. 13: 18. 1922. (Braun-Blanquet and Maire 1922: 18).

Figure 5, t. 39–47

Convolvulus pitardii var. glaouorum (Braun-Blanquet & Maire) Sauvage et Vindt, Fl. Maroc 3: 28. 1954. (Sauvage and Vindt 1954: 28). Type. Based on *Convolvulus glaouorum* Braun-Blanquet & Maire
Convolvulus mesatlanticus Andr., Ind. Hort. Bot. Univ. Budapest 1934: 112. 1934. (Andreánzky 1934: 112). Type. MOROCCO, Azrou, no details of collector or collection given (holotype BP?).

##### Type.

MOROCCO, Demnate, *R. Maire* s.n. (lectotype MPU 000022!, designated here; isolectotypes P!, AL?).

##### Description.

Perennial herb with relatively slender rootstock (c. 3 mm wide) from which emerge various short decumbent or ascending stems 5–15 (-20) cm long, vegetative parts pubescent. Leaves petiolate, 2–4 × 1–3 cm, dimorphic, lower leaves ovate-deltoid, obtuse, margin undulate to dentate, base truncate to shallowly cordate and shortly cuneate on the petiole; upper leaves somewhat smaller, strongly dentate, apex acute; petioles 1–5 cm. Flowers solitary on axillary peduncles; peduncles 0.3–2.5 cm long, very variable in length; bracteoles 4–6 mm, filiform; pedicels 1–2.5 cm, commonly flexuose; calyx in flower about as long as broad, outer sepals 4.5–7 × 3.5–6 mm, oblong-obovate, mucronate, adpressed pubescent; inner sepals c. 7 × 5 mm, broadly obovate, mucronate, soon scarious; corolla 2.6–3.3 cm, white or pink, unlobed, midpetaline band terminating in a point, nearly glabrous (slightly scabrous); filaments glandular below; ovary glabrous; style glabrous, divided 9 mm above base; stigmas 4 mm. Capsule glabrous; seeds finely tuberculate. [Sa’ad 1967: 231]

##### Distribution.

Endemic to Morocco: High Atlas (*Davis* 54093, *Davis & King* 68145, 68533, *Jahandiez* 7, *Whiting & Richmond* 228, *Weiller* 270, *Maire* s.n. [8/4/1926], *Podlech* 45982, *Guzmán et al.* s.n. [23/3/1989]) with isolated stations at Fez (*Trethewy* 370) and Djebel Tazzeka (*Jury et al.* 16800). Usually on limestone.

##### Notes.

Similar to *Convolvulus vidalii* and *Convolvulus pitardii*, differing from the former by presence of peduncles, the colouring of the corolla and larger sepals and from the latter by its dwarf habit and obovate sepals, the calyx only slightly longer than broad. The short, possibly ascending stems are characteristic. *Whiting & Richmond* 59 (BM) seems intermediate between this species and *Convolvulus pitardii* in indumentum and sepal form.

It appears that the sheet with the original collection in Maire’s herbarium was cut in two and part deposited at Montpelier. This part is selected as the lectotype. The other part of the sheet may be at AL. We have not been able to trace type material of *Convolvulus mesatlanticus* but the illustration provided by Andreánszky (1934: 115) appears to be of *Convolvulus glaouorum* and this concurs with the opinion of Dobignard and Chatelain (2011: 338).

#### 21. Convolvulus vidalii

Taxon classificationPlantaeSolanalesConvolvulaceae

Pau, Bol. Soc. Esp. Hist. Nat. 21: 279. 1921. (Vidal y López 1921: 279).

##### Type.

MOROCCO, *Vidal y López* s.n. (holotype MA!; isotype BC).

##### Description.

Perennial herb from a stout tap root with decumbent stems to 30 cm, vegetative parts pilose. Leaves petiolate, 0.7–2.8 × 0. 5–3.3 cm, dimorphic, lower leaves ovate-deltoid, obtuse, margin crenate, base cordate to truncate, upper leaves deltoid, apex acute, margin incised-lobed, base cordate; petioles 1–3 cm, flexuose. Flowers solitary, borne on axillary pedicels; peduncles absent; bracteoles 2–4 mm, filiform; pedicels 3–35 mm, becoming strongly recurved in fruit; sepals 2.5–5.5 × 2.5–5.5, ovate to obovate, acute or obtuse and apiculate; corolla 1.7–3 cm long, purple with cream centre and (usually) dark purple marks around throat, midpetaline bands pilose towards apex; filaments glandular below; ovary glabrous; style glabrous, divided 5–9 mm above base; stigmas c. 4 mm. Capsule glabrous; seeds finely tuberculate.

##### Distribution.

Endemic to the Western Rif in Morocco (*Carine et al.* 239, *Font Quer* 318, *Wall* 22/5/1936).

##### Notes.

Distinguished from the *Convolvulus pitardii* and *Convolvulus glaouorum* by the complete absence of peduncles, the strongly recurved fruiting pedicels, the shorter sepals and smaller corolla, this is purple with a cream centre and with five distinct dark purple markings in the throat.

#### 22. Convolvulus althaeoides

Taxon classificationPlantaeSolanalesConvolvulaceae

L., Sp. Pl. 156. 1753. (Linnaeus 1753: 156).

Figure 5, t. 23–30

##### Type.

Southern Europe, (lectotype LINN 218.26!, designated by Sa’ad 1967: 210).

##### Description.

Trailing or twining perennial herb with slender creeping rootstock; stems terete, to 2 m long; vegetative parts thinly pilose to densely sericeous-tomentose. Leaves petiolate, strongly dimorphic; lower leaves 2–4 × 1–3 cm, ovate-deltoid, apex apiculate, acute or obtuse, margin irregularly crenate, base cordate and shortly cuneate; upper leaves slightly larger, to 6 × 6 cm, similar in outline but deeply sinuate-lobed to 3–5-partite with narrowly oblong, entire to coarsely dentate segments; petioles 1–5 cm long. Flowers axillary, pedunculate, solitary or in a dichasial cyme with up to 4 flowers; peduncles 3–10 cm; bracteoles 3–12 mm, filiform, linear to narrowly linear-lanceolate; pedicels 5–13 mm; outer sepals 5–9 × 4–6 mm, variable in shape, elliptic to obovate, acute to obtuse, glabrous to hirsute, margin often scarious, undulate, inner sepals slightly broader with broad scarious margins, often basally auriculate; corolla (1.6-)1.8–4.5 cm long (very variable in size), pink (rarely white), very weakly lobed, midpetaline bands darker, shortly pubescent; filaments glandular below; ovary glabrous; style glabrous, divided 5–10 mm above base; stigmas 2. 5–5 mm, relatively stout. Capsule glabrous; seeds glabrous, obscurely tuberculate. [Sa’ad 1967: 210]

##### Notes.

A very variable species in indumentum, sepal form and flower size, this reflected in the extensive synonymy below. Two subspecies, sometimes treated as separate species, can usually be distinguished although intermediates occur occasionally, for example *Fitz* 74/1978 (W) from Tunisia, *Dubuis* 8549 (BM) from Algeria, *Pampanini 3729* (FI), the type of var. angustisectus from Libya or *Faure* s.n. (MPU) the type of var. dissectus, from Algeria, all of which have the distinct linear leaf lobes of subsp. tenuissimus without the softly sericeous indumentum.

#### 22a. Convolvulus althaeoides subsp. althaeoides

Taxon classificationPlantaeSolanalesConvolvulaceae

Convolvulus gracilis Salisb., Prodr. Stirp. Chap. Allerton 124. 1796, superfluous name for *Convolvulus althaeoides* L. (Salisbury 1796: 124). Type. FRANCE, Sète [Cette], collector and whereabouts unknown.
Convolvulus bryonifolius Sims, Bot. Mag. t. 943. 1806. (Sims 1806: t. 943).
Convolvulus althaeoides subsp. althaeoides Type. Cultivated at Brompton, t. 943 (Sims 1806) based on a cultivated plant of uncertain origin.
Convolvulus hirsutus Tenore, Fl. Napol. 1: 60, t. 15. 1811-15, nom. illeg., non *Convolvulus hirsutus* M.Bieb. (1808). (Tenore 1811-15: 60). Type. ITALY, Capri and Ischia, *Tenore* s.n. (NAP).
Convolvulus argyraeus DC., in Lamarck & de Candolle, Fl. Franc. ed. 3. 6: 423. 1815. (Lamarck and de Candolle 1815: 423). Type. ITALY, Calabria (holotype G?).
Convolvulus italicus Roem. & Schult., Syst. Veg, ed. 15 bis [Roemer & Schultes] 4: 266. 1819. (Roemer and Schultes 1819: 266). Type. Based on *Convolvulus hirsutus* Tenore
Convolvulus alceifolius Bory & Chaub., Nouv. Fl. Pélop. 14. 1838. (Bory and Chaubard 1838: 14). Type. GREECE, between Koron and Modon, *Chaubard* s.n. (lectotype P00608773!, designated by Sa’ad 1967: 212).
Convolvulus althaeoides var. nanus Choisy, Prodr. [A.P. de Candolle] 9: 409. 1845. (Choisy 1845: 409). Type. Not specified.
Convolvulus althaeoides var. hirsutus Choisy, Prodr. [A.P. de Candolle] 9: 409. 1845. (Choisy 1845: 409). Type. Based on *Convolvulus hirsutus* Tenore
Convolvulus althaeoides var. ferrugineus Choisy, Prodr. [A.P. de Candolle] 9: 409. 1845. (Choisy 1845: 409). Type. Not specified.
Convolvulus althaeoides var. sericeus Choisy, Prodr. [A.P. de Candolle] 9: 409. 1845. (Choisy 1845: 409). Type. Image in Thesaurus botanicus, t. 57. (Trattinnick 1805-1819).
Convolvulus althaeoides var. argyreus Choisy, Prodr. [A.P. de Candolle] Prodr. 9: 409. 1845. (Choisy 1845: 409). Type. Based on *Convolvulus argyraeus* DC.
Convolvulus althaeoides var. angustisectus Pamp., Bull. Soc. Bot. Ital. 1914: 15.1914. (Pampanini 1914: 15). Type. LIBYA, Ras Tecut, *Pampanini* 3729 (holotype FI).
Convolvulus althaeoides var. albidiflorus Braun-Blanq. & Maire, Bull. Soc. Hist. Nat Afrique N. 13: 19. 1922. (Braun-Blanquet and Maire 1922: 19). Type. MOROCCO, Djebbel Amsitten, S de Mogador, *Maire* s.n. (holotype RAB 078144!).
Convolvulus althaeoides var. repandus Faure & Maire, Bull. Soc. Hist. Nat Afrique N. 23: 200. 1932. (Maire 1932: 200). Type. ALGERIA, Oran, *Faure* s.n. (holotype MPU 002967!).
Convolvulus althaeoides var. dissectus Faure & Maire, Bull. Soc. Hist. Nat Afrique N. 23: 200. 1932. (Maire 1932: 200). Type. ALGERIA, Lamoricière, *Faure* s.n. (holotype MPU 002968!).
Convolvulus althaeoides subsp. darnitanus Maire, Bull. Soc. Hist. Nat Afrique N. 30: 293 (Maire and Weiller 1939: 293). Type. LIBYA, Cyrenaica, Wadi Derna, *Maire & Weller* 1117 (holotype MPU!).

##### Distinguishing features.

Variably hirsute but hairs not appressed and sericeous. Leaves variously dissected but lobes not linear. [Feinbrun-Dothan 1978: plate 64; Tohmé and Tohmé 2007: 213 (photo); Silvestre 2012: 257; Siddiqi 1977: 9 (Figure 3); Strid and Strid 2009: 390–391 (plate); Pignatti 1982: 389; Boulos 2000: 331].

##### Distribution.

Madeira (*MacGilvray* 54); Canary Islands (*Gilli* 23/6/1977); Portugal (Moller 1177); Spain (*Beltrán* s.n. [5/1933]); France (*Salis* 1821); Sardinia (*Titchen* 123); Italy (*Fiori & Beguinot* 2145); Sicily (*Zerny* 337); Malta–Gozo (*Hepper* 4818); Morocco (*Balls* 2935); Algeria (*Faure* s.n. [30/4/1920], Choulette 375); Tunisia (*Buxbaum & Schussnig* s.n. [18/4/1924]); Libya (*Pampanini* 6179); Egypt (*Boulos* 19773); Palestine/Israel (*Meyers & Dinsmore* 620); Syria (Gombault 3998); Jordan (*Swann* s.n.[4/1976]); Lebanon (fide Tohmé and Tohmé 2007); Turkey (*Davis* 41803); Crete (*Barclay* 3637); Rhodes (*Bourgeau* s.n.[16/5/1871]); Cyprus (*Sintenis & Rigo* 59). Naturalised in the United States of America: California (*True & Howell* 7467).

#### 22b. Convolvulus althaeoides subsp. tenuissimus

Taxon classificationPlantaeSolanalesConvolvulaceae

(Sm.) Batt., Fl. Algérie 592. 1890. (Battandier 1890: 592)

Convolvulus elegantissimus Mill., Gard. Dict. ed. 8: 22. 1768. (Miller 1768: 22). Type. A plant cultivated in the Chelsea Physic Garden, *Miller* s.n. (holotype BM001035799!).
Convolvulus sericeus Forssk., Fl. Egypt-Arab. 204. 1775, nom. illeg., non *Convolvulus sericeus* L. (1767). (Forsskål 1775: 204). Type. TURKEY, Sea of Marmora, *Forsskål* (syntypes C).
Convolvulus tenuissimus Sm., Fl. Graec. Prodr (Sibthorp & Smith) 1: 134. 1806. (Sibthorp and Smith 1806: 134). Type. GREECE, collector unknown (holotype OXF-SIB0463A!).
Convolvulus althaeoides var. pedatus Choisy, Prodr. [A.P. de Candolle] 9: 409. 1845. (Choisy 1845: 409). Type. Without locality, *Forsskål* s.n. (lectotype BM001014569!, designated here).
Convolvulus althaeoides subsp. elegantissimus (Mill.) Quézel & Santa, Nouv. Fl. Algérie 758. 1963. (Quézel and Santa 1963: 758). Type. Based on *Convolvulus elegantissimus* Mill.

##### Type.

Based *Convolvulus tenuissimus* Sm.

##### Distinguishing features.

Plant softly sericeous in all parts. Leaves finely dissected with narrow linear lobes. [Tohmé and Tohmé 2007: 215 (photo as *Convolvulus elegantissimus*); Pignatti 1982: 389 (as *Convolvulus elegantissimus*); Polunin 1980 (Plate 35); Strid and Strid 2009: 392–393 (plate as *Convolvulus elegantissimus*)].

##### Distribution.

Mostly East Mediterranean region with scattered records in the west: Turkey (*Davis* 41184, *Sintenis* 338); Lebanon (*Mouterde* 12135); Aegean Islands (*Platt* 86, *Rechinger* 3816); Greece (*Heldreich* 235); Albania (*Baldacci* 1892); Croatia (*Denis* 121); Hungary (*Degen* 2934); Rumania (*Degen* 90); Italy (*Fiori & Béguinot* s.n. [1924]); Malta (*Adler* s.n. [12/5/1994]); France (*Bruyas* 3345); Malta (*Davis* 50632); Algeria (*Gombault* s.n. [24/3/1935]).

##### Notes.

The species as a whole is widely distributed around the Mediterranean extending to Madeira and the Canary Islands, where it may be introduced. Subsp. tenuissimus is the predominant subspecies in SE Europe extending from southern Austria and Hungary to Italy, Malta, Algeria, the Aegean Islands and Turkey, perhaps centred on the Adriatic. It should be noted that if this subspecies is recognised at specific level its correct name is Convolvulus elegantissimus Mill. subsp. althaeoides is the only subspecies in the west Mediterranean region and the predominant subspecies in Cyprus, the Levant and North Africa. It is naturalised in California (Hickman 1993: 521). Records from Eritrea (Sebsebe 1999: 78), where it is presumably a garden plant or garden escape, do not specify a subspecies.

This species is morphologically very similar to *Convolvulus capensis* from South Africa. Both species are extremely variable and some forms are not easily distinguished except on geographical grounds. Molecular studies, however, indicate they belong to different clades and *Convolvulus capensis* has distinctly shorter, thicker stigmas (Williams et al. 2014).

### Species 23–41. Southern African species

This group is exceptionally complex and there are few clearly demarcated species. Although *Convolvulus sagittatus* has long been recognised as the centre of a complex of species, the difficulties in species delimitation extend to almost every species in the group apart from perhaps *Convolvulus argillicola* and *Convolvulus kilimandschari*. Apparent intermediates between species are quite frequently found and in the absence of any clear evidence for hybridisation are difficult to explain. Any attempt to unite two taxa joined by intermediates will tend to result in a series of species collapsing into a single amorphous unit. Attempts have been made here to pick out what seem to be the core taxa and indicate the existence of intermediates. No attempt has been made to describe as new the occasional single collections, which cannot easily be accommodated. *Convolvulus sagittatus*, *Convolvulus aschersonii*, *Convolvulus thomsonii*, *Convolvulus austroafricanus* and *Convolvulus farinosus* form an especially complex group of species, which extend from South Africa northwards to Nigeria, Algeria, Ethiopia and SW Arabia but molecular studies tend to support their recognition as separate species The first two are very similar to the South American *Convolvulus demissus* and *Convolvulus bonariensis* respectively but molecular studies suggest they are not very closely related (Williams et al. 2014). The three species (*Convolvulus capensis*, *Convolvulus bidentatus* and *Convolvulus namaquensis*) share very short, somewhat stout stigmas, which set them apart from the other southern African species and are unusual in *Convolvulus* as a whole.

#### 23. Convolvulus kilimandschari

Taxon classificationPlantaeSolanalesConvolvulaceae

Engl., Abh. Königl. Akad. Wiss. Berlin 2: 348. 1892. (Engler 1892: 348).

Figure 6, t. 1–6

Convolvulus schimperi Engl., Abh. Königl. Akad. Wiss. Berlin 2: 347. 1892, nom. illeg., non *Convolvulus schimperi* Boiss. (1849). (Engler 1892: 347). Type. ETHIOPIA, Begemeder, *Schimper* 1465 (holotype B†; isotype K).
Convolvulus kilimandschari var. glabratus Hallier f., Bot. Jahrb. Syst. 18: 109. 1893 [“1894”]. (Hallier 1894: 109). Type. Based on *Convolvulus schimperi* Engl.
Convolvulus cephalantha Baker, Bull. Misc. Inform. Kew 1894: 69. 1894. (Baker 1894: 69). Type. TANZANIA, Kilimanjaro, *H.H. Johnston* s.n. (holotype K).
Bonamia althoffiana Dammer, Pflanzenw. Ost-Afrikas, C 329. 1895. (Engler 1895: 329). Type. TANZANIA, Kilimanjaro, Kilema, *Volkens* 1559 (holotype B†; isotype BR).
Hewittia kilimandschari (Engl.) Hallier f., Bull. Herb. Boiss 5: 1008. 1897. (Hallier 1897: 1008). Type. Based on *Convolvulus kilimandschari* Engl.
Convolvulus keniensis Standl., Smithsonian Misc. Collect. 68(5): 11. 1917. (Standley 1917: 11). Type. KENYA, western slopes, Mt. Kenya, *Mearns* 1294 (holotype US).
Calystegia glabrata (Hallier f.) Chiov., Racc. Bot. Miss. Consol. Kenya 85. 1935. (Chiovenda 1935: 85). Type. Based on Convolvulus kilimandschari var. glabratus Hallier f.

##### Type.

TANZANIA, Kilimanjaro, *H. Meyer* 302 (holotype B†); TANZANIA, Kilimanjaro [Kilimanscharo], 4 miles below Bismarck Hut, 16 Jan 1955, *B. Verdcourt* 1207 (neotype EA, designated by Verdcourt 1963: 38); isoneotypes FT, K!, MO, PRE).

##### Description.

Vigorous twining herb with stems reaching 2 m, vegetative parts varying from densely hirsute to subglabrous. Leaves petiolate, 3–8.5 × 1.8–6 cm, ovate-deltoid, acute, margin entire or obscurely crenate, base cordate (rarely hastate); petioles 7–30 mm. Flowers in many-flowered, axillary, pedunculate, bracteate heads; peduncles 1–13 cm long; bracteoles 5–10 × 3–7.5 mm, ovate, acute, scarious, tardily caducous; pedicels very short, 2–4 mm long; outer sepals 9–13 × 5–8 mm, broadly ovate, acute to apiculate, villous, becoming scarious; corolla 2.5–3(-4) cm long, very wide at the mouth, white, purplish or pink with a dark centre, unlobed, midpetaline bands pubescent; ovary glabrous; style glabrous, divided c. 7 mm above base; stigmas 2–2.5 mm, linear, slightly shorter than the style arm. Capsule glabrous; seeds glabrous, the surface with wavy, white-topped ridges. [Verdcourt 1963: 38–39 (plate); Sebsebe 2006 182]

**Figure 6.**
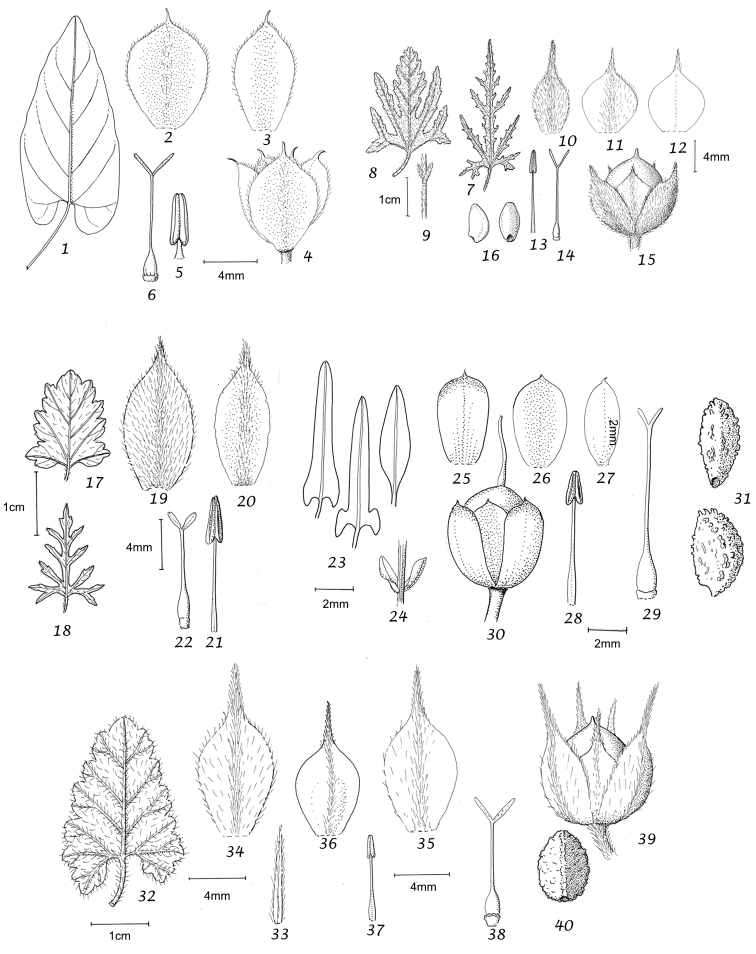
**1–6** *Convolvulus kilimandschari* **1** leaf **2** outer sepal **3** inner sepal **4** calyx **5** stamen **6** ovary and style. From *Gilbert* 1086 (K) **7–16** *Convolvulus thunbergii* **7** leaf **8** leaf **9** bracteoles **10** outer sepal **11** middle sepal **12** inner sepal **13** stamen **14** ovary and style **15** capsule **16** seeds **7 & 10–14** from *Schlieben* 7092 (K) **8–9 & 15–16** from *MacOwan* 586 (K) **17–22** *Convolvulus capensis* **17** leaf **18** leaf **19** outer sepal **20** inner sepal **21** stamen **22** ovary and style **17** from *Drège* s.n. (OXF) **18–22** from *Bolus* 9971 (K) **23–31** *Convolvulus dregeanus* **23** leaves **24** bracteoles **25** outer sepal **26** middle sepal **27** inner sepal **28** stamen **29** ovary and style **30** capsule **31** seeds. From *Gemmell* 7/11/1949 (K) **32–40** *Convolvulus argillicola* **32** leaf **33** bracteole **34** outer sepal **35** middle sepal **36** inner sepal **37** stamen **38** ovary and style **39** capsule **40** seed. From *Seydel* 4170 (K).

##### Distribution.

Mountains of eastern Africa from 1800 to 3500 m:. Ethiopia (*Friis et al.* 7359, *de Wilde* 8973); Kenya (*Fries & Fries* 459, *Greenway & Kanuri* 13886, *Tweedie* 1738); Uganda (*Wesche* 797); Tanzania (*Verdcourt* 1553, *Richards* 24124).

##### Notes.

A very distinctive afromontane species because of its many-flowered capitate inflorescence but variable in indumentum, plants from Ethiopia commonly less hirsute and with slightly larger corollas than those from further south.

#### 24. Convolvulus capensis

Taxon classificationPlantaeSolanalesConvolvulaceae

Burm.f., Fl. Ind. (N.L. Burman) Prodr. Fl. Cap. 5. 1768. (Burman 1768: 5).

Figure 6, t. 17–22.

Convolvulus plicatus Desr., Encycl. [J. Lamarck et al.] 3: 558. 1792. (Desrousseaux 1792: 558). Type. SOUTH AFRICA, *Sonnerat* s.n. (holotype P-Lam, not seen).
Convolvulus alceifolius Lam., Tabl. Encycl.1: 461. 1793. (Lamarck 1793: 461). Type. SOUTH AFRICA, *sin col.* (P [Herb. Lam.]).
Convolvulus falkia Jacq., Hort Schoenbr. 2: 38, t. 198. 1797, nom illeg., non *Convolvulus falkia* Thunb. (1794). (Jacquin 1797: 38). Type. Icon, t.198 in Hort. Schoenbr.
Convolvulus filiformis Thunb., Fl. Cap. 2 :16. 1818, nom. illeg., non *Convolvulus filiformis* Desr. (1789). (Thunberg 1818: 16). Type. SOUTH AFRICA, *Thunberg* s.n. (various syntypes UPS).
Convolvulus inconspicuus Hallier f., Bot. Jahrb. Syst. 18: 106. 1893 [“1894”]. (Hallier 1894: 106). Type. SOUTH AFRICA, Western Cape, Namaqualand, Lilyfontein, *Drège* s.n. (holotype B†; isotypes K!, L, W!).
Convolvulus capensis var. dissectus Hallier f., Bot. Jahrb. Syst. 18: 105. 1893 [“1894”], nom. illeg., superfluous name for autonymic variety (Hallier 1893: 105).
Convolvulus capensis var. malvifolius Hallier f., Bot. Jahrb. Syst. 18: 106. 189. 1893 [“1894”]. (Hallier 1893: 106). Type. SOUTH AFRICA, Cape, *Zeyher & Eckler* 24 (syntype ?B†), *Drege*, ‘C. alceifolius’ a, b, (syntype ? B†).
Convolvulus capensis var. plicatus (Desr.) Baker, Fl. Cap. (Harvey) 4(2): 78. 1904. (Baker and Wright 1904: 78). Type. Based on *Convolvulus plicatus* Desr.

##### Type.

SOUTH AFRICA, Cape, without collection data (holotype G, not seen).

##### Description.

Perennial herb, usually coarsely brown-pubescent on all vegetative parts, occasionally glabresent or white-pubescent; stems to 1.5 m, climbing or prostrate, sometimes woody towards the base. Leaves petiolate, 1.5–5 × 1.5–4 cm, very variable in form but always palmately veined, oblong, ovate or reniform in outline, sometimes unlobed with coarsely crenate to laciniate margins, often deeply palmately lobed or palmatisect, base more or less hastate, apex acute or obtuse; petioles 0.4–1.5 cm. Flowers solitary or arranged in few-flowered cymes, peduncles 4–10 cm; pedicels 4–15 mm; bracteoles 4–7 mm, filiform to linear; outer sepals 8–13 × 5–8 mm, oblong-ovate, acute or acuminate, often scarious-margined; corolla 2–3.2 cm, white, unlobed, fimbriate, the midpetaline bands thinly pilose, terminating in a small tooth; ovary glabrous; style glabrous, divided 5–6 mm above base, stigmas 1.5–2 × 0.5–0.75 mm, very narrowly ellipsoid. Capsule glabrous; seeds glabrous, smooth except for the obscurely rugose angles. [Meeuse 1958: 692; Meeuse and Welman 2000: 40 (map)]

##### Distribution.

South Africa: Eastern and Western Cape (*Parker* 4618, *Bolus* 5211, *Thompson* 768, *Garside* 1610, *Acocks* 14813, *Galpin* 10544).

##### Notes.

Recognised by the large unlobed, often fimbriate, white corolla combined with palmately-veined leaves and very short, thick stigmas. Some specimens are almost indistinguishable from the Mediterranean *Convolvulus althaeoides*, except by the distinctive stigmas.

Plants from Namaqualand (northwestern Cape) have a shorter calyx (6–7 mm long) and smaller corolla (12–16 mm) and have been treated as a distinct species, *Convolvulus inconspicuus*. They resemble *Convolvulus multifidus* very closely but can be distinguished by the presence of a distinct peduncle. The more distinctly elongate, 2 mm long stigmas of the type suggest they might be hybrids: *C. capensis × multifidus*.

#### 25. Convolvulus bidentatus

Taxon classificationPlantaeSolanalesConvolvulaceae

Bernh. ex Krauss, Flora 27(2): 829. 1844. (Krauss 1844: 829).

Convolvulus hastatus Thunb., Prodr. Fl. Cap. 35. 1794, nom illeg., non *Convolvulus hastatus* Forssk. (1775). (Thunberg 1794: 35). Type. SOUTH AFRICA, *Thunberg* s.n. (holotype UPS).
Convolvulus hastatus var. major , Hallier f., Bot. Jahrb. Syst. 18: 105.1893 [“1894”], nom. illeg., based on *Convolvulus bidentatus*. (Hallier 1893: 105). Type. Based on *Convolvulus bidentatus* Bernh. ex Krauss

##### Type.

SOUTH AFRICA, Western Cape, George near Zivarte valley, *Krauss* s.n. (holotype B†; isotypes FI!, W!).

##### Description.

Perennial herb, glabrous to thinly pubescent in all vegetative parts; rootstock thick; stems to 3 m, prostrate or climbing. Leaves petiolate, very narrowly hastate, the central lobe 2–6 × 0.1–0.6 cm, linear to oblong, auricles very small, 0.2–1.2 × 0.1–0.4 cm, usually bifid, apex acute, margin entire; petioles 0.3–1.4 cm. Flowers axillary, pedunculate, usually paired (rarely solitary), peduncles 3–8(-14) cm; bracteoles 3–4 mm, subulate to narrowly lanceolate; pedicels 2–11 mm; outer sepals (5-)6–9 × (4-)6–8 mm, obovate, the apex truncate or rounded and often mucronate, margins scarious; corolla (1.2-)1.5–2.5(-3) cm long, white or pinkish, shallowly lobed, the midpetaline bands thinly pubescent terminating in teeth; ovary glabrous; style glabrous, divided 15 mm above base; stigmas ellipsoid, c 1 × 0.75 mm. Capsule glabrous; seeds glabrous, rugose with pallid ridges. [Meeuse 1958: 685; Meeuse and Welman 2000: 39 (map)]

##### Distribution.

South Africa: along the southern fringes of Western and Eastern Cape (*Acocks* 23072, *Bolus* 2405, *Johnson* 1105, *Fourcade* 2626, *Long* 883, *Zeyher* 239).

##### Notes.

Recognised by the very narrow, hastate leaves combined with rounded, scarious-margined sepals, 2-flowered peduncles and relatively large corolla. The distinctive stigma suggests a close affinity with *C. capensis. Long* 822 from Port Elizabeth has 3-flowered peduncles and stigmas 3 mm long and might be of hybrid origin but has the distinctive scarious sepals of *Convolvulus bidentatus*.

Meeuse (1958: 685) selected *Thunberg* s.n. (UPS) as a neotype but this was unnecessary as there are isotypes of the Krauss collection in the Webb herbarium at FI and at W.

#### 26. Convolvulus namaquensis

Taxon classificationPlantaeSolanalesConvolvulaceae

(Schltr. ex A.Meeuse) J.R.I.Wood & R.W.Scotland stat. nov.

urn:lsid:ipni.org:names:77147669-1

Convolvulus sagittatus Thunb. var. namaquensis Schltr. ex A.Meeuse, Bothalia 6: 682. 1958 [1957]. (Meeuse 1958: 682).

##### Type.

SOUTH AFRICA, Western Cape, *Schlechter* 11124 (holotype PRE!; isotypes BM000930470!, K!, P!).

##### Description.

Very similar to *Convolvulus bidentatus* differing in little more than the obovate, pubescent sepals, 6–7 mm long, which are abruptly narrowed to an acute to apiculate apex and are not scarious-margined. The peduncles can be up to 5-flowered. Leaves 1.4–4 × 0.8–1.5, narrowly ovate-deltoid to oblong in outline, hastate at base but auricles simple, margin sinuate or coarsely crenate; corolla 1.7–2 cm long; ovary conical, glabrous, style divided 5–6 mm above base; stigmas 1 mm, narrowly ellipsoid, sometimes unequal in length as in the isotype at K.

##### Distribution.

South Africa (Western Cape, especially in the Clanwilliam area) and Namibia centred on Namaqualand (*Le Roux* 2836, *Hardy & Bayliss* 1073, *Hugo* 6993, *Bolus* 9424, *Dickson* 1879, *Moss* 17985, *Macdonall* 18, *Galpin* 10544, *Leipoldt* 321).

##### Notes.

Molecular studies (Williams et al. 2014) suggest this species is distinct from *Convolvulus sagittatus*. The shape of the stigmas suggests a relationship with *Convolvulus bidentatus* and *Convolvulus capensis*, rather than *Convolvulus sagittatus*.

We have only cited the isotypes we have seen. The isotype at W appears to represent *Convolvulus sagittatus* and it is possible that not all collections of *Schlechter* 11124 represent *Convolvulus namaquensis* and this may explain why Meeuse treated this as a variety of *Convolvulus sagittatus*.

#### 27. Convolvulus thunbergii

Taxon classificationPlantaeSolanalesConvolvulaceae

Roem. & Schult., Syst. Veg, ed. 15 bis [Roemer & Schultes] 4: 268. 1819. (Roemer and Schultes 1819: 268).

Figure 6, t. 7–16.

Convolvulus transvaalensis Schltr., J. Bot. 34: 502. 1896. (Schlechter 1896: 502). Type. SOUTH AFRICA, Mpumalanga, Barberton, *E.E.Galpin* 430 (BOL, GRA, K!, PRE).
Convolvulus natalensis var. angustifolia C.H.Wright, Fl. Cap. (Harvey) 4(2): 77. 1904. (Wright 1904: 77). Type. SOUTH AFRICA, Mpumalanga, Barberton, *E.E.Galpin* 430 (holotype K!; isotypes BOL GRA, PRE).
Convolvulus natalensis var. transvaalensis (Schltr.) A.Meeuse, Bothalia 6: 689. 1958 [1957]. (Meeuse 1958: 689). Type. Based on *Convolvulus transvaalensis* Schltr.

##### Type.

SOUTH AFRICA, Cape. *Thunberg* s.n. (holotype BM000930466! ex Herb. Roemer; isotype UPS).

##### Description.

Perennial herb, thinly pubescent in all vegetative parts; rootstock thin, woody; stems to 70 cm, prostrate. Leaves petiolate, relatively small, lanceolate to ovate in outline, the central lobe 1– 5 × 0.3–0.8, cm, oblong to lanceolate, dentate, pinnatisect to pinnatifid, characteristically cordate-deltoid, auricles prominent, usually bifurcate, 0.3–1.5 cm, usually dentate, apex acute or obtuse; petioles 3–12 mm. Flowers solitary (rarely paired), axillary, pedunculate, peduncles 0.8–3 cm; bracteoles 3–7 mm, subulate to linear; pedicels 3–12 mm; outer sepals 9–12 × 4–6 mm, ovate, acute to acuminate, inner sepals scarioius, pubescent only in central vertical lines; corolla 1.6–2.8 cm, white or pink, very shallowly lobed, the midpetaline bands pubescent, terminating in a tooth; ovary glabrous; style glabrous, divided c. 7 mm above base; stigmas 4 mm, linear. Capsule glabrous; seeds smooth. [Meeuse 1958: 690; Meeuse and Welman 2000: 47 (map and plate)]

##### Distribution.

South Africa: Eastern Cape, KwaZulu-Natal, Free State, North West, Gauteng, Mpumalanga, Limpopo (*Moss* 7122, *Bolus* 6847, *Cooper* 790, *Meeuse* 9376, *Schlechter* 3479); Lesotho (*Dieterlen* 387).

##### Notes.

Distinguished by its essentially pinnately-nerved central leaf lobe. It seems close to *Convolvulus natalensis* particularly as represented by *Hoggarth in Wood* 4179, *Dietelen* 38751, *Galpin* 430 & *Williams* 154, which Meeuse (1958: 689) treated as var. transvaalensis of *Convolvulus natalensis* and is also close to some forms of *Convolvulus austrafricanus* differing in little more than the larger flowers. It has been much confused historically being also treated as a variety of *Convolvulus capensis* (Baker and Wright 1904).

#### 28. Convolvulus dregeanus

Taxon classificationPlantaeSolanalesConvolvulaceae

Choisy, Prodr. [A.P. de Candolle] 9: 411. 1845. (Choisy 1845: 411).

Figure 6, t. 23–31

Convolvulus liniformis Rendle, J. Bot. 39: 61. 1901. (Rendle 1901: 61). Type. SOUTH AFRICA, *Zeyher* 1220 (lectotype BM000930471!, designated here; isolectotype P!).

##### Type.

SOUTH AFRICA, Northern Cape, *Drège* 7828 (holotype G; isotypes BM!, L, P!).

##### Description.

Completely glabrous perennial herb with decumbent to ascending stems to 30 cm long from a central taproot. Leaves shortly petiolate, 1–2 (-3) cm long, very variable in form on the same plant and between plants, sometimes linear with a hastate base and minute auricles (*Zeyher* 1220), more commonly dimorphic, the basal leaves ovate, weakly cordate and apically obtuse with coarsely serrate margins, becoming pinnatifid upwards, the upper leaves 5-fid with a long, linear central lobe and shorter basal lobes (*Drège* 7828); petioles 1–5 mm (shorter in linear-leaved plants). Flowers solitary, pedunculate; peduncles 0.5–3 cm long, pedicels 1– 5 mm, bracteoles 1–2 mm, linear-lanceolate to spathulate; outer sepals 4–5.5(-7) × 2.5–3 mm, obovate, rounded, rounded and mucronate or fimbriate; corolla 1–1.4 (-2) cm, white to pale pink, midpetalline bands glabrous; ovary glabrous; style glabrous, divided 6–9 mm above base, stigmas 1.5 mm, slightly widened upwards. Capsule glabrous; seeds glabrous, rugose. [Meeuse 1958: 671; Meeuse and Welman 2000: 41 (map)]

##### Distribution.

South Africa except KwaZulu-Natal (*Gemmell* 4976, *Acocks* 20833, *Hutchinson* 3100, *Verdoorn* 899), Lesotho (*Christol* s.n. [1907-8]).

##### Notes.

Distinct for being completely glabrous with small, delicate leaves and short obovate, rounded to slightly fimbriate sepals.

#### 29. Convolvulus boedeckerianus

Taxon classificationPlantaeSolanalesConvolvulaceae

Peter, Nat. Pflanzenfam. [Engler & Prantl] 4(3a): 36. 1891. (Peter 1891: 36).

##### Type.

SOUTH AFRICA, Free State, *Boedecker* s.n. (lectotype GOET-002454, designated by Meeuse and Welman 2000: 40).

##### Description.

Perennial herb with woody taproot from which spread numerous stems to 60 cm, plant covered in adpressed brown to silvery hairs. Leaves 1–2.5 × 0.5–2 cm, lanceolate to ovate in outline, variable in form from pinnatisect to palmately 5-lobed, often with the terminal lobe much longer and deeply toothed and the basal lobes bifid, base truncate to shallowly cordate; petioles 1–5 mm long. Flowers solitary, axillary, pedicellate but not pedunculate (rarely peduncle to 1mm); bracteoles 1–2 mm long, subulate; pedicels 2–6(-10) mm, outer sepals 4–5(-6) × 2–3 mm, ovate to oblong-elliptic, acute; corolla 7–10 mm long, pink or white, shallowly lobed, midpetaline bands pubescent with brown hairs; ovary glabrous; style glabrous, divided 2.5 mm above base; stigmas 2.5 mm, slightly widened upwards. Capsule glabrous; seeds glabrous, smooth but muricate on angles. [Meeuse 1958: 674; Meeuse and Welman 2000: 40 (map)]

##### Distribution.

South Africa except KwaZulu-Natal (*Prosser* 1529, *Werger* 289, *Shaw* 123, *Brierley* 173, *Flanagan* 2112, *Duparquet* 107).

##### Notes.

Distinguished by the solitary, pedicellate flowers and near absence of peduncles combined with the very small calyx, the sepals usually about 5 mm long and thinly covered in brownish hairs. The inflorescence is similar to that of *Convolvulus ocellatus* but in that species the calyx is >6 mm long and the whole plant is densely tomentose. It can be confused with *Convolvulus multifidus* but in *Convolvulus multifidus* the calyx is larger. It could also be confused with *Convolvulus austroafricanus* but that species usually has several flowers which are always borne on a peduncle.

There are specimens apparently intermediate with *Convolvulus austroafricanus* including *Moss* 4718 from Belmont, *Goosseno* 728 from Free State and *Eyres* 1820 and *Jacobsen* 1772 from Zimbabwe. These have short but very distinct peduncles 5–10 mm long which bear 1–2 flowers, similar in dimensions to *Convolvulus boedeckerianus*. Unlike *Convolvulus austroafricanus* these plants are not very hirsute. Given the increasing evidence for hybridisation within *Convolvulus* these specimens may represent plants of hybrid origin.

#### 30. Convolvulus multifidus

Taxon classificationPlantaeSolanalesConvolvulaceae

Thunb., Prodr. Pl. Cap. 35. 1794. (Thunberg 1794: 35).

##### Type.

SOUTH AFRICA, Eastern Cape, *Thunberg* s.n. (holotype UPS!).

##### Description.

Perennial herb similar in facies to *Convolvulus boedeckerianus*, densely villous to tomentose in all vegetative parts; rootstock a thickened, woody taproot; stems 15–75 cm long, prostrate. Leaves 0.5–2.5 × 0.5–1 cm, palmately lobed with the central lobe pinnatisect, more or less ovate in outline with weakly cordate base; petioles 3–8 mm. Flowers solitary, axillary, pedunculate; peduncles 0–8 mm, pedicels 8–15 mm; bracts linear 2–7 × 0.5 mm; outer sepals 6.5–9 × 5–mm, broadly elliptic, acute, villous, somewhat glabrescent towards the margins; corolla 10–13 mm, pale pink or white, deeply lobed, midpetaline bands pubescent with brownish hairs; ovary glabrous; style glabrous, stigmas 3.5–4 mm, linear. Capsule glabrous; seeds glabrous, smooth except for muricate angles. [Meeuse 1958: 675; Meeuse and Welman 2000: 43 (map)]

##### Distribution.

South Africa, almost endemic to the Cape (*Burchell* 1839, *Acocks* 21861, *Baur* 1020).

##### Notes.

Distinguished from *Convolvulus boedeckerianus* by the larger calyx and (usually) pedunculate flowers.

#### 31. Convolvulus argillicola

Taxon classificationPlantaeSolanalesConvolvulaceae

Pilg., Bot. Jahrb. Syst. 48: 348. 1912. (Pilger 1912: 348).

Figure 6, t. 32–40

##### Type.

NAMIBIA, *Dinter* 1892 & 2153 (syntypes B†, SAM).

##### Description.

Densely hispid-pilose perennial with prostrate/trailing stems from a central taproot to 70 cm; hairs rusty-brown in colour. Leaves petiolate, 1–3.5 (-5) × 0.5–2.5 cm, ovate in outline, deeply pinnatisect, abruptly narrowed and cuneate onto the petiole; petioles 1–8 mm. Flowers 1–2, axillary, subsessile; peduncles to c. 0.3 cm; pedicels 0; bracts filiform, 5–9 × 0.5 mm; outer sepals broadly ovate with a long caudate apex, c. 7–8 mm at anthesis, accrescent to 12–13 mm, becoming somewhat scarious, the margin crisped; corolla 10–12 mm long, deeply lobed for c. 4 mm, nearly concealed by calyx, white with pilose midpetaline bands; ovary glabrous, divided c. 4 mm above base; stigmas 2 mm, linear. Capsule glabrous; seeds glabrous, rugose. [Meeuse 1958: 670; Meeuse and Welman 2000: 37 (map)]

##### Distribution.

Namibia (*Seydel* 3695, 4170, *Merxmuller* 1032, *Pearson* 9562, *Dinter* 4284). 1500–2000 m. *Acacia* bushland on sand; apparently rare.

##### Notes.

Very distinct because of the subsessile flowers and accrescent calyx, which almost conceals the corolla.

#### 32. Convolvulus ocellatus

Taxon classificationPlantaeSolanalesConvolvulaceae

Hook., Bot. Mag. 70: t. 4065. 1844. (Hooker 1844: t.4065).

Figure 7, t. 7–14

Convolvulus ornatus Engl., Bot. Jahrb. Syst. 10: 247. 1888. (Engler 1888: 247). Type. SOUTH AFRICA, Northern Cape, *Marloth* 716 (holotype B†; isotype PRE).
Convolvulus ocellatus var. ornatus (Engl.) A.Meeuse, Bothalia 6: 673. 1958 [1957]. (Meeuse 1958: 673). Type. Based on *Convolvulus ornatus* Engl.
Convolvulus dinteri Pilger, Bot. Jahrb. Syst. 45: 219. 1910. (Pilger 1910: 219). Type. NAMIBIA, Kraaifontein, *Dinter* 812 (holotype B†; isotypes SAM, PRE, not seen).

##### Type.

Plate 4065 in Curtis, Botanical Magazine 70 (1844); epitype (designated here): SOUTH AFRICA, North West Province, Gauteng, Magaliesberg, *Burke* 119 ex Herb. Hooker (K!).

##### Description.

Perennial herb with all vegetative parts tomentose with brown or grey hairs; rootstock stout, woody; stems 20–100 cm long, decumbent and trailing to erect, occasionally apparently rambling over shrubs, often woody towards the base. Leaves subsessile or shortly petiolate, 1–2.5 × 0.1–1.5 cm, narrowly oblong with or without basal auricles to palmately 5-fid (var. ornatus), the central lobe much longer than the bifid basal lobes, margin characteristically revolute, petioles 0.5–3 mm. Flowers almost always solitary; peduncles 0–5 mm long; pedicels 3–11 mm; bracts 1–5 mm, linear; outer sepals 6–8 × 3–4, oblong-lanceolate, abruptly contracted above middle and then narrowed to an obtuse to subacute apex; corolla 12–14 mm long, pink or white, distinctly lobed, midpetaline bands pubescent with brown hairs; ovary pilose or glabrous; style thinly pilose or glabrous, divided c. 5 mm above base; stigmas 3.5–4 mm, linear; Capsule pilose at the apex; seeds smooth. [Meeuse 1958: 673 p. p.; Meeuse and Welman 2000: 45 (map)]

**Figure 7.**
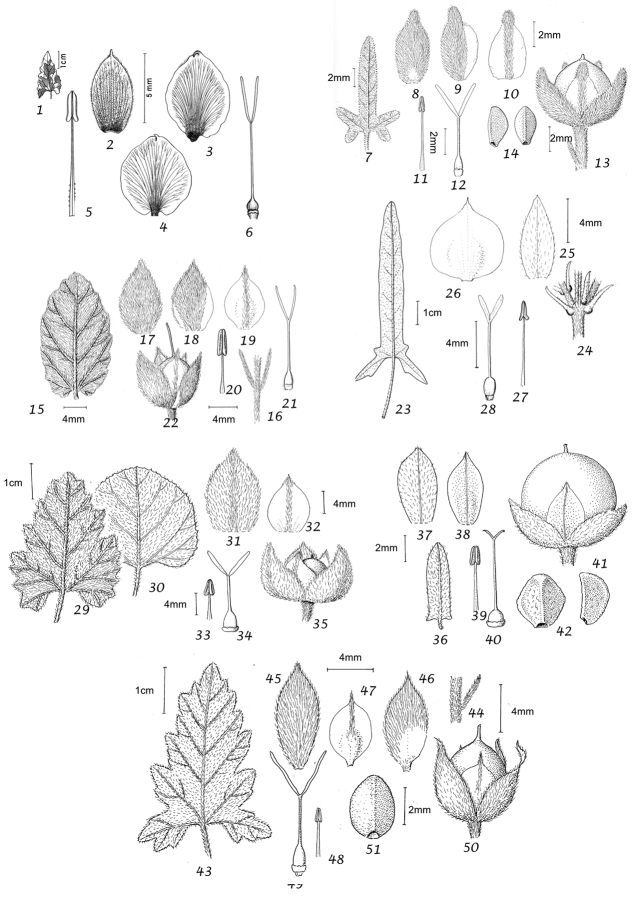
**1–6** *Convolvulus farinosus* **1** leaf **2** outer sepal **3** middle sepal **4** inner sepal **5** stamen **6** ovary and style. From *Voeke* 3803 (GOET) **7–14** *Convolvulus ocellatus* **7** leaf **8** outer sepal **9** middle sepal **10** inner sepal **11** stamen **12** ovary and style **13** bracteoles, calyx and capsule **14** seeds. From *Adams* 10/1920 (K) **15–22** *Convolvulus randii* **15** leaf **16** bracteoles **17** outer sepal **18** middle sepal **19** inner sepal **20** stamen **21** ovary and style **22** calyx and capsule. From *Leach and Muller* 11720 (K) **23–28** *Convolvulus aschersonii* **23** leaf **24** bracteoles showing flower buds **25** outer sepal **26** inner sepal **27** stamen **28** ovary and style. From *Moss* 6304 (BM) **29–35** *Convolvulus natalensis* **29** leaf **30** leaf **31** outer sepal **32** inner sepal **33** stamen **34** ovary and style **35** calyx and capsule **29 & 31–35** from *Hilliard* 5023 (K) **30** from *Drège* s.n. (OXF) **36–42** *Convolvulus sagittatus* **36** leaf **37** outer sepal **38** inner sepal **39** stamen **40** ovary and style **41** calyx and capsule **42** seeds. From *Wood* 3239 (BM) **43–51** *Convolvulus austroafricanus* **43** leaf **44** bracteole **45** outer sepal **46** middle sepal **47** inner sepal **48** stamen **49** ovary and style **50** calyx and capsule **51** seed. From *Noorgrann* 423 (K).

##### Distribution.

South Africa (*Burke* 119, *Burchell* 2412, *Leistner* 2036); Namibia (*Merxmuller & Giess* 1160, *Engler* 6243, *Wanntorp* 762); Botswana (*Skarpe* 201). Often in dry semi-desert conditions.

##### Notes.

Usually easily recognised by the densely tomentose indumentum combined with revolute leaf margins. The calyx and corolla are similar in size to that of *Convolvulus multifidus* but the sepals are abruptly contracted above the middle and then gradually narrowed to the apex.

#### 33. Convolvulus randii

Taxon classificationPlantaeSolanalesConvolvulaceae

Rendle, J. Bot. 40: 189. 1902. (Rendle 1902: 189).

Figure 7, t. 15–22

Convolvulus ocellatus var. plurinervius Verdc., Kirkia 1: 28, pl. 4. 1961. (Verdcourt 1961: 28). Type. ZIMBABWE, *Wild* 3926 (holotype K!; isotypes EA, SRGH).

##### Type.

ZIMBABWE, Gweru, *Rand* 274 (holotype BM000930474!).

##### Description.

Perennial herb, all vegetative parts covered in appressed sericeous hairs; rootstock woody, very stout, apparently horizontally spreading; stems erect or ascending, rarely rambling over shrubs, 20–80 cm high. Leaves shortly petiolate, 0.8–3 × 0.2–2 cm, oblong to obovate, apex acute to apiculate, margin entire to crenate, not revolute, base truncate to cordate, prominently veined especially on the lower surface; petioles 0.5–3 mm. Flowers solitary, axillary, pedunculate; peduncles (0.1-)0.3–2.5 cm; bracteoles 3–5 mm, linear; pedicels 2–10 mm; outer sepals 8–10 × 3–6 mm, broadly to narrowly ovate, tapered to an apiculate apex; corolla 16–20 mm long, white or pale pink, shallowly lobed, the midpetaline bands pubescent, terminating in teeth; ovary glabrous, finely acuminate; style glabrous, divided 5 mm above base, stigmas 5 mm, linear. Capsule glabrous seeds smooth.

##### Distribution.

Endemic to Zimbabwe, gowing in grassland on serpentine deposits, 1270–1700 m. (*Brummitt & Drummond* 15281, *Drummond* 6166, *Wild* 5594, *Chase* 7247).

##### Notes.

Somewhat variable in habit but readily recognised by the broad oblong-obovate leaves, silvery sericeous indumentum, acute sepals and larger corollas. *Walters* 2433 could be interpreted as a hybrid with *Convolvulus ocellatus* – it is geographically and morphologically intermediate.

#### 34. Convolvulus austroafricanus

Taxon classificationPlantaeSolanalesConvolvulaceae

J.R.I.Wood & R.W.Scotland sp. nov.

urn:lsid:ipni.org:names:77147660-1

Figures 2a and 7, t. 43–51

##### Diagnosis.

Affine Convolvulus farinosi L. et *Convolvulus aschersonii* Engler sed pilis asperis, longis instructis et lobis medianis foliorum inciso-dentatis distinctis.

*Convolvulus aschersonii* sensu Meeuse (1958: 677).

##### Type.

ZIMBABWE, Salisbury [Harare], a weed, 29 June 1927, *R.G. Young 18497* (holotype BM001035803!; isotype PRE).

##### Description.

Perennial herb, all vegetative parts pubescent with somewhat asperous, sometimes rufous hairs; rootstock a woody taproot; stems prostrate or twining, up to 2 m long. Leaves petiolate, 3–6 × 0.5–2.5 cm, variable in shape, ovate-deltoid, auriculate, sometimes the auricles lobed, the central lobe commonly oblong, apex acute, the margins undulate, sinuate-dentate to pinnatisect, base hastate; petioles 3–30 mm. Flowers 1–6 together (very rarely all solitary on the same plant) in axillary pedunculate cymes; peduncles 10–35 mm; pedicels 2–15 mm, bracts 2.5–4 mm, linear; sepals very unequal, outer sepals 6–8 × 4–5 mm, ovate to elliptic, acute; inner sepals 4–6 × 3–4 mm, nearly glabrous, apiculate; corolla 9–12 mm long, white or pale pink, lobed, the midpetaline bands pubescent, terminating in prominent teeth; ovary glabrous; style glabrous, divided c. 3 mm above base, stigmas 2.5 mm, linear. Capsule glabrous; seeds glabrous, smooth. [Meeuse and Welman 2000: 38 (map), under *Convolvulus aschersonii*]

##### Distribution.

South Africa (*Codd* 8732, *Meeuse* 2237, *Hutchinson* 2895, *Codd* 8732); Zimbabwe (*Blenkison in Moss* 14811, *Peter* 51118, *Drummond* 4904, *Leach* 8369); Zambia (*Fanshawe* 6566); Ethiopia (*Mooney* 5548). It is centred on Northern South Africa-Zimbabwe and is absent north of southern Zambia apart from two collections from Ethiopia.

##### Notes.

This species was treated as *Convolvulus aschersonii* by Meeuse (1958) and Meeuse and Welman (2000) but is very different from the type of that species. It is distinguished from all similar species by the spreading pubescent, slightly asperous indumentum of stem, leaves and flower parts, the pinnatisect leaves and (usually) the 4–6-flowered cymes. Towards the north of its range it tends to have fewer flowers and specimens intermediate with *Convolvulus thomsonii* and *Convolvulus aschersonii* are sometimes found. Although quite often united with *Convolvulus sagittatus* molecular studies (Williams et al. 2014) support of the retention of *Convolvulus austroafricanus* as a distinct species.

*Convolvulus austroafricanus* is common in the area where Zimbabwe and South Africa meet and should be classified as Least Concen (LC) using IUCN (2012) guidelines. The epithet “*austroafricanus*” meaning southern Africa refers to its distribution.

A cultivated plant (*Meeuse* 9237A) looks very like a hybrid between *Convolvulus austroafricanus* and *Convolvulus farinosus*.

#### 35. Convolvulus aschersonii

Taxon classificationPlantaeSolanalesConvolvulaceae

Engl., Abh. Königl. Akad. Wiss. Berlin 2: 349. 1892. (Engler 1892: 349).

Figure 7, t. 23–28

Convolvulus hastatus var. multiflorus Choisy, Prodr. [A.P. de Candolle] 9: 407. 1845. (Choisy 1845: 407). Type. SOUTH AFRICA, Northern Cape, *Drège* 7829 (lectotype L, designated here; isolectotype P!).
Convolvulus ulosepalus Hallier f., Bot. Jahrb. Syst. 18: 103. 1893 [“1894”]. (Hallier 1893: 103). Type. SOUTH AFRICA, Northern Cape, *Drège* 7829 (lectotype L, designated by Meeuse and Welman 2000: 46); isolectotype P!).
Convolvulus rhynchophyllus Baker & Engl. ex Hallier f., Bull. Herb. Boissier 6: 534. 1898. (Hallier 1898a: 534). Type. *Bolus* 252 (K, lectotype, designated here).
Convolvulus sagittatus subvar. linearifolius Hallier f., Bull. Herb. Boissier 6: 534. 1898, as “Convolvulus sagittatus var. grandiflorus subvar. linearifolius”. (Hallier 1898a: 534). Type. SOUTH AFRICA, Mpumalanga, Barberton, *Galpin* 1037 (isotypes K!).
Convolvulus sagittatus var. linearifolius (Hallier f.) Baker & C.H.Wright, Fl. Cap. (Harvey et al.) 4(2): 72. 1904. (Baker and Wright 1904: 72). Type. Based on Convolvulus sagittatus subvar. linearifolius Hallier f.
Convolvulus sagittatus var. ulosepalus (Hallier f.) Verdcourt, Kew Bull. 12:346. 1957. (Verdcourt 1957: 346). Type. Based on *Convolvulus ulosepalus* Hallier f.

##### Type.

ETHIOPIA, *Schimper* 660 (holotype B†; isotypes BM001011617!, E005-7479!).

##### Description.

Perennial herb, all vegetative parts similarly obscurely puberulent to pubescent; rootstock a woody taproot; stems prostrate or twining, up to 2 m long. Leaves petiolate, (0.5-) 2–10 × 0.2–4 cm, variable in shape, narrowly deltoid in outline, auriculate with the basal auricles simple or, more commonly bifurcate, the central lobe oblong to oblong-lanceolate, much longer than the auricles, apex acute to apiculate, margin entire to undulate, base commonly more or less truncate and briefly cuneate onto the petiole, leaves near base of stem often with a broader central lobe than those near apex; petioles 5–25 mm. Flowers (1-) 2–6 together (very rarely solitary) in compact axillary, pedunculate cymes; peduncles 8–35 mm; bracteoles 2–5 mm, linear or linear-lanceolate; pedicels 1–10 mm, outer sepals 5–6 (-7) × 2–3 mm, lanceolate to ovate, acute, usually pubescent, inner sepals up to 5 mm wide, suborbicular, apiculate, margins scarious, glabrous or pubescent on the midrib only; corolla 7–12 mm long, white or pink, lobed, the midpetaline bands pubescent, terminating in prominent teeth; ovary glabrous; style glabrous, divided 3–4 mm above the base; stigmas c. 3 mm. Capsule glabrous; seeds glabrous, smooth. [Collenette 1999: 232 (as *Convolvulus sagittatus*); Meeuse 1957: 678 as *Convolvulus ulosepalus*; Meuse and Welman 2000: 436. p. p.; Verdcourt 1963: 44 p. p.; Sebsebe 2006 185 as Convolvulus sagittatus var. aschersonii]

##### Distribution.

South Africa (*Baur* 901, *Bolus* 252, *Tyson* 124, *Moss* 14129); Namibia (*Merxmuller* 813, *Wanntorp* 815); Lesotho (*Dinter* 144, *Dieterlen* 97); Botswana (*de Winter* 7403, *Brown* 7952); Mozambique (*Macuácua* 1333); Madagascar (*White* s.n. [16/9/1929]), *Baron* 5213); Angola (*Welwitsch* 6204); Zimbabwe (*Rand* 510, *Chubb* 375); Zambia (*Fanshawe* 5519, *Sanane* 307); Malawi (*Patel & Kwatha* 2708); Democratic Republic of the Congo (*Symoens* 13595); Ruanda (*Troupin* 4802); Tanzania (*Grimshaw* 93463, *Richards* 26827); Kenya (*Mearns* 1157, *Lugard* 168); Uganda (*Scott Elliot* 1145); Somalia (*Thulin* 10918); Ethiopia (*Schimper* 1130, *Hildebrandt* 498, *Scott* 305, *Bidgood et al.* 4970, *Cufodontis* 47); Eritrea (*Schweinfurth & Riva* 1061, *Schweinfurth* 1739, *Ryding* 1116); Yemen (*Spellenberg* 5426); Saudi Arabia (*Collenette* 5367); Nigeria (*Lely* 362).

##### Notes.

The type of *Convolvulus aschersonii* (*Schimper* 660) has leaves with a relatively broad central lobe 5–10 mm wide and this is matched in South African material (*Dieterlen* 97a has even wider leaves) but narrower lobes are much more common in southern Africa. Plants towards the northern end of the range have few-flowered cymes, quite frequently reduced to single flowers. They are often more strongly pubescent and with less pronounced, often simple basal auricles whereas bifurcate auricles are the norm further south. Examples of this northern form include *Simwanda* 108 from Zimbabwe, *Robinson* 8 from Zambia, *Symoens* 13595 from Congo, *Eggeling* 2593 from Uganda, *Bally* 5592 from Kenya, *Newbould* 774 from Somalia, *Scott Jones* 32 from Eritrea, *Wood* 3281 from Yemen and *Collenette* 5367 from Saudi Arabia,

*Convolvulus aschersonii* is most readily distinguished from *Convolvulus austroafricanus* by the leaf shape and the short pubescence. The central leaf lobe is entire and often very narrow, most notably in specimens from Namibia. Possible hybrids or intermediates with *Convolvulus austroafricanus* with strongly sinuate leaf lobes occur quite frequently in Transvaal but are hardly known elsewhere. (*Hanekom* 2528, *Meeuse* 9020, *Frieberberg* 3195, *Wilms* 983, *Mogg* 12299, *Acocks* 2169, *Marais* 36). From *Convolvulus sagittatus* and *Convolvulus thomsonii* it is distinguished by the usually 2–5-flowered peduncles, bifurcate auricles and corolla less than twice as long as the calyx, rarely exceeding 12 mm in length. Intermediates or possibly hybrids with *Convolvulus sagittatus* also occur in South Africa, (*Baur* 350, *Galpin* 1037, *Wilms* 2158, *Meeuse* 10253, *Pillans* 5605), Zimbabwe (*Eyles* 8473) and Zambia (*Best* 107). These have larger flowers than typical of *Convolvulus aschersonii* but the peduncles are 2-flowered and the leaves like those of *Convolvulus aschersonii*. These were, at least mostly, treated as “Convolvulus sagittatus subsp. grandiflorus var. linearifolius” by Meeuse (1958: 683).

#### 36. Convolvulus farinosus

Taxon classificationPlantaeSolanalesConvolvulaceae

L., Mant. Pl. 2: 203. 1771 (Linnaeus 1771: 203).

Figure 7, t. 1–6

Convolvulus cordifolius Thunb., Prodr. Pl. Cap. 35. 1794. (Thunberg 1794: 35). Type. SOUTH AFRICA, Cape, *Thunberg* (holotype UPS, not seen).
Convolvulus quinqueflorus Vahl., Symb. Bot. 3: 31. 1794 (Vahl 1794: 31). Type. *Bourbon* ex Herb. Thouin (holotype C10009603!).
Convolvulus micranthus Willd. ex Spreng., Syst. Veg. 1: 601. 1824, nom. illeg., non *Convolvulus micranthus* Roem. & Schult. (1819). (Sprengel 1824: 601). Type. Of unknown origin, sin col. (holotype B-W 03636-010).
Convolvulus sprengelii Choisy, Prodr. [A.P. de Candolle] 9: 416. 1845. (Choisy 1845: 416). Type. Based on *Convolvulus micranthus* Willd. ex Spreng.
Convolvulus penicillatus A. Rich., Tent. Fl. Abyss. 2: 74. 1851. (Richard 1851: 74). Type. ETHIOPIA, *Quartin Dillon & Petit* (holotype P-04067180!).
Convolvulus schweinfurthii Engl., Abh. Königl. Akad. Wiss. Berlin 2: 348. 1892. (Engler 1892: 350). Type. ETHIOPIA, Anedehr, *Schimper* 599 (holotype B†; isotype BM001035801!).
Convolvulus sagittatus subvar. abyssinicus Hallier f., Bull. Herb. Boissier 6: 533. 1898. (Hallier 1898a: 534). Type. Based on *Convolvulus penicillatus* A.Rich.
Convolvulus hilsenbergianus Rendle, J. Bot. 39: 61. 1901. (Rendle 1901: 61). Type. MADAGASCAR, *Hilsenberg & Bojer* s.n. (lectotype BM-000930463!, designated here).
Convolvulus sagittatus var. abyssinicus (Hallier f.) Baker & Rendle, Fl. Trop. Africa (Oliver et al.) 4(2): 96. 1905. (Baker and Rendle 1905: 96). Type. Based on Convolvulus sagittatus subvar. abyssinicus Hallier f.
Convolvulus variegatus Sa’ad, Meded. Bot. Mus.Herb. Rijks Univ. Utrecht 281: 246 1967. (Sa’ad 1967: 246). Type. Cultivated plant, *Vocke* 3803 (holotype GOET!).

##### Type.

Cultivated plant grown at Uppsala (lectotype LINN 218.6!, designated by Meeuse 1958: 684).

##### Description.

Perennial herb, appressed pubescent to farinose in all vegetative parts, especially the younger stems; rootstock not known; stems to c. 1 m, twining or prostrate. Leaves petiolate, 3–9 × 2–6 cm, characteristically cordate-deltoid, auricles usually acute, apex acute to acuminate, margin entire, undulate or serrate; petioles 1–4.5 cm. Flowers 1–6 in axillary pedunculate cymes, peduncles 1.5–5 cm; bracteoles 1–2 mm, subulate; pedicels 1–15 mm; outer sepals 6–8 × 3–5 mm, lanceolate, ovate or elliptic, acute the apex often slightly reflexed, pubescent, inner sepals suborbicular with scarious margins, glabrous; corolla 10–15 mm, white or pinkish, lobed, the midpetaline bands pubescent; ovary glabrous; style glabrous, divided 4 mm above base, stigmas 1–1.5 mm, linear. Capsule glabrous; seeds glabrous, rugose. [Sa’ad 1967: 225; Meeuse 1958: 684 (map); Meeuse and Welman 2000: 42; Gonçalves 1987: 28–30 (plate); Verdcourt 1963: 41; Silvestre 2012: 257]

##### Distribution.

South Africa (*Salter* 9401, *Moss* 14480, *Schlechter* 2132); Swaziland; Madagascar (*Bosser* 12002); Reunion (*Bosser* 21493); Mozambique (*Nuvunga & Boane* 296, *Junod* 423); Zimbabwe (*Chase* 5314); Zambia (*Hutchinson & Gillett* 3387); Malawi (*Pawek* 13136); Congo (*Cambridge Congo Exped.* 18); Ruanda (*Michel* 4893); Tanzania (*Bidgood et al.* 548, *Schlieben* 881); Kenya (*Fries & Fries* 198); Uganda (*Purseglove* 3709); Ethiopia (*Schimper* 599); Eritrea (?), Yemen (*Wood* 2990, 3192). Naturalised in Portugal (*Welwitsch* 805), the Azores and also apparently in Mexico (*Bourgeau* 362 at K, P, *Argüelles* 2000 at NY).

##### Notes.

Usually readily recognised by the triangular-ovate, shortly pubescent to farinose, very acute leaves and small, deeply lobed corolla. However, occasionally plants are seen in which the leaves are ovate or sinuately lobed to more or less palmatisect, particularly in South Africa (*Meeuse* 9035, *Moss* 9855) and these are best distinguished from *Convolvulus austroafricanus* by the appressed pubescent indumentum. Occasional specimens suggest possible hybridisation with *Convolvulus aschersonii*, such as *Archbold* 2546 from Tanzania or *Pawek* 11875 from Malawi. As the only real difference between the two species lies in the leaf shape any intermediate specimen could be the result of hybridisation.

#### 37. Convolvulus sagittatus

Taxon classificationPlantaeSolanalesConvolvulaceae

Thunberg, Prodr. Pl. Cap. 35. 1794. (1794: 35).

Figure 7, t. 36–42

Convolvulus paradoxus Poir., Encycl. Suppl. [J. Larmarck et al.] 5(2): 720. 1817. (Poiret 1817: 720). Type. Not found at P.
Convolvulus steudneri Engl., Abh. Königl. Akad. Wiss. Berlin 2: 350. 1892. (Engler 1892: 350). Types: ETHIOPIA, Tauta bei Magdala, *Steudner* 956 (syntype B†) and Talenta, *Rohfls* s.n (syntype B†); ETHIOPIA, Sennen, *Schimper* 165 (neotype W!, designated here; isoneotype K).
Convolvulus angolensis Baker, Bull. Misc. Inform. Kew 1894: 67. 1894. (Baker 1894: 67). Type. ANGOLA, Cuenza, *H.H.Johnson* (holotype K!).
Ipomoea huillensis Baker, Bull. Misc. Inform. Kew 1894: 70. 1894. (Baker 1894: 70). Type. ANGOLA, Huilla, *Welwitsch* 6131 (holotype BM001035800!; isotypes COI, G, K!, P!).
Convolvulus sagittatus var. parviflorus Hallier f., Bull. Herb. Boissier 6: 533. 1898 (Hallier 1898a: 533). Type. No specimens cited; based on subvarieties.
Convolvulus sagittatus subvar. australis Hallier f., Bull. Herb. Boissier 6: 533. 1898, illegitimate name for autonymic subvariety (Hallier 1898a: 533).
Convolvulus sagittatus var. grandiflorus Hallier f., Bull. Herb. Boissier 6: 534. 1898. (Hallier 1898a: 534). Type. No specimens cited; based on subvarieties.
Convolvulus sagittatus subvar. subcordata Hallier f., Bull. Herb. Boissier 6: 534. 1898, “Convolvulus sagittatus var. grandiflorus subvar. subcordata” (Hallier 1898a: 534).nom. illeg. for autonymic subvariety. (Hallier 1898a: 533). Type. Based on *Convolvulus steudneri* Engl., *Convolvulus angolensis* Baker and *Ipomoea huillensis* Baker.
Convolvulus sagittatus subvar. graminifolia Hallier f., Bull. Herb. Boissier 6: 534. 1898, as “Convolvulus sagittatus var. grandiflorus subvar. graminifolia” (Hallier 1898a: 534). Type. SOUTH AFRICA, KwaZulu-Natal, *Rehmann* 7823 (holotype Z, not seen).
Convolvulus phyllosepalus Hallier f., Bull. Herb. Boissier 6: 535. 1898. (Hallier 1898a: 535). Type. SOUTH AFRICA, *Rehman* 3796 (lectotype Z, designated by Meeuse 1957: 681).
Convolvulus hirtellus Hallier f., Bull. Herb. Boissier 6: 536. 1898. (Hallier 1898a: 536). Type. SOUTH AFRICA, *Burke* s.n. (lectotype K!, designated by Meeuse 1957: 681).
Convolvulus hastatus Thunb. var. natalensis Baker in Baker & C.H.Wright, Fl. Cap. (Harvey et al.) 4(2): 72. 1904. (Baker and Wright 1904: 72). Type. SOUTH AFRICA, KwaZulu-Natal, *Gerrard* 1333 (lectotype K 000097310, portion at top of sheet, designated here).
Convolvulus sagittatus var. graminifolius (Hallier f.) Baker & C.H.Wright, Fl. Cap. (Harvey et al.) 4(2): 72. 1904. (Baker and Wright 1904: 72). Type. Based on Convolvulus sagittatus subvar. graminifolia Hallier f.
Convolvulus sagittatus var. latifolius C.H.Wright in Baker & Wright, Fl. Cap. (Harvey et al.) 4(2): 72. 1904. (Baker and Wright 1904: 72). Type. SOUTH AFRICA, Transvaal, October (18)76, *E. Holub* 1948-1951 (lectotype K, designated here). This appears to be a single sheet, rather than four separate numbers.
Convolvulus huillensis (Baker) Rendle, Fl. Trop. Africa [Oliver et al.] 4(2): 97. 1905. (Baker and Rendle 1905: 97). Type. Based on *Ipomoea huillensis* Baker
Convolvulus sagittatus var. subcordata (Hallier f.) Baker & Rendle, Fl. Trop. Africa [Oliver et al.] 4(2): 97. 1905, nom. illeg., autonymic variety based on “Convolvulus sagittatus var. grandiflorus subvar. subcordata Hallier f.” (Baker and Rendle 1905: 97). Type. Based on Convolvulus sagittatus subvar. subcordata Hallier f.
Convolvulus thymoides Schwartz, Mitt. Inst. Allg. Bot. Hamburg 10: 202. 1939. (Schwartz 1939: 202). Type. YEMEN, *Von Wissmann* 2097 (lectotype HBG!, designated here).
Convolvulus sagittatus var. phyllosepalus (Hallier f.) A.Meeuse, Bothalia 6: 681. 1958 [1957], as “Convolvulus sagittatus subsp. sagittatus var. phyllosepalus” (Meuse 1958: 681). Type. Based on *Convolvulus phyllosepalus* Hallier f.
Convolvulus sagittatus var. hirtellu s (Hallier f.) A.Meeuse, Bothalia 6: 682. 1958 [1957], as “Convolvulus sagittatus subsp. sagittatus var. hirtellus” (Meuse 1958: 682). Type. Based on *Convolvulus hirtellus* Hallier f.
Convolvulus sagittatus subsp. grandiflorus (Hallier f.) A.Meeuse, Bothalia 6: 683. 1958 [1957]. (Meuse 1958: 683). Type. Based on Convolvulus sagittatus var. grandiflorus Hallier f.

##### Type.

SOUTH AFRICA, Cape, *Thunberg* s.n. (lectotype UPS, sheet 1, designated by Meeuse 1958: 679).

##### Description.

Very variable perennial herb, the vegetative parts usually thinly to densely pubescent, very rarely glabrous; stems decumbent, trailing, rambling or ascending usually < 60 cm long; rootstock a stout taproot. Leaves petiolate, sometimes dimorphic with ovate-deltoid (below) and narrowly lanceolate leaves on the same plant, 1–2.8 (-5.5) × (0.1-) 0.3–1.4 cm, ovate-deltoid to narrowly lanceolate, apex acute, margin entire (very rarely undulate), base sagittate or hastate, the basal auricles not bifid, varying greatly in width; petioles 2–4 (-7) mm. Flowers axillary, pedunculate, 1(-2); peduncles (3-) 6–33 mm; bracteoles 2–3 mm, linear to linear-lanceolate; pedicels 2–5 (-12) mm, outer sepals 5.5–8 × 4–5 mm, ovate, broadly oblong to obovate, acute to obtuse, the apex often somewhat bent outwards, glabrous or pubescent, inner sepals glabrous; corolla (1-) 1.2–1.7 cm. pink or white, shallowly lobed, midpetaline bands pubescent, terminating in a tooth; ovary glabrous, style glabrous, divided 3–4 mm above base; stigmas 2–4 mm, linear. Capsule glabrous; seeds glabrous, minutely rugose. [Meeuse 1958: 679 p. p.; Meeuse and Welman 2000: 46 p. p.; Sebsebe 2007: 184 as *Convolvulus steudneri*]

##### Distribution.

South Africa (*Moss* 10572, *Schlechter* 3362, 3484, *Bolus* 10905, *Burtt-Davy* 2316, *Bester* 4286, *Gerard* 1333); Lesotho (*Dieterlen* 97b p. p.); Botswana (*Baum* 180); Namibia (?); Zimbabwe (*Gilliland* 1900, *Wild* 4917, *Whellan & Davis* 988); Angola (*Pritchard* 310, Santos 577); Ethiopia (*Schimper* 169, *Hall* 128, *Degen* s.n. [4/1902], *Mooney* 4725); Yemen (*Wood* 75/381, 3239, 3245); Saudi Arabia (*Hillcoat* 56); Algeria: Hoggar (fide Quezel and Santa 1963: 758). The lack of records from East Africa seems to reflect a real absence. It is more obviously montane in Ethiopia and SW Arabia.

##### Notes.

Distinguished from related species by the solitary pedunculate flowers, the corolla typically 1.2–1.7 cm long. The leaves are essentially ovate-deltoid, becoming linear in some cases, although often very narrowly so and the basal auricles are simple. The petioles are often very short.

The type of *Convolvulus sagittatus* has narrowly lanceolate leaves, whereas the type of *Convolvulus phyllosepalus* has broadly ovate leaves with very short petioles and conspicuous broad sepals. However, as noted above, both leaf forms can occur on the same specimen.

Although our concept of *Convolvulus sagittatus* is narrower than that of Meeuse and Welman (2000) or of Verdcourt (1963), it still represents an aggregate, which certainly contains distinct varieties and possibly distinct species. All the four cited specimens from Angola have ovate, acute to acuminate outer sepals and could be recognised as *Convolvulus angolensis*. Plants from Ethiopia and SW Arabia were separated off by Wood (1997) as *Convolvulus thymoides* and by Sebsebe (2006) as *Convolvulus steudneri* and could be recognised under the latter name. They have broadly oblong, obtuse, pubescent outer sepals but they are scarcely distinct from some forms of *Convolvulus sagittatus* found in South Africa including the lectotype of *Convolvulus hirtellus*. The syntypes of *Convolvulus steudneri* in Berlin were destroyed. No isotype is known so we have designated *Schimper* 165 (W) as a neotype. This was identified as *Convolvulus steudneri* by Hallier in December 1892, comes from the correct part of Ethiopia and may well be distributed elsewhere, as are many of Schimper’s collections. Another distinct form with very narrow, hirsute leaves is represented by *Eyles* 8473 from Zimbabwe and *Baum* 180 from Botswana. *Santos* 554 from Angola has unusual oblong leaves. All these have relatively large solitary flowers and it seems best to retain them in *Convolvulus sagittatus* until more detailed study can clarify their status.

#### 38. Convolvulus thomsonii

Taxon classificationPlantaeSolanalesConvolvulaceae

Baker, Bull Misc. Inform. Kew 1894: 67. 1894. (Baker 1894: 67).

Convolvulus sagittatus subvar. villosus Hallier f., Bull. Herb. Boissier 6: 533. 1898, as “Convolvulus sagittatus var. parviflorus Hallier f. subvar. villosus Hallier f.” (Hallier 1898a: 533). Type. Based on *Convolvulus thomsonii* Baker
Convolvulus sagittatus var. villosus (Hallier f.) Rendle, Fl. Trop. Africa (Oliver et al.) 4(2): 96. 1905. (Baker and Rendle 1905: 96). Type. Based on Convolvulus subvar. villosus Hallier f.
Convolvulus bussei Pilg., Bot. Jahrb. Syst. 41: 295. 1908. (Pilger 1908: 295). Type. TANZANIA, Songea District, *Busse* 938 (holotype B†; isotype EA).
Convolvulus hallierianus Schulze-Menz, Notizbl. Bot. Gart. Berlin-Dahlem 14: 377. 1939. (Schulze-Menz 1939: 377). Type. TANZANIA, Matengo hills, *Zerny* 17 (holotype W!).
Convolvulus zernyi Schulze-Menz, Notizbl. Bot. Gart. Berlin-Dahlem 14: 377. 1939. (Schulze-Menz 1939: 377). Type. TANZANIA, Matengo hills, *Zerny* 370 (holotype W!).

##### Type.

TANZANIA, N. of Lake Nyasa, *Thomson* s.n. (holotype K!).

##### Description.

Prostrate perennial herb, densely tomentose on all vegetative parts, often brownish when dry. Leaves petiolate, 1.2–5 × 0.5–2.5 cm, variable in shape, ovate, lanceolate-deltoid or, most commonly oblong, margin undulate to crenate, base hastate to sagittate; petioles 3–6 (-20) mm. Flowers solitary, axillary, pedunculate; peduncles solitary or, occasionally, paired, 1.5–4 cm, often arching; bracteoles 5–7 mm, linear; pedicels 3–8 (-15) mm; outer sepals 9–11 × 5 mm, ovate, acute to shortly acuminate, densely hairy; corolla (1.3-)1.5–1.8 cm long, white, unlobed, midpetaline bands pubescent, terminating in a tooth; ovary glabrous. Capsule glabrous; seeds glabrous, nearly smooth but somewhat rugose on the angles. [Verdcourt 1963: 42 (as *Convolvulus zernyi*)]

##### Distribution.

Malawi (*Synge* WC 251, *Pawek* 13111, *Richards* 20646); Tanzania (*Lovett et al.* 2089, *Mgaza* 121). 1800–2100 m.

##### Notes.

We have widened the concept of this species from that of Verdcourt (1963) to include plants with a somewhat smaller corolla and calyx, including the type of *Convolvulus thomsonii*, which is atypical in its relatively short calyx and corolla but is clearly an immature specimen of this taxon. This species appears to be frequent in the highlands of northern Malawi and southern Tanzania. It is very close to *Convolvulus sagittatus* but differs in the larger sepals, 9–11 mm in length and the tomentose indumentum, which dries brown. The rather long, often arching peduncles are also very characteristic. An unusual feature is the occasional presence of paired peduncles. It can be distinguished from *Convolvulus aschersonii* and *Convolvulus austroafricanus* by the larger solitary flowers as well as the tomentose indumentum. It is also close to *Convolvulus galpinii* differing principally in the longer, gradually narrowed sepals.

As with many other African species, intermediates with other species are found. These have the same indumentum, relatively large corollas and sepals of *Convolvulus thomsonii* but inflorescences of 2–3 flowers. *Phillips* 2738 and 3920 from northern Malawi and *Richards* 6071 from Zambia appear to be intermediate with *Convolvulus austroafricanus* or possibly *Convolvulus aschersonii*.

#### 39. Convolvulus galpinii

Taxon classificationPlantaeSolanalesConvolvulaceae

C.H.Wright, Fl. Cap. (Harvey) 4(2): 75. 1904. (Baker and Wright 1904: 75).

##### Type.

SOUTH AFRICA, Eastern Cape, *E.E.Galpin* 2110 (holotype K!; isotypes BOL, GRA, PRE).

##### Description.

Perennial herb, densely brownish or whitish villous in all vegetative parts; rootstock not known; stems to 60 cm, slender, twining or (?) prostrate. Leaves petiolate, 2–4 × 0.7–1.2 cm, deltoid with cordate, hastate or sagittate base, apex acute, margin undulate or crenate; petioles 4–7 (-12) mm. Flowers axillary, pedunculate, solitary or paired, peduncles 1.5–2 (-6) cm; bracteoles 6–7 mm, linear; pedicels 3–6 (-10) mm; outer sepals 8 × 4 mm, ovate, abruptly narrowed above the middle to an acuminate apex; corolla 16–21 mm long, white, shallowly lobed, the midpetaline bands densely pilose, terminating in a tooth; ovary glabrous; style glabrous, divided 8–10 mm above the base, stigmas 2.5 mm linear. Capsule glabrous; seeds glabrous, obscurely rugose with pallid ridges, not puberulous as stated by Meeuse (1957: 687). [Meeuse and Welman 2000: 43 (map)]

##### Distribution.

South Africa: Eastern Cape (*Phillipson* 1541, *Krook* 841).

##### Notes.

Distinguished by the relatively slender, twining stems, dense indumentum, hastate or sagittate leaves and abruptly acuminate sepals.

#### 40. Convolvulus natalensis

Taxon classificationPlantaeSolanalesConvolvulaceae

Bernh. ex Krauss, Flora 27(2): 829. 1844. (Krauss 1844: 829).

Figure 7, t. 29–35

Convolvulus calycinus Drège ex Choisy, Prodr. [A.P. de Candolle] 9: 408. 1845. (Choisy 1845: 408), nom. illeg., non *Convolvulus calycinus* Kunth (1818). Type. SOUTH AFRICA, Cape, *Drège* s.n. (isotypes K!, L, OXF!, MO!).
Convolvulus natalensis var. integrifolia C.H.Wright, Fl. Cap. (Harvey) 4(2): 77. 1904. (Baker and Wright 1904: 77). Type. LESOTHO, *Cooper* 929 (lectotype K 000405826!, designated here).

##### Type.

SOUTH AFRICA, KwaZulu-Natal, Pietmaritzburg, *Krauss* 465 (holotype B†; isotypes BM000930469!, BOL, W!).

##### Description.

Perennial herb, densely hirsute with brownish hairs in all vegetative parts; rootstock woody; stems to c. 1 m, apparently trailing (rarely climbing), relatively stout. Leaves petiolate, 1–6 × 0.8–4 cm, ovate-deltoid, simple, apex acute, margin undulate to irregularly dentate, base cordate; petioles 5–10 (-15) mm. Flowers 1–5; peduncles 2–6.5 cm; pedicels 3–6 (-15) mm; bracteoles 6–12 × 1–2 mm, linear, narrowly lanceolate or narrowly oblanceolate; outer sepals 14–18 × 7–9 mm, broadly to narrowly ovate, obtuse or acute, margin undulate or crenate; corolla 15–30 mm, white or cream, shallowly lobed, the lobes broadly triangular, acute, c. 5 mm long, the midpetaline bands densely pilose; ovary glabrous, acuminate; style glabrous, divided 5–8 mm above base, stigmas 6 mm, linear. Capsule glabrous; seeds tuberculate. [Meeuse 1957: 687; Meeuse and Welman 2000: 44 (map)]

##### Distribution.

South Africa: centred on KwaZula-Natal extending to Eastern Cape, Free State, Lesotho, Swaziland and Northern Province (*Hilliard* 5023, *Sanderson* 282, *Codd* 7655, *Wood* 3462, *Rudatis* 633, *Strey* 3460).

##### Notes.

Distinguished by its tomentose, entire, ovate to oblong, cordate hirsute leaves. Plants described as var. integrifolia are somewhat similar to *Convolvulus galpinii* in leaf shape but 2-3 flowers are borne on each peduncle and the corolla is similar to *Convolvulus natalensis* in size.

#### 41. Convolvulus bullerianus

Taxon classificationPlantaeSolanalesConvolvulaceae

Rendle, J. Bot. 39: 62. 1901. (Rendle 1901: 62).

##### Type.

SOUTH AFRICA, KwaZulu-Natal, Mooi River, *J. Medley-Wood [J.M. Wood*] 6206 (holotype BM000930467!; isotype PRE).

##### Description.

Perennial herb, shortly pilose with stiff spreading hairs in all vegetative parts; rootstock not known; stems to at least 80 cm, apparently trailing. Leaves petiolate, very narrowly hastate, the central lobe 2 -5 × 0.2–0.4 cm, linear-lanceolate, basal auricles 3 -4 mm long, bifid, apex acute to apiculate, margin entire; petioles 5–16 mm. Flowers axillary, pedunculate, 1 (–2), peduncles 3–5.5 cm; bracteoles 6–10 mm, linear; pedicels 3–8 mm, noticeably more densely hirsute than peduncles; outer sepals 14–18 × 8 mm, ovate, long acuminate, margin undulate, inner sepals distinctly shorter; corolla 25–30 mm, yellow-green, deeply lobed, the lobes triangular, acuminate, c. 10 mm long, the midpetaline bands densely pilose, terminating in the apex of the lobes; ovary glabrous, style glabrous, divided 6–10 mm above base, stigmas 3–5 mm.

##### Distribution.

South Africa: KwaZulu-Natal (*Wood* 4071, 4382; *Johnston* 191, 778) and Eastern Cape (*Bester* 1479).

##### Notes.

Included by Meeuse (1958) in *Convolvulus natalensis* but distinct in its leaves, profoundly lobed corolla and long acuminate sepals.

### Species 42–55. American species

Apart from two anomalous species (*Convolvulus simulans* and *Convolvulus hasslerianus*) all species are perennial trailing or twining herbs with distinctly petiolate leaves, the lamina with a hastate, truncate or sagittate base. Dimorphic leaves are mainly features of *Convolvulus equitans* and *Convolvulus chilensis*. Only Convolvulus hermanniae subsp. erosus has a hirsute ovary and capsule. Taxonomy is based much on sepal and corolla size, number of flowers in each cyme and on indumentum. *Convolvulus hasslerianus* is the only American species with a woody xylopodium being adapted to the cerrado biome. It has erect stems and subsessile leaves, the lamina abruptly narrowed at the base.

The first two species treated here, *Convolvulus equitans* and *Convolvulus carrii* are clearly closely related and share several unusual even unique characters: auriculate sepals, styles pubescent below stigmas, persistent in fruit and somewhat exserted. Curiously these characters are present in some but not all specimens of both species. However, the presence or absence of these characters does not correlate well with other characters and shows no obvious geographical patterning. While *Convolvulus carrii* is easy to identify, it represents a very local population and it is not impossible that similar distinct local populations may be revealed elsewhere within the range of *Convolvulus equitans* following intensive field studies.

The taxonomy of the American species is difficult as can be appreciated by the synonomies listed under many species, the same infraspecific entity being placed variously under different species. However, we believe that O’Donell (1957, 1959) had correctly assigned most specimens to the correct species.

#### 42. Convolvulus equitans

Taxon classificationPlantaeSolanalesConvolvulaceae

Benth., Pl. Hartw. 16. 1839. (Bentham 1839: 16).

##### Type.

MEXICO, León, *Hartweg* 98 (lectotype K-000613111!, portion placed diagonally across sheet ex Herb. Bentham with Bentham’s annotation, designated here; isolectotypes K ex Herb. Hooker K-000613113!, W!).

##### Description.

Pubescent perennial herb from a stout tap root; stems decumbent or trailing to at least 1 m. Leaf blade very variable in size and form, 1.5–4 (-6.5) × 1–2.5 cm, most commonly with a narrow linear-ligulate central lobe much longer than the small lobed or bifurcate auricles, sometimes palmatisect, sometimes broadly ovate-deltoid, auriculate, usually densely and finely pubescent, apex acute, base cordate, margin entire, undulate or (rarely) crenate-serrate; petioles 0.5–2.5 cm. Flowers 1–3 in pedunculate, axillary cymes; peduncles 1.5–9 cm; bracteoles 1.5–2.5 × 1 mm, linear-lanceolate; pedicels 2–9 (-17) mm; outer sepals 6–8(-12) mm, narrowly elliptic, truncate to auriculate at base, margin entire to crenate, apex truncate and mucronate to acute; corolla 1.4–1.8(-3.0) cm long, white, white with dark centre or pink, shallowly lobed; midpetaline bands pubescent, terminating in a mucro; filaments eglandular; ovary glabrous; style glabrous or pubescent just below the stigmas, somewhat persistent, divided 5–7 mm above base; stigmas 2 mm, weakly exserted. Capsule glabrous, seeds minutely rugose. [Turner 2009: 400 (maps); Carranza 2008: 8]

##### Notes.

A very variable plant in many respects. However the vast majority of specimens have small leaves with a narrow linear-ligulate central lobe and short bifurcate or otherwise lobed auricles. In most plants the outer sepals are abruptly narrowed to auriculate at the base, but in many specimens including the type, they are gradually narrowed to the base. Plants are usually densely pubescent. The recognition of the following varieties only account for some of the great variation seen in this species.

#### 42a. Convolvulus equitans var. equitans

Taxon classificationPlantaeSolanalesConvolvulaceae

Figure 8, t. 1–8

Convolvulus incanus auct. mult., non Vahl (1794).
Convolvulus hermannioides A.Gray, Syn. Fl. N. Amer. 2(1): 216. 1878. (Gray 1878: 216). Type. UNITED STATES OF AMERICA, Texas, no collection specified.

##### Distinguishing features.

Flowers relatively small; sepals 6 – 8 mm long; corolla 1.4–1.8(2.3) cm long, usually pink.

##### Distribution.

Mexico: north and central (*Palmer* 147, *Parry & Palmer* 629, *Pringle* 6635); United States: semi-desert states from Texas and Kansas west to Arizona and Colorado, California (?) (*Lindheimer* 470, *Correll* 15376, *Pringle* s. n. [20/5/1884], *Fendler* 661).

**Figure 8.**
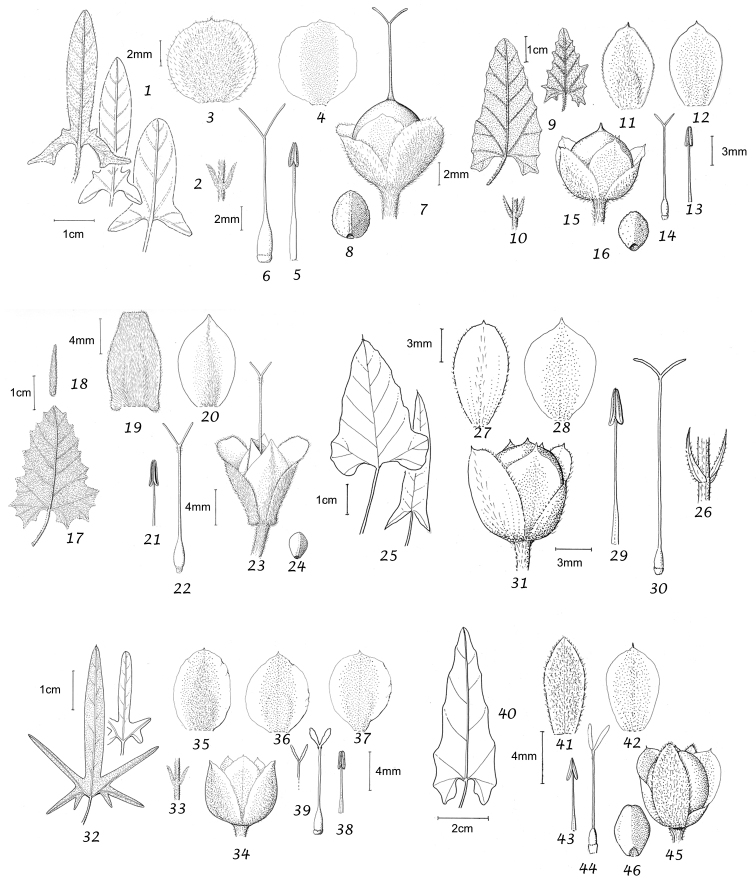
**1–8** *Convolvulus equitans* **1** leaves **2** bracteoles **3** outer sepal **4** inner sepal **5** stamen **6** ovary and style **7** capsule **8** seed **1–2 & 5–8** from *Correll* 27128 (TEX) **3–4** from *Turner* 21-787 (TEX) **9–16** *Convolvulus crenatifolius* **9** (North American) leaves **10** bracteole **11** outer sepal **12** inner sepal **13** stamen **14** ovary and style **15** capsule **16** seed **9–14** from *Runyon* 2599 (TEX) **15–16** from *Runyon* 4479 (TEX) **17–24** *Convolvulus carrii* **17** leaf **18** bracteole **19** outer sepal **20** inner sepal **21** stamen **22** style **23** calyx **24** seed. From *Correll & Correll* 38844 (TEX) **25–31** Convolvulus crenatifolius subsp. montevidensis **25** leaves **26** bracteole **27** outer sepal **28** inner sepal **29** stamen **30** ovary and style **31** capsule. From *Hawkes et al.* 3263 (K) **32–39** *Convolvulus chilensis* **32** leaves **33** bracteole **34** calyx **35** outer sepal **36** middle sepal **37** inner sepal **38** stamen **39** ovary and style showing stigma variation. From *Cuming* s.n. (OXF) **40–46** Convolvulus crenatifolius subsp. crenatifolius **40** leaf **41** outer sepal **42** inner sepal **43** stamen **44** ovary and style **45** capsule **46** seed **40–44** from *Buchtien* 2450 (K) **45–46** from *Wood* 17714 (K).

#### 42b. Convolvulus equitans var. lindheimeri

Taxon classificationPlantaeSolanalesConvolvulaceae

J.R.I.Wood & R.W.Scotland var. nov.

urn:lsid:ipni.org:names:77147665-1

##### Diagnosis.

A var, typo floribus grandioribus, sepalis 11 – 12 mm longis, corolla 2.5–3 cm longa, plerumque alba, in centro atropurpurea.

*Convolvulus sagittifolius* Scheele (1849: 747), nom illeg., non *Convolvulus sagittifolius* Michx. (1803).

**Type.** Texas, “Neubraunfels”, *Lindheimer* s.n. (B†).

##### Type.

UNITED STATES OF AMERICA, Texas, New Braunfels, *F. Lindheim* er, fasc. IV No. 469 (holotype K; isotypes BM, FHO, LE, P, W).

##### Distinguishing features.

Distinguished by it is larger flowers; sepals 11–12 mm long; corolla 2.5–3 cm long, usually white but often with a dark centre.

##### Distribution.

United States, Texas: widely distributed but not common in at least seven Texan counties (*Lindheimer* fasc. 3: 469, *Siedo* 447, *Logan Smith* 774, *Hutchins* 1035).

##### Notes.

Intermediates with var. equitans occur and var. lindheimeri may have arisen as a result of hybridisation between *Convolvulus carrii* and *Convolvulus equitans* although it is not sympatric with *Convolvulus carrii*.

#### 43. Convolvulus carrii

Taxon classificationPlantaeSolanalesConvolvulaceae

B.L.Turner, Phytologia 91: 394. 2009. (Turner 2009: 394).

Figure 8, t. 17–24

##### Type.

UNITED STATES OF AMERICA, Texas, *B.L. Turner & Jana Kos* 09-03 (holotype TEX; isotype OXF!).

##### Description.

Trailing or twining herb with stems at least 60 cm long from a central rootstock, the vegetative parts densely pubescent to whitish-tomentellous. Leaves petiolate, 2–5 × 1.3–3 cm, ovate-deltoid to broadly oblong, obtuse or acute, margin undulate to incised-dentate, base shallowly cordate and cuneate onto the petiole, auricles simples or toothed, veins very prominent on lower surface; petioles 1–2.5 cm. Flowers 1(-2) borne on long axillary peduncles; peduncles 3–5 cm, often bent at apex; bracteoles 1–2 mm, minute, linear-lanceolate; pedicels 3–14 mm; sepals 9–12 × 5 mm, broadly oblong, apex rounded to emarginate and mucronate, base truncate to somewhat auriculate; corolla 2.5–3 cm long, white, usually with a maroon centre, unlobed, midpetaline bands pilose, terminating in a tooth; ovary glabrous; style divided c. 8–10 mm above base, glabrous or, just below the stigmas, pubescent; stigmas 2 mm, weakly exserted. Capsule glabrous; seeds glabrous, smooth, black. [Turner 2009: 398–9, figs 1–3]

##### Distribution.

Endemic to Texas in the United States of America: restricted to Holocene sands in Brooks and Hidalgo Counties (*Carr* 26646, *Correll & Correll* 38844).

##### Notes.

A recently described species which requires further study. It may prove only to be an unusually distinct form of *Convolvulus equitans*.

#### 44. Convolvulus chilensis

Taxon classificationPlantaeSolanalesConvolvulaceae

Pers., Syn. Pl. 1: 180. 1805. (Persoon 1805: 180).

Figure 8, t. 32–39

Convolvulus dissectus Cav., Icon. 5: 54, tab. 480(1). 1799, nom. illeg., non *Convolvulus dissectus* Jacq. (1767). (Cavanilles 1799: 54). Type. CHILE, Chillán, *Née* s.n. (lectotype MA-475569!, sheet with corolla, designated here).
Convolvulus canescens Phil., Linnaea 33: 182. 1864. (Philippi 1864: 182). Type. CHILE, San Felipe de Aconcagua, *Landbeck* s.n. (?SGO).
Convolvulus dissectus var. canescens (Phil.) Reiche, Anales Univ. Chile 120: 828. 1907. (Reiche 1907: 828). Type. Based on *Convolvulus canescens* Phil.

##### Type.

Based on *Convolvulus dissectus* Cav.

##### Description.

Thinly to densely pubescent herb from a thick rootstock, sometimes sericeous on young parts, but more or less glabrescent; stems trailing (rarely twining), up to 2.5 m long. Leaves petiolate, 2–8 × 2–6 cm, very variable in form, usually linear or oblong with prominent elongate bifurcate basal auricles, but occasionally ovate-deltoid to suborbicular with rounded auricles, apex usually acute, margin entire or undulate, base cordate to truncate; petioles 0.5–3.5 cm. Flowers 1–2 (-3), axillary, pedunculate; peduncles 2–4 (-6.5) cm; bracteoles 2–4 mm, lanceolate; pedicels 5–10 mm; outer sepals 7–9 × 5–7 mm, elliptic, obtuse, mucronate; corolla 1.5–2.5 cm long, pink, very shallowly lobed with slightly fimbriate margins, midpetaline bands dark, pilose, terminating in a pilose mucro; ovary glabrous; style divided 6–10 mm above base, stigmas 1.5–3.5 mm, cylindrical to linear, unusually variable. Capsule glabrous; seeds rugose. [O’Donell 1957: 161–166 (Figure 6); Hoffmann 1998: 207]

##### Distribution.

Endemic to central Chile from Antofagasta south to Santiago (*Worth & Morrison* 16236, *Bridges* s.n., *Gardner & Knees* 5652, 8467, *DCI* 1791). 0–1800 m.

##### Notes.

This polymorphic species is usually easily distinguished from all other South American species by the leaves with bifurcate auricles combined with pink corollas usually around 2–2.5 cm long. However, some specimens from Coquimbo (*Simon* 312 (MICH, RSA), *Wagenknecht* 18445 (F)) have small, suborbicular sericeous leaves and merit further study. The type location is given as Chillán, but this is almost certainly an error as the plant has never subsequently been collected so far south (O’Donell 1957: 161).

#### 45. Convolvulus bonariensis

Taxon classificationPlantaeSolanalesConvolvulaceae

Cav., Icon. 5: 54, pl. 480(2). 1799. (Cavanilles 1799: 54).

Aniseia diversifolia Walpers, Nov. Act. Acad, Caes. Leop. Carol. Nat. Cur. 367. 1843. (Walpers 1843: 367). Type. CHILE, Valparaiso, *Meyen* s.n. (holotype B?†).
Convolvulus triflorus Phil., Linnaea 33: 183. 1864, nom. illeg., non *Convolvulus triflorus*Vahl (1794). (Phillipi 1864: 183). Type. CHILE, Santiago, *Philippi* s.n. (whereabouts unknown).
Ipomoea cordobana Peter, Nat. Pflanzenfam. [Engler & Prantl] 4(3a): 36. 1891. (Peter 1891: 36). Type. ARGENTINA, Cordoba, *Lorentz* 130 (lectotype GOET005724, designated by Staples et al. 2012: 676).
Convolvulus bonariensis var. multiflorus Phil., Anal. Univ. Santiago 90: 222. 1895. (Phillipi 1895: 222). Type. CHILE, Santiago, Quinta Normal, *Phillipi* s.n. (SGO, not seen).
Convolvulus dissectus var. diversifolius (Kunze ex Walp.) Reiche, Anal. Univ. Chile 120: 827. 1907. (Reiche 1907: 827). Type. Based on *Aniseia diversifolia* Walpers

##### Type.

ARGENTINA, Pampas de Buenos Aires, *Née* s.n. (lectotype MA-475568!, sheet with location “pampas de Buenos Ayres” and determination by O’Donell, designated here).

##### Description.

Finely adpressed-pubescent herb from a thick rootstock, sometimes sericeous on young parts; stems trailing, up to 2 m long, 1–3 mm in diameter. Leaves petiolate, 3–6 (-11) × 1.2–3 (-6) cm, very variable in shape but usually oblong-lanceolate or strap-shaped with pronounced (rarely bifurcate) auricles, occasionally ovate-deltoid, characteristically 5 times as long as broad, apex usually obtuse, mucronate, margin undulate to crenate or serrate, base hastate to cordate; petiole 5–40 mm. Flowers (1-) 2–5 in axillary, pedunculate cymes; peduncles 0.6–4 (-6) cm; bracteoles 2–4 mm, narrowly lanceolate; pedicels 3–12 mm; outer sepals 6–8 × 3–5 mm, elliptic, acute; corolla 1–1.5 cm long, pink, shallowly lobed, midpetaline bands pubescent terminating in teeth; ovary glabrous, style divided 3–6 mm above base; stigmas c. 1.5 mm, narrowly ellipsoid. Capsule glabrous; seeds smooth to indistinctly tuberculate. [O’Donell 1957: 159-160 (Figure 5); O’Donell 1959: 267–270 (Figure 44)]

##### Distribution.

Argentina (*Hieronymus* 916, *Grisebach* 130, *Pedersen* 8251); Uruguay (*King* s.n.); Chile (*Gay* s.n., *Phillipi* s.n.). 0–1950 m. Records from Bolivia are errors.

##### Notes.

The adpressed pubescent, lanceolate to strap-shaped leaves combined with the small corolla are characteristic. Flower size and usually leaf shape serve to distinguish it from *Convolvulus chilensis*, with which it sometimes grows in Chile. It is morphologically extraordinarily similar to *Convolvulus aschersonii* from South Africa which differs in its paler, more deeply lobed corollas and usually shorter sepals.

#### 46. Convolvulus demissus

Taxon classificationPlantaeSolanalesConvolvulaceae

Choisy, Prodr. [A.P. de Candolle] 9: 404. 1845. (Choisy 1845: 404).

Convolvulus andinus Phil., Linnaea 33: 184. 1864. (Phillipi 1864: 184). Type. CHILE, Santiago, *Philippi* s.n. (holotype SGO, not seen).
Convolvulus ovatus Phil., Anal. Univ. Santiago 90: 221. 1895. (Phillipi 1895: 221). Type. CHILE, Maule, Rio Maule, *Phillipi* s.n. (holotype SGO, not seen).
Convolvulus demissus var. andinus (Phil.) Reiche, Anal. Univ. Chile 120: 826 (Reiche 1907: 826). Type. Based on *Convolvulus andinus* Phil.
Convolvulus demissus var. ovatus (Phil.) Reiche, Anal. Univ. Chile 120: 826 (Reiche 1907: 826). Type. Based on *Convolvulus ovatus* Phil.

##### Type.

CHILE, Coquimbo, *Gay* s.n. (holotype G; several isotypes P!).

##### Description.

Glabrous or puberulent herb from a deep rootstock. Stems 30(-50) cm long, numerous, trailing. Leaf blade 0.6–2 × 0.4–1.6 cm, ovate-deltoid; base truncate and briefly cuneate onto the petiole, auricles not well-developed; apex obtuse and mucronate or acute; margin entire; petiole 4–8 (-12) mm. Flowers solitary (rarely paired), axillary, pedunculate; peduncles 10–18 mm; bracteoles 2–6 mm, linear; pedicels 2–4 mm; outer sepals 7–10 × 5–7 mm, elliptic, obtuse; corolla (1-) 1.5 (-2) cm long, pink, shallowly lobed, midpetaline bands pilose, terminating in small teeth; ovary glabrous; style divided 4–5 mm above base, stigmas 2–3 mm. Capsule glabrous; seeds smooth but with slightly muricate angles. [O’Donell 1957: 165–167 (Figure 7); O’Donell 1959: 276]

##### Distribution.

Central Chile (*Morisson* 16746, *Cuming* 214, *Gardner et al.* 52, *UCEXC* 43) and adjacent parts of Argentina (fide O’Donell 1959: 277). 1500–2700 m.

##### Notes.

This is an Andean species variable in indumentum with a superficial resemblance to *Convolvulus arvensis* but with much larger sepals and ovate-deltoid leaves with poorly developed basal auricles. It is also very similar to some forms of the African *Convolvulus sagittatus*.

#### 47. Convolvulus schulzei

Taxon classificationPlantaeSolanalesConvolvulaceae

O’Donell, Lilloa 26: 360, f. 3. 1953. (O’Donell 1953: 360).

##### Type.

ARGENTINA, Chaco, *Schulz* 3556 (holotype LIL!).

##### Description.

Finely pubescent trailing or twining herb from a thick rootstock, stems up to 1 m long. Leaves petiolate, 1–5 × 0.5–2 cm, ovate-deltoid, auriculate, apex obtuse and mucronate, margin weakly crenate, base cordate; petioles 3–13 mm. Flowers 1–4 in axillary, pedunculate cymes; peduncles 1–3 (-8.5) cm; bracteoles 1.5–2.5 mm, narrowly ovate; pedicels 5–15 mm; outer sepals 4–6 × 4–5 mm, broadly elliptic to obovate, obtuse, inner sepals truncate; corolla 0.7–0.8 cm long, pale pink, shallowly lobed, midpetaline bands pilose in the upper half terminating in small teeth; ovary glabrous, acuminate; style glabrous, divided 3.5–4 mm above base; stigmas 1.5 mm. Capsule glabrous; seeds strongly tuberculate. [O’Donell 1959: 291]

##### Distribution.

Endemic to Argentina: Corrientes and Chaco (*Pedersen* 4418).

##### Notes.

A local endemic with a small corolla growing on sand deposits in river valleys.

#### 48. Convolvulus laciniatus

Taxon classificationPlantaeSolanalesConvolvulaceae

Desr., Encycl. [Lamarck et al.] 3: 546. 1792. (Desrousseaux 1792: 546).

Figure 9, t. 1–7

Convolvulus laciniatus var. hirsutus Desr., Encycl. [Lamarck et al.] 3: 546. 1792. (Desrousseaux 1792: 546). Type. URUGUAY, *Commerson* (holotype P, not found).
Convolvulus lasianthus Cav., Icon. 5: 53, t. 479(1). 1799. (Cavanilles 1799: 53). Type. CHILE, Talcahuano, *Née* (lectotype, MA-475572!, sheet numbered “1365” with rootstock and two corollas, designated here).
Convolvulus laciniatus var. peduncularis Meisn., Fl. Bras. (Martius) 7: 314. 1869. (Meisner 1869: 314). Type. Brazil. *Sello* s.n. (lectotype P03560958, designated here).
Ipomoea polymorpha Meisn. var. glabra Griseb. Symb. Bot. Arg. 264. 1879. (Grisebach 1879: 264). Type. ARGENTINA, Tucuman, (GOET?).
Convolvulus geranioides Phil., Anales Univ. Santiago 90: 222. 1895. (Phillipi 1895: 222). Type. CHILE, Bucalemu and Cahuil, *L. Sanfurgo* (syntypes SGO, not seen).

##### Type.

URUGUAY, Montevideo, *Commerson* s.n. (holotype P-Juss!).

##### Description.

**Figure 9.**
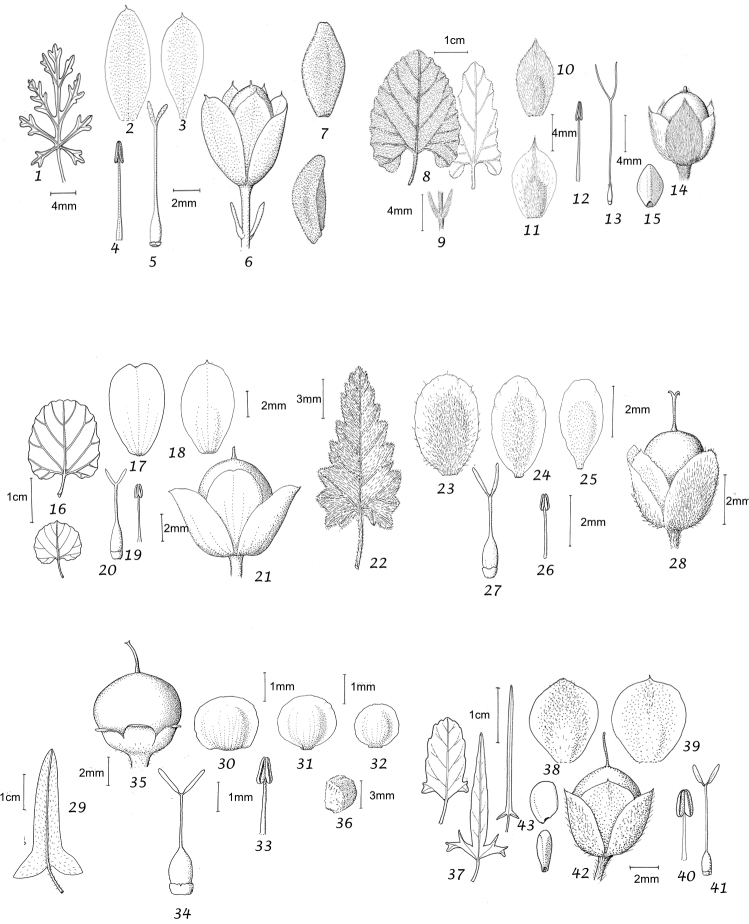
**1–7** *Convolvulus laciniatus* **1** leaf **2** outer sepal **3** inner sepal **4** stamen **5** ovary and style **6** capsule with calyx and bracteoles **7** seeds. From *Wood et al.* 22627 (K) **8–15** Convolvulus hermanniae subsp. erosus **8** leaves **9** bracteoles **10** outer sepal **11** inner sepal **12** stamen **13** ovary and style **14** capsule, apically hirsute **15** seeds. From *Buchtien* 15/11/1885 (OXF) **16–21** *Convolvulus montanus* **16** leaves **17** outer sepal **18** inner sepal **19** stamen **20** ovary and style **21** calyx and capsule. From *Tutin* 1008 (BM) **22–28** *Convolvulus crispifolius* **22** leaf **23** outer sepal **24** middle sepal **25** inner sepal **26** stamen **27** ovary and style **28** calyx and capsule. From *Chinnock* 2915 (AD) **29–36** *Convolvulus microsepalus* **29** leaf and bracteole (left) **30** outer sepal **31** middle sepal **32** inner sepal **33** stamen **34** ovary and style **35** calyx and capsule **36** seed. From *Orchard* 211 (AD) and *Badman* 32 (AD) **37–43** *Convolvulus angustissimus* **37** leaves showing three forms on same plant **38** outer sepal **39** inner sepal **40** stamen **41** ovary and style **42** calyx and capsule **43** seeds. From *Spicer* 31/1/1875 (OXF).

Very variable perennial herb with numerous, often branched, trailing stems from a stout central rootstock, most commonly glabrous, sometimes thinly pubescent and rarely white-tomentose. Leaves petiolate, 1–3(-5) × 1–3(-5) cm, very variable in form but always deeply divided, usually profoundly palmatisect or pinnatisect with narrow laciniate segments, rarely with broader segments; apex obtuse or acute; margin undulate or entire; petioles 3–15 mm. Flowers 1–2(-3), axillary, pedunculate; peduncles 1–3(-5) cm; bracteoles 2–3.5 mm; pedicels 2–10 mm; outer sepals 6–9 × 5–6 mm, elliptic to obovate, margins scarious; corolla 1–2 cm long, white or white with purple centre, lobed with acute apices, exterior glabrous to thinly pilose in correlation with overall plant indumentum, midpetaline bands present or absent but if present dark violet, pilose; ovary glabrous; style glabrous, divided 5–11 mm above base; stigmas 1.5–2 mm. Capsule glabrous; seeds smooth, black. [O’Donell 1957: 169 -172, Figure 9; O’Donell 1959: 281]

##### Distribution.

Argentina (*Tressens et al.* 2282, *Pastore* 1262), Chile, Uruguay (*Gibert* 40); Brazil (fide O’Donell 1959: 284); Bolivia (*Wood* 22627, *Bang* 959, *Fiebrig* 2587). 0–3800 m.

##### Notes.

An extremely variable species easily recognised by its deeply divided leaves and white flowers, which are occasionally with dark violet midpetaline bands. The following specimens are outstanding and could each constitute a distinct taxon, but which I hesitate to recognise in the absence of any matching material: *Venturi* 8624 (BM, K, MO) from Argentina, which has very large leaves 5 × 5 cm with broad segments, the peduncles up to 6 cm long bearing up to three flowers and is apparently similar to *Convolvulus geranioides* from Chile; *Sandoval & Stark* 1025 (K) from Chile, which has small pinnatisect, white-tomentose leaves. There are also densely pubescent specimens from Uruguay (*Gay* s.n., *Seijo et al.* 2381, for example) which may accord with var. hirsutus Desr. Corolla indumentum and colouring are also outstandingly variable, some corollas completely glabrous while in others the midpetaline bands are pubescent.

Two specimens from Bolivia (*Wood et al.* 21956 and *Bastian* 783, both LPB) have finely dissected leaves like *Convolvulus laciniatus* but a hairy apex to the ovary. They probably represent the hybrid between *Convolvulus laciniatus* and Convolvulus hermanniae subsp. erosus, certainly *Wood* 21956 was growing in the vicinity of both parents.

#### 49. Convolvulus hermanniae

Taxon classificationPlantaeSolanalesConvolvulaceae

L’Hér., Stirp. Nov. 67. 1788, t 33. 1788. (L’Héritier 1788: 67).

##### Type.

PERU, Huara, *Dombey* (lectotype P-00608800!, sheet labelled “Perou Dombey”, designated here; isolectotypes P!, BM000953290!).

##### Description.

Trailing or (less commonly) twining herb from a thickened woody rootstock c. 1 cm thick, all vegetative parts grey-tomentose. Stems up to 1 m long, apparently more slender in twining plants, numerous. Leaves petiolate, 2–6.5 × 0.5–3 cm, ovate to ovate-deltoid, the auricles not well-developed, apex acute to obtuse, margin undulate to irregularly dentate, base cordate; petioles 5–12 (-22) mm. Flowers 1–2 (-3), axillary, pedunculate; peduncles 1–3 (-6)cm, often shorter than the leaves; bracteoles 2–4 mm, linear-lanceolate; pedicels 5–12 mm; outer sepals 7–10 × 4–6 mm, (narrowly) elliptic, usually acute; corolla 1.4–1.8 cm long, white, shallowly lobed, midpetaline bands extended into mucros, tomentose; ovary conical, 1.5–2 mm, glabrous or apically pilose, style divided 5–7 mm above base, glabrous except immediately above ovary, stigmas 2.5–3 mm. Capsule glabrous or apically pilose; seeds smooth.

We recognise two subspecies based on ovary and capsule indumentum:

#### 49a. Convolvulus hermanniae subsp. hermanniae

Taxon classificationPlantaeSolanalesConvolvulaceae

Convolvulus incanus Vahl, Symb. Bot. 3: 23. 1794. (Vahl 1794: 23). Type. PERU, *Dombey* s.n. (lectotype C!, sheet with Dombey’s name, designated here).
Ipomoea hermanniae (L’Hér.) G. Don, Gen Hist. 4: 276. 1838. (Don 1838: 276). Type. Based on *Convolvulus hermanniae* L’Hér.

##### Distinguishing features.

Ovary and capsule completely glabrous.

##### Distribution.

The principal or only subspecies in Ecuador and Peru extending south into Bolivia: Ecuador (*Spruce* 5810); Peru (*Thomas* 3/1, *Mathews* 377); Bolivia (*Badcock* 607, *Beck et al.* 31637), northern Argentina (*Fortunato et al.* 4648). 2200–2880 m.

##### Notes.

The name *Convolvulus incanus* was, and still is (Hyam 2011: 554), commonly misapplied to *Convolvulus equitans* from North America, although it is clearly indicated that the type was collected by Dombey; perhaps it was grown from seeds with the same origin as those from which the type of *Convolvulus hermanniae* was grown.

#### 49b. Convolvulus hermanniae subsp. erosus

Taxon classificationPlantaeSolanalesConvolvulaceae

(Desr.) J.R.I.Wood & R.W.Scotland stat. nov.

urn:lsid:ipni.org:names:77147670-1

Figure 9, t. 8–15

Convolvulus erosus Desr., Encycl. [Lamarck et al.] 3: 558. 1792. (Desrousseaux 1792: 558). Type. URUGUAY, Montevideo, *Commerson* s.n. (holotype P-Juss!).
Convolvulus crenatus Vahl, Symb. Bot. 3: 31. 1794, nom. illeg., non *Convolvulus crenatus* Jacq. (1789). (Vahl 1794: 31). Type. BRAZIL, *Thouin* s.n. (?C. not seen).
Convolvulus vahlii Roem. & Schult., Syst. Veg, ed. 15 bis [Roemer & Schultes] 4: 280. 1819. (Roemer and Schultes 1819: 280). Type. Based on *Convolvulus crenatus* Vahl
Convolvulus costatus Meyen, Reise Erde 1: 264. 1834. (Meyen 1834: 264). Type. CHILE, Santiago, *Meyen* s.n. (B†).
Aniseia costata (Meyen) Walp., Nov. Act.Acad, Caes, Leop. Carol.Nat. Cur.367 (1843). (Walpers 1843: 367). Type based on *Convolvulus costatus* Meyen
Convolvulus hermanniae var. elongatus Choisy, Prodr. [A.P. de Candolle] 9: 409. 1845. (Choisy 1845: 409). Type. URUGUAY, Montevideo, *Commerson* s.n. (holotype P [Herb. Juss.]).
Convolvulus hermanniae var. viridis Meisn., Fl. Bras. (Martius) 7: 312. 1869. (Meisner 1869: 312). Type. URUGUAY, Montevideo, *Sellow* s.n. (not found).
Convolvulus mollis var. albidovillosus Chodat & Hassl., Bull. Herb. Boissier ser. 2, 5: 699. 1905. (Chodat and Hassler 1905: 699). Type. PARAGUAY, Valenzuela, *Hassler* 7103 (holotype G).
Convolvulus hermanniae var. albidovillosus (Chodat & Hassl.) Hassl., Repert. Spec. Nov. Regni Veg. 9: 195. 1911. (Hassler 1911: 195). Type. Based on Convolvulus mollis var. albidovillosus Chodat & Hassl.

##### Type.

Based on *Convolvulus erosus* Desr.

##### Distinguishing features.

Ovary and capsule apically pilose. [O’Donell 1957: 167 p. p.; O’Donell 1959: 271 p.p.].

##### Distribution.

The only subspecies present in the Southern Cone extending north to Bolivia and Brazil: Chile (*Cuming* 280, *Buchtien* s.n. [19/11/1895]); Bolivia (*Wood* 19217, *Bang* 990); Argentina (*Seijo* 1354, *Venturi* 5464); Uruguay (*Gibert* s.n.); Brazil (*Glaziou* 19668). 0–2800 m.

##### Notes.

*Convolvulus hermanniae* is distinguished from *Convolvulus crenatifolius* by its few-flowered peduncles and dense white-tomentellous indumentum. From *Convolvulus bonariensis* it differs in its white-tomentellous leaves. Subsp. erosus is unique amongst American taxa because of its hirsute ovary and capsule. O’Donell 1957: 167–169 Figure 8; O’Donell 1959: 277.

#### 50. Convolvulus montanus

Taxon classificationPlantaeSolanalesConvolvulaceae

Ooststr., Meded. Bot. Mus. Herb. Rijksuniv. Utrecht 7, 30: 199, f. 1, 3. 1933. (Ooststroom 1933: 199).

Figure 9, t. 15–21

##### Type.

PERU, Junin, Huancayo, *Killip & Smith* 22018 (holotype F!; isotypes BM001035802!, MA!, US).

##### Description.

Herb apparently from a deep rootstock, glabrous or with a few scattered hairs on vegetative parts, stems trailing, 5–15 cm long. Leaves petiolate, 0.6–1.6 × 0.5–1.3 cm, ovate, apex more or less rounded, margin strongly crenate, base truncate; petioles 3–8 mm. Flowers 1(-2), axillary, pedunculate; peduncles 1.2–1.6 cm; bracteoles 2.5–4 mm, oblong; pedicels 2–5 mm; outer sepals 6–8 × 4.5–6 mm, obovate-elliptic, concave, scarious, emarginate, inner sepals similar emarginated or mucronulate; corolla 1.2–1.5 cm long, white to pale pink with a dark centre, deeply lobed, midpetaline bands glabrous terminating in a small tooth; ovary glabrous, style divided 5–7 mm above base, stigmas c. 2 mm. Capsule glabrous; seeds smooth, glabrous. O’Donell 1959: 288–290 (Figure 47).

##### Distribution.

Peru: Junin, Cusco (*Stafford* 220); Bolivia: La Paz (*Gütte* 141); Argentina: Mendoza, Buenos Aires (*Melis et al.* 427). Unusually disjunct geographically and altitudinally: 3300–3500 in Peru and Bolivia but below 2000 m in Argentina.

##### Notes.

A distinctive nearly glabrous species with small ovate, basally truncate, crenate leaves.

#### 51. Convolvulus incisodentatus

Taxon classificationPlantaeSolanalesConvolvulaceae

J.R.I.Wood & R.W.Scotland nom. nov.

urn:lsid:ipni.org:names:77147667-1

Convolvulus incisus Choisy, Prodr. [A.P. de Candolle] 9: 409. 1845. (Choisy 1845: 409). Type. PERU, Chinchin, Dombey s.n. (holotype P-03537718!, possible isotype MA 814635!).

##### Type.

Based on *Convolvulus incisus* Choisy.

##### Description.

Perennial herb from a tap root, thinly pubescent on all vegetative parts. Stems 15-40 cm long, trailing. Leaf blade1–2.3 × 0.6–1.2 cm, ovate-deltoid; base cordate and briefly cuneate onto the petiole, auricles prominent; apex shortly mucronate; margin incised-dentate; petiole 3–5 mm. Flowers solitary (rarely paired), axillary, pedunculate; peduncles 8–14 mm; bracteoles 1–2 mm, filiform; pedicels 3–5 mm; outer sepals 6–7 × 4mm, ovate, obtuse; inner sepals 6-7 × 6-7mm, suborbicular, rounded, slightly scarious; corolla 1.5–1.6 cm long, white, lobed, midpetaline bands pubescent, terminating in triangular teeth; ovary glabrous; style divided 4–5 mm above base, stigmas 2.5 mm. Capsule glabrous; seeds smooth, glabrous.

##### Distribution.

Moquegua (*Dillon et al.* 3327) and Piura in Peru, 600–700 m.

##### Notes.

A poorly-known species growing at low altitudes in Peru but easily recognised by its incised-dentate leaves. Morphologically it would appear to lie between *Convolvulus laciniatus* and *Convolvulus montanus*.

#### 52. Convolvulus crenatifolius

Taxon classificationPlantaeSolanalesConvolvulaceae

Ruiz & Pav., Fl. Peruv. 2: 10. 1799. (Ruiz and Pavón 1799: 10, tab. 118).

##### Type.

PERU, Huanuco *Ruiz & Pavón s.n.* s.n. (lectotype MA-814634, designated here; isolectotypes MA 814632, 814633).

##### Description.

Pubescent to densely hirsute herb; stems twining up to 3 m high. Leaves petiolate, 3–8 × 1–4 cm, ovate-deltoid, strongly auriculate, usually large, apex usually obtuse and mucronate, margin undulate to sinuate, base broadly cordate to hastate with midrib area cuneate onto petiole; petioles 7–15 mm. Flowers (1-) 3–7 in compact axillary, pedunculate umbellate cymes; peduncles 1.5–12 cm; bracteoles 2–5 mm, narrowly lanceolate; pedicels 2–12 mm, apparently accrescent after anthesis; outer sepals 6–6.5 × 3.5–5 cm, elliptic, obtuse or acute; corolla 1.1–1.5 cm long, white to pink, deeply lobed, midpetaline bands brownish, pilose, terminating in a mucro; ovary glabrous; style divided c. 7 mm above base; stigmas 3 mm, more or less included. Capsule glabrous; seeds smooth. [O’Donell 1959: 271 p. p., Carranza (2008: 4ff.)]

##### Notes.

We recognise two subspecies, which are distinct through most of their range, but intergrade in parts of northern Argentina (*Morel* 5885 from Formosa, *Schwarz* 6391 from Misiones and *Risso* 30 from Santiago de Estero are examples) and in the São Paulo area of Brazil (*Hoehne* 265), mostly at altitudes of around 1000 m.

#### 52a. Convolvulus crenatifolius subsp. crenatifolius

Taxon classificationPlantaeSolanalesConvolvulaceae

Figure 8, t. 40–46

Convolvulus crenatifolius var. peruvianus Hallier f., Jahrb. Hamburg. Wiss. Anst. 16, beiheft 3: 34. 1899, nom illeg. superfluous name for autonymic variety (Hallier 1899: 34).

##### Distinguishing features.

Distinguished by the more numerous flowers (there are nearly always some cymes with >3 flowers), the relatively short pedicels, the cymes usually forming rather tight umbellate clusters, and the smaller, usually pinkish, lobed corolla.

##### Distribution.

Amphitropical, Andes and southern Brazilian highlands in South America; United States of America and Mexico in North America: Ecuador (*Lodiro* 113/5); Peru (*Stafford* 1041, *Lechler* 2116); Bolivia (*Wood* 17714, *Bang* 1158); Argentina (Meyer 5018, *Villa* 543); Brazil (*Meireles et al.* 2783, *Tamandaré & Brade* 6987); United States: Texas (*Runyon* 2599, 4479, *Correll & Wasshausen* 27684); Mexico: Guadelupe (*Schmitz* 1098 (W), Hidalgo, *Rose et al.* 8946 (P, US). Approximately 1500–3000 m in South America but to near sea level in Texas.

##### Notes.

In South America this species appears to be distinctly montane in distribution being limited to the Andes and the higher mountains of southeastern Brazil. Specimens from Andean Bolivia, Peru and Ecuador are very consistent in habit. Its status in North America is uncertain. The leaves are often more strictly triangular and more coarsely dentate than in South American plants but some specimens such as *Dusén* 7788 from Paraná, Brazil are indistinguishable. It may be an introduction in North America like *Convolvulus farinosus* – we have seen no specimens collected before the 20^th^ century – but equally it may be the result of long-term dispersal. Species of Convolvulaceae in varous genera, such as *Ipomoea amnicola* Morong and *Evolvulus arizonicus* A.Gray show a disjunct amphitropical distribution between North and South America so a distribution of this kind is not improbable.

#### 52b. Convolvulus crenatifolius subsp. montevidensis

Taxon classificationPlantaeSolanalesConvolvulaceae

(Spreng.) J.R.I.Wood & R.W.Scotland. stat. nov.

urn:lsid:ipni.org:names:77147671-1

Figure 8, t. 25–31

Convolvulus montevidensis Spreng., Syst. Veg. [Sprengel] 1: 604. 1824. (Sprengel 1824: 604). Type. URUGUAY, Montevideo, *Sellow* s.n. (B†); [URUGUAY], Montevideo, *Sellow* 278 (neotype NY 00318926, designated here).
Ipomoea montevidensis (Spreng.) G. Don, Gen. Hist. 4: 276. 1838. (Don 1838: 276). Type. Based on *Convolvulus montevidensis* Spreng.
Ipomoea ottoensis Choisy, Prodr. [A.P. de Candolle] 9: 378. 1845. (Choisy 1845: 378). Type. URUGUAY, Montevideo, *Otto* s.n. (P, not found).
Convolvulus ottonis Meisn., Fl. Bras. (Martius) 7: 311, t. 113. 1869. (Meisner 1869: 311). Type. BRAZIL, Minas Gerais, *Lindberg* 165 and URUGUAY, Montevideo, *Otto* s.n. (syntypes B†).
Convolvulus montevidensis var. megapotamicus Meisn., Fl. Bras. (Martius)7: 312. 1869. (Meisner 1869: 312). Type. specimen annotated *Convolvulus megapotamicus* Spreng. (holotype B†).
Convolvulus crenatifolius var. montevidensis (Spreng.) Hallier f., Jahrb. Hamburg. Wiss. Anst. 16, Beih. 3: 34. 1899. (Hallier 1899: 34). Type. Based on *Convolvulus montevidensis* Spreng.
Convolvulus crenatifolius var. argentinicus Hallier f., Jahrb. Hamburg. Wiss. Anst. 16, beiheft 3: 34. 1899. (Hallier 1899: 34). Type. ARGENTINA, *Lorentz & Hieronymus* 1057, 945, *Lorentz* 1538, *Kuntze* s.n.Dec. 1891 (syntypes B, all†)
Convolvulus montevidensis var. ottonis (Meisn.) Chodat & Hassl., Bull. Herb. Boissier, ser. 2, 5: 699. 1905. (Chodat and Hassler 1905: 699). Type. Based on *Convolvulus ottonis* Choisy

##### Type.

Based on *Convolvulus montevidensis* Spreng.

##### Distinguishing features.

Distinguished from subsp. crenatifolius by the more slender stems (c. 1 mm diameter on flowering shoots), 1–3-flowered cymes, relatively long pedicels, so inflorescence appears rather lax, and the larger (1.5–2.5 cm long), almost unlobed cream corollas with stigmas very slender, often exserted. The reddish-brown, somewhat scarious, slightly larger (7–9 mm long) outer sepals are often distinctive too. O’Donell 1959: 271–276 p. p., figure 45 under *Convolvulus crenatifolius*.

##### Distribution.

Northern Argentina (*Renvoize* 3072, *Tweedie* 379, *Tressens et al.* 2467, *Hawkes et al.* 3263); Paraguay (*Pedersen* 60, *Jorgensen* 4033, *Lourteig* 2061, *Balansa* 1066); Uruguay (*St.-Hilaire* 2384, 2430); Southern Brazil, particularly Rio Grande do Sul (*Rambo* 46807) and Parana (*Kissmann* 7788); Bolivia (*Villarroel et al.* 1466). 0–?1500 m (Upper limit uncertain). Apparently centred on Paraguay.

##### Notes.

According to the image in *Flora Brasiliensis*, *Convolvulus ottonis* represents a plant with broadly elliptic sepals similar to the neotype of *Convolvulus montevidensis*. The neotype may or may not represent part of the original collection on which Sprengel’s description was based but was collected by Sellow at Montevideo and originates from the Berlin herbarium. It shows the large corolla and reddish brown sepals so characteristic of this subspecies which is common in parts of Paraguay, southern Brazil, northern Argentina and Uruguay.

There is at least one collection from Paraguay (*Hansen* 7679 [C, NY]) which shows evidence of introgression with *Convolvulus hasslerianus*. The calyx is that of Convolvulus crenatifolius subsp. montevidensis but the flowers are solitary and the leaves are very shortly petiolate with subentire margins.

#### 53. Convolvulus lilloi

Taxon classificationPlantaeSolanalesConvolvulaceae

O’Donell, Lilloa 29: 293. 1959. (O’Donell 1959: 285).

##### Type.

ARGENTINA, Corrientes, Ituzaingó, *Meyer* 5786 (holotype LIL!).

##### Description.

Densely pubescent or tomentose trailing or twining herb from a thickened rootstock, stems to at least 1 m long, relatively stout. Leaves petiolate, 2–7(-10) × 0.7–2.8 cm, lanceolate-oblong with pronounced basal auricles, these sometimes lobed, apex acute and mucronate, margin entire to weakly undulate, base hastate; petioles 3–10(-15) mm. Flowers solitary (very rarely paired), axillary, pedunculate; peduncles 2–5 cm; bracteoles 2–5 mm, linear-lanceolate; pedicels 10–15 mm; outer sepals 10–13 × 5–6.5 mm, elliptic, obtuse; corolla 2.5–4 cm long, white with dark centre, shallowly lobed, midpetaline bands pilose, extended into teeth; ovary glabrous, style glabrous, divided c. 16 mm above base, stigmas 2–3 mm, cylindrical. Capsule glabrous; seeds smooth to finely tuberculate. [O’Donell 1959: 285–288 (Figure 46)]

##### Distribution.

Argentina: Corrientes and Misiones (*Pedersen* 1912, *Ferraro* 3094); Brazil: Rio Grande do Sul (*Lindeman et al.* 8417, *Ferreira* 110). 0–500 m.

##### Notes.

An apparently very distinctive local endemic characterised by its large, usually solitary flowers and densely hairy indumentum. However, a number of specimens indicate that it is more variable than O’Donell supposed. *Ibarrola* 1305 from Corrientes probably fits but the sepals are only 9 mm long and the corolla a mere 2.5 cm in length. *Dusén* 7310 and *Smith & Kiela* 7814 from southern Brazil appear to be intermediate with *Convolvulus hasslerianus* as the leaves are only very shortly petiolate.

#### 54. Convolvulus ensifolius

Taxon classificationPlantaeSolanalesConvolvulaceae

P.P.A.Ferreira & Simão-Bianchini, Phytotaxa 135: 29. 2013. (Ferreira et al. 2013: 29).

##### Type.

BRAZIL, Rio Grande do Sul, *P. P.A. Ferreira 300* (holotype ICN; isotypes K!, SP).

##### Description.

Prostrate perennial with woody base, the stems winged, twining at the apices. Leaves shortly petiolate, 2–6 × 0.2–0.4 cm, linear-oblong, base sagittate, apex acute, glabrous; petiole 2–6 mm. Flowers solitary; peduncles 25–60 mm, shortly winged; bracteoles 3–5 nn, lanceolate, deciduous; pedicels 8–12 mm; outer sepals 6–8 × 3–4 mm, obovate, truncate, mucronulate, glabrous or canescent, the margins ciliolate; inner sepals similar but with scarious margins; corolla 1.8–2.2 cm long, white, glabrous, midpetaline bands sericeous; ovary glabrous, subglobose; style divided 6–9 mm above base; stigmas 3–4 mm. Capsule glabrous, apiculate; seeds black, glabrous.

##### Distribution.

Brazil (Rio Grande do Sul, Paraná).

##### Notes.

Resembling *Convolvulus lilloi* and *Convolvulus hasslerianus* in the solitary flowers and shortly petiolate leaves but near glabrous, the leaves linear-oblong and the sepals much shorter.

#### 55. Convolvulus hasslerianus

Taxon classificationPlantaeSolanalesConvolvulaceae

(Chodat) O’Donell, Lilloa 23: 430. 1950. (O’Donell 1950: 430).

Breweria hassleriana Chodat, Bull. Herb. Boiss, ser, 2, 5: 683. 1905. (Chodat and Hassler 1905: 683). Type. PARAGUAY, Carimbatay, *Hassler* 4541 (holotype G; isotype NY!).

##### Type.

Based on *Breweria hassleriana* Chodat

##### Description.

Usually erect but occasionally decumbent to ascending cerrado perennial with woody xylopodium, vegetative parts villous with long spreading cobwebby hairs; stems several, erect, herbaceous, 20–30(-50) cm high. Leaves sessile or very shortly petiolate, 1.5–4.5 × 0.8–1.5 cm, ovate to oblong-ovate; base rounded, truncate to subcordate or sagittate; apex acute or (above) apiculate; margin entire; petiole 0(-2) mm. Flowers solitary (rarely paired); peduncles 20–50 mm; bracteoles 3–5 × 0.5–1 mm, linear-lanceolate, finely acuminate; pedicels 5–10 mm; outer sepals 10–15 × 5–7 mm, ovate, acuminate; corolla 3.2–4 cm long, white, unlobed, midpetaline bands pink, long-pilose, terminating in a mucro; ovary glabrous; style glabrous, divided 8–12 mm above base, persistent in fruit; stigmas 3 mm. Capsule glabrous, unusually large, c. 1 cm diameter; seeds smooth. [O’Donell 1950: 430–431, f. 3]

##### Distribution.

Paraguay (*Krapovickas et al*. 45901, *Hassler* 9123, *Balansa* 1056); Brazil: Parana (*Hatschbach* 3675, 20011, 30722), Rio Grande do Sul (*Gaudichaud* 659). 0–800 m.

##### Notes.

Unique species because of its adaptation to the cerrado biome. The erect habit, xylopodium, woolly, often cobwebby indumentum, subsessile leaves and large corolla all render it distinct from other American species.

### Species 56–69. Australasian species

Herbaceous trailing or twining species, relatively slender compared with species from other regions. Leaves petiolate, with the lamina narrowed to a sagitate or hastate base, commonly dimorphic or even trimorphic; basal leaves often simple, stem leaves often lobed with narrow segments. The ovary, style and capsule are always glabrous. Although Johnson (2001) describes all species as perennials, several appear at least sometimes to be annuals and we have seen specimens of *Convolvulus crispifolius*, *Convolvulus eyreanus* and *Convolvulus recurvatus*, which certainly appear to be annuals. All three are species with recurved fruiting peduncles, a character which is often associated with the annual habit in *Convolvulus*.

The taxonomy of Australasian species is difficult. Many species are superficially similar especially when young or showing only one leaf form, and the taxonomy is often based on the direction of the fruiting peduncle and seed sculpture so non-fruiting specimens and incomplete specimens can be impossible to name. In many herbaria, all species from this region were once filed under the name *Convolvulus erubescens* following Bentham (1869), but this is clearly a gross oversimplification. However, it seems probable that further intensive study is needed before species delimitation is entirely satisfactory–*Convolvulus angustissimus* and *Convolvulus clementii* in particular appear to embrace a variety of forms from which the more distinct entities have been separated off as separate species.

Australian species are recorded as adventives or naturalised in other countries including the British Isles (Sell and Murrell 2009) and New Zealand (Heenan et al. 2003), usually under the name *Convolvulus erubescens*. We have not seen any of these collections and it is not certain to which Australian species these records refer.

#### 56. Convolvulus microsepalus

Taxon classificationPlantaeSolanalesConvolvulaceae

R.W.Johnson, Austrobaileya 2: 410. 1987. (Johnson 1987: 410).

Figure 9, t. 29–36

##### Type.

AUSTRALIA, South Australia, *Orchard* 2626 (holotype AD!; isotypes NCU, COLO).

##### Description.

Perennial herb from a central taproot with trailing stems to 1 m, plant adpressed pubescent to glabrescent. Leaves petiolate, 1–2 × 0.4–05 cm, lanceolate-deltoid, acute, margin entire to slightly undulate, base shallowly cordate and auriculate, the auricles entire or bifid; petioles 3–8 mm long. Flowers solitary, pedunculate, axillary, becoming recurved in fruit; peduncles mostly 1–2 cm long; bracteoles filiform, 1–1.5 mm; pedicels 3–12 mm; outer sepals 2–3(-4) × 2–3 mm, obovate to broadly elliptic, rounded and minutely apiculate, scarious, glabrous or thinly appressed pubescent; inner sepals similar; corolla 5–7 mm long, white or pink, very shallowly lobed, midpetaline bands almost glabrous except for a few hairs at apex; ovary glabrous; style glabrous, divided c. 2 mm above base, stigmas c. 1.5–2 mm. Capsule glabrous; seeds coarsely and irregularly tuberculate. [Johnson 1987: 410–411, figure 1; Johnson 2001: 10, figs 3–4, map 1].

##### Distribution.

Australia: South Australia and adjacent New South Wales (*Orchard* 211, *Badman* 32, *Copley* 192; *Vonow* 584, *Mueller* 1852).

##### Notes.

Very distinctive because of the tiny calyx, lanceolate-deltoid leaves with basal auricles and the distinctive seed ornamentation.

#### 57. Convolvulus graminetinus

Taxon classificationPlantaeSolanalesConvolvulaceae

R.W.Johnson, Austrobaileya 6: 12. 2001. (Johnson 2001: 12).

##### Type.

AUSTRALIA, Queensland, *R.W.Johnson* 5300 (holotype BRI; isotypes CANB, K!, NE, NSW).

##### Description.

Perennial herb with trailing or twining stems to at least 50 cm, plant thinly pubescent to glabrescent. Leaves petiolate, dimorphic; petioles 2–10 mm; lowermost leaves 2.5–4 × 0.6–1.6 cm, deltoid, obtuse and finely mucronate, entire, base truncate and briefly cuneate onto the petiole, auricles absent; middle leaves similar but base more or less cordate, auricles present, often bifid or tridentate, the central lobe longer and narrower; middle and upper leaves with a narrowly linear-lanceolate central lobe 3–6 × 0.1–0. 6 cm, the basal auricles more or less reflexed so base sagittate, inconspicuous, bifid or trifid, 3–5 mm long, segments usually very narrow. Flowers solitary (very rarely paired), pedunculate, axillary; peduncles 1.2–3.5(-5.5) cm, recurved in fruit; bracteoles 1–2.5 mm, filiform; pedicels 4–12 mm; sepals 4–6 × 2.5–3 mm, obovate or elliptic, rounded and mucronate, scarious-margined, glabrous or pubescent on dorsal surface near apex; corolla 0.7–1.6 cm long, pink, shallowly lobed with triangular lobes, midpetaline bands pilose; ovary glabrous; style glabrous, divided 3–4 mm above base, stigmas 1.5–2 mm. Capsule glabrous; seeds with prominent wavy tubercles. [Johnson 2001: 12–14, figs3–4, map 2]

##### Distribution.

Australia: Northern Territory, Queensland and New South Wales (*Evans* 3248, *Must* 1510, *Johnson* 2075, *Hubbard* 3171, *McDonald* 46, *Clemens* s.n. [9/1945], *McBarron* 14875, *Melville* 3425). Reported from New Zealand (Heenan et al. 2003) but possibly extinct.

##### Notes.

Very immature plants could be confused with *Convolvulus arvensis* but the corolla is much smaller. More mature plants without lower leaves are similar morphologically to *Convolvulus remotus* but the fruiting peduncle is recurved. The strongly tuberculate seeds and the leaves with a very long narrow central lobe combined with the small bifid auricles are also rather distinct.

#### 58. Convolvulus remotus

Taxon classificationPlantaeSolanalesConvolvulaceae

R.Br., Prodr. Fl. Nov. Holland 483. 1810. (Brown 1810: 483)

Convolvulus preissii de Vriese, Pl. Preiss. 1: 346. 1845. (Lehmann 1845: 346). Type. WESTERN AUSTRALIA, Cape Riche, *Preiss* 1927 (holotype LD!).
Convolvulus huegelii de Vriese, Pl. Preiss. 1: 346 1845. (Lehmann 1845: 346).
Convolvulus remotus Type. WESTERN AUSTRALIA, Maddington, Canning River, *Preiss* 1928 (holotype LD, not seen).

##### Type.

AUSTRALIA, South Coast, Bay 10 (Port Lincoln) *R. Brown* 2766 (lectotype BM!, portion on left side of sheet, designated here; isotype K!, possible isotype MEL).

##### Description.

Perennial herb with twining or (occasionally) trailing stems to at least 50 cm, plant adpressed pubescent to more or less strigose. Leaves petiolate, not strongly dimorphic, 2.1–7.5 × 1–1.5 cm, narrowly deltoid, basally truncate and shortly cuneate onto the petiole, the central lobe linear, oblong, lanceolate or oblanceolate, acute, entire, 2–6 mm wide, basal auricles always present, 2–10 mm long, usually simple, occasionally bifurcate or toothed; petioles 6–10 (-20) mm. Flowers solitary or paired (rarely 3), pedunculate, axillary; peduncles mostly 1–3.5 cm long, not recurved in fruit; bracteoles 1.5–2.5 mm long, filiform; pedicels 4–10 mm; sepals 4.5–6 × 3–4.5 mm, broadly elliptic to obovate, rounded and mucronate at apex, margin somewhat scarious, dorsal surface pubescent; corolla 1–1.8 cm long, pink, lobed with broadly triangular lobes, midpetaline bands pilose towards apex; ovary glabrous; style glabrous, divided 4–6 mm above base, stigmas c. 2 mm. Capsule glabrous; seeds nearly smooth with obscure tubercles. [Johnson 2001: 14–15, f. 3–4, map 3]

##### Distribution.

Widespread in Australia, except the east coast, but most abundant in South Australia and Western Australia (*Chorney* 991, *Symon* 3578, *Aplin* 1792; *Lazarides & Palmer* 005; *Elkins & Sweedman* 20050042, *Rechinger* 58286).

##### Notes.

The usually very obviously twining stems with adpressed indumentum and narrowly deltoid auriculate leaves and straight peduncles serve to distinguish this species. The seeds are only obscurely tuberculate unlike those of *Convolvulus graminetinus*.

Robert Brown did not cite either a precise location or specimen in the protologue so Johnson was wrong to cite the Port Lincoln collection as holotype as there is another syntype mounted on the same sheet from a different location. In order to avoid future uncertainty we are formally designating the Port Lincoln collection at BM as lectotype. There is an isolectotype at Kew.

#### 59. Convolvulus crispifolius

Taxon classificationPlantaeSolanalesConvolvulaceae

F.Muell., Linnaea 25: 423. 1853. (Mueller 1853: 423).

Figure 9, t. 22–28

##### Type.

SOUTH AUSTRALIA, Cudnaka [Kanyaka], *Mueller* s.n. (holotype MEL; isotypes MEL, P!).

##### Description.

Perennial herb with trailing stems to 1 m, vegetative parts covered in long, whitish appressed velvety hairs, densely so on young growth. Leaves dimorphic: leaves of non-flowering shoots long-petiolate, 1–2 × 0.6–1.5 cm, ovate-deltoid, rounded, margin serrate, base truncate (very rarely slightly auriculate) and shortly cuneate onto the petiole, petiole 1–2 cm; leaves of flowering shoots 1–1.5(-2.5) × 0.5–1.4 cm, ovate-deltoid in outline but deeply toothed and incised towards the base; petioles 0.2–0.8 cm. Flowers solitary, pedunculate, axillary; peduncles 0.3–0.8 (-1.2 )cm, becoming recurved in fruit; bracteoles linear c. 1 mm; pedicels 1–3 mm; outer sepals 4 × 3.5 mm, elliptic, apiculate, the point recurved, sericeous; inner sepals similar but with fewer hairs; corolla 5–6 mm long, white or pink, lobed with somewhat triangular lobes, midpetaline bands sericeous; ovary glabrous, style glabrous, divided 2–2.5 mm above base; stigmas c. 1.5 mm. Capsule glabrous; seeds slightly winged and with low irregular sinuate ridges. [Johnson 2001: 15–19, f. 5–6, map 4]

##### Distribution.

Australia: Southern South Australia and adjacent parts of Victoria and New South Wales (*Alcock* 652, *Kuchel* 1470, *Chinnock* 2915, *Hill et al.* 5426). Johnson (2001) cites *Copley* 571 but this looks like an error for *Convolvulus eyreanus* as the peduncles are 1.5–2 cm long and mostly bear two flowers while the leaves are not so strongly sericeous.

##### Notes.

This species shares with *Convolvulus eyreanus* a dense indumentum of longish appressed hairs. Fruiting specimens can be recognised by the short recurved peduncles bearing single flowers, small capsules (4–4.5 mm in diameter) and small seeds (< 3 mm long).

#### 60. Convolvulus eyreanus

Taxon classificationPlantaeSolanalesConvolvulaceae

R.W.Johnson, Austrobaileya 2: 408. 1987. (Johnson 1987: 408).

##### Type.

AUSTRALIA, South Australia, *Donner* 3531 (holotype AD).

##### Description.

Perennial herb similar in general habit and distinctive features to *Convolvulus crispifolius* but generally larger: leaves on flowering shoots 0.7–4 × 5–2.5 cm, the basal half very incised-lobed almost to the midrib; peduncles commonly 2-flowered, (1-)1.2–2.9(-5) cm, rather tardily reflexing; pedicels 5–8 mm; outer sepals 4.5–6 × 3–4 mm, obovate-elliptic; corolla 6–8 mm long; ovary, style and capsule glabrous (or, fide Johnson, with a few hairs). Capsule 5–5.5 mm diameter, seeds 3.2–4.5 mm. [Johnson 2001: 19–20, f. 5–6, map 4]

##### Distribution.

Australia: northeastern South Australia and adjacent Queensland (*Whibley* 3455, *Filson* 3330, *Cornwall* 109, *O’Leary* 4541, *Weber* 8851).

#### 61. Convolvulus clementii

Taxon classificationPlantaeSolanalesConvolvulaceae

Domin, Biblioth. Bot. 89: 539. 1928. (Domin 1928: 539).

Comvolvulus clementii var. biflorus Domin, Biblioth. Bot. 89: 539. 1928. (Domin 1928: 539). Type. AUSTRALIA, Queensland, Dividing Range, Jericho, *Domin* (holotype PR, not seen).

##### Type.

AUSTRALIA, Western Australia, between Ashburton and Grey Rivers, *Clement* s.n. (holotype PR; isotype K!).

##### Description.

Perennial herb from a taproot with trailing (sometimes twining) stems to at least 75 cm, vegetative parts pubescent with adpressed or spreading hairs. Leaves petiolate, very plastic in shape, more or less trimorphic; petioles 0.3–3 cm, diminishing in length upwards; lowermost leaves 1–3 × 0.6–1.5 cm, deltoid, acute, margin undulate to crenate, base truncate and shortly cuneate onto the petiole; lower leaves sometimes distinct, 3–4 × 3–4 cm, deeply laciniate; middle leaves with elongate oblong undulate- or crenate-margined middle lobe, 2.5–3.5 × 0.4–0.8 cm and deeply lobed, more or less laciniate auricles; upper leaves with a very narrowly oblong central lobe 2–3.5 × 0.2–0.5 cm, two slightly shorter ascending lateral lobes and smaller bifurcate basal lobes. Flowers solitary or paired, pedunculate, axillary, not recurved in fruit; peduncles 0.6–3 cm; bracteoles 1–2 mm, filiform; pedicels 3–8 mm; sepals 4–5 × 2.5–4 mm, obovate or elliptic, acute or rounded, mucronate, pubescent; corolla 7–9 mm, white or pink, weakly lobed, midpetaline band pubescent; ovary and style glabrous; style divided 2–3 mm above base, stigmas c. 1.5 mm. Capsule glabrous, 4–6 mm in diameter; seeds winged, raised-tuberculate. [Johnson 2001: 35–37, f. 9–10, map 8]

##### Distribution.

Generally distributed in Australia but most common in central-western areas (*Hill* 167, *Macdonald* 435, *Newby* 10756, *Pedley* 914, *Jacobs* 2106).

##### Notes.

This species is extremly variable in leaf form and flowering specimens are indistinguishable from *Convolvulus recurvatus*.

#### 62. Convolvulus tedmoorei

Taxon classificationPlantaeSolanalesConvolvulaceae

R.W.Johnson, Austrobaileya 6: 37. 2001. (Johnson 2001: 37).

##### Type.

AUSTRALIA, New South Wales, *Moore* 5863 (holotype CANB; isotypes BRI, NSW).

##### Description.

A local endemic close to *Convolvulus clementii* but differing in its robust prostrate habit: stems stout almost to the apex, leaves up to 5 × 4 cm, capsules 6–7 mm wide, the seeds unwinged and rather large reaching 3.8 × 3.2 mm, more finely appressed tuberculate. The ripe capsules are suborbicular, 6–7 mm in diameter, conspicuously larger than in *Convolvulus clementii*. [Johnson 2001: 37–38, f. 9–10, map 5].

##### Distribution.

Australia: New South Wales (*Bates* 56321).

#### 63. Convolvulus recurvatus

Taxon classificationPlantaeSolanalesConvolvulaceae

R.W.Johnson, Austrobaileya 6: 32. 2001. (Johnson 2001: 32).

##### Type.

AUSTRALIA, South Australia, *Copley* 827 (holotype AD; isotype K!).

##### Description.

Perennial herb with trailing stems to at least 50 cm, vegetative parts sparsely to roughly pubescent with adpressed or spreading hairs. Leaves dimorphic, petiolate; petioles 5–20 mm; leaves of non-flowering shoots 1–2.5 × 0.5–0.9 cm, oblong-subrectangular, truncate at apex and base, sometimes cordate and weakly auriculate, margins coarsely dentate; leaves of flowering shoots 1.2–3 × 0.5–1.6 cm, ovate or lanceolate in outline but deeply incised lobed, characteristically with the terminal lobe prominent, linear or narrowly oblong, acute to emarginate, the margin undulate to coarsely dentate, basal part deeply bi-quadrilobed. Flowers solitary (rarely paired), pedunculate, axillary; peduncles 0.8–2.2 cm, becoming recurved in fruit; bracteoles c. 1 mm, linear; pedicels 2–6 mm; sepals 3–5 × 3–3.5 mm, obovate or elliptic, acute or rounded with a small recurved mucro; corolla 5–9 mm, white or pink, weakly lobed, midpetaline band pubescent; ovary and style glabrous; style divided 2 mm above base, stigmas 1–2 mm. Capsule glabrous, 4–4.5 mm in diameter; seeds obscurely winged and with irregular ridging. [Johnson 2001: 312–35, f. 9–10, map 5]

##### Notes.

We recognise two subspecies:

#### 63a. Convolvulus recurvatus subsp. recurvatus

Taxon classificationPlantaeSolanalesConvolvulaceae

##### Distinguishing features.

Larger in all its parts, the sepals 4–5 mm and corolla 7–9 mm.

##### Distribution.

Australia: eastern South Australia, New South Wales and Victoria (*Lothian* 2422, *Blaylock* 606, *Browne* 557).

#### 63b. Convolvulus recurvatus subsp. nullarborensis

Taxon classificationPlantaeSolanalesConvolvulaceae

R.W.Johnson, Austrobaileya 6: 33. 2001. (Johnson 2001: 33).

##### Type.

AUSTRALIA, South Australia, *Wilson* 1692 (holotype AD; isotypes BRI, MEL).

##### Distinguishing features.

A relatively distinctive subspecies because of the small corolla 5–7 mm in length and the very small calyx, the sepals 3–4 mm long. The leaves on the flowering stems are usually sparsely hairy and the central lobe is narrowly oblong with a distinctive emarginate apex.

##### Distribution.

Australia: South Australia and adjacent parts of Western Australia centred on the Nullabar plains (*Ising* 6/9/1920, *Ising* 1528, *Donner* 7216, *Crisp* 181, *Weber* 7921).

##### Notes.

*Convolvulus recurvatus* is very similar to *Convolvulus clementii* differing only in the recurved fruiting peduncles and the more strongly tuberculate seeds. Flowering specimens cannot be safely distinguished.

#### 64. Convolvulus wimmerensis

Taxon classificationPlantaeSolanalesConvolvulaceae

R.W.Johnson, Austrobaileya 6: 22. 2001. (Johnson 2001: 22).

##### Type.

AUSTRALIA, Victoria, *Beauglehole* 82670 (holotype MEL).

##### Description.

Perennial herb with densely adpressed pubescent, trailing or twining stems, similar in facies to *Convolvulus recurvatus* with which it shares the distinctive recurved fruiting peduncle. It differs principally in the larger corolla, c. 0.9–1.2 cm long. The leaves are strongly dimorphic, the basal leaves triangular-ovate with undulate margin and poorly developed spreading, obtuse auricles. Flowers 1–2; corolla pink; seeds unwinged. [Johnson 2001: 22–23, f. 5–6, map 5]

##### Distribution.

Australia: Victoria and New South Wales (*Jeanes* 1703, *Harvey* 1854, *Mueller* 1868).

##### Notes.

This species occupies a situation somewhat intermediate both geographically and morphologically between *Convolvulus recurvatus* and *Convolvulus angustissimus* and might just be of hybrid origin.

#### 65. Convolvulus erubescens

Taxon classificationPlantaeSolanalesConvolvulaceae

Sims, Bot. Mag. t. 1067. 1807. (Sims 1807: t. 1067).

##### Type.

Plate in Bot. Mag. t.1007 (1807), lectotype, designated here; AUSTRALIA, plant from Hawkesbury River, New South Wales, collected by R. Brown on left side of sheet *Brown* s.n. [Bennett 2767] (epitype BM!, designated here).

##### Description.

Perennial herb with trailing or twining stems reaching at least 50 cm, stems crisped-pubescent, stouter than in other Australian species, commonly exceeding 2 mm in width. Leaves petiolate, variable in size but not markedly dimorphic, 2.5–8 × 1–3.5 cm, deltoid, apex obtuse and mucronate, margin crenate or repand, base broadly cordate and cuneate onto the petiole with prominent auricles, these variable, simple, toothed or laciniately lobed; petioles 1.2–2.5 cm, diminishing in size upwards. Flowers 1–4, usually clearly cymosely arranged, axillary, pedunculate, not recurved in fruit; peduncles 1–2 per axil, 2–6 cm, usually straight; bracteoles 1–3 mm long, filiform; pedicels 8–25 mm, very variable in length and strikingly unequal in individual inflorescences, often sinuate; sepals 5.5–7 × 3.5–5 cm. narrowly elliptic, terminating in a recurved mucro; corolla 1.2–1.5 cm, pinkish, lobed with triangular lobes, midpetaline bans pubescent near apex; ovary glabrous; style glabrous, divided 3–7 mm above the base, stigma 2 mm. Capsule glabrous, seeds tuberculate, unwinged. [Johnson 2001: 20–22, f. 5–6, map 2]

##### Distribution.

Australia: eastern coast of New South Wales and Queensland (*Beckler* s.n., *McBarron* 4110, *Coveny* 11781, *Mossman* 1854), apparently rather rare.

##### Notes.

The most robust Australian species, the peduncles usually bearing several flowers and unusually sometimes with two peduncles per leaf axil. Most similar to *Convolvulus clementii* but leaves more obviously deltoid in form, the corolla larger and the seeds unwinged. *Johnson* 3364 (P) from Northern Territory will key out here (unwinged fruit, paired 2-flowered cymes) but flowers and location fit *Convolvulus clementii*.

All Australian and New Zealand native species were once treated under this name following Bentham (1869).

#### 66. Convolvulus angustissimus

Taxon classificationPlantaeSolanalesConvolvulaceae

R.Br., Prodr. Fl. Nov. Holland 482. 1810. (Brown 1810: 482).

Figure 9, t. 37–43

Convolvulus geniculatus Lehm., Index Seminarum (HBG) 1826: 17. 1826. (Lehmann 1826: 17). Type. None cited.
Convolvulus acaulis Choisy, Prodr. [A.P. de Candolle] 9: 406. 1845. (Choisy 1845: 406). Type. SOUTH AUSTRALIA, Kangaroo Island, coll. not known (holotype P).
Convolvulus erubescens var. angustissimus (R.Br.) Choisy, Prodr. [A.P. de Candolle] 9: 412. 1845. (Choisy 1845: 412). Type. Based on *Convolvulus angustissimus* R.Br.
Convolvulus adscendens de Vriese, Pl. Preiss 1: 346. 1845. (Lehmann 1845: 346). Type. WESTERN AUSTRALIA, York District, *Preiss* 1924 (holotype LD; isotypes MEL 689918, MEL689919).
Convolvulus ascendens Walp., Repert. Bot. Syst. 6: 540. 1846, lapsus [spelling mistake] for *Convolvulus adscendens* de Vriese (1845). (Walpers 1846: 540).
Convolvulus subpinnatifidus de Vriese, Pl. Preiss 1: 346. 1845. (Lehmann 1845: 346). Type. AUSTRALIA, Beljarup, Hay, *Preiss* 1925 (holotype LD!; isotypes MEL689916, MEL689917).
Convolvulus erubescens var. fililobus Wawra, Itin. Princ. S. Coburgi 1: 102. 1883. (Wawra 1883: 102). Type. AUSTRALIA, Victoria, *Wawra* 438. (holotype W!).
Convolvulus erubescens var. albus Guilf., Austral. Pl. Gard. 117. 1911. (Guilfoyle 1911: 117). Type. AUSTRALIA, no type specified.
Convolvulus angustissimus subsp. fililobus (Wawra) R.W.Johnson, Austrobaileya 6: 30. 2001. (Johnson 2001: 30). Type. Based on Convolvulus erubescens var. fililobus Wawra
Convolvulus angustissmus subsp. omnigracilis R.W.Johnson, Austrobaileya 6: 27. 2001. (Johnson 2001: 27). Type. AUSTRALIA, Victoria, *Forbes & Scarlett* 1867 (holotype MEL; isotype BRI).
Convolvulus angustissimus subsp. peninsularum R.W.Johnson, Austrobaileya 6: 31. 2001. (Johnson 2001: 31). Type. AUSTRALIA, South Australia, *Alcock* 4733 (holotype AD; isotype SYD).

##### Type.

AUSTRALIA, Tasmania, “Van Dieman’s Land near Risdon Cove” *R. Brown* s.n. [Bennett 2765] (lectotype BM!, portion on right side of sheet, designated here, isolectotype K!, also perhaps MEL).

##### Description.

Perennial herb with trailing or twining stems, pubescent to subglabrous to at least 40 cm but commonly short. Leaves extremely variable and often di/trimorphic on the same plant, petiolate, petioles 0.5–7 cm, diminishing in length upwards; lowermost leaves (if present) 1–2 × 0.3–1.5 cm, ovate-deltoid, obtuse, margin entire, undulate or sinuate-lobed especially towards the base, base cordate or truncate, the auricles poorly developed, entire to bi(tri-)fid; lower stem leaves 2–3 (-6) × 1.3–5 cm, broadly or narrowly ovate-deltoid in outline, entire undulate or deeply sinuate lobed, the basal auricle prominent, lobed with a short ascending lobe; upper stem leaves usually finely lobed, the central lobe linear-oblong, mostly 2.5–4.5 × 0.1–0.3 cm, acute or apiculate; auricles usually with a prominent ascending lobe resembling the terminal lobe but half its length together with short bifid reflexed lobes. Flowers pedunculate, axillary, usually solitary; peduncles 0.5–5 cm long, becoming recurved in fruit; bracteoles filiform, 1–2 mm long; pedicels 3–20 mm; sepals 4–7 × 2–2.5 mm, elliptic, rounded or acute, minutely mucronate, inner sepals slightly smaller; corolla 1.2–2 cm long, usually pink, weakly lobed, midpetaline bands pubescent only near apex; ovary glabrous; style glabrous, divided 3–10 mm above the base, stigmas 1–2 mm. Capsule glabrous; seeds 3–4 mm long, covered in low reticulate ridges. [Johnson 2001: 23 -32, f. 7–8, map 7]

##### Distribution.

Principally southeastern Australia: Tasmania, Victoria, New South Wales, South Australia, Queensland, Western Australia (*Milligan* 90, *Scarlett* 83-395, *Aston* 2367, *Hoogland* 3078, *Lea* 1885, *Coveny & Hind* 11502, *Jeanes* 2084, *Tilden* 723, *Coomber* 2215, *Gunn* 721).

##### Notes.

Johnson’s (2001) division of this species into four subspecies is not satisfactory. In the first place the type subspecies is a plant whose leaves have entirely linear segments quite unlike the subsp. angustissimus illustrated by Johnson (2001: 28). There are in fact quite a lot of specimens which accord well with Robert Brown’s type collection including *Wawra* 438, the type of var. fililobus and *Morrison* 1445, *Robertson* 221, *Constable* 56058 and *Burns* 7. The second difficulty with Johnson’s infraspecific classification is that the majority of specimens we have seen are not accommodated satisfactorily in any one or other of his subspecies. Specimens which accord with a particular subspecies can be recognised but a large residue which fit none remains. In this account, therefore, *Convolvulus angustissimus* is treated as a single variable species without recognised subspecies, usually easily identified by its relatively large corolla, 1.2–2 cm long. However, specimens from Western Australia and South Australia have smaller corollas and seem to intergrade with *Convolvulus remotus*. Examples of specimens showing this introgression include *Lea* 26/9/1885, *Andrews* 657, *Black* 12, *Koch* 5/1898.

#### 67. Convolvulus fractosaxosus

Taxon classificationPlantaeSolanalesConvolvulaceae

Petrie, Trans & Proc. New Zealand Inst. 45: 271. 1913 [1912]. (Petrie 1913: 271).

##### Type.

NEW ZEALAND, South Island, *Cockayne* s.n. (holotype WELT-4828).

##### Description.

Greyish-pubescent creeping herb arising from underground rhizome; stems to 30 cm. Leaves petiolate, 1.5–3.6 × 0.2–0.8 cm, dimorphic and very variable, mostly deltoid or ovate, sometimes hastate and always with some leaves with an oblong or linear terminal lobe 1–5 cm long, combined with small basal auricles arising at right angles to the terminal lobe; petioles 1–5 cm. Flowers solitary, axillary, pedunculate; peduncles 2–6 cm long, 1-flowered, slender, pubescent; bracteoles 2–3 mm long, linear; pedicels 2–6 mm, pubescent; outer sepals 6–8 × 5–6 mm, broadly ovate, pubescent, larger than inner sepals; corolla 1.7–2 cm long, white, midpetaline bands pink; ovary glabrous; style glabrous, divided 6–7 mm above base. Capsule glabrous; seeds finely tuberculate. [Moore and Irwin 1978: 157]

##### Distribution.

New Zealand: South Island (*Travers* 1864, *Hombron* 1841), 300–1600 m.

##### Notes.

Similar to forms of the Australian *Convolvulus angustissimus* found in Tasmania and Victoria but corolla and sepals slightly longer.

#### 68. Convolvulus verecundus

Taxon classificationPlantaeSolanalesConvolvulaceae

Allan, Fl. N. Zeal. 1: 967. 1961. (Allan 1961: 967).

##### Type.

NEW ZEALAND, South Island, *A.W. Anderson* 15 Jan 1941 (holotype CHR 76122).

##### Description.

Perennial herb from an underground rhizome, stems decumbent and trailing to ascending up to 20 cm long, thinly pubescent on vegetative parts. Leaves petiolate, 6.5–11.5 × 5–12.5 mm, deltoid, ovate or broadly oblong, always lacking basal auricles, retuse or obtuse at apex, margin undulate, base *more or less* truncate and shortly cuneate; petioles 1.5–2 cm. peduncles 1–3 cm, 1-flowered, very slender; bracteoles 1–3 mm, linear; pedicels 2–6 mm, pubescent; sepals 4 × 3.5–3.8 cm, obovate or broadly oblong, margin fimbriate and translucent, abaxially pubescent; corolla 18–19 mm, white or pink, unlobed, midpetaline bands pink, pubescent upwards; ovary glabrous; style glabrous, divided 5–8 mm above base, stigmas 1.5–2 mm, unequal. Capsule glabrous, seeds glabrous, covered in low ridges and tubercles.

##### Distribution.

New Zealand: South Island (*Cockayne* 2370), 200–1000 m, always inland.

#### 69. Convolvulus waitaha

Taxon classificationPlantaeSolanalesConvolvulaceae

(Sykes) Heenan, Molloy & de Lange, New Zealand J. Bot. 41: 450. 2003. (Heenan et al. 2003: 450).

Convolvulus verecundus subsp. waitaha Sykes, New Zealand J. Bot. 25: 154. 1987. (Connor and Edgar 1987: 153-154). Type. NEW ZEALAND, *Melville et al.* 5059 (holotype CHR-129450; isotype K!).

##### Type.

Based on Convolvulus verecundus subsp. waitaha Sykes

##### Description.

Similar in overall morphology to *Convolvulus verecundus* but a more vigorous plant with stems to 80 cm differing in being almost completely glabrous to thinly pubescent, the corolla smaller (8–13 mm long) with greenish, glabrous midpetaline bands, the undivided part of the style < 3 mm long and the seeds more prominently tuberculed and ridged. The fruiting peduncles are recurved but no fruiting material of *Convolvulus verecundus* has been seen.

##### Distribution.

New Zealand: North and South Islands (*Colenso* 131, *Melville* 5714, *Douglass* 65208, *Védel* 1847). Mostly coastal, 0–500 m in moister areas than other species.

### Species 70–85. Old World species with petiolate leaves not markedly hastate or sagittate at base

This is a morphologically and geographically heterogeneous group formed from Clade B (Figure 1) which includes the spiny shrub *Convolvulus leiocalycinus*, the submaritime *Convolvulus persicus*, the lianas from Macaronesia and a number of mostly trailing undershrubs. The only morphological character holding the group together, albeit weakly, is that the petiolate leaves show a tendency to be cuneate, rounded or truncate at the base, rather than hastate or sagittate. Species 75–81 form a relatively distinct subgroup which possesses a distinctly conical ovary and a robust, often liana-like habit. They are restricted to Madeira, the Canary Islands and Portugal.

#### 70. Convolvulus leiocalycinus

Taxon classificationPlantaeSolanalesConvolvulaceae

Boiss., Diagn. Pl. Orient. 7: 28. 1846. (Boissier 1846: 28).

Figure 10, t. 1–8

##### Type.

IRAN, “In rupestris apricis. inter Abuschir et Schiras”, *Kotschy* 39 (lectotype G, designated by Sa’ad. 1967: 67); isolectotypes E!, GOET, K!, OXF!, P!, W!).

##### Description.

Intricately branched spiny shrub reaching 1 m in height; branches woody, finely appressed sericeous, small stem spines present. Leaves shortly petiolate, 1–2.4 × 0.5–0.9 cm, variable in shape, oblong, lanceolate, ovate or suborbicular, acute, entire, base cuneate, truncate or auriculate, glabrous, pubescent or sericeous; petioles 2–4 mm. Flowers axillary, pedunculate, solitary; peduncle 1–10 mm, stout, woody; bracteoles minute, c. 1 mm, squamose, caducous or absent; pedicels 2–7 mm, compressed, finely pubescent, often not differentiated from peduncle; sepals lax, somewhat scarious, 4–7 × 2.5–5 mm, ovate or broadly elliptic to obovate, obtuse or acute, obviously veined or not, somewhat scarious, becoming more or less erect and adpressed to capsule in fruit or spreading or reflexed; corolla 2–3 cm long, white or pinkish, unlobed, midpetaline bands pilose near the tips only; filaments glandular below; ovary pubescent or glabrous; style glabrous, divided c. 10 mm above the base, stigmas c. 2 mm. Capsule glabrous; seeds glabrous, smooth. [Sa’ad 1967: 67; Austin and Ghazanfar 1979: 10; Petrov 1935: 133 (plate), Nowroozi 2002: 19 (plate), 100 (map); Breckle and Rafiqpoor 2010: 417 (photo)]

**Figure 10.**
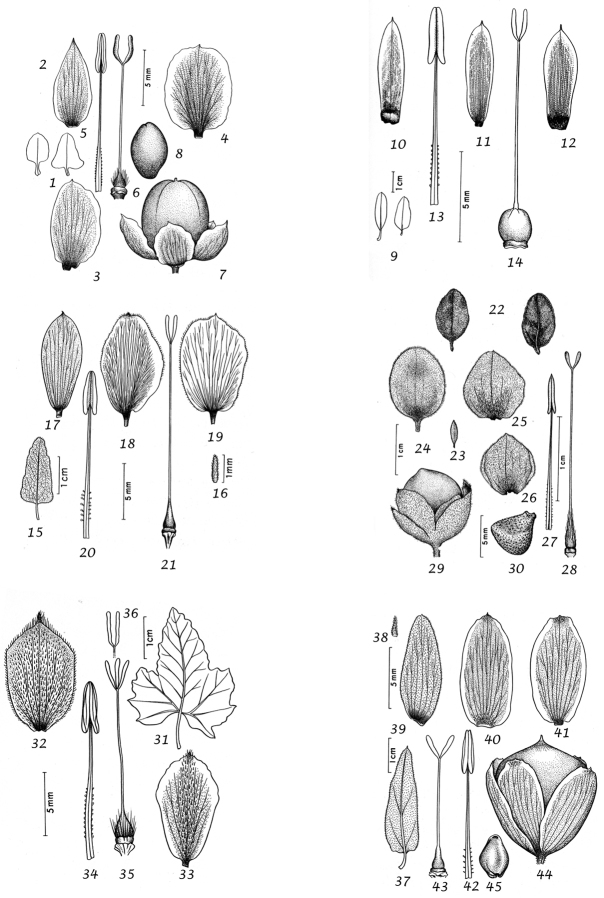
**1–8** Convolvulus leiocalycinus var. leiocalycinus **1** leaves **2** outer sepal **3** middle sepal **4** inner sepal **5** stamen **6** ovary and style **7** capsule **8** seed **1–2** from *Kotschy* **39** (W) **3–6** from *Strauss* s.n. (W) **7–8** from *Bornműller* 3884 (B) **9–14** Convolvulus leiocalycinus var. retrosepalus **9** leaves **10** outer sepal **11** middle sepal **12** inner sepal **13** stamen **14** ovary and style. From *Lindberg* 317 (W) **15–21** *Convolvulus dryadum* **15** leaf **16** bracteole **17** outer sepal **18** middle sepal **19** inner sepal **20** stamen **21** ovary and style. From *Sennen & Mauricio* 9471 (W) **22–30** *Convolvulus persicus* **22** leaves **23** bracteole **24** outer sepal **25** middle sepal **26** inner sepal **27** stamen **28** ovary and style **29** capsule **30** seed. **22** from *Dubiansky* s.n. (W) **23–28** from *Aznavour* s.n. (W) **29–30** from *Szovitz* s.n. (W) **31–36** *Convolvulus maireanus* **31** leaf **32** outer sepal **33** inner sepal **34** stamen **35** ovary and style with 3 stigmas **36** apex of style with 2 stigmas. From *Pampanini* 6206 (G) **37–45** *Convolvulus lanjouwii* **37** leaf **38** bracteole **39** outer sepal **40** middle sepal **41** inner sepal **42** stamen **43** ovary and style **44** capsule **45** seed. From *Griffith* 678 (K).

##### Notes.

*Convolvulus leiocalycinus* is the only spiny species in Central Asia with leaves abruptly narrowed at the base into a distinct petiole. Another unusual feature is the lax sepals which are not appressed to the base of the corolla. It is a variable species in many details but always with a common facies. Variety glaber was described on the basis of its glabrous leaves, *Convolvulus olgae* on its sericeous leaves and *Convolvulus lycioides* on its oblong leaves but the species, in fact, shows a wide range of leaf shape and indumentum with no obvious geographical patterning. We recognise two varieties.

#### 70a. Convolvulus leiocalycinus Boiss. var. leiocalycinus

Taxon classificationPlantaeSolanalesConvolvulaceae

Convolvulus lycioides Boiss., Diagn. Pl. Orient. 7: 28. 1846. (Boissier 1846: 29). Type. IRAN, “In collibus calcareis inter Abuschir et Schiras”, *Kotschy* 39 (holotype G; isotypes K!, OXF!, P!).
Convolvulus lasiophlaeus Jaub. & Spach, Illustr. Plant Or. 4: 105, t. 368. 1852. (Jaubert and Spach 1852: 105).
Convolvulus leiocalycinus Boiss. var. leiocalycinus Type. IRAN, Shiraz, *Aucher-Eloy* 4935 (holotype P).
Convolvulus leicalycinus var. stocksii Boiss., Diagn. Pl. Or. Nov. 2(3): 123. 1856. (Boissier 1856: 123). Type. IRAN, Shiraz, *Aucher-Eloy* 4935 (lectotype G, designated by Sa’ad 1967: 68).
Convolvulus leicalycinus var. lycioides (Boiss.) Boiss., Fl. Orient. [Boissier] 4: 86. 1875. (Boissier 1875b: 86). Type. Based on *Convolvulus lycioides* Boiss.
Convolvulus olgae Regel & Schmalh., Izv. Imp. Obsc. Ljubit. Estesv. Moskovsk. Univ. 34(2): 55. 1882. (Regel 1882: 55). Type. TAJIKISTAN, Mount Daschti Kasi, *Fedstchenko* 31/5/1869 (holotype LE!).
Convolvulus campanulatus Zapr., Trans. Tajikst. Acad. Sc. 1: 73. 1933, nom. illeg., non *Convolvulus campanulatus* Spreng. (1825). (Zaprjagiev 1933: 73). Type. TAJIKISTAN, Mount Karatau, *Zaprjagaev* 55 (location not certainly known ? TAK).
Convolvulus leiocalycinus var. glaber Ghaz., Fl. W. Pakistan 126: 11. 1979. (Austin and Ghazanfar 1979: 126). Type. PAKISTAN, Balochistan, *Sultan ul Abedin* 4893 (holotype KUH, not seen).

##### Distinguishing features.

Characterised by the lanceolate to ovate or obovate outer sepals 4–7 × 2.5–5 mm wide, these patent to erect in fruit. Leaves and ovary glabrous or hirsute.

##### Distribution.

Tajikistan (*Varivtseva* 209, *Botchantsev & Egorova* 690, *M.Popov* 932); Iran (*Bornműller* 3884b, *Stapf* 2325, *Edmondson & Miller* 1547, *Davis & Bokhari* 56083); Afghanistan (*Rechinger* 33380, 3875); Pakistan (*Duthie* 18841, *Stocks* 870, *Lamond* 710); Oman (*McLeish* 3736).

#### 70b. Convolvulus leiocalycinus var. retrosepalus

Taxon classificationPlantaeSolanalesConvolvulaceae

(Sa’ad) J.R.I.Wood & R.W.Scotland stat. nov.

Figure 10, t. 9–14

Convolvulus retrosepalus Sa’ad, Meded. Bot. Mus. Herb. Rijks Univ. Utrecht 281: 69. 1967. (Sa’ad 1967: 69).

##### Type.

AFGHANISTAN, *Lindberg* 317 (holotype W!).

##### Distinguishing features.

This differs from var. leiocalycinus by the narrowly lanceolate, reflexed sepals, c. 7 × 1 mm, the veins usually conspicuous. The leaves and ovary are glabrous.

##### Distribution.

Restricted to Afghanistan, Farah Province (*Hedge & Ekberg* 7252, *Hedge et al.* 7739, *Grey-Wilson & Hewer* 557, *Carter* 824).

##### Note.

Intermediates with var. leiocalycinus occur quite frequently (*Grey-Wilson & Hewer* 534, *Podlech* 21729, *Rechinger* 33380, *Koie* 4414). These are similar to var. retrosepalus in their lack of hairs but the sepals are obovate and spreading.

#### 71. Convolvulus dryadum

Taxon classificationPlantaeSolanalesConvolvulaceae

Maire, Bull. Soc. Bot. France 60: 253. 1913. (Maire 1913: 253).

Figure 10, t. 15–21

##### Type.

ALGERIA, Djebel Babor, *Maire* s.n. (holotype AL?; isotypes MPU005286!, P00417695!, P00417696!).

##### Description.

Perennial herb from a rhizomatous rootstock with branched trailing stems to at least 40 cm; stem and vegetative plants adpressed pubescent or (rarely) glabrous. Leaves petiolate, 2–5 × 1.5–3 cm, ovate-deltoid, apex obtuse, margin undulate to sinuate, base truncate to very shallowly cordate; petioles 0.3–0.8(-1.5) cm. Flowers solitary, axillary, pedunculate; peduncles 4–9 cm, often bent at apex; bracteoles 1–2 mm, filiform; pedicels 5–7 mm; outer sepals 9–10 × 4–5 mm, broadly elliptic, mucronate; inner sepals slightly larger c. 11 × 6 mm, somewhat scarious; corolla 2.5–3.1 (-4.5) cm long, white with dark centre, midpetaline bands pink, pubescent near the apex, unlobed but margin slightly undulate; filaments glandular; ovary glabrous, conical; style glabrous, divided c. 12–14 mm above base; stigmas 2–3 mm, stout. Capsule and seed not known. [Sa’ad 1967: 223]

##### Distribution.

Djebel Tazekka and Rif Mountains of Morocco (*Sennen & Mauricio* 9471, *Davis* 54878, *Ait Lafkih et al.* 127) and Algeria.

##### Notes.

Distinctive because of the long pedunculate, solitary flowers, triangular, basally more or less truncate leaves and very long style.

A distinctive variety was described from Djebel Tazekka, an isolated massif in Morocco, by Sauvage and Vindt (1954: 31) based on the glabrous or subglabrous leaves and large corolla up to 4.5 cm in length. This “var. tazekkensis” (based on *Guinet et al.* 68) was never validly published but merits further investigation.

#### 72. Convolvulus persicus

Taxon classificationPlantaeSolanalesConvolvulaceae

L., Sp. Pl. 1: 158. 1753. (Linnaeus 1753: 158).

Figure 10, t. 22–30

##### Type.

TURKEY, Constantinople, *G. V. Aznavour* (neotype BM!, designated by Staples in Staples and Jarvis 2006: 1021); isoneotypes B, E!, P!).

##### Description.

Perennial branched undershrub with woody rootstock and lower branches, the whole plant shortly tomentose; stems very stout, 3–5 mm thick. Leaves petiolate, coriaceous, 2–5 × 1–3.5 cm, elliptic, oblong-elliptic or obovate, apex obtuse to rounded, margin entire, base broadly cuneate to rounded, slightly asymmetric; petioles 0–6 cm. Flowers solitary, axillary, pedunculate, usually arising from the middle part of the stem; peduncles 0.8–3.3 cm; bracteoles 3–5 × 1–3 mm, ovate to oblong-elliptic, acute; pedicels 5–7 mm; sepals 12–15 × 7–9 mm, ovate, obtuse or acute; corolla 3–4.5 cm, white, unlobed, undulate, midpetaline bands pilose; filaments glandular below; ovary very narrow, thinly pilose at apex; style divided c. 18 mm above base; stigmas 2 mm, narrowly elliptic. Capsule glabrous, seeds tuberculate. [Sa’ad 1967: 163; Nowrooze 2002: 103 (map); Grigoriev 1953: 11 (plate)]

##### Distribution.

Coasts of the Black Sea: Georgia (*Cosson* 8 in Hohenacker), Turkey (*Olivier & Brugière* s.n., *Uslu* 2141), Bulgaria (*Bosseva et al*. 88), Romania (*Grintescu* 1094b) and Caspian Sea: Russia [Daghestan] (*Becker* 1876), Azerbaijan (*Lewandowsky* 519, *Kerimov* 32), Iran (*Aucher-Eloy* 4940), Turkmenistan (*Dubiansky* s.n. [14/7/1932]). Coastal sand dunes.

##### Notes.

A very distinct, apparently isolated species.

#### 73. Convolvulus maireanus

Taxon classificationPlantaeSolanalesConvolvulaceae

Pamp., Arch. Bot. (Forlì) 12: 178. 1936. (Pampanini 1936b: 178).

Figure 10, t. 31–36

##### Type.

LIBYA, Cyrenaica, “Messe a ovesti di Cirene Sfonta–Ruheina, 8 Mag 1934,” *Pampanini & Pichi-Sermolli* 6207 (syntype FI)

##### Description.

Perennial trailing, scrambling or twining herb with puberulent, sharply 4-angled to weakly winged stems of “a considerable height”, presumably 1–2 m. Leaves petiolate, somewhat dimorphic, 2.5–7 × 2–5.5 cm, younger leaves ovate, apex acute to obtuse, margin undulate to irregularly dentate with some large teeth, base cordate and briefly cuneate with a broad right-angled sinus, minutely adpressed pubescent; mature leaves more deltoid, deeply lobed with acute segments in the lower part, strongly auriculate; petioles 0.5–2 cm, puberulent. Flowers in a terminal cluster of up to 7 at the apex of a long axillary peduncle; peduncles 8–22 cm, pubescent; bracteoles filiform to linear, 4–8 mm, pubescent; pedicels 3–8 mm, pubescent; outer sepals 8–9 × 5–6 cm, broadly obovate-elliptic, mucronate, pubescent; inner sepals 4–5 cm wide,less hirsute and with scarious margins; corolla 2.8–4.5 cm, white with purplish throat, unlobed, basal tube narrow, midpetaline bands thinly adpressed pubescent; filaments glandular; ovary pilose; style divided c. 9 mm above base; stigmas 2 or 3, 2 mm long, rather stout. Capsule and seeds not seen. [Sa’ad 1967: 234]

##### Distribution.

Libya: Cyrenaica (*Sandwith* 2625, *Park* 506, *Pampannini & Pichi-Sermolli* 6206).

##### Notes.

A remarkable species with flowers clustered at the apex of a very long peduncle. The stigmas are short and stout and commonly 3 in number.

#### 74. Convolvulus lanjouwii

Taxon classificationPlantaeSolanalesConvolvulaceae

Sa’ad, Meded. Bot. Mus. Herb. Rijks Univ. Utrecht 281: 232. 1967. (Sa’ad 1967: 232).

Figure 10, t. 37–45

##### Type.

AFGHANISTAN, *Griffith* 678/K.D. 5872 (holotype K!; isotype P!).

##### Description.

Trailing or scrambling herb, probably branched at base with stems to at least 40 cm, all vegetative parts densely pubescent to tomentose. Leaves petiolate, 2.2–3.5 × 0.7–1.5 cm, ovate-deltoid, apex acute, margin entire, base truncate; petioles 4–5 mm. Flowers up to 3 in pedunculate, axillary cymes, but often reduced to 1–2; peduncles 2.5–5 cm; bracteoles 2–3 mm, linear-lanceolate; pedicels 3–6 mm; outer sepals 9–10 × 4–4.5 mm, broadly oblong-rectangular, obtuse to truncate and mucronate, inner sepals scarious-margined and subglabrous; corolla 2.5–2.7 cm, colour unknown, unlobed, midpetaline bands thin, pilose; filaments glandular near base; ovary glabrous; style glabrous, divided c. 6 mm above base, stigmas 2 mm, relatively stout. Capsule glabrous, seeds minutely hirsute.

##### Distribution.

Afghanistan–only known from the type collected at “Seh Baba”.

##### Notes.

This species and *Convolvulus rectangularis* have unique, distinct, broadly oblong-rectangular sepals.

#### 75. Convolvulus rectangularis

Taxon classificationPlantaeSolanalesConvolvulaceae

Rech.f., Biol. Skr. 10(3): 81. 1959. (Rechinger 1959: 81).

##### Type.

AFGHANISTAN, *Volk* 1018 (holotype W!).

##### Description.

Perennial herb with woody rootstock from which arise many wiry, probably scrambling stems to at least 25 cm; stems striate, glabrescent. Leaves very shortly petiolate, 1–3.5 × 0.4–0.6 cm, lanceolate-deltoid, acute, margin entire to sinuate-lobed, base truncate to auriculate, shortly crisped pubescent when young, glabrescent; petioles 1–3 mm. Flowers 1–3, borne on axillary peduncles; peduncles 1–1.5 cm; bracteoles 2.5 mm, filiform; pedicels 4–10 mm; outer sepals 9–10 × 6 mm, broadly oblong-rectangular, truncate and minutely mucronate, margin scarious, purplish, pubescent and with ciliate margins, inner sepals oblong, c. 4 mm wide; corolla 2.3–3.2 cm long, pinkish, midpetaline bands pubescent; filaments glandular below; ovary glabrous, style glabrous, divided c. 7 mm above base; stigmas not seen. Capsule glabrous; seeds wrinkled. [Sa’ad 1967: 104; Rechinger 1963: 17]

##### Distribution.

Afghanistan (*Rechinger* 35902, 37103).

##### Notes.

Although placed as distantly related in different sections by Sa’ad, *Convolvulus rectangularis* and *Convolvulus lanjowii* are quite close and might prove to be conspecific. The description above is based on the two widely distributed Rechinger collections cited above. These differ clearly in their sparse indumentum and in the sinuate-lobed narrower leaves with a rather rigid texture from *Convolvulus lanjouwii*. However the type and *Amsel* s.n. (W) are much more hirsute and with unlobed leaves so approaching *Convolvulus lanjouwii*. Further collections are needed to confirm whether two distinct species are really involved.

#### 76. Convolvulus massonii

Taxon classificationPlantaeSolanalesConvolvulaceae

F. Dietr., Nachtr. Vollst. Lex. Gärtn. 2: 377. 1816. (Dietrich 1816: 377).

Figure 11, t. 1–7

Convolvulus saxatilis Salisb., Prodr. Stirp. Chap. Allerton 124 (1796), nom. illeg., non *Convolvulus saxatilis*Vahl (1794). (Salisbury 1796: 124). Type. MADEIRA, *Masson* s.n. (probably BM000839753).
Convolvulus suffruticosus Dryand., Hort. Kew ed. 2 [W.T Aiton] 1: 331. 1810, nom. illeg., non *Convolvulus suffruticosus* Desf. (1798). (Aiton 1810: 331).
Convolvulus massonii Type. MADEIRA, *Masson* s.n. (holotype BM000839753!).
Convolvulus solanifolius Lowe, Trans. Cambridge Philos. Soc. 4: 22. 1831, nom. illeg., non *Convolvulus solanifolius* Spreng. (1824). (Lowe 1831: 22). Type. MADEIRA, *Lowe* (whereabouts uncertain).
Bucharea maderensis Raf., Fl. Tellur. 4: 84. 1838. (Rafinesque 1838: 84). Type. Based on *Convolvulus suffruticosus* Dryand. ex W.T.Aiton.
Convolvulus canariensis var. massonii (F. Dietrich) Sa’ad, Meded. Bot. Mus. Herb. Rijks Univ. Utrecht 281: 250.1967. (Sa’ad 1967: 250). Type. Based on *Convolvulus massonii* F. Dietr.

##### Type.

Based on *Convolvulus suffruticosus* Dryand.

##### Description.

Perennial undershrub with trailing or twining stems to 4 m, becoming woody below with age, the whole plant glabrous to thinly adpressed pilose on younger parts. Leaves petiolate, 4–11 × 1.5–6 cm, ovate, acute to shortly acuminate, entire, truncate to cordate at the base, more or less reticulate veined on abaxial surface, glabrous or nearly so; petioles 1.5–4 cm. Flowers 1–6, in very lax, pedunculate, axillary cymes; peduncles 3–6 cm; bracteoles 14 × 0.5–1 mm, filiform to linear; pedicels 15–30 mm; sepals 9–15 × 4–7 mm, obovate or oblanceolate, with a triangular apiculate apex; corolla 2–2.5 cm, white, unlobed, midpetaline bands pink, pilose; filaments glandular; ovary pubescent at apex, style pubescent, divided 4 mm above the base, stigmas 4 mm. Capsule large, acute c. 6 mm long, glabrous; seeds (immature), smooth, with pale reticulation. [Jardim and Francisco 2000: 134 (photo)]

**Figure 11.**
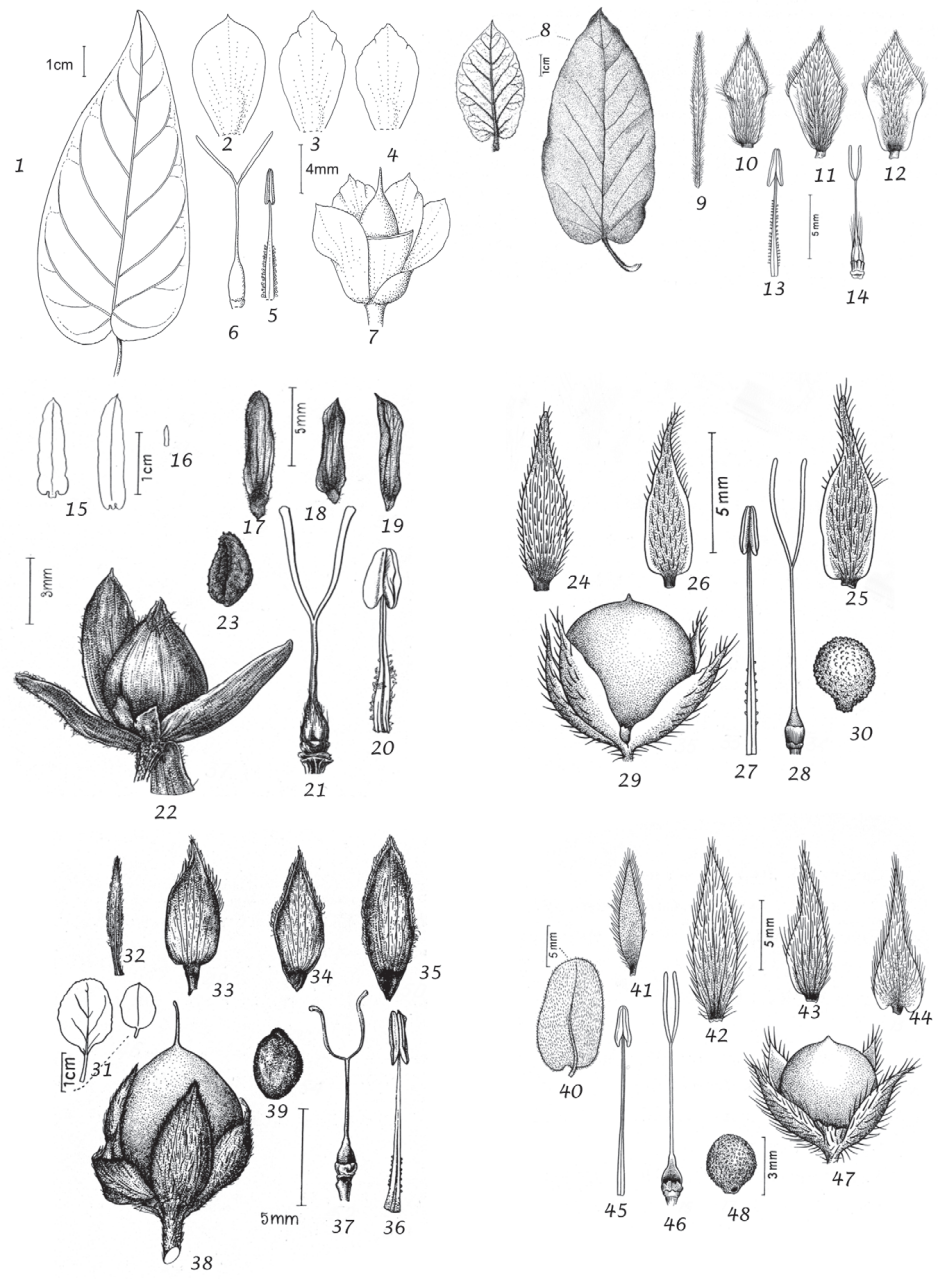
**1–7** *Convolvulus massonii* **1** leaf **2** outer sepal **3** middle sepal **4** inner sepal **5** stamen **6** ovary and style **7** capsule **1–4** & **6–7** from *Mandon* 180 (BM) **5** from *Press & Short* 446 (BM) **8–14** *Convolvulus canariensis* **8** leaves, adaxial (right) and abaxial surfaces (left) **9** bracteole **10** outer sepal **11** middle sepal **12** inner sepal **13** stamen **14** ovary and style. From *Bourgeau* 1428 (C) **15–23** *Convolvulus fruticulosus* **15** leaves **16** bracteole **17** outer sepal **18** middle sepal **19** inner sepal **20** stamen **21** ovary and style **22** capsule **23** seed **15–21** from *Bornműller* 2612 (W) **22–23** from *Perraudière* 1429 (C) **24–30** *Convolvulus valentinus* **24** outer sepal **25** middle sepal **26** inner sepal **27** stamen **28** ovary and style **29** capsule **30** seed **24–28** from *Balansa* 358 (W) **29–30** from *Bourgeau* 80 (GOET) **31–39** *Convolvulus sabatius* **31** leaves **32** bracteole **33** outer sepal **34** middle sepal **35** inner sepal **36** stamen **37** ovary and style **38** capsule **39** seed. From *Jahandiez* 308 (E) **40–48** *Convolvulus supinus* **40** leaf **41** bracteole **42** outer sepal **43** middle sepal **44** inner sepal **45** stamen **46** ovary and style **47** capsule **48** seed **40** from *Raymond* 13k (RAB) **41–46** from *Letourneaux* 2 (C) **47–48** from *Kralik* 68 (C).

##### Distribution.

Endemic to Madeira (*Mandon* 180, *Lowe* 576, *Press & Short* 446), 400–1000 m.

##### Notes.

Very similar to *Convolvulus canariensis* but distinguished by its nearly glabrous leaves.

#### 77. Convolvulus canariensis

Taxon classificationPlantaeSolanalesConvolvulaceae

L., Sp. Pl. 155. 1753. (Linnaeus 1753: 155).

Figure 11, t. 8–14

Convolvulus pallidus Salisb., Prodr. Stirp. Chap. Allerton 123. 1796, illegitimate superfluous name for *Convolvulus canariensis* L. (Salisbury 1796: 124).
Convolvulus pannifolius Salisb., Parad. Lond. 1: t.20 (Salisbury 1805: t.20). Type. Icon, in Parad. Lond. 1: t.20 drawn from a plant from Tenerife (Canary Islands).
Nemostima canariensis (L.) Raf., Fl. Tellur. 4: 82. 1838. (Rafinesque 1838: 82). Type. Based on *Convolvulus canariensis* L.
Periphas pannifolius (Salisb. )Raf. Fl. Tellur. 4: 85. 1838. (Rafinesque 1838: 85). Type. Based on *Convolvulus pannifolius* Salisb.
Convolvulus bourgaei Bolle, Bonplandia 9: 54. 1861. (Bolle 1861: 54). Type. CANARY ISLANDS, Arafo, Ternerife, *Bolle* (whereabouts uncertain).

##### Type.

CANARY ISLANDS (lectotype LINN 218.17!, designated by Sa’ad 1967: 248).

##### Description.

A liana or scrambling shrub to 10 m, old stems woody with brown bark, young stems villous. Leaves petiolate, 4–10 × 2–5 cm, ovate to oblong-ovate, acute, entire, cordate at base, densely villous, the veins prominent on the lower surface; petioles 1–1.5 cm. Flowers 3–7 in axillary, pedunculate cymes; peduncles c. 2.5–3.5 cm, bracteoles linear, acuminate; pedicels 5–18 mm; sepals 8 × 4 mm, elliptic-rhomboid, apiculate, pilose, the inner sepals with glabrous, membranous margins; corolla 1.8–2.2 cm long, pale blue with a white centre, unlobed, midpetaline bands pilose; filaments glandular; ovary sparsely pilose at apex; style glabrous, divided 3–4 mm above base; stigmas c. 4 mm. Capsule glabrous; seeds smooth, glabrous. [Sa’ad 1967: 117 p. p., Bramwell and Bramwell 2001: 264–265 (photo); Schönfelder and Schönfelder 1997: 174–175 (photo)]

##### Distribution.

Endemic to the Canary Islands, but common in and around laurel forest, 400–1000 m. Gran Canaria, Tenerife, La Palma, La Gomera, El Hierro (*Asplund* 927, *Murray* s.n. [19/5/1892], *Bourgeau* 1428).

#### 78. Convolvulus volubilis

Taxon classificationPlantaeSolanalesConvolvulaceae

Brouss. ex Link, Phys.Beschr. Canar. Ins. 145. 1828 [1825]. (Buch 1828: 145).

Rhodorrhiza volubilis (Brouss. ex Link) Bolle, Bonplandia 9: 54. 1861. (Bolle 1861: 54). Type. Based on *Convolvulus volubilis* Brouss. ex Link
Convolvulus diversifolius Mendoza-Heuer, Cuad. Bot. Canaria 12: 27. 1971, nom. illeg., non *Convolvulus diversifolius* Spreng. (1824). (Mendoza-Heuer 1971: 27). Type. CANARY ISLANDS, *Bourgeau* 1427b (holotype P00434110!).

##### Type.

CANARY ISLANDS, Tenerife, *Buch* 204 (holotype B†); Tenerife, Risco de Tagana, 27 Mar. 1855, *Bourgeau* 1427b (neotype P00434110!, designated here).

##### Description.

A liana or scrambling shrub, the stems woody below, vegative parts glabrous to thinly pilose. Leaves petiolate, 5–7 × 0.5–2.2 cm, linear-lanceolate, acute to obtuse, entire, base rounded, glabrescent to thinly pilose, veins prominent beneath; petioles 4–12 mm. Flowers 1–3 in pedunculate axillary cymes; peduncles 1–2.5 cm, slender; bracteoles 1–3 mm, filiform; pedicels 3–7 mm; outer sepals 6 × 3 mm, broadly oblong, slightly constricted below triangular, acute, with a greenish apical portion, inner sepals ovate, slightly smaller; corolla 2 cm, whitish with pink, pilose midpetaline bands, deeply lobed with lanceolate lobes; ovary glabrous; style glabrous, divided c. 3 mm above base, stigmas 6 mm. Capsule not seen.

##### Distribution.

Endemic to the Canary Islands: Tenerife and La Gomera; 300–800 m (*Bourgeau* 1427, *Carine & Santos Guerra* 196c, *Lowe* s.n. [19/4/1861]).

#### 79. Convolvulus lopezsocasii

Taxon classificationPlantaeSolanalesConvolvulaceae

Svent., Addit. Fl. Canar. 1: 46. 1960. (Sventenius 1960: 46).

##### Type.

CANARY ISLANDS, *Lopezsocas* s.n. (probable holotype ORT-22418– but collection date does not correspond exactly).

##### Description.

Similar to *Convolvulus volubilis* but leaves 3–9 × 3–4.2 cm, ovate-elliptic, obtuse, entire, base rounded to subcordate, dark green, glabrous. Flowers 1–6 in axillary pedunculate cymes; peduncles 1.5–3.5 cm, bracteoles 5–12 mm, filiform, somewhat caducous; pedicels 10–18 mm, sepals 9–12 mm, oblong-obovate with a distinct triangular apiculate apex, subglabrous; corolla 1.6–2 cm, pink, unlobed, midpetaline bands darker; ovary c. 3 mm long, conical, thinly pilose; style glabrous, divided c. 4 mm above base. Capsule and seeds not seen. [Bramwell and Bramwell 2001: 264–265 (photo)]

##### Distribution.

Endemic to Lanzarote in the Canary Islands at 400–600 m (*Murray* s.n. [16/5/1902], *Stearn* 1124–cultivated on Gran Canaria).

#### 80. Convolvulus sp. A

Taxon classificationPlantaeSolanalesConvolvulaceae

##### Description.

Twining perennial of unknown size, stems pubescent. Leaves petiolate, 3.5–7 × 1.2–2 cm, narrowly ovate, apiculate, entire, base cordate, weakly auriculate, veins prominent beneath, both surfaces minutely tomentellous. Flowers 2–5 in axillary, pedunculate cymes; peduncles 1.5–2.5 cm; bracteoles 7–10 × 0.5–2 mm, shortly petiolate, oblong, acuminate; pedicels 6–18 mm; outer sepals 10–13 × 3–4.5, narrowly ovate, acuminate, tomentose, inner sepals 8–10 mm long, acute; corolla 2.2–2.4 cm long, lobed, midpetaline bands pilose; ovary c. 4 mm long, densely hirsute; style pilose, divided 3–4 mm above base. Capsule and seeds not seen.

##### Distribution.

Only known from La Palma in the Canary Islands (*Bramwell & Humphries* 3448).

##### Note.

This appears to be a distinct species but we hesitate to describe it given the complexity of the Canary species and the fact that it is only known from a single collection. Its sepals are distinctly longer than those of both *Convolvulus fruticulosus* and *Convolvulus canariensis* and the tomentellous leaves are different from all similar species except *Convolvulus fruticulosus* but in that species the leaves are oblong not ovate. Further collections are needed to elucidate its exact status.

#### 81. Convolvulus fruticulosus

Taxon classificationPlantaeSolanalesConvolvulaceae

Desr., Encycl. [Lamarck et al.]3: 54. 1792. (Desrousseaux. 1792: 541).

##### Type.

CANARY ISLANDS, a plant grown at Paris from seed sent by Collignon from the Canary Islands (holotype P [Herb. Lam.]!).

##### Description.

Woody-based scrambling plant with long trailing stems to 1.5 m. Leaves shortly petiolate, 1–5 × 0.5–1.4(-2.5) cm, oblong, apiculate, entire, base subcordate, weakly auriculate, thinly to densely tomentellous on both surfaces; petioles 2–4 mm. Flowers 1–3 in shortly pedunculate, axillary cymes the flowers and leaves crowded together; peduncles 0–10 mm; bracteoles, 5–6 mm, filiform, caducous; pedicels 5–12 mm; sepals 6–7 (-9) × 2.5–3 mm, pubescent, outer sepals broadly oblong-elliptic, obtuse to subacute, inner sepals oblong, acute to mucronate; corolla 1.5–1.7 cm long, pale blue, weakly lobed, midpetaline bands pilose; filaments glandular, ovary 3.5 mm long, pilose at apex or glabrous; style glabrous except sometimes near the base, divided 3 mm above base; stigmas 3 mm. Capsule globose-conical, apiculate, c. 4–8 mm long, pilose apically or glabrous; seeds tuberculate, glabrous. [Sa’ad 1967: 250, Bramwell and Bramwell 2001: 262–263 (photo); Schönfelder and Schönfelder 1997: 176–177 (photo)]

##### Notes.

*Convolvulus fruticulosus* is a very variable species endemic to the Canary Islands, and populations appear to vary from island to island and from one part of an island to another part. Populations on the smaller islands are poorly known. Variation is mostly in indumentum, shape of the leaf base, development of the inflorescence and sepal shape. These differences do not correlate well with each other and the various names cited in the synonymy below seem to have been applied somewhat arbitrarily to one variant or another. As sepal shape is commonly of significance in taxon delimitation in *Convolvulus*, our infraspecific classification is based primarily on this character as was Sa’ad’s (1967: 251–252) but we disagree on the decisive sepal characters and on the interpretation or assignation of the various names. We recognise two subspecies:

#### 81a. Convolvulus fruticulosus subsp. fruticulosus

Taxon classificationPlantaeSolanalesConvolvulaceae

Figure 11, t. 15–23.

Convolvulus perraudieri Coss., Bull. Soc. Bot. France 3: 58. 1856. (Cosson 1856: 58). Type. CANARY ISLANDS: *Perraudière* s.n. (holotype P00417707; isotypes FI, K, P).
Rhodorrhiza perraudieri (Coss.) Bolle, Bonplandia 9: 54. 1861. (Bolle 1861: 54). Type. Based on *Convolvulus perraudieri* Coss.
Convolvulus venosus Hallier f., Bot. Jahrb. Syst. 18: 109. 1893 [“1894”], nom. illeg., non *Convolvulus venosus* Desr. (1792). (Hallier 1894: 109). Type. Based on *Convolvulus fruticulosus*Bolle (1861: 54), whose typification is unclear being apparently based at least particially on *Convolvulus fruticulosus* Desr.
Rhodorrhiza subauriculata Burchard, Repert. Spec. Nov. Regni Veg. 13: 57. 1913. (Burchard 1913: 57). Type. CANARY ISLANDS, Gomera, *Burchard* s.n. (type location unknown).
Convolvulus subauriculatus (Burchard) Linding., Abh. Auslandsk., Reihe C, Naturwiss. 8: 190.1926. (Lindinger 1926: 190). Type. Based on *Rhodorrhiza subauriculata* Burchard

##### Distinguishing features.

Characterised by the obtuse to subacute outer sepals. Plants are almost always densely tomentellous and leaves usually subcordate.

##### Distribution.

Endemic to the Canary Islands: Tenerife (*Bornmüller* 2612, *Asplund* 4680, *Murray* s.n. [4/5/1899], *Acebes et al.* s.n. [1/5 1976]), La Gomera (*Carine & Santos Guerra* 197, 198).

##### Notes.

Plants referred to *Convolvulus perraudieri* differ from the the type of *Convolvulus fruticulosus* in having somewhat larger leaves, 3–5 × 0.6–1.4 cm, and cymose flowers with a distinct peduncle but, at least in the Santa Cruz area of Tenerife, occur very close to, if not together with smaller leaved plants, in which the peduncle is suppressed.

#### 81b. Convolvulus fruticulosus subsp. glandulosus

Taxon classificationPlantaeSolanalesConvolvulaceae

(Webb) J.R.I.Wood & R.W.Scotland stat. nov.

urn:lsid:ipni.org:names:77147672-1

Rhodorrhiza glandulosa Webb, Edwards’Bot. Reg. 27(Misc.): 70. 1841. (1841: 70, full description in Webb and Berthelot 1844: 32). Type. CANARY ISLANDS, Gran Canaria, portion of sheet 132087 (*Despréaux* 1) consisting of two shoots covered by collector’s label from “Roches del Barranco de las flores G. C. May 1839” to which the label “TYPUS” is affixed (lectotype FI, designated here).
Convolvulus glandulosus (Webb) Hallier f., Bot. Jahrb. Syst. 18: 102. 1894 [pub.1893]. (Hallier 1894: 102). Type. Based on *Rhodorrhiza glandulosa* Webb
Convolvulus fruticosus var. glandulosus (Webb) Sa’ad, Meded. Bot. Mus. Herb. Rijks Univ. Utrecht 281: 251. 1967. (Sa’ad 1967: 251). Type. Based on *Rhodorrhiza glandulosa* Webb

##### Type.

Based on *Rhodorrhiza glandulosa* Webb

##### Distinguishing features.

Best distinguished from subsp. fruticulosus by the narrowly ovate or oblong-ovate, acuminate (and apiculate) sepals. Additionally the leaves are rounded or truncate at the base and both leaves and sepals are commonly glabrous to thinly pubescent. The ovary and style vary from glabrous to hirsute.

##### Distribution.

Endemic to the Canary Islands: Gran Canaria, La Palma, 500–1000 m.

##### Notes.

Indumentum varies from nearly completely glabrous (*Carine & Durães* 163, 165, *Bramwell & Humphries* 3040, *Bramwell* 1177), to thinly pubescent with somewhat crisped hairs (*Murray* s.n. [9/5/1894]) and to densely tomentellous (*Lowe* s.n. [28/5/1875], *Bramwell* 1905).

#### 82. Convolvulus fernandesii

Taxon classificationPlantaeSolanalesConvolvulaceae

P.Silva & Teles, Bol. Soc. Brot., ser. 2, 53: 515, t. 1. 1980. (Silva and Teles 1980: 515).

##### Type.

PORTUGAL, *Pinto da Silva, Teles & Pina* 9337 (holotype LISE 94263!).

##### Description.

A scrambling or twining shrub, old stems woody, glabrescent, young stems pubescent. Leaves petiolate, 1–6 × 2.5–3.5 cm, elliptic or oblong-elliptic, retuse, entire, base rounded to subcordate, puberulent on the nerves beneath but soon glabrescent; petiole 1–2.5 cm. Flowers 3–6 in axillary, pedunculate cymes; peduncles 1–2 cm; bracteoles 5–9 × 1–2.5 mm, oblanceolate; pedicels 5–10 mm; sepals 6–9 × 3–3.5 mm, obovate to elliptic, mucronate, puberulent, inner sepals scarious, puberulent near base only; corolla 1.5–1.7 cm, white, sinuate, midpetaline bands tomentose; filaments glandular near base; ovary glabrous; style glabrous, divided c. 4 mm above base, stigmas c. 2 mm. Capsule and seeds unknown. [Silvestre 2012: 260]

##### Distribution.

Portugal: Cabo Espichel. Rare, narrow endemic of dolomitic rock at 125 m.

##### Notes.

Although seeds are clearly visible on the image of the type specimen no description has been provided.

This species is related to *Convolvulus canariensis* and its allies from the Canary Islands and Madeira. It differs from *Convolvulus canariensis* in its near glabrous leaves and from *Convolvulus massonii* in its shorter sepals, smaller corolla and glabrous ovary. It is similar to *Convolvulus volubilis* but the leaves are oblong-elliptic, not linear lanceolate.

#### 83. Convolvulus valentinus

Taxon classificationPlantaeSolanalesConvolvulaceae

Cav., Icon. 2: 65, t. 180. 1793. (Cavanilles 1793: 65).

Figure 11, t. 24–30

Convolvulus suffruticosus Desf., Fl. Atlant. 1: 175. 1798. (Desfontaines 1798: 175). Type. ALGERIA, *Desfontaines* s.n. (holotype P).
Convolvulus valentinus var. oranensis Pomel, Nouv.Mat. Fl. Atl. 86. 1874. (Pomel 1874: 86). Type. ALGERIA, Oran, Bou-Tlélis, *Pomel* s.n. (holotype AL, probably divided with MPU!; isotype P00434103!).
Convolvulus valentinus var. melillensis Pau, Ann. Sci. Acad. Polytec. Porto 6: 96. (Pau 1911: 96). Type. MOROCCO, Ain Tellout, *Henry* 6-462 (holotype MA!).
Convolvulus valentinus subsp. suffruticosus (Desf.) Maire, Cat. Pl. Maroc 3: 588. 1934. (Jahandiez and Maire 1934: 588). Type. Based on *Convolvulus suffruticosus* Desf.

##### Type.

SPAIN, Valencia, Alicante, *Cavanilles* s.n. (holotype MA 475578!).

##### Description.

Perennial herb from a rhizomatous rootstock with decumbent stems to 40 cm long; vegetative parts appressed hairy to pilose, often with both types of hair on the same plant. Leaves shortly petiolate, 1.5–4 × 0.2–1(-2.3) cm, lanceolate, oblong or oblong-elliptic, often falcate, acute, entire, base truncate; petioles 1–6 mm. Flowers 1–2 in pedunculate axillary cymes; peduncles 1–4 cm; bracteoles 8–20 × 0.5–2 mm, linear or linear-lanceolate, pedicels 0–3 mm, very short; outer sepals 7–9 × 2.5–4 mm, oblong-ovate to ovate, acuminate, inner sepals oblong-elliptic, cuspidate with broad membranous margins; corolla 2–2.5 cm, blue, pale violet, weakly lobed, midpetaline bands darker on the exterior, adpressed-pilose; filaments glandular below; ovary conical, glabrous; style divided 4–6 mm above base, glabrous, stigmas 3.5–5 mm. Capsule glabrous; seeds glabrous, tubercled. [Sa’ad 1967: 206; Silvestre 2012: 262, 263 (plate); Carine and Robba 2010: 12]

##### Distribution.

SE Spain (*St. Lager* s.n. [27/5/1890], *Porta & Rigo* 67, *Ellman & Sandwith* 1162); Mallorca; Morocco (*Carine et al.* 369, *Font Quer* 489, *Calvo* 2381); Algeria (*Balansa* 358, *Bourgeau* 80, *Chevalier* s.n.[17/4/1897]).

##### Notes.

The unusually short pedicels and often falcate leaves serve to make this species distinct.

#### 84. Convolvulus sabatius

Taxon classificationPlantaeSolanalesConvolvulaceae

Viv., Fl. Libyc. Spec. 67. 1824. (Viviani 1824: 67).

##### Type.

ITALY, Liguria, *Viviani* s.n. (holotype GE; isotype G-DC).

##### Description.

Rather variable perennial herb from a woody rootstock, the stems sometimes short and straight and sometimes with trailing, flexuose stems at least 40 cm long; vegetative parts varying from appressed puberulent to villous. Leaves petiolate, 0.5–3 × 0.3–2.2 cm, ovate to suborbicular, rounded to obtuse, entire, base truncate to subcordate and shortly cuneate onto the petiole; petioles 1–5 mm. Flowers 1–3 in shortly pedunculate axillary dichasial cymes; peduncles 0.5–3.5 cm, commonly flexuose and sometimes recurving in fruit; bracteoles 3–13 × 0.5–2.5 mm, linear to oblong-lanceolate; pedicels 3–12 mm; outer sepals 5–7 × 2–2.5 mm, oblong-lanceolate, acute; inner sepals broader (c. 3 mm wide), thinly pubescent except ciliate margins, membranous; corolla 1.6–2 cm long, blue or violet, unlobed, midpetaline bands pilose; filaments glandular below; ovary glabrous; style glabrous, divided c. 4 mm above ovary, stigmas 3 mm. Capsule glabrous; seeds glabrous, tuberculate. [Sa’ad 1967: 196; Carine and Robba 2010: 17; Pignatti 1982: 388]

##### Notes.

Carine and Robba (2010) circumscribed two subspecies as indicated below. There is much variation in leaf size, especially in North African material. The two subspecies are distinguished by sepal characters but are perhaps most easily recognised because of their geographical disjunction.

#### 84a. Convolvulus sabatius subsp. sabatius

Taxon classificationPlantaeSolanalesConvolvulaceae

Convolvulus pseudosiculus Cav. var. multiflorus Choisy, Prodr. [A.P. de Candolle] 9: 407. 1845. (Choisy 1845: 407). Type. Based on *Convolvulus sabatius* Viv.
Convolvulus georgicus Sa’ad, Meded. Bot. Mus. Herb. Rijks Univ. Utrecht 281: 180. 1967. (Sa’ad 1967: 180). Type. “Georgia, Mount Swant”, *Hohenacker* s.n. (holotype W!).

##### Distinguishing features.

Sepals pubescent with appressed hairs, the margins glabrous.

##### Distribution.

Italy: Liguria (*Bicknell* 3346, *Joad* 1882).

##### Notes.

The type of *Convolvulus georgicus* is a small scrap with a single flower and the appearance of *Convolvulus sabatius*. Examination of the sepals confirms not only that they are the same shape as those of subsp. sabatius but also have its distinct indumentum with a band of appressed hairs along the centre leaving the margin glabrous. The collection might have been of a cultivated plant but was more probably mislabelled in the herbarium. *Convolvulus sabatius* should not be included in the list of species occurring in Georgia.

#### 84b. Convolvulus sabatius subsp. mauritanicus

Taxon classificationPlantaeSolanalesConvolvulaceae

(Boiss.) Murb., Acta Univ. Lund., ser. 2, 19: 19. 1923. (Murbeck 1923: 19).

Figure 11, t. 31–39

Convolvulus mauritanicus Boiss., Voy. Bot. Espagne 2: 418. 1841. (Boissier 1841: 418). Type. ALGERIA, Constantine, *Séjourné* s.n. (holotype G, not seen).
Convolvulus sabatius var. atlanticus Ball, J. Linn. Soc. 16: 578. 1878. (Ball 1878: 578). Type. MOROCCO, Ait Mesan, *J. Ball* s.n. (holotype P!).
Convolvulus sabatius var. mauritanicus (Boiss.) Sa’ad, Meded. Bot. Mus. Herb. Rijks Univ. Utrecht 281: 197. 1967. (Sa’ad 1967: 197). Type. Based on *Convolvulus mauritanicus* Boiss.

##### Type.

Based on *Convolvulus mauritanicus* Boiss.

##### Distinguishing features.

Distinguished by the prominent spreading hairs on the calyx and often also on the stem and leaves.

##### Distribution.

Morocco (*Balls* 2934, *Lindberg* 3902, *Gattefossé* 3/8/1935), Algeria (*Cosson* 9/6/1875). Reaches at least 2300 m in Morocco (Jury 17634). It is also widely cultivated and reported to have escaped in Sicily.

##### Notes.

The flexuose peduncles, prominent bracteoles, essentially broadly oblong leaves and violet flowers make *Convolvulus sabatius* a relatively distinct species.

#### 85. Convolvulus supinus

Taxon classificationPlantaeSolanalesConvolvulaceae

Coss. & Kralik, Bull. Soc. Bot. France 4: 400. 1857. (Cosson and Kralik 1857: 400).

Figure 11, t. 40–48

##### Type.

ALGERIA, Oran, *Bourgeau* 60 (lectotype P00417713!, designated by Sa’ad 1967: 202); isolectotypes C, E!, GOET, K!, P!, W!).

##### Description.

Perennial herb from a deep, somewhat woody rootstock, usually branched at base with trailing herbaceous stems to c. 40 cm long, vegetative parts all (thinly to) densely lanate. Leaves shortly petiolate, 0.6–1.6 × 0.3–0.8 cm, oblong to oblong-elliptic or oblong-ovate, obtuse, acute or shortly mucronate, entire, abruptly narrowed to a truncate or cordate base; petioles 0–1 mm. Flowers 1–3 in shortly pedunculate axillary dichasial cymes, the inflorescence as a whole appearing racemose; peduncles 0.5–3 cm; bracteoles 5–9 × 0.5–1 mm, linear; pedicels 0–3(-8) mm, outer sepals 8–11 × 3 mm. oblong-elliptic, acute; inner sepals ovate, c. 4 mm wide, membranous; corolla 1.8–2.8 cm long, yellow or creamy-yellow, unlobed, midpetaline bands pilose; filaments glandular below; ovary glabrous or with a few hairs; style glabrous, divided 6–10 mm above ovary, stigmas 3–4 mm. Capsule glabrous or with a few hairs; seeds glabrous, tuberculate. [Sa’ad 1967: 202; Siddiqi 1977: 15 (Figure 6); Carine and Robba 2010: 14]

##### Notes.

There is much variation in indumentum and two varieties can be recognised:

#### 85a. Convolvulus supinus var. supinus

Taxon classificationPlantaeSolanalesConvolvulaceae

Convolvulus brevipes Pomel, Nouv.Mat. Fl. Atl. 86. 1874. (Pomel 1874: 86 ). Type. ALGERIA, El Abiad Sidi Cheeikh, *Pomel* s.n. (holotype AL; isotype MPU!).
Convolvulus leucotrichus Pomel, Nouv.Mat. Fl. Atl. 87. 1874. (Pomel 1874: 87). Type. ALGERIA, Metilili, *Pomel* s.n. (holotype AL; isotypes MPU004908!, P00417716!).
Convolvulus supinus var. sulphurescens Maire & Wilczek, Bull. Soc. Nat. Hist. Afrique N. 25: 311. 1934. (Maire 1934: 311). Type. MOROCCO. Tazouggest, *Maire & Wilczek* s.n. (holotype MPU003306!; isotype MPU003307!).
Convolvulus supinus var. atrichogynus Maire & Wilczek, Bull. Soc. Nat. Hist. Afrique N. 25: 311. 1934. (Maire 1934: 311). Type. MOROCCO, Beni-Ouziem oasis, *Maire & Wilczek* s.n. (holotype MPU003305!; isotype P00417712).
Convolvulus supinus subsp. brevipes (Pomel) Quézel and Santa, Nouv. Fl. Algérie 757. 1963. (Quézel and Santa 1963: 757). Type. Based on *Convolvulus brevipes* Pomel

##### Distinguishing features.

Leaves thinly to densely lanate.

##### Distribution.

North African Maghreb: Libya (*Sandwith* 2087, *Park* 359), Tunisia (*Kraik* 398, *Staudinger* 1791), Algeria (*Choulette* 364, *Kralik* 68), Morocco (*Jury* 14592, *Sauvage* 11810, *Maire & Wilczek* s.n. [15/4/1933]) and Niger (*Adnan des Iforas*).

#### 85b. Convolvulus supinus var. melliflorus

Taxon classificationPlantaeSolanalesConvolvulaceae

(Pau) Carine & Robba, Phytotaxa 14: 16. 2010. (Carine and Robba 2010: 16).

Convolvulus valentinus var. melliflorus Pau, Ann. Sci. Acad. Polytec. Porto 6: 6. 1911. (Pau 1911: 6). Type. MOROCCO, Zeluan, *Pau* s.n. (holotype MA).
Convolvulus suffruticosus var. sulfureus Batt., Contrib. Fl. Atlan. 61. 1919 (Battandier 1919: 61). Type. *Ducellier* s.n. (holotype MPU!; isotype P00434104).
Convolvulus valentinus var. sulfureus (Batt.) Maire & Wilczek, Bull. Soc. Nat. Hist. Afrique N. 25: 311. 1934, as “Convolvulus valentinus subsp. suffruticosus var. sulfureus” (Maire 1934: 311). Type. Based on Convolvulus suffruticosus var. sulfureus Batt.
Convolvulus valentinus var. transiens Maire & Wilczek, Bull. Soc. Nat. Hist. Afrique N. 25: 311. 1934, as “Convolvulus valentinus subsp. suffruticosus var. transiens” (Maire 1934: 311). Type. MOROCCO, Tazzouget, *Maire & Wilczek* s.n. (?AL, P00434107!, MPU!, RAB013775).
Convolvulus valentinus var. adpressipilis Maire & Wilczek, Bull. Soc. Nat. Hist. Afrique N. 25: 311. 1934, as “Convolvulus valentinus subsp. suffruticosus var. adpressipilis” (Maire 1934: 311). Type. MOROCCO, Oued Zerzef, NE of Erfoud, *Maire & Wilczek* s.n. (AL?, P00434101!, MPU003308!, RAB 013774!).
Convolvulus valentinus var. simulans Maire, Bull. Soc. Nat. Hist. Afrique N. 27: 250. 1936, as “Convolvulus valentinus subsp. suffruticosus var. simulans” (Maire 1936: 250). Type. MOROCCO, Grand Atlas, *Nain* 10 (lectotype MPU003679!, designated by Sa’ad 1967: 208; isolectotypes P00434105!, P00434102).

##### Type.

Based on Convolvulus valentinus var. melliflorus.

##### Distinguishing features.

Leaves glabrous or glabrescent on the upper surface.

##### Distribution.

Principally in northeastern Morocco with scattered populations in Algeria: Morocco (*Jury* 16928, *Carine et al.* 368, *Mordant* 1100), Algeria (*Dubuis* 13437).

##### Note.

Distinctive because of its lanate indumentum, small, very shortly petiolate leaves and usually elongate, raceme-like inflorescence with yellow corollas and short pedicels, the bracteoles immediately below the sepals.

### Species 86–92. Annuals with blue or bluish flowers

A relatively well-defined group of annual species essentially Mediterranean in their distribution except *Convolvulus simulans*, which grows in California. Molecular studies (Williams et al. 2014) confirm this species is in the same clade as its European relatives. Apart from its isolation this species is also remarkable for having the smallest corolla in *Convolvulus*. In all species in this group with pedunculate flowers, the peduncle becomes recurved in fruit.

#### 86. Convolvulus gharbensis

Taxon classificationPlantaeSolanalesConvolvulaceae

Batt. & Pit., in C.J.E.Pitard, Explor. Sci. Maroc, Bot. 74. 1913. (Pitard 1913: 74).

Figure 12, t. 11–20

##### Type.

MOROCCO, *Picard* 1806 (lectotype AL (possibly transferred to MPU006495), designated by Sa’ad 1967: 181; isolectotypes, K!, MPU!, P00417698!, P00417699!, P00417700!).

##### Description.

Annual herb, commonly much branched at the base, reaching c. 35 cm, stems thinly pubescent. Leaves 2–8 × 1–2.5 cm, obovate-spathulate, obtuse to acute, entire, base attenuate or, above, abruptly cuneate, clearly sessile, glabrous or with a few marginal cilia. Flowers several in a sessile terminal head; bracts 1.5–2.3 × 0.8–1.5 cm, ovate, acute, rounded at the base, thinly pilose with long white hairs, green with a palid area near the base; peduncles absent; bracteoles 12–15 × 1–3 mm, oblong, very variable in size, pilose with long white hairs; pedicels absent; outer sepals 9–10 × 2 mm, oblong, acute, densely pilose with white hairs; inner sepals similar but narrowly lanceolate; corolla 1.5–2.5 cm long, blue, unlobed, midpetaline bands pilose, terminating in a small tooth; filaments with sessile glands; ovary glabrous; style glabrous, divided c. 7 mm above base, stigmas 5 mm. Capsule glabrous, much exceeding calyx; seeds tuberculate.

**Figure 12.**
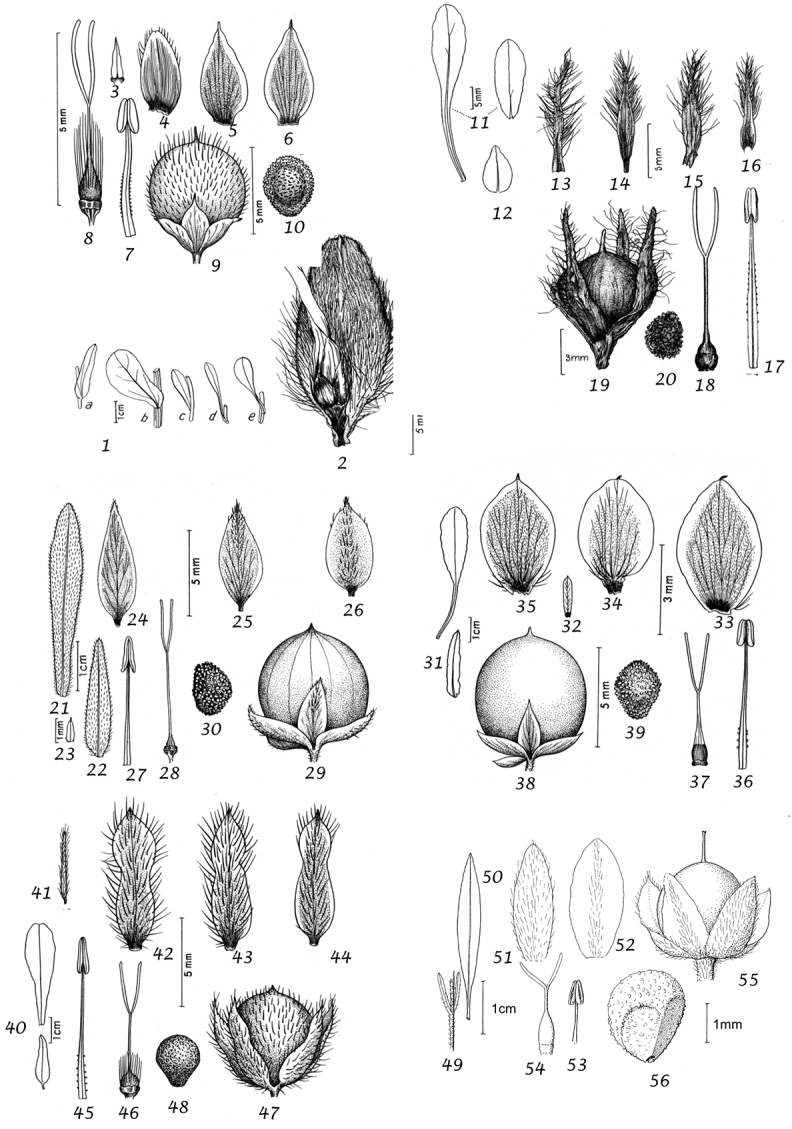
**1–10** *Convolvulus humilis* **1 (a–e)** leaves **2** flower **3** bracteole **4** outer sepal **5** middle sepal **6** inner sepal **7** stamen **8** ovary and style **9** capsule **10** seed **1** from *Bicknell* s.n. (W) **2** from *Faure* s.n. (CAIM) **3–8** from *Choulette* 164 (W) **9–10** from *Huet du Pavillon* s.n. (G) **11–20** *Convolvulus gharbensis* **11** leaves **12** bract **13** bracteole **14** outer sepal **15** middle sepal **16** inner sepal **17** stamen **18** ovary and style **19** capsule **20** seed **11–18** from *Samuelsson* 7188 (B) **19–20** from *Pitard* s.n. (E) **21–30** *Convolvulus meonanthus* **21** leaf **22** bract **23** bracteole **24** outer sepal **25** middle sepal **26** inner sepal **27** stamen **28** ovary and style **29** capsule **30** seed **21–28** from *Ferreira* 1955 (W) **29–30** from *Henriques* s.n. (W) **31–39** *Convolvulus pentapetaloides* **31** leaves **32** bracteole **33** outer sepal **34** middle sepal **35** inner sepal **36** stamen **37** ovary and style **38** capsule **39** seed. **31** from *Silva et al.* 1890 (G) **32–37** from *Davis* 2506 (E) **38–39** from *Huter et al.* 341 (G) **40–48** Convolvulus tricolor subsp. tricolor **40** leaves **41** bracteole **42** outer sepal **43** middle sepal **44** inner sepal **45** stamen **46** ovary and style **47** capsule **48** seed **40–46** from *Ross* 168 (L) **47–48** from *Faure* s.n. (U) **49–56** *Convolvulus simulans* **49** portion of stem with leaves **50** leaf **51** outer sepal **52** inner sepal **53** stamen **54** ovary and style **55** capsule **56** seed **49–54** from *Twisselmann* 10597 (BM) **55–56** from *Boyd & Ross* 6405 (BM).

##### Distribution.

Endemic to Morocco (*Sauvage* 8000, *Davis* 54326, *Mathez et al.* 2426, *Lewalle* 12771).

#### 87. Convolvulus siculus

Taxon classificationPlantaeSolanalesConvolvulaceae

L., Sp. Pl. 1: 156. 1753. (Linnaeus 1753: 156).

##### Type.

ITALY, Sicily (lectotype LINN 218.40!, designated by Verdcourt 1963: 41).

##### Description.

Annual herb, commonly branched at base with prostrate to erect stems to 40 cm long, thinly pilose with brownish hairs on vegetative parts. Leaves petiolate, 1–5 × 0.4–2 cm, ovate-deltoid, acute, base cuneate to abruptly truncate, margin entire; petioles 2–5 (-11) mm. Flowers 1–3 (-4); peduncles 0.6–2.5 cm, bracteoles 2–11 × 1 mm, filiform, linear or linear-lanceolate; pedicels 0–10 mm, becoming recurved in fruit; outer sepals 5–6 × 1.5–3 mm, lanceolate, ovate to subrhomboid, acute; corolla 5–7 mm long, white or lilac, deeply lobed for c. 2 mm, midpetaline bands glabrous; filaments glandular below; ovary glabrous; style glabrous, divided c. 2 mm above base, stigmas 2 mm. Capsule glabrous; seeds glabrous, tuberculate. [Sa’ad 1967: 197; Feinbrun-Dothan 1978 plate 62); Collenette 1999: 232 (photo); Silvestre 2012: 259, 261 (plate); Pignatti 1982: 388; Tohmé and Tohmé 2007: 216; Strid and Strid 2009: 394–395 (plate)]

##### Notes.

We recognise two subspecies, which intergrade occasionally (e.g. *Ascherson* 1054 from the Libyan desert in Egypt, *Finlay* s.n. from Madeira).

#### 87a. Convolvulus siculus subsp. siculus

Taxon classificationPlantaeSolanalesConvolvulaceae

Figure 13, t. 1–8

Convolvulus ovatus Moench, Method. 450. 1794. (Moench 1794: 450). Type. t. 48 (p. 89) in Boccone, Icones & descriptions rariorum plantarum Sicilia.
Convolvulus parviflorus Salisb., Prodr. Stirp. Chap. Allerton 125. 1796, illegitimate superfluous name for *Convolvulus siculus* L. (Salisbury 1796: 125).
Symethus siculus (L.) Raf., Fl. Tellur. 4: 83. 1838. (Rafinesque 1838: 83). Type. Based on *Convolvulus siculus* L.
Convolvulus siculus var. major Choisy, Prodr. (A.P. de Candolle) 9: 407. 1845. (Choisy 1845: 407). Type. Specimen ex Herb. Martius (M).
Convolvulus flexuosus Pomel, Nouv. Mat. Fl. Atl. 84. 1874, nom. illeg., non *Convolvulus flexuosus* Spreng. (1824). (Pomel 1874: 84). Type. ALGERIA, *Garrouban* (holotype AL).
Convolvulus siculus var. flexuosus (Pomel) Batt., Fl. Algérie 2: 546. 1890. (Battandier 1890: 546). Type. Based on *Convolvulus flexuosus* Pomel

##### Distinguishing features.

Bracteoles narrowly lanceolate, but variable in size: 15 × 3.5 mm in the Linnean type but usually much smaller to 4 × 1 mm; pedicels very short (0–1 mm) so bracteoles adjacent to sepals.

**Figure 13.**
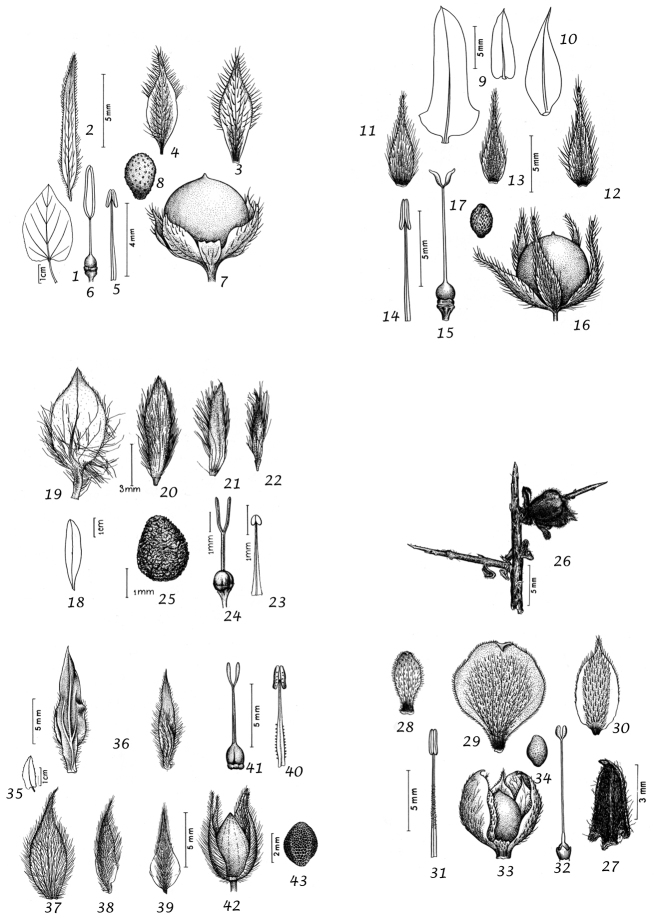
**1–8** Convolvulus siculus subsp. siculus **1** leaf **2** bracteole **3** outer sepal **4** inner sepal **5** stamen **6** ovary and style **7** capsule **8** seed. *From Sharobiem & Shalaby* 942 (CAIM) **9–17** Convolvulus virgatus var. virgatus **9** leaves **10** bracteole **11** outer sepal **12** middle sepal **13** inner sepal **14** stamen **15** ovary and style **16** capsule **17** seed **9–10 & 16–17** from *Rechinger* 3988 (W) **11–15** from *Aucher-Eloy* 4955 (W) **18–25** *Convolvulus rhyniospermus* **18** leaf **19** bracteole **20** outer sepal **21** middle sepal **22** inner sepal **23** stamen **24** ovary and style **25** seed. From *Kotschy* 235 (W) **26–34** Convolvulus hystrix subsp. hystrix **26** habit showing spines and flower position **27** leaf **28** bracteole **29** outer sepal **30** inner sepal **31** stamen **32** ovary and style **33** capsule **34** seed. From *Sa’ad* 1444 (CAIM) **35–43** *Convolvulus glomeratus* **35** leaf **36** bracteole showing variation in shape **37** outer sepal **38** middle sepal **39** inner sepal **40** stamen **41** ovary and style **42** capsule **43** seed. **35** from *Schimper* 784 (W) **36–41** from *Täckholm et al.* 786 (CAI) **42–43** from *Täckholm et al.* 944 (CAI).

##### Distribution.

European Mediterranean, main Mediterranean islands and Western Middle East from Turkey south to Egypt, Jordan and northern Saudi Arabia: Portugal; Spain (*Jerónimo* 8285); Southern France (*Billot* 3435); Balearic Islands (*Bowden & Sims* 6810); Corsica (*Burdon* s.n. [21/5/1913]); Sardinia (*Müller* s.n.); Sicily (*Rigo* 136); Malta (*Kramer & Westra* 4211); Greece (*Stamatiadou* 14643); Aegean Islands (*Davis* 1594); Cyprus (*Merton* 2581); Turkey (*Davis* 41234, 41825); Palestine/Israel (*Davis* 8561, 41234, *Meyer & Dinsmore* 7468); Jordan (*Western* 44); Saudi Arabia (*Collenette* 8527); Egypt (*Simpson* 527); Libya (*Davis* 49978, 50180, *Vaccari* 163); Tunisia (*Fay* 945); Algeria (*Balansa* 359); Morocco (*Faure* s.n. [23/4/1929]); Madeira (*Mandon* 1866); Canary Islands (*Bourgeau* 887, *Sprague & Hutchinson* 391, *Murray* s.n. [16/5/1902]).

#### 87b. Convolvulus siculus subsp. elongatus

Taxon classificationPlantaeSolanalesConvolvulaceae

Batt., Fl Algérie: 595. 1890. (Battandier 1890: 595).

Convolvulus pseudosiculus Cav., Descr. Pl. 97. 1801. (Cavanilles 1801: 97). Type. Cultivated plant (holotype MA 222471!).
Convolvulus elongatus Willd., Enum. Pl. 205. 1809, illegitimate superfluous name for *Convolvulus pseudosiculus* Cav. (Willdenow 1809: 205). Type. Cultivated plant of uncertain origin (lectotype B-W03724010, designated here).
Evolvulus agrestis Schweinf., Beitr. Fl. Aethiop. 92. 1867. (Schweinfurth 1867: 92). Type. ETHIOPIA, *Schimper* 73 (holotype B†; isotype P).
Convolvulus refractus Pomel, Nouv. Mat. Fl. Atl. 1: 84. 1874. (1874: 84). Type. ALGERIA, Mers-el-Kebir, *Pomel* s.n. (holotype AL, not seen).
Convolvulus agrestis (Schweinf.) Hallier f., Bot. Jahrb. Syst. 18: 101. 1893 [“1894”], comb. illeg., non *Convolvulus agrestis* Mart. ex Choisy (1845). (Hallier 1893: 101). Type. Based on *Evolvulus agrestis* Schweinf.
Convolvulus siculus subsp. agrestis (Schweinf.) Verdc., Kew Bull. 12: 344. 1957. (Verdcourt 1957: 344). Type. Based on *Evolvulus agrestis* Schweinf.

##### Type.

Based on *Convolvulus elongatus* Willd.

##### Distinguishing features.

Bracteoles filiform, pedicels 5–10 mm long so bracteoles distant from sepals. [Verdcourt 1963: 41].

##### Distribution.

Principally in NW Africa and the southern Red Sea area: Canary Islands (*Bourgeau* 458, *Aedo et al.* 12447); Spain (*Bourgeau* 1295); Portugal (?); Sardinia (fide Stace 1972); Morocco (*Trethewy* 109, *Jury et al.* 14203, *Jahandiez* 222); Algeria (*Romieux* s.n. [29/4/1883]); Sudan (*Schweinfurth* 2192); Ethiopia (*Schimper* 362, 1294); Eritrea (*Ryding* 1411, *Pappi* 1757); Somalia (*Thulin & Gifri* 8711); Socotra (*Popov* SO/331); Yemen (*Wood* Y/72/177); Saudi Arabia (*Collenette* 488). Rare and scattered elsewhere: Tanzania (*Peter* 43022); Kenya (*Kokwaro* 2840); Oman: Dhofar (*Miller & Nyberg* 9133, *Mandaville* 7359); India (*Beddome* 5618 (BM), from Secunderabad).

##### Notes.

As a species *Convolvulus siculus* is distinctive because of its petiolate, basally truncate leaves and blue flowers.

Some publications (e.g. Dobignard and Chatelain 2011) treat subsp. elongatus and subsp. agrestis as separate taxa but there seems to be no reason for this.

*Convolvulus elongatus* is illegitimate as Willdenow cited the earlier *Convolvulus pseudosiculus* in synonymy. The change of type face in Battandier (1890: 595) indicates *Convolvulus elongatus* is being treated as a subspecific name of *Convolvulus siculus* following an earlier explanation (Battandier 1890: 4).

#### 87a × 91. Convolvulus × beguinotii

Taxon classificationPlantaeSolanalesConvolvulaceae

Maire & Weller, Bull. Soc. Hist. Nat. Afrique N. 30: 293. 1939 (Maire and Weller 1939: 293)

##### Type.

LIBYA, *Maire* 1114 (holotype MPU004048!).

##### Distinguishing features.

This hybrid differs from *Convolvulus siculus* in its oblong-spathulate leaves attenuate at the base and from *Convolvulus humilis* by the upper leaves lanceolate, narrowed, not rounded at the apex.

##### Distribution.

Found once in Libya (Cyrenaica).

##### Notes.

This taxon represents Convolvulus siculus × humilis.

#### 88. Convolvulus pentapetaloides

Taxon classificationPlantaeSolanalesConvolvulaceae

L., Syst. Nat. ed. 12, 3: 229. 1768. (Linnaeus 1768: 229).

Figure 12, t. 31–39

Convolvulus arcuatus C. Presl, Fl. Sic. xxxiii. 1826. (Presl 1826: xxxiii). Type. None specified.
Convolvulus tricolor subsp. pentapetaloides (L.) O. Bolòs & Vigo, Collect. Bot. (Barcelona) 14: 90. 1983. (Bolòs and Vigo 1983: 90). Type. Based on *Convolvulus pentapetaloides* L.

##### Type.

Without locality, *Latourette* s.n. (lectotype LINN 218.41!, designated by Sa’ad 1967: 207).

##### Description.

Annual herb with slender rootstock, often branched at base; stems adpressed pubescent. Lower leaves c. 3–6 × 0.7–1.2 cm; spathulate with an attenuate, pseudopetiolar base, apex obtuse, margin entire, nearly glabrous but more or less ciliate on the margins; upper stem leaves and bracts clasping, 2–4 (-6) × 0.4–0.8 cm, lanceolate (to oblanceolate). Flowers solitary, pedunculate, axillary; peduncles 5–18 mm, pubescent, becoming recurved in fruit; bracteoles 1–3 mm, filiform to narrowly lanceolate; pedicels 3–8 mm, pubescent; sepals somewhat scarious, 5 × 2.5 mm, ovate, acute and mucronate, glabrous apart from long basal trichomes; corolla 0.7–0.9 mm long, blue, shallowly lobed, midpetaline bands pubescent with brown hairs; filaments sparsely glandular below; ovary glabrous or with a few very long trichomes; style glabrous, divided 3–4 mm above the base, stigmas c. 2 mm. Capsule glabrous, strongly exserted from the sepals; seeds covered in pointed tubercles. [Sa’ad 1967: 188; Feinbrun-Dothan 1978 (plate 60); Tohmé and Tohmé 2007: 215 (photo); Pignatti 1982: 388; Silvestre 2012: 269; Strid and Strid 2009: 396–397 (plate)]

##### Distribution.

Circum-mediterranean, east to Iraq: Portugal (*Daveau* 2428); Spain; Balearic Islands (*White* s.n. [4/3/1903]); Italy (*Bicknell & Pollini* 632); Sardinia; Sicily (*Todaro* 923); Malta (*Duthie* s.n. [23/3/1874]); Greece (*Guiol* s.n. [7/1930]); Cyprus (*Meikle* 2006, *Sintenis & Rigo* 60); Turkey (*Siehe* 103); Lebanon (*Gombault* 4504); Syria (*Haradjian* 4344); Palestine/Israel (*Davis* 4486); Jordan (*Trought* s.n. [21/4/1953]); Iraq (*Al-Rawi* 8853); Libya (*Sandwith* 2314, Archibald 968); Russia: Caucasus/Balkaria (*Czermak* s.n.).

##### Notes.

Similar morphologically to *Convolvulus siculus* but distinguished by its sessile leaves.

#### 89. Convolvulus meonanthus

Taxon classificationPlantaeSolanalesConvolvulaceae

Hoffmanns. & Link, Fl. Portug. 1: 369, t.69. 1820. (Hoffmannsegg and Link 1813–20: 369).

Figure 12, t. 21–30

Convolvulus tricolor subsp. meonanthus (Hoffmanns. & Link) Maire, Cat. Pl. Maroc. 3: 589. 1934. (Jahandiez and Maire 1934: 589). Type. Based on *Convolvulus meonanthus* Hoffmanns. & Link

##### Type.

PORTUGAL, Coimbra, *Brotero* s.n. (LISU).

##### Description.

Annual herb, commonly branched at the base, reaching c. 40 cm, stems and leaves with long, stiff spreading hairs. Basal leaves 2.5–4.5 × 0.7–1 cm, obovate-spathulate, obtuse, entire, base attenuate into a pseudopetiole, stem leaves 1.5–5.5 × 0.2–1(-1.8) cm, oblong or lanceolate, sessile. Flowers solitary, axillary, pedunculate; bracts lanceolate, acute; peduncles 1.5–5 cm, slender, flexuose, becoming recurved in fruit; bracteoles 0.5 mm, triangular, acute; pedicels 2–4 mm, not well differentiated from the peduncles; sepals 5–6 × 2–2.5 mm, ovate, acute to apiculate, margin, scarious, ciliate or glabrous, sepals otherwise more or less glabrous; corolla 1.5–2.4 cm long, tricoloured blue, white and yellow, weakly 5-angled with apices of lobes pointed, midpetaline bands adpressed pilose; ovary glabrous; style glabrous, divided c. 5 mm above base, stigmas 2.5–3 mm. Capsule glabrous, much exceeding calyx; seeds tuberculate. [Sa’ad 1967: 185; Pignatti 1982: 388; Silvestre 2012: 268, 271 (plate)]

##### Distribution.

West Mediterranean region, perhaps only in Europe: Algeria (?); Morocco (?); Portugal (*Brummitt & Ernst* 5985); Spain (*Boissier & Reuter* 1841, *Todaro* 922); Italy (*Woolley-Dod* 1756).

##### Notes.

Somewhat resembling *Convolvulus pentapetaloides* but corolla much larger.

#### 90. Convolvulus tricolor

Taxon classificationPlantaeSolanalesConvolvulaceae

L., Sp. Pl. 158. 1753. (Linnaeus 1753: 158).

Figure 12, t. 40–48

##### Type.

Herb. Clifford 68, *Convolvulus* 12, sheet A, (lectotype BM-000558104, designated by Sa’ad 1967: 204).

##### Description.

Annual herb, commonly branched at the base, reaching c. 40 cm, stems and leaves with long, stiff spreading hairs mixed with short, appressed hairs. Lower leaves 2.5–4.5 × 0.7–1.4 cm, obovate-spathulate, obtuse or emarginate, entire, base tapering, stem leaves 1.5–4 (-5) × 0.2–1(-1.8) cm, oblong, obovate or oblanceolate, sessile. Flowers solitary, axillary, pedunculate; bracts lanceolate, acute, resembling upper leaves; peduncles 1–4 cm, slender, flexuose, becoming recurved in fruit; bracteoles 2–3 mm, filiform, acute; pedicels 3–7 mm, not well differentiated from the peduncles; sepals 5.5–7 × 2.5–3 mm, broadly oblong to pandurate, scarious-margined, pilose, clearly differentiated into two parts, the upper green, acute to apiculate, the lower part colourless; corolla 2–3 (-3.5) cm long, tricoloured blue, white and yellow, weakly 5-angled with apices of lobes pointed, midpetaline bands adpressed pilose; filaments glandular below, ovary pilose; style glabrous, divided c. 5–6 mm above base, stigmas 5–6 mm. Capsule pilose, much exceeding calyx; seeds tuberculate. [Sa’ad 1967: 204; Feinbrun-Dothan 1978 (plate 63); Pignatti 1982: 388; Tohmé and Tohmé 2007: 217 (photo); Silvestre 2012: 270, 271 (plate)]

##### Notes.

We recognise two subspecies whose ranges overlap in North Africa:

#### 90a. Convolvulus tricolor subsp. tricolor

Taxon classificationPlantaeSolanalesConvolvulaceae

Convolvulus minor Mill., Gard. Dict. ed. 8: 24. 1768. (Miller 1768: 24). Type. An unspecified cultivated plant.
Convolvulus tricolor var. pseudotricolor Bertol., Fl. Ital. 2; 450. 1835. (Bertoloni 1835: 450). Type. ITALY, Genoa, *Sturla* (GE†).
Convolvulus tricolor var. hortensis Batt., Fl. Algerie 594. 1890. (Battandier 1890: 594).
Convolvulus tricolor subsp. tricolor Type. Plate in Reichenbach (1858: t.137, I, II, 1-10).
Convolvulus maroccanus Batt., Bull. Soc. Bot., France 58: 187. 1911. (Battandier 1911: 187). Type. MOROCCO, Casablanca, *Gentil* s.n. (holotype MPU007512).
Convolvulus tricolor subsp. cupanianus var. guttatus Batt. & Maire, Bull. Soc. Hist. Nat. Afrique N. 19:61. 1928. (Maire 1927: 74). Type. ALGERIA, between Madaurum and Mount Ouenza, Mdaourouch, *Maire* s.n.(syntype MPU010254) and Guelma, *Battandier* s.n. (syntype).
Convolvulus tricolor subsp. hortensis (Batt.) Maire, Bull. Soc. Hist. Nat. Afrique N. 19: 61. 1928. (Maire 1928: 61). Type. Based on Convolvulus tricolor var. hortensis Batt.
Convolvulus tricolor var. quadricolor Batt. & Maire, Bull. Soc. Hist. Nat. Afrique N. 19: 61. 1928. (Maire 1928: 61). Type. TUNISIA, Tunis, *Battandier* s.n. (lectotype MPU001918, designated here).
Convolvulus tricolor var. maroccanus (Batt.) Maire, Cat. Pl. Maroc. 3: 589. 1934. (Jahandiez and Maire 1934: 589). Type. Based on *Convolvulus maroccanus* Batt.

##### Distinguishing features.

The upper, green part of the sepals is acute and shorter than the lower colourless part.

##### Distribution.

Mostly western Mediterranean: Spain (*Boissier* 1837); Portugal (*Atchley* 454); France (*Meebold* 1928); Italy (*Joad* 1882); Greece (*Turner* 43); Morocco (*Davis* 417, *Jury et al.* 19326); Algeria (*Faure* s.n. [3/5/1931]). Cultivated and adventive in Turkey, Crete, Lebanon, the Middle East, Pakistan and doubtless elsewhere.

#### 90b. Convolvulus tricolor subsp. cupanianus

Taxon classificationPlantaeSolanalesConvolvulaceae

(Todaro ex Batt. and Trab.) Cavara & Grande, Bull. Soc. Bot. Ital. 1925: 104. 1925. (Cavara & Grande 1925: 104)

Convolvulus tricolor var. cupanianus Todaro ex Batt. & Trab., Fl. Synop. Alg. Tun. 230. 1905. (Battandier and Trabut 1905: 230). Type. None specified.
Convolvulus tricolor var. heterocalyx Maire, Bull. Soc. Hist. Nat. Afrique N. 28: 369. 1937. (Maire 1937: 369). Type. ALGERIA, between Affreville and Miliana, *Maire* s.n. (holotype AL?; isotype MPU003792!).

##### Type.

ITALY, Sicily, “in campis argillosis–Palermo,” *Todaro* s.n. (lectotype FI!, sheet with label headed ‘Todaro Flora Sicula Exiccata’ annotated “Comp da Todaro in Gen 1864”, designated here).

##### Distinguishing features.

The upper green part of the sepals is acuminate and longer than the lower colourless part.

##### Distribution.

Central Mediteranean: Malta; Sicily; Morocco (*Pitard* 1802); Algeria (*Choulette* 163, *Faure* s.n. [7/6/1929]); Tunisia (*Rico* 1888, *Pitard* s.n. [3/1909]).

##### Notes.

Todaro never provided a description for *Convolvulus cupanianus* and the epithet was first validated at varietal level by Battandier and Trabut (1905) with a brief (and misleading) description in a key. This validation was overlooked by Sa’ad (1967).

#### 91. Convolvulus humilis

Taxon classificationPlantaeSolanalesConvolvulaceae

Jacq., Collectanea 4: 209. 1791. (Jacquin 1791: 209, t. 22).

Figure 12, t. 1–10

Convolvulus undulatus Cav., Icon. 3: 39, t. 277. 1794. (Cavanilles 1794: 39). Type. Plant of unknown origin ex Herb. Cavanilles (lectotype MA 94198!, sheet with original label, designated here).
Convolvulus ciliatus Roth, Catal. Bot. 1: 39. 1797. (Roth 1797: 39). Type. Cultivated plant (holotype BREM).
Convolvulus evolvuloides Desf., Fl. Atlant. 1: 176. 1798. (Desfontaines 1798: 176). Type. NORTH AFRICA, between Algiers and Tripoli (P, not seen).
Convolvulus strictus Lehm., Ind. Sem. Hort. Hamburg 1823: 17. 1823. (Lehmann 1823: 17). Type. Cultivated plant (HBG, not seen).

##### Type.

Plant of unknown origin, cultivated in Vienna by Jacquin (not found at W).

##### Description.

Annual herb, commonly branched at the base with decumbent or ascending stems 5–25 cm long, vegetative parts glabrescent or shortly pubescent. Leaves 0.5–5 × 0.3–1.5 cm, oblong-oblanceolate, apex obtuse to rounded, margin entire, basal leaves gradually narrowed into a long petiole-like base up to 2 cm in length (so appearing spathulate), stem leaves abruptly narrowed at base, sessile and sometimes clasping and auriculate. Flowers solitary, subsessile in the axils of the upper leaves, becoming crowded towards the apex; peduncles and pedicels not clearly differentiated, 0–1 mm long; bracteoles filiform, minute; sepals 2.5–3.5 × 1.5–3 mm, narrowly elliptic to obovate, acute to rounded, thinly pilose; corolla 1–1.1 cm long, blue with a pale tube, distinctly lobed with triangular lobes, midpetaline bands pubescent, darkish; ovary long-pilose, style divided 1–1.5 mm above base, glabrous, stigmas 2 mm. Capsule pilose with stiff coarse hairs; seeds strongly tuberculate. [Sa’ad 1967: 183; Feinbrun-Dothan 1978 (plate 59); Silvestre 2012: 272; Pignatti 1982: 388; Strid and Strid 2009: 398–399 (plate)]

##### Distribution.

Nearly circum-mediterranean but apparently absent from France, Turkey and the Balkans: Spain (*Rivas* s.n. [31/4/1946]); Portugal; Italy (*Todaro* 920); Sicily (Todaro); Morocco (*Jahandiez* 244, *Hooker* s.n. [5/1871]); Algeria (*Balansa* 357, *Cosson* s.n. [22/5/1852]); Libya (*Pampanini & Pichi-Sermolli* 6263); Palestine (*Dinsmore* 3721); Jordan (*Abu Laila et al*. 2005JOR-10-1); Syria: Jebel Druze (*Gombault* 5961); Cyprus (*Syngrassides* 1195).

##### Notes.

Similar morphologically to *Convolvulus pentapetaloides* but flowers sessile or nearly so.

#### 92. Convolvulus simulans

Taxon classificationPlantaeSolanalesConvolvulaceae

L.M.Perry, Rhodora 33: 76. 1931. (Perry 1931: 76).

Figure 12, t. 49–56

Breweria minima A.Gray, Proc. Amer. Acad. Arts 17: 228. 1882. (Gray 1882: 228). Type. MEXICO, Baja California near Tia Juana [Tijuana], *M.E. Jones* 3720 (holotype GH; isotypes K, NY),

##### Type.

Based on *Breweria minima* A.Gray

##### Description.

Annual with adventious root; stems 5–25 cm long from central rootstock, decumbent, indumentum of scattered long hairs mixed with some shorter pubescence. Leaves 1–5 × 0.2–0.6 cm, oblong-oblanceolate; apex rounded; base attenuate, petiole-like, margin entire. Flowers solitary, axilary; peduncles 0.8–1.6 cm, often becoming recurved in fruit; bracteoles 3–6 (-9) × 1–2 mm, oblanceolate; pedicels 1–3 mm; outer sepals 5 × 1 mm, lanceolate-oblong, acute to apiculate, differing from the oblong-obovate inner sepals; corolla 5–6 mm long, deeply lobed, pale blue with white midpetaline bands radiating from the centre; midpetaline bands glabrous, terminating in a mucro; filaments eglandular; ovary glabrous; style glabrous, divided 1–1.5 mm above base, stigmas 1 mm. Capsule glabrous; seeds tuberculate.

##### Distribution.

United States of America: California (*Greene* 1665, *Pringle* s.n. [6/4/1882]), Arizona (fide Austin 2006: 79); Mexico: Baja California (*Raven et al.* 12670).

##### Notes.

Although separated geographically from related species, *Convolvulus simulans* is very close to *Convolvulus pentapetaloides* and its allies morphologically and this is confirmed by our molecular studies which show they all belong to a single clade (Williams et al. 2014).

### Species 93–107. Red Sea group

Vegetatively extremely variable including annual and perennial herbs, spiny and unarmed undershrubs and some species with fastigiate branching. The outstanding feature of many (but not all) species lies in the structure of the stigmas. In many species in this clade (Clade A in Figure 1) the stigmas are widened upwards and oblong-elliptic in shape. In the three species formerly placed in *Seddera* (*Convolvulus kossmatii*, *Convolvulus socotranus*, *Convolvulus semhaensis*), the stigma is not co-extensive with the style arm, its lower part being differentiated from the stigma proper. Another unusual feature lies in the flower colour. Most species in this group are blue-flowered as in the annual species 84–90 and the clade appears to be centred geographically on the Red Sea region.

#### 93. Convolvulus rhyniospermus

Taxon classificationPlantaeSolanalesConvolvulaceae

Hochst. ex Choisy, Prodr. [A.P. de Candolle] 9: 405. (Choisy 1845: 405).

Figure 13, t. 18–25.

Convolvulus hamphilahensis Terracc., Ann. Inst. Bot. Roma 5: 105. 1894. (Terracciano 1894: 105). Type. ERITREA, *Terracciano* 148 (holotype FT).
Convolvulus densiflorus Blatt. & Hallb., J. Bombay Nat. Hist. Soc. 26: 545. 1919, nom. illeg., non *Convolvulus densiflorus* Hook. & Arn. (1827). (Blatter and Hallberg 1919: 545). Type. INDIA, *Blatter* 3515 (lectotype BLATT!, designated by Bhandari 1964: 327).
Convolvulus blatteri Bhandari, Bull. Bot. Surv. India 6: 327. 1964. (Bhandari 1964: 327). Type. Based on *Convolvulus densiflorus* Blatt. & Hallb.
Convolvulus rhyniospermus var. leavis Sa’ad, Meded. Bot. Mus. Herb. Rijks Univ. Utrecht 281: 195.1967. (Sa’ad 1967: 195). Type. SAUDI ARABIA, Jiddah, *Kruijt* 191 (holotype L).

##### Type.

SUDAN, Kordofan [Kurdufan], *Kotschy* 235 (holotype G; isotypes BM001050391!, K!, OXF!, P!).

##### Description.

Annual herb, often branched at base, pubescent in all vegetative parts, stems prostrate to ascending, up to 60 cm long. Leaves shortly petiolate, 0.5–4.5 × 0.1–1.3 cm. ovate, lanceolate, elliptic or oblanceolate, obtuse or acute, margin entire, base cuneate, rarely truncate; petioles 2 mm long. Flowers 3–6 in subsessile, axillary, bracteate heads; peduncles 0–0.5(-3) cm long; bracteoles 4–8 × 2–3 mm, lanceolate or ovate; outer sepals 4–7 × 0.5–1.5 mm, lanceolate, acute, densely villous; corolla 4–5 mm long, white or pale pink, deeply lobed, the lobes longer than broad, 2–2.5 mm long, more or less hidden by calyx hairs, midpetaline bands glabrous, ovary glabrous; style glabrous, divided 2–2.5 mm above base, stigmas c. 1.5 mm long; capsule glabrous, seeds tuberculate or (var. laevis Sa’ad) smooth. [Sa’ad 1967: 194; Collenette 1999: 231 (photo); Thulin 2006: 235; Austin and Ghazanfur 1979: 27, 25 (plate)]

##### Distribution.

A Saharo-Sindian species, unexpectedly absent from southern Arabia and the Gulf region: India (*Aggarwal* s.n. [12/1/1955]); Pakistan (*Stocks* 474, *Jafri* 816); Saudi Arabia (*Collenette* 4734, *Trott* 1338); Egypt (*Khattab* 6346); Sudan (*MacDougal & Sykes* 36); Chad (*Gaston* 568); Ethiopia: Bale/Barrei (*Rippstein* 1236); Eritrea (*Popov* 1396); Djibouti (*Collenette* 8679, *Audru* 7117); Somalia (*Bally* 10218, *Thulin et al.* 9233); Yemen, Hadramaut (*Monod* 16530, 17334); Socotra (*Popov* SO/98).

##### Notes.

The glabrous midpetaline bands of this, the previous and the following species are unusual.

*Convolvulus blatteri* was distinguished by its very small pale pink corolla, precisely the distinguishing character for *Convolvulus rhyniospermus* and images of the type kindly sent by the Blatter herbarium (BLATT) do not suggest any other differences. According to Bhandari his *Convolvulus rhyniospermus* has pedunculate brownish-villous heads with a corolla 16 mm. This fits *Convolvulus glomeratus*, known from Sind adjacent to the Jodhpur region and it seems likely that *Convolvulus blatteri* was described as a consequence of confusion with *Convolvulus glomeratus*, which was treated under the name *Convolvulus auricomus* by Bhandari. *Convolvulus hamphilahensis* was distinguished by the corolla being shallowly lobed with lobes broader than long, 0.5–1 mm in length but this does not justify specific or subspecific rank in the absence of other distinguishing morphological or ecological features.

#### 94. Convolvulus capituliferus

Taxon classificationPlantaeSolanalesConvolvulaceae

Franch., Sert. Somal. 41. 1882. (Franchet 1882: 41).

##### Type.

SOMALIA, *Recoil* 73 (holotype P).

##### Description.

Annual or briefly perennial herb with a small tap root, similar to *Convolvulus rhyniospermus*; stems usually numerous, decumbent or ascending from the base to 40 cm, but usually much less; vegetative parts pubescent. Leaves subsessile, 0.5–6 × 0.2–1.8 cm, oblong or, less commonly, oblanceolate or elliptic, obtuse and sometimes mucronulate, entire, cuneate at the base into an indistinct petiole. Flowers many, in subsessile, bracteate heads forming an elongate leafy inflorescence; peduncles 0–0.4 mm; bracteoles 4–9 × 1–3 mm, linear, oblong-elliptic or lanceolate, acute, ciliate especially in the lower half; sepals 5–7 × 0.5–2.5 mm, ovate, acute, pilose and strongly ciliate, the outer sepals c. 1 mm wider than the inner ones; corolla blue, 7–12 mm long, shallowly lobed, midpetaline bands with a few inconspicuous hairs; ovary glabrous, style glabrous, divided 2.5 mm above base, stigmas linear, 2–3 mm, slightly widened towards apex and sometimes unequal. Capsule glabrous, seeds papillate. [Verdcourt 1963: 40 (as *Convolvulus rhyniospermus*); Verdcourt 1982: 461; Sebsebe 2006 181]

##### Notes.

This species is quite variable, approaching *Convolvulus jefferyi* and *Convolvulus stenocladus* (see discussion under both species) at one extreme and *Convolvulus rhyniospermus* at the other. Specimens without corollas cannot be safely separated from *Convolvulus rhyniospermus*. Following Verdcourt (1982), we recognise two infraspecific taxa, their extremes being very different in appearance:

#### 94a. Convolvulus capituliferus subsp. capituliferus

Taxon classificationPlantaeSolanalesConvolvulaceae

Convolvulus littoralis Vatke, Linnaea 43: 519. 1882. (Vatke 1882: 519). Type. SOMALIA, Las Khoreh, *Hildebrandt* 865b (holotype B†).
Convolvulus capituliferus var. suberectus Franchet, Sert Somal. 41. 1882. (Franchet 1882: 41). Type. SOMALIA, without precise location, *Franchet* s.n. (holotype P00434258!).
Convolvulus sphaerophorus Baker, Bull. Misc. Inform. Kew 1895: 221. 1895. (Baker 1895: 221). Type. SOMALIA, foot of Golis Range, *Edith Cole* s.n. (holotype K).

##### Distinguishing features.

The type variety has small leaves and bracts 0.5–1.5 cm long, equalling or only slightly exceeding the flower heads. The corollas are only 7–8 mm long.

##### Distribution.

Apparently restricted to Somalia and Djibouti, where it is the common variety (*Thulin et al.* 9233, *Glover & Gilliland* 705, *Hemming* 1848, *Collenette* 146). Records from Ethiopia require confirmation.

#### 94b. Convolvulus capituliferus subsp. foliaceus

Taxon classificationPlantaeSolanalesConvolvulaceae

(Verdc.) J.R.I.Wood & R.W.Scotland stat. nov.

urn:lsid:ipni.org:names:77147673-1

Convolvulus capituliferus var. foliaceus Verdc., Kew Bull. 37: 462. 1982. (Verdcourt 1982: 462). Type. KENYA, *Kirrika* 140 (K).

##### Type.

Based on Convolvulus capituliferus var. foliaceus Verdc.

##### Distinguishing features.

Leaves and bracts 3–6 cm long giving the inflorescence a leafy appearance. Corolla 9–12 mm long.

##### Distribution.

This seems to be the only subspecies in Kenya (*Gillett* 21325, *Padwa* 194) and Ethiopia (*Ellis* 232, *de Wilde* 5909) but also occurs in Somalia (*Hemming & Deshmukh Jess* 298).

##### Notes.

Distinguished from *Convolvulus rhyniospermus* by the larger, easily visible, shallowly lobed, blue corolla. Thulin (2006: 235) treated this species as a synonym of *Convolvulus rhyniospermus* but Verdcourt (1982: 459) drew attention to the distinctive characters of the two species.

#### 95. Convolvulus jefferyi

Taxon classificationPlantaeSolanalesConvolvulaceae

Verdc., Kew Bull. 12: 344. 1957. (Verdcourt 1957: 344).

Figure 14, t. 8–12

##### Type.

KENYA, *Jeffery* 749 (holotype EA; isotype K).

##### Description.

Perennial herb with prostrate or twining stems to 50 cm, vegetative parts adpressed pilose, when young with silvery hairs. Leaves shortly petiolate, 1.5–4 × 0.35–1(-2) cm, oblong, ovate or elliptic, apex rounded to acute, base cuneate to subhastate, margin entire; petioles 1–4 mm long. Flowers 2–5 in pedunculate, bracteate heads; peduncles 1–4.5 cm; bracteoles 6–14 × 2–8 mm, lanceolate or oblong; outer sepals 5–9 × 1–4 mm, elliptic to obovate, villous; corolla 8–10 mm long, blue, unlobed, midpetaline bands pubescent; ovary glabrous; style glabrous, divided 5 mm above base, stigmas 3–3.5 mm long, linear. Capsule glabrous; seeds glabrous, tuberculate. [Verdcourt 1963: 40]

**Figure 14.**
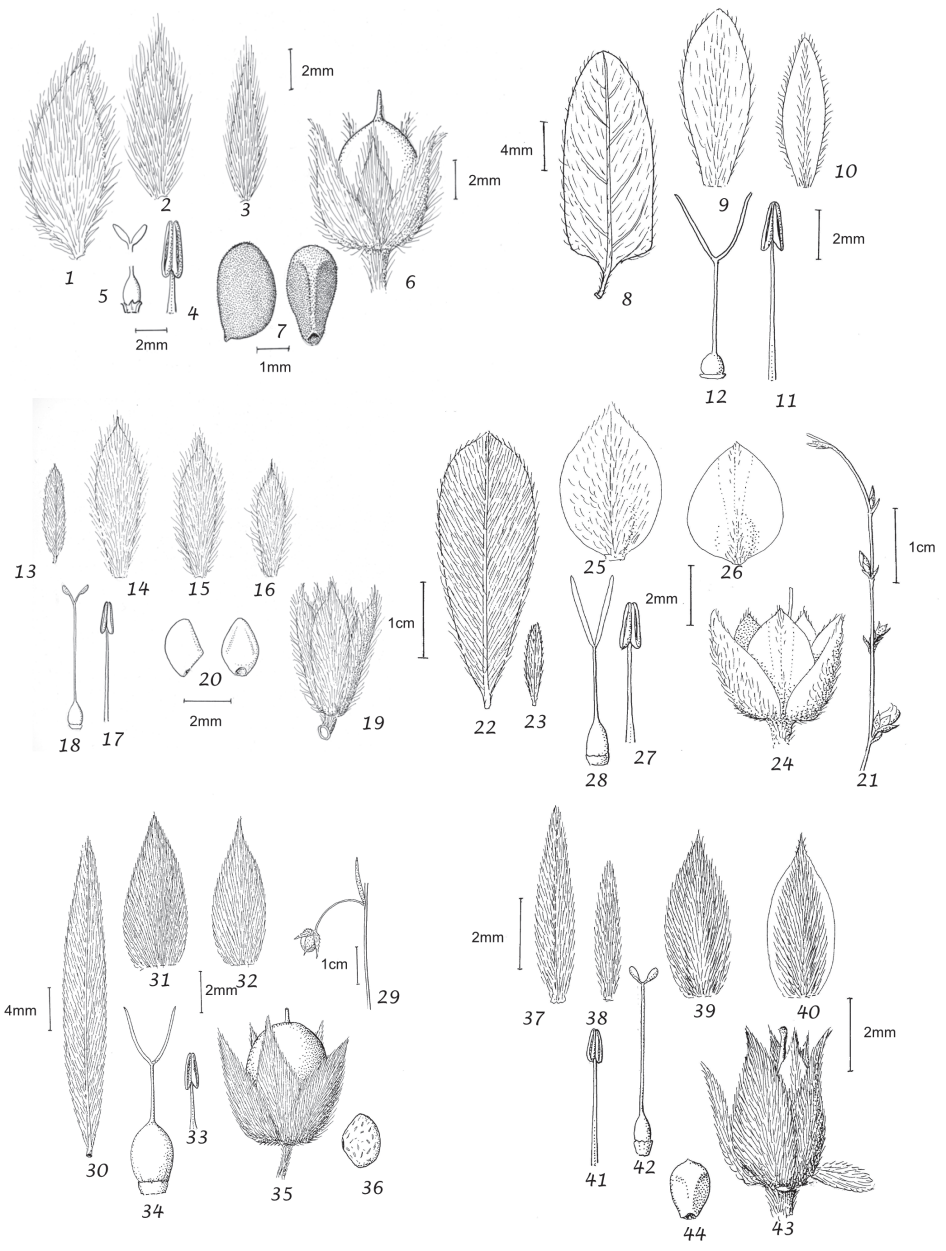
**1–7** *Convolvulus oppositifolius* **1** leaf **2** outer sepal **3** inner sepal **4** stamen **5** ovary and stigma **6** capsule **7** seeds. From *Popov* 68/46 (BM) **8–12** *Convolvulus jefferyi* **8** leaf **9** outer sepal **10** inner sepal **11** stamen **12** ovary and style. From *Greenway* 10454 (K) **13–20** *Convolvulus scopulatus* **13** leaf **14** outer sepal **15** middle sepal **16** inner sepal **17** stamen **18** ovary and style **19** calyx when fruiting **20** seeds. From *Miller at al*. 8142 (E) **21–28** *Convolvulus sericophyllus* **21** habit showing inflorescence **22** basal leaf **23** bract **24** calyx **25** outer sepal **26** inner sepal **27** stamen **29** ovary and style. From *Waring* 57 (BM) **29–36** *Convolvulus stenocladus* **29** habit showing inflorescence and bracteole **30** leaf **31** outer sepal **32** inner sepal **33** stamen **34** ovary and style **35** capsule **36** seed. From *Thulin* 6589 (K) **37–43** *Convolvulus socotranus* **37** leaf **38** bract **39** outer sepal **40** inner sepal **41** stamen **42** ovary and style with bracteole **43** calyx and capsule **44** seed. From *Balfour et al.* 73 (OXF).

##### Distribution.

East Africa, coastal sand, coral outcrops and grassland near the sea: Tanzania (*Tanner* 2211); Kenya (*Irwin* 271, *Greenway* 10454); Somalia (*Terry* 3466); ?Mozambique (Verdcourt 1982: 483).

##### Notes.

This species is not so distinct from *Convolvulus capituliferus* differing principally in its perennial habit and long-pedunculate heads. Three collections (*Tanner* 2338, *Hicks* 808, *Kokwaro* 15114) are intermediate having subsessile inflorescences combined with a clearly perennial habit. The first is from Mlangoni in Tanga Province in Tanzania while the last two are from the Tsavo National Park area (3° 17’S, 38° 32’ E), all in the interior behind coastal *Convolvulus jefferyi* but some distance from any known station for *Convolvulus capituliferus*, which grows in northern Kenya.

#### 96. Convolvulus stenocladus

Taxon classificationPlantaeSolanalesConvolvulaceae

Chiov., Fl. Somala 1: 229. 1929. (Chiovenda 1929: 229).

Figure 14:, t. 29–36

##### Type.

SOMALIA, *Puccioni & Stefanini* 414 & 584 (syntypes FT).

##### Description.

Perennial herb with prostrate stems to 50 cm, vegetative parts appressed pilose. Leaves shortly petiolate, 0.7–3 × 0.1–0.3 cm, linear to narrowly oblong, acute, base cuneate, margin entire; petioles 0.5–1.5 mm long. Flowers 1 (– 2) in pedunculate, axillary, bracteolate heads; peduncles 12–30 mm long, often flexuose or reflexed; bracteoles 4–7 × 1.25–1.5 mm, linear to lanceolate, appressed to sepals; pedicels absent; outer sepals 5–8 × 2–3 mm, lanceolate to ovate; corolla 12 mm long, blue, unlobed, midpetaline bands pubescent; ovary glabrous; style glabrous, divided c. 2 mm above base, stigmas 2.5 mm, linear. Capsule glabrous; seeds glabrous, sinuate-ridged. [Thulin 2006: 237]

##### Distribution.

Somalia (*Thulin & Dahir* 6589; *Gillett et al.* 22497; *Drake-Brockman* 972).

##### Notes.

Easily recognised by its flexuose peduncles, one-flowered heads and bracteoles appressed to the sepals.

Two specimens (*Lavranos & Carter* 23310, *Gillett & Becket* 206) from north of Mogadishu in Somalia have the appearance of hybrids or intermediates between *Convolvulus stenocladus* and *Convolvulus capituliferus*. The heads are few-flowered (mostly 2–3-flowered), sessile towards the apex but pedunculate below.

#### 97. Convolvulus bidrensis

Taxon classificationPlantaeSolanalesConvolvulaceae

Sebsebe, Kew Bull. 54: 67. 1999. (Sebsebe 1999: 67).

##### Type.

ETHIOPIA, *Gilbert & Sebsebe D.* 8640 (holotype ETH; isotype K).

##### Description.

Perennial herb with numerous prostrate stems to 50 cm from a central woody rootstock, vegetative parts finely adpressed pubescent. Leaves shortly petiolate, 1–3.7 × 0.1–0.5 cm, linear to narrowly oblong, apex apiculate, margin entire, base narrowly cuneate; petioles 1–2 mm. Flowers 1–3, subsessile in shortly pedunculate, axillary, bracteate heads; peduncles 2–5 mm long; bracteoles 7–11 × 1–2 mm, narrowly oblong, glabrous; pedicels 0–1.5 mm; outer sepals 8–10 × 1.5–2 mm, narrowly elliptic, acute; corolla 8–10 mm long, blue with a whitish centre, unlobed, weakly crenate, midpetaline bands thinly pilose; ovary and style glabrous, style divided 4–5 mm above base, stigmas 2 mm, linear. Capsule glabrous; seeds glabrous, tuberculate. [Sebsebe 2006: 181 (plate)]

##### Distribution.

Endemic to Ethiopia (*Sebsebe* 2486); only known from Bidre in Bale floristic region.

##### Notes.

Perhaps most similar to *Convolvulus jefferyi* but the base distinctly woody, the leaves linear-oblong and the flowers few, in numerous shortly pedunculate heads.

#### 98. Convolvulus vollesenii

Taxon classificationPlantaeSolanalesConvolvulaceae

Sebsebe, Kew Bull. 54: 69. 1999. (Sebsebe 1999: 69).

##### Type.

ETHIOPIA, *Sidamo et al.* 8236 (holotype ETH; isotype K!).

##### Description.

Perennial herb with prostrate stems to 40 cm, vegetative parts covered in adpressed silvery hairs. Leaves shortly petiolate, 1–2.5 × 0.3–0.6 cm, linear-oblong, acute, margin entire, base more or less truncate; petioles 1–1.5 mm. Flowers 1–2, subsessile in shortly pedunculate, axillary bracteolate heads; peduncles 0–2.5(-5) mm, bracteoles 6–9 × 1.5–2 mm, lanceolate; outer sepals 8–9 × 2.5–4 mm, ovate, shortly acuminate; corolla 9–12 mm, blue, very shallowly lobed, midpetaline bands pilose; ovary glabrous, style glabrous, divided c. 3 mm above base; stigmas c. 2 mm, linear. Capsule glabrous; seeds glabrous, tuberculate. [Sebsebe 2006: 182 (plate)]

##### Distribution.

Ethiopia (*Bidgood et al.* 4980, *Gilbert & Sebsebe* 8661).

##### Notes.

Similar to *Convolvulus bidrensis* but differing in the indumentum, cuneate leaf bases and peduncles 2–5 mm long.

#### 99. Convolvulus subspathulatus

Taxon classificationPlantaeSolanalesConvolvulaceae

Vatke, Linnaea 43: 519. 1882. (Vatke 1882: 520).

##### Type.

SOMALIA, *Hildebrandt* 1312 (holotype B†).

##### Description.

Perennial herb with prostrate or twining stems to 50 cm long, vegetative parts densely sericeous becoming golden-brown when old. Leaves shortly petiolate, 0.6–2 × 0.6–2 cm, suborbicular, apex rounded or, rarely, emarginate or obtuse, base cordate, margin entire; petioles 1–6 mm long. Flowers 2–5 in shortly pedunculate, bracteate heads; peduncles 0.4–1.5 cm; bracteoles 7–10 × 5–7 mm, oblong-elliptic to ovate; outer sepals 5–7 × 2–3 mm, oblong to obovate, villous; corolla 9–11 mm long, blue, unlobed, midpetaline bands pilose; ovary glabrous; style glabrous, divided 3 mm above base; stigmas 1.75–2 mm, narrowly cylindrical and thicker than style. Capsule glabrous; seeds glabrous, tuberculate. [Thulin 2006: 235]

##### Distribution.

Endemic to Somalia (*Thulin & Warfa* 4533, 5907; *Friis et al*. 5024; *Revoil* s.n. [6/1883], *Tardelli & Bavazzano* 502). On coastal sand dunes.

##### Notes.

Thulin (2006: 235) suggests that this species is scarcely different from *Convolvulus jefferyi* and his description of *Convolvulus subspathulatus* in *Flora of Somalia* seems to anticipate uniting the two species. However the suborbicular leaf shape and sericeous indumentum serve to distinguish the two species easily.

#### 100. Convolvulus virgatus

Taxon classificationPlantaeSolanalesConvolvulaceae

Boiss., Diagn. Pl. Orient. 7:24. 1846. (Boissier 1846: 24).

Figure 13, t. 9–17

##### Type.

IRAN, *Aucher-Eloy* 4955 (holotype G; isotypes K!, P!, W!).

##### Description.

Undershrub forming a small bush up to 40 cm high and 60 cm wide; stems from a deep woody taproot, many, ascending, rigid, green, glabrous, sometimes spinescent at the tips, weakly divaricate. Leaves sessile, 1.7–2.5 × 0.2–0.4 cm, lanceolate or linear-lanceolate, glabrous, acute or acuminate, margin entire, base truncate to obscurely auriculate. Inflorescence of few-flowered, axillary, pedunculate, hirsute heads; peduncles 1.5–4.5(-8) cm, rigid, woody; bracteoles 5–12 × 1–2 mm, very variable in size, linear or lanceolate, acute; pedicels 1–2 mm, often bent at a sharp angle to the peduncle; sepals 8–9 × 2–2.5 mm, ovate, acuminate, villous; corolla 1.3–2.1 cm, usually white, sometimes pinkish, shallowly lobed, the midpetaline bands ending in teeth, pubescent, sometimes darker pink; ovary glabrous; style glabrous, divided c. 5.5 mm above the base, stigmas 1–1.25 mm, elliptic. Capsule glabrous; 1–2-seeded; seeds tuberculate, glabrous. [Sa’ad 1967: 86; Austin and Ghazanfar 1979: 16; Jongbloed 2003: 316 (photo); Pickering and Patzelt 2008: 169 (photo)]

##### Notes.

We recognise two varieties:

#### 100a. Convolvulus virgatus var. virgatus

Taxon classificationPlantaeSolanalesConvolvulaceae

##### Distinguishing features.

Plant lax in habit, the branches neither very numerous nor markedly spinescent; leaves all or mostly > 2 cm long; corolla > 1.5 cm long.

##### Distribution.

Locally frequent in desert in southern Gulf region: U.A.E. (*Ghazanfar* 4318, *Western* 23; *York* 80; *Müller-Hohenstein* 86077); Oman (*Radcliffe-Smith* 3615); Iran (*Grey-Wilson & Hewer* 261; *Léonard* 5876, *Rechinger* 3460); Pakistan (*Pierce* s.n., *Lamond* 219).

#### 100b. Convolvulus virgatus var. subaphyllus

Taxon classificationPlantaeSolanalesConvolvulaceae

Boiss., Fl. Orient. [Boissier] 4: 89. 1875. (Boissier 1875b: 89).

Convolvulus mascatensis Boiss. Diagn. Pl. Orient. 7:25. 1846. (Boissier 1846: 25). Type. OMAN, Muscat, *Aucher-Eloy* 4938 (holotype G; isotypes BM 000049109, P, W).

##### Type.

Based on *Convolvulus mascatensis* Boiss.

##### Distinguishing features.

Differs from the type in being very compact with short, intricately branched. spinescent shoots, relatively short, broad leaves, small heads and short corollas. Possibly an adaptation to extreme arid conditions.

##### Distribution.

Oman (*Gallagher* 7758, 7965, *McLeish 1848, Whitcombe* 340 and *Miller* 6539).

##### Notes.

*Convolvulus virgatus* is sometimes confused with *Convolvulus glomeratus* but the branches are rigid and woody and the leaves linear-oblong, glabrous.

#### 101. Convolvulus glomeratus

Taxon classificationPlantaeSolanalesConvolvulaceae

Hochst. ex Choisy, Prodr. [A.P. de Candolle] 9: 401. 1845. (Choisy 1845: 401).

Figure 13, t. 35–43

##### Type.

SAUDI ARABIA, Jiddah, *Schimper* 784 (lectotype G-DC, designated by Sa’ad 1967: 182); isolectotypes GOET, OXF!, P!, W!).

##### Description.

Perennial herb with prostrate, ascending or twining stems to 1 m, rootstock thick and somewhat woody, pubescent on vegetative parts; stems sometimes slightly woody. Leaves shortly petiolate, 1–4.5 × 0.5–1.5 cm, lanceolate or ovate, acute, base truncate to cordate, margin entire; petioles 0–5 mm long. Flowers 4–10 in axillary, pedunculate, villous, bracteate heads formed of compact scorpioid cymes; peduncles 1–4 (-7) cm long, straight or recuvedbracteoles 8–12 (-28) × 2.5–5 (-9) mm, ovate, acuminate, villous; outer sepals 8–12 × 3.5–4.5 mm, ovate, long-acuminate, broader than the inner sepals; corolla 8–12 mm long, white to pale blue, undulate, midpetaline bands pilose; ovary glabrous; style glabrous, divided c. 4 mm above base, stigmas ellipsoid, 1.5–2 mm. Capsule glabrous, seeds glabrous, tuberculate. [Feinbrun-Dothan 1978: plate 58; Nowroozi 2002: 79 (plate), 105 (map); Collenette 1999: 229 (photo); Austin and Ghazanfur 1979: 24, 25 (plate); Jongbloed 2003: 312 (plate)]

##### Notes.

We recognise two varieties:

#### 101a. Convolvulus glomeratus var. glomeratus

Taxon classificationPlantaeSolanalesConvolvulaceae

Ipomoea auricoma A.Rich., Tent. Fl. Abyss. 2: 65. 1850. (Richard 1850: 65). Type. ETHIOPIA, *Quartin-Dillon* s.n. (holotype P!; isotype BM000930475!).
Convolvulus glomeratus var. volubilis C.B.Clarke, Fl. Brit India (J.D. Hooker) 4: 249. 1884. (Clarke 1884: 249) Type. PAKISTAN, Sind, *Dalzell* 53. (lectotype K!, designated here).
Convolvulus faurotii Franch., J. Bot. (Morot) 1: 121. 1887 (Franchet 1887: 121) Type. DJIBOUTI, Tadjourah, *Faurot* (holotype P, not seen).
Convolvulus arabicus Hochst. ex Hallier f., Bot. Jahrb. Syst. 18: 100, 1894 [pub.1893]. illegitimate superfluous name, *Convolvulus glomeratus* Choisy cited in synonymy (Hallier 1894: 100). Type. Various syntypes (from an annotation on *Schimper* 784).
Convolvulus glomeratus var. sericeus Dinsm., Fl. Syria (G.E. Post), ed. 2, 2: 206. 1933. (Post 1933: 206). Type. PALESTINE/ISRAEL, Engedi, *Dinsmore* and Usdum to Engedi, *Post herbarium* (syntypes BEI).
Convolvulus zargarianus Parsa, Kew Bull. 2: 214. 1948. (Parsa 1948: 214). Type. IRAN, Bandar Abbas, *Parsa* 568 (holotype K!).
Convolvulus auricomus (A.Rich.) Bhandari, Bull. Bot. Surv. India 6: 327. 1964. (Bhandari 1964: 327). Type. Based on *Ipomoea auricoma* A.Rich.
Convolvulus auricomus var. volubilis (C.B.Clarke) Bhandari, Bull. Bot. Surv. India 6: 327. 1964. (Bhandari 1964: 327). Type. Based on Convolvulus glomeratus var. volubilis C.B.Clarke
Convolvulus glomeratus var. gymnospermus Sa’ad, Meded. Bot. Mus. Herb. Rijks Univ. Utrecht 281: 183.1967. (Sa’ad 1967: 183). Type. EGYPT, Wadi Ise, *Sa’ad* 1398 (holotype CAIM, not seen).
Convolvulus auricomus var. ferrugineus Fl. Indian Desert 245. 1978. (Bhandari 1978: 245). Type. INDIA, Rajasthan, Jodhpur, *Bhandari* 363 (holotype JAC; isotype CAL).

##### Distinguishing features.

Corolla 8–12 mm long; sepals usually < 10 m long; pedunces usually straight.

##### Distribution.

A characteristic Saharo-Sindian species: Niger (*Newby* 62); Nigeria (*Sharland* 1679); Egypt (*Khattab* 6478, *Danin* s.n. [14/12/1968]); Sudan (*Schweinfurth* 2167); Eritrea: Doomairah Island (*Courbon* 385); Ethiopia (*Friis et al.* 10733); Djibouti (*Aubert de la Rue* s.n. [3/1938]); Somalia (*Newbould* 995); Socotra (*Smith & Lavranos* 724, *Schweinfurth* 387); Kenya (*Luke* 5447); Saudi Arabia (*Zohrab* 268); Yemen (*Miller & Long* 3316, *Deflers* 715, *Smith & Lavranos* 58); Oman (*Radcliffe-Smith* 5161); U.A.E (*Heller* 293); Palestine/Israel (*Meyers & Dinsmore* 1189, *Davis* 3806); Jordan (*Abu Laila* 38-2); Iran; Pakistan (*Stocks* 376, *Lamond* 792, *Rechinger* 28606); India (*Santapau* 16602, *Shetty* 2317).

#### 102b. Convolvulus glomeratus var. sachalitarum

Taxon classificationPlantaeSolanalesConvolvulaceae

R.R.Mill ex J.R.I.Wood & R.W.Scotland var. nov.

urn:lsid:ipni.org:names:77147666-1

##### Diagnosis.

A subsp. typo corolla 15–20 mm longa (non < 12 mm longa) et usque 2.5 cm diametro dignoscenda.

##### Type.

OMAN, Dhofar, Cliff near Dalkut, *McLeish* 2813 (holotype E00132890!).

##### Distinguishing features.

The key feature of this variety is the very large corolla 1.5–2 cm long when dry and up to 2.5 cm in diameter when living. The leaves are always relatively large, up to 4 × 2.5 cm, the peduncles long and commonly gently curved, reaching 7 cm in length, and the sepals may reach 12 mm. However, all characters apart from the corolla size can be matched in other populations of *Convolvulus glomeratus* and as the corolla size is hardly constant, varietal status seems appropriate.

##### Distribution.

Oman (Dhofar): *McLeish* 697, 2248, 2415, 2661, 2814; *Ash* 126; *Collenette* 8347; *Miller* 2262, 2331, 2356; *Miller & Whitcombe* 2082.

##### Notes.

There has been some uncertainty about the correct name for this species. *Convolvulus glomeratus* Thunb. is a *nomen nudum* so the combination *Convolvulus auricomus* is unnecessary. *Convolvulus arabicus* Hochst. & Steud. also appears to be a *nomen nudum*, so *Convolvulus glomeratus* remains the accepted name. *Convolvulus zargarianus* is a synonym of *Convolvulus glomeratus*. Although Sa’ad describes the ovary as velutinous and the style as hairy and shows this in her illustration, this is an error.

#### 102. Convolvulus oppositifolius

Taxon classificationPlantaeSolanalesConvolvulaceae

Alfarhan, Bot. J. Linn. Soc. 106: 259. 1991. (Alfarhan 1991: 259).

Figure 14, t. 1–7

##### Type.

OMAN, *Gallagher* 6763/2 (holotype E00285430!).

##### Description.

Low undershrub from a woody base reaching c. 30 cm; stems densely tomentellous with white, spreading hairs, becoming stiff and rather woody when old and said to form a candelabra-shaped plant. Leaves alternate below but mostly opposite upwards, subsessile, 5–13 × 2–5 mm, elliptic or oblong-elliptic, apex acute to rounded, margin entire, base cuneate to truncate, glandular and hirsute with short spreading hairs on both surfaces; petioles 0–1 mm. Flowers 1–2 in small sessile, axillary clusters near the branch tips; peduncles and pedicels absent or nearly so; bracteoles 6 × 2 mm, oblong, acute; outer sepals 7–8 × 1.8–2 mm, lanceolate, acute, green near apex but membranous below, sericeous; inner sepals narrower (1.5–1.8 mm wide) with membranous margin; corolla 11–13 mm long, pinkish or “pale blue”, midpetaline bands villous; ovary glabrous; style glabrous, divided c. 6 mm above base, stigmas short, clavate. Capsule glabrous, seeds minutely rugulose.

##### Distribution.

Endemic to Oman (*Miller* 6492; *Miller & Nyberg* 9462, *McLeish* 3263; *Hughes & Gallagher* 7895/15, *Popov* 68/35, 68/46).

##### Notes.

Very distinct because of the short clavate stigmas and small, tomentose leaves arranged oppositely towards the branch tips. Some plants have very reduced leaves giving the plant a somewhat different facies (e.g., *Popov* 68/23, BM) and have been confused with *Convolvulus hystrix*.

#### 103. Convolvulus scopulatus

Taxon classificationPlantaeSolanalesConvolvulaceae

Thulin, Nordic J. Bot. 23: 629. 2005. (Thulin 2005: 629).

Figure 14: 13–20

##### Type.

SOMALIA, *Glover & Gilliland* 686 (holotype K!; isotypes BM!, FHO!).

##### Description.

Slender virgate unarmed shrub of fastigiate habit to 1 m high; young stems glabrous to thinly adpressed pubescent, bluish-grey, rigid and woody when mature. Leaves sessile, usually erect, 1.5–4 × 0.5 mm, linear to narrowly elliptic, acute, glabrous or minutely pubescent. Flowers 1–5 (- 8) in subsessile, bracteate, silky villous heads; peduncles 0.5–3 mm long, bracteoles 3–7 × 1–2 mm, linear-lanceolate, acute, villous; outer sepals 7–9 × 1–1.5 mm, lanceolate to oblong -oblanceolate, acuminate, villous, inner sepals 5–7 mm; corolla 10–12 mm long, pale blue, unlobed, midpetaline bands pilose; ovary glabrous; stigma narrowly elliptic. Capsule glabrous; seeds 1, glabrous, smooth. [Thulin 2006: 235]

##### Distribution.

Northern Somalia (*Gillett & Watson* 23869); Yemen: Hadramaut (*Rauh & Lavranos* 13262, *Thulin et al*. 8067, 8207, *Miller et al.* 8142). Coastal desert.

##### Notes.

The type collection virtually lacks leaves. The Somalia plants and *Miller at al*. 8142 from Yemen have glabrous stems whereas the stems are thinly adpressed-pubescent in the other Yemen collections.

#### 104. Convolvulus hystrix

Taxon classificationPlantaeSolanalesConvolvulaceae

Vahl, Symb. Bot. 1: 16. 1790. (Vahl 1790: 16).

##### Type.

Based on *Convolvulus spinosus* Forssk., non *Convolvulus spinosus* Burm.f.

##### Description.

Subshrub, much branched with entangled spinescent branchlets to 1 (- 2) m, vegetative parts glabrous, sericeous, pubescent or densely pilose. Leaves subsessile, 4–10 (-15) × 1–3 (-5) mm, narrowly oblong, elliptic or ovate, acute, base broadly cuneate, truncate to auriculate, margin entire. Flowers 1–6 in subsessile, elliptic to suborbicular axillary clusters; peduncles absent; bracteoles 3–7 × 2–5 mm, narrowly elliptic to obovate; pedicels 0–1 mm; outer sepals 7–9 × 6–8 mm, broadly ovate or elliptic, acuminate, wider than inner ones; corolla 0.8–1.4 cm long, pale to dark violet, weakly lobed, midpetaline bands pubescent, brown; ovary glabrous; style glabrous, divided 6–7 mm above base, arms unequal c. 1–1.5 mm, stigmas short and narrowly elliptic, c. 1 mm. Capsule glabrous; seeds glabrous.

##### Notes.

Within Arabia and along the Red Sea this species is reasonably constant although there is variation in whether hairs are appressed or spreading and in the number of flowers in each head, but much of this variation seems random geographically. In Somalia variation is much greater and three subspecies are here recognised:

#### 104a. Convolvulus hystrix subsp. hystrix

Taxon classificationPlantaeSolanalesConvolvulaceae

Figure 13, t. 26–34

Convolvulus spinosus Forssk., Fl. Aegypt.-Arab. cvi. 1775, nom. illeg., non *Convolvulus spinosus* Burm.f. (1768). (Forsskål 1775: cvi). Type. YEMEN, Bait al Faqih, *Forsskål* (holotype C, isotype BM000049217).
Convolvulus armatus Delile, Descr. Egypte, Hist. Nat. 189. 1813. (Delile 1813: 189). Type. EGYPT, Red Sea, *Delile* s.n. (holotype MPU; isotype P!).
Convolvulus hystrix subsp. dhofaricus R.R.Mill, Edinburgh J. Bot. 70: 373. 2013. (Mill 2013: 373). Type. OMAN, Dhofar, *Miller & Nyburg* 9288 (holotype E!; isotype K!).

##### Distinguishing features.

Plant spiny with numerous short spine-like lateral branchlets arising on the main spinescent branches.Stems and leaves pubescent to pilose. Flowers in clusters of up to 6 forming a suborbicular head; bracteoles obovate, about as broad as long, corolla 1.2–1.4 cm long. Sa’ad 1967: 75; Collenette 1999: 229 (photo); Boulos 2000: 331.

##### Distribution.

Red Sea coastal regions of Egypt (*Boulos & Tackholm* s.n. [12/3/1965]), Sinai (*Danin* s.n. [27/3/1971]), Sudan (*Schweinfurth* 2151), Eritrea (*Bally* 6913), Djibouti, Somalia (*Gillett* 4735, *Collenette* 38), Saudi Arabia (*Kercher* 44, *Zohrab* 5) and Yemen (*Wood* Y/75/2); also in the Hadramaut region of Yemen (*Woodford* 31, *Thulin et al.* 9559) and the Dhofar region of Oman (*Miller & Whitcomb* 2050, *Miller & Nyberg* 9288).

##### Note.

The type of subsp. dhofaricus (*Miller & Nyberg* 9288) is an exceptionally villous specimen but there exist many intermediate specimens with more typical *Convolvulus hystrix* and we do not think this taxon merits recognition. There are also plants with strikingly sericeous stems and leaves in Dhofar (*Miller* 6326a).

#### 104b. Convolvulus hystrix subsp. ruspolii

Taxon classificationPlantaeSolanalesConvolvulaceae

(Dammer ex Hallier f.) J.R.I.Wood & R.W.Scotland stat. nov.

urn:lsid:ipni.org:names:77147674-1

Convolvulus ruspolii Dammer ex Hallier f., Ann. Reale Inst. Bot. Roma 7: 225. 1898. (Hallier 1898b: 225). Type. ETHIOPIA, Ogaden, *Riva* 297 (holotype FT!).

##### Type.

Based on *Convolvulus ruspolii* Dammer ex Hall f.

##### Distinguishing features.

Similar to subsp. hystrix in armature but the spine-like side branches rather slender. Stems glabrous to shortly pubescent. Leaves 5–11 × 1–2 mm, oblong or lanceolate to elliptic, glabrous to puberulent or sericeous. Flowers usually solitary, sometimes in pairs forming a narrowly oblong-lanceolate head twice as long as broad; bracteoles 3–3.5 × 1–1.5 mm, oblong, oblanceolate or obovate, longer than broad; corolla 0.8–1 cm long. [Sebsebe 2006: 182]

##### Distribution.

Ogaden region of Ethiopia (*Hemming* 1530) and Somalia (*Popov* 1008; *Glover & Gilliland* 636; *Thulin & Warfa* 5562; *Bally* 10164).

##### Notes.

Some specimens from Somalia (*Gillett, Hemming & Watson* 21885, *Bally & Melville* 16271, *Thulin & Warfa* 5562, *Kasmi et al.* 850) have ellipsoid flower clusters with 2–4 flowers and are intermediate with subsp. hystrix and suggestive of hybrid origin.

#### 104c. Convolvulus hystrix subsp. inermis

Taxon classificationPlantaeSolanalesConvolvulaceae

(Chiov.) J.R.I.Wood & R.W.Scotland stat. nov.

urn:lsid:ipni.org:names:77147676-1

Convolvulus hystrix forma inermis Chiov., Fl. Somala 1: 230. 1929. (Chiovenda 1929: 230). Type. SOMALIA, Nogol, *Puccioni & Stefanini* 942 (holotype FT!).

##### Type.

Based on Convolvulus hystrix forma inermis Chiov.

##### Distinguishing features.

Plant virtually unarmed, the side branches long, relatively slender, spinescent when old, short lateral branchlets absent or, when present, neither rigid nor spine-like. Indumentum and flower heads similar to subsp. hystrix.

##### Distribution.

Bari and Nogol regions of NE Somalia (*Hansen & Heemstra* 6297, *Becket* 687, *Nugent* 33, *Hemming* 1866).

##### Notes.

Occasional specimens intermediate with subspecies hystrix are known from the same region (*Lavranos & Carter* 24645, *Bally & Melville* 15450).

Two other specimens from NE Somalia (*Beckett* 42, *Thulin et al*. 9488) are close to both Convolvulus hystrix subsp. inermis and *Convolvulus scopulatus*. They are similar in habit to both species but the stems and leaves are sericeous, the leaves oblong-lanceolate and the bracteoles similar to the leaves but acuminate to a fine point. They may represent an undescribed species or a form of *Convolvulus hystrix* or *Convolvulus scopulatus*.

#### 105. Convolvulus socotranus

Taxon classificationPlantaeSolanalesConvolvulaceae

Verdc., Kew Bull. 12: 344. 1957. (Verdcourt 1957: 344).

Figure 14, t. 37–43

Breweria fastigiata Balf.f., Proc. Roy. Soc. Edinburgh 12: 83. 1884, non *Convolvulus fastigiatus* Roxb. (1832). (Balfour 1883: 83). Type. SOCOTRA, *Balfour et al.* 73 (lectotype K, designated by Sebsebe Demissew in Sebsebe et al. 2009: 230); isolectotypes BM, E, OXF).

##### Type.

Based on *Breweria fastigiata* Balf.f.

##### Description.

Fastigiate undershrub reaching 60 cm in height but more in width, silvery-sericeous when young with most hairs appressed but some spreading, glabrescent and becoming brown when old; branches rigid but not spinescent. Leaves sessile, 2–6 × 0.5–1 (-2) mm, linear-lanceolate, acute, entire, cuneate at the base, sericeous. Flowers solitary, axillary, sessile; bracts resembling small leaves; bracteoles 1.5 × 0.5 mm, ovate, acute; sepals 5–7 × 2–3 mm, ovate to elliptic, concave, the apex acute, bent outwards, pubescent; corolla 5.5–7 mm long, white, midpetaline bands pilose; filaments glandular below; ovary glabrous; style glabrous, divided 3–4 mm above base, arms somewhat unequal 0.5–1 mm long, stigmas 0.5 mm, obovoid. Capsule 1-seeded, glabrous, seeds smooth, glabrous.

##### Distribution.

Endemic to Socotra (*Thulin & Gifri* 8954, *Popov* 50/111, *Smith & Lavranos* 101).

#### 106. Convolvulus kossmatii

Taxon classificationPlantaeSolanalesConvolvulaceae

Vierh., Denkschr. Akad., Wien, Math.-Naturwiss Kl. 71: 416. 1907. (Vierhapper 1907: 416)

Breweria spinosa Vierh., Österr. Bot. Zeit. 54: 287. 1904. (Vierhapper 1904: 287). Type. SOCOTRA, Abd al Kuri Island, *Paulay* s.n. (lectotype WU, designated by R.R. Mill in Sebsebe et al. 2009: 228).
Seddera spinosa (Vierh.) Verdc., Hooker’s Icon. Pl. 7(4): t. 3688.1971. (Verdcourt 1971: 1–3). Type. Based on *Breweria spinosa* Vierh.

##### Type.

Based on *Breweria spinosa* Vierh.

##### Description.

Undershrub to 60 cm, branches fastigiate, sharply spinescent, sericeous when young but later glabrescent. Leaves subsessile, 4–12 × 1–1.5 mm, acute, entire, cuneate at the base, silvery pubescent. Flowers solitary, axillary, subsessile; peduncles absent; bracteoles 2–3 × 0.5 mm, oblong, acute; pedicels c. 1 mm; outer sepals 6 × 3 mm, ovate to elliptic, acute, pubescent on the margins; inner sepals c. 4 mm wide, subrhomboid with hyaline margins; corolla 10–11 mm long, white, midpetaline bands pubescent; filaments glandular basally; ovary glabrous, style glabrous, divided c. 8 mm above base; style arms 0 and 0.5 mm, unequal, stigmas subglobose. Capsule and seeds not known.

##### Distribution.

Endemic to Abd al Kuri Island in the Socotra group (*Smith & Lavranos* 645, *Miller* 11444).

#### 107. Convolvulus semhaensis

Taxon classificationPlantaeSolanalesConvolvulaceae

(R.R.Mill) J.A.Luna & Carine, Phytotaxa 156(1): 51. 2014. (Luna et al. 2014).

Seddera semhaensis R.R.Mill, Kew Bull. 64: 231. 2009. (Sebsebe et al. 2009: 231). Type. SOCOTRA, Semha Island, *Miller* 11461A (holotype E, not seen).

##### Type.

Based on *Seddera semhaensis* R.R.Mill

##### Description.

Grey undershrub to 50 cm, branches straight and rigid, densely grey canescent when young, becoming more sparsely hirsute with age and reddish-brown in colour. Leaves shortly petiolate, 2.5–8 × 0.5–1.5 mm, narrowly lanceolate, subacute, cuneate at base, densely grey-sericeous; petioles 0.5–1 mm. Flowers solitary, axillary, subsessile; peduncle absent; bracteoles 4–5 × 1.5 mm, narrowly oblong-elliptic, densely pilose; pedicels 0.5 mm; outer sepals 6–6.5 × 1.5 mm, oblong, acuminate; inner sepals shorter, scarious; corolla 7–8.5 mm long, white faintly flushed pink, midpetaline bands long-sericeous to base; filaments glandular at base; ovary pilose; style divided 4 mm above base, stigmas 0.75 mm, obovoid. Capsule pilose at apex; seeds glabrous.

##### Distribution.

Endemic to Semha Island in the Socotra islands of Yemen.

##### Notes.

*Miller* 12511, the only specimen we have seen of this species, has a glabrous ovary and style.The sepals are shorter and narrower than in *Convolvulus kossmatii* but the differences are not very great. These two species are very close and may prove to be conspecific.

### Species 108–131. Mostly Middle Eastern species with a fastigiate habit

All species in this group (Clade I of Figure 1) belong to the second major clade within *Convolvulus*, in which the leaves are not distinctly petiolate. Most are more or less fastigiate in form with stiff woody branches, which are not spiny in character (although *Convolvulus erinaceus* is sometimes interpreted as spiny) but there are important exceptions. *Convolvulus verdcourtianus* (not sampled) and *Convolvulus trabutianus* are spiny undershrubs while *Convolvulus rottlerianus*, *Convolvulus prostratus*, *Convolvulus pilosellifolius*, *Convolvulus grantii* and, often, *Convolvulus sarmentosus* are herbaceous. *Convolvulus rottlerianus* is unique in being annual.

#### 108. Convolvulus verdcourtianus

Taxon classificationPlantaeSolanalesConvolvulaceae

Sebsebe, Kew Bull. 48: 381. 1993. (Sebsebe 1993: 209).

##### Type.

SOMALIA, *Bally & Melville* 15583 (holotype K).

##### Description.

Spiny undershrub, appressed pubescent/strigose in its vegetative parts, branches spinescent; sterile spines also present. Leaves sessile, 10–35 × 1–2.5 mm, linear to narrowly lanceolate, obtuse, cuneate at the base. Flowers borne on usually paired spinescent side branches 1.5–3 cm long, the individual flowers solitary or paired arising in the axils of minute bracteoles; bracteoles 0.5 × 0.3 mm, oblong, caducous; pedicels 1–2 mm long; outer sepals 3–4.5 × 1.5–2 mm, oblong to lanceolate, apex obtuse and apiculate; corolla 8 –11 mm long, white, sometimes flushed pink or pale blue, very shallowly lobed, midpetaline bands pubescent; ovary glabrous; style glabrous, divided 1.5–3.5 mm above base; stigmas 3.5–4.5 mm, linear. Capsule glabrous; seeds shortly pubescent, smooth.

##### Distribution.

Endemic to northern Somalia (*Becket* 736).

##### Notes.

Superficially rather similar to *Convolvulus hystrix* but distinguished by the tiny bracteoles, cuneate-based leaves and pubescent seeds. Perhaps more significant are the long, linear stigmas, which contrast strongly with the short, narrowly elliptic stigmas of *Convolvulus hystrix*.

#### 109. Convolvulus trabutianus

Taxon classificationPlantaeSolanalesConvolvulaceae

Schweinf. & Muschl., Repert. Spec. Nov. Regni Veg. 9: 566. 1911. (Schweinfurth and Muschler, 1911: 566).

Figure 15, t. 1–7

Convolvulus ifniensis Caball., Trab. Mus. Ci. Nat., ser. Bot. 30: 7. 1935. (Caballero 1935: 7). Type. MOROCCO, Ifni, *Caballero* s.n. (lectotype MA, designated by Sa’ad 1967: 71).

##### Type.

ALGERIA, Oran, *Diels* s.n. (holotype B†).

##### Description.

Intricate, nearly leafless, spiny undershrub to 50 cm, stems and branches spinescent, branches arising at right angles, stems and vegetative parts glabrous to strongly adpressed pubescent. Leaves alternate on younger shoots but commonly clustered on very short thick brachyblasts on older shoots, sessile, 0.4–2.2 × 0.1–0.4 cm, oblanceolate, obtuse, entire, attenuate at the base. Flowers solitary (very rarely paired), axillary but sometimes appearing to be in clusters on brachyblasts from which the leaves have fallen; peduncles 0–6 mm, woody, persistent and spinescent; bracteoles c. 1 mm long, scale-like; pedicels 1–3.5 mm, commonly recurved; calyx somewhat globose, sepals similar, 4–5 × 2.5–3 mm, elliptic, rounded and minutely mucronulate, pubescent; corolla 1.4–1.6 cm, white, very shallowly lobed, midpetaline bands pilose, pink; ovary very sparsely pilose; style sparsely pilose below, divided 5 mm above base, stigmas 4–6 mm, somewhat unequal. Capsule and seeds not seen. [Sa’ad 1967: 71 p. p.]

**Figure 15.**
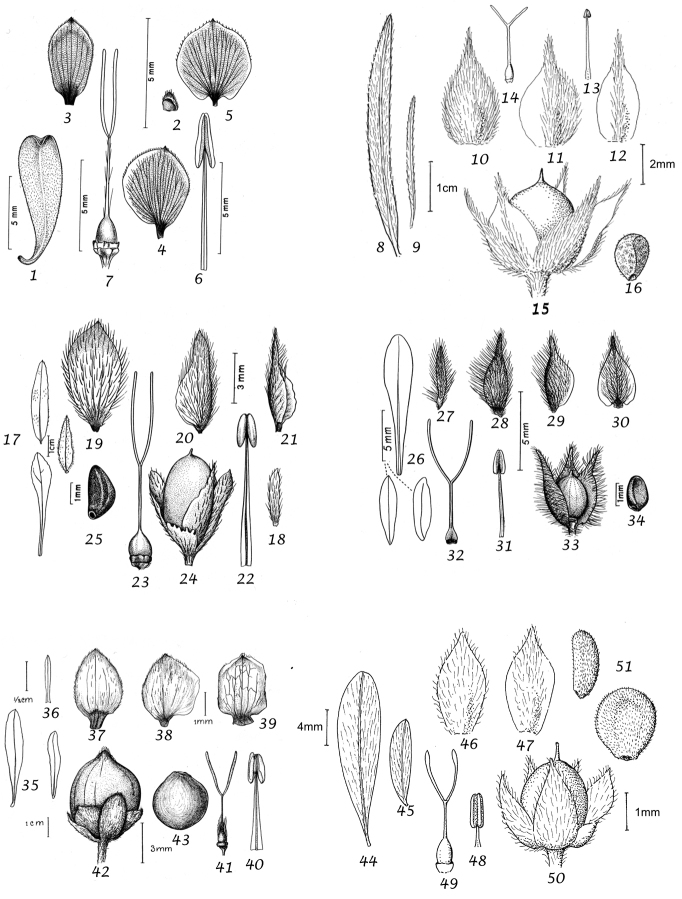
**1–7** *Convolvulus trabutianus* **1** leaf **2** bracteole **3** outer sepal **4** middle sepal **5** inner sepal **6** stamen **7** ovary and style. From *Raymond* 32 (RAB) **8–16** Convolvulus rottlerianus subsp. rottlerianus **8** leaf **9** bracteole **10** outer sepal **11** middle sepal **12** inner sepal **13** stamen **14** ovary and style **15** capsule **16** seed. From *Wight* s.n. (OXF) **17–25** *Convolvulus pilosellifolius* **17** leaves **18** bracteole **19** outer sepal **20** middle sepal **21** inner sepal **22** stamen **23** ovary and style **24** capsule **25** seed **17** from *Bornműller* 1530 (STU) **18–23** from *Bornműller* 1531 (W) **24–25** from *Sintenis* 516 (B) **26–34** *Convolvulus prostratus* **26** leaves **27** bracteole **28** outer sepal **29** middle sepal **30** inner sepal **31** stamen **32** ovary and style **33** capsule **34** seed **26–32** from *Sieber* s.n. (L) **33–34** from *Letourneaux* 280 (W) **35–43** *Convolvulus chondrilloides* **35** leaves **36** bract **37** outer sepal **38** middle sepal **39** inner sepal **40** stamen **41** ovary and style **42** capsule **43** seed **35–41** from *Bornműller* 7640 (W) **42–43** from *Schmid* 6469 (W) **44–51** *Convolvulus sarmentosus* **44** leaf **45** bract **46** outer sepal **47** inner sepal **48** stamen **49** ovary and style **50** capsule **51** seeds. From *Miller et al.* 10221 (K).

##### Distribution.

Restricted to the Maghreb of northwestern Africa: Mauritania (*Chevalier* s.n., *Monod* 19597); Morocco (*Lewalle* 9758, *Jury et al.* 14457, 19615, 20738, *Jehandiez* 156, *Davis* 48684, *Allorge* s.n. [17/4/1913], *Podlech* 49240); Algeria (*Humbert* s.n. [4/1924]).

##### Notes.

A rather variable plant, sometimes glabrous, sometimes densely adpressed pubescent so approaching *Convolvulus caput-medusae* in this respect; flowers can be solitary and pedunculate (e.g. *Davis* 48684), when the spine-like peduncles persist, but may also develop in apparent pedicellate clusters on virgate brachyblasts. These differences merit further study.

Sa’ad (1967) treated *Convolvulus trabutianus* under *Convolvulus caput-medusae* (it is *Convolvulus trabutianus* that is illustrated under the name *Convolvulus caput-medusae*) but the two plants are distinct. *Convolvulus caput-medusae* is always white-sericeous, only the branches are spinescent, the flowers are more or less sessile and only 8–10 mm long, amongst many differences.

#### 110. Convolvulus rottlerianus

Taxon classificationPlantaeSolanalesConvolvulaceae

Choisy, Mém. Soc. Phys. Genève 6: 477. 1834. (Choisy 1834: 477).

##### Type.

INDIA, *Rottler* s.n. in *Wallich* 6669 (lectotype G-DC!, designated here; isolectotype K-W!).

##### Description.

Annual herb from a thin tap root, commonly branched at base with erect or ascending stems, 10–45 cm high, vegetative parts adpressed pilose, occasionally with some stiff spreading hairs. Leaves mostly on lower part of stem, sessile, 1.5–3.5 (-7.5) × 0.2–0.6 (-1) cm, lanceolate, oblong or narrowly oblong-lanceolate, mucronate, entire, gradually narrowed to a pseudopetiolate base. Flowers 1–3 in axillary pedunculate cymes; peduncles 1–5 cm, ascending; bracteoles 2–5 × 0.5 mm, fiiform to linear-lanceolate; pedicels 2–5 mm; outer sepals 4–6 × 2–2.5 mm, ovate to elliptic, acuminate and mucronate, glabrous or adpressed pilose; inner sepals slightly smaller, glabrous; corolla 0.7–1.2 cm long, pink, distinctly lobed, midpetaline bands pilose; ovary glabrous; style glabrous, divided 2–4 mm above base, stigmas 3 mm long. Capsule glabrous; seeds pubescent with patches of adpressed hairs.

##### Notes.

We recognise two subspecies:

#### 110a. Convolvulus rottlerianus subsp. rottlerianus

Taxon classificationPlantaeSolanalesConvolvulaceae

Figure 15, t. 8–16

Convolvulus gilbertii Sebsebe, Kew Bull. 54: 74. 1999. (Sebsebe 1999: 74). Type. Ethiopia, *Gilbert et al.* 8290 (holotype ETH; isotype K!).

##### Distinguishing features.

Outer sepals adpressed pilose. [Austin and Ghazanfar 1979: 27, 25 (plate)]

##### Distribution.

India, (*Rottler* s.n. [18/11/1795], *Perrotet* 888, *Wight* 2234, 2293, *Talbot* 2177, *Deane* 107, *Beddome* 5608/9), Pakistan, Ethiopia (*Corrá* 8, *Friis et al.* 3688).

##### Notes.

*Convolvulus rottlerianus* has only recently been found in Ethiopia and is unknown in Iran and Arabia. Ethiopian material was described as a separate species, *Convolvulus gilbertii*, probably because the possibility of the occurrence of an Indian species was not considered. No difference between specimens from the two areas can be discerned. Little is known about the habitat of this subspecies in India but it is found in drier areas. In Ethiopia it is a rare plant of Acacia bushland.

#### 110b. Convolvulus rottlerianus subsp. stocksii

Taxon classificationPlantaeSolanalesConvolvulaceae

(Boiss.) J.R.I.Wood & R.W.Scotland comb. et stat. nov.

urn:lsid:ipni.org:names:77147677-1

Convolvulus tenellus Stocks, Hooker’s J. Bot. Kew Gard. Misc. 4: 172. 1852, nom. illeg., non *Convolvulus tenellus* Desr. (1792). (Stocks 1852: 172). Type. PAKISTAN, Balochistan, *Stocks* s.n. (K!).
Convolvulus stocksii Boiss., Fl. Orient. [Boissier] 4: 110. 1875. (Boissier 1875b: 110). Type. Based on *Convolvulus tenellus* Stocks
Convolvulus rottlerianus var. tenellus (Stocks) C.B.Clarke, Fl. Brit. India [J.D. Hooker] 4: 219. 1883. (Clarke 1883: 219). Type. Based on *Convolvulus tenellus* Stocks

##### Type.

Based on *Convolvulus tenellus* Stocks

##### Distinguishing features.

Outer sepals glabrous.

##### Distribution.

Endemic to Pakistan: Balochistan and Sind (*Dalziel* 57). Apparently replaces subsp. rottlerianus in Balochistan and perhaps in parts of Sind.

#### 111. Convolvulus prostratus

Taxon classificationPlantaeSolanalesConvolvulaceae

Forssk., Fl. Aegypt.-Arab. 203. 1775. (Forsskål 1775: 203).

Figure 15, t. 26–34.

Ipomoea microphylla Roth ex Schult., Syst. Veg., ed. 15 bis [Roemer & Schultes] 4: 248. 1819. (Roemer and Schultes 1819: 248). Type. INDIA, *Heyne* s.n. (B†).
Convolvulus parvifolius Spreng., Syst. Veg. [Sprengel] 1: 611. 1824. (Sprengel 1824: 611). Type. Based on *Ipomoea microphylla* Roth ex Schult.
Convolvulus microphyllus Sieber ex Spreng., Syst. Veg. [Sprengel] 1: 611. 1824. (Sprengel 1824: 611). Type. EGYPT, *Sieber* s.n. (holotype LE; isotypes E!, K!, L).
Evolvulus pilosus Roxb., Fl. Ind. (Carey & Wallich ed.) 2: 106. 1832. (Roxburgh 1832: 106). Type. INDIA, plant cultivated in Calcutta Botanic Garden (whereabouts unknown).
Convolvulus pluricaulis Wall. ex Choisy, Mém. Soc. Phys. Genève 6: 477. 1834. (Choisy 1834: 477). Type. INDIA, “montes Hindostania meridionalis 1825”, *Wallich* 1316 (lectotype G 00135972!, sheet annotated “*Convolvulus pluricaulis* Ch.”, designated here).
Convolvulus evolvuloides Boiss., Diagn. Pl. Orient. 7: 25. 1846, nom. illeg., non *Convolvulus evolvuloides* Desf. (1798). (Boissier 1846: 25) Type. IRAN, Makran, *Aucher-Eloy* 4953 (holotype G; isotypes K!, BM!).
Convolvulus scindicus Boiss., Diagn. Pl. Orient., ser. 2, 3: 123. 1856, nom. illeg., non *Convolvulus scindicus*Stocks (1852). (Boissier 1856: 123). Type. PAKISTAN, Sind, *Stocks* (holotype ?G, possible isotypes K!).
Convolvulus microphyllus var. boissieri C.B.Clarke, Fl. Brit. India [J.D. Hooker] 4: 218. 1883. (Clarke 1883: 218). Type. Based on *Convolvulus scindicus* Boiss.
Convolvulus prostratus var. boissieri (C.B.Clarke) Parmar, J. Econ. Taxon. Bot. 18: 251.1994. (Parmar 1994: 251). Type. Based on *Convolvulus scindicus* Boiss.
Convolvulus pluricaulis var. macra C.B.Clarke, Fl. Brit. India [J.D. Hooker] 4: 218. 1883. (Clarke 1883: 218). Type. INDIA, Falconer s.n. (lectotype K, sheet labelled “*Evolvulus alsinoides* var.” and annoted Convolvulus pluricaulis var. macra” in Clarke’s handwriting, designated here).
Convolvulus deserti Hochst. & Steud. ex Baker & Rendle, Fl. Trop. Africa (Oliver et al.) 5(2): 92. 1905. (Baker and Rendle 1905: 92). Type. SAUDI ARABIA, Jeddah, *Schimper* 783 (syntypes BM 000049019!, GOET, HAL, HBG, K!, L, OXF!, P!, STU, W!).
Convolvulus microphyllus var. longipes Maire, Bull. Mus. Natl. Hist. Nat. 22: 535. 1931. (Maire 1931b: 535). Type. Central Sahara (ALGERIA?), *Mauod* 312 (holotype AL?; isotype P00417710!).
Convolvulus heterotrichus Maire, Bull. Soc. Hist. Nat. Afrique N. 26: 159.1935. (Maire 1935: 159). Type. Western Sahara, *Lutherear* s.n. (holotype ?AL; isotypes MPU006233!, P00434263!).
Convovulus microphyllus var. heterotrichus (Maire) Maire, Bull. Soc. Hist. Nat. Afrique N. 27: 251. 1936. (Maire 1936: 251). Type. Based on *Convolvulus heterotrichus* Maire
Convolvulus austroaegyptiacus Abdallah & Sa’ad, Acta Bot. Neerl. 15: 190. 1966. (Abdallah and Sa’ad 1966: 190). Type. EGYPT, *Abdallah* s.n. (holotype U; isotypes CAI, CAIM, K!, WAG).
Convolvulus cancerianus Abdallah & Sa’ad in Sa’ad, Meded. Bot. Mus. Herb. Rijks Univ. Utrecht 281: 176. 1967. (Sa’ad 1967: 176). Type. EGYPT, *Abdallah* 1646 (holotype U; isotype CAIM, WAG).
Convolvulus microphyllus var. macra (C.B.Clarke) S.K.Sharma & Tiagi, Fl. N. E. Rajasthan 262 (Sharma and Tiagi 1979: 262). Type. Based on Convolvulus pluricaulis var. macra C.B.Clarke
Convolvulus prostratus var. deserti (Hochst. & Steud. ex Baker & Rendle) Parmar, J. Econ. Taxon. Bot. 18: 251. 1994. (Parmar 1994: 251). Type. Based on *Convolvulus deserti* Hochst. & Steud.
Convolvulus austroaegytiacus var. cancerianus (Abdallah & Sa’ad) Alfarhan, Fl. Kingdom Saudi Arabia 2(2): 166. 2001. (Alfarhan 2001: 166). Type. Based on *Convolvulus cancerianus* Abdallah & Sa’ad

##### Type.

YEMEN, Mor, *Forrskål* s.n. (lectotype C, designated by Sa’ad 1967: 192).

##### Description.

Very variable perennial herb with ascending or prostrate stems up to 70 cm long, vegetative parts thinly to densely pubescent or villous, stems often somewhat rigid and woody when old. Leaves subsessile, 0.8–3 × 0.2–0.6 cm, oblong, linear, lanceolate, narrowly lanceolate or oblanceolate, acute or obtuse, margin entire, cuneate, the lowermost tapered at the base. Flowers 1–3 (-11) in sessile or pedunculate bracteate clusters, these occasionally rather lax with individual flowers clearly separate; bracts leaf-like, exceeding, equalling or shorter than the flowers; peduncles 0–5 (-10) cm long; bracteoles 3–7 × 1–2 mm, filiform, linear to lanceolate or narrowly oblong, sometimes caducous; pedicels 0–3 (-6) mm long; outer sepals 4–8 × 1.5–6 mm, lanceolate to ovate, acuminate, green in the upper half, paler in the lower half; inner sepals caudate; corolla (6-)10–15 mm long, white or pale pink, unlobed, midpetaline bands pilose; ovary glabrous; style glabrous, divided 3–4 mm above the base, stigmas c. 4 mm. Capsule glabrous, seeds smooth, pubescent. [Sa’ad 1967: 192; Nowroozi 2002: 105 (map); Collenette 1999: 230 photo); Austin and Ghazanfar 1979: 23, 21 (plate); Jongbloed 2003: 314 (photo)]

##### Distribution.

A characteristic Saharo-Sindian species, apparently absent from Palestine/Israel, Syria, most of Iraq and countries north of Iran: Cape Verde Islands (Lobin 1986: 155); Senegal (*Raynall* 5778); Mauritania (*Monod* 18317, *Adam* 19415-2); Niger (*Popov* 41, *Monod* 13855); Chad; Algeria (*Monod* 312); Sudan (*Kotschy* 354, *Schweinfurth* 2149); Libya (fide Greuter et al. 1986); Egypt (*Letourneaux* 2802, *Schweinfurth* 138); Djibouti (*Mosnier* 718); Somalia (*Thulin & Warfa* 5800); Saudi Arabia (*Trott* 1458, *Zohrab* 107, *Collenette* 2418, 7887); Qatar (*Mandaville* 4109); Oman (*Lawton* 2052, *Mandaville* 7329); UAE (*Borosova et al.* 107); Yemen (*Deflers* 34, *Lunt* 158); Iraq (Rechinger 9353); Iran (*Furlonge* 15, *Aucher-Eloy* 1234); Afghanistan (*Griffith* 681); Pakistan (*Lace* 3397, *Popov* 63/413); India (*Popov* 63/380).

##### Notes.

*Convolvulus prostratus* is easily confused with *Convolvulus pilosellifolius* particularly in the Arabian peninsular. It is best distinguished by the lanceolate to ovate outer sepals which taper to an acuminate apex. In contrast, in *Convolvulus pilosellifolius* the outer sepals are oblong-oblanceolate, widest above the middle, and merely acute. Records of *Convolvulus pilosellifolius* from Yemen and much of the Persian/Arabian Gulf are probably errors for *Convolvulus prostratus*.

*Convolvulus prostratus* is a polymorphic species and the extremes can look very different. Variation is particularly complex in the Arabian peninsula. At least some of the variation is explained by response to rainfall (Wood 1997: 233–234) but some of it is geographically related. The following are among the more distinct forms but it should be emphasised that all kinds of intermediates occur.

In the types of *Convolvulus prostratus* and *Convolvulus microphyllus* the flower clusters are compact, 1–3-flowered, subsessile or at most shortly pedunculate. The flower clusters appear lanceolate in form and relatively small. This is the common form in Africa, where other forms are rare or absent. Similar forms occur across Arabia to India.

In India, Pakistan and Afghanistan plants similar to (i) occur commonly but a second form corresponding to the type of *Convolvulus pluricaulis* is also frequent. This differs in the presence of prominent, narrowly oblong to oblanceolate bracts reaching to the apex of the stem thus resembling a perennial form of *Convolvulus rottlerianus*. These forms are virtually restricted to the Indian subcontinent but occur very rarely elsewhere, such as *Heudelot* 403 from Senegal. Examples include *Haines* 3412, *Thomson* 72, *Clarke* 28110 and *Mooney* 2025 from India, *Duthie* 7177, *Stewart* 7037, *Drummond* 14701 and *Popov* 63/413 from Pakistan and *Griffith* 681 from Afghanistan.

In Arabia and rather rarely elsewhere there occur plants which accord with the types of *Convolvulus austroaegyptiacus* and *Convolvulus cancerianus*. These are usually vigorous plants with stout, somewhat woody stems, the hairs spreading on the leaves and stems and the inflorescence rather lax so individual flowers are visible as in *Convolvulus pilosellifolius*. In some examples the flowers are very numerous, up to at least 10. Examples include *Collenette* 909 and *Chaudhary* 6696 from Saudi Arabia, *Willcox* 216, *Lumley* 50 and *Borosova et al.* 138 from U.A.E., *Lawton* 2390 from Oman and *Wood* 3427 and *Thulin et al.* 8303 from Yemen. These forms are rare elsewhere but occur in Egypt and Iran (*Wright & Bent* 503-103).

Occasional plants with spinescent branches occur. These appear to be restricted to Arabia and include *Boulos* 10931 from Qatar and *Collenette* 4143 from Saudi Arabia.

Plants with very woody stems which could be treated as shrubs occur occasionally, particularly in Oman. Good examples are *Miller & Whitcombe* 2031 & 2049 from Oman, but *Collenette* 9077 & 9150 from Saudi Arabia and *York & El-Keblawy* 55 from UAE are also distinctly woody.

Dwarf forms with very small leaves occur on Bahrain (*Naguib* 72, *Fernandez* 373, *Good* 196, *Cornes* 304) and Farasan Island in the Red Sea (*Collenette* 8993). These may be the result of arid conditions or salt spray and may constitute an ecotype similar to var. pumilus of *Convolvulus oleifolius*.

There is much variation in the number of flowers in each cluster but occasional plants occur where the flowers are solitary. Examples include *Schweinfurth* 2150 from Egypt, *Trott* 225 from Saudi Arabia and *Borosova* 107 from U.A.E.

No attempt has been made here to give formal names to any of this variation but it is very clear that the *Convolvulus prostratus* complex merits much more detailed study.

#### 112. Convolvulus pilosellifolius

Taxon classificationPlantaeSolanalesConvolvulaceae

Desr., Encycl. [Lamarck et al.] 3: 551. 1792. (Desrousseaux 1792: 551).

##### Type.

“Levant”, *Vaillant & Tournefort* (holotype P-Juss!).

##### Description.

Perennial herb with a woody taproot, stems decumbent or ascending to 70 cm, plant usually thinly adpressed pubescent but sometimes villous. Lower leaves with petioles to 5 cm, narrowly oblanceolate, obtuse, stem leaves sessile, 1.5–9 × 2–1.5 cm, linear, lanceolate or oblong, acute, margin entire or wavy, attenuate at base. Flowers pedunculate, sometimes solitary but mostly clearly separated and arranged in somewhat congested, pedunculate monchasial or diachasial cymes; peduncles (1-) 3–5 (-10) cm. much exceeding the subtending bracts; bracteoles 3–6 × 1–2 mm, linear or narrowly oblong; pedicels 1.5–6 mm; sepals with green herbaceous acute, upper part, outer sepals 5–8 × 3–5 mm, oblong-oblanceolate, acute, inner sepals ovate, caudate, corolla 10–15 mm, pink or white, unlobed (or, rarely, divided to base), midpetaline bands pubescent; ovary and style glabrous; style divided 3–4 mm above base, stigmas c. 4 mm; capsule glabrous, seeds smooth, shortly pubescent. [Feinbrun-Dothan 1978: plate 61; Nowroozi 2002: 79 (plate), 105 (map); Jongbloed 2003: 313 (photo); Collenette 1999: 230 (photo); Austin and Ghazanfar 1979: 22]

##### Notes.

We recognise two varieties of this species:

#### 112a. Convolvulus pilosellifolius var. pilosellifolius

Taxon classificationPlantaeSolanalesConvolvulaceae

Figure 15, t. 17–25

Convolvulus piloselloides D.Dietr., Syn. Pl. 1: 677.1839, lapsus [spelling mistake] for *Convolvulus pilosellifolius* Desr. (Dietrich 1839: 677).
Convolvulus sogdianus Bunge, Beitr. Fl. Russl. 219. 1852. (Bunge 1852: 395). Type. UZBEKISTAN, Bukhara, *Lehmann* s.n. (holotype LE!; isotype P!).
Convolvulus ammocharis Boiss. & Hausskn., Pl. Orient. Nov. (dec. prim.) 1: 6. 1875. (Boissier 1875a: 6). Type. IRAN, Bushehr, *Haussknecht* s.n. (holotype G; isotypes BM001014570!, P03551021!, W!).
Convolvulus ammophilus St.-Lag, Ann. Soc. Bot. Lyon 7: 123. 1880, illegitimate superfluous name for *Convolvulus ammocharis* Boiss. & Hausskn. (Saint-Lager 1880: 123). Type. Based on *Haussknecht* s.n.
Convolvulus bornmuelleri Hausskn., Mitth. Thüring. Bot. Vereins n.f., 6: 56. 1894. (Bornmüller 1894: 56). Type. IRAN, Bandar Abbas, *Bornmüller* 468 (holotype B†; isotype K!).
Convolvulus pilosellifolius var. leiocalycinus Sa’ad, Meded. Bot. Mus. Herb. Rijks Univ. Utrecht 281: 191. 1967. (Sa’ad 1967: 191). Type. TURKEY, Gazintep, *Haussknecht* s.n. (holotype P).
Convolvulus sistanicus de Marco & Dinelli, Ann. Bot. (Rome) 35–36: 427. 1978 [1976-7]. (Dinelli and de Marco 1978: 427). Type. IRAN, Sistan Depression, *de Marco* 75044 (holotype URT!).
Convolvulus chaudharyi Alfarhan, Brittonia 45: 170. 1993. (Alfarhan 1993: 170). Type. SAUDI ARABIA, Jiddah, *Collenette* 6961 (holotype RIY; isotypes E!, K!).

##### Distinguishing features.

Leaves lanceolate or oblong, at least 2 cm wide. Inflorescence usually of several flowers.

##### Distribution.

Middle East and Central Asia but apparently absent from the Indian subcontinent and Africa, apart from Egypt and Libya; in Arabia rare and absent from the south: Libya (*Guichard* Lib/356); Egypt (*Simpson* 6073); Jordan (*Townsend* 65/389, *Trought* 21/5/1952); Palestine/Israel (*Meyers & Dinsmore* 3632); Syria (*Haradjian* 3433); Turkey; Iraq (*Barklay et al.* 5055, *Guest* 2475, *Rustam* 3492); Kuwait (*Boulos* 16221, *Rawi et al.* 10774); U.A.E. (*Western* 551); Saudi Arabia (*Collenette* 2521, 4443); Iran (*Haussknecht* s.n. [5/1867], *Rechinger* 4118, *Davis & Bokhari* 56081); Azerbaijan (*Grossheim & Schiskin* 315); Uzbekistan (*Androssow* s.n. [27/6/1901], *Vvedensky* 443 in Herb. Fl. As. Med); Turkmenistan (*Litwinow* 1644, *Nevski* 300), Kirgizstan (*Lazkov* s.n.); Tajikistan (*Korshinsky* 3299); Kazakhstan (*Litvinov* s.n. [23/5/1899]); Afghanistan (*Griffith* 680, *Koie* 2884); Pakistan: Balochistan (*Ghafoor & Goodman* 5076).

##### Notes.

*Convolvulus chaudharyi* is a form with the corolla lobes divided nearly to the base.

#### 112b. Convolvulus pilosellifolius var. linearifolius

Taxon classificationPlantaeSolanalesConvolvulaceae

Sa’ad, Meded. Bot. Mus. Herb. Rijks Univ. Utrecht 281: 191. 1967. (Sa’ad 1967: 191).

##### Type.

IRAN, Bushehr, *Koie* 268 (holotype B).

##### Distinguishing features.

This is a delicate form with linear-lanceolate leaves to 2 cm wide and mostly solitary flowers.

##### Distribution.

The occasional specimen of this variety occurs throughout most of the range of *Convolvulus pilosellifolius* but especially around the Arabian/Persian Gulf, such as *Good* 193 from Bahrain.

##### Notes.

*Convolvulus pilosellifolius* can be confused with *Convolvulus cantabrica*, especially forms of that species previously assigned to *Convolvulus linearis*, but differs in the shorter corolla (to 15 mm) and glabrous ovary and capsule.

#### 113. Convolvulus chondrilloides

Taxon classificationPlantaeSolanalesConvolvulaceae

Boiss., Diagn. Pl. Orient. 11: 83. 1849. (Boissier 1849: 83).

Figure 15, t. 35–43

##### Type.

IRAN, *Aucher-Eloy* 4941 (lectotype G, designated by Sa’ad 1967: 87); isolectotypes P!, W!).

##### Description.

Erect perennial undershrub with a woody base and glabrous, herbaceous branches to at least 1 m, nearly leafless above. Leaves sessile, somewhat dimorphic, basal leaves 2–3.5 × 0.5–1 cm, spathulate to oblanceolate with a long petiole-like base; stem leaves 2.5–3 × 0.2–2 cm, linear to lanceolate, acute, entire, base cuneate. Flowers in a large, nearly leafless, branched terminal inflorescence composed of axillary cymes of 1–6-flowered cymes, the flowers irregularly grouped; ultimate branches rather slender, not rigid; bracts 7 × 1 mm, linear, bracteoles minute; pedicels 0–10 mm, densely pubescent; sepals 2–4 × 1.5–4 mm, ovate to suborbicular, mucronulate, pubescent, the inner sepals broader, scarious; corolla 1.3 cm long, white, midpetaline bands pilose; ovary pilose; style glabrous, divided c. 3 mm above base; stigmas 3 mm. Capsule pubescent at apex, 1-seeded; seeds glabrous, black, smooth. Sa’ad 1967: 87; Nowroozi 2002: 55 (plate), 102 (map).

##### Notes.

We recognise two varieties of which only var. chondrilloides is common:

#### 113a. Convolvulus chondrilloides var. chondrilloides

Taxon classificationPlantaeSolanalesConvolvulaceae

Evolvulus virgatus Choisy, Prodr. [A.P. de Candolle] 9: 446. 1845, nom illeg., non *Evolvulus virgatus* Willd. ex Spreng. 1824. (Choisy 1845: 446). Type. IRAQ, Baghdad, *Aucher-Eloy* 1410 (holotype G-DC).
Convolvulus chondrilloides subsp. eriocalycinus Bornm. & Gauba, Repert. Spec. Nov. Regni Veg. 51: 215. 1942. (Bornmüller and Gauba 1942: 215). Type. IRAN, Karaj, *Gauba* 1624 (holotype B†).

##### Distinguishing features.

Sepals pubescent.

##### Distribution.

Turkey (*Davis* 45425); Iraq (*Wheeler-Haines* 338, *Bornmüller* 1534), Iran (*Davis & Bokhari* 56214, *Jacobs* 4787, *Schmid* 6469, *Rechinger* 382, *Bornmüller* 7640).

#### 113b. Convolvulus chondrilloides var. burzianus

Taxon classificationPlantaeSolanalesConvolvulaceae

Sa’ad, Meded. Bot. Mus. Herb. Rijks Univ. Utrecht 281: 87. 1967. (Sa’ad 1967: 87).

##### Type.

IRAN, Tehran, *Koelz* 16059 (holotype W!).

##### Distinguishing features.

Distinguished by the glabrous sepals

##### Distribution.

Apparently very rare and reported only from Iran.

#### 114. Convolvulus sericophyllus

Taxon classificationPlantaeSolanalesConvolvulaceae

T.Anderson, J. Proc. Linn. Soc. Bot. 5 (suppl. 1): 25. 1860. (Anderson 1860: 25).

Figure 14, t. 21–28

Convolvulus acicularis Vatke, Linnaea 43: 518. 1882. (Vatke 1882: 518). Type. SOMALIA, *Hildebrandt* 885 (holotype B†; isotype BM!).
Convolvulus somalensis Franch., Sert. Somal. 43. 1882, nom. illeg., non *Convolvulus somalensis*Vatke (1882). (Franchet 1882: 43). Type. SOMALIA, *Revoil* s.n. (P, not seen)

##### Type.

YEMEN, Aden, *Hooker & Thomson* s.n. (holotype K!).

##### Description.

Slender, erect branched shrub 0.5–3 m high, vegetative parts characteristically sericeous to strigose. Leaves subsessile, 4–13(-40) × 1–5 (-10) mm, filiform, oblong to obovate, acute. Inflorescence with the appearance of long narrow branched racemes; flowers in dichasial cymes commonly reduced to clusters of 1 –2(-3), arising from the axils of linear bracts; peduncles 0–3.5 cm long; bracteoles linear 1–5 mm long; pedicels 2–3 mm long; outer sepals 2.5–3.5 × 1.5–2 mm, ovate, acute, glabrous to sericeous; corolla 6–10 mm long, white, weakly lobed, midpetaline bands strigose, red; ovary glabrous, style glabrous, divided 3 mm above base, stigmas 1 mm. Capsule glabrous; seeds pubescent. [Thulin 2006: 233]

##### Distribution.

Yemen: south (*Schweinfurth* 99, *Deflers* 32); Somalia: north (*Gillett* 4490, *Revoil* 75).

##### Notes.

Specimens collected after good rainfall have a rather different morphology with well-developed, obovate lower leaves, *Waring* 57 being a good example.

#### 115. Convolvulus grantii

Taxon classificationPlantaeSolanalesConvolvulaceae

Balf.f., Nat. Hist. Sokotra 524. 1903. (Forbes 1903: 524).

##### Type.

SOCOTRA, Abd al Kuri Island, *Ogilvie-Grant exped.* 13, 45, 46 & 50 (syntypes E!, LIV).

##### Description.

Herb with thickened woody taproot and a basal rosette; stems ascending to c. 30 cm, vegetative parts sericeous and with some spreading hairs. Leaves petiolate, dimorphic; rosette leaves 3.5–4.5 × 0.7–1.4 cm. obovate to oblanceolate-spathulate, obtuse to acute, margin entire to undulate, base attenuate onto the petiole; stem leaves 0.3–1.5(-3) × 0.2–1 cm, acute, oblanceolate, oblong or elliptic, margin deeply sinuate, base attenuate; petioles 2–5 mm long. Flowers solitary, pedunculate; peduncles c. 3 mm long; bracteoles 2 × 0.75 mm, linear; pedicels c. 3.5 mm long; outer sepals 4–4.5 × 2–2.5 mm, ovate, acute or shortly acuminate; corolla 10–12 mm long, white or pinkish, lobed with broadly triangular lobes, midpetaline bands pilose; ovary glabrous; style glabrous, divided c. 2.5–3 mm above base; stigmas 4 mm long. Capsule glabrous; seeds glabrous. [Miller and Morris 2004: 517]

##### Distribution.

Endemic to Abd al Kuri Island in the Socotra archipelago (*Paulay* 1899, *Smith & Lavranos* 660; *Miller et al.* 19032).

##### Notes.

Very distinct because of the rosette habit and small, sinuately-lobed stem leaves. The corolla is slightly larger than in *Convolvulus sarmentosus* and *Convolvulus hildebrandtii*. It is the most herbaceous of the three species.

Miller and Morris (2004: 517) suggest that this species is an annual but this seems to be an error.

#### 116. Convolvulus sarmentosus

Taxon classificationPlantaeSolanalesConvolvulaceae

Balf.f., Proc. Roy. Soc. Edinburgh 12: 83. 1884. (Balfour 1884: 83).

Figure 15, t. 44–51

##### Type.

SOCOTRA, *Balfour et al.* 302 (holotype K!; isotype OXF!).

##### Description.

Perennial herb with numerous decumbent stems to 30 cm long, vegetative parts densely greyish strigose-pubescent when young but indumentum much sparser on older parts. Leaves forming a somewhat persistent basal rosette, sessile, 0.8–2.5 × 0.2–0.8 cm, obovate to oblanceolate, apex obtuse, margin entire, at base attenuate into a pseudopetiole, densely silvery strigose; stem leaves narrower upwards and merging with bracts, thinly strigose. Inflorescence a raceme-like thyrse formed of single-flowered cymes; peduncles 2–10(-16) mm long; bracteoles 1–3 mm long, filiform to narrowly elliptic; pedicels 3–5 mm long; outer sepals 2.5–4 × 1.5–2 mm, ovate, acute, green with scarious margin; corolla (7-)9–11 mm long, white, unlobed, midpetaline bands strigose; ovary glabrous; style white, glabrous, divided c. 2 mm above base, stigmas 2.5 mm, red. [Miller and Morris 2004: 517]

##### Distribution.

Yemen: Socotra (*Miller et al.* 10221, 10191, 19107) and Hadramaut (*Thulin & Gifri* 8652; *Thulin et al.* 8196).

##### Notes.

The sericeous basal leaves separate *Convolvulus sarmentosus* from *Convolvulus hildebrandtii* and the entire leaf margin from *Convolvulus grantii*. Mature specimens without basal parts can be confused with *Convolvulus hildebrandtii* but the stems are herbaceous and “leafy,” the bracts oblong-oblanceolate, rather than linear.

#### 117. Convolvulus hildebrandtii

Taxon classificationPlantaeSolanalesConvolvulaceae

Vatke, Linnaea 43: 519. 1882. (Vatke 1882: 519).

Convolvulus filipes Balf.f., Proc. Roy. Soc. Edinburgh 12: 82. 1884. (Balfour 1884: 82). Type. SOCOTRA, *Balfour et al.* 238 (lectotype K!, designated here).

##### Type.

SOMALIA, Wodderi, *Hildebrandt* 884 (holotype B†; isotype BM001050393!).

##### Description.

Perennial herb, base woody, stems erect, to 70 cm, more or less herbaceous to somewhat woody and fastiagiate, pilose below with hairs up to 3 mm long, becoming strigose upwards, glabrescent when old and woody. Leaves crowded towards the base of the stem, diminishing in size upwards, 1–4 × 0.2–0.4 cm, oblanceolate, acute, gradually narrowed into a long petiole-like base, long-pilose, often absent; stem leaves 1–2.8 × 0.1–0.2 cm, diminishing in size upwards, all linear to very narrowly linear-oblanceolate, apparently caducous. Inflorescence a large, lax terminal thyrse up to 30 cm long, formed of dichasial cymes bearing up to 3 flowers each but commonly reduced to single flowers; bracts on main axis 5–7 mm, linear; peduncles 0.6–6 cm long, becoming shorter on the secondary and tertiary flowers, often single-flowered; bracteoles 1 mm, linear; pedicels 2–4 mm long; outer sepals 2.5–3.5 × 1.5 mm, ovate, acuminate, somehat scarious but with green midrib, inner sepals narrower, strigose; corolla 8–9 mm long, white, weakly lobed, midpetaline bands strigose; ovary 2 mm long, glabrous (*Hildebrandt* 884, *Balfour et al*. 116 or thinly pilose (*Miller et al.* 19120); style glabrous, divided c. 2 mm above base; stigmas c. 3 mm. Capsule glabrous or with a few apical hairs; seeds glabrous to pubescent. [Miller and Morris 2004: 517; Thulin 2006: 234]

##### Distribution.

Somalia (type only); Socotra (*Balfour et al.* 116, *Gwynne* 19, *Thulin & Gifri* 8814 & 8516, *Miller et al.* 10181 & 19120).

##### Notes.

The capsule is only 2.5 mm wide, not 5 mm as described by Thulin (2006).

We agree with Vierhapper (1907) and Miller and Morris (2004) in uniting *Convolvulus filipes* with *Convolvulus hildebrandtii* rather than with Thulin (2006) who kept the two species separate. The striking differences between specimens in stem, leaf, ovary and seed indumentum do not correlate well each other or with geographical distribution.

#### 118. Convolvulus peninsularis

Taxon classificationPlantaeSolanalesConvolvulaceae

J.R.I.Wood & R.W.Scotland sp. nov.

urn:lsid:ipni.org:names:77147661-1

**Figure 16.**
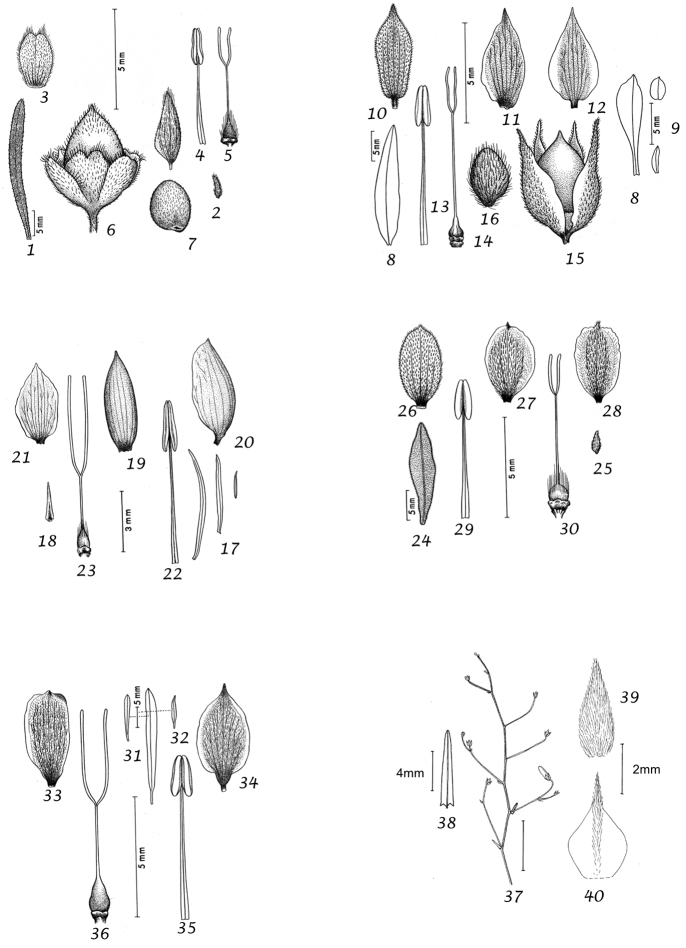
**1–7** *Convolvulus erinaceus* **1** leaf **2** bracteole **3** outer sepal **4** stamen **5** ovary and style **6** capsule **7** seed from *Granitov* 442 (C) **8–16** *Convolvulus eremophilus* **8** leaves **9** bracteole **10** outer sepal **11** middle sepal **12** inner sepal **13** stamen **14** ovary and style **15** capsule **16** seed. From *Rechinger* 1313 (W) **17–23** *Convolvulus koieanus* **17** leaves **18** bracteole **19** outer sepal **20** middle sepal **21** inner sepal **22** stamen **23** ovary and style. From *Køie* 684 (B) **24–30** *Convolvulus lindbergii* **24** leaf **25** bracteole **26** outer sepal **27** middle sepal **28** inner sepal **29** stamen **30** ovary and style. From *Lindberg* 409 (W) **31–36** *Convolvulus leptocladus* **31** leaves **32** bract **33** outer sepal **34** inner sepal **35** stamen **36** ovary and style **31–32** from *Rechinger* 3389 (W) **33–36** from *Scharif* 832E (W) **37–40** *Convolvulus peninsularis* **37** portion of inflorescence **38** bract **39** outer sepal **40** inner sepal. From *Whitcombe* 807 (E).

Figures 2b and 16, t. 37–40

##### Diagnosis.

Sepalis ovatis acuminatis 2.5–4 mm longis *Convolvulus sarmentosum* et *Convolvulus hildebrandtii* tangit sed ramis lignosis, sepalis pubescentibus pilis patentibus, affine *Convolvulus leptocladi* Boissier sed sepalis ovatis dignoscenda.

##### Type.

OMAN, Breik Qotait, Mussandan Peninsula, 30 March 1980, *R. Whitcombe* 807 (holotype E00507115!).

##### Description.

Small unarmed undershrub c. 15 cm high, stems rigid, minutely adpressed pubscent. Leaves almost completely absent, probably lanceolate, certainly acute, entire, thinly pubescent. Inflorescence elongate, formed of pedunculate dichasial cymes arising in the axils of bracts at right angles to the stem; bracts 2–10 × 0.5–2 mm, linear to narrowly oblong, acute, entire, cuneate at base, thinly appressed pubescent; peduncles 1.5–3 cm long, straight, rigid, appressed pubescent; bracteoles 1–2 × 0.5 mm, linear, pubescent; pedicels c. 5–15 mm; flowers almost always solitary; sepals 3.5–4 × 1.5 mm, ovate, acuminate, pubescent with spreading hairs, inner sepals similar but somewhat scarious-margined; corolla c. 10 mm long, of unknown colour and shape, midpetaline bands pubescent; filaments c. 1 mm; anthers c. 1.5 mm; ovary c. 1.25 mm, acuminate, glabrous; style glabrous, divided c. 2 mm above base, stigmas c. 2 mm.

##### Distribution.

Known only from the type collection made on the Musandam Peninsula in Oman.

##### Notes.

This species is very similar morphogically to *Convolvulus leptocladus* and is similarly nearly aphyllous but differs in the ovate-acuminate sepals which are pubescent with spreading hairs. It is more obviously woody than *Convolvulus hildebrandtii* with stouter peduncles, recalling those of *Convolvulus eremophilus*, and the inflorescence is of well-developed dichasial cymes. The sepals are similar in shape to those of *Convolvulus hildebrandtii* but slightly larger. There is abundant spreading pubescence on all parts of the inflorescence, not just on the sepals.

This species is only known from a single collection. It may be rare and threatened or simply overlooked but for the time being should be classified as Data Deficient (DD) within IUCN (2012) guidelines. The epithet “*peninsularis*” refers to the Mussandan Peninsula in Oman where the type collection was made. No information is known about its precise habitat.

#### 119. Convolvulus leptocladus

Taxon classificationPlantaeSolanalesConvolvulaceae

Boiss., Diagn. Pl. Orient. 7: 25. 1846. (Boissier 1846: 25).

Figure 16, t. 31–36

##### Type.

IRAN, *Aucher-Eloy* 4942 (holotype G; isotypes K!, LE!, P!, W!).

##### Description.

Much-branched fastigiate undershrub to c. 40 cm, the branches usually arising at right angles to the main stem, adpressed pubescent, hairs resembling cystoliths. Leaves commonly absent, sessile, 1. 5–2.5 cm, linear to linear oblanceolate, acute, entire, attenuate at base, subglabrous or with few adpressed hairs. Flowers 2–3 in very lax axillary dichasial cymes borne on rigid branches, somewhat spinescent after flowers have fallen; peduncles 1.5–2 cm; bracteoles 1 mm, oblong-elliptic, minute; pedicels 2–8 mm, very fine; sepals 3–4 × 2 mm, obovate, shortly mucronate, uniformly pale green, adpressed pubescent, inner sepals slightly longer and broader; corolla 11–14 mm long, white, weakly lobed, midpetaline bands pilose; ovary glabrous; style glabrous, divided c. 3 mm above base, stigmas 4 mm. Capsule glabrous; seeds brown, glabrous, smooth. [Sa’ad 1967: 99; Nowroozi 2002: 45 (plate), 102 (map)]

##### Distribution.

Endemic to southern Iran (*Léonard* 5921, *Grey-Wilson & Hewer* 127, *Rechinger et al*. 3389, *Scharif* 832E, *Fasy* 337, *Foroughi* 10671)

##### Notes.

The very short pubescent sepals are a good character as are the very slender peduncles arising at or near 90°. There seems to be some variation in inflorescence branching, sometimes clearly dichasial (*Grey-Wilson & Hewer* 127), sometimes almost racemose (*Léonard* 5921).

### 

The following species (species 120–126) form a complex of mostly ill-defined taxa. Although frequently misidentified, *Convolvulus erinaceus* is the best defined, usually easily identified by its distinctive morphology, small deeply lobed corolla and short rounded sepals. *Convolvulus hamadae* has the same appressed sericeous indumentum but with acute sepals and a larger unlobed corolla. The others have varyingly pubescent to tomentose stems and differ in details of bract shape, ovary indumentum, sepal form and habit. Further collection and study may demonstrate several to be conspecific or hybrids.

#### 120. Convolvulus erinaceus

Taxon classificationPlantaeSolanalesConvolvulaceae

Ledeb., in Eichwald, Pl. Nov. Cauc. Casp. 11. 1831. (Eichwald 1831:11).

Figure 16, t. 1–7

Convolvulus excelsus R.R.Mill, Edinburgh J. Bot. 70: 368. 2013. (Mill 2013: 368). Type. SAUDI ARABIA, *Collenette* 6385 (holotype E00699569!; isotype K!).

##### Type.

AZERBAIJAN (?), “Caspian Sea”, *Eichwald* 474 (lectotype LE!, sheet labelled 474 and with Sa’ad’s annotation, designated here; isolectotypes HAL ?, LE!).

##### Description.

Much-branched undershrub with somewhat zigzag, rigid branches forming an intricate ball-like plant to 1(-3) m in height, the lower part sometimes with a distinct trunk up to 1.5 cm in diameter; young stems sericeous, branching at 80–90°. Basal leaves 10–30 × 1 mm, stem leaves usually few, sessile, 6–7 (-20) × 0.5–1 mm, linear, pubescent. Flowers usually solitary (rarely paired) borne on thin, rigid peduncles 4–15 (-20 )mm long; bracteoles 1 mm, triangular, scale-like, puberulent; pedicels 1–5 mm, puberulent, often recurved; outer sepals 3–4 × 2.5 mm, elliptic-obovate, rounded to emarginate, sericeous-pubescent; inner sepals narrower, 1–1.5 mm wide; corolla 0.6–0.9 cm long, deeply lobed to about quarter of its length, white or pink, midpetaline bands narrowly triangular, pubescent, terminating in a point; ovary sericeous; style glabrous, very short, divided c. 2 mm above base, stigmas c. 1.5 mm; capsule pubescent or glabrous, 1-seeded; seeds pubescent. [Sa’ad 1967: 95 p. p. excl. *Convolvulus hamadae* and Convolvulus erinaceus var. kermanensis; Petrov 1935: 137 (plate); Nowroozi 2002: 32 (plate), 101 (map); Collenette 1999: 228 as *Convolvulus excelsus* (photo); Grigoriev 1953: 11 (plate)]

##### Distribution.

Russia: Dagestan (*Teimurov* s.n. [20/6/2011]), “Siberia” (*Turczaninov*); Azerbaijan (*Shipzchinsky* s.n. [14/7/1925]); Uzbekistan (*Neustreuva-Knorring* 3769, *Rodin & Arkadyev* s.n. [8/5/1948]); Turkmenistan (*Androsov* s.n. [10/7/1932], *Babrov* 535, *Granitov* in Herb. Fl. As. Med.442; Kirgizstan (*Minkwitz* 376); Tadjistan (*Nikitin* 2780); Kazakhstan (*Dubrayansky* 852, 856, *Afannasiev* 3767, *Berg* s.n. [28/6/1900], *Spiridonow* s.n. [1914], *Rodin et al.* 468); Northern Iran (*Reino Alava & Iranshahr* 23405, *Furse* 7614); Afghanistan (*Aitchison* 701, *Koie* 2225, *Neubauer* 4206), Pakistan (?), Saudi Arabia (*Collenette* 6385). A plant of sandy and stony desert.

##### Notes.

Distinguished by the sericeous stem, short obtuse sepals, small, lobed corolla and tendency to have short rigid peduncles giving the plant a characteristic intricate habit.

The recently described *Convolvulus excelsus* from Saudi Arabia is distinguished principally by its tall habit and disjunct distribution. However neither its height nor its disjunction is as distinctive as Mill (2013) suggests, there being records from scattered locations in many countries (see above) and images and descriptions of specimens at least 1 m high.

#### 121. Convolvulus hamadae

Taxon classificationPlantaeSolanalesConvolvulaceae

(Vved.) Petrov, Byull. Moskovsk. Obshch. Isp. Prir., Otd. Biol., n.ser., 44: 132. 1935. (Petrov 1935: 132).

Convolvulus subsericeus subsp. hamadae Vved., Byull. Sredne-Aziatsk. Gosud. Univ. 15 (Suppl.): 32. 1927. (Popov and Vvedensky 1927: 32). Type. KAZAKHSTAN, Kara-tau, *Popov* 5/6/1926 (holotype TAK; isotypes B, BM!, E!, K!, LE!, P!, W!.)

##### Type.

Based on Convolvulus subsericeus subsp. hamadae Vved.

##### Description.

Erect undershrub, intricately branched from the base to at least 40 cm; stems, thinly sericeous. Leaves 1–5 × 0.2 cm, linear-oblong, acute, base tapering into a long pseudopetiole, thinly adpressed pubescent and appearing greenish. Flowers 1 (-2) at the apex of rigid, straight, relatively slender woody peduncles 0.8–1.5 cm long; bracteoles minute, linear, c. 1 mm, pedicels 0.5–1 mm, bent at 90° to peduncle; sepals (3-) 4 (-5) × 1.5 mm, narrowly oblong, acute, densely pubescent; corolla (8-)10 mm, white, unlobed, broadly infundibuliform, midpetaline bands pilose, terminating in a tooth; ovary pilose. Capsule pilose, seeds pubescent.

##### Distribution.

Kazakhstan (*Drobov* s.n. [3/8/1996], *Krasheninnikov* 158); Kyrgyzstan (*Knorring* 115, *Drobov* 246); Uzbekistan (*Tishchenko* 98, *Muravliansky* 1932, *Knorring* 60); Tajikistan (*Nikitin* 167, s.n. [19/6/1936], *Knorring* 129); Turkmenistan (*Litvinow* 1647, *Sintenis* 1285); Afghanistan (*Aitchison* 731); Iran (*Pabot* DK 518). Most common in Uzbekistan.

##### Notes.

The indumentum and corolla size suggests this lies between *Convolvulus erinaceus* and *Convolvulus subsericeus* or more probably *Convolvulus eremophilus*, given the distribution of the three species. It has a laxer, less rigid appearance than *Convolvulus erinaceus* with branches less divaricate, the corolla is longer and unlobed and the sepals are acute, not obtuse. From *Convolvulus subsericeus* it is distinguished by the shorter, pungent branches, flowers generally solitary and a little smaller, the sepals acute, not acuminate. From *Convolvulus eremophilus* it is distinguished by the sparse, adpressed indumentum of the stems and shorter corolla. A few specimens are completely glabrous, such as *Botchantzev* 468 from Uzbekistan, but seem to fit here best.

Sa’ad treated this species as a synonym of *Convolvulus erinaceus* but the sepals and corolla are quite different.

#### 122. Convolvulus subsericeus

Taxon classificationPlantaeSolanalesConvolvulaceae

Schrenk, Enum. Pl. Nov. [F.E.L. Fischer & C.A. Meyer] 1: 19. 1841. (Fischer and Meyer 1841: 19).

Convolvulus cantabrica subsp. subsericeus (Schrenk) Choisy, Prodr. [A.P. de Candolle] 9: 402. 1845. (Choisy 1845: 402). Type. Based on *Convolvulus subsericeus* Schrenk
Convolvulus neglectus Palib., Russk. Bot. Zhurn. 1913(3): 24. 1915. (Palibine 1915: 24). Type. “TURKESTAN”, various collections cited (LE).

##### Type.

KAZAKHSTAN, Balkash, *Schrenk* s.n. (lectotype LE!, sheet annotated “Typus” by Grigoriev, designated here; isolectotypes BM001035795!, K!, LE!, OXF!, W!).

##### Description.

Branched (often divaricately) undershrub to at least 50 cm, stems sericeous to adpressed puberulent, somewhat zigzag, branches arising at an acute angle. Basal leaves unknown, stem leaves sessile, 1–3 × 0.1–0.3 cm, linear-lanceolate, acute, entire, base cuneate, sericeous, Flowers up to 3 in pedunculate, cymes but commonly reduced to solitary flowers; peduncles 2.5–6 cm, characteristically long and slender, often bent at transition to pedicel; bracteoles 2 × 0.5 cm, deltoid-lanceolate, caducous; pedicels very short, 1–2 mm; sepals 5–7 × 2 mm, narrowly ovate, shortly acuminate, pubescent; corolla 1.1–1.5 cm, white, shallowly lobed, the midpetaline bands, pilose, terminating in teeth; ovary pilose; style glabrous, divided c. 5 mm above base, stigmas 2 mm. capsule pilose at apex; seeds pubescent.

##### Distribution.

Kazakhstan (*Karolin & Kiriloff* 1720, *Sokolov* s.n. [11/6/1908], *Schipczinsky* 305, *Dubiansky & Basilevskaja* s.n. [9/8/1927], *Chaffanjon* 660). Apparently rare and limited to sandy desert in the area around Lake Balkash.

##### Notes.

*Convolvulus subsericeus* is sometimes confused with *Convolvulus divaricatus* from which it is distinguished by the subsericeous stems, the narrow, linear-lanceolate stem leaves and the long slender branches branches arising at an acute angle.

#### 123. Convolvulus divaricatus

Taxon classificationPlantaeSolanalesConvolvulaceae

Regel & Schmalh., Trudy Imp. S.-Petersburgsk. Bot. Sada 6: 338. 1879. (Regel 1879: 338).

Figure 17, t. 1–7

Convolvulus deserticulus Podlech, Mitt. Bot. Staatssammel. München 17: 482. 1981. (Podlech 1981: 482). Type. AFGHANISTAN, Kandahar, *Podlech* 30744 (holotype M; isotype W!).
Convolvulus afanassievii Luferov, Kumarovia 1: 59. 1999. (Luferov 1999: 59). Type. UZBEKISTAN, Kzyl-Kum, *Afanassiev* 238 (holotype LE!; isotypes LE!).

##### Type.

UZBEKISTAN, Chiwa (Khiva), *Koralkov* s.n. (holotype LE!).

##### Description.

Perennial undershrub from a woody rootstock forming a small bush; stems numerous to 50 cm, rigid and woody below, herbaceous above, slightly divaricate, densely grey-pilose. Basal leaves not seen; stem leaves sessile, 0.3–1.5 × 0.3–1.5 cm, very variable in size from plant to plant, oblanceolate, lanceolate, ovate or suborbicular, acute, entire to somewhat undulate, basally cuneate to rounded, pilose. Flowers 1–3 (- 5) in axillary dichasial cymes but often solitary; peduncle 0.5–6 cm, somewhat terete to rigid; bracteoles 3 mm, filiform; pedicels 2–10 mm; outer sepals 5–6 × 2.5 mm, lanceolate, ovate or elliptic, acuminate or caudate, densely lanate; inner sepals broader (c. 3 mm), glabrous; corolla 1.1–1.3 (-1.5) cm long, cream or pinkish, unlobed, midpetaline bands darker, pilose, terminating in a tooth; ovary glabrous or with a few apical hairs; style glabrous, divided c. 2 mm above base, stigmas c. 2 mm; capsule glabrous, 1-seeded (?always); seeds hirsute. [Sa’ad 1967: 89]

**Figure 17.**
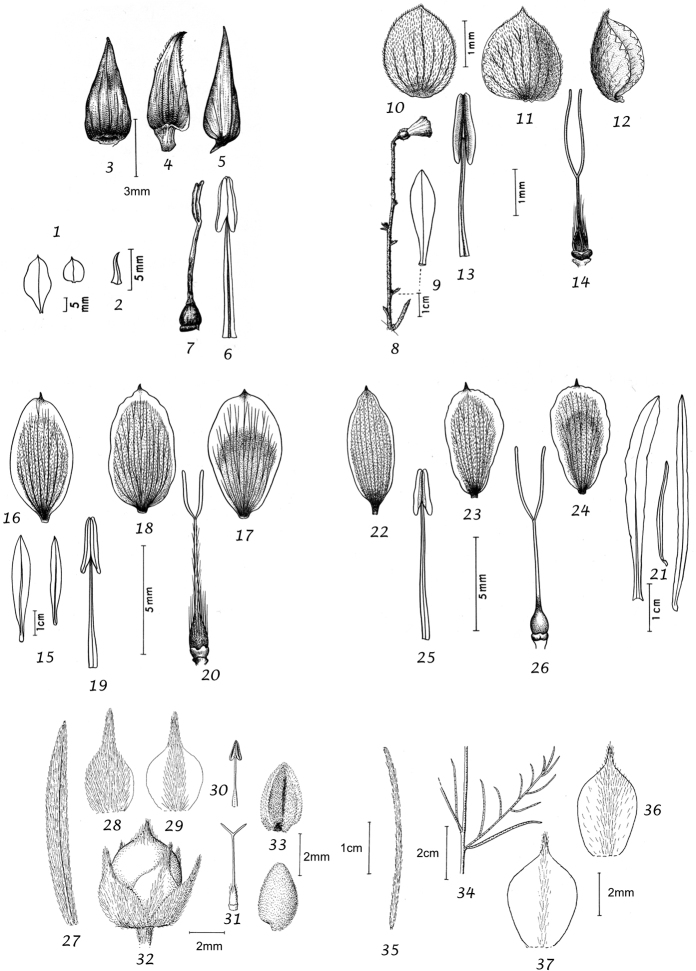
**1–7** *Convolvulus divaricatus* **1** leaves **2** bracteole **3** outer sepal **4** middle sepal **5** inner sepal **6** stamen **7** ovary and style. From *Drabov* 287 (B) **8–14** *Convolvulus gracillimus* **8** flowering branch **9** leaf **10** outer sepal **11** middle sepal **12** inner sepal **13** stamen **14** ovary and style. From *Rechinge* r 16080 (W) **15–20** *Convolvulus sarothrocladus* **15** leaves **16** outer sepal **17** middle sepal **18** inner sepal **19** stamen **20** ovary and style **15** from *sin coll*. (JE) **16–20** from *Haussknech* t s.n. (W) **21–26** Convolvulus pseudocantabrica subsp. askabadensis **21** leaves **22** outer sepal **23** middle sepal **24** inner sepal **25** stamen **26** ovary and style. From *Gauba* 1623 (B) **27–33** *Convolvulus ammannii* **27** leaf **28** outer sepal **29** inner sepal **30** stamen **31** ovary and style **32** sepals and capsule **33** seeds. From *Hsia* 20/7/1931(K) **34–37** *Convolvulus xanthopotamicus* **34** shoot showing woody stem and branching **35** leaf **36** outer sepal **37** inner sepal. From *Purdom* s.n. (K).

##### Distribution.

Uzbekistan/Kazakhstan: Kzyl-Kum (*Knorring* 155, *Rodin & Arkadyev* 213, *Rodin et al.* 634); Turkmenistan (*Dubiansky* s.n. [9/5/1925], *Petrov* s.n. [10/5/1930], *Litwinow* 1425); Tajikistan (*Korshinsky* 7298); Afghanistan (*Hewer* 1060, *Furse* 5574, *Rechinger* 34194b). Common in sandy deserts.

##### Notes.

Distinctive because of the pilose indumentum with spreading hairs combined with the lanceolate to ovate stem leaves but very close to *Convolvulus subsericeus* and *Convolvulus eremophilus*. From the latter it is best distinguish by the less rigid, less flexuouse branches arising at around 60° from the stem as well as the broader leaves.

Specimens intermediate between *Convolvulus divaricatus* and Convolvulus eremophilus exist (Grigoriev 1953: 19) and have been called *Convolvulus michelsonii* (V. Petrov) V. Petrov ex Grigoriev (1953: 19) or Convolvulus korolkowii var. michelsonii V.Petrov (1935:136), the type being *Michelson* s.n. [29/5/1914] (holotype LE) from the Kyzyl-Kum desert in Uzbekistan. However, their status is uncertain and they may be hybrids. Examples include *Litwinow* 873a (LE) from Turkmemistan.

#### 124. Convolvulus tujuntauensis

Taxon classificationPlantaeSolanalesConvolvulaceae

Kinzik., Fl. Tadzhikskoi SSR 7: 524. 1984. (Kinzikaeva 1984: 524).

##### Type.

TAJIKISTAN, *Kinzikaeva, Soskov & Possadskaja* s.n. [29/8/1960] (holotype LE!; isotype LE!).

##### Description.

Small grey-pilose undershrub similar in general facies to *Convolvulus divaricatus* but stems more rigidly erect and only reaching 30 cm, leaves 1–3 × 0.2–0.5 cm, linear to linear-oblanceolate. Flowers subsessile or shortly pedunculate with peduncles to 1.2 cm, bracteoles persistent, pedicels very short, < 1 mm, sepals c. 6 mm long, narrowly ovate, acuminate, corolla somewhat larger, 1.2–1.6 mm long. Ovary, style and capsule all pilose. Also close to *Convolvulus subsericeus* but much more hirsute.

##### Distribution.

Tajikistan, Tujun-tau, apparently only known from the type.

##### Notes.

This species needs revision. It may or may not prove to be distinct from *Convolvulus divaricatus*. It should be noted that some details in the protologue are incorrect, particularly as to the size and shape of the leaves.

#### 125. Convolvulus eremophilus

Taxon classificationPlantaeSolanalesConvolvulaceae

Boiss. & Buhse, Nouv. Mém. Soc. Imp. Naturalises Moscou 12: 148. 1860. (Boissier and Buhse 1860: 148).

Figure 16, t. 8–16

Convolvulus erinaceus C.A.Mey., Verz. Pfl. Casp. Meer 102. 1831, nom. illeg., non *Convolvulus erinaceus* Ledeb. (1831). (Meyer 1831: 102). Type. AZERBAIJAN, between Baku and Sallian, near Eszek Caravanserai, *C.A. Meyer* s.n. (LE, not seen).
Convolvulus korolkowii Regel & Schmahl., Trudy Imp. S.-Petersburgsk. Bot. Sada 6: 338. 1879. (Regel 1879: 338). Type. UZBEKISTAN, Chiwa (Khiva), *Korolkow* s.n. (holotype LE!).
Convolvulus dorycnium var. turcomanicu s Kuntze, Trudy Imp. S.-Peterburgsk. Bot. Sada 10: 221. 1887. (Kuntze 1887: 221). Type. TURKMENISTAN, 5/1886, *O. Kuntze* s.n. (holotype LE!).
Convolvulus turcomanicus (Kuntze) Petrov, Byull. Moskovsk. Obshch. Isp. Prir., Otd. Biol., n.ser., 44: 136. 1935. (Petrov 1935: 136). Type. Based on Convolvulus dorycnium var. turcomanicu s Kuntze
Convolvulus spinosus var. kermanensis Bornm., Beih. Bot. Centralbl. 61: 82. 1941. (Bornmüller 1941: 82). Type. IRAN, Kerman, *Bornmüller* 3886 (holotype B; isotypes K!, P!, W!).
Convolvulus spinosus var. subinermis Parsa, Kew Bull. 3: 213. 1948. (Parsa 1948: 213). Type. IRAN, Kerman, *Bornmüller* 3886 (holotype K!; isotypes B, P!, W!).
Convolvulus erinaceus var. kermanensis (Bornm.) Sa’ad, Meded. Bot. Mus. Herb. Rijks Univ. Utrecht 281: 96. 1967. (Sa’ad 1967: 96). Type. Based on Convolvulus spinosus var. kermanensis Bornm.
Convolvulus longipedunculatus Podlech, Mitt. Bot. Staatssamml. München 17: 483. 1981. (Podlech 1981: 483). Type. AFGHANISTAN, Ghorat, *Podlech* 21799 (holotype M!).

##### Type.

IRAN, Kashan, *Buhse* 1463 (holotype G; isotypes K!, P!).

##### Description.

Undershrub from a thickened woody rootstock to 60 cm with rigid (but not spiny) branches spreading widely at almost a right angle and forming an entwined mass; stems and leaves pubescent with spreading hairs. Leaves sessile, 0.5–5.5 × 0.1–0.9 cm, linear to oblong, acute, margin entire to undulate, pubescent, merging upwards into the bracts. Flowers pedunculate, solitary or in 2–3-flowered cymes; bracts 3–20 × 0.5–2.5 mm, suborbicular to broadly ovate; peduncles 0.8–5.5 cm, straight, rigid, usually arising at 90° to the stem; bracteoles 2–3 mm, filiform; pedicels 0–1 mm, often at an angle from the peduncle; outer sepals 4–5 × 2 mm, ovate, acute with mucronate apex, pilose, pubescent or, rarely, glabrous on the abaxial surface, sometimes glabrescent in fruit; inner sepals similar but less hairy and commonly convex; corolla 1.1–1.8 cm, white or purplish-flushed, somewhat undulate but not lobed, midpetaline bands pubescent; ovary glabrous to hirsute, style glabrous; style divided 5–6 mm above base; capsule glabrous or with a few apical hairs, seeds hirsute. [Petrov 1935: 138 (plate under *Convolvulus turcomanicus*); Sa’ad 1967: 94; Nowroozi 2002: 43 (plate), 101 (map); Breckle and Rafiqpoor 2010: 415 (photo as *Convolvulus korolkowii*)]

##### Distribution.

Turkmenistan (*Litwinov* 1641, 1654; *Sintensis* 330, 487, *Smirnova* 13436, *Androsov & Bubyir* s.n. [4/6/1912]); Uzbekistan (*Rodin & Arkadyev* 243); Kazakhstan (*Kudishchov* s.n. [14/7/1928], *Rodin et al.* 476, *Popetsky* s.n. [26/3/1932]), Azerbaijan, Afghanistan (*Hedge & Wendelbo* 3569, 3839, 8407, *Neubauer* 4253, *Rechinger* 34194); Iran (*Schmidt* 6091, 6275, *Bunge* s.n. [8/1/1858], *Renz & Iranshahr* s.n. [8/7/1974], *Rechinger* 1285, 1313, 46317, 51477, 53682, 55800, 57447). In sandy deserts; abundant in Turkmenistan and parts of northern Iran and Afghanistan.

##### Notes.

We are proposing a broad concept of this ill-defined species. It is characterised by the presence of spreading hairs at least on the lower part of the stem, acute to mucronate sepals and straight rigid peduncles arising at 80–90° to the branch. The types of *Convolvulus eremophilus*, *Convolvulus korolkowii* and *Convolvulus turcomanicus* have a glabrous ovary while that of *Convolvulus longipedunculatus* is hirsute. However this is hardly discrete as specimens with ovaries having a few hairs (*Hedge & Wendelbo* 3569, *Rechinger* 46317, 51447, *Schmidt* 6275) occur as well as plants with a densely hairy ovary (*Rechinger* 55800, *Neubauer* 4253, *Schmidt* 6091). Sa’ad separated what she considered *Convolvulus eremophilus* (*Aellen & Scharif* 5318E, *Moussouri & Tehrani* 30693E mostly from Kashan) from *Convolvulus korolkowii* by its suborbicular, not linear bracts. This is a difficult character as bracts are often not present and in any case there are specimens whose bracts are elongate in form, such as *Hedge & Wendelbo* 3839 identified as *Convolvulus eremophilus* by Sa’ad. *Convolvulus turcomanicus* was separated by its glabrous apiculate sepals. Apiculate sepals are common even on specimens with pubescent sepals while glabrous and pubescent sepals are sometimes found on the same plant. It seems glabrescence is common as the plant ages. There is also considerable variation in corolla size but most corollas are about 12–15 mm long. Despite these differences all specimens have a common facies and the different variations do not correlate well with each other or show any very distinct geographical patterning. Many specimens are in any case difficult to evaluate as bracts, corollas and capsules are often wanting.

#### 126. Convolvulus lindbergii

Taxon classificationPlantaeSolanalesConvolvulaceae

Sa’ad, Meded. Bot. Mus. Herb. Rijks Univ. Utrecht 281: 99. 1967. (Sa’ad 1967: 99).

Figure 16, t. 24–30

##### Type.

AFGHANISTAN, *Lindberg* 409 (holotype W!).

##### Description.

Much-branched undershrub to 30 cm, stem and all vegetative parts grey-sericeous, branches rigid and with few leaves, ascending at about 60°. Leaves sessile, 6–20 × 1–5 mm, linear to narrowly oblong, obtuse, entire, narrowed at both ends. Flowers 1–3, arising in the axils of small bracts on a rigid peduncle; bracts 2 × 1 mm, triangular; peduncles 0.8–2.5 cm; bracteoles c. 1 mm; pedicels 0–1.5 mm, sepals 3–3.5 × 2 mm, oblong-elliptic, densely sericeous, outer rounded, inner mucronulate; corolla 0.9–1 cm long, white, midpetaline bands sericeous, weakly lobed; ovary globose, pubescent (or glabrous in *Rechinger* 35336), style glabrous, divided c. 4 mm above base, stigmas 1.5 mm. Capsule and seeds unknown.

##### Distribution.

Afghanistan (*Rechinger* 35336, *Gibbons* 227, *Breckle* 4828).

##### Notes.

This poorly known species is characterised by the grey sericeous stems and leaves, small sericeous mucronulate calyx and short corolla. It may be only a form of *Convolvulus eremophilus*.

#### 127. Convolvulus sarothrocladus

Taxon classificationPlantaeSolanalesConvolvulaceae

Boiss. & Hausskn., Fl. Orient. [Boissier] 4: 92. 875. (Boissier 1875b: 92).

Figure 17, t. 15–20

##### Type.

IRAQ, between Kirkuk and Sulaymaniyah, *Haussknecht* 653 (lectotype G, designated by Sa’ad 1967: 105).

##### Description.

Undershrub with fastigiate branches to 40 cm, stems appressed pubescent. Leaves sessile, the basal leaves 2.5–6 × 0.5–3 cm, oblanceolate, acute to obtuse, attenuate at the base, appressed pubescent; stem leaves linear 1.5–3.5 × 0.2–0.4 cm. Flowers axillary, solitary or paired forming a lax, sparsely branched inflorescence, which is commonly dichasial in structure apically; peduncles 0.5–2 cm long, slender, bracteoles minute; pedicels 2–6 mm; sepals 6–7 × 3–4 mm, elliptic to obovate, mucronate, the margins scarious, the inner sepals narrower; corolla 12 mm long, white, midpetaline bands pubescent; ovary comose; style pilose, divided 4 mm above base, stigmas 1–2 mm. Capsule not seen. [Sa’ad 1967: 105]

##### Distribution.

Iraq (*Helbaek* 1726, *Haussknecht* s.n. [6/1867]); Iran (*Koelz* 15840).

##### Notes.

This species is distinguished by the relatively large, glabrous sepals and narrow, sparingly branched inflorescence which is clearly cymose in structure apically. It seems close to Convolvulus pseudocantabrica subsp. askabadensis but the ovary is hirsute and the corolla smaller.

#### 128. Convolvulus kurdistanicus

Taxon classificationPlantaeSolanalesConvolvulaceae

Mozaff., Pakistan J. Bot. 34: 395, f. 2. 2002. (Mozaffarian 2002: 395).

##### Type.

IRAN, Kermanshah, *Mozaffarian* 79475 (holotype TARI).

##### Description.

Perennial undershrub with woody taproot, from which arise many sericeous stems to 50 cm. Basal leaves unknown; stem leaves sessile, 20–40 cm long, linear-filiform. Flowers 3–5 in axillary and terminal dichasial cymes; bracteoles linear, acute; sepals 8 mm long (fide description) or 4–4.5 mm (fide illustration), ovate, mucronate, glabrous; corolla c. 1.8 cm long, white, midpetaline bands pubescent; ovary glabrous; style glabrous, much longer than stigmas (fide illustration). Capsule glabrous, 1-seeded.

##### Distribution.

Iran; Iraq (*Rawi* 5720).

##### Notes.

We have not seen the type material. *Rawi* 5720 is annotated in the Kew herbarium as “Convolvulus leptocladus subsp. glabrisepalus Kandemir” and seems to fit perfectly with *Convolvulus kurdistanicus* as long as the sepals of *Convolvulus kurdistanicus* are the more probable 4.5 mm long (as illustrated), rather than 8 mm long as described. *Convolvulus kurdistanicus* is also extremely close to *Convolvulus koieanus* differing only in the glabrous ovary. As *Convolvulus kurdistanicus* and *Convolvulus koieanus* grow in neighbouring provinces of Iran, it seems quite possible that they will prove to beconspecific.

#### 129. Convolvulus koieanus

Taxon classificationPlantaeSolanalesConvolvulaceae

Bornm., Dan. Sci. Invest. Iran 4: 37. 1945. (Köie 1945: 37).

Figure 16, t. 17–23

##### Type.

IRAN, Luristan, Chah-Bazan, *Köie* 1301 (lectotype C, designated by Sa’ad 1967: 97; isolectotype B).

##### Description.

Undershrub with woody taproot, from which arise many rigid branched stems to c 40 cm, stems minutely adpressed pilose, the hairs resembling cystoliths. Leaves sessile, 10–30 × 0.5 mm, filiform, acute, adpressed pubescent. Flowers 1–3 in long-pedunculate, axillary and terminal dichasial cymes; peduncles up to 20 cm long, straight, slender and not very rigid; bracteoles 1–2 × 0.25 mm, filiform; sepals 5–6 × 4 mm, obovate-elliptic, apiculate, glabrous; corolla1.4–1.5, colour unknown, midpetaline bands pubescent; ovary comose; style divided 2 mm above base; stigmas 3 mm. Capsule and seeds not known. [Sa’ad 1967: 97, Nowroozi 2002: 53 (plate), 102 (map)]

##### Distribution.

Iran (*Köie* 1302, 684).

##### Notes.

Appears to be close to *Convolvulus leptocladus* but is distinguished by its filiform leaves, pubescent ovary and longer, oblong sepals.

#### 130. Convolvulus gracillimus

Taxon classificationPlantaeSolanalesConvolvulaceae

Rech.f., Anz. Österr. Akad. Wiss., Math.-Naturwiss. Kl 92: 274. 1955. (Rechinger 1955: 274).

Figure 17, t. 8–14

##### Type.

IRAN, Tehran, *Koelz* 16080 (holotype W!; isotypes E!, US).

##### Description.

Slender, sericeous, rigid undershrub of grey appearance to c. 30 cm with numerous erect stems and very rigid branches arising at c. 60°. Leaves sessile, 0.8–2.8 × 0.2–0.3 cm, linear to oblanceolate, acute, entire, decurrent at the base. Inflorescence of solitary pedunculate flowers, becoming somewhat dichasial cymose apically; bracts oblong, caducous; peduncles 5–20 mm, slender; bracteoles minute, scale-like; pedicels 3–7 mm, curved; sepals 2 × 2 mm, suborbicular to broadly elliptic, obtuse, the outer ones pubescent, the inner glabrous, membranous, mucronate; corolla 0.6 cm long, white, midpetaline bands pilose; ovary pilose, style pilose, divided 1 mm above base, stigmas 2 mm. Capsule and seeds not seen. [Sa’ad 1967: 109; Rechinger 1963: 25 (plate)]

##### Distribution.

Iran. Only known from the type collection.

##### Notes.

The tiny sepals and corolla and the very rigid grey stems are characteristic.

#### 131. Convolvulus pseudocantabrica

Taxon classificationPlantaeSolanalesConvolvulaceae

Schrenk, Enum. Pl. Nov. [F.E.L. Fischer & C.A. Meyer] 1: 21. 1841. (Fischer and Meyer 1841: 21).

##### Type.

KAZAKHSTAN, Koksu River, *Schrenk* s.n. (lectotype LE!, sheet with single rootstock, designated here).

##### Description.

Perennial undershrub with tough woody rootstock from which arise several erect, woody, branched fastigiate stems to 50 cm; stems adpressed pubescent. Basal leaves sessile, 2–4 × 0.2–0.5 cm. oblong, acute, entire, attenuate to somewhat abruptly narrowed at the base, glabrous to thinly adpressed pubescent on the veins and lower surface; stem leaves similar but linear, diminishing in size upwards. Inflorescence terminal and panicle-like, formed of terminal dichasial cymes and solitary flowers at the apex of lateral branches; bracteoles 2–5 mm, filiform; pedicels 1–10 mm; sepals 5–7 × 2 mm, oblong to obovate, acute to mucronate, glabrous, the inner sepals larger, c. 3 mm wide, obovate, with a clearly demarcated wing-like margin; corolla 1.8–2.2 cm, pink, undulate, midpetaline bands pilose, terminating in a broad tooth; ovary glabrous, style glabrous, divided c. 4 mm above base, stigmas 3 mm. Capsule glabrous, 1-seeded, seeds pubescent (fide Sa’ad). Sa’ad 1967: 100; Austin and Ghazanfar 1979: 14.

##### Notes.

Two well-defined subspecies can be recognised:

#### 131a. Convolvulus pseudocantabrica subsp. pseudocantabrica

Taxon classificationPlantaeSolanalesConvolvulaceae

Convolvulus dianthoides Kar. & Kir., Bull. Soc. Imp. Naturalistes Moscou 14: 708. 1841. (Karelin and Kiriloff 1841: 708). Type. KAZAKHSTAN, Mount Tarbagatay, *Karelin & Kiriloff* 329 (holotype MW; isotypes BM!, K!, LE!, OXF!, P!, W!).
Convolvulus pseudocantabrica subsp. dianthoides (Kar. & Kir.) Vved., Byull. Sredne-Aziatsk. Gosud. Univ. 11 (Suppl.): 16. 1925. (Popov and Vvedensky (1925: 16). Type. Based on *Convolvulus dianthoides* Kar. & Kir.
Convolvulus pseudocantabrica var. dianthoides (Kar. & Kir.) Sa’ad, Meded. Bot. Mus. Herb. Rijks Univ. Utrecht 281: 104. 1967. (Sa’ad 1967: 104). Type. Based on *Convolvulus dianthoides* Kar. & Kir.

##### Distinguishing features.

Characterised by the shorter sepals, c. 5 mm long, which are obovate and abruptly mucronate.

##### Distribution.

China (fide Fang and Staples 1997: 290); Afghanistan (*Rechinger* 16476, 31103, *Grey-Wilson & Hewer* 1217, *Aitchison* 847, *Volk* 169); Pakistan (*Siddiqui & Rahman* 26848); Kyrgistan (*Nikitin* s.n. [7/7/1967], *Drobov* 2779, *Federov & Ilina* 305); Tajikistan (*Kamelin* s.n. [24/7/1962], *Bornmüller* 518B, *Kinzikayeva* 1066); Kazakhstan (*Goloskokov* 4836, *Vassinger* 20); Uzkekistan (*Minkwitz* 1289, *Borodin & Kallistow* 78).

#### 131b. Convolvulus pseudocantabrica subsp. askabadensis

Taxon classificationPlantaeSolanalesConvolvulaceae

(Bornm. & Sint.) Vved., Byull. Sredne-Aziatsk. Gosud. Univ. 11 (Suppl.): 16. 1925. (Popov and Vvedensky 1925: 16).

Figure 17, t. 21–26

Convolvulus askabadensis Bornm. & Sint., Beih. Bot. Centralbl. 20(2): 181. 1906. (Bornműller 1906: 181). Type. TURKMENISTAN, *Sintenis* 1892 (holotype B†; isotypes BM!, E!, JE, K!, L, LE!, P!, STU).

##### Type.

Based on *Convolvulus askabadensis* Bornm. & Sint.

##### Distinguishing features.

Characterised by having oblong, acuminate sepals, c. 7 mm in length. Petrov 1935: 140 (plate); Nowroozi 2002: 51 (plate as *Convolvulus pseudocantabrica*), 102 (map)

##### Distribution.

Apparently restricted to Iran and Turkmenistan: Northern Iran (*Rechinger* 1744, 53148, 55562, *Schmid* 6120, *Wendelbo & Foroughi* 12677, *Sharif* 39, *Hewer* 3823, *Furse* 2901), Turkmenistan (*Meyer* 642, *Lipsky* s.n. [25/5/1912], *Litwinow* 1646, *Sintenis* 798).

##### Notes.

Sa’ad (1967) erroneously considered *Convolvulus askabadensis*, rather than *Convolvulus dianthoides*, to be conspecific with *Convolvulus pseudocantabrica*, being possibly confused by Vvidensky’s description of a superfluous subsp. dianthoides. Both Petrov (1935) and Grigoriev (1953) treated these two taxa correctly.

### Species 132–171. Old World undershrubs with sericeous leaves

A relatively distinct group of shrubs or undershrubs or, if herbaceous, woody at base, never twining or trailing, the leaves always lacking a distinct petiole. All species are hirsute and usually sericeous, often cushion-forming and/or with spinescent branches. The ovary is hirsute except in *Convolvulus lanuginosus* and *Convolvulus carduchorum*. This group is widely distributed from the Canary Islands through the Mediterranean region and Central Asia to China and Siberia. It is particularly characteristic of Central Asia where many species are armed. Hybridisation has been demonstrated more frequently amongst species of this group than in any other group and it is suspected that *Convolvulus suendermannii* and *Convolvulus sericocephalus* are also of hybrid origin.

#### 132. Convolvulus ammannii

Taxon classificationPlantaeSolanalesConvolvulaceae

Desr., Encycl. [Lamarck et al.] 3: 549. 1792. (Desrousseaux 1792: 549).

##### Type.

RUSSIA, Siberia, *Patrin* (holotype P [Herb. Lam.]!).

##### Description.

Grey-sericeous perennial herb with thick woody taproot; stems short, decumbent or weakly ascending, more or less herbaceous, to 12 (-22) cm long. Leaves sessile, 0.7–3 × 0.15–0.5 mm, linear or linear-oblanceolate, obtuse, tapering at the base. Flowers pedunculate from the axils of leaf-like bracts or, rarely, terminal only; peduncles 0.4–2.2 cm, 1-flowered; bracteoles, 4–13 × 0.5 mm, linear; pedicels 1–6 mm, often bent or curved; outer sepals 4.5–6 × 2–2.5 mm, ovate, abruptly narrowed and drawn out to an obtuse apex, the inner sepals much broader, c. 7 × 4 mm; corolla 0.8–1.3 cm long, white or very pale pink, very obscurely lobed, midpetaline bands pilose, terminating in a blunt tooth; ovary and style pilose; style divided c. 3 mm above base, stigmas c. 3 mm long. Capsule pilose at apex only; seeds smooth, pubescent. Figure 17: 27–33.

##### Distribution.

Northern China and Manchuria (*Sino-British Qinghai Expedition (1997)* 1006, *Rock* 13233); Korea (?); Mongolia (*Potanin* s.n.. [18/8/1886], *Pipe-Wolferstein & Phillips* 37); Turkmenistan (*Schipczinsky* 341); Kazakhstan (*Kossinsky* 884, *Karamisheva et al.* 5872); Uzbekistan (*Kutscherovskaya* 426); Russia: Siberia (*Timochina & Pashchenko* 7544, *Maltzev* 3764a). Very common in southern Siberia.

#### 133. Convolvulus xanthopotamicus

Taxon classificationPlantaeSolanalesConvolvulaceae

J.R.I.Wood & R.W.Scotland sp. nov.

urn:lsid:ipni.org:names:77147662-1

Figures 2c and 17, t. 34–37.

##### Diagnosis.

Maxime affine *Convolvulus ammannii* Desr. sed floribus terminalibus solitariis vel in cymis terminalibus dispositis, sepalis corollaque longioribus, caule lignosa distincta.

##### Type.

CHINA, Shensi, Hancheng Sian, 1914, *W. Purdom* s.n. (holotype K001067037!; isotypes K!).

##### Description.

Much branched perennial undershrub with erect stems to c. 20 cm; old stems woody and rigid, weakly divaricate, up to 10 mm thick, new growth herbaceous grey-sericeous. Leaves sessile, 4–40 × 0.5 mm, linear, acute, grey-sericeous. Flowers terminal, solitary or in small dichasial cymes, the branches appressed pilose, up to 6 mm long; bracts and bracteoles not clearly differentiated, 1–10 × 0.5 mm, linear; peduncles not differentiated from the stem; pedicels 1–3 mm; outer sepals 6–7 × 2.5–3.5 mm, elliptic to obovate, caudate, densely pilose; inner sepals 5 × 3–4 mm, ovate, caudate, convex, somewhat scarious, thinly pilose; corolla 1.5–2 cm, unlobed, midpetaline bands pilose; filaments 4 mm long, glabrous, anthers 2 mm, sagittate; ovary 2 mm long, conical, comose, style pilose below, divided 3–7 mm above base, persistent; stigmas 1.75 mm; capsule 2 × 3.5 mm, pubescent, capped by the persistent style; seeds 1.5 × 1 mm, conical with a rounded base, pubescent, blackish-brown.

##### Distribution.

China: Shanxi, Hanchengsian; Henan, Loyan, Shan Xian, Sanmenxia Gorge, on left bank of Yellow River (*M.P. Petrov* s.n. [27/5/1957]).

##### Notes.

Similar to *Convolvulus ammannii* but flowers all terminal, the stem somewhat woody and slightly divaricate, the sepals and corolla both significantly longer and leaves strictly linear.

This species is only known from two collections from the Hwang Ho (Yellow River) region of north China. The Petrov collection at LE has entirely solitary terminal flowers, while in the Purdom collection (K) some branches terminate in solitary flowers while in others the flowers are arranged in terminal dichasial cymes.

This species may be rare and threatened or simply overlooked but for the time being should be classified as Data Deficient (DD) within IUCN (2012) guidelines. The epithet “*xanthopotamicus*” refers to the Yellow River or Hwang Ho to whose valley system it is restricted.

#### 134. Convolvulus grigorjevii

Taxon classificationPlantaeSolanalesConvolvulaceae

Kamelin, Bot. Zhurn. (Moscow & Leningrad) 82(7): 124. 1997. (Kamelin and Lazcov, 1997: 124).

##### Type.

KYRGYZSTAN, Jalalabad, *Pimenov et al.* s.n. [9/6/1996] (holotype LE!).

##### Description.

Low undershrub with thick woody rootstock from which arise various short stiff stems to c. 20 cm, stems densely pilose, dead branches persistent and spinescent. Leaves sessile, 12–20 × 1–1.5 mm, linear-oblanceolate, obtuse, flat, silvery sericeous. Flowers subsessile from the uppermost leaf axils, 1–4 together forming a subterminal cluster; peduncles absent; bracteoles 1–3 mm, linear; pedicels 0–2 mm, pilose; sepals 6–7 × 2.5, elliptic or obovate, narrowed to an apiculate apex, densely pilose; corolla 1.7–2 cm, pink, obscurely lobed, the lobes pubescent, midpetaline bands densely sericeous; ovary and style pilose. Capsule and seeds not seen.

##### Distribution.

Kyrgyzstan (*Ajdarova & Gorbunova* s.n. [2/7/1964]).

#### 135. Convolvulus krauseanus

Taxon classificationPlantaeSolanalesConvolvulaceae

Regel & Schmahl., Trudy Imp. S.-Peterburgsk. Bot. Sada 6: 339. 1879. (Regel 1879: 339).

##### Type.

“near Tashkent”, *Krause* s.n. (syntypes LE!, 2 sheets).

##### Description.

Densely grey canescent undershrub with thick woody base, up to 1.5 cm in diameter, from which arise numerous, erect, straight, rigid but not spinescent woody stems to 40 cm, these near leafless upwards and somewhat scape-like. Leaves mostly on lower part of stem, numerous, sessile, 5–25 × 0.5 mm, linear to very narrowly linear-oblanceolate, obtuse. Flowers subsessile, 1–5 grouped at the apex of the stem, sometimes with a single flower on the stem below the apical cluster; bracts and peduncles absent; bracteoles 1–2 mm, linear-lanceolate, obtuse; pedicels 1–2 mm; sepals 4–6 × 2.5–3 mm, broadly ovate to suborbiculate, shortly apiculate, margins scarious, densely silky-pubescent; corolla 1.3–1.5 cm long, white, unlobed but with pilose border, midpetaline bands densely pilose, terminating in teeth; ovary densely pilose; style pilose, divided c. 8 mm above base; stigmas 3 mm. Capsule pilose apically. [Petrov 1935: 139 (plate)]

##### Distribution.

Endemic to Kyrgyzstan (*Knorring & Minkwitz* s.n. [24/7/1924], *Gudenov* s.n. [19/8/1958], *Botchantsov* 78, *Kamelin et al.* 9090, *Lazkov* s.n. [4/5/2005]).

##### Notes.

Very distinct because of the dense grey-canescent indumentum, the plentiful linear leaves and the long, scape-like stem with subsessile flowers at or near the apex

The cited type locality is thought to be an error as this species has never subsequently been found near Tashkent (Uzbekistan).

#### 136. Convolvulus tragacanthoides

Taxon classificationPlantaeSolanalesConvolvulaceae

Turcz., Bull. Soc. Imp. Naturalistes Moscou 5: 201. 1832. (Turczaninow 1832: 201).

Figure 18, t. 10–15

##### Type.

MONGOLIA, Tzagan-Balgassu, *Turczaninow* s.n. (holotype KW!).

##### Description.

Low grey-sericeous undershrub with woody rootstock, often more or less cushion-forming; branches short, woody, spinescent when old. Leaves sessile, 1–2.8 × 0.1–0.3 cm, linear to narrowly linear-oblanceolate, obtuse, entire, tapering at the base. Flowers in clusters of 1–4 in the axils of the leaves towards the tips of the branches; peduncles absent; bracteoles c. 2 mm long, filiform or linear; pedicels 1–2 mm; outer sepals 4–6 × 3.5 mm, obovate, rounded to truncate and slightly fimbriate, pilose abaxially; inner sepals broader, 4–4.5 mm wide, glabrous to thinly pilose; corolla 1.5–2.5 cm long, pink, unlobed, midpetaline bands pubescent; ovary pilose; style thinly pilose, divided c. 4.5 mm above base, stigmas c. 3 mm. Capsule pubescent; seeds glabrous. [Sa’ad 1967: 77]

**Figure 18.**
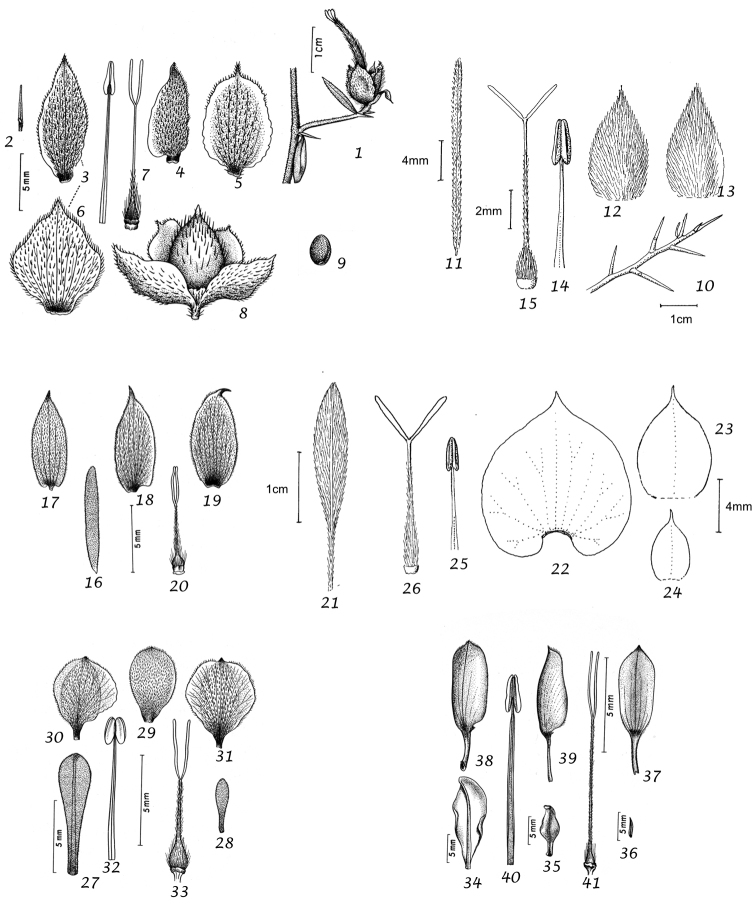
**1–9** *Convolvulus fruticosus* **1** shoot showing bract (leaf) and flower **2** bracteole **3** outer sepals (two forms) **4** middle sepal **5** inner sepal **6** stamen **7** ovary and style **8** capsule **9** seed **1** from *Borissova* 3768a (C) **2–7** from *Paulson* 303 (C) **8–9** from *Rechinger* 5502 (W) **10–15** *Convolvulus tragacanthoides* **10** shoot showing spines **11** leaf **12** outer sepal **13** inner sepal **14** stamen **15** ovary and style. From *Cowdry* 1382 (K) **16–20** *Convolvulus spinifer* **16** bract (leaf) **17** outer sepal **18** middle sepal **19** inner sepal **20** ovary and style. From *Regel* s.n. (W) **21–26** *Convolvulus gortschakovii* **21** leaf **22** outer sepal **23** middle sepal **24** inner sepal **25** stamen **26** ovary and style. From *Karelin & Kiriloff* 326 (K) **27–33** *Convolvulus spinosus* **27, 28** leaves **29** outer sepal **30** middle sepal **31** inner sepal **32** stamen **33** ovary and style. From *Griffith* 5857 (GOET) **34–41** *Convolvulus argyracanthus* **34, 35** leaves **36** bracteole **37** outer sepal **38** middle sepal **39** inner sepal **40** stamen **41** ovary and style **34–36** From *Rechinger et al.* 3228 (W) **37–41** from *Rechinger et al.* 3234 (W).

##### Distribution.

Widespread in central Asia: China (*Chung* 127, *Wilson* 288, *Farrer & Purdom* 99, *Bodinier* s.n., *Popov* 133); Mongolia (*Pipe-Wolferstan & Phillips* 37, *Petrov* s.n. [10/6/1958], *David* 2641); Kyrgyzstan (*Botchantsov* s.n. [29/7/1974], *Minkwitz* 506); Uzbekistan (*Kamelin* 36, *Ismatova* 233).

##### Notes.

*Convolvulus tragacanthoides* has the appearance of a spiny *Convolvulus ammannii*, with which it is sometimes confused, but flowers sessile and clustered.

#### 137. Convolvulus spinifer

Taxon classificationPlantaeSolanalesConvolvulaceae

Popov, Trudy Turkestansk. Gosud. Univ. 4: 56, pl. between 64–65. 1922. (Popov 1922: 56,).

Figure 18, t. 16–20

##### Type.

KAZAKHSTAN, *Popov* 546 (holotype LE!).

##### Description.

Sometimes treated as a synonym of *Convolvulus tragacanthoides*, this species appears to be distinct. It is less obviously a cushion plant having stems to at least 30 cm in height, but commonly 5–10 cm, the branches all spinescent even when young; leaves linear to oblanceolate, 0.2–0.6 cm wide; flowers subsessile in clusters of 1–5 at the apex of the stem, often with a solitary pedunculate flower borne on a rigid, woody peduncle below the apical cluster; peduncles on axillary flowers 4–13 mm; pedicels 0.5–2 mm; sepals concave, all dorsally pubescent, 5–6 mm long, acute to acuminate. [Petrov 1935: 134 (plate)]

##### Distribution.

China : Kashgar (*Popov* 241, 614); Kazakstan (*Goloskokov* s.n. [29/6/1971], *Divnogorski* s.n. [24/5/1907], *Shishkin* s.n. [17/7/1935], *Ptaschicki* 632, 642); Kyrgyzstan (*Abolin* 712, *Dessiatoff* 1864); sin loc. (*Chaffanjon* 158).

##### Notes.

*Convolvulus spinifer* appears to be somewhat intermediate between *Convolvulus krauseanus* and *Convolvulus tragacanthoides*.

#### 138. Convolvulus fruticosus

Taxon classificationPlantaeSolanalesConvolvulaceae

Pall., Reise Russ. Reich. 2: 734. 1773. (Pallas 1773: 734).

Figure 18, t. 1–9

Convolvulus fruticans Pall., Reise Russ. Reich. 2: 474. 1773, lapsus [spelling mistake] for *Convolvulus fruticosus* Pall. (Pallas 1773: 74).
Convolvulus spinosus Desr., Encycl. [Lamarck et al.] 3: 548. 1792, nom. illeg., non *Convolvulus spinosus* Burm.f. (1768). (Desrousseaux 1792: 548). Type. RUSSIA, Siberia, Irtib River, *Patrin* s.n. (holotype P).
Convolvulus eichwaldii Boiss., Diagn. Pl. Orient. 7: 27. 1846. (Boissier 1846: 27). Type. TAJIKISTAN (?). Tjuk-Karagan, *Eichwald* s.n. (whereabouts unknown).
Convolvulus frutescens Pall. ex Ledeb., Fl. Ross. (Pallas) 3: 88 (1847), lapsus [spelling mistake] for *Convolvulus fruticosus* Pall. (Ledebour 1847: 88)
Convolvulus brevispinus Jaub. & Spach, Ill. Pl. Orient. 4: 106. 1852. (Jaubert and Spach 1852: 106). Type. IRAN, Shiraz, *Aucher-Eloy* 4937 (holotype P).

##### Type.

RUSSIA, Irtysh, *Pallas* s.n. (lectotype BM 0010145880, designated here, isolectotypes BM 0010145879, BM 0010145881, BM 0010145882, LE!, W!).

##### Description.

Intricately branched spiny shrub to 50 cm, stems adpressed pubescent. Leaves sessile, 1–3.5 × 0.3–0.6 cm, oblong to narrowly oblong-elliptic, acute, margin entire, base cuneate, adpressed-pubescent to sericeous; cauline spines abundant on young shoots. Flowers borne on short spinescent axillary peduncles, usually solitary, sometimes up to 3 in a diachasial cyme; peduncles 12 mm, spine-like; bracteoles 9–11 × 1–2 mm, linear to linear-elliptic; pedicels 2 mm, recurved; sepals very lax, outer sepals 10–11 × 3–6 mm, elliptic or rhomboid, acute to mucronate, densely pubescent; inner sepals much shorter, 7–8 mm long; corolla 2.3–2.5 cm long, pink, somewhat undulate, midpetaline bands darker pink, adpressed pilose; ovary and style pilose; style divided 7 mm above the base, stigmas 3 mm long. Capsule pilose; seeds puberulous. [Sa’ad 1967: 66: Austin and Ghazanfar 1979: 13; Nowroozi 2002: 29 (plate), 100 (map)]

##### Distribution.

China (fide Fang and Staples 1997: 290); Mongolia (*Grabov* 5514, *Przewalski* 48); Russia: Siberia; Kazakhstan (*Schiskin* s.n. [10/7/1937], *Ianatov & Kuznetzov* s.n. [13/6/1956], *Pojarkova* 417, *Spiridonow* s.n. [1914]); Kyrgyzstan (*Dessiatoff* 1759); Uzbekistan (*Kamelin et al.* 589); Tadjikistan (*Botchantsev* 237, *Browicz* 76); Turkmenistan (*Borissova* 3768b, *Rodin et al.* 205); Afghanistan (*Aitchison* 418; *Koie* 4413); Iran (*Schmidt* 6150; *Merton* 3943, *Andersen & Jensen* 2100, *Rechinger* 5502).

#### 139. Convolvulus gortschakovii

Taxon classificationPlantaeSolanalesConvolvulaceae

Schrenk, Enum. Pl. Nov. [F.E.L. Fischer & C.A. Meyer] 1: 18. 1841. (Fischer and Meyer 1841: 18).

Figure 18: t. 21–26

Convolvulus pungens Kar. & Kir., Bull. Soc. Imp. Naturalistes Moscou 14: 709. 1842. (Karelin and Kiriloff 1842: 405). Type. KAZAKHSTAN/RUSSIA, Songaria, *Karelin & Kiriloff* 326 (syntypes LE!, W!).

##### Type.

KYRGYZSTAN, *Schrenk*, s.n. (holotype LE!; isotypes BM001046244!, OXF!, W!).

##### Description.

Low spiny undershrub, often cushion-like, 10–20(-30) cm high, stems divaricate, branches arising at right angles, grey-sericeous when young, persistent and woody-spinescent when old; cauline spines also present. Leaves sessile, 1–2.5 × 0.2–0.5 cm, oblanceolate, acute, entire, tapering to a long petiole-like base. Flowers solitary, axillary, pedunculate; peduncles 5–7 mm, woody, straight, spinescent; bracteoles 6–12 × 1–2 mm, foliose, linear to linear-oblong; pedicels 1–2 mm; outer sepals 7–11 × 7–11 mm, orbicular, apex rounded and apiculate, base cordate, glabrous, papery in texture; inner sepals 5–7 × 4–5 mm, ovate, acuminate; corolla 1.8–2.2 cm long, pink, undulate but not lobed, midpetaline bands brownish-pilose terminating in a small tooth; ovary pubescent; style divided 7 mm above base, pubescent below; stigmas 3 mm. Capsule apically pubescent; seeds not seen.

Similar in inflorescence, habit and overall morphology to *Convolvulus fruticosus* but distinctive because of the glabrous orbicular cordate papery outer sepals, which are much larger than the ovate, acuminate inner sepals.

##### Distribution.

Kyrgyzstan (*Karelin & Kirilov* 326); Kazakhstan (*Saposhnikov* 1914, *Mishenkova* 331); Tajikistan; Russia: Altai (*Ladrigin* 445), Mongolia (*Cheney* 65, *Potanin* s.n. [13/8/1879]), China (fide Fang and Staples 1997: 289).

##### Notes.

There are a number of specimens in which the sepals are pilose but similar in shape to those of *Convolvulus gortshakovii*. These include *Kossinsky* 889 from Kazakhstan (Semipalatinsk) and *Przewalski* s.n. [25/5/1879], *Kalinina* s.n. [17/8/1949] and *Klemenz* 178 from Mongolia (all at LE). Without further evidence it is impossible to decide whether these are hybrids or some other kind of intermediate with *Convolvulus fruticosus*.

#### 140. Convolvulus spinosus

Taxon classificationPlantaeSolanalesConvolvulaceae

Burm.f., Fl. Ind. (N.L. Burman) Prodr. Fl. Cap. 47. 1768. (Burman 1768: 47).

Figure 18, t. 27–33

Convolvulus genistoides Jaub. & Spach, Ill. Pl. Orient. 4: 107. 1852. (Jaubert and Spach 1852: 107). Type. IRAN, *Aucher-Eloy* 4933 (holotype P!).
Convolvulus affghanus Vatke, Österr. Bot. Z. 25: 168. 1875. (Vatke 1875: 168). Type. AFGHANISTAN, *Griffith* 5857 (holotype W!; isotypes C, GOET, K!).

##### Type.

IRAN, *Garzin* s.n. (holotype G).

##### Description.

Intricately branched spiny undershrub to 60 cm, stems finely adpressed pubescent, branches spinescent and with some stem spines near branch tips formed from old peduncles. Leaves sessile, 0.5–1.2 × 0.3–0.4 cm, oblong to oblanceolate, obtuse, margin entire, base attenuate, adpressed-pubescent. Flowers axillary, usually solitary, sometimes up to 3 in a diachasial cyme; peduncles 4–8 mm, woody, spinescent, bent at apex; bracteoles 1–1.5 mm, scale-like; pedicels c. 1 mm; outer sepals 3 × 2 mm, oblong to elliptic, obtuse and mucronate, convex, pubescent with spreading hairs; inner sepals 4.5–5 × 3 mm, pubescent with spreading hairs; corolla 1.3–1.6 cm long, white, midpetaline bands adpressed pilose; ovary pubescent; style pubescent, divided c. 4 mm above base, stigmas 3 mm; capsule comose; seeds smooth, glabrous. [Sa’ad 1967: 70; Austin and Ghazanfar 1979: 12; Nowroozi 2002: 27 (plate), 100 (map)]

##### Distribution.

Iran (*Parris* 75.477, *Faroughi* 10731, *Ruenemark et al.* 22367, *Aucher-Eloy* 1279); Afghanistan (*Breckle* 1074); Pakistan: Balochistan (*Lamond* 1224, *Ghafoor & Goodman* 4994, *Rechinger* 28121, 29401). Most common in Balochistan.

##### Notes.

The inner sepals are noticeably longer than the outer sepals.

#### 141. Convolvulus argyracanthus

Taxon classificationPlantaeSolanalesConvolvulaceae

Rech.f., Aellen & Esfand., Anz. Österr. Akad. Wiss., Math.-Naturwiss. Kl. 87: 303. 1950. (Rechinger et al. 1950: 303).

Figure 18, t. 34–41

##### Type.

Iran, *K.H. Rechinger et al.* 3228 (holotype W!; isotypes E!, G, K!).

##### Description.

Grey spiny undershrub to 60 cm, stems grey-sericeous, spinescent, lateral spines formed from dead peduncles present near branch tips. Leaves sessile, 1–1.7 × 0.3–0.6 cm, obtuse, margin entire, base attenuate, grey-sericeous. Flowers solitary (or, fide Sa’ad (1967), 2–3 in axillary diachasia), axillary, pedunculate; bracts leaf-like; peduncles 3–4 mm, woody; bracteoles c. 1.5 mm, linear, obtuse; pedicels 1.2 mm long, bent at an angle to the peduncle; sepals all similar, 3–6 × 2–4 mm, obovate, mucronulate, abruptly narrowed at base, adpressed-pilose; corolla 1.6–2.2 cm, white with slight bluish flush, weakly lobed, midpetaline bands pilose; ovary pilose, style pubescent, divided c. 7 mm above base, stigmas 3 mm; capsule and seed not known. [Sa’ad 1967: 63; Rechinger 1963: f. 2]

##### Distribution.

Iran: Kerman, Fars (*Davis & Bokhari* 56223, *Grey-Wilson & Hewer* 301, *Popov* 51/75, *Foroughi* 10731).

##### Notes.

Resembles *Convolvulus spinosus* but most easily distinguished by the obovate leaves and all five sepals equal in size and shape.

#### 142. Convolvulus acanthocladus

Taxon classificationPlantaeSolanalesConvolvulaceae

Boiss. & Kotschy, Diagn. Pl. Orient. 7: 27. 1846. (Boissier 1846: 27).

Figure 19, t. 16–23

##### Type.

IRAN, Shiraz, *Kotschy* 352 (lectotype G, designated by Sa’ad 1967: 62; isolectotypes BM000047962, C, E!, FI, GOET, HAL, JE, K!, OXF!, P!, W!).

##### Description.

Intricately branched spiny undershrub, 10–60 cm high, stems woody, weakly divaricate and spreading at a wide angle, grey-sericeous, spine-tipped and with numerous short lateral spines on older shoots formed from old peduncles. Leaves sessile, 0.5–1.5 × 0.2–0.4 cm, oblong-elliptic, oblanceolate or oblong-lanceolate, obtuse, margin entire, attenuate at the base, grey-sericeous. Inflorescence of few-flowered terminal clusters, sometimes also with solitary flowers in the leaf axils below; peduncles 3–8 mm, rigid, spinescent, sericeous; bracteoles 4–6 × 0.5–1.5 mm, linear, acute; pedicels 1–3 mm, villous, sometimes recurved; outer sepals 7–10 × 2–3 mm, ovate, abruptly narrowed into an acuminate and mucronate apex, densely pilose with long, pinkish hairs, inner sepals narrower and with scarious margins; corolla 1.5–2.6 cm, pink or white, very shallowly lobed, the midpetaline bands with long hairs; ovary and style pilose; style divided 6–7 mm above base, the stigmas relatively short, c. 2 mm long; capsule pilose at apex, one-seeded; seeds puberulous. [Sa’ad 1967: 62; Rechinger 1963: 8; Nowroozi 2002: 22, 25 (plate), 100 (map); Austin and Ghazanfar 1979: 10; Jongbloed 2003: 309 (photo)]

**Figure 19.**
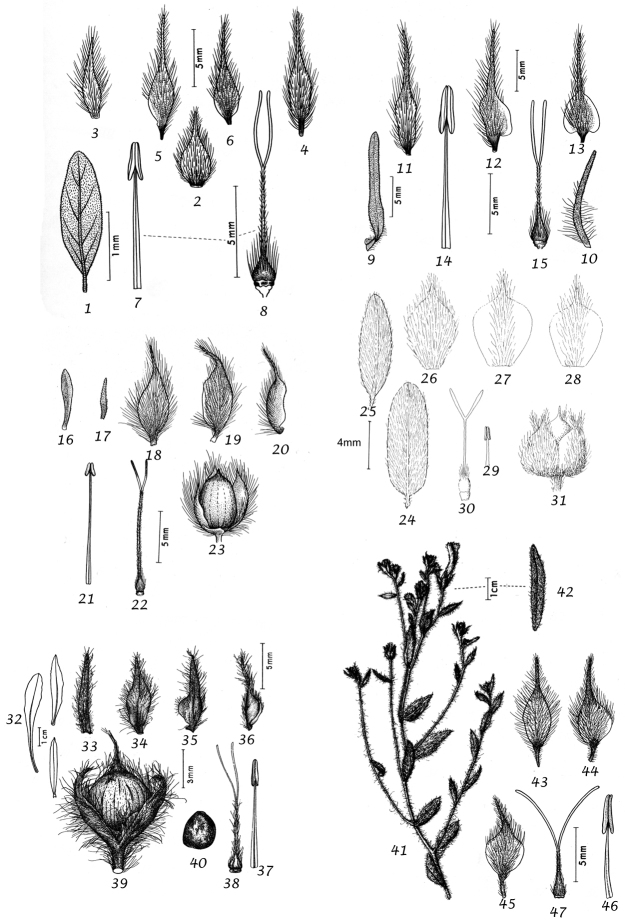
**1–8** *Convolvulus turrillianus* **1** leaf **2** bract **3** bracteole **4** outer sepal **5** middle sepal **6** inner sepal **7** stamen **8** ovary and style. From *Mirzagen* 830E (B) **9–15** *Convolvulus urosepalus* **9** leaf **10** bracteole **11** outer sepal **12** middle sepal **13** inner sepal **14** stamen **15** ovary and style. From *Koelz* 18056 (W) **16–23** *Convolvulus acanthocladus* **16** leaf **17** bracteole **18** outer sepal **19** middle sepal **20** inner sepal **21** stamen **22** ovary and style **23** capsule **16–22** from *Stapf* 362 (W) **23** from *Stapf* 361 (K) **24–31** *Convolvulus iranicus* **24** leaf **25** bract **26** outer sepal **27** middle sepal **28** inner sepal **29** stamen **30** ovary and style **31** capsule. From *Alava & Bokhari* 10629 (E) **32–40** *Convolvulus cantabrica* **32** leaves **33** bracteole **34** outer sepal **35** middle sepal **36** inner sepal **37** stamen **38** ovary and style **39** capsule **40** seed. From *Haussknecht* s.n. (JE) **41–47** *Convolvulus aucheri* **41** habit **42** bract **43** outer sepal **44** middle sepal **45** inner sepal **46** stamen **47** ovary and style. From *sin coll.* 1944 (W).

##### Distribution.

Pakistan (fide Austin and Ghazanfar 1979); Iran (*Parris* 75.260; *Léonard* 5877, *Rechinger* 3226, *Remaudière* 1959, *Soltani* 6397B, *Alava & Bokhari* 10755, *Wendelbo & Foroughi* 15771), U.A.E., Oman (*Radcliffe-Smith* 4110, *Mandaville* 6784).

##### Notes.

The spiny sericeous habit, spinescent peduncles and sepals with very long hairs make this distinct from all but *Convolvulus iranicus*. However both corolla and sepal length is quite variable.

#### 143. Convolvulus iranicus

Taxon classificationPlantaeSolanalesConvolvulaceae

J.R.I.Wood & R.W.Scotland sp. nov.

urn:lsid:ipni.org:names:77147663-1

Figures 2d and 19, t. 24–31

##### Diagnosis.

*Convolvulus acanthocladi* Boiss. & Kotschy similis sed sepalis parvioribus, 4–4.5 (non 7–10) mm longis, tenuiter pilosis, floribus solitariis, corolla valde breviore (1.5–1.7 cm non usque 2.5 cm) longa, ramis brunneolis, pubescentibus pilis disperses, adpressis, non albo-canescentibus.

##### Type.

IRAN, Fars province, 20 km from Shiraz along road to Bushehr, 26 Jun 1972, *Reino Alava & M.H. Bokhari* 10629 (holotype W!; isotypes E00456799!, TUR, not seen).

##### Description.

Much branched, spiny woody undershrub forming a compact intricate bushlet c. 20–25 cm high, young stems pale brown with appressed hairs. Branch tips spinescent, lateral spines present, formed from old fertile and (towards the branch tips) sterile peduncles. Leaves sessile, 7–10 × 3–4 mm, oblanceolate to obovate, obtuse, entire, attenuate at the base, appressed pubescent with whitish hairs. Flowers solitary, axillary; bracts resembling reduced leaves, 2–3 × 1.5–2 mm, oblong-elliptic, obtuse; peduncles 12–14 mm, straight, rigid, spinescent, arising at 90° from the branch, appressed pubescent; bracteoles c. 0.25 mm, minute, filiform; pedicels 1–1.5 mm, often bent at 90° to the peduncle, distinctly more densely pubescent with spreading hairs that the peduncle; outer sepals 4–4.5 × 3 mm, elliptic-rhomboid, acute to shortly acuminate, thinly pilose, often (?always) purplish, inner sepals c. 4 × 1.5 mm, colourless, very thinly pilose; corolla 1.5–1.7 cm, base somewhat inflated, colour unknown, midpetaline bands densely pilose; filaments glabrous, anthers 1.25 mm, ovary subglobose, c. 1 mm, thinly pilose; style 5 mm long, pilose, stigmas 2 mm, slightly widened apically. Capsule and seeds unknown.

##### Distribution.

Only known from the type.

##### Notes.

Most similar to *Convolvulus acanthocladus* but indumentum much less dense, the stem brown and thinly adpressed-pubescent rather than grey-sericeous, the corolla and sepals much shorter, the sepals more or less rhomboid being widest in the middle and gradually narrowed to the apex. The flowers appear always to be solitary.

This species is only known from a single collection. It may be rare and threatened or simply overlooked but for the time being should be classified as Data Deficient (DD) within IUCN (2012) guidelines. Nothing is known of its habitat except that it grows on a hillside. The epithet “*iranicus*” refers to Iran, to which this species is endemic.

#### 144. Convolvulus urosepalus

Taxon classificationPlantaeSolanalesConvolvulaceae

Pau, Trab. Mus. Ci. Nat., Ser. Bot. 14: 27. 1918. (Pau 1918: 27).

Figure 19, t. 9–15

##### Type.

IRAN, *de la Escalera* s.n. (holotype MA-94152!).

##### Description.

Similar to *Convolvulus acanthocladus*. Stems erect, straight and spreading at a very acute angle to the main axis, grey-sericeous and pilose with spreading hairs, branches spine-tipped but lateral spines lacking. Leaves linear, 5–15 × 1–2 mm. Flowers solitary or paired in the uppermost leaf axils, sessile, characteristically overtopped by the spinescent branches; sepals 12–13 mm long, similar in shape to those of *Convolvulus acanthocladus* but more abruptly caudate with a pronounced apical mucro and distinctly longer; corolla c. 2.5 cm long, white but pinkish in bud. [Rechinger 1961: 22; 1963: 25; Nowroozi 2002: 25 (plate), 100 (map)]

##### Distribution.

Endemic to Iran (*Koelz* 18056).

#### 145. Convolvulus turrillianus

Taxon classificationPlantaeSolanalesConvolvulaceae

Parsa, Kew Bull. 3: 213. 1948. (Parsa 1948: 213).

Figure 19, t. 1–8

##### Type.

IRAN, Karevandar, *Parsa* 566 (holotype K).

##### Description.

Small undershrub to c. 25 cm, stems and branches sericeous, spinescent, sterile cauline spines present, few. Leaves sessile, 1.5–3 × 0.5 cm, obovate or elliptic, obtuse, entire, the base attenuate to a petiole-like base, densely sericeous. Flowers in compact capitate cymes, terminal on naked peduncle-like branches; bracts ovate, acuminate, densely pilose; bracteoles c. 8 × 2 mm, linear-lanceolate, finely acuminate, densely pilose; pedicels absent; sepals 8–9 × 1.5–2.5 mm, lanceolate, acuminate, pilose; corolla c. 1.8 mm long, cream, midpetaline bands pilose; ovary pilose; style pubescent, divided c. 5 mm above base, stigmas c. 3 mm. Capsule not seen. [Sa’ad 1967: 85; Nowroozi 2002: 37 (plate), 101 (map), Rechinger 1963: Figure 3]

##### Distribution.

Endemic to southeastern Iran (*Popov* 51/157, *Rechinger* 55064, *Mirzayan* 830E, *Assadi in Rechinger* 54802, *Runemark et al.* 22616).

##### Notes.

Superficially similar to *Convolvulus oxysepalus* but the stem and leaves greyish-sericeous with adpressed hairs, the plant more obviously spiny with short-lateral spinescent branchlets in addition to the spinescent main shoots; the ovary is densely pilose and the style thinly pubescent, the stigmas only 3 mm long.

#### 146. Convolvulus cantabrica

Taxon classificationPlantaeSolanalesConvolvulaceae

L., Sp. Pl. 1: 158. 1753. (Linnaeus 1753: 158)

Figure 19, t. 32–40

Convolvulus terrestris L., Sp. Pl. ed. 2, 1: 224. 1762. (Linnaeus 1762: 224). Type. “In Europa Australi & Africa” (lectotype LINN No. 218.49!, designated by Sa’ad 1967: 124).
Convolvulus linearifolius Mill., Gard. Dict. ed. 8: 28. 1768. (Miller 1768: 28). Type. Cultivated plant from Chelsea Physic Garden (holotype BM001035797!).
Convolvulus cantabrica var. terrestris (L.) L., Mant. Pl. 2: 236. 1771. (Linnaeus 1771: 236). Type. Based on *Convolvulus terrestris* L.
Convolvulus linearis Lam., Fl. Franç. 2: 267. 1779. (Lamarck 1779: 267). Type. specimen in Herb. Tournefort (P, not seen).
Convolvulus terminalis Salisb., Prodr. Stirp. Chap. Allerton 125. 1796, illegitimate superfluous name or possibly a variant of *Convolvulus cantabrica* L. (Salisbury 1796: 125). Type. No type cited.
Nemostima cantabrica (L.) Raf., Fl. Tellur. 4: 82. 1838. (Rafinesque 1838: 82). Type. Based on *Convolvulus cantabrica* L.
Convolvulus dorycnioides De Not., Repert. Fl. Ligust. 283. 1844. (De Notaris 1844: 283). Type. ITALY, Liguria, *Traverso* s.n. (whereabouts uncertain).
Convolvulus cardiosepalus Boiss., Fl. Orient. [Boissier] 4: 96. 1875. (Boissier 1875b: 96). Type. TURKEY, near Bouloukli, *Balansa* 698 (holotype G).
Convolvulus longipilis Gand., Dec. Pl. Nov. [Gandoger] 2: 14. 1876. (Gandoger 1876: 14). Type. *Gandoger* (LY, not seen).
Convolvulus leptosepalus Gand., Dec. Pl. Nov. [Gandoger] 2: 15. 1876. (Gandoger 1876: 15). Type. *Gandoger* (LY, not seen).
Convolvulus villiflorus Gand., Dec. Pl. Nov. [Gandoger] 2: 15. 1876. (Gandoger 1876: 15). Type. *Gandoger* (LY, not seen).
Convolvulus cantabrica var. villosus Post, Fl. Syria 560. 1896. (Post 1896: 560). Type. LEBANON, Mount Cassius (whereabouts unknown, probably BEI).
Convolvulus cantabrica subsp. medius Bornm., Beih. Bot. Centralbl. 20(2): 181. 1906. (Bornmüller 1906: 181). Type. IRAN, Sultanabad, *Strauss* s.n. (holotype B?†).
Convolvulus euxinus Petrov, Byull. Moskovsk. Obshch. Isp. Prir., Otd. Biol., n.ser., 44: 142. 1935. (Petrov 1935: 142). Type. RUSSIA, “Abkasia”, *W. Steup* (holotype LE?).

##### Type.

“In Italia, Sicilia, Narbona, Verona” (lectotype LINN No. 218.48!, designated by Sa’ad 1967: 124).

##### Description.

Undershrub with stout woody base and erect or ascending stems to 50 cm; stems commonly branched in the lower half; all vegetative parts pilose to pubescent. Leaves sessile, the basal and lowermost leaves 4–8 × 0.5–2 cm, obovate-spathulate with a long attenuate petiole-like base; cauline leaves 2–5 × 0.2–0.8(-1.5) cm, linear, linear-oblong, lanceolate or oblanceolate, acute, entire, cuneate at the base. Flowers in long-pedunculate axillary and terminal diachasial cymes, these sometimes congested and subcapitate, often forming a large lax, panicle-like inflorescence or, sometimes, appearing in the form of a lax raceme; bracts leaf-like, linear to oblong, diminishing in size upwards; peduncles 1–9 cm long; bracteoles 5–7 mm, linear, pedicels 0–10 mm; outer sepals 7–8 × 4–5 mm, lanceolate, acuminate to caudate, densely pilose, the lower half colourless, the apical portion green, the inner sepals slightly broader, ovate; corolla 1.7–2.5 cm long, pink, unlobed, midpetaline bands pilose, darker; ovary pilose, style pilose, divided c. 4 mm above base, stigmas 3 mm. Capsule pilose, seeds puberulent. [Sa’ad 1967: 121; Tohmé and Tohmé 2007: 214 (photo); Silvestre 2012: 265; Pignatti 1982: 387]

##### Distribution.

Mediterranean region east to Afghanistan: Morocco (*Hooker* s.n. [5/1871]); Algeria (*Tiaret* s.n. [4/1858]); Tunisia (*Fay* 1150); Spain (*Canto et al.* s.n. [20/5/1982]); Balearic Islands; France (*Hepper* 9491, *Jolinon* 607, *Schulz* 173); Corsica (*Chevalier* s.n. [21/6/1889]); Sardinia; Sicily (*Todaro* 627); Italy (*Pamannini* s.n. [26/6/1912]); Croatia; Greece (*Stainton* 7759); Albania (*Alston & Sandwith* 1450); Slovakia (*Weber* s.n. [5/1935]); Hungary (*Kovacs* 978); Romania (*Wisniewski* 2038); Bulgaria (*Schneider* 989); Bosnia and Herzegovina (*Callier* 112); Ukraine; Russia (*Karpov* s.n. [6/1998], *Marcovicz* 3766); Turkey (*Dudley* 34927, *Leblebici et al.* s.n. [5/6/1972], *Sintenis* 318, *Duzenli* 681); Lebanon (*Maitland* 433); Syria (*Haradjian* 3494); Palestine (*Meyers* 3345); Iran (*Furse & Synge* 275, *Gauba* 81); Azerbaijan (*Klochkova* 386); Afghanistan (*Hewer* 1237); Armenia (*Campbell* 165); Georgia (*Davis* 33807).

##### Notes.

This species can look very similar to forms of *Convolvulus pilosellifolius* but differs in the larger corolla and pilose ovary and capsule.

#### 147. Convolvulus aucheri

Taxon classificationPlantaeSolanalesConvolvulaceae

Choisy, Prodr. [A.P. de Candolle] 9: 402. 1845. (Choisy 1845: 402).

Figure 19, t. 41–47

##### Type.

TURKEY, (Gazi)antap, *Aucher-Eloy* 1405 (lectotype G-DC, designated by Sa’ad 1967: 108); isolectotypes K!, OXF!, P!).

##### Description.

Similar to *Convolvulus cantabrica* in general appearance and most characteristics but differing in the always densely spreading pilose indumentum, the always oblong leaves 1.5–5 × 0.5–1 cm, flowers in loose terminal cymes, usually solitary at the apex of the cyme branches. The sepals are green in the apical part but whish in the basal part and the corolla is white or pale pink with prominent pink midpetaline bands. [Sa’ad 1967: 108]

##### Distribution.

Apparently endemic to Turkey, being found principally around Gaziantap (*Balls* 1184, *Albury et al.* 1055). Records from Syria require confirmation.

##### Notes.

Sa’ad’s (1967) placement of this species with *Convolvulus turcomanicus* and *Convolvulus gracillimus* is misleading as bracts and bracteoles are present and the species is clearly very close to *Convolvulus cantabrica* in floral details.

#### 148. Convolvulus schirazianus

Taxon classificationPlantaeSolanalesConvolvulaceae

Boiss., Diagn. Pl. Orient. 11: 82. 1849. (Boissier 1849: 82).

Figure 20, t. 15–22

Convolvulus chamaerhacos Bornm., Mitth. Thüring. Bot. Vereins, n.f., 37: 53. 1927. (Bornmüller 1927). Type. IRAN, Mount Kuh-i-Besri, Sultanabad, *Strauss* s.n. (holotype B).

##### Type.

IRAN, *Kotschy* 379 (holotype G; isotypes BM, C, E, GOET, FI, OXF, P, W).

##### Description.

Woody based perennial with deep taproot, usually branched at base with several ascending stems in a loose tuft to c. 35 cm in height, stems and all vegetative parts obscurely to densely appressed pubescent. Leaves sessile, 3 × 0.2–0.3 cm, linear, acute, entire, attenuate at base. Inflorescence of terminal heads, occasionally with a few flowers in the uppermost leaf axil; bracts 6–9 × 1.5–2 mm, lanceolate; peduncles and pedicels absent; bracteoles 7 × 1 mm, linear, acute, often overtopping the head; outer sepals 8–9 × 2.5 mm, ovate, abruptly narrowed around the middle with a long acuminate apex, villous; inner sepals similar but with glabrous margins; corolla 1.7–2.5 cm long, cream, midpetaline bands pilose; ovary pilose; style pilose, divided 7 mm above the base, stigmas 4 mm. Capsule pilose. [Sa’ad 1967: 119]

**Figure 20.**
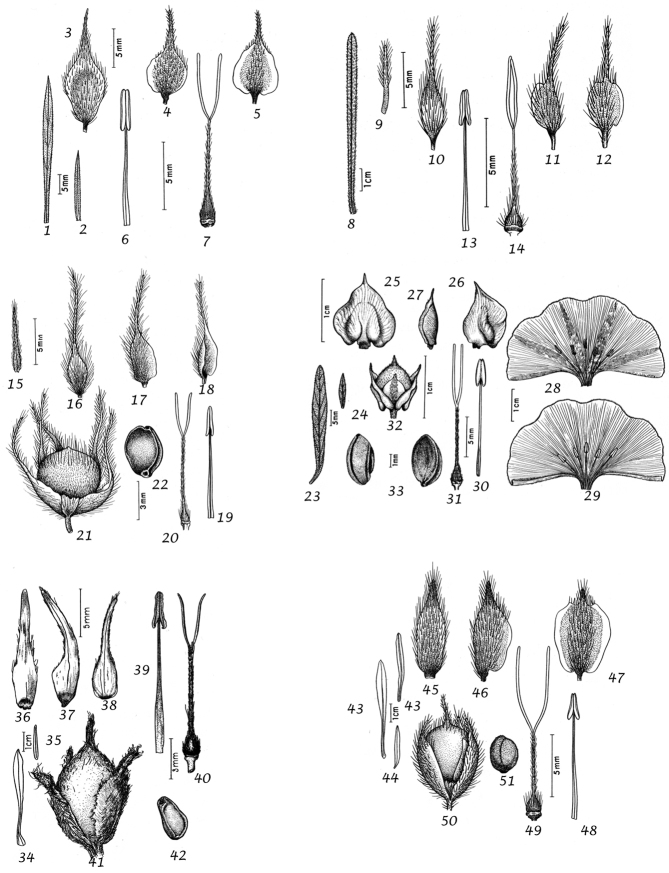
**1–7** *Convolvulus sericocephalus* **1** leaf **2** bract **3** outer sepal **4** middle sepal **5** inner sepal **6** stamen **7** ovary and style. From *Yanata & Doych* s.n. (LE) **8–14** *Convolvulus calvertii* **8** leaf **9** bracteole **10** outer sepal **11** middle sepal **12** inner sepal **13** stamen **14** ovary and style. From *Calvert & Zohrab* s.n. (E) **15–22** *Convolvulus schirazianus* **15** bracteole **16** outer sepal **17** middle sepal **18** inner sepal **19** stamen **20** ovary and style **21** capsule **22** seed. From *Kotschy* 379 (W) **23–33** *Convolvulus holosericeus* **23** leaf **24** bracteole **25** outer sepal **26** middle sepal **27** inner sepal **28** exterior of corolla showing midpetaline bands **29** interior of corolla showing stamens **30** stamen **31** ovary and style **32** capsule **33** seed **23–31** from *Sintenis* 4082 (JE) **32–33** from *Sintenis* 4082b (GOET) **34–42** *Convolvulus lineatus* **34** leaf **35** bracteole **36** outer sepal **37** middle sepal **38** inner sepal **39** stamen **40** ovary and style **41** capsule **42** seed **34–40** from *Spencer* 17/4/1893 (G) **41–42** from *Roux* 8/7/1860 (G) **43–51** *Convolvulus oleifolius* **43** leaves **44** bracteole **45** outer sepal **46** middle sepal **47** inner sepal **48** stamen **49** ovary and style **50** capsule **51** seed **39–49** from *Rechinger* 7819b (W) **50–51** from *Tunta* 901 (W).

##### Distribution.

Endemic to Iran (*Schmidt* 5474, *Grant* 17704, *Stapf* 375).

##### Notes.

Distinguished from other species with a capitate inflorescence by its narrow, linear leaves and adpressed stem indumentum. The sepals are shorter than in *Convolvulus commutatus*.

#### 149. Convolvulus commutatus

Taxon classificationPlantaeSolanalesConvolvulaceae

Boiss., Diagn. Pl. Orient. 11: 81. 1849. (Boissier 1849: 81).

Convolvulus modestus Boiss., Diagn. Pl. Orient. 11: 82. 1849. (Boissier 1849: 82). Type. AZERBAIJAN, *Aucher-Eloy* 4947 (holotype G; isotypes BM001046241!, OXF!, P!, W!).

##### Type.

IRAQ, Mosul, *Aucher-Eloy* 1411 (holotype G; isotypes BM001046242!, OXF!, P!).

##### Description.

Woody based perennial with thick woody taproot, usually branched at base, sometimes more or less cushion-forming, with several erect stems in a loose tuft to c. 40 cm in height, stems and all vegetative parts densely appressed pilose. Basal leaves 3–5 × 0.4–0.8 cm, oblanceolate to spathulate, acute, entire, attenuate at base; stem leaves sessile, 1.5–4.5 × 0.2–0.8 cm, oblong-lanceolate, acute, entire, base cuneate to attenuate. Inflorescence mostly of sessile terminal heads but sometimes with 1–2 flowers in the axils of the upper leaves; heads with 1–5 flowers; bracts 10–22 × 1.5–3 mm; peduncle absent; bracteoles 4–6 × 1 mm, linear, pedicels 0–3(-9) mm; outer sepals 11–13 × 3–4 mm, broadly lanceolate, abruptly narrowed to a long attenuate apex, pilose; inner sepals ovate but slightly shorter, c. 10 mm long; corolla 2–2.8 cm long, pale pink or white, unlobed, midpetaline bands adpressed pilose, pink; ovary pilose, style pilose, divided 6–8 mm above base, stigmas 3 mm; capsule pilose, 1-seeded (fide Sa’ad 1967), seeds puberulous. [Sa’ad 1967: 116; Rechinger 1963: 14]

##### Distribution.

Iran (*Grossheim & Schiskin* 215, *Furse & Synge* 664, *Merton* 3516, *Bornmüller* 7642, *Cowan & Darlington* 1487); Iraq; Azerbaijan (*Aucher-Eloy* 4947, *Wendelbo et al.* 11891); Armenia (*Schelkovnikov* s.n. 31/5/1926). Records from Turkey are unconfirmed.

##### Notes.

The short-pedicellate flowers which give the inflorescence a looser appearance than in related species are noteworthy. The appressed indumentum especially on the stem serves to separate this species from *Convolvulus calvertii*.

#### 150. Convolvulus elymaiticus

Taxon classificationPlantaeSolanalesConvolvulaceae

Mozaff., Iranian J. Bot. 16: 206. 2010. (Mozaffarian 2010: 206).

##### Type.

IRAN, Ilam, *Mozaffarian* 88391 (holotype TARI).

##### Description.

Very similar to *Convolvulus commutatus* differing in the indumentum of long spreading hairs and the inflorescence with rather loose heads, arising at both the apex of the stem and in the upper 3 leaf axils.

##### Distribution.

Endemic to Iran and only known from the type collection.

##### Notes.

*Convolvulus elymaiticus* appears to lie between *Convolvulus commutatus* and *Convolvulus calvertii*. The lax inflorescence, white corolla and geographical distribution suggest an affinity with *Convolvulus commutatus* while the corolla size (2 cm long) places it in intermediate position. The spreading hairs on the stem, however suggest a closer affinity with *Convolvulus calvertii* and the presence of individual flowers in the uppermost leaf axils, which also occurs occasionally in *Convolvulus calvertii*, confirms this. Without seeing material it is impossible to be certain whether this is a good species or not but it seems likely to be a form of the variable *Convolvulus calvertii*.

#### 151. Convolvulus calvertii

Taxon classificationPlantaeSolanalesConvolvulaceae

Boiss., Diagn. Pl. Orient., ser. 2, 3: 124. 1856. (Boissier 1856: 124).

##### Type.

TURKEY, *Calvert & Zohrab* 1282 (holotype G; isotypes E!, OXF!).

##### Description.

Variable woody based perennial with thick woody taproot, sometimes cushion-forming, with several ascending stems in a loose tuft to c. 20 cm in height, stems and all vegetative parts with long spreading hairs. Basal leaves 4–8 × 0.3–0.8 cm, oblong or oblanceolate, acute, entire, narrowed to a long petiole-like base; stem leaves sessile, 3–5 × 0.6–0.9 cm, oblong-elliptic, acute, entire, cuneate at the base. Flowers in many-flowered terminal heads, occasionally also 1–2 flowers in the axil of the uppermost leaf; bracts 1.3–3 × 0.1–0.6 cm, linear-oblong or lanceolate, acute, pilose; bracteoles 11–16 × 0.5–1 mm, linear, pilose; pedicels 0–3 mm; outer sepals 6–10 × 1.5–2 mm, lanceolate, long acuminate, pilose; inner sepals abruptly narrowed around the middle with a caudate apex; corolla 1.5–2 cm, white or pink, not lobed, midpetaline bands pilose, darker; ovary pilose; style pilose, divided c. 3 mm above base, stigmas 3–5 mm. Capsule pilose; seeds pubescent. [Sa’ad 1967: 114]

##### Notes.

We recognise two subspecies:

#### 151a. Convolvulus calvertii subsp. calvertii

Taxon classificationPlantaeSolanalesConvolvulaceae

Figure 20, t. 8–14

Convolvulus saxatilis M.Bieb., Fl. Taur.-Caucas. 1: 146. 1808, nom. illeg., non *Convolvulus saxatilis*Vahl (1794). (Marschall von Bieberstein 1808: 146). Type. CRIMEA, near Karassubasar and Sympheropolin (whereabouts unknown).
Convolvulus lanuginosus sensu Ledeb., Fl. Ross. 3: 88. 1847, nom. illeg., non *Convolvulus lanuginosus* Desr. (1792). (Ledebour 1847: 88).
Convolvulus calvertii var. tauricus Bornm., Beih. Bot. Centralbl. 22(2): 181.1906. (Bornmüller 1906: 181). Type.CRIMEA, *Callier* 155 (lectotype K! ex Herb Churchill, designated here; isolectotypes E!, HBG, JE, K!, OXF!, P!, STU, W!, reported from LE but not seen there).
Convolvulus tauricus (Bornm.) Juz., Bot. Mater. Gerb. Bot. Inst. Komarova Akad. Nauk. S.S.S.R. 12: 214. 1950. (Juzepczuk 1950: 214). Type. Based on Convolvulus calvertii var. tauricus Bornm.
Convolvulus calvertii subsp. tauricus (Bornm.) Smoljan., Fl. Evropeiskoi Chasti SSSR (A.Federova) 5: 97. 1981. (Smoljaninova 1981: 97) Type. Based on Convolvulus calvertii var. tauricus Bornm.
Convolvulus triqueter Rehmann ex Petrov, Byull. Moskovsk. Obshch. Isp. Prir., Otd. Biol., n.ser., 44: 144. 1935, nom. illeg., non *Convolvulus triqueter*Vahl (1794). (Petrov 1935: 144). Type. Based on *Convolvulus saxatilis* M.Bieb.
Convolvulus bracteosus Juz., Bot. Mater. Gerb. Bot. Inst. Komarova Akad. Nauk. S.S.S.R. 12: 217. 1950. (Juzepczuk 1950: 217) Type. CRIMEA, *Rehmann* 663 (holotype location uncertain, probably LE; isotype P!).
Convolvulus calvertii subsp. bracteosus (Juz.) Smoljan., Fl. Evropeiskoi Chasti SSSR (A. Federova)5: 99. 1981. (Smoljaninova 1981: 99). Type. Based on *Convolvulus bracteosus* Juz.

##### Distinguishing features.

Stems and leaves with prominent spreading hairs as well as appressed sericeous hairs. Leaves oblong or oblanceolate, 5–10 times longer than broad.

##### Distribution.

Turkey (*Balls* 1542, *Stainton & Henderson* 5372, *Watson* 2935); Crimea (*Busch* s.n. [6/6/1905], *Callier* 4563); Iran (*Furse* 7519, *Rechinger* 4765); Turkmenistan (*Polyakova* 226, *Gubanov* 399, *Federov* s.n. [3/6/1917]).

##### Notes.

Bornmüller (1906) did not mention any specific type specimen for his var. tauricus but made reference to collections from Crimea by Marschal von Bieberstein and Callier. Juzepczuk (1950) mentioned no type but the only collection cited was *Callier* 155 “pro (maxima?) parte”. Sa’ad (1967) cited the same collection from LE as holotype without qualification and without seeing the specimen. Smolyaninonova (1981) proposed a lectotype from the location “in collibus cretaceis ad Barultsscha prope Karasabazar” but without collector or number. In order to end the uncertainty we have designated *Callier* 155 collected at “Kreideberge in Karakusch bei Karasubazar” at Kew as the duplicate at Berlin is presumed destroyed and we were unable to find a specimen at LE. This material is widely distributed and all specimens we have seen represent the same species.

*Convolvulus bracteosus* is a form of *Convolvulus calvertii* with narrowly oblong-elliptic bracts 5–8 mm wide, these exceeding the head. The basal leaves are oblanceolate. *Juzepczuk & Kurgianov* 1580 (LE) is a good example of this form. The exact identity of *Convolvulus saxatilis* is uncertain but it is probably Convolvulus calvertii subsp. calvertii.

#### 151b. Convolvulus calvertii subsp. ruprechtii

Taxon classificationPlantaeSolanalesConvolvulaceae

(Boiss.) J.R.I.Wood & R.W.Scotland stat. nov.

urn:lsid:ipni.org:names:77147678-1

Convolvulus ruprechtii Boiss., Fl. Orient. [Boissier] 4: 96. 1875. (Boissier 1875b: 96). Type. RUSSIA, Daghestan, *Ruprecht* s.n. (lectotype G 00330221!, specimen from “Daghestania prope Kutuschi,” designated here; isolectotypes G!, LE).

##### Type.

Based on *Convolvulus ruprechtii* Boiss.

##### Distinguishing features.

Leaves and stem silvery with appressed sericeous hairs; spreading hairs absent or almost so. Leaves broadly oblong to obovate, up to three times as long as broad. Petrov 1935: 144 (plate).

##### Distribution.

Crimea (*Vasak* s.n. [29/7/1977]); Russia: Dagestan (*Tsvelev et al*. 1020, 2850, 3164, *Alexeenko* 952, 9355, *Grossheim* s.n. [25/6/1915]); Armenia (*Fayvush et al.* 04-0424); Azerbaijan (*Grossheim et al.* s.n. [10/6/1947]); Iran (*Miller et al.* s.n. [17/5/2005]). Principally in the eastern Caucasus around 1500 m.

##### Notes.

*Convolvulus calvertii* is recognised by the near sessile flowers with bracts often overtopping the heads are distinctive. It is easily confused with *Convolvulus commutatus* and *Convolvulus schirazianus* but is distinguished by the distinct spreading hairs on the stem. *Convolvulus lanuginosus* differs in the pilose ovary and *Convolvulus sericocephalus* in the sparse indumentum of the inflorescence so the sepals are easily visible.

#### 152. Convolvulus sericocephalus

Taxon classificationPlantaeSolanalesConvolvulaceae

Juz., Bot. Mater. Gerb. Bot. Inst. Komarova Akad. Nauk. S.S.S.R. 12: 219. 1950. (Juzepczuk 1950: 219).

Figure 20, t. 1–7

##### Type.

CRIMEA, *Yanata & Doych* 27/5/1913 (lectotype LE, designated here).

##### Description.

Perennial herb with leaves arranged in a basal rosette, from which arise erect stems 30–40 cm high; stems adpressed pilose. Leaves sessile, mostly basal, 2–5 × 0.3–0. 5 cm, linear to oblanceolate, acute, tapered at the base to a pseudopetiole, adpressed pilose to subsericeous. Flowers congested at the top of the stem forming a headlike inflorescence with a single head arising from the uppermost leaf axil; bracts resembling linear-lanceolate reduced leaves, erect, slightly exceeding the inflorescence; peduncles of lateral capitula 2–22 mm, bracteoles filiform, pedicels 0–3 mm, sepals 14–15 × 5–6 mm, obovate, strongly cuspidate, adpressed pubescent and with a few spreading hairs, the inner sepals smaller; corolla 1.8–2 cm long, pink, midpetaline bands pilose; ovary hirsute; style pilose, divided 5 mm above the base; stigmas 4 mm. Capsule not seen.

##### Distribution.

Crimea (*Lindemann* s.n.).

##### Notes.

This species appears to be almost certainly the hybrid between *Convolvulus holosericeus* and *Convolvulus calvertii*, resembling the former in habit, leaf shape and indumentum but the latter in the presence of some spreading hairs on the sepals and the less saccate calyx. Unlike *Convolvulus calvertii* the sparse indumentum makes the sepals clearly visible.

Juzepczuk (1950) did not indicate type specimens for *Convolvulus sericocephalus* and a lectotype was incorrectly proposed by Smolyaninonova (1981) with the location as “Zapadnaya storona Fyeodosiskovo shosse” but without specimen citation. This appears to refer to *Yanata & Doych* s.n. [27/5/1913], designated as lectotype above and, incidentally, the only specimen of this taxon seen by Sa’ad. Sa’ad’s citation of *Juzepczuk* s.n. as the type is an inexplicable error as Juzepczuk does not cite any of his own collections.

#### 153. Convolvulus holosericeus

Taxon classificationPlantaeSolanalesConvolvulaceae

M.Bieb., Fl. Taur.-Caucas. 1: 147. 1808. (Marschall von Bieberstein, 1808: 147).

Figure 20, t. 23–33

##### Type.

CRIMEA, *Marschall von Bieberstein* s.n. (holotype LE!; isotype BM!).

##### Description.

Undershrub with a thick woody taproot from which arise numerous short prostrate to weakly ascending stems to 30 cm forming a mat, vegetative parts all grey-sericeous. Leaves sessile, 2–4.5 × 0.2–0.6 cm, linear, oblong or oblanceolate, acute, entire, base long-attenuate.. Flowers in terminal cymose clusters, usually with single flowers or few-flowered, diachasia, subsessile or borne on peduncles up to 1.7 cm long from the axils of the uppermost leaves; bracts resembling the leaves but smaller, linear- oblong; bracteoles 3–6 × 0.5 mm, linear; pedicels 1–4 mm; outer sepals 10–15 × 5–12 mm, ovate to suborbicular, acuminate, gibbous with broad membranous wing-like margins; inner sepals slightly smaller; corolla 2–2.7 cm long, pale yellow or cream; unlobed but slightly undulate, midpetaline bands adpressed pilose; ovary pubescent, style pubescent, divided c. 7–8 mm above ovary, stigmas 5 mm. Capsule pubescent, seeds puberulent. [Sa’ad 1967: 132; Parris 1978: 207]

##### Notes.

We recognise two subspecies based on sepal size, although intermediates are not uncommon.

#### 153a. Convolvulus holosericeus subsp. holosericeus

Taxon classificationPlantaeSolanalesConvolvulaceae

##### Distinguishing features.

Sepals relatively small and narrow, 7–10 × 5–10 mm.

##### Distribution.

Croatia (?); Greece; Bulgaria; Macedonia (*Soska* s.n. [15/6/1922]); Crimea (*Busch* s.n. [6/6/1905]); Russia: Daghestan (*Woronov* 385); Turkey (*Dönmez* 2507, *Davis & Coode* 36630, *Siehe* 622, *Balls* 1432, *Bourgeau* 241); Syria (*Haussknecht* 1865).

#### 153b. Convolvulus holosericeus subsp. macrocalycinus

Taxon classificationPlantaeSolanalesConvolvulaceae

Hausskn. & Bornm., Mitth. Thüring. Bot. Vereins 6: 66. 1894. (Bornmüller 1894: 66).

##### Type.

TURKEY, Harput, *Sintenis* 427 (lectotype LD, designated by Parris 1978: 208); isolectotypes B, E!, HBG, JE, P!, W!).

##### Distinguishing features.

Sepals large, suborbicular 11–17 × 11–13 mm, the type being an extreme example of the subspecies.

##### Distribution.

Turkey (*Siehe* 622, *Davis & Hedge* 29166); Iraq (*Omar et al.* 49666, *Aucher Eloy* 1409). It appears to be the only subspecies present in Iraq.

##### Notes.

The gibbous sepals are very distinctive in this species. Convolvulus holosericeus var. macrocalycinus was originally lectotypified by Sa’ad (1967: 133) but as she used the erroneous name “*macrosepalus*”, valid lectotypification dates from the *Flora of Turkey* (Parris 1978).

#### 154. Convolvulus boissieri

Taxon classificationPlantaeSolanalesConvolvulaceae

Steud., Nomencl. Bot., ed. 2, 1: 407. 1840. (Steudel 1840: 407).

Convolvulus nitidus Boiss., Elench. Pl. Nov. 65. 1838, nom. illeg., non *Convolvulus nitidus* Desr. (1792). (Boissier 1838: 65). Type. SPAIN, Sierra Nevada, *Boissier* s.n. (holotype G; isotypes C, E!, G, GOET, HAL, JE, K!, L, W!).

##### Type.

Based on *Convolvulus nitidus* Boiss.

##### Description.

Cushion-forming undershrub, stems short, spreading, the bases covered in leaf remains; vegetative parts silvery-sericeous. Leaves sessile, 0.3–2 × 0.2–0.6 cm, obovate to oblanceolate, obtuse or acute, entire, cuneate to a broad base, the venation very prominent, especially below. Flowers usually solitary, terminal or axillary; peduncle 1–3 mm long; bracteoles 4–8 × 0.5–1 mm, linear; pedicels 1–3 mm; sepals 7–10 × 2–3 mm, oblong-lanceolate with caudate apex, base membranous, apex pilose, inner sepals oblong-ovate with membranous margins; corolla 1.7–2.1 cm long, pink, weakly lobed, midpetaline bands darker, pilose; ovary sericeous; style sericeous, divided 3–4 mm above the ovary, stigmas c. 4 mm. Capsule hirsute; seeds glabrous, smooth. [Sa’ad 1967: 138; Silvestre 2012: 264, 263 (plate]

##### Notes.

Divisable into two, geographical disjunct but morphologically similar subspecies:

#### 154a. Convolvulus boissieri subsp. boissieri

Taxon classificationPlantaeSolanalesConvolvulaceae

Figure 21, t. 42–49

##### Distinguishing features.

Leaves obovate or oblanceolate, the hairs weakly appressed to somewhat spreading and rather similar to the indumentum of the calyx.

**Figure 21.**
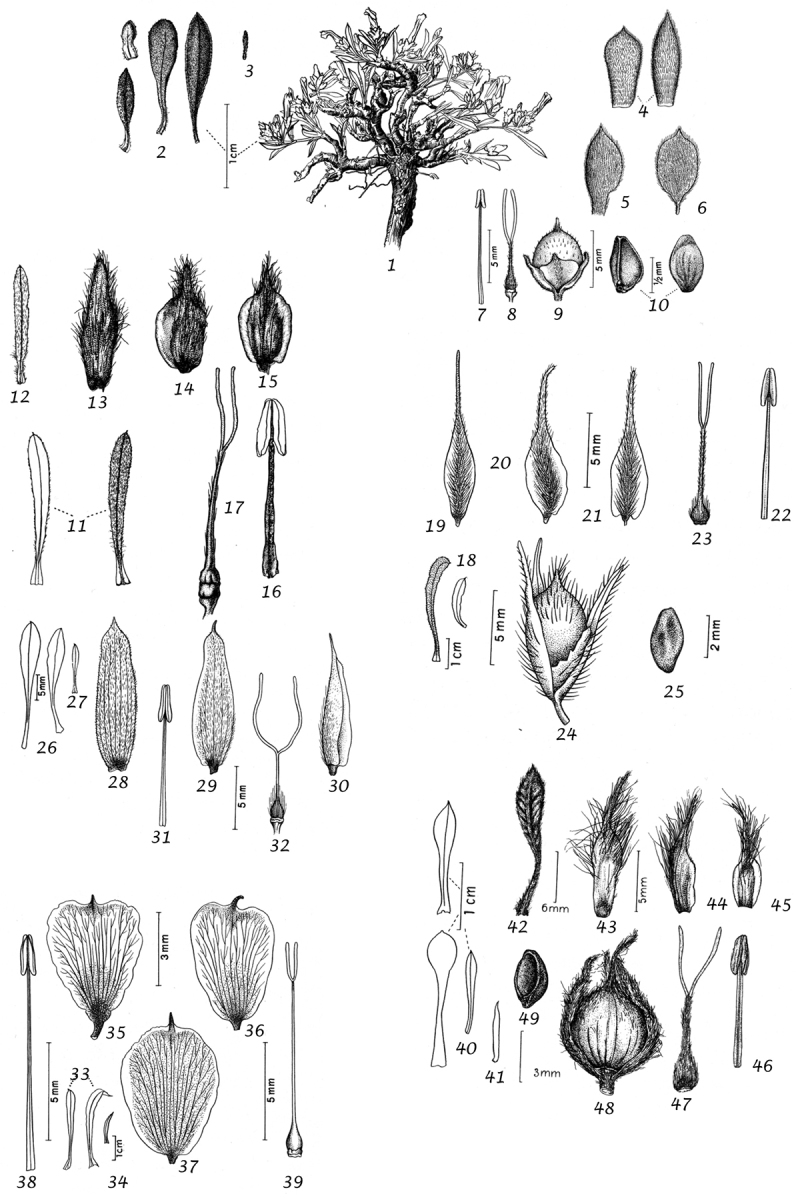
**1–10** *Convolvulus libanoticus* **1** habit **2** leaves **3** bracteole **4** outer sepals **5** middle sepal **6** inner sepal **7** stamen **8** ovary and style **9** capsule **10** seed **1–8** from *Zerny* s.n. (W) **9–10** from *Kotschy* 54 (W) **11–17** *Convolvulus mazicum* **11** leaves **12** bracteole **13** outer sepal **14** middle sepal **15** inner sepal **16** stamen **17** ovary and style. From *Sauvage* 13602 (RAB) **18–25** *Convolvulus cataonicus* **18** leaves **19** outer sepal **20** middle sepal **21** inner sepal **22** stamen **23** ovary and style **24** capsule **25** seed. From *Haussknecht* s.n. (W) **26–32** *Convolvulus phrygius* **26** leaves **27** bracteole **28** outer sepal **29** middle sepal **30** inner sepal **31** stamen **32** ovary and style. From *Scheibe* 1075 (B) **33–39** *Convolvulus carduchorum* **33** leaves **34** bracteole **35** outer sepal **36** middle sepal **37** inner sepal **38** stamen **39** ovary and style. From *Handel-Mazzetti* 2572 (W) **40–41** Convolvulus boissieri subsp. compactus **40** leaves **41** bracteole. From *Kotschy* 139 (W) **42–49** Convolvulus boissieri subsp. boissieri **42** leaf **43** outer sepal **44** middle sepal **45** inner sepal **46** stamen **47** ovary and style **48** capsule, **49** seed **42–47** from *Bourgeau* 784 (G) **48–49** from *Hackel* 8 (W).

##### Distribution.

Spain (*Porta & Rigo* 546, *Bourgeau* 784). In the southern Sierra Nevada region.

#### 154b. Convolvulus boissieri subsp. compactus

Taxon classificationPlantaeSolanalesConvolvulaceae

(Boiss.) Stace, Bot. J. Linn. Soc. 64: 58. 1971. (Stace 1971: 58).

Figure 21, t. 40–41

Convolvulus compactus Boiss., Diagn. Pl. Orient. 4: 40. 1844. (Boissier 1844: 40). Type. TURKEY, Caria, *Pinard* s.n. (lectotype G, designated by Sa’ad 1967: 140; isolectotypes K!, OXF!, W!).
Convolvulus cochlearis Griseb. Spic. Fl. Rumel. 2: 76. 1844. (Grisebach 1844: 76). Type. TURKEY, Eastern Anatolia, *Donietti* s.n. (holotype GOET).
Convolvulus parnassicus Boiss. & Orph., Diagn. Pl. Orient., ser. 2, 3: 125. 1856. (Boissier 1856: 125). Type. Greece, *Orphanides* 2532 (holotype G; isotype K!).
Convolvulus compactus subsp. parnassicus (Boiss. & Orph.) Sa’ad, Meded. Bot. Mus. Herb. Rijks Univ. Utrecht 281: 141. 1967. (Sa’ad 1967: 141). Type. Based on *Convolvulus parnassicus* Boiss. & Orph.
Convolvulus boissieri subsp. parnassicus (Boiss. & Orph.) Kuzmanov, Fl.Nar. Republ. Bulgariya 8: 451. 1982. (Kuzmanov 1982: 451). Type. Based on *Convolvulus parnassicus* Boiss. & Orph.
Convolvulus konyacus Sa’ad, Meded. Bot. Mus. Herb. Rijks Univ. Utrecht 281: 142. 1967. (Sa’ad 1967: 142). Type. TURKEY, between Beyshir and Konya, *Dudley* 35857 (holotype E00285412!; isotype K!).

##### Type.

Based on *Convolvulus compactus* Boiss.,

##### Distinguishing features.

Leaves obovate to narrowly linear-oblanceolate, hairs strongly appressed, sometimes sericeous and clearly differentiated from the spreading hairs of the calyx. [Sa’ad 1967: 140; Polunin 1980 (Plate 35)]

##### Distribution.

Balkan peninsular and Turkey: Croatia (*Botteri* s.n.); Albania (*Alston & Sandwith* 2154); Montenegro; Macedonia (?); Bulgaria (?); Greece (*Rechinger* 9545, *Guicciardi* 2963); Turkey (*Balansa* 1168, *Dudley* 37177, *Davis* 21878, *Post* 528).

##### Notes.

Subsp. compactus is very variable in leaf shape, plants with almost linear leaves from Turkey such as *Balls* 1366 fit the type of *Convolvulus konyacus*, but this only seems to be one extreme in the range of variation.

#### 154b × 153a. Convolvulus × turcicus

Taxon classificationPlantaeSolanalesConvolvulaceae

Aykurt & Sümbül, Ann. Bot. Fenn. 48: 432. 2011. (Aykurt and Sümbül 2011a: 432)

##### Type.

TURKEY, *Aykurt & Kemaloğlu* 2172 (holotype AKDU, not seen).

##### Distinguishing features.

This apparently sterile hybrid differs from *Convolvulus holosericeus* in the absence of pouched sepals and differs from Convolvulus boissieri subsp. compactus in the adpressed sericeous indumentum and in the stigma that is distinctly shorter than (rather than about equalling) the style.

##### Distribution.

Reported from a single locality in central Anatolia.

##### Notes.

This taxon represents Convolvulus holosericeus subsp. holosericeus × Convolvulus boissieri subsp. compactus.

#### 154b × 153b. Convolvulus × peshmenii

Taxon classificationPlantaeSolanalesConvolvulaceae

Aykurt & Sümbül, Nordic J. Bot. 29: 409. 2011. (Aykurt and Sümbül 2011b: 409)

##### Type.

TURKEY, *Aykurt & Kemaloğlu* 1495 (holotype AKDU, not seen).

##### Distinguishing features.

It resembles Convolvulus boissieri subsp. compactus in its cushion-forming habit but differs in its adpressed sericeous outer sepals and stems up to 20 cm high.

##### Distribution.

This hybrid is reported from a single locality in south central Anatolia.

##### Notes.

This taxon represents Convolvulus holosericeus subsp. macrocalycinus × Convolvulus boissieri subsp. compactus.

#### 154b × 157c. Convolvulus × pseudocompactus

Taxon classificationPlantaeSolanalesConvolvulaceae

Aykurt & Sümbül, Nordic J. Bot. 29: 409. 2011. (Aykurt and Sümbül 2011b: 409)

##### Type.

TURKEY, *Aykurt & Kemaloğlu* 1006 (holotype AKDU, not seen).

##### Distinguishing features.

Resembles Convolvulus boissieri subsp. compactus in its cushion-forming habit but differs in its pedicellate flowers and distinct stems 3–10 cm high.

##### Distribution.

Reported from a single locality in SW Anatolia.

##### Notes.

This taxon represents Convolvulus boissieri subsp. compactus × Convolvulus oleifolius var. angustifolius (as var. deserti).

#### 155. Convolvulus suendermannii

Taxon classificationPlantaeSolanalesConvolvulaceae

Bornm., Repert. Spec. Nov. Regni Veg. 43: 152. 1938. (Bornmüller 1938: 152).

Convolvulus boissieri subsp. suendermannii (Bornm.) Kuzmanov, Fl. Nar. Republ. Bulgariya 8: 451 (1982). (Kuzmanov 1982: 451). Type. Based on *Convolvulus suendermannii* Bornm.

##### Type.

Plant from Bulgaria, Ali Botush Mountain, cultivated in Berlin, *Sündermann* s.n. (holotype B!).

##### Distinguishing features.

Intermediate between *Convolvulus boissieri* and *Convolvulus lineatus*. Stems short, ascending; leaves sessile, obovate to oblanceolate, acute, cuneate to a broad base.

##### Distribution.

Endemic to the area of Ali Botush Mountain, Bulgaria

##### Notes.

*Convolvulus suendermannii* is an interesting plant. We agree with Sa’ad (1967) that it has the appearance of *Convolvulus lineatus* but Bornmüller’s comment that it lies in “apparent midposition” between *Convolvulus lineatus* and *Convolvulus compactus* is readily understandable because of its dwarf habit so it is not difficult to see why Kuzmanov (1982) treated it as subsp. suendermannii of *Convolvulus boissieri*. It might well represent the hybrid Convolvulus lineatus × Convolvulus boissieri subsp. compactus. What adds to the interest is the type locality, which is precisely the same place where *Stoïanov* 868 was collected. This is the plant whose identity troubled Turrill and Stace (1971: 57). If this is indeed a hybrid or intermediate in some way between *Convolvulus lineatus* and Convolvulus boissieri subsp. compactus rather than a geographically anomalous population of Convolvulus boissieri subsp. boissieri, the geographical difficulties in Stace’s infraspecific classification of *Convolvulus boissieri* disappear. Some support for this view is provided by the presence of a distinct peduncle-like stem in the part of *Stoïanov* 868 preserved in the envelope of the specimen at Kew. While the leaves are clearly those of *Convolvulus boissieri* the inflorescence is thus atypical of that species and similar to that of the type of *Convolvulus suendermannii*. Another specimen (*Velčev et al.* 711 (W, E) from nearby Slavjanka appears to be the same taxon. Careful field observation is necessary to confirm whether or not *Convolvulus suendermannii* is a hybrid. [Strid 1991: 18–20].

#### 156. Convolvulus lineatus

Taxon classificationPlantaeSolanalesConvolvulaceae

L., Syst. Nat. ed. 10, 2: 923. 1759. (Linnaeus 1759: 923).

Figure 20, t. 34–42

Convolvulus spicifolius Desr. in Lamarck. Encycl. 3: 549. 1792. (Desrousseaux 1792: 549). Type. Plant cultivated in Jarden du Roi (P [Herb. Lam.]).
Convolvulus humilis Salisb., Prodr. Stirp. Chap. Allerton 125. 1796, illegitimate superfluous name for *Convolvulus lineatus* L. (Salisbury 1796: 125).
Convolvulus intermedius Loisel., J. Bot. (Desvaux) 2: 264. 1809. (Loiseleur-Deslongchamps 1809: 264). Type. FRANCE, Avignon, *Requien* s.n. (holotype P, not seen).
Convolvulus gerardii Roem. & Schult., Syst. Veg, ed. 15 bis [Roemer & Schultes] 4: 294.1819. (Roemer and Schultes 1819: 294). Type. No type cited, presumably France, with reference to “Gérard, Fl. Gallo-Prov. 317, n.3”.
Convolvulus besseri Spreng., Syst. Veg. 1: 610. 1824. (Sprengel 1824: 610). Type. UKRAINE, Pedolia, *Besser* s.n. (B†).
Convolvulus nitens K.Koch, Linnaea 22: 743. 1849. (Koch 1849: 743). Type. ARMENIA, Yerevan, *Koch* s.n. (B†).
Convolvulus lineatus var. pentapetaloides Batt., Bull. Soc. Hist. Nat. Afrique N. 12: 27. 1921. (Battandier and Jahandiez 1921: 27). Type. MOROCCO, Oudjda, *Jahandiez* s.n. (holotype MPU009741!).
Convolvulus lineatus var. minutus Maire & Weller, Bull. Soc. Hist. Nat. Afrique N. 31: 28. 1940. (Maire 1940: 28). Type. MOROCCO, Grand Atlas, *Maire & Weller* 628 (holotype AL, not seen; isotype MPU004309!).
Convolvulus tshegemensis Galushko, Novosti Sist Vyssh. Rast. 13: 252. 1976. (Galushko 1976: 252). Type. RUSSIA, northern Caucasus (Balkaria), *Galushko* 12/7/1963 (holotype cited from but not received at LE, whereabouts unknown).

##### Type.

Without locality, *Löfling* 163 (lectotype LINN No. 218.43!, designated by Sa’ad 1967: 128).

##### Description.

Perennial herb, often mat-forming, from a thick, branched underground rhizome; stems ascending to 30 cm, vegetative parts densely but minutely puberulent to shiny white-sericeous. Basal leaves 4.5–8 × 0.8–1.8 cm, oblong to oblanceolate, acute, entire, attenuate at base into a long pseudopetiole; stem leaves distinctly sessile, 1.5–5 × 0.2–0.7 cm, narrowly linear-oblanceolate, acute. Flowers in terminal diachasial cymes and axillary cymes of 1–5 flowers; bracts and bracteoles not clearly differentiated, 3–25 × 1–3 mm, linear to oblanceolate, acute, very variable in size; pedicels 3–4 mm; sepals of two parts–pale base and acute, commonly reflexed, green apex; outer sepals 6–10 × 2 mm, oblong-lanceolate; inner sepals 6–8 × 3 mm, broadly ovate, margins scarious; corolla 1.8–2.5 cm long, pink with white centre and paler midpetaline bands, shallowly lobed, midpetaline bands appressed pilose; ovary conical, sericeous, style pilose, divided 7–10 mm above base, stigmas 5–7 mm; capsule pubescent; seeds shortly pubescent. [Sa’ad 1967: 128; Meikle 1985: 1170; Austin and Ghazanfar 1979: 18; Nowroozi 2002: 65 (plate), 103 (map). Polunin 1980 (Plate 35); Strid and Strid 2009: 400–401 (plate)]

##### Distribution.

Around the Mediterranean and Black Seas and through Iran and central Asia to western China (Xinjiang) and Siberia (Altai): Portugal; Spain (*Ellman & Hubbard* 282, *Lewalle* 9116); France (*André* 21/6/1855); Italy (*Davis & Sutton* 65425); Sicily (*Alexander* 1845); Morocco (*Carine et al.* 367); Algeria (*Alston & Simpson* 37688, *Faure* 25/5/1916); *Tunisia* (*Davis & Lamond* 56988); *Malta* (*Duthie* 4/11/1874); Libya; Egypt (*Ehrenberg* s.n.); Lebanon (*Gombault* 4482); Syria (*Delessert* 1965); Turkey (*Davis* 46875, *Watson* 272, *Callier* 672 ); Cyprus; Greece: Karpathos (*Gathorne-Hardy* 416); Dodecanese (*Raus* 9902); Ukraine (*Shiraevsky* 16/7/1903, *Callier* 672), Armenia (*Gabrilian* 5/6/1975); Georgia (*Hohenacker* 6/1831, *Kozlowsky* 12/6/1924); Azerbaijan (*Aucher-Eloy* 4946); Russia: Kuban (*Busch et al.* 367), Altai (*Shishkin et al.* 17/6/1931); ); Iran (*Rechinger* 4314, *Edmondson* 1282); Afghanistan (*Grey-Wilson & Hewer* 1197); Pakistan (*Lace* 3716); Kyrgyzstan (*Borosova* 27); Turkmenistan (*Litwinov* 1655); Kazakhstan (*Androssov* 3770a); Tajikistan (*Nikitin & Soskov* 430); Uzbekistan (*Bukinitsch* 71); China (*Roborowski* 146); Mongolia/Songaria (*Potanin* 1876).

##### Notes.

Although we have not seen the type of *Convolvulus tshegemensis* we are confident in treating it as a synonym of *Convolvulus lineatus* as the diagnosis does not distinguish it from *Convolvulus lineatus* in any way.

Apart from *Convolvulus arvensis*, *Convolvulus lineatus* is the most widely distributed species of *Convolvulus*. Preliminary molecular studies suggest that this species is polyphyletic with significant genetic variation but until now no significant morphological variation has been discerned so it is here treated as a single species.

#### 157 × 158a. Convolvulus × cyprius

Taxon classificationPlantaeSolanalesConvolvulaceae

Boiss., Fl. Orient. [Boissier] 4: 93. 1875. (Boissier 1875b: 93).

Convolvulus lineatus var. angustifolius Kotschy ex Sa’ad, Meded. Bot. Mus. Herb. Rijks Univ. Utrecht 281: 130. 1967. (Sa’ad 1967: 130). Type. Based on *Convolvulus × cyprius* Boiss.

##### Type.

CYPRUS, Lania, *Kotschy* 627 (holotype G; isotype W!)

##### Distinguishing features.

Similar to *Convolvulus lineatus* but with somewhat longer stems, linear-lanceolate leaves < 5 mm wide and the flowers clustered at the apex of the stem. [Meikle 1985: 1171]

##### Distribution.

Endemic to Cyprus: Cape Gata (*Meikle 2908*, *Davis 3573K*).

##### Notes.

This taxon represents Convolvulus lineatus × oleifolius. Convolvulus × cyprius is reported to be one of a series of interconnecting plants between *Convolvulus lineatus* and *Convolvulus oleifolius* in the type locality (Meikle 1985: 1171).

#### 157. Convolvulus oleifolius

Taxon classificationPlantaeSolanalesConvolvulaceae

Desr., Encycl. [Lamarck et al.] 3: 552. 1792. (Desrousseaux 1792: 552)

Figure 20, t. 43–51

##### Type.

“Levant”, plant cultivated in Paris (holotype P [Herb. Lam.]).

##### Description.

60 cm, the old growth woody, young shoots herbaceous, flower-bearing; vegetative parts grey-sericeous. Leaves sessile, (1-)2.5–6 × (0.1-) 0.4–0.8 cm, linear, oblong or oblanceolate, obtuse to acute, entire, the base long-attenuate. Flowers in terminal diachasial clusters, sometimes with single flowers or few-flowered, diachasia borne on peduncles up to 3 cm long from the axils of the uppermost leaves; bracts resembling the leaves but smaller, always linear-oblong; bracteoles 8–10 × 1–1.5 mm, linear; pedicels 0–10 mm; outer sepals 6–9 × 2.5–5 mm, ovate, shortly acuminate to an obtuse apex, villous; inner sepals broader with scarious margins; corolla 2–2.5 cm long, pink, very shallowly lobed, the midpetaline bands appressed pilose, brown, terminating in a tooth; ovary pilose, style pilose, divided c. 3 mm above base, the stigmas 5 mm. Capsule pilose; seeds, densely pubescent. [Sa’ad 1967: 131; Meikle 1985: 1167, 1169 (plate); Pignatti 1982: 387; Strid and Strid 2009: 402–403 (plate)]

##### Notes.

*Convolvulus oleifolius* is a variable species and we recognise three varieties:

#### 157a. Convolvulus oleifolius var. oleifolius

Taxon classificationPlantaeSolanalesConvolvulaceae

Convolvulus oleifolius var.β Desr. in Lamarck, Encycl. 3: 552. 1792. (Desrousseaux 1792: 552). Type. without data (P-JU).
Convolvulus linearis Curtis, Bot. Mag. t.289. 1795, nom. illeg., non *Convolvulus linearis* Lam. (1779). (Curtis 1795: t. 289) Type. Icon. t. 289 in Curtis, Bot.Mag. (1795).
Convolvulus oleifolius var. pauciflorus Feinbrun, Palestine J. Bot., Jerusalem Ser. 2: 97.1940. (Eig and Feinbrun 1940: 97). Type. PALESTINE/ISRAEL, *Eig et al.* s.n., four syntypes given.
Convolvulus oleifolius var. scopulorum Rech.f., Akad. Wiss. Wien, Math.-Naturwiss. Kl., Denkschr. 105: 107. 1944. (Rechinger 1944: 107). Type. GREECE, Crete, Kissamos, Grabusa Dimitraki Island, *Rechinger* 12165 (holotype W).
Convolvulus oleifolius subsp. scopulorum (Rech.f.) Greuter & Pleger, Willdenowia 13: 55. 1983. (Greuter et al. 1983: 55). Type. Based on Convolvulus oleifolius var. scopulorum Rech.f.

##### Distingushing features.

Very variable in habit but commonly ascending with stems > 10 cm in length and branches herbaceous. Leaves very variable but usually 2.5–6 × 0.4–0.8 cm.

##### Distribution.

Eastern Mediterranean: Malta (*Wright* s.n.); Greece (*Aucher-Eloy* 1388, *Townsend* 71/158); Crete (*Rechinger* 12165); Cyprus (*Merton* 530, *Davis* 3286K); Aegean Islands (*Boratyńska et al.* 88); Turkey (*Dudley* 35421); Libya (*Pampannini & Pichi-Sermolli* 6220, *Guichard* Lib/558); Egypt (*Wanntorp & Sjödin* 2390); Palestine/Israel (*Grierson* 4/1970).

#### 157b. Convolvulus oleifolius var. pumilus

Taxon classificationPlantaeSolanalesConvolvulaceae

Pamp., Arch. Bot. (Forlì) 12: 41.1936. (Pampanini 1936a: 41).

##### Type.

LIBYA, Cyrenaica, *Pampanini* s.n. (holotype FI).

##### Distinguishing features.

Distinguished by its prostrate, pulvinate habit – it does not normally exceed 10 cm in height. The leaves are oblanceolate, small, 1–2 × 0.4–0.6 cm.

##### Distribution.

Appears to be a maritime ecotype and is reported from Libya and Cyprus (*Seligman* s.n. in Casey 1656, *Kennedy* 1783).

#### 157c. Convolvulus oleifolius var. angustifolius

Taxon classificationPlantaeSolanalesConvolvulaceae

Bég. & A.Vacc., Sp. Rare Fl. Libia 2. 1912. (Béguinot and Vaccari 1912: 2).

Convolvulus oleifolius var. deserti Pamp., Arch. Bot. (Forlì) 12: 40.1936. (Pampanini 1936a: 40). Type. LIBYA, Cyrenaica, *Pampanini* s.n. (holotype FI!).

##### Type.

LIBYA, Cyrenaica, *Vaccari* 437 (holotype FI!)

##### Distinguishing features.

Distinguished by its rigid, more or less fastigiate habit, the branches all woody. Leaves are linear, 1–1.5 × 0.2 cm and mostly absent below. The hairs on the bracteoles and calyx are shorter than in the type.

##### Distribution.

Reported from Libya, Cyprus and Turkey (*Syngrassides* 381, *Seligman* s.n. in *Casey* 1657).

#### 158. Convolvulus argyrothamnos

Taxon classificationPlantaeSolanalesConvolvulaceae

Greuter, Bauhinia 3: 251. 1967. (Greuter 1967: 251).

##### Type.

GREECE, Crete, Ierápetra. *Greuter* 7802 (holotype Hb. Greuter (B or PAL?); isotypes E00288017!, G, LD, W!).

##### Distinguishing features.

Undershrub clearly related to *Convolvulus oleifolius* and distinguished by its unusual habit. It is a pendulous plant, strongly acrotonous in its branching with the leaves in fasciculate bunches. No floral differences from *Convolvulus oleifolius* are known.

##### Description.

Endemic to Crete.

##### Notes.

It is impossible to confirm whether this is a distinct relict species or some peculiar adaptation of *Convolvulus oleifolius* to the cliff habitat. Its population is apparently restricted to a small number of plants on one or two limestone cliffs.

#### 159. Convolvulus mazicum

Taxon classificationPlantaeSolanalesConvolvulaceae

Emb. & Maire, Mat. Fl. Maroc. 21-22: 43 (23). 1930 [1929]. (Emberger and Maire 1929: 43 (23)).

Figure 21, t. 11–17

Convolvulus cantabrica var. mazicum (Emb. & Maire) Font Quer, Mem. Real Acad. Vi. Barcelona 22(18): 15. 1931. (Font Quer 1931: 15). Type. Based on *Convolvulus mazicum* Emb. & Maire
Convolvulus cantabrica subsp. mazicum (Emb. & Maire) Maire, Bull. Soc. Hist. Nat. Afrique N. 28: 370. 1937. (Maire 1937: 370). Type. Based on *Convolvulus mazicum* Emb. & Maire

##### Type.

MOROCCO, Middle Atlas, J. Guebb-er-Rahal, *Emberger* s.n. (syntypes RAB078146!, P00417706!, MPU 006033, MPU006032, ?AL).

##### Description.

A small cushion plant, 7–13 cm in diameter with a stout woody taproot, stem sericeous. Leaves sessile, 2–3 × 0.2–0.4 cm, linear to linear-oblanceolate, acute, entire, base attenuate, grey sericeous-pubescent on the lower surface and margins of upper surface, glabrous on central area. Flowers solitary or paired, sessile or on very short terminal peduncles up to 10 mm long; bracteoles linear, 3 × 0.5 mm, pedicels 2 mm; outer sepals 5–6 × 2 mm, lanceolate, obtuse, pilose; inner sepals ovate, c. 4 mm wide, membranous, abruptly narrowed to a green, pilose mucro; corolla 1.6–1.7 cm long, white, lobed with red, pubescent, midpetaline bands terminating in a broadly triangular lobe; ovary conical, pilose at apex; style pilose, divided c. 4 mm above base, stigmas 3 mm. Capsule and seeds not seen. [Sa’ad 1967: 146]

##### Distribution.

Morocco (*Jury et al.* 17622, *Balls* 3086, *Güemes et al.* 1577). 2000–3000 m on limestone rocks, somewhat disjunct in distribution in the High Atlas, Middle Atlas and the Rif mountain ranges.

##### Notes.

Dwarf plants of *Convolvulus lineatus* are similar but have longer sepals and corollas and the upper surface of the leaves are uniformly sericeous.

#### 160. Convolvulus phrygius

Taxon classificationPlantaeSolanalesConvolvulaceae

Bornm., Repert Spec. Nov. Regni Veg. 5: 168. 1908. (Bornmüller 1908: 168).

Figure 21, t. 26–32

Convolvulus pulvinatus Sa’ad, Meded. Bot. Mus. Herb. Rijks Univ. Utrecht 281: 148. 1967. (Sa’ad 1967: 148). Type. TURKEY, Eskisehir, *Scheibe* 1075 (holotype B!).

##### Type.

TURKEY, Eski-Scheher, *Warburg & Endlich* 515 (holotype B!).

##### Description.

Compact cushion-forming undershrub with thick woody taproot, branched at base with short woody prostrate to ascending branches to c. 15 cm, young stems sericeous. Leaves sessile, 1.2–2 × 0.3–0.4 cm, linear-oblanceolate, acute, attenuate at the base, sericeous and with some spreading hairs. Flowers solitary, axillary, shortly pedunculate but becoming crowded, sessile and subcapitate towards the branch tips; peduncles 0–5 mm, pilose; bracts leaf-like; bracteoles similar but shorter; sepals 8–11 × 3 mm, oblong, acute to apiculate, concave, adpressed pilose, not bicoloured; corolla 1.7–2.3 cm long, white with distinct pink, pubescent midpetaline bands, unlobed; ovary pilose; style glabrous, divided c. 4 mm above base; stigmas 4 mm. Capsule and seeds unknown.

##### Distribution.

Endemic to Turkey (*Rechinger* 61014, *Fitz & Spitzenberger* 721, *Sorger* s.n. [28/5/1964]).

##### Notes.

Could be confused with Convolvulus boissieri subsp. compactus but the 1-nerved, near linear leaves are distinct.

Although Parris (1978: 209) treated *Convolvulus phrygius* and *Convolvulus pulvinatus* as distinct species, they were collected at the same place and the distinctions in measurements are minor. The corollas are similar in size and the sepals are only about 2 mm different in length. There is no good reason to treat them as separate species.

#### 161. Convolvulus libanoticus

Taxon classificationPlantaeSolanalesConvolvulaceae

Boiss., Diagn. Pl. Orient. 11: 82. 1849. (Boissier 1849: 82).

Figure 21, t. 1–10

Convolvulus radicosus Heldr. & Sart., Diagn. Pl. Orient., ser. 2, 3: 124.. 1856. (Boissier 1856: 124). Type. GREECE, Keyllenes, *Heldreich* s.n. [21/6/1848] (B†; neotype *Heldreich* 961 (B, designated by Sa’ad 1967: 143); isoneotypes C, CAIM, E, G, K, JE, HAL, OXF, P, STU, W).
Convolvulus cantabrica subsp. radicosus (Heldr. & Sart.) Maire, Cat. Pl. Maroc 3: 588. 1934. (Jahandiez and Maire 1934: 588). Type. Based on *Convolvulus radicosus* Heldr. & Sart.

##### Type.

LEBANON, *Boissier* s.n. (lectotype G, designated by Sa’ad 1967: 143).

##### Description.

Cushion plant with branched woody rootstock, herbaceous flowering stems to 5 cm, vegetative parts adpressed-pilose to subglabrous. Leaves sessile, 1–2.5(-6) × 0.2–0.4 cm, linear to oblanceolate, obtuse to acute, entire, base attenuate; glabrous or very sparsely hairy on the upper surface. Flowers in small terminal diachasial cymes of up to 5 flowers with solitary axillary flowers or axillary cymes; bracts leaf-like; peduncles 0–3 cm; bracteoles 3 × 0.5 mm; sepals 5–8 × 1.5–2 mm, oblong-obovate, scarious with distinct, acute, green triangular apex, inner sepals scarious; corolla 1.2–1.5 cm, white or pink, unlobed, midpetaline bands pilose, terminating in a tooth; ovary pilose; style pubescent, divided c. 2 mm above the base; stigmas c. 4 mm. Capsule pubescent, seeds puberulent. [Sa’ad 1967: 143; Tohmé and Tohmé 2007: 215 (photo); Strid 1991: 17–18]

##### Distribution.

Lebanon (*Bornmüller* 1107, *Gombault* 4483), Syria (*Kotschy* 54); Turkey (*Davis* 13514); Greece (*Stamatiadou* 6559); Crete (?). Disjunct on mountains 1600–2700 m.

#### 162. Convolvulus assyricus

Taxon classificationPlantaeSolanalesConvolvulaceae

Griseb., Spic. Fl. Rumel. 2: 75. 1844. (Grisebach 1844: 75).

Figure 22, t. 39–47

Convolvulus strigulosus Boiss., Diagn. Pl. Orient. 11: 83. 1849. (Boissier 1849: 83). Type. TURKEY, between Ankara and Tkat, *Aucher-Eloy* 4939 (holotype G).

##### Type.

TURKEY, *Donietti* s.n. (holotype GOET).

##### Description.

Perennial cushion-forming plant with woody taproot and short spreading woody branches, the herbaceous parts densely covered with weakly appressed to spreading, stiff hairs. Leaves sessile, dimorphic, small obovate-spathulate leaves 3–5 mm long with a broad base mixed with linear to oblanceolate, acute entire leaves, 1–2 × 0.2–0.5 cm, with an attenuate base. Flowers terminal, solitary (?always), sessile; bracteoles 5–7 mm, linear; outer sepals 3 × 1.5 mm, obovate, cuspidate, whitish, pilose; inner sepals 3 × 2 mm, broadly obovate, cuspidate, corolla pink, 2–2.2 cm, unlobed, midpetaline bands pilose; stamens very unequal; ovary pilose, style thinly pilose, divided c. 3 mm above base; stigmas 6–10 mm long; capsule pilose; seeds puberulent. [Sa’ad 1967: 138]

**Figure 22.**
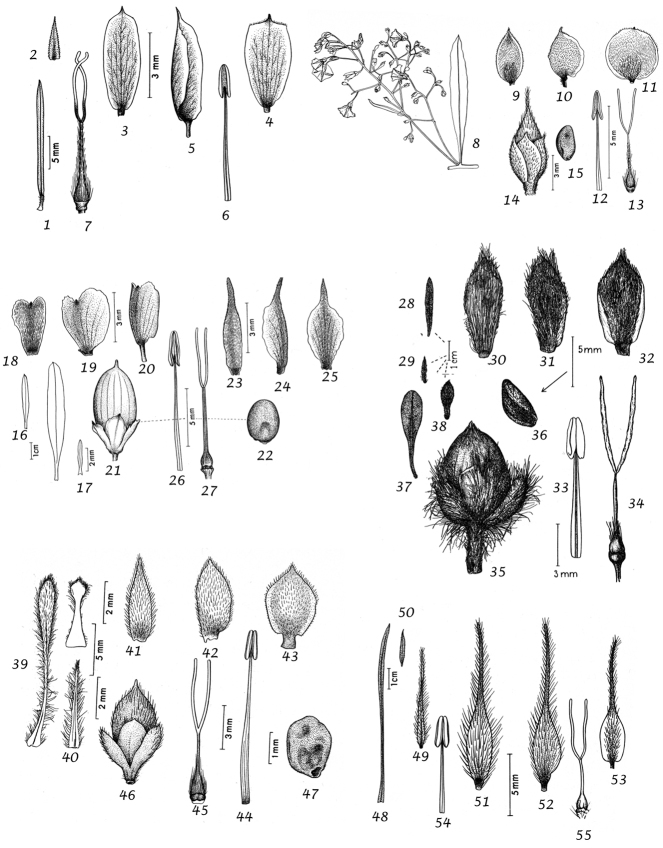
**1–7** *Convolvulus scoparius* **1** leaf **2** bracteole **3** outer sepal **4** middle sepal **5** inner sepal **6** stamen **7** ovary and style. From *Bourgeau* 1427 (C) **8–15** *Convolvulus floridus* **8** habit **9** outer sepal **10** middle sepal **11** inner sepal **12** stamen **13** ovary and style **14** capsule **15** seed. From *sin coll.* ex Tenerife (W) **16–22** Convolvulus dorycnium subsp. dorycnium **16** leaves **17** bracteole **18** outer sepal **19** middle sepal **20** inner sepal **21** capsule **22** seed **16–17** from *Leonis* s.n. (W) **18–20** from *Heldreich* 1264 (STU) **21–22** from *Heldreich* 36a (W) **23–27** Convolvulus dorycnium subsp. oxysepalus **23** outer sepal **24** middle sepal **25** inner sepal **26** stamen **27** ovary and style. From *Handel-Mazzetti* 1948 (W) **28–36** Convolvulus cneorum var. cneorum **28** leaf **29** bract **30** outer sepal **31** middle sepal **32** inner sepal **33** stamen **34** ovary and style **35** capsule **36** seed. From *Todaro* s.n. (U) **37–38** Convolvulus cneorum var. latifolius **37** leaf **38** bract. From *sin coll*. (W) **39–47** *Convolvulus assyricus* **39** leaves **40** bracteole **41** outer sepal **42** middle sepal **43** inner sepal **44** stamen **45** ovary and style **46** capsule **47** seed **39–45** from *Stainton* 5109 (E) **46–47** from *Balansa* 973 (W) **48–55** *Convolvulus lanuginosus* **48** leaf **49** bract **50** bracteole **51** outer sepal **52** middle sepal **53** inner sepal **54** stamen **55** ovary and style. From *Stud. Biol.* 912/1962 (U).

##### Distribution.

Endemic to Turkey (*Stainton & Henderson* 5100, *Davis* 21910, *Kotschy* 179, *Manissadjian* 137).

##### Notes.

A very distinctive species on account of its indumentum, dimorphic leaves, tiny calyx, pink corolla and unusually long stigmas.

#### 163. Convolvulus cataonicus

Taxon classificationPlantaeSolanalesConvolvulaceae

Boiss. & Hausskn., Pl. Orient. Nov. (dec. prim.) 1: 5. 1875. (Boissier 1875a: 5).

Figure 21, t. 18–25

Convolvulus huber-morathii P.H.Davis, Notes Roy. Bot. Gard. Edinburgh 24: 24. 1962. (Davis 1962: 24). Type. TURKEY, *Stainton & Henderson* 5471 (holotype E00285414!; isotype K!).
Convolvulus abdallahi Sa’ad, Meded. Bot. Mus. Herb. Rijks Univ. Utrecht 281: 114. 1967. (Sa’ad 1967: 114). Type. TURKEY, Kastamonu, *Davis* 21640 (holotype K!; isotype E!).

##### Type.

TURKEY, Beryt Dagh, *Haussknecht* s.n. (holotype G; isotype W!).

##### Description.

Woody based perennial, branched at base with a thick, woody tap root, somewhat cushion-forming with short spreading woody branches and ascending herbaceous stems to c. 20 cm, adpressed pilose with some spreading hairs. Leaves mostly basal, sometimes folded, sessile, 1–6 × 0.2–0.3 cm, linear or linear-oblanceolate, acute, entire with long tapering base; lower surface and margins pilose, upper surface glabrous except at margins. Flowers in few-flowered terminal heads, occasionally also with solitary sessile or very shortly pedunculate heads from the axils of the uppermost leaves; bracts 10–20 × 1–2 cm, linear, acute; peduncles 0–2 cm; bracteoles 3–6 mm, linear; pedicels 0–1 mm; sepals 6–10 × 2 mm, ovate, the scarious base abruptly narrowed to a green caudate apex 2–3 mm long, shortly pilose; inner sepals with broad, scarious, glabrous margins; corolla 1.5–2 cm long, white, unlobed, midpetaline bands pilose, terminating in a tooth; ovary pubescent; style divided c. 4 mm above base, stigmas 3 mm. Capsule pilose; seeds not seen. [Sa’ad 1967: 139; Parris 1978: 211]

##### Distribution.

A rare species of central and eastern Turkey (*Buchner* 16/8/1982).

##### Notes.

Similar to *Convolvulus calvertii* but distinguished by the calyx bicoloured, the apical part green.

#### 164. Convolvulus carduchorum

Taxon classificationPlantaeSolanalesConvolvulaceae

P.H.Davis, Notes Roy. Bot. Gard. Edinburgh 24: 24. 1962. (Davis 1962: 24).

Figure 21, t. 33–39

Convolvulus glabrescens P.H.Davis & Hub.-Mor., Notes Roy. Bot. Gard. Edinburgh 24: 27. 1962. (Davis 1962: 24). Type. TURKEY, Tunceli, pass between Pülümür and Mutu, Huber-Morath 15657 (BASBG?, not seen, photo E!).
Convolvulus anatolicus Sa’ad, Meded. Bot. Mus. Herb. Rijks Univ. Utrecht 281: 135. 1967. (Sa’ad 1967: 135). Type. TURKEY, Malatya-Kjacta, Kurdistania, *Handel-Mazetti* 2226 (holotype W).
Convolvulus orophilus Sa’ad, Meded. Bot. Mus. Herb. Rijks Univ. Utrecht 281: 147. 1967. (Sa’ad 1967: 147). Type. TURKEY, Hasarbaba Dagh near Goldschik Lake, *Handel-Mazzetti* 2572 (holotype W).

##### Type.

TURKEY, *Davis & Polunin* 23382 (holotype E00285413!; isotypes BM001014567!, K!).

##### Distinguishing features.

Woody based perennial similar to *Convolvulus cataonicus* but leaves, bracts and sepals adpressed pilose to subglabrous, sepals gradually acuminate and ovary and style glabrous or very thinly pilose. [Parris 1978: 211]

##### Distribution.

Endemic to Turkey and apparently rare.

##### Notes.

This species may prove only to be a variety or subspecies of *Convolvulus cataonicus*. It is readily distinguished by the near absence of hairs on the leaves, sepals and ovary, but at least one intermediate is known. *Brant & Strangeways* 1840 combines absence of hairs with a sepal shape similar to that of *Convolvulus cataonicus*.

#### 165. Convolvulus lanuginosus

Taxon classificationPlantaeSolanalesConvolvulaceae

Desr., Encycl. [Lamarck et al.] 3: 551. 1792. (Desrousseaux 1792: 551).

Figure 22, t. 48–55

Convolvulus argenteus Pourr., Hist. & Mém. Acad. Roy. Sci. Toulouse 316. 1788, nom. illeg, non *Convolvulus argenteus* Lam. (1778). (Pourret 1788: 316). Type. SPAIN, Montserrat, *Salvador & de Jussieu* (not found at P).
Convolvulus capitatus Cav., Icon. 2: 72. 1793, nom. illeg., non *Convolvulus capitatus* Desr. (1789). (Cavanilles 1793: 72). Type. SPAIN, (“in monte Bañeres” (syntype MA 93917), “in montibus Monduber” (syntype MA 93915), “prope Gilet” (syntype MA 93916).
Convolvulus saxatilis Vahl, Symb. Bot. 3: 33. 1794. (Vahl 1794: 33). Type. SPAIN, *M. Barnadez* s.n. (holotype C!).
Convolvulus saxatilis var. sericeus Boiss., Voy. Bot. Espagne 2: 416. 1841. (Boissier 1841: 416). Type. SPAIN, not specified.
Convolvulus saxatilis var. villosus Boiss., Voy. Bot. Espagne 2: 416. 1841. (Boissier 1841: 416). Type. SPAIN, not specified.
Convolvulus lanuginosus var. canescens Choisy, Prodr. [A.P. de Candolle] 9: 401. 1845. (Choisy 1845: 401). Type. Based on *Convolvulus capitatus* Cav.
Convolvulus lanuginosus var. sericeus (Boiss.) Willk., Prodr, Fl. Hispan. 2: 516. 1870. (Willkomm and Lange 1870: 516). Type. Based on Convolvulus saxatilis var. sericeus Boiss.
Convolvulus lanuginosus var. villosus (Boiss.) Sa’ad, Meded. Bot. Mus. Herb. Rijks Univ. Utrecht 281: 119. 1967. (Sa’ad. 1967: 119). Type. Based on Convolvulus saxatilis var. villosus Boiss.
Convolvulus lanuginosus subsp. sericeus (Boiss.) Rivas Goday & Rivas Mart., Anales Inst. Bot. Cavanilles 25: 158. 1969 [1967]. (Rivas-Goday and Rivas-Martinez 1969: 158). Type. Based on Convolvulus saxatilis var. sericeus Boiss.

##### Type.

Without locality (lectotype P [Herb. Lam.], designated by Sa’ad 1967: 118).

##### Description.

Woody based perennial, branched at base with erect stems to 40 cm; vegetative parts appressed pilose to villous with spreading hairs. Basal leaves 1.5–6 × 1–4 cm. oblanceolate with a long attenuate base; stem leaves sessile, 2.5–5 × 0.1–0.3 cm. linear or oblong, acute or obtuse, entire, somewhat narrowed at the base. Flowers in many-flowered terminal heads; bracts 7–22 × 2–6 mm, oblong-elliptic or ovate, acute; bracteoles 7–8 × 0.5 mm, linear; sepals 8–9 × 2.5–3 mm, narrowly ovate and abruptly narrowed into a long fine point, margin somewhat scarious; corolla 1.5–2. 8 cm long, white or pink with darker midpetaline bands, weakly lobed with broadly triangular lobes; midpetaline bands dark, pilose; ovary glabrous; style glabrous, divided c. 4 mm above base; stigmas c. 4 mm. Capsule glabrous, seeds smooth, pubescent. [Sa’ad 1967: 117; Silvestre 2012: 267; Polunin and Smythies 1973 (plate 38)]

##### Distribution.

France (*Billot* 3157, *Roux* 1877); Spain (*Jerónimo* 4946, *Miles at al*. 311, 506, *Bourgeau* 332, 1297); Morocco (?).

##### Notes.

A somewhat variable plant, the type adpressed pubescent, but villous plants with spreading indumentum, e.g. *Font-Quer & Rothmaler* 12/1935 and *Simpson* 51450 are frequent. Some specimens are much reduced and cushion-like, mostly from mountains in Spain (*Bourgeau* 334, *Lofthouse* s.n. [21/6/1926]).

#### 166. Convolvulus cneorum

Taxon classificationPlantaeSolanalesConvolvulaceae

L., Sp. Pl. 1: 157. 1753. (Linnaeus 1753: 157).

Figure 22, t. 28–38

Convolvulus argenteus Desr., Encycl. [Lamarck et al.] 3: 552, 1789. (Desrousseaux 1789: 552) Type. Cultivated specimen (P [Herb. Lam.]) said (erroneously) to be of Cretan origin.

##### Type.

Plate “Convolvulus Creticus rectus s. *Dorycnium quorundam* Ponae” in Morison (1680: 11, sect. 1, plate 3, f.1), lectotype, designated by Sa’ad 1967: 126).

##### Description.

Perennial undershrub to c. 30 cm, the flowering shoots herbaceous with all vegetative parts densely grey-sericeous. Leaves sessile, 2–3.5(-5) × 0.3–0.8(-1.2) cm, oblong to oblanceolate, acute or obtuse, entire, attenuate at base. Flowers in a dense terminal cymose cluster, sometimes with one or two flowers in the axils of bracts immediately below the cluster, borne on peduncles 1–2(-4) cm long; bracts as for leaves but smaller; bracteoles 9–14 × 1 mm, linear, acuminate and apiculate; pedicels 0–3 mm; sepals 7–9 × 2–3 mm, oblong-oblanceolate, acute, densely pilose, the inner sepals broader (c. 3.5 mm) with scarious margins; corolla 2–7 cm, white, unlobed, the midpetaline bands densely pilose; ovary pilose; style glabrous or pilose at base, divided c. 3 mm above the base; stigmas 5 mm. Capsule pilose; seeds pubescent. [Sa’ad 1967: 126; Pignatti 1982: 387]

##### Notes.

We recognise two varieties:

#### 166a. Convolvulus cneorum var. cneorum

Taxon classificationPlantaeSolanalesConvolvulaceae

Convolvulus argenteus Salisb., Prodr. Stirp. Chap. Allerton 125. 1796, illegitimate superfluous name for *Convolvulus cneorum* L. (Salisbury 1796: 125). Type. Based on *Convolvulus cneorum* L.

##### Distinguishing features.

Plants representative of the type subspecies have linear-oblong leaves and are apparently restricted to Sicily.

##### Distribution.

Endemic to Sicily (*Todaro* s.n., *Heldreich* s.n. [15/5/1840]).

#### 166b. Convolvulus cneorum var. latifolius

Taxon classificationPlantaeSolanalesConvolvulaceae

Rchb., Icon. Fl. Germ. Helv. 18: 83. 1858. (Reichenbach 1858: 83).

Convolvulus cneorum subsp. latifolius (Rchb.) Sa’ad, Meded. Bot. Mus. Herb. Rijks Univ. Utrecht 281: 127. 1967. (Sa’ad 1967: 127). Type. Based on Convolvulus cneorum var. latifolius Rchb.

##### Type.

CROATIA, *Petter* 48 (holotype W).

##### Distinguishing features.

Distinguished by the oblanceolate leaves.

##### Distribution.

Much the most common variety: Croatia (*Pichler* s.n. [4/6/1868], *Berger* 12/5/1910); Albania (fide Greuter et al. 1983: 4); Italy (*Bornmüller* 48, *Guadagno* s.n. [10/6/1907]); Sicily (*Prior* s.n. [4/1845], *Gabriel* s.n. [19/4/1874], *Bieringer* 99); Tunisia (fide Pottier-Alapette 1981: 716).

##### Notes.

Var. latifolius is not separated from var. cneorum geographically and does not merit the subspecific status given it by Sa’ad (1967: 127).

#### 167. Convolvulus dorycnium

Taxon classificationPlantaeSolanalesConvolvulaceae

L., Syst. Nat. ed. 10, 2: 923. 1759. (Linnaeus 1759: 923).

##### Type.

“Oriente,” *Hasselquist* s.n. (lectotype LINN 218.50, designated by Sa’ad 1967: 90).

##### Description.

Erect, perennial, much-branched undershrub from a woody rootstock to 1 m, the branches rigid and woody, adpressed pubescent. Leaves sessile, 1.5–6 × 0.2–2 cm, narrowly oblong to narrowly oblanceolate, acute, entire, attenuate at base, villous, the stem leaves smaller than those at the base. Flowers in a large, nearly leafless, branched terminal inflorescence composed of axillary cymes of 1–3(-7)-flowered diachasial cymes, the flowers appearing solitary; branches stout and woody; bracteoles 2–3 mm, linear; pedicels 0–4 mm; sepals 2.5–5 × 2–5 mm, very variable in form, oblong-elliptic to obovate, acuminate to emarginate and mucronate, adpressed pubescent, the inner sepals broader than the outer sepals; corolla 1.2–1.7 cm, pink (very rarely pure white), the midpetaline bands pilose; ovary glabrous, style glabrous, divided c. 5 mm above base, often persistent in fruit, stigmas c. 3 mm; capsule glabrous, seeds subglobose, puberulent. [Sa’ad 1967: 90; Meikle 1985: 1166; Siddiqi 1977: 13 (Figure 5); Strid and Strid 2010: 2–3 (plate)]

##### Notes.

We recognise three subspecies but intermediates are quite frequently found: *Reino Alava* 6986 from Turkey, for example, is intermediate between subsp. dorycnium and subsp. oxysepalus.

#### 167a. Convolvulus dorycnium subsp. dorycnium

Taxon classificationPlantaeSolanalesConvolvulaceae

Figure 22, t. 16–22

##### Distinguishing features.

Branches rigid, woody; stem leaves few; sepals broadly obovate, truncate and mucronate.

##### Distribution.

Tunisia (*Cosson et al.* s.n. [10/6/1883]); Greece (*Aitchley* 257, *Halaczy* s.n. [5/7/1888], *Strid* 31888); Crete (*Verdcourt* 4153); Cyprus (*Economides* 1188); Turkey (*Balls & Gourlay* 1194); Aegean Islands (*Rechinger* 707), Lebanon (*Breidy & Khairallah* 626), Syria (*Kotschy* 375), Palestine (*Dinsmore* 10187), Jordan (*Dinsmore* 12187); Egypt (?).

#### 167b. Convolvulus dorycnium subsp. oxysepalus

Taxon classificationPlantaeSolanalesConvolvulaceae

(Boiss.) Rech.f., Österr. Bot. Z. 94: 170. 1948. (Rechinger 1948: 170).

Figure 22, t. 23–27

Convolvulus dorycnium var. oxysepalus Boiss., Fl. Orient. [Boissier] 4: 92. 1875. (Boissier 1875b: 92). Type. PALESTINE/ISRAEL, Tiberias, *Boissier* s.n. (lectotype G, designated by Sa’ad 1967: 9); isolectotypes K!, P!).

##### Type.

Based on Convolvulus dorycnium var. oxysepalus Boiss.

##### Distinguishing features.

Branches rigid, woody; sepals oblong-elliptic to lanceolate, acuminate. [Nowroozi 2002: 102 (map); Tohmé and Tohmé 2007: 214 (photo)]

##### Distribution.

Syria (*Haradjian* 630, *Ehrenberg* 133, *Haussknecht* s.n. [15/6/1865]); Palestine/Israel (*Aucher-Eloy* 1397); Iran (*Kotschy* 436, *Hewer* 3997, *Bornmüller* 3883, *Bokhari & Edmondson* 2087); Afghanistan (*Gibbons* 457). This subspecies has a more eastern distribution than subsp. dorycnium.

#### 167c. Convolvulus dorycnium subsp. subhirsutus

Taxon classificationPlantaeSolanalesConvolvulaceae

(Regel & Schmalh.) Sa’ad, Meded. Bot. Mus. Herb. Rijks Univ. Utrecht 281: 91. 1967. (Sa’ad 1967: 91).

Convolvulus subhirsutus Regel & Schmalh., Trudy Imp. S.-Peterburgsk. Bot. Sada 6: 339. 1879. (Regel 1879: 339). Type. KAZAKHSTAN, *Regel* 270 (lectotype LE!, designated by Sa’ad 1967: 94; isolectotypes E!, K!, LE!).

##### Type.

Based on *Convolvulus subhirsutus* Regel & Schmalh.

##### Distinguishing features.

Stems leafy and more or less herbaceous; sepals narrowly oblong-obovate, more or less abruptly narrowed to a mucronate apex. Nowroozi 2002: 49 (plate), 102 (map); Breckle and Rafiqpoor 2010: 415 (photo).

##### Distribution.

Apparently abundant in Central Asia: Northeastern Iran (*Merton* 3955, *Andersen & Petersen* 308, *Sabetí* 977); Afghanistan (*Grey-Wilson & Hewer* 848, *Podlech* 10760, *Hedge & Wendelbo* 3618); Uzbekistan (*Vassilejeva & Vassilczenko* 4837, *Sintenis* 384, *Bornmüller* 73); Turkmenistan (*Capus* 954, *Djilenko* 723); Kirgizstan (*Popov* 288, *Bobrov* 110); Kazakhstan (*Minkwitz* s.n. [23/6/1914], *Fedshenko* s.n. [8/8/1902]); Tajikistan (*Linczevski & Masslennikova* 737, *Ovczinnikov* 394).

#### 168. Convolvulus tschimganicus

Taxon classificationPlantaeSolanalesConvolvulaceae

Popov & Vved., Byull. Sredne-Aziatsk. Gosud. Univ. 15 (Suppl.): 31. 1927. (Popov and Vvedensky (1927: 31).

##### Type.

Western Tian Shan, *Popov* in Fl. As. Med. Fasc. 12, 288 (holotype TAK; isotypes E!, G, K!, LE!, P!, W!).

##### Distinguishing features.

As rightly noted by Popov and Vvedensky (1927: 31) *Convolvulus tschimganicus* is intermediate morphologically between *Convolvulus pilosellifolius* and Convolvulus dorycnium subsp. subhirsutus differing from the former by the distant flowers borne on nearly straight branches and the sepals 5–6 mm long, oblong-obovate, mucronate, not distinctly bicoloured and the larger corolla 1.6–1.8 cm long. It is distinguished from the latter by its more slender, less rigid habit, spreading indumentum and the stems unbranched except near the apex so creating a less divaricate inflorescence.

##### Distribution.

Endemic to Uzbekistan (*Korovin* s.n. [11/7/1928], *Khokhryekov* s.n. [8/8/1960]). Apparently rare.

##### Notes.

The status of this species is unclear and it may be of hybrid origin.

#### 169. Convolvulus caput-medusae

Taxon classificationPlantaeSolanalesConvolvulaceae

Lowe, Ann. Mag. Nat. Hist., ser. 3,6: 155. 1860. (Lowe 1860: 155).

##### Type.

CANARY ISLANDS, Fuerteventura, *Lowe* s.n. (holotype BM000056985!; isotype K!).

##### Description.

Hummock-forming undershrub 10 to 30 cm high, spreading horizontally to c. 60 cm; stems white-sericeous, stiff, spinescent when old. Leaves mostly alternate but sometimes clustered in short brachyblasts, sessile, 0.4–1.5 × 1.5–2.5 cm, oblanceolate to spathulate, acute, entire, cuneate at base, grey-sericeous with short, appressed scaly hairs. Flowers more or less sessile, axillary, 1 (-2) together; bracts similar to leaves but smaller, 5–6 × 1.5 mm; pedicels c. 1 mm, bracteoles 1.5 × 0.5 mm, oblong, acute; sepals all similar, 3–4 × 1.5–2 mm, broadly oblong-obovate, acute to mucronate, pubescent; corolla 8–10 mm long, white or pale pink, unlobed, midpetaline bands adpressed pilose, reddish; ovary with a bright orange disc, pilose; style pilose, divided c. 2.5 mm above base; stigmas 2.5 mm. Capsule reddish, densely pilose with the style persistent; seeds not seen. [Sa’ad 1967: 71 p.p.]

##### Distribution.

Canary Islands: Gran Canaria, Fuerteventura (*Carine & Durães* 158, *Bramwell & Humphries* 3095, *Kunkel* 13, *Beckett* 736; *Peck* 5). Globally rare.

#### 170. Convolvulus scoparius

Taxon classificationPlantaeSolanalesConvolvulaceae

L.f., Suppl. Pl. 135. 1782 [“1781”]. (Linnaeus 1782: 135).

Figure 22, t. 1–7

Breweria scoparia (L.f.) Lindl., Fl. Med. 400. 1838. (Lindley 1838: 400). Type. Based on *Convolvulus scoparius* L.f.
Rhodoxylon scoparium (L.f.) Raf., Fl. Tellur. 4: 80. 1838. (Rafinesque 1838: 80). Type. Based on *Convolvulus scoparius* L.f.
Rhodorhiza scoparia (L.f.) Webb, Bot. Reg. (Edwards et al.) 27(Misc.): 70. 1841. (Webb 1841: 70). Type. Based on *Convolvulus scoparius* L.f.
Convolvulus benehoavensis Bolle, Bonplandia 9: 54. 1861. (Bolle 1861: 54). Type. CANARY ISLANDS, Palma, *Bolle* s.n. (?B†), ex descr.

##### Type.

CANARY ISLANDS, Barrancas, *Masson* s.n. (holotype BM000829855!).

##### Description.

A branched unarmed undershrub to 2 m, vegetative parts glabrous or sparsely adpressed pilose. Leaves sessile, caducous, 0.5–4.5 × 0. 1 mm, filiform, acute, entire. Flowers (1-) 5–6 in pedunculate, terminal and axillary cymes; peduncles 2–7 (-11) mm, pubescent; bracteoles 2–3 × 1 mm, lanceolate, acuminate, base clasping, appressed to the calyx, pubescent; pedicels 3–7 mm, stout, pubescent; outer sepals 4–6 × 2–2.5 mm, broadly oblong, mucronate; inner sepals obovate, abruptly narrowed to a mucronate apex; corolla 1–1.2 cm long, white or pinkish, deeply lobed, midpetaline bands pilose; ovary pilose; style pilose, divided 3–4 mm above base, stigmas c. 3 mm. Capsule not seen but presumably pilose. [Sa’ad 1967: 106; Bramwell 2001: 262–263 (photo); Schönfelder and Schönfelder 1997: 174–175 (photo)]

##### Distribution.

Endemic to the Canary Islands: Tenerife, Gran Canaria, La Gomera, La Palma (?), El Hierro (*Bourgeau* 1427, *Bramwell* 1427, *Murray* s.n. [11/6/1899]).

#### 170 × 171. Convolvulus × despreauxii

Taxon classificationPlantaeSolanalesConvolvulaceae

A. Santos & Carine, Bot. J. Linn Soc.154: 200. 2007. (Carine et al. 2007: 200)

Rhodorrhiza virgata Webb & Berthel., Hist. Nat. Iles Canaries 3: 30, tab. 138. 1844. (Webb and Berthelot 1844: 30). Type. CANARY ISLANDS, *Despréaux* s.n. (holotype FI-Webb!).
Convolvulus scoparius var. virgatus (Webb & Berthel.) Choisy, Prodr. [A.P. de Candolle] 9: 404. 1845. (Choisy 1845: 404). Type. Based on *Rhodorrhiza virgata* Webb & Berthel.
Convolvulus floridus var. virgatus (Webb & Berthel.) Mendoza-Heuer, Cuad. Bot. Canaria 12: 24. 1971. (Mendoza-Heuer 1971: 24). Type. Based on *Rhodorrhiza virgata* Webb & Berthel.

##### Type.

Based on *Rhodorrhiza virgata* Webb & Berthel.

##### Distinguishing features.

The hybrid Convolvulus scoparius × floridus has leaves that are 1.12–4.86 mm wide, so intermediate in width between the parents. The inflorescence is generally compound unlike the simple inflorescences of *Convolvulus scoparius* but less branched than that of *Convolvulus floridus*.

##### Distribution.

La Gomera and Tenerife in the Canary Islands (*Bourgeau* 890, *Hernández & Pérez* s.n. [16/4/1976], *Carine & Santos Guerra* 202).

##### Notes.

This taxon represents *Convolvulus scoparius* × *Convolvulus floridus*.

#### 171. Convolvulus floridus

Taxon classificationPlantaeSolanalesConvolvulaceae

L.f., Suppl. Pl. 136. 1782 [“1781”]. (Linnaeus 1782: 136).

Figure 22, t. 8–15

Rhodoxylon floridum (L.f.) Raf., Fl. Tellur. 4: 80. 1838. (Rafinesque 1838: 80). Type. Based on *Convolvulus floridus* L.f.
Rhodorhiza florida (L.f.) Webb, Bot. Reg. (Edwards et al.) 27(misc.): 70. 1841. (Webb 1841: 70). Type. Based on *Convolvulus floridus* L.f.
Convolvulus floridus var. densiflorus Christ, Bot. Jahrb. Syst. 9: 125. 1888. (Christ 1888: 125). Type. CANARY ISLANDS, Punta de Tenerife, *Hillebert* s.n. *Rhodorhiza florida var. genuina* Pit., in J.-C. M. Pitard & L. Proust, Iles Canaries 281. 1908, nom. illeg., superfluous name for autonymic variety. (Pitard and Proust 1908: 281).
Rhodorhiza florida var. densiflora (Christ.) Pit., in J.-C. M. Pitard & L. Proust, Iles Canaries 281 (Pitard and Proust 1908: 281). Type. Based on Convolvulus floridus var. densiflorus Christ
Rhodorhiza florida var. angustifolia Pit., in J.-C. M. Pitard & L. Proust, Iles Canaries 281. 1908. (Pitard and Proust 1908: 281). Type. CANARY ISLANDS, various syntypes cited.
Convolvulus floridus var. angustifolius (Pit.) G.Kunkel, Cuad. Bot. Canaria 28: 59. 1977 [1976]. (Kunkel 1977: 59). Type. Based on Rhodorhiza florida var. angustifolia Pit.

##### Type.

CANARY ISLANDS, *Masson* s.n. (lectotype BM000829857!, designated by Sa’ad 1967: 110).

##### Description.

A branched unarmed shrub up to 4 m in height, vegetative parts shortly adpressed pubescent, somewhat glabresent on older parts. Leaves sessile, 2–14 × 0.5–2.6 cm, narrowly to broadly oblong, dark green, acute to obtuse, entire, base attenuate. Inflorescemce branches from the upper leaf axils forming a terminal, panicle-like inflorescence of branched cymes with primary branches to 7 cm long, the axes densely pubescent; bracteoles 1 × 0.5 mm, scale-like, caducous, pedicels 2–15 mm; outer sepals 4 × 2 mm, broadly lanceolate, apiculate, the margin ciliolate; inner sepals 4 × 4 mm, mucronate, elliptic or suborbicular, membranous; corolla 1.1–1.5 cm long, white, unlobed, midpetaline bands pilose; anthers exserted; ovary pilose; style pilose, divided c. 3 mm above base; stigmas c. 3 mm. Capsule acute, pilose, 1-seeded; seeds minutely hirsute. [Sa’ad 1967: 110; Bramwell and Bramwell 2001: 262 (photo); Schönfelder and Schönfelder 1997: 174–175 (photo)]

##### Distribution.

Endemic to the Canary Islands: Gran Canaria, Tenerife, La Palma, La Gomera, Lanzarote, Fuerteventura (*Bourgeau* 1426, *Asplund* 721, *Murray* s.n. [30/4/1894]).

##### Notes.

A very distinctive species because of its shrubby habit and white flowers in a terminal paniculate inflorescence.

### Species 172–190. Mostly villous undershrubs with flowers in capitulae

All species are perennial undershrubs, usually with leaves densely villous and lacking distinct petioles. Most are unarmed. The flowers are arranged in sessile or pedunculate capitulae. They are plants mostly of the Middle East from Egypt and Saudi Arabia east to Pakistan, but not in the republics of former Soviet Central Asia.

The first two species, *Convolvulus oxyphyllus* and *Convolvulus hamrinensis* are anomalous in having spinescent branches and few-flowered capitula and form a difficult complex. As defined here, they are mostly easy to separate morphologically and geographically, their range only overlapping in Iraq. However, there are atypical specimens which do not fit comfortably in either species and the problem is intensified by what may be extreme adaptation to drought and desert conditions resulting in densely spiny, almost aphyllous specimens. Our molecular studies (Williams et al. 2014) suggest further study using extensive sampling might unravel this complex.

#### 172. Convolvulus oxyphyllus

Taxon classificationPlantaeSolanalesConvolvulaceae

Boiss., Diagn. Pl. Orient. 7: 26. 1846. (Boissier 1846: 26).

##### Type.

IRAN, *Aucher-Eloy* 4950 (holotype G; isotypes BM!, K!, P!).

##### Description.

A very variable undershrub 25–50 cm high from a woody rootstock, with woody, branches which are usually spinescent, vegetative parts usually densely white-tomentellous to villous but indumentum sometimes very short; side branches long and slender, reaching 30cm or short, stout and spinescent. Basal leaves sessile 0.7–2.5 (-4.5) × 0.3–0.7, oblong-lanceolate, apex acute and mucronate, margin entire, narrowed to a petiole-like base, both surfaces tomentose to tomentellous, but the adaxial surface often greener; stem leaves and bracts smaller than the lower leaves, commonly ovate, acute to apiculate. Flowers 1–several in numerous subsessile villous axillary capitula (very rarely helicoid cymose in form), forming an elongate spicate inflorescence; peduncles absent, bracteoles 3.5–8 × 1.5–3 mm, lanceolate to oblong, acute; outer sepals 5–8 × 2–3.5, narrowly elliptic to oblong-obovate, acute, villous; inner sepals slightly narrower; corolla 1–1.4 cm, white or pink, weakly lobed, midpetaline bands pilose; ovary and style pilose; style divided c 5 mm above base, the stigmas 2.5 mm long. Capsule not known. [Sa’ad 1967: 76; Collenette 1999: 230; Rechinger 1961: 22 ff.; Nowroozi 2002: 34 (plate), 101 (map); Daoud and Al-Rawi 1985 (Plate 214)]

##### Notes.

We recognise two subspecies:

#### 172a. Convolvulus oxyphyllus subsp. oxyphyllus

Taxon classificationPlantaeSolanalesConvolvulaceae

Figure 25, t. 28–35

Convolvulus oxyphyllus subsp. cateniflorus Rech.f., Anz. Österr. Akad. Iss., Math.-Naturwiss. Kl. 98: 23. 1961. (Rechinger 1961: 23). Type. IRAQ, Diyala River, near Mandali, *Rechinger* 9639 (holotype W!).
Convolvulus cateniflorus (Rech.f.) Sa’ad, Meded. Bot. Mus. Herb. Rijks Univ. Utrecht 281: 154. 1967. (Sa’ad 1967: 154). Type. Based on Convolvulus oxyphyllus subsp. cateniflorus Rech.f.

##### Distinguishing features.

The type subspecies is characterised by the sessile, sometimes clasping, very acute, sometimes apiculate stem leaves and bracts, long slender inflorescence branches with flowers usually 2 -3 together, the sepals acute and the corolla 8–13 mm long. Spinescent side branches are few or absent.

##### Distribution.

Iraq (*Wheeler-Haines* 1127, *Rawi* 21548; *Guest et al.* 14347, *Guest* 4016, 4020, *Rechinger* 8072, 9658, *Barklay* 2430, *Uvarov* s.n. [26/5/1932], *Hazim* 30665, *Katib & Tikriti* 29735); Iran (*Haussknech* t s.n. [6/1868]; *Olivier & Brugière* s.n.); Kuwait (*Dickson* 243). Mainly in Iraq.

##### Notes.

*Rawi & Alizzi* 34452 is atypical because of short side branchlets, these and the main branches terminating in slender sharp spines. It is suggested that *Convolvulus cateniflorus* differs from typical *Convolvulus oxyphyllus* in the shorter, less spinescent, more densely tomentose stems and solitary flowers (Rechinger 1964: 483) but all kinds of intermediates can be found.

#### 172b. Convolvulus oxyphyllus subsp. oxycladus

Taxon classificationPlantaeSolanalesConvolvulaceae

Rech.f., Anz. Österr. Akad. Iss., Math.-Naturwiss. Kl. 98: 11. 1961. (Rechinger 1961: 11).

##### Type.

IRAQ, between Ramadi and Rutbah, *Rechinger* 9886 (holotype W!; isotype E!).

##### Distinguishing features.

Lateral branches numerous, short (2–4 cm), rigid, stout, strongly spinescent. Leaves somewhat caducous. Flowers several together, the inflorescence often elongating at maturity, reaching 3 cm in length

##### Distribution.

Iraq (*Rechinger* 8190, 9571, *Barklay & Abbas-al-Ani* 3606, *Alizzi & Omar* 35230); Iran (*Bokhari et al.* 14790). Less common than the type variety.

##### Notes.

The type of this subspecies is immature and almost flowerless. It could easily be interpreted as a form of *Convolvulus hamrinensis* with apiculate leaves. We have based our interpretation of this subspecies on *Guest, Rawi & Rechinger* 16058, apparently the same collection as *Rechinger* 8190).

#### 173. Convolvulus hamrinensis

Taxon classificationPlantaeSolanalesConvolvulaceae

Rech.f., Anz. Österr. Akad. Iss., Math.-Naturwiss. Kl. 98: 11. 1961. (Rechinger 1961: 11).

Figure 25, t. 36–42

Convolvulus oxyphyllus subsp. sheilae R.R.Mill, Edinburgh J. Bot. 70: 376. 2013. (Mill 2013: 376. Type. SAUDI ARABIA, *Collenette* 2836 (holotype E!; isotype K!).
Convolvulus infantispinosus R.R.Mill, Edinburgh J. Bot. 70: 374. 2013. (Mill 2013: 374). Type. SAUDI ARABIA, *Collenette* 2210 (holotype E!; isotype K!).

##### Type.

IRAQ, *Rechinger* 8083 (holotype W!; isotype E00699568!).

##### Description.

Undershrub with spinescent branches forming a low bush to 60 cm high and up to 1 m wide; young branchlets white-tomentellous, side branches rather short, c. 5 cm long, sometimes spine-like. Basal leaves 0.5–3 × 0.3–0.6, oblanceolate, obtuse, margin undulate, base attenuate into a long petiole-like base, densely puberulent on both surfaces, paler beneath; stem leaves and bracts 3 × 2 mm, oblong to oblanceolate, obtuse to subacute, cuneate at base. Flowers 1 (-2), sessile in the axils of bracts; bracteoles 2 × 0.5 mm, linear-oblong, pedicels 0–1 mm; sepals 5–6 × 2–3 mm, oblong to oblanceolate, obtuse, densely pilose; corolla 0.6–1cm long, white, slightly lobed, midpetaline bands pilose; ovary hirsute, style glabrous, divided c. 4 mm above base, stigmas 4 mm. Capsule not seen.

##### Distribution.

Locally abundant principally in sandy desert: Syria (?); Iraq (*Guest et al.* 16151B, *Mohallal* 19504, both sterile); Saudi Arabia: Jouf, Hail, Riyadh to Eastern Province. *Mandaville* 199, 217, 2271, 2328, 2465, 2485, 3124, 3208; *Popov* 69/224, 72/57: *Chaudhary* 3467; *Wood* 71/269; *Philby* s.n. [15/8/1931]; *Vesey-Fitzgerald* 15888; *Hillcoat* 364; *Dickson* 1074; *Collenette* 97, 1851, 5332, 7214, 7879, 7883, 7886. The type is from east of Baghdad but the numerous records from Saudi Arabia seem indistinguishable.

##### Notes.

Arabian specimens of this species have been misidentified as *Convolvulus lanatus*. The type of *Convolvulus infantispinosus* (*Collenette* 2210) has more delicate spines but seems to be only a form of *Convolvulus hamrinensis*.

This species possibly intergrades with *Convolvulus oxyphyllus* but is nearly always easily identified. The plant has a characteristic appearance with short rigid spine-like side branches to the stiff woody spinescent main stem. Leaves and bracts are typically obtuse, the lower leaves petiolate. Flowers are solitary (rarely paired) axillary, usually much exceeding the rather inconspicuous bracts. At one extreme a few specimens (*Collenette* 5351, *Rechinger* 9886, *Chaudhary* 3467, *Omar et al.* 43961) have acute leaves and bracts, clearly approaching *Convolvulus oxyphyllus*. At the other extreme, specimens with obtuse, undulate leaves and bracts are found (*Collenette* 1851, 5332, 7874, *Mandaville* 7850). *Collenette* 1851 is especially close to the type of *Convolvulus hamrinensis*.

#### 174. Convolvulus kotschyanus

Taxon classificationPlantaeSolanalesConvolvulaceae

Boiss., Diagn. Pl. Orient. 7: 23. 1846. (Boissier 1846: 23).

Figure 24, t. 20–27

Convolvulus gonocladus Boiss., Diagn. Pl. Orient. 7: 22. 1846. (Boissier 1846: 22). Type. IRAN, *Kotschy* 207 (holotype G; isotypes P!, W!).
Convolvulus haussknechtii Boiss., Fl. Orient. [Boissier] 4: 102. 1875, p.p. illegitimate superfluous name for both *Convolvulus gonocladus* Boiss. and *Convolvulus pyrrotrichus* Boiss. (1846) cited in synonymy; specimens from Iran (Boissier 1875b: 102). Type. Various syntypes.

##### Type.

IRAN, Shiraz, *Kotschy* 357 (holotype G; isotypes BM 001014562!, C, E!, GOET, HAL, JE, L, OXF!, P!, W!).

##### Description.

Weakly cushion-forming plant with a woody rootstock, from which arise ascending stems to 45 cm, vegetative parts pilose with somewhat stiff spreading hairs. Basal leaves 3–8 × 0.5–0.9 cm, oblong-oblanceolate, acute, entire, base long-attenuate and petiole-like up to 4 cm in length; stem leaves 2–5 × 0.5–1 cm, oblanceolate to oblong, acute, entire, base cuneate to attenuate. Flowers in sessile (above) or shortly pedunculate (below) 2–3-flowered axillary heads; bracts usually < 3 cm long, lanceolate (if longer, than narrowly lanceolate); peduncles 0–1.5 cm; bracteoles 9–11 × 1 mm, linear-oblong, acute, densely pilose; pedicels absent; outer sepals 9–11 × 2–2.5 mm, lanceolate, acuminate, densely pilose; inner sepals 8–9 × 1 mm, linear-lanceolate; corolla 2–2.5 cm long, pink, shallowly lobed, midpetaline bands darker, adpressed pilose; ovary glabrous; style glabrous, divided 3 mm above base, stigmas 4.5–5 mm; capsule glabrous; seeds glabrous. [Sa’ad 1967: 162; Austin and Ghazanfar 1979: 19]

##### Distribution.

Pakistan; southern Iran (*Davis & Bokhari* 55970, *Macmillan* 225, *Koelz* 15039, *Stutz* 979, *Stapf* 298); Iraq (*Thamer* 47695).

##### Notes.

This species has been confused with *Convolvulus prostratus*, but is distinguished by its much larger corolla, monocoloured sepals and more developed cushion-like base. It has also been confused with *Convolvulus pyrrotrichus* but is distinguished by the narrower, lanceolate bracts < 3 × 1 cm.

#### 175. Convolvulus pyrrotrichus

Taxon classificationPlantaeSolanalesConvolvulaceae

Boiss., Diagn. Pl. Orient., ser. 2, 3: 122. 1856. (Boissier 1856: 122).

Figure 24, t. 38–44

Convolvulus gonocladus subsp. pyrrotrichus (Boiss.) Rech.f., Biol. Meddel. Kongel. Danske Vidensk. Selsk. 10: 80. 1959. (Rechinger 1959: 80) Type. Based on *Convolvulus pyrrotrichus* Boiss.
Convolvulus haussknechtii Boiss., Fl Orient. [Boissier] 4: 102. 1875, p.p. illegitimate superfluous name for for both *Convolvulus gonocladus* Boiss. and *Convolvulus pyrrotrichus* Boiss. (1846) cited in synonymy; specimens from Afghanistan (*Griffith* 5879) (Boissier 1875b: 102). Type. Various syntypes.

##### Type.

AFGHANISTAN, *Griffith* 5859 (holotype G; isotypes K!, P!).

##### Description.

Vigorous perennial with stout, woody rootstock and stem base, to 50 cm high, the whole plant densely covered in long, soft white hairs. Basal leaves 3–13 × 0.6–2.5 cm, oblong-lanceolate, acute at both ends and with a long winged petiolate base; stem leaves sessile, 3–12 × 0.3–3.8 cm, elliptic or oblong-elliptic, acute, entire, broadly to narrowly cuneate at base, diminishing in size upwards, veins prominent. Inflorescence of sessile (above) or shortly pedunculate (below) capitula or scorpioid cymes arising from the upper part of the stem; bracts mostly 3–4 cm × 1.2–2 cm, ovate; peduncles 0–3 cm, bracteoles 12–23 × 1.5–3 mm, linear-lanceolate, acuminate; pedicels 0–1 mm; outer sepals 10–14 × 3.5 mm, ovate, acuminate, covered with an indumentum of short dense hairs mixed with longer spreading hairs; inner sepals convex, glabrous; corolla 2–2.2 cm. white, weakly lobed, midpetaline bands pilose; ovary and style glabrous, style divided c. 2.5 mm above base, stigmas c.2.5 mm; capsule glabrous, seeds pubescent. [Sa’ad 1967: 164; Austin and Ghazanfar 1979: 20, 21(plate); Breckle and Rafiqpoor 2010: 417 (photo)]

##### Distribution.

Afghanistan (*Grey-Wilson & Hewer* 1025, 1073; *Hedge & Wendelbo* 4283, 5107; *Lamond* 1902, 2534; *Rechinger* 16974, 32408); Pakistan (*Lowndes* 688).

#### 176. Convolvulus lanatus

Taxon classificationPlantaeSolanalesConvolvulaceae

Vahl, Symb. Bot. 1: 16. 1790. (Vahl 1790: 16).

Figure 25, t. 1–7

Convolvulus cneorum Forssk., Fl. Aegypt.-Arab. 63.1775, nom. illeg., non *Convolvulus cneorum* L. (1753). (Forsskål 1775: 63). Type. EGYPT, Sinai, *Forsskål* 456 (syntypes BM!, C).
Convolvulus forskalei Delile, Descr. Egypte, Hist. Nat. 190. 1813. (Delile 1813: 190). Type. EGYPT, El Salhiya, *Delile* s.n. (holotype MPU).

##### Type.

Based on *Convolvulus cneorum* Forssk.

##### Description.

Undershrub from a woody base to 35 cm, the lower branches woody and spinescent, younger brances herbaceous; vegetative parts softly white-tomentose with spreading hairs. Leaves sessile, 1–3 × 0.3–0.5 cm, oblanceolate below, oblong above, acute, margin entire, base cuneate. Flowers in subsessile heads along the upper part of the stem; bracts oblong-elliptic to ovate; peduncles 0–5 mm; bracteoles 10 × 5 mm, ovate; pedicels absent; outer sepals 10 × 4 mm ovate, acute; inner sepals 7 × 2 mm, lanceolate, much narrower than outer sepals; corolla 1.8–2.3 cm, pale pink or white, somewhat undulate, midpetaline bands pilose; ovary and style glabrous; style divided 5 mm above base, the stigmas 4 mm. Capsule glabrous; seeds smooth, glabrous (fide Feinbrun-Dothan 1978: 37). [Sa’ad 1967: 80; Parris 1978: 203 p.p.; Boulos 2000: 331; Strid and Strid 2010: 4–5 (plate].

##### Distribution.

Lower Egypt and Sinai (*Schweinfurth* 2148, 1246; *Keller* 32; *Boulos et al.* 20333, 20349; *Danin* S-1990, *Davis* 10540, *Schimper* 727); Palestine/Israel: Negev (*Zohary* 7325); Turkey (*Palmer* T/60). The occurrence of this species in Turkey is correct, although unexpected, but records from other countries including Arabia are probably errors.

##### Notes.

Replaces *Convolvulus secundus* in Egypt and Sinai. Differs principally in habit, forming a compact spiny bushlet, the lower branches and side shoots woody and spinescent. The sepals and bracteoles are more densely villous with the flower heads almost contiguous.

#### 177. Convolvulus secundus

Taxon classificationPlantaeSolanalesConvolvulaceae

Desr., Encycl. [Lamarck et al.] 3: 553. 1792. (Desrousseaux 1792: 553).

Figure 25, t. 8–14

Convolvulus salvifolius Sieber ex Link, Enum. Hort. Berol. Alt. 1: 203. 1821. (Link 1821: 203). Type. Palestine/Israel, *Sieber* s.n. (?B†).
Convolvulus secundus var. latifolius Post, Fl. Syria: 561. 1896. (Post 1896: 561). Type. No type cited.
Convolvulus secundus var. parvifolius Post, Fl. Syria: 561. 1896. (Post 1896:561). Type. No type cited.

##### Type.

“Levant”, *sin coll.* (holotype P [Herb.Lam.]!).

##### Description.

Perennial herb from a woody base, stems prostrate, reaching 50 cm, the whole plant densely villous. Leaves sessile, 2–4 × 0.4–1.3 cm, oblanceolate or oblong, becoming elliptic upwards, obtuse, margin entire, lower leaves attenuate at base, upper leaves and bracts cuneate, veins prominent, internodes short so leaves and flower heads more crowded than in related species. Inflorescence characteristically narrow, flowers 1–5 in subsessile, congested head-like scorpioid cymes; peduncle 0–1.5 cm; bracteoles 8–11 × 3–5 mm, lanceolate to ovate, acute; pedicels absent; outer sepals 12–15 × 3–4 mm, oblong, acuminate, flat; middle sepal asymmetric; inner sepals slightly shorter and narrower; corolla 2–2.8 cm long, usually creamy with a yellowish centre, weakly lobed, midpetaline bands long-sericeous; ovary and style glabrous, style divided 6 mm above base, stigmas 3mm; capsule glabrous, 1-seeded (fide Sa’ad 1967); seeds glabrous. [Sa’ad 1967: 168; Tohmé and Tohmé 2007: 216 (photo)]

##### Distribution.

Turkey (*Uslu* 1363, 2671); Syria (*Aucher-Eloy* 1398); Lebanon (*Gombault* 693, *Blanche* 1457); Palestine/Israel (*Eig & Grizzi* 369, *Bornmüller* 1108, 1109; *Meyers & Dinsmore* 272; *Davis* 4424, 4672). On coastal sand. Records from Egypt are probably errors.

##### Notes.

Unlike *Convolvulus lanatus* this species appears to have long trailing stems, which may become somewhat woody when old, but it lacks the spinescent basal branches of *Convolvulus lanatus*. The sepals are also more shortly villous so the heads are more or less separate. It differs from *Convolvulus schimperi* in the subsessile heads and leaves not undulate-margined and from *Convolvulus spicatus* in the subsessile heads although short peduncles are clearly present in some specimens such as *Melville* 70/31 and *Samuelson* 527 (both K).

Species 178–181 are extremely similar and may prove to be conspecific. *Convolvulus cephalodus* differs in the hirsute ovary, but other distinctions are essentially minor differences of leaf shape. Clearly further collections are needed but not even the ovary indumentum is very convincing in the absence of other distinguishing characters.

#### 178. Convolvulus spicatus

Taxon classificationPlantaeSolanalesConvolvulaceae

Peter ex Hallier f., Bot. Jahrb. Syst. 18: 99. 1893 [“1894”]. (Hallier 1893: 99).

Figure 24, t. 7–13

##### Type.

EGYPT, Sinai, Wadi Feiran, *March* s.n. (lectotype GOET, designated by Sa’ad 1967: 169).

##### Description.

Villous perennial to 40 cm. Leaves sessile, 1.3–7 × 0.4–1.2 cm, oblong to oblanceolate, acute, margin entire, attenuate at the base, softly white-villous. Flowers in dense axillary heads; peduncles 1.5–3.5 cm, exceeding bracts; bracteoles 6–15 × 1–2 mm, linear-lanceolate, acuminate, densely villous; outer sepals 7–10 × 2–3 mm, lanceolate, acuminate, inner sepals narrower, 1–2 mm wide; corolla 2–2.5 cm, unlobed, midpetaline bands pubescent, terminating in a tooth; ovary and style glabrous; style divided c. 3 mm above base, the stigmas 5 mm, longer than the united part of the style. [Sa’ad 1967: 169; Boulos 2000: 247]

##### Distribution.

Egypt: Sinai (*Bové* 503, *Danin* s.n. [28/5/1962]), Jordan: Wadi Sirhan (*Hunting Survey* 117a). Apparently very rare.

##### Notes.

Differs from the two previous species in the pedunculate heads but superficially identical to Convolvulus cephalopodus subsp. bushiricus, from which it is distinguished by the glabrous ovary. Very similar to *Convolvulus schimperi* differing in the ascending habit and in the leaves which are not undulate.

The lectotype is a meagre specimen and all the other cited specimens represent more complete material.

#### 179. Convolvulus jordanensis

Taxon classificationPlantaeSolanalesConvolvulaceae

Sa’ad, Meded. Bot. Mus. Herb. Rijks Univ. Utrecht 281: 166. 1967. (Sa’ad 1967: 159).

Figure 24, t. 1–6

##### Type.

JORDAN, *Robertson* 120 (holotype K!).

##### Description.

Similar to the previous species. Perennial to 40 cm; stems slightly zigzag. Leaves sessile, 1.3–4 × 0.3–0.4 cm, oblong, acute (not spinescent as stated by Sa’ad), margin entire, shortly tomentose. Flowers in dense axillary heads, becoming scorpioid at maturity; peduncles 1.5–3 cm, exceeding bracts; bracteoles 6–7 × 3.5–4 mm, ovate, caudate (not spinescent), densely villous; outer sepals 7 × 3 mm, elliptic, shortly acuminate or caudate, inner sepals oblong, c. 1 mm wide; corolla 2–2.5 cm, unlobed, midpetaline bands pubescent, terminating in a tooth; ovary and style glabrous; style divided c. 2 mm above base, the stigmas 5–6 mm long.

##### Distribution.

Endemic to Jordan where it is known from one unlocalised record.

##### Notes.

The most distinct species in this complex because of the narrowly oblong, shortly tomentose leaves combined with the pointed bracteoles and sepals but only known from the type.

#### 180. Convolvulus schimperi

Taxon classificationPlantaeSolanalesConvolvulaceae

Boiss., Diagn. Pl. Orient. 11: 81. 1849. (Boissier 1849: 81).

Figure 24, t. 14–19

##### Type.

EGYPT, Sinai, *Schimper* s.n. (holotype G; isotypes BM!, K!, P!).

##### Description.

Perennial herb from a stout woody rootstock to 50 cm in height, all parts covered in long spreading hairs, which are rust-coloured when dry. Basal leaves petiolate, 1.5–6.5 × 0.5–1 cm, obovate or oblanceolate, obtuse, margin strongly undulate, with long petiole-like base; stem leaves sessile, shorter (< 2.5 cm long), linear-oblanceolate. Flowers in pedunculate heads with up to 5 flowers; bracts 1–1.5 × 0.5–0.7 cm, oblong-elliptic; peduncles 0–11 mm long, about equalling the subtending bract; bracteoles 12 × 2 mm, linear-oblanceolate; pedicels absent; outer sepals 10–11 × 2–3 mm, lanceolate, acute, villous; inner sepals narrower and shorter, c. 8–9 × 1 mm; corolla 2–2.2 cm, colour unknown, wavy at the apex, scarcely lobed, midpetaline bands pilose; ovary and style glabrous divided c. 5 mm above base, stigmas c. 5 mm. Capsule not seen. [Sa’ad 1967: 167; Boulos 2000: 247]

##### Distribution.

Endemic to Sinai in Egypt (*Holland* s.n., *MacDonald* s.n., *Hart* s.n. [1883/4]). Apparently a rare species. Records from Arabia are errors.

##### Notes.

Distinguished from Convolvulus spicatus and *Convolvulus jordanensis* by the strongly undulate leaves. It is very similar to some forms of *Convolvulus cephalopodus* but distinguished by the glabrous ovary. It appears to be prostrate like *Convolvulus secundus*, but is distinguished by the shortly pedunculate heads as well as the distinctive leaves.

#### 181. Convolvulus cephalopodus

Taxon classificationPlantaeSolanalesConvolvulaceae

Boiss., Diagn. Pl. Orient. 7: 24. 1846. (Boissier 1846: 24).

##### Type.

IRAN, Makran, *Aucher-Eloy* 4949 (holotype G; isotypes BM000049216!, K!, OXF!, W!).

##### Description.

Perennial herb from a woody taproot, from which arise ascending stems 30–35 cm high. Branches stout; whole plant densely pubescent with grey hairs. Leaves sessile, 1.5–4 × 0.4–1 cm, oblong-oblanceolate, obtuse, margin crisped-undulate, attenuate to a long petiole-like base, sometimes the abaxial surface notably paler. Inflorescence of axillary, pedunculate heads, which are occasionally elongated into more or less scorpioid cymes; peduncles 1.2–1.5 cm; bracteoles 8 × 5 mm, ovate-elliptic, acute or shortly acuminate; outer sepals 10–12 × 3 mm, broadly lanceolate to ovate, acuminate; inner sepals shorter and narrower, c. 8 × 2 mm; corolla 1.5–2 cm, pinkish, unlobed, midpetaline bands pilose; ovary comose; style glabrous or with a few hairs, divided c. 2.5 mm above base, stigmas c. 2 mm long; capsule comose, seeds pubescent. [Sa’ad 1967: 156; Nowroozi 2002: 77 (plate), 105 (map); Austin and Ghazanfar 1979: 20, 21 (plate); Jongbloed 2003: 311 (photo); Pickering and Patzelt 2008: 168 (photo)]

##### Notes.

We recognise two, not very well-defined subspecies, which are bridged by intermediates:

#### 181a. Convolvulus cephalopodus subsp. cephalopodus

Taxon classificationPlantaeSolanalesConvolvulaceae

Figure 23, t. 9–15

Convolvulus sericeus Burm.f., Fl. Indica 49. 1768, nom. illeg., non *Convolvulus sericeus* L. (1767). (Burman 1768: 49). Type. IRAN, *Garzin* s.n. (holotype G).
Convolvulus omanensis S.Moore, J. Bot. 37: 405. 1899. (Moore 1899: 405). Type. OMAN, *A.S.G.Jayakar* 171 (holotype BM000049219!).
Convolvulus beluchistanensis Biswas, J. Bot. 75: 259. 1937. (Biswas 1937: 259). Type. PAKISTAN, Balochistan, *Ramchandra Rao & Krandikar* 30 (holotype CAL; isotype BM001014563!).
Convolvulus undulifolius Parsa, Kew Bull. 3: 214. 1948. (Parsa 1948: 214). Type. IRAN, Bandar Abbas, *Parsa* 564 (holotype K!).
Convolvulus undulifolius var. secundus Parsa, Kew Bull. 3: 214. 1948 (Parsa 1948: 214). Type. IRAN, Charbar, *Parsa* 565 (holotype K!).

##### Distinguishing features.

Leaves relatively short, usually less than 5 cm long, indumentum asperous. Style glabrous or with a few hairs.

**Figure 23.**
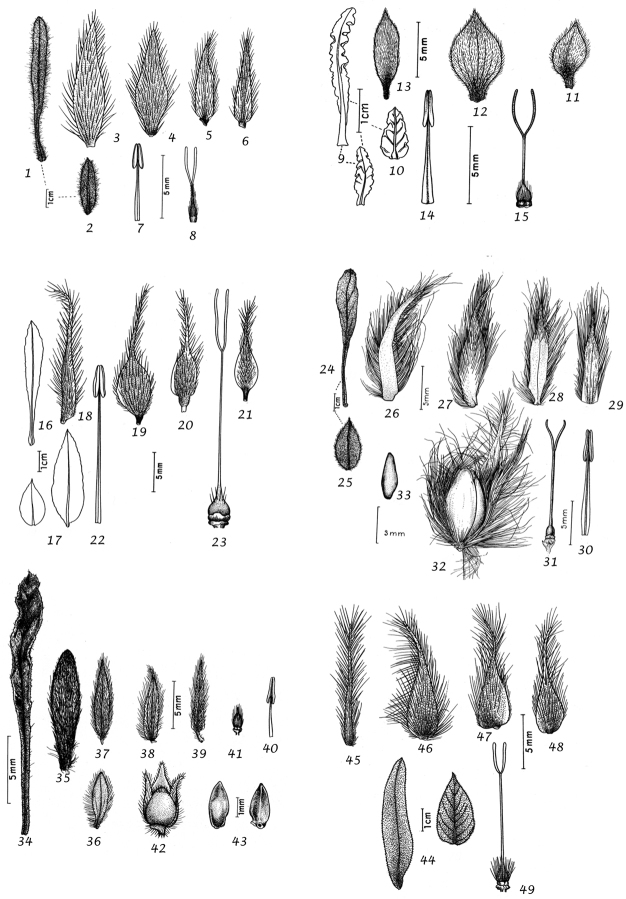
**1–8** Convolvulus cephalopodus subsp. bushiricus **1–2** leaves **3** bracteole **4** outer sepal **5** middle sepal **6** inner sepal **7** stamen **8** ovary and style **1–2** from *Rechinger* 8803 (W) **3–8** from *Køie* 272 (B) **9–15** Convolvulus cephalopodus subsp. cephalopodus **9** leaves **10** bract **11** bracteole **12** outer sepal **13** inner sepal **14** stamen **15** ovary and style **9–10** from *Rechinger* 3448a (W) **11–15** from *Rechinger* 3448b (W) **16–23** *Convolvulus cephalophorus* **16** leaf **17** bracts **18** bracteole **19** outer sepal **20** middle sepal **21** inner sepal **22** stamen **23** ovary and style. **16** from *Kotschy* 138 (W) **17–23** from *Stutz* 906 (W) **24–33** *Convolvulus euphraticus* **24** leaf **25** bract **26** bracteole **27** outer sepal **28** middle sepal **29** inner sepal **30** stamen **31** ovary and style **32** capsule **33** seed. From *Rechinger* 9959 (W) **34–43** *Convolvulus asyrensis* **34** leaf **35** bract **36** bracteole **37** outer sepal **38** middle sepal **39** inner sepal **40** stamen **41** ovary **42** capsule **43** seeds. From *sin coll*. 102.148 (W) **44–49** *Convolvulus stapfii* **44** leaves **45** bracteole **46** outer sepal **47** middle sepal **48** inner sepal **49** ovary and style **44–48** from *Stapf* 374 (W), **49** from *Stapf* 1062 (K).

##### Distribution.

Pakistan (*Ghafoor & Goodman* 4829, *Popov* 29); Iran (*Wendelbo & Foroughi* 15571A), *Merton* 3616, *Léonard* 5913, *Grey-Wilson & Hewer* 286); Bahrain (*Good* 192); U.A.E. (*Ghazanfar* 4254, *Western* 258); Qatar (*Boulo* s 11145); Saudi Arabia (*Collenette* 5281); Oman (*Radcliffe-Smith* 3907, *Gallagher* 6692, *Guichard* KG/83/Oman).

##### Notes.

One collection from Iranian Baluchestan (*Pierce* s.n. [11/1880]) appears to be intermediate with *Convolvulus pyrrotrichus*.

Fruiting heads often become distinctly scorpioid in structure as in *Western* 421, *Ghazanfur* 4254 & *Osborne* 602. Occasionally the heads are sessile as in *Collenette* 4159 and 9385 from Saudi Arabia and *Parsa* 565 from Iran; these forms resemble *Convolvulus kotschyanus* morphologically but can be distinguished by the undulate leaves and hirsute ovary.

#### 181b. Convolvulus cephalopodus subsp. bushiricus

Taxon classificationPlantaeSolanalesConvolvulaceae

(Bornm.) J.R.I.Wood & R.W.Scotland stat. nov.

urn:lsid:ipni.org:names:77147680-1

Figure 23, t. 1–8

Convolvulus bushiricus Bornm., Dan. Sci. Invest. Iran 4: 35. 1945. (Köie 1945: 35). Type. IRAN, Bushehr, *Köie* 272 (holotype C; isotype B!).

##### Type.

Based on *Convolvulus bushiricus* Bornm.

##### Distinguishing features.

Differs from subsp. cephalopodus in the longer, softer villous indumentum, the longer leaves (2.5–9 v. 1.5–4 cm) with less obviously undulate margins, the hairy style, in which the stigmas are longer than the united part, the lanceolate bracteoles and narrower sepals, the outer only 2–2.5 mm wide. The defining characters are not well differentiated and intermediates are known to occur. The ovary is hirsute, not glabrous as stated by Rechinger (1964: 486). [Sa’ad 1967: 151; Collenette 1999: 228 (photo); Daoud and Al-Rawi 1985 (Plates 215–217)]

##### Distribution.

Principally around the head of the Persian/Arabian Gulf: Iran (*Kasy* 356); Iraq (*Rawi & Rechinger* 17242, *Rechinger* 8812); Kuwait (*Dickson* 60, 748, *Rawi et al.* 10521); Saudi Arabia (*Holm* 60, *Mandaville* 552, *Collenette* 2497, 9406).

##### Notes.

Mandaville (1990: 246) suggests that the distinguishing features of subsp. bushiricus may be the result of seasonal or edaphic factors. This seems unlikely in the case of *Convolvulus cephalopodus* as only material from around the head of the Gulf seems to conform to subsp. bushiricus.

*Convolvulus euphraticus* is similar morphogically but has consistently larger heads c. 2.5 cm in diameter as opposed to 1.5 cm in diameter in Convolvulus cephalopodus subsp. bushiricus.

#### 182. Convolvulus euphraticus

Taxon classificationPlantaeSolanalesConvolvulaceae

Bornm., Beih. Bot. Centralbl. 20(2): 181. 1906. (Bornmüller 1906: 181).

Figure 23, t. 24–33

##### Type.

IRAQ [probably], inter Arrah et Deïr, *Strauss* s.n. (B!).

##### Description.

Perennial herb from a woody taproot and base with stems to 40 cm, plant roughly tomentose with longish white hairs. Basal leaves 5–11 × 0.6–1.7 cm, oblanceolate, obtuse or rounded, margin entire to slightly undulate, base narrowed into a pseudopetiole c. 2–4 cm long; stem leaves and bracts sessile, 2–5 × 1.5–2 cm, ovate, acute, base broadly cuneate. Flowers in many-flowered, axillary, pedunculate heads, mostly 2–2.5 cm in diameter, occasionally somewhat elongate; peduncles 1.5–5 cm; bracteoles 10–15 × 1–3 mm, linear to lanceolate, long acuminate, pilose; pedicels absent; sepals 8–9 × 2.5–3 mm, lanceolate, acuminate, long-pilose, inner sepals slightly narrower, c. 2 mm wide; corolla 2–2.3 cm long, pink, midpetaline bands pilose, very shallowly lobed with midpetaline bands terminating in a comose point; ovary glabrous; style glabrous, divided 5 mm above base, stigmas 4 mm. Capsule glabrous; seeds glabrous. [Sa’ad 1967: 157; Rechinger 1961: 24]

##### Distribution.

Iran, Iraq (*Barklay & Palmatier* 2266; *Rechinger* 9797, 9959; *Alizzi & Husain* 34096; *Rawi & Nur* 27028; *Hamad* 48878); Saudi Arabia? Common in Iraq but very rare or absent elsewhere.

##### Notes.

Resembles Convolvulus cephalopodus subsp. bushiricus very closely in overall morphology but the heads are larger, pedunculate almost to the apex of the stem, the bracteoles are much longer and the ovary and style are glabrous.

#### 183. Convolvulus asyrensis

Taxon classificationPlantaeSolanalesConvolvulaceae

Kotschy, Sitzungsber. Kaiserl. Akad. Wiss., Math.-Naturwiss. Cl., 52: 260, pl. 5. 1866. (Kotschy 1866: 260).

Figure 23, t. 34–43

Convolvulus cephalopodus subsp. abhansis Alfarhan, Bot. J. Linn. Soc. 106; 262. 1991. (Alfarhan 1991: 262). Type. SAUDI ARABIA, Asir, *Nasher* H-20 (holotype E00285433!).

##### Type.

SAUDI ARABIA, Asir, *coll. unknown* (holotype W!).

##### Description.

Cushion-like perennial from a woody rootstock with decumbent to ascending stems up to 75 cm long, plant densely white-tomentellous. Basal leaves petiolate, 0.3–2 × (0.1-)0.4–0.8 cm, broadly oblanceolate, acute, base cuneate, petiole up to 2 cm long; stem leaves sessile, 2–5 × 0.5–0.8 cm, similar in shape, diminishing in size upwards and merging into bracts. Inflorescence of sessile axillary clusters towards the branch tips, raceme-like; bracteoles 3 × 2 mm, elliptic, acute; outer sepals 7 × 2 mm, oblong-lanceolate, acute, pilose; inner sepals narrower (c 1 mm wide) and slightly shorter, the margins membranous; corolla 1.6–2 cm, pink or white, undulate, midpetaline bands thinly pilose; ovary comose; style glabrous; capsule comose, seeds glabrous (or puberulent fide R.R.Mill). [Sa’ad 1967: 150; Collenette 1999: 226 (photo)]

##### Distribution.

Endemic to Saudi Arabia: Asir region (*Collenette* 1686, 4159, 6815).

##### Notes.

Distinguished by the cushion habit, sessile heads and usually small leaves.

#### 184. Convolvulus aitchisonii

Taxon classificationPlantaeSolanalesConvolvulaceae

C.B.Clarke, J. Linn. Soc., Bot. 19: 179. 1882. (Aitchison 1882: 179).

Convolvulus lanuginosus sensu Aitch., Cat. Pl. Punjab Sindh: 98. 1869, non *Convolvulus lanuginosus* Desr. (1792).

##### Type.

AFGHANISTAN/PAKISTAN, Kurram Valley, *Aitchison* 15 (holotype K!).

##### Description.

Cushion/hummock forming plant with woody taproot, branched just below the ground surface to produce many very short shoots from which arise flowering stems 1–9 cm high. Leaves aggregated at the shoot tips, sessile, 2–2.5 × 0.2–0.3(-0.4) cm, oblanceolate, acute, margin entire, attenuate at the base, densely villous. Flowers 1–4 in subsessile helicoid cymes, usually reduced to heads which arise in the axils of the upper bracts; bracts 1.5 cm long, oblong, villous; peduncles 0–2 mm; bracteoles 10 × 0.5 mm, linear, acuminate, villous; pedicels absent; outer sepals villous; outer sepals 11–13 × 2–3 mm, ovate acuminate; inner sepals narrower, lanceolate c. 2 mm wide; corolla 1.4–1.5 cm long, cream (?), weakly lobed, midpetaline bands pilose; ovary and style glabrous; style divided c. 3 mm above base, stigmas 4–4.5 mm long; Capsule and seeds not known. [Sa’ad 1967: 134; Austin and Ghazanfar 1979: 19]

##### Distribution.

Pakistan (*Drummond* 14414, *Duthie* 14984, *Rechinger* 30916); Afghanistan (*Rechinger* 35506, 35719).

##### Notes.

Distinguished by its cushion-like habit and subsessile heads borne on a short stem.

#### 185. Convolvulus reticulatus

Taxon classificationPlantaeSolanalesConvolvulaceae

Choisy, Prodr. [A.P. de Candolle] 9: 399. 1845. (Choisy 1845: 399).

Figure 24, t. 28–37

##### Type.

IRAQ, *Aucher-Eloy* 1408 (lectotype G-DC, designated by Sa’ad 1967: 165).

##### Description.

Prostrate perennial to 50 cm from a woody taproot, the whole plant densely velvety-tomentose of characteristic dark brown colour in herbarium specimens; stems 2–3 mm thick. Leaves bullate above, reticulate and greener beneath, becoming leathery when old; lower leaves up to 9 × 2.2 cm, oblong-elliptic, obtuse, entire, attenuate at base; stem leaves and bracts ovate, acute, abruptly rounded at base. Flowers in pedunculate (below) or subsessile (above) or all subsessile, axillary several-flowered heads; bracts c. 1.5–3 × 1–2.2 cm, ovate, nearly as broad as long; peduncles 0–5 cm, tomentose; bracteoles 6–10 × 2–5 mm, lanceolate to elliptic, acute; sepals 8–11 × 1.5–2.5 mm, lanceolate, acuminate, pilose; inner sepals narrower, c. 0.5–1 mm wide; corolla 1.2–1.5 cm long, white, unlobed but slightly undulate, midpetaline bands adpressed brown-pilose; ovary glabrous; style glabrous, divided c. 3 mm above base; stigmas 4 mm; capsule glabrous; seeds pubescent (fide Sa’ad). [Sa’ad 1967: 165]

**Figure 24.**
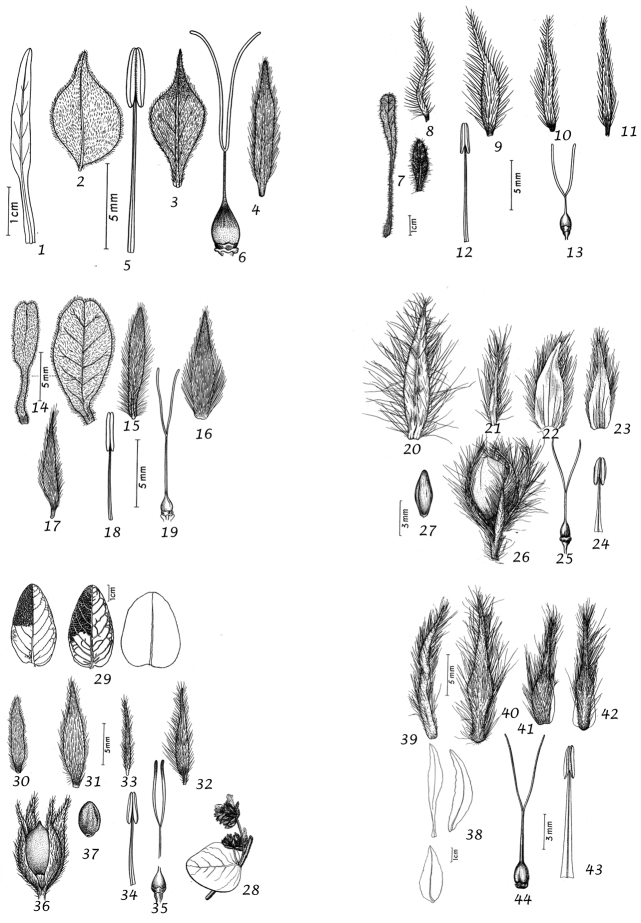
**1–6** *Convolvulus jordanensis* **1** Leaf **2** bracteole **3** outer sepal **4** inner sepal **5** stamen **6** ovary and style. From *Robertson* 120 (K) **7–13** *Convolvulus spicatus* **7** leaves **8** bracteole **9** outer sepal **10** middle sepal **11** inner sepal **12** stamen **13** ovary and style **7** from *Drar* 162 (CAIM) **8–13** from *March* s.n. (GOET) **14–19** *Convolvulus schimperi* **14** leaves **15** bracteole **16** outer sepal **17** inner sepal **18** stamen **19** ovary and style. From *Bornműller* 10896 (B) **20–27** *Convolvulus kotschyanus* **20** leaf **21** bracteole **22** outer sepal **23** inner sepal **24** stamen **25** ovary and style **26** capsule **27** seed. From *Bent & Wright* 503-103 (W) **28–37** Convolvulus reticulatus subsp. reticulatus **28** habit **29** leaves **30** bracteole **31** outer sepal **32** middle sepal **33** inner sepal **34** stamen **35** ovary and style **36** capsule **37** seed **28–29 & 36–37** from *Handel Mazzetti* 2975 (W) **30–35** from *Davis* 22109 (E) **38–44** *Convolvulus pyrrotrichus* **38** leaves **39** bracteole **40** outer sepal **41** middle sepal **42** inner sepal **43** stamen **44** ovary and style. From *Gilli* 3087 (W).

##### Notes.

We recognise two subspecies:

#### 185a. Convolvulus reticulatus subsp. reticulatus

Taxon classificationPlantaeSolanalesConvolvulaceae

##### Distinguishing features.

Stems relatively slender 2–3 mm wide, bracteoles lanceolate 2–3 mm wide, sepals lanceolate, 1.5 to 2.5 cm wide.

##### Distribution.

Western Iran (*Rechinger* 43614, *Andersen & Petersen* 85, *Koelz* 18630); Turkey (*Davis* 22109, *Sintenis* 1289); Iraq (*Bornmüller* 1525, *Handel-Mazzetti* 2975, *Hossain* 4265, *Guest* 4044, *Rechinger* 10087, *Kotschy* 338); Syria (?).

#### 185b. Convolvulus reticulatus subsp. waltherioides

Taxon classificationPlantaeSolanalesConvolvulaceae

(Boiss. & Hausskn.) Sa’ad, Meded. Bot. Mus. Herb. Rijks Univ. Utrecht 281: 166. 1967. (Sa’ad 1967: 166).

Convolvulus waltherioides Boiss. & Hausskn., Pl. Orient. Nov. (dec. prim.) 1: 6. 1875. (Boissier 1875a: 6). Type. IRAN, Behbehan, *Haussknecht* s.n. (lectotype G, designated by Sa’ad 1967: 166); isolectotypes JE, W!)

##### Type.

Based on *Convolvulus waltherioides* Boiss. & Hausskn.

##### Distinguishing features.

Distinguished by its stout stems, c. 5 mm thick, elliptic bracteoles 4–5 mm wide and obovate sepals c. 4 mm wide. [Sa’ad 1967: 166; Rechinger 1963: 16]

##### Distribution.

Iran (*Aucher-Eloy* 1407, *Lee* 57); Iraq (*Rawi* 20701, 22867, *Rechinger* 10614, *Kaisi & Yahya* s.n. [19/10/1977]).

##### Notes.

Appears to be mostly sympatric with subsp. reticulatus and might be best treated as as a variety although the differences between the subspecies are relatively substantial.

#### 186. Convolvulus stapfii

Taxon classificationPlantaeSolanalesConvolvulaceae

Rech.f., Ann. Naturhist. Mus. Wien 55: 5. 1947. (Rechinger 1947: 5).

Figure 23, t. 44–49

##### Type.

IRAN, W. Kazerun, *Stapf* 374 (holotype W!).

##### Description.

Perennial herb from a woody rootstock stems to c. 30 cm, the whole plant densely velvety-tomentose of characteristic dark brown colour in herbarium specimens. Basal leaves unknown; stem leaves sessile, 4–7 × 1.2–2 cm, oblong-lanceolate, acute, margin entire, base cuneate, leathery in texture. Flowers in sessile clusters in the axils of bracts along the upper part of the stem; bracts triangular-ovate, 3.5 cm long below but diminishing in size upwards, bracteoles 10 × 1 mm, linear, villous; sepals 10–11 × 3 mm, ovate, finely acuminate to a long aristate point, densely villous; corolla 2–2.2 cm long, colour unknown, unlobed, midpetaline bands adpressed pilose; ovary apically pilose; style glabrous, divided c. 12 mm above base; stigmas (fide Sa’ad 1967) c. 3 mm long. Capsule not seen. [Sa’ad 1967: 172; Nowroozi 2002: 69 (plate), 104 (map)]

##### Distribution.

Endemic to Iran (*Stapf* 1042). Apparently very rare and we have seen no recent collections.

##### Notes.

Shares with *Convolvulus reticulatus* a distinctive indumentum in texture and colour but differs in the sessile flower clusters, pilose ovary, long style and aristate sepals. It is a poorly known species apparently not collected recently.

#### 187. Convolvulus cephalophorus

Taxon classificationPlantaeSolanalesConvolvulaceae

Boiss., Diagn. Pl. Orient. 7: 22. 1846. (Boissier 1846: 22).

Figure 23, t. 16–23

##### Type.

IRAN, *Kotschy* 138 (holotype G; isotypes E!, FHO!, P!).

##### Description.

Perennial herb from a woody rootstock with stems to at least 40 cm, the whole plant densely but very shortly tomentose leaving leaf veins visible; branches somewhat woody. Lower leaves c. 8 × 1.5 cm, oblanceolate, narrowed to a long petiole-like base; stem leaves 2.5–4.5 × 1.5–2.5 cm, ovate, obtuse, margin entire, base cordate and subsessile. Flowers in dense axillary heads, sessile above, shortly pedunculate below; bracts 1.3–2.5 × 1–2.2 cm, ovate, acuminate, basally cordate; peduncles 0–1.5 cm; bracteoles 10–15 × 1–2 mm, linear, long-pilose; pedicels absent; outer sepals 14–16 × 3.5–4 mm, ovate, abruptly narrowed, rounded and with a long, aristate point, long pilose; inner sepals somewhat smaller, c. 12 × 1.5–3 mm; corolla 1.5–1.8 cm, pink, midpetaline bands long-pilose; ovary comose; style glabrous, divided c. 6 mm above base, stigma 3 mm; capsule not seen. [Sa’ad 1967: 155; Nowroozi 2002: 75 (plate), 104 (map)]

##### Distribution.

Endemic to Iran (*Stapf* 373, *Haussknecht* s.n. [4/1868], *Stutz* 984). Apparently rare.

##### Notes.

Sa’ad described the ovary as glabrous but it is comose as in her illustration.

#### 188. Convolvulus oxysepalus

Taxon classificationPlantaeSolanalesConvolvulaceae

Boiss. Diagn. Pl. Orient 7: 23. 1846. (Boissier 1846: 23).

Figure 25, t. 21–27

##### Type.

IRAN, *Aucher-Eloy* 4948 (holotype G; isotype P!).

##### Description.

Perennial undershrub to about 25 cm with stems and branches rigid and somewhat spinescent, tomentellous with matted hairs. Leaves sessile, 1–2.5 × 0.2–0.8 cm, linear-oblanceolate, obovate-elliptic to subspathulate, acute, margin entire, base attenuate, adpressed pubescent with matted hairs. Inflorescence of more or less globose terminal heads; bracts 9–12 × 4–6 mm, broadly ovate, acute; pedicels 0; bracteoles 14 × 1–1.5 mm, lanceolate, acuminate; outer sepals 10–12 × 2.5–3.5mm, oblong-elliptic, acute, densely pilose; inner sepals similar but lanceolate; corolla 1.5–1.8 cm long, colour unknown, midpeline bands pilose; ovary and style glabrous; style divided 3 mm above base, stigmas c. 6 mm long; capsule glabrous. [Sa’ad 1967: 84; Nowroozi 2002: 41 (plate), 101 (map)]

**Figure 25.**
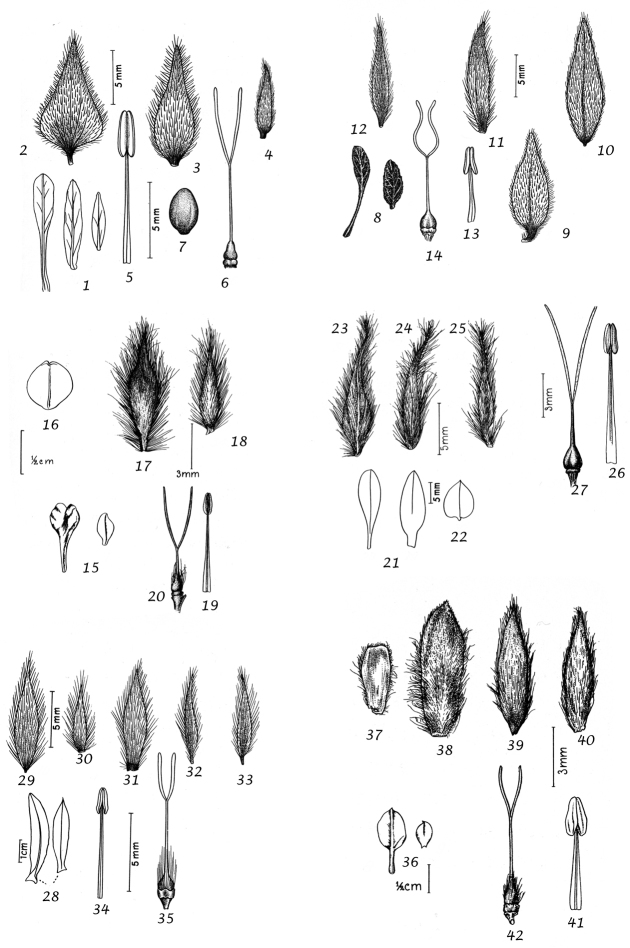
**1–7** *Convolvulus lanatus* **1** leaves **2** bracteole **3** outer sepal **4** inner sepal **5** stamen **6** ovary and style **7** seed. From *Khattam & Scharobeim* 2923 (CAIM) **8–14** *Convolvulus secundus* **8** leaves **9** bracteole **10** outer sepal **11** middle sepal **12** inner sepal **13** stamen; ovary and style **8** from *Eig & Grizl* (CAIM) **9–14** from *Täckholm* s.n. (CAI) **15–20** *Convolvulus ulicinus* **15** leaves **16** bracteole **17** outer sepal **18** inner sepal **19** stamen **20** ovary and style. From *Aucher-Eloy* 3936 (W) **21–27** *Convolvulus oxysepalus* 21leaves **22** bract **23** outer sepal **24** middle sepal **25** inner sepal **26** stamen **27** ovary and style. From *Rechinger* 3211 (E) **28–35** Convolvulus oxyphyllus subsp. oxyphyllus **28** leaves **29** bract **30** bracteole **31** outer sepal **32** middle sepal **33** inner sepal **34** stamen **35** ovary and style. From *Rechinger* 9639 (W) **36–42** *Convolvulus hamrinensis* **36** leaves **37** bracteole **38** outer sepal. From *Rechinger* 8083 (W).

##### Distribution.

Endemic to southern Iran (*Rechinger* 3211, 3864, 4005, *Assadi et al.* 1878, *Popov* 51/201).

##### Notes.

This species has an inflorescence of terminal heads like *Convolvulus scindicus*, but lacks the reddish-brown hairs and the distinctive leaves of that species.

#### 189. Convolvulus ulicinus

Taxon classificationPlantaeSolanalesConvolvulaceae

Boiss., Diagn. Pl. Orient. 7: 26. 1846. (Boissier 1846: 26).

Figure 25, t. 15–20

##### Type.

OMAN, Muscat, *Aucher-Eloy* 4936 (holotype G; isotypes BM000049125!, LE!, P!, W!).

##### Description.

Intricately branched spiny undershrub to c. 30 cm, both the main and side branches spinescent, young stems sericeous. Leaves sessile, (6-) 8–11 × 4–6 mm, obovate or spathulate, apex rounded, margin entire, base attenuate, sericeous especially abaxially. Flowers in dense sessile, axillary clusters of up to 4; bracts 2–4 × 1–3 mm, oblong-elliptic; peduncles absent; bracteoles 1–3 × 1–2 mm, elliptic, apiculate or retuse, sericeous and with spreading hairs; outer sepals 5–6 × 2 mm, narrowly elliptic, acute, densely covered in long woolly hairs; inner sepals similar but smaller, c. 4 × 1 mm; corolla 11 mm long, white, midpetaline bands woolly; ovary apically pilose; style glabrous, divided 2–3 mm above base; stigmas 6 mm. Capsule not seen. [Sa’ad 1967: 78; Jongbloed 2003: 315 (photo)]

##### Distribution.

Almost restricted to Oman (*Mandaville* 3446, 6230; *Popov* 57/86, *Lebrun* 91, *McLeish* 3561, *Edmondson* 3285); United Arab Emirates (fide Jongbloed 2003: 315).

##### Notes.

A little-known species similar to *Convolvulus acanthocladus*, differing in the obovate leaves, flowers arranged in sessile, axillary clusters and elliptic bracteoles. The corolla size may also be significant.

#### 190. Convolvulus scindicus

Taxon classificationPlantaeSolanalesConvolvulaceae

Stocks, Hooker’s J. Bot. Kew Gard. Misc. 4: 173. 1852. (Stocks, 1852: 173).

Convolvulus brachyphyllus Boiss., Diagn. Pl. Orient., ser. 2, 3: 122. 1856. (Boissier 1856: 122). Type. “Scinde” [Sind], *Stocks* s.n. (G-BOIS?).

##### Type.

PAKISTAN, Sind, *Stocks* 433 (holotype K!).

##### Description.

Perennial undershrub with rigid but not spinescent branches, 20–60 cm high, branches softly grey-tomentellous. Leaves alternate or more or less clustered, sessile, coriaceous, 5–15 × 4–6 mm, obovate or elliptic, rounded to obtuse, margin undulate, gradually narrowed at base, grey or rufous-tomentose, veins strongly impressed. Inflorescence of sessile terminal heads covered in reddish-brown hairs; bracteoles 6–7 × 4 mm, obovate-elliptic, lacking veins, villous; sepals 6–7 × 1.5 mm, lanceolate, densely reddish-brown villous; corolla 10–12 mm long, white, shallowly lobed, midpetaline bands pubescent; ovary and style glabrous; capsule glabrous, apparently always 1-seeded; seeds glabrous, smooth. [Austin and Ghazanfar 1979: 17]

##### Distribution.

Probably endemic to Pakistan (*Lamond* 810, *Jaffri* 3712, *Rechinger* 28498). Records from India (Bhandari 1978: 248) require confirmation.

##### Notes.

Very distinctive because of the reddish-brown-haired terminal heads combined with the woody, but not spinescent, branches and the small, often clustered leaves with strongly impressed veins.

## Excluded and poorly understood species

The following is a list of species placed in *Convolvulus* in recent literature such as Austin et al. (2012) and here treated as belonging to other genera together with names of species correctly placed in *Convolvulus* but not treated in the main text because their exact identity is unknown or they were never correctly published. Historically many species of Convolvulaceae were originally described in *Convolvulus* but have long since been transferred to other genera. Most of these names and their modern equivalents, where known, can be found in Staples (2014).

*Breweria montevidensis* Peter, Nat. Pflanz. Fam. [Engler & Prantl] 4 (3a): 16. 1891. Uncertain species with no type at GOET. Probably = Convolvulus crenatifolius subsp. montevidensis (Spreng.) J.R.I.Wood & R.W.Scotland and possibly based on. *Convolvulus montevidensis* Spreng. although this is not cited as basionym (Staples et al. 2012).

*Bucharea atlantica* Raf., Fl. Tellur. 4: 84. 1838. = Unidentified *Convolvulus* sp. from North Africa.

*Convolvulus adscendens* Steud., nom nud. (Boissier 1875b: 100) = *Convolvulus reticulatus* Choisy

*Convolvulus argenteus* Lam., Fl. Franç. (Lamarck) 2: 266. 1779. No type known; unlikely to be *Convolvulus cneorum* L. (unless cultivated) but could be an older name for *Convolvulus lanuginosus* Desr.

*Convolvulus argyrophyllus* Hoffmans., Verz. Pfl.-Kult. 53. 1824, nom. nud. = Convolvulus althaeoides subsp. tenuissimus (Sm.) Batt.

*Convolvulus aridus* Greene, Pittonia 3(19C): 330. 1898. = Calystegia macrostegia subsp. arida (Greene) Brummitt

Convolvulus arvensis var. angustatus Ledeb., Fl. Altaic. [Ledebour] 1: 225. 1829. Type. Russia, Altai, Rivers Ulegumen and Katunja (LE?, not seen) uncertain species of *Convolvulus* ? = *Convolvulus chinensis* Ker-Gawl.

Convolvulus arvensis var. biflorus Pau, in Font Quer, Iter Marocc. 525. 1930, nom illeg., non Choisy (1845). Species in Pau’s Iter Maroccanum were not validly published in that work.

*Convolvulus arvensis var. cordifolius* Lasch, Linnaea 4: 407.1829, nom. nud. = *Convolvulus arvensis* L.

Convolvulus arvensis var. minutus Maire, Cat. Pl. Maroc 3: 587. 1934, nom nud.; no description has been traced.

Convolvulus arvensis var. sagittifolius Turcz., Bull. Soc. Imp. Naturalistes Moscou 11: 97. 1838, nom. nud. = *Convolvulus chinensis* Ker-Gawl.

Convolvulus arvensis var. vulgaris Ledeb., Fl. Altaic. [Ledebour] 1: 224. 1829, no type specified ? = *Convolvulus arvensis* L.

Convolvulus arvensis var. volubilis Ledeb., Fl. Altaic. [Ledebour] 1: 224. 1829. Type. Russia, Altai, Alexandrowsk (LE?, not seen) ? = *Convolvulus chinensis* Ker-Gawl.

*Convolvulus binghamiae* Greene = Calystegia sepium subsp. binghamiae (Greene) Brummitt

*Convolvulus burmannii* Choisy, Prodr. [A.P. de Candolle] 9: 405. 1845, no type at G, possibly = *Convolvulus ocellatus* Hook.

*Convolvulus cantaber* Pall., Tableau physique et topografique de la Tauride St Petersburg, 1-59 1795.and Nov. Act. Ac. Sc. Petr 10: 257–320. 1797. Possible spelling error for *Convolvulus cantabrica* L.

Convolvulus cantabrica subsp. atlantis Emb., Bull. Soc. Sci. Nat. Maroc 15: 214 (1936), nom. inval. (no Latin description) = *Convolvulus mazicum* Emb. & Maire

*Convolvulus capensis var. bowienanus* (Rendle) A.Meeuse = *Merremia* sp. (probably).

Convolvulus chondrilloides var. villosus Bornm., Repert. Spec. Nov. Regni Veg.51: 215.1942. (Bornmüller and Gauba 1942: 215). nom. illeg, no Latin diagnosis.

*Convolvulus cupanianus* Todaro, Ann. Sci. Nat., Bot., ser. 4, 20: 304. 1863, nom. nud.. = Convolvulus tricolor subsp. cupanianus (Todaro ex Batt. & Trab.) Cavara & Grande

*Convolvulus cyclostegius* House = Calystegia macrostegia subsp. cyclostegia (House) Brummitt

*Convolvulus dasycephalus* Pall., nom. nud = ? *Convolvulus calvertii* Boiss.

*Convolvulus denudatus* Petrov, Byull. Moskovsk. Obshch. Isp. Prir., Otd. Biol., n.ser., 44: 132 (1935). nom. nud., sin descr. = *Convolvulus hamadae* (Vved.) Petrov.

*Convolvulus el-arishensis* Boulos, Bull. Fac. Sci., Cairo Univ. 34: 77. 1958, not validly published, no type cited. = *Convolvulus lanatus* Vahl

Convolvulus erubescens var. dilatatus Choisy, Prodr. [A.P. de Candolle] 9: 412. 1845 = *Convolvulus eyreanus* R.W.Johnson, fide Johnson (2001: 20).

*Convolvulus flavus* Willd. = *Merremia hederacea* (Burm.f.) Hallier f.

*Convolvulus fulcratus* (A.Gray) Greene = Calystegia occidentalis subsp. fulcratus (A.Gray) Brummitt

*Convolvulus geniculatus* Munby ex Pomel, Nouv. Mat. Fl. Atl. 1: 85. 1874, nom nud. = unknown species of *Convolvulus*.

*Convolvulus glaucifolius* (L.) Spreng. = *Ipomoea glaucifolia* L., nom rej., a species of *Convolvulus* of uncertain identity. See Staples et al. in Taxon 55: 535 (2006).

*Convolvulus hastatus* Sieber (Choisy, 1845: 412) = *Convolvulus fatmensis* Kunze

*Convolvulus heterophyllus* Ehrenb., nom. nud. (Boissier 1875b: 109) = *Convolvulus coelesyriacus* Boiss.

*Convolvulus involucellatus* Klotzsch, Naturw. Reise Mossambique [Peters] 6(Bot., 1): 241. 1861 = possibly *Convolvulus jefferyi* fide Verdcourt (1982: 463), but type destroyed in Berlin in 1943 and name may be illegitimate.

*Convolvulus japonicus* Thunb. = *Calystegia hederacea* Wall.

*Convolvulus jemensis* Kotschy = unknown species, neither protologue nor specimen can be traced.

*Convolvulus linoides* Bornm. = *Seddera virgata* Hochst. & Steud. ex Hochst.

*Convolvulus longipes* S.Watson = *Calystegia longipes* (S. Watson) Brummitt

*Convolvulus macrostegius* Greene = *Calystegia macrostegia* (Greene) Brummitt

*Convolvulus malacophyllus* Greene = *Calystegia malacophylla* (Greene) Brummitt

Convolvulus mazicum var. atlantis Sauvage & Vindt, Fl. Maroc 2: 38 (1954), not validly published; no type or Latin description. = *Convolvulus mazicum* Emb. & Maire

*Convolvulus mollisimus* Pall., Prodr. [A.P de Candolle] 9: 403. 1845. nom. nud. = *Convolvulus holosericeus* M.Bieb.

*Convolvulus nodiflorus* Desr. = *Jacquemontia nodiflora* (Desr.) G.Don

*Convolvulus nyctagineus* Greene = Calystegia atriplicifolia Hallier f. subsp. buttensis Brummitt in part

*Convolvulus occidentalis* A.Gray = *Calystegia occidentalis* (A.Gray) Brummitt

*Convolvulus peirsonii* Abrams = *Calystegia occidentalis* (A.Gray) Brummitt

Convolvulus pilosellifolius var. orreanus Murb. (Maire 1936: 251). Appears to be a nom. nud. = *Convolvulus prostratus* Forssk.

*Convolvulus polymorphus* Greene = *Calystegia occidentalis* (A.Gray) Brummitt

*Convolvulus procumbens* Pall., Syst. Veg, ed. 15 bis [Roemer & Schultes] 4: 298. 1819, nom. nud. = *Convolvulus holosericeus* M.Bieb.

*Convolvulus pusillus* Pall., Syst. Veg, ed. 15 bis [Roemer & Schultes] 4: 295. 1819 nom. nud. = *Convolvulus calvertii* Boiss.

*Convolvulus rozynskii* (Standl.) Lewis & Oliver = *Jacquemontia rozynskii* Standl.

*Convolvulus rubescens* Poir., Encycl. [Lamarck et al.] Suppl.3: 466 (1814) = *Convolvulus erubescens* Sims

*Convolvulus rupestris* Buch, Phys. Beschr. Canar. Ins. 193. 1828 [1825]. (Buch 1828: 193) = Unknown species of *Convolvulus*.

*Convolvulus salonifolius* Lowe, Trans. Cambridge Philos. Soc. 4: 22. 1831 = Unknown species of *Convolvulus*.

Convolvulus schimperi var. ellipticus Post, Fl. Syria: 561. 1896. from Gaza with no cited type, most likely = *Convolvulus secundus* Desr.

*Convolvulus sepium* L. = *Calystegia sepium* (L.) R.Br.

*Convolvulus soldanella* L. = *Calystegia soldanella* (L.) R.Br.

*Convolvulus spithameus* L. = *Calystegia spithameus* (L.) Pursch.

*Convolvulus subacaulis* (Hook. & Arn.) Greene = *Calystegia subacaulis* Hook. & Arn.

*Convolvulus undulatus* Aucher, nom. nud. (Choisy 1845: 400) = *Convolvulus reticulatus* Chosy

Convolvulus valentinus var. suffruticosus (Desf.) Pau & Font Quer, Iter Marocc. 489. 1927: comb. ined. = *Convolvulus valentinus* Cav.

*Convolvulus tomentellus* Greene = *Calystegia occidentalis* (A.Gray) Brummitt

*Convolvulus tournefortii* Sieber ex Spreng., Syst. Veg. 1: 611. 1824, nom. nud. = *Convolvulus cantabrica* L.

*Convolvulus translucens* Hance, J. Bot. 7: 165. 1869; Type. *Williams* 14690 (BM001014564, fragment) from Mongolia, = ? *Convolvulus arvensis* L.

Convolvulus turcumanicus var. villosus Sa’ad, Meded. Bot. Mus. Herb. Rijks Univ. Utrecht 281: 108. 1967, not validly published, no type cited = *Convolvulus eremophilus* Boiss. & Buhse

*Convolvulus wallichianus* Spreng. = *Calystegia hederacea* Wall.

*Evolvulus ferrugineus* Wall., Numer. List (Wallich) n. 1316. 1829, nom. nud. = *Convolvulus prostratus* Forssk.

*Retzia pilosa* Rottler ex Choisy, Mém. Soc. Phys. Genève 6: 477. 1834, nom. nud. = Convolvulus rottlerianus Choisy subsp. rottlerianus.

## Index of names

Aniseia costata 113

diversifolia 107, 108

Bonamia althoffiana 83

Breweria fastigiata 168

hassleriana 118

minima 155

montevidensis 251

scoparia 231

spinosa 168

Bucharea atlantica 251

maderensis 136

Calystegia glabrata 83

Convolvulus abdallahi 224

acanthocladus 21, 22, 27, 28, 45, 48, 202-204, 250

acaulis 126

acicularis 179

adscendens 126, 251

afanassievii 187

affghanus 201

agrestis 149, 150

agrophilos 73

aitchisonii 20, 22, 24, 49, 247

alceifolius 80, 85

aleppensis 69

althaeoides 13, 14, 17, 20, 21, 23, 30, 38, 39, 40, 42, 57, 74, 79-82, 86, 252

althaeoides subsp. althaeoides 40, 42, 74, 79

althaeoides subsp. darnitanus 81

althaeoides subsp. tenuissimus 21, 40, 42, 81, 252

althaeoides var. albidiflorus 80

althaeoides var. angustisectus 80

althaeoides var. argyreus 80

althaeoides var. dissectus 80

althaeoides var. ferrugineus 80

althaeoides var. hirsutus 80

althaeoides var. pedatus 81

althaeoides var. repandus 80

althaeoides var. sericeus 80

ambigens 61

amblycalyx 65

ammannii 20, 21, 26-28, 53, 54, 188, 195-197

ammocharis 177

Convolvulus ammophilus 177

amoenus 69

anatolicus 226

andinus 108

angolensis 97, 98, 100

angustissimus 22, 28, 31, 109, 119, 125-127

angustissimus subsp. angustissimus 126, 127

angustissimus subsp. fililobus 126, 127

angustissimus subsp. omnigracilis 126

angustissimus subsp. peninsularum 126

arabicus 163, 164

arcuatus 150

argenteus 227, 228, 251

argillicola 23, 24, 32, 82, 84, 90

argyracanthus 21, 27, 48, 198, 201

argyraeus 80

argyrophyllus 252

argyrothamnos 13, 27, 41, 220

aridus 252

armatus 166

armenus 69

arvensis 12, 14, 16, 20-22, 24, 29-32, 34, 39, 40, 42, 45, 47, 51, 54, 56, 58, 60-65, 70, 110, 120, 218, 252, 254

arvensis subsp. crispatus 62

arvensis var. angustatus 252

arvensis var. aphacoefolius 61

arvensis var. auriculatus 61

arvensis var. biflorus 61, 252

arvensis var. cordifolius 252

arvensis var. crassifolius 63

arvensis var. crispatus 62

arvensis var. erectus 64

arvensis var. filicaulis 61

arvensis var. hastulatus 61

arvensis var. linearifolius 61

arvensis var. minutus 252

arvarvensis var. multiflorus 61

arvensis var. obtusifolius 60

arvensis var. paui 62

arvensis var. pumilus 60

arvensis var. sagittatus 63

arvensis var. sagittifolius 252

arvensis var. trigonophyllus 62

Convolvulus arvensis var. villosus 61

arvensis var. volubilis 252

arvensis var. vulgaris 252

ascendens 126

aschersonii 14, 23, 24, 26, 32, 35, 45, 82, 92-97, 101, 108

askabadensis 50, 53, 188, 191, 194

assyricus 17, 20, 23, 24, 26-28, 44, 224, 225

asyrensis 20, 22, 27, 28, 46, 240, 246

atriplicifolius 67

aucheri 23, 24, 26, 27, 43, 203, 207

auricomus 158, 163, 164

auricomus var. ferrugineus 164

auricomus var. volubilis 163

auriculatus 60, 61

austroaegyptiacus 174, 176

austroaegyptiacus var. cancerianus 175

austroafricanus 18, 19, 32, 35, 82, 89, 90, 92-95, 97, 101

beguinotii 150

beluchistanensis 245

benehoavensis 232

besseri 217

betonicifolius 20, 24, 27, 40, 47, 52, 67-71

betonicifolius subsp. betonicifolius

betonicifolius subsp. peduncularis 69

betonicifolius var. armenus 69

bicuspidatus 63

bidentatus 22, 26, 32, 82, 86, 87

bidrensis 20, 22, 24, 34, 160, 161

binghamiae 252

blatteri 156

boedeckerianus 23, 32, 89, 90

boissieri 20, 22, 27, 40, 41, 44, 214-216, 222, 223

boissieri subsp. boissieri 41, 214, 216, 222

boissieri subsp. compactus 22, 41, 44, 214-216, 222, 223

boissieri subsp. parnassicus 215

boissieri subsp. suendermannii 216

bonariensis 23, 24, 29, 82, 107, 113

bonariensis var. multiflorus 107

bornmuelleri 177

bourgaei 136

brachyphyllus 251

bracteosus 211

brevipes 145

Convolvulus brevispinosus 199

bryonifolius 79

bullerianus 24, 25, 33, 102

burmannii 252

bushiricus 46, 50, 240, 241, 245, 246

bussei 100

calvertii 20-23, 26-28, 44, 208-212, 226, 252, 253

calvertii subsp. bracteosus 211

calvertii subsp. calvertii 41, 49, 52, 210, 211

calvertii subsp. ruprechtii 41, 52, 211

calvertii subsp. tauricus 210

calvertii var. tauricus 210

calycinus 102

campanulatus 131

canariensis 20, 25-27, 39, 134, 136, 138, 141

canariensis var. massonii 136

cancerianus 174, 175, 176

canescens 106, 107, 227

cantaber 252

cantabrica 17, 23, 24, 26-28, 44, 208-212. 226, 252, 253

cantabrica subsp. atlantis 252

cantabrica subsp. mazicum 221

cantabrica subsp. medius 206

cantabrica subsp. radicosus 223

cantabrica subsp. subsericeus 186

cantabrica var. mazicum 221

cantabrica var. terrestris 205

cantabrica var. villosus 206

capensis 14, 23, 24, 26, 33, 82, 84-88, 252

capensis var. bowienanus 252

capensis var. dissectus 85

capensis var. malvifolius 85

capensis var. plicatus 85

capitatus 227

capituliferus 20, 22, 25, 34, 158-160

capituliferus subsp. capituliferus 158

capituliferus subsp. foliaceus 159

capituliferus var. foliaceus 159

capituliferus var. suberectus 158

cappadocicus 59

caput-medusae 21, 23, 27, 38, 172, 231

cardiosepalus 205

carduchorum 20, 24, 44, 195, 222, 226

carrii 13, 21, 24, 26, 30, 103, 105, 106

cassius 20, 27, 42, 57, 68, 71

Convolvulus cataonicus 20, 24, 27, 44, 222, 224, 226

cateniflorus 235

cephalantha 83

cephalophorus 22, 24, 27, 49, 240, 249

cephalopodus 19, 22, 24, 26-28, 46, 50, 240, 241, 244-246

cephalopodus subsp. abhansis 246

cephalopodus subsp. bushiricus 46, 50, 240, 241, 245, 246

cephalopodus subsp. cephalopodus 46, 50, 240, 245

chamaerhacos 207

chaudharyi 177, 178

cherleri 60, 61

chilensis 23, 25, 26, 29, 103, 105, 106, 108

chinensis 14, 20, 22-24, 52, 54, 58, 62-64, 252

chinensis subsp. chinensis 52, 63

chinensis subsp. triangularis 23, 52, 64

chondrilloides 21, 24, 26-28, 43, 50, 171, 178-179, 252

chondrilloides subsp. eriocalycinus 179

chondrilloides var. burzianus 179

chondrilloides var. chondrilloides 179

chondrilloides var. villosus 252

ciliatus 154

clementii 24, 31, 119, 122-125

clementii var. biflorus 122

cneorum 13, 21, 23, 26-28, 43, 50, 171, 178-179

cneorum subsp. latifolius 229

cneorum var. cneorum 225, 228

cneorum var. latifolius 225, 229

cochlearis 214

coelesyriacus 20, 24, 28, 41, 57, 74, 75, 253

commutatus 22-24, 27, 28, 49, 53, 207, 209, 212

compactus 22, 41, 44, 214-216, 222, 223

compactus subsp. parnassicus 215

cordifolius 96

corsicus 60, 61

costatus 113

crenatifolius 24, 25, 29, 30, 67, 105, 113, 115-117, 251

crenatifolius subsp. crenatifolius 30, 105, 115

Convolvulus crenatifolius subsp. montevidensis 25, 29, 105, 116, 117, 251

crenatifolius var. argentinicus 116

crenatifolius var. megapotamicus 116

crenatifolius var. montevidensis 116

crenatifolius var. peruvianus 115

crenatus 113

crispifolius 20, 21, 24, 28, 31, 109, 119, 121, 122

cupanianus 40, 153, 154, 252

cyclostegius 252

cyprius 218, 219

damascenus 72

dasycephalus 252

demissus 14, 22, 25, 29, 82, 108

demissus var. andinus 108

demissus var. ovatus 108

densiflorus 156

denudatus 252

deserti 174, 175

deserticulus 187

despreauxii 232

dianthoides 193, 194

dinteri 91

dissectus 106-108

dissectus var. canescens 107

dissectus var. diversifolius 108

divaricatus 13, 20, 27, 28, 51, 54, 186-189

diversifolius 137

dorycnioides 205

dorycnium 13, 20, 24, 28, 36, 41, 43, 51, 53, 189, 225, 229-231

dorycnium subsp. dorycnium 43, 225, 229

dorycnium subsp. oxysepalus 43, 51, 225, 230

dorycnium subsp. subhirsutus 13, 51, 53, 230, 231

dorycnium var. oxysepalus 230

dorycnium var. turcomanicus 189

dregeanus 22, 23, 25, 32, 84, 88

dryadum 20, 23, 130, 132

dryadum var. tazekkensis

durandoi 20, 22, 24, 25, 26, 58, 59

eichwaldii 199

el-arishensis 252

elegantissimus 81, 82

elongatus 57, 149, 150

elymaiticus 22, 23, 27, 49, 209

Convolvulus ensifolius 13, 22, 29, 118

equitans 23, 25, 26, 30, 103-106, 113

equitans var. equitans 104, 106

equitans var. lindheimeri 30, 104

eremophilus 13, 19, 20, 24, 26-28, 51, 53, 54, 182, 183, 186, 187, 189-191, 254

erinaceus 20, 21, 24, 25, 27, 28, 46, 51, 53, 169, 183-186, 189

erinaceus var. kermanensis 185, 189

erosus 113

erubescens 17, 31, 119, 125, 126, 252, 253

erubescens var. albus 126

erubescens var. angustissimus 126

erubescens var. dilatatus 252

erubescens var. fililobus 126

euphraticus 8, 22, 50, 240, 246

europaeus 61

euxinus 206

evolvuloides 154, 174

excelsus 184, 185

eyreanus 20, 21, 28, 31, 119, 122, 252

falkia 85

farinosus 20, 23, 24, 27, 30, 32, 35, 39, 40, 45, 82, 92, 94, 96, 116

fastigiatus 168

fatmensis 20, 24, 25, 28, 33, 35, 42, 44, 45, 47, 57, 58, 65, 253

faurotii 163

fernandesii 13, 20, 40, 140,

filiformis 85

filipes 181

fischerianus 63

flavus 66, 252

flexuosus 149

floridus 13, 17, 20, 23, 24, 26-28, 38, 225, 232, 233

floridus var. angustifolius 233

floridus var. densiflorus 233

floridus var. virgatus 232

forskalei 238

fractosaxosus 127

frutescens 199

fruticans 199

fruticosus 21, 24, 27, 28, 48, 52, 55, 140, 198-200

fruticulosus 20, 23, 25-27, 39, 134, 138-140

fruticulosus subsp. fruticulosus 39, 139

Convolvulus fruticulosus subsp. glandulosus 140

fruticulosus var. glandulosus 140

fulcratus 253

galaticus 20, 21, 27, 43, 57, 73-75

galpinii 33, 101, 102

geniculatus 125, 253

genistoides 201

georgicus 142, 143

geranioides 111

gerardii 217

germaniciae 20, 21, 24, 27, 28, 43, 74, 75

gharbensis 20, 23, 25, 144, 146

gilbertii 172, 173

glandulosus 140

glaouorum 20, 23, 28, 74, 76-79

glaucifolius 253

glomeratus 22, 24, 25, 34, 37, 42, 45, 48, 148, 156, 158, 162-164

glomeratus var. glomeratus 163

glomeratus var. gymnospermus 164

glomeratus var. sachalitarum 24, 164

glomeratus var. sericeus 163

glomeratus var. volubilis 163

gonocladus 236, 237

gortschakovii 21, 24, 27, 52, 54, 198, 200

gracilis 79

gracillimus 20, 21, 24, 26, 27, 51, 188, 192, 207

graminetinus 28, 31, 120, 121

grantii 20, 22-24, 26, 34, 46, 169, 180, 181

grigorjevii 21, 23, 27, 52, 53, 196

hamadae 13, 20, 21, 27, 51, 53, 184, 185, 252

hamphilahensis 156, 158

hamrinensis 22, 23, 27, 46, 49, 234-236, 243

hasslerianus 19-22, 24, 29, 103, 117, 118

hastatus 60, 86, 94, 98, 253

hastatus var. major 86

hastatus var. multiflorus 94

hastatus var. natalensis 98

hastifolius 60

haussknechtii 236, 237

hermanniae 21, 26, 29, 30, 67, 103, 109, 111-113

hermanniae subsp. erosus 26, 29, 103, 109, 111, 113

hermanniae subsp. hermanniae 26, 112

hermanniae var. elongatus 113

Convolvulus hermanniae var. viridis 113

hermannioides 104

heterophyllus 253

heterotrichus 174

hildebrandtii 20, 23, 24, 26, 28, 34, 47, 180-182

hilsenbergianus 96

hirsutus 67, 69, 80

hirsutus var. tomentosus 69

hirsutus var. virescens 69

hirtellus 98-100

holosericeus 20, 21, 24, 26-28, 41, 44, 49, 52, 208, 212-213, 215-216, 253

holosericeus subsp. holosericeus 44, 213, 215

holosericeus subsp. macrocalycinus 44, 213, 216

holosericeus var. macrocalycinus 213

huber-morathii 224

huegelii 120

huillensis 97, 98

humilis 20, 23-26, 40, 41, 144, 150, 154, 217

hystrix 22, 23, 25, 27, 28, 33, 34, 45, 148, 165-168, 170

hystrix forma inermis 167

hystrix subsp. dhofaricus 166

hystrix subsp. hystrix 33, 148, 166

hystrix subsp. inermis 34, 167

hystrix subsp. ruspolii 33, 167

ifniensis 170

incanus 61, 104, 112

incanus var. glabratus 61

incisodentatus 29, 114

incisus 114

inconspicuus 85, 86

infantispinosus 235, 236

intermedius 217

involucellatus 253

iranicus 18, 21, 22, 24, 27, 48, 202-204

italicus 80

japonicus 253

jefferyi 20-22, 25, 34, 157-161, 253

jemensis 253

jordanensis 19, 22, 26, 43, 241, 242, 244

keniensis 83

kilimandschari 17, 20, 24, 27, 33, 82-84

kilimandschari var. glabratus 83

koieanus 20, 26, 27, 51, 183, 192

Convolvulus konyacus 215

korolkowii 187, 189, 190

korolkowii var. michelsonii 187

kossmatii 15, 19, 21, 23, 25, 33, 45, 155, 168, 169

kotschyanus 22, 24, 26, 50, 236, 242, 245

krauseanus 21, 23, 27, 52, 53, 196, 199

kurdistanicus 20, 50, 192

laciniatus 22, 25, 29, 109-111, 114

laciniatus var. hirsutus 110

laciniatus var. peduncularis 111

lanatus 21, 22, 24, 26, 28, 43, 236, 238, 239, 243, 252

lanjouwii 19, 20, 24, 28, 47, 130, 133, 135

lanuginosus 21, 23, 26, 28, 41, 67, 195, 210, 212, 225, 227, 247, 251

lanuginosus subsp. sericeus 227

lanuginosus var. sericeus 227

lanuginosus var. villosus 227

lasianthus 110

lasiophlaeus 129

leiocalycinus 21, 24-28, 45, 48, 52, 128-131

leiocalycinus var. glaber 131

leiocalycinus var. leiocalycinus 130

leiocalycinus var. retrosepalus 130, 131

leiocalycinus var. stocksii 131

leoicalycinus var. lycioides 131

leptocladus 20, 23, 24, 26, 51, 182, 183, 192

leptocladus subsp. glabrisepalus 192

leptosepalus 206

leucochnous 77

leucotrichus 145

libanoticus 17, 20, 26-28, 40, 44, 222, 223

lilloi 24, 29, 117, 118

lindbergii 19-21, 27, 51, 183, 191

linearifolius 205

linearis 178, 205, 219

lineatus 14, 20, 21, 23, 24, 27, 28, 41, 44, 49, 52, 54, 208, 216-219, 221

lineatus var. angustifolius 218

lineatus var. minutus 217

lineatus var. pentapetaloides 217

liniformis 88

linoides 253

littoralis 158

longipedicellatus 20, 42, 68, 70

longipedunculatus 189, 190

Convolvulus longipes 253

longipilis 205

lopezsocasii 20, 22, 24, 26, 27, 39, 138

lycioides 129, 131

macrostegius 253

maireanus 20, 23, 25, 27, 130, 133

mairei 13, 20, 24, 27, 28, 40, 58, 64

malacophyllus 253

malcolmii 60

maroccanus 153

mascatensis 162

massonii 20, 24, 26, 27, 39, 134-136, 141

mauritanicus 143

mazicum 17, 20, 21, 27, 221, 222, 252, 253

mazicum var. atlantis 253

meonanthus 20, 25, 36, 40, 144, 151

megapotamicus 116

mesatlanticus 77, 78

michelsonii 187

micranthus 96

microphyllus 173, 174, 176

microphyllus var. boissieri 174

microphyllus var. heterotrichus 174

microphyllus var. longipes 174

microphyllus var. macra 174

microsepalus 22, 24, 28, 31, 109, 119

minor 60, 152

modestus 209

mollisimus 253

mollis var. albidovillosus 113

montanus 22, 24, 25, 29, 109, 114

montevidensis 116, 117, 251

montevidensis var. megapotamicus 116

montevidensis var. ottonis 116

multifidus 24, 32, 86, 89-91

namaquensis 14, 22, 26, 33, 82, 87

natalensis 24, 25, 33, 87, 88, 92, 98, 102, 103

natalensis var. angustifolia 87

natalensis var. integrifolia 102

natalensis var. transvaalensis 88

neglectus 186

nitens 217

nitidus 214

nodiflorus 253

nyctagineus 253

occidentalis 253, 254

Convolvulus ocellatus 22, 23, 26, 32, 89, 91-93, 252

ocellatus var. ornatus 91

ocellatus var. plurinervius 91

oleifolius 21, 23, 26-28, 37, 41, 176, 208, 216, 219-221

oleifolius subsp. scopulorum 219

oleifolius var. angustifolius 216, 220

oleifolius var. deserti 220

oleifolius var. oleifolius 219

oleifolius var. pauciflorus 219

oleifolius var. pumilus 176, 220

oleifolius var. scopulorum 219

olgae 129, 131

omanensis 245

oppositifolius 22, 24, 25, 45, 157, 165

ornatus 91

orophilus 226

ottoensis 116

ottonis 116, 117

ovatus 108, 147

oxyphyllus 13, 21-23, 27, 45, 46, 48, 49, 234-236, 243

oxyphyllus subsp. cateniflorus 234, 235

oxyphyllus subsp. oxycladus 46, 48, 235

oxyphyllus subsp. oxyphyllus 45, 48, 234, 243

oxyphyllus subsp. sheilae 235

oxysepalus 14, 21-24, 26, 43, 48, 49, 51, 205, 225, 229, 230, 243, 250

palaestinus 20, 23, 25, 27, 28, 38, 42, 57, 68, 72, 73

palaestinus var. diversifolius 72

palaestinus var. stenophyllus 72, 73

pallidus 136

pannifolius 136

paradoxus 97

parnassicus 214, 215

parviflorus 147

parvifolius 173

peduncularis 69

pedunculatus 69

peirsonii 253

penicillatus 96

peninsularis 18, 20, 23, 24, 26, 46, 182, 183

pentapetaloides 20, 25, 26, 28, 36, 40, 42, 47, 51, 144, 150-152, 154, 155, 217

perraudieri 139, 140

Convolvulus persicus 17, 20, 22, 24, 25, 39, 42, 48, 52, 128, 130, 132

peshmenii 215

phrygius 20, 27, 44, 221-223

phyllosepalus 98, 99

pilosellifolius 13, 14, 22-24, 26, 28, 36, 43, 44, 46, 50, 53, 169, 171, 175-178, 206, 231, 253

pilosellifolius var. leiocalycinus 177

pilosellifolius var. linearifolius 178

pilosellifolius var. orreanus 253

pilosellifolius var. pilosellifolius 177

piloselloides 177

pitardii 20, 23, 28, 57, 74, 76-79

pitardii var. glaouorum 77

pitardii var. leucochnous 77

pitardii var. pitardii 77

plicatus 85

pluricaulis 173, 174, 176

pluricaulis var. macra 174

polymorphus 253

preissii 120

procumbens 253

prostratus 17, 21-24, 26, 28, 34, 36, 38, 46, 49, 50, 60, 169, 171, 173-176, 237, 253, 254

prostratus var. boissieri 174

prostratus var. deserti 174, 175

pseudocantabrica 20, 27, 28, 50, 53, 54, 188, 191, 193-194

pseudocantabrica subsp. askabadensis 188, 191, 194

pseudocantabrica subsp. dianthoides 193

pseudocantabrica subsp. pseudocantabrica 50, 53, 193

pseudocantabrica var. dianthoides 194

pseudocompactus 216

pseudoscammonia 12, 20, 22, 24, 25, 42, 57, 59

pseudosiculus 142, 149, 150

pseudosiculus var. multiflorus 142

pubescens 67

pulvinatus 221, 223

pungens 200

pusillus 253

pyrrotrichus 22, 24, 26, 50, 236, 237, 242, 245

quadriflorus 72

Convolvulus quinqueflorus 96

radicosus 223

randii 19-21, 23, 32, 35, 91, 92

rectangularis 19, 20, 24, 47, 135

recurvatus 20, 24, 28, 31, 119, 123-125

recurvatus subsp. nullarborensis 124

recurvatus subsp. recurvatus 124

refractus 149

remotus 20, 22, 31, 120, 127

reticulatus 22, 26, 28, 43, 50, 242, 248-249, 251, 254

reticulatus subsp. reticulatus 50, 242, 248

reticulatus subsp. waltherioides 50, 248

retrosepalus 130, 131

rhynchophyllus 94

rhyniospermus 20, 22, 25, 33, 35, 47, 148, 156, 158, 159

rhyniospermus var. leavis 156

rottlerianus 20, 28, 33, 47, 169, 171-173, 176, 254

rottlerianus subsp. rottlerianus 47, 171, 172

rottlerianus subsp. stocksii 47, 173

rottlerianus var. tenellus 173

rozynskii 253

rubescens 253

rufescens 20, 23, 24, 48, 66

rupestris 253

ruprechtii 211

ruspolii 167

sabatianus 20, 25, 26, 37, 40, 134, 142-143

sabatius subsp. mauritanicus 143

sabatius subsp. sabatius 40, 142

sabatius var. atlanticus 143

sabatius var. mauritanicus 143

sagittatus 14, 17-19, 23, 27, 32, 33, 35, 37, 45, 82, 87, 92, 94-100, 110

sagittatus subsp. grandifolius 99

sagittatus subsp. grandifolius var. linearifolius 94, 96

sagittatus subsp. sagittatus var. hirtellus 99

sagittatus subsp. sagittatus var. phyllosepalus 99

sagittatus subvar. abyssinicus 96

sagittatus subvar. australis 98

sagittatus subvar. graminifolia 98

sagittatus subvar. linerifolius 94

sagittatus subvar. subcordata 98

Convolvulus sagittatus subvar. villosa 100

sagittatus var. abyssinicus 96

sagittatus var. aschersonii 95

sagittatus var. graminifolius 98

sagittatus var. grandiflorus 98, 99

sagittatus var. grandiflorus subvar. graminifolia 98

sagittatus var. grandiflorus subvar. linearifolia 94

sagittatus var. grandiflorus subvar. subcordata 98

sagittatus var. hirtellus 99

sagittatus var. latifolius 98

sagittatus var. linearifolius 94

sagittatus var. namaquensis 87

sagittatus var. parviflorus 98

sagittatus var. parviflorus subvar. linearifolius 94

sagittatus var. parviflorus subvar. villosus 100

sagittatus var. phyllosepalus 99

sagittatus var. subcordata 98

sagittatus var. ulosepalus 94

sagittatus var. villosus 100

sagittifolius 60, 63, 67, 104, 252

salonifolius 253

salvifolius 239

sarmentosus 20, 24, 26, 28, 34, 47, 169, 171, 180, 181

sarothrocladus 20, 27, 51, 188, 191

saxatilis 135, 210, 211, 227

saxatilis var. sericeus 227

saxatilis var. villosus 227

scammonia 12, 14, 20, 22, 23, 24, 25, 37, 40, 42, 47, 52, 57-59, 73

scammonia var. pseudoscammonia 59

schimperi 19, 22, 24, 26, 36, 83, 239, 241, 242, 244, 254

schimperi var. ellipticus 254

schirazianus 22, 23, 27, 49, 207, 208, 212

schulzei 29, 110

schweinfurthii 96

scindicus 20, 23, 49, 174, 250, 251

scoparia 231, 232

scoparium 232

scoparius 13, 20, 25-27, 38, 225, 231, 232

scoparius var. virgatus 232

Convolvulus scopulatus 20, 22, 26, 27, 34, 46, 157, 165, 167, 168

secundus 22, 24, 26, 43, 238, 239, 243-245, 254

secundus var. latifolius 239

secundifolius var. parvifolius 239

segobricensis 61

semhaensis 15, 19, 21, 23, 25, 26, 33, 45, 155, 169

sepium 252, 254

sericeus 81, 245

sericocephalus 13, 22, 24, 27, 41, 53, 195, 208, 212

sericophyllus 20, 21, 23, 24, 28, 34, 46, 157, 179

sibthorpii 69

siculus 20, 25, 28, 33, 35, 36, 38, 39, 42, 44, 47, 147-151

siculus subsp. agrestis 150

siculus subsp. elongatus 36, 39, 44, 149

siculus subsp. siculus 35, 38, 39, 44, 147, 148

siculus var. flexuosus 149

siculus var. major 149

simulans 15, 20, 25, 28, 30, 103, 144, 155

sintenisii 75

sinuatodentatus 20, 54, 66, 67

sistanicus 177,

socotranus 15, 20, 23, 25, 34, 46, 155, 157, 168

sogdianus 177

solanifolius 136

soldanella 254

somalensis 179

sphaerophorus 158

spicatus 19, 22, 26, 36, 43, 239, 241, 242, 244

spicifolius 217

spinifer 21, 23, 27, 52, 55, 197-199

spinosus 21, 24, 27, 48, 166, 189, 198, 199, 201, 202

spinosus var. kermanensis 189

spinosus var. subinermis 189

spithameus 254

sprengelii 96

stachydifolius 20, 38, 43, 48, 68, 71, 72, 75

stachydifolius var. villosus 72

stapfii 22, 24, 27, 49, 240, 249

Convolvulus stenocladus 20, 22, 28, 34, 157, 158, 160

stenophyllus 72, 73

steppicola 20, 23, 54, 65, 66

steudneri 97-100

stocksii 173

strictus 154

strigulosus 224

subacaulis 254

subauriculatus 139

subhirsutus 230

subpinnatifidus 126

subsericeus 17, 20, 27, 53, 185-187, 189

subsericeus subsp. hamadae 185

subspathulatus 20, 21, 25, 26, 34, 161

suendermannii 20, 21, 27, 41, 195, 216, 217

suffruticosus 13, 135, 136, 141, 145, 146, 254

suffruticosus var. sulfureus 145

supinus var. atrichogynus 145

supinus var. melliflorus 145

supinus var. sulphurescens 145

supinus var. supinus 145

tauricus 210, 211

tedmoorei 31, 123

tenellus 173

tenuissimus 81

terminalis 205

terrestris 205

thomsonii 21, 23, 24, 35, 82, 94, 95, 100, 101

thunbergii 24, 33, 84, 87

thymoides 98, 100

tomentellus 254

tomentosus 69

tournefortii 254

trabutianus 21, 26, 36, 169-172

tragacanthoides 20, 21, 27, 52, 55, 197-199

translucens 254

transvaalensis 87, 88

tricolor 13, 20, 24, 25, 28, 36, 38, 40, 42, 144, 151-153

tricolor subsp. cupanianus 36, 40, 153, 252

tricolor subsp. cupanianus var. guttatus 153

tricolor subsp. cupanianus var. hortensis

tricolor subsp. meonanthus 151

tricolor subsp. pentapetaloides 151

Convolvulus tricolor subsp. tricolor 36, 40, 144, 152

tricolor var. cupanianus 153

tricolor var. heterocalyx 153

tricolor var. hortensis 152, 153

tricolor var. maroccanus 153

tricolor var. pseudotricolor 152

tricolor var. quadricolor 153

triflorus 107

triqueter 211

tschimganicus 54, 231

tshegemensis 217, 218

tujuntauensis 20, 27, 28, 54, 187

turcicus 215

turcomanicus 189, 190, 207

turcomanicus var. villosus 254

turrillianus 21, 22, 26, 27, 48, 49, 203, 205

ulicinus 21, 22, 26, 27, 45, 243, 250

ulosepalus 94, 95

undulatus 154, 254

undulifolius 245

undulifolius var. secundus 245

urosepalus 22, 24, 27, 48, 203, 204

vahlii 113

valentinus 13, 17, 20, 25, 26, 37, 39, 134, 141, 145, 146, 254

valentinus subsp. suffruticosus var. adpressipilis 146

valentinus subsp. suffruticosus var. simulans 146

valentinus subsp. suffruticosus var. sulfureus 146

valentinus subsp. suffruticosus var. transiens 146

valentinus var. adpressipilis 146

valentinus var. melliflorus 145, 146

valentinus var. melillensis 141

valentinus var. oranensis 141

valentinus var. simulans 146

valentinus var. suffruticosus 254

valentinus var. sulfureus 146

valentinus var. transiens 146

variegatus 96

venosus 139

verdcourtianus 21, 24, 28, 33, 169, 170

verecundus 24, 31, 128

verecundus subsp. waitaha 128

Convolvulus vidalii 20, 23, 24, 28, 38, 76, 77, 78

villiflorus 206

virgatus 21, 22, 25, 45, 48, 148, 161, 162

virgatus var. subaphyllus 162

vollesenii 20, 21, 34, 161

volubilis 20, 24, 25, 26, 39, 137, 138, 141, 163, 252

waitaha 22, 24, 25, 28, 31, 128

wallichianus 254

waltherioides 248

wimmerensis 31, 124

xanthopotamicus 18, 21, 23, 27, 28, 54, 188, 195, 196

zargarianus 163, 164

zernyi 100, 101

Evolvulus agrestis 149, 150

ferrugineus 254

pilosus 173

virgatus 179

Hewittia kilimandschari 83

Ipomoea auricoma 163

cordobana 107

glaucifolia 253

hermanniae 112

Ipomoea huillensis 97

microphylla 173

montevidensis 116

ottoensis 116

polymorpha var. glabra 111

Nemostima canariensis 136

cantabrica 205

Periphas pannifolius 136

Retzia pilosa 254

Rhodorrhiza glandulosa 140

florida 233

florida var. angustifolia 233

florida var. genuina 233

perraudieri 139

scoparia 232

subauriculata 139

virgata 232

volubilis 137

Rhodoxylon floridum 233

scoparium 232

Seddera semhaensis 169

spinosa 168

virgata 253

Symethus siculus 147
